# Supplementary material for: Cobalt-catalyzed atroposelective C−H activation/annulation to access N−N axially chiral frameworks
Source: Nat Commun. 2023 Aug 29;14:5271. doi: 10.1038/s41467-023-40978-4 (PMC10465517; doi:10.1038/s41467-023-40978-4)
Supplement: Supplementary file 1 — Supplementary Information [file 41467_2023_40978_MOESM1_ESM.pdf]

Supplementary Information

Cobalt-Catalyzed Atroposelective C−H Activation/Annulation to Access N−N Axially Chiral Frameworks

Tong Li,[1,2] Linlin Shi,[1,2] Xinhai Wang,[1] Chen Yang,[1] Dandan Yang,[1]* Mao-Ping Song,[1] and Jun-Long Niu[1]*

[1]College of Chemistry, Zhengzhou University, Zhengzhou 450001 (P. R. China).

[2]These authors contributed equally to this work: Tong Li, Linlin Shi.

*Corresponding author. Email: yangdandan@zzu.edu.cn; niujunlong@zzu.edu.cn

Table of Contents

1. **General Information** **S3**
2. **Optimization of reaction conditions** **S4**
3. **General procedure for the synthesis of substrates** **S10**
4. **General procedure for synthesis of products 3** **S17**
5. **Mechanistic studies** **S18**
6. **Synthetic applications** **S21**
7. **Study on products stabilities** **S27**
8. **The nonlinear effect studies** **S30**
9. **X-ray crystal structure of 3aa** **S31**
10. **Computational studies** **S33**
11. **Characterization data and HPLC chromatograms** **S34**
12. **NMR spectra for new compounds** **S59**
13. **Supplementary references** **S139**

1. General Information

All materials were commercially obtained and used without further purification. ^{1}H NMR, ^{13}C NMR and ^{19}F NMR ^{31}P NMR spectra were recorded at 600 MHz, 151 MHz, 565 MHz, and 243 MHz respectively on a Bruker DPX instrument using Me_4Si as an internal standard. Chemical shift multiplicities are reported as follows: (s = singlet, d = doublet, t = triplet, q = quartet, m = multiplet, br = broad, dd = doublet of doublet, dt = doublet of triplet, td = triplet of doublet)., The conversion of starting materials was monitored by thin layer chromatography (TLC) using silica gel plates (silica gel 60 F254 0.25 mm), and components were visualized by observation under UV light (254 and 365 nm). Column chromatography was performed on silica gel 200-300 mesh. The enantiomeric excess (ee) of the products were determined by high-performance liquid chromatography (HPLC) with a chiral stationary phase in comparison with the authentic racemate sample. The absolute configuration of **3ba** was assigned by the X-ray analysis.

2. Optimization of reaction conditions

Supplementary Table 1. Optimization of ligand[a]

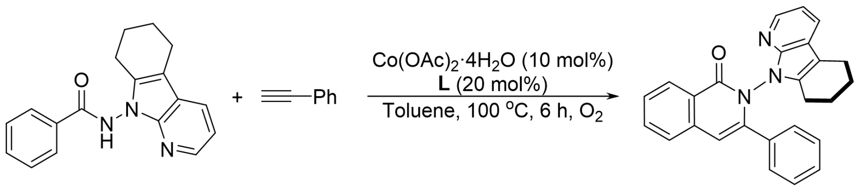

1a 2a 3aa

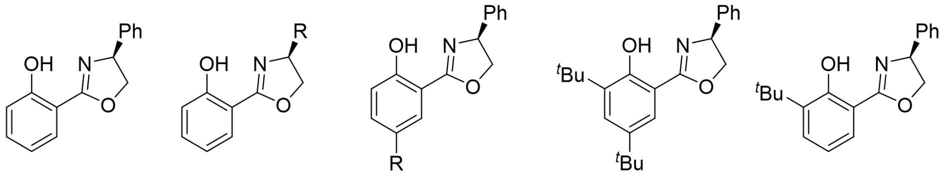

L1 15%, 99% ee; L2, R = Bn, N.R; L3, R = ^{t}Bu, N.R; L4, R = OMe, 15%, 99% ee; L5, R = F, 5%, 99% ee; L6 48%, 99% ee; L7 42%, 98% ee

Entry	Ligand	Yield (%)	ee (%)
1	**L1**	15	99
2	**L2**	N.R.[b]	-
3	**L3**	N.R.	-
4	**L4**	15	99
5	**L5**	5	99
**6**	**L6**	**48**	**99**
7	**L7**	42	98

[a]Unless otherwise mentioned, all reactions were carried out using **1a** (0.1 mmol), **2a** (0.12 mmol), $Co(OAc)_2·4H_2O$ (0.01 mmol), Ligand (0.02 mmol) in toluene (1 mL) at 100 °C under O_2 for 6 h, isolated yield. [b]No reaction.

Supplementary Table 2. Optimization of cobalt salts[a]

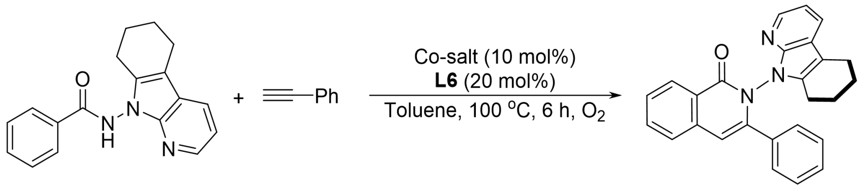

1a 2a 3aa

Entry	Co-salt	Yield (%)	ee (%)
**1**	**$Co(OAc)_2·4H_2O$**	**48**	**99**
2	$Co(OAc)_2$	48	98
3	$Co(OBz)_2$	45	98
4	$Co(C_2O_4)_2·4H_2O$	N.R.[b]	-
5	$CoSO_4·H_2O$	N.R.	-
6	$CoCl_2·6H_2O$	N.R.	-

[a]Unless otherwise mentioned, all reactions were carried out using **1a** (0.1 mmol), **2a** (0.12 mmol), Co-salt (0.01 mmol), **L6** (0.02 mmol) in toluene (1 mL) at 100 °C under O_2 for 6 h, isolated yield. [b]No reaction.

Supplementary Table 3. Optimization of solvent[a]

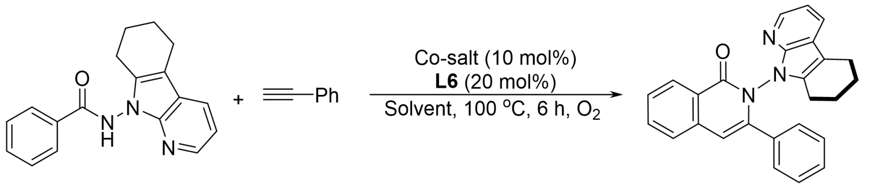

1a 2a 3aa

Entry	Solvent	Yield (%)	ee (%)
1	Toluene	48	99
**2**	**1,4-Dioxane**	**68**	**98**
3	THF	42	98
4	DME	43	98
5	CPME	57	98
6	PhOMe	N.R.[b]	-

[a]Unless otherwise mentioned, all reactions were carried out using **1a** (0.1 mmol), **2a** (0.12 mmol), $Co(OAc)_2 \cdot 4H_2O$ (0.01 mmol), **L6** (0.02 mmol) in solvent (1 mL) at 100 °C under O_2 for 6 h, isolated yield. [b]No reaction.

Supplementary Table 4. Optimization of additive[a]

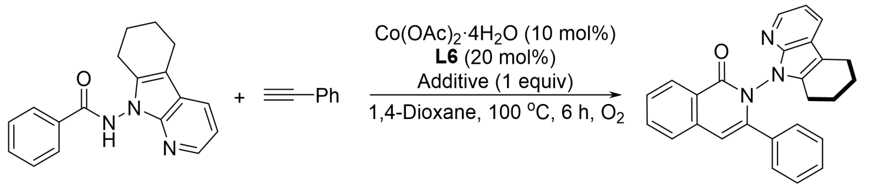

1a 2a 3aa

Entry	Additive	Yield (%)	ee (%)
1	$NaOPiv \cdot H_2O$	72	98
2	Na_2CO_3	49	98
3	PhCOONa	52	98
4	DBU	N.R.[b]	-
**5**	**AdCOOH**	**94**	**98**
6	AcOH	69	98

[a]Unless otherwise mentioned, all reactions were carried out using **1a** (0.1 mmol), **2a** (0.12 mmol), $Co(OAc)_2 \cdot 4H_2O$ (0.01 mmol), **L6** (0.02 mmol), additive (0.1 mmol), in 1,4-dioxane (1 mL) under O_2 for 6 h, isolated yield. [b]No reaction.

Supplementary Table 5. Optimization of temperature[a]

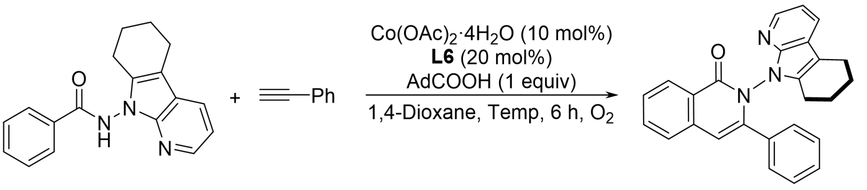

1a 2a 3aa

Entry	Temp (°C)	Yield (%)	ee (%)
1	60	46	98
**2**	**80**	**94**	**98**
3	100	94	98

[a]Unless otherwise mentioned, all reactions were carried out using **1a** (0.1 mmol), **2a** (0.12 mmol), $Co(OAc)_2 \cdot 4H_2O$ (0.01 mmol), **L6** (0.02 mmol), AdCOOH (0.1 mmol) in 1,4-dioxane (1 mL) under O_2 for 6 h, isolated yield.

Supplementary Table 6. Optimization of reaction time[a]

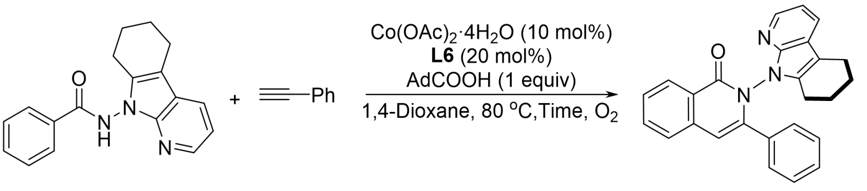

1a 2a 3aa

Entry	Time (h)	Yield (%)	ee (%)
1	4	89	98
**2**	**6**	**94**	**98**
3	8	92	98
4	10	89	98

[a]Unless otherwise mentioned, all reactions were carried out using **1a** (0.1 mmol), **2a** (0.12 mmol), $Co(OAc)_2 \cdot 4H_2O$ (0.01 mmol), **L6** (0.02 mmol), AdCOOH (0.1 mmol)in 1,4-dioxane (1 mL) at 80 °C under O_2, isolated yield.

Supplementary Table 7. Optimization of the amount of 1,4-Dioxane[a]

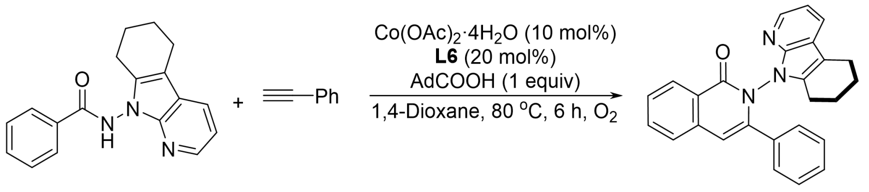

1a **2a** **3aa**

Entry	1,4-Dioxane	Yield (%)	ee (%)
1	0.5 ml	86	98
**2**	**1 ml**	**94**	**98**
3	2 ml	70	98
4[b]	1 ml	67	98

[a]Unless otherwise mentioned, all reactions were carried out using **1a** (0.1 mmol), **2a** (0.12 mmol), $Co(OAc)_2 \cdot 4H_2O$ (0.01 mmol), **L6** (0.02 mmol), AdCOOH (1 equiv)in 1,4-dioxane (x mL) at 80 °C under O_2 for 6 h, isolated yield. [b]Under air.

Supplementary Table 8. Optimization of the amounts of cobalt salt and ligand[a]

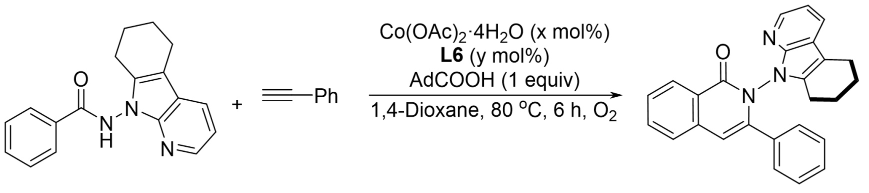

1a **2a** **3aa**

Entry	x	y	Yield (%)[b]	ee (%)
1	5	10	75	98
2	10	10	85	98
3	10	15	88	98
**4**	**10**	**20**	**94**	**98**

[a]Unless otherwise mentioned, all reactions were carried out using **1a** (0.1 mmol), **2a** (0.12 mmol), $Co(OAc)_2 \cdot 4H_2O$ (x mol%), **L6** (y mol%), AdCOOH (0.1 mmol) in 1,4-dioxane (1 mL) at 80 °C under O_2 for 6 h, isolated yield.

Expand the reaction scale to 0.2 mmol[a]

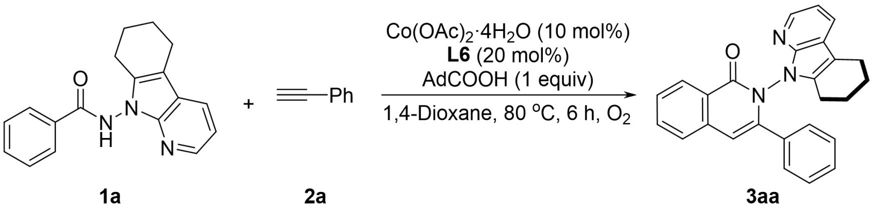

1a **0.2 mmol** **2a** **0.24 mmol** **3aa** **90%, 98% ee**

[a]Reaction conditions: **1a** (0.2 mmol), **2a** (0.24 mmol), $Co(OAc)_2 \cdot 4H_2O$ (0.02 mmol), **L6** (0.04 mmol), AdCOOH (0.2 mmol) in 1,4-dioxane (2 mL) at 80 °C under O_2 for 6 h, isolated yield.

Supplementary Table 9. Optimization of electrochemical reaction conditions

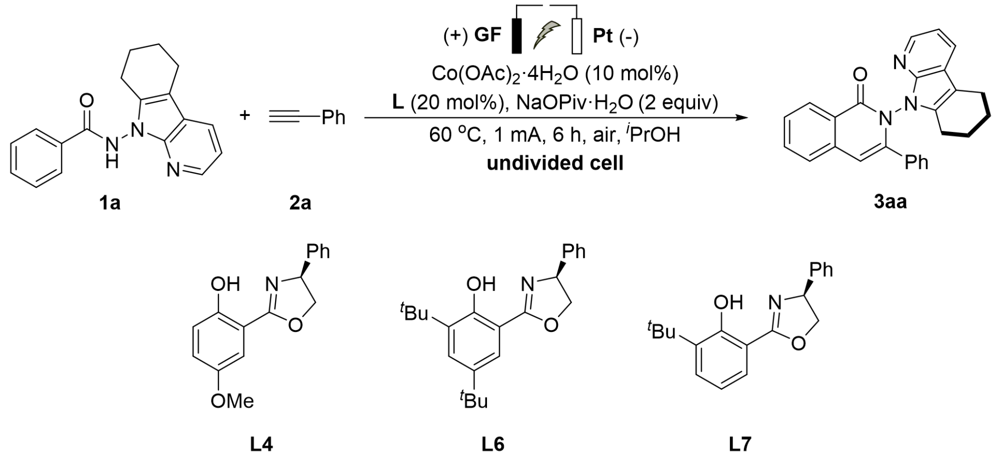

Entry	Ligand	Solvent	Current	Yield (%)	ee (%)
1	**L4**	^{i}PrOH	1mA	trace	-
2	**L6**	^{i}PrOH	1mA	21	98
3	**L7**	^{i}PrOH	1mA	32	96
4	**L7**	^{i}PrOH	2mA	34	95
5	**L7**	EtOH	2mA	33	98
6	**L7**	2-Butanol	2mA	26	92
7	**L7**	TFE	2mA	62	98
8[b]	**L7**	TFE	2mA	N.R.[e]	-
9[c]	**L7**	TFE	2mA	65	98
**10[c, d]**	**L7**	**TFE**	**2mA**	**69**	**98**
11[c, d]	**L7**	TFE	-	N.R.	-

[a]Unless otherwise mentioned, all reactions were carried out using raphite felt (10 mm × 15 mm × 6 mm) anode,Pt-plate (10 mm × 10 mm × 0.1 mm) cathode, constant current = 2 mA, **1a** (0.2 mmol), **2a** (1.5 equiv), $Co(OAc)_2·4H_2O$ (10 mol%), **L** (20 mol%), $NaOPiv·H_2O$ (2 equiv) in 10 mL of solvent at 60 °C under 1mA for 6 h, isolated yield. [b]None of $NaOPiv·H_2O$. [c]5 mL of solvent. [d]**2a** (1.2 equiv). [e]No reaction.

Supplementary Table 10. Competitive experiment

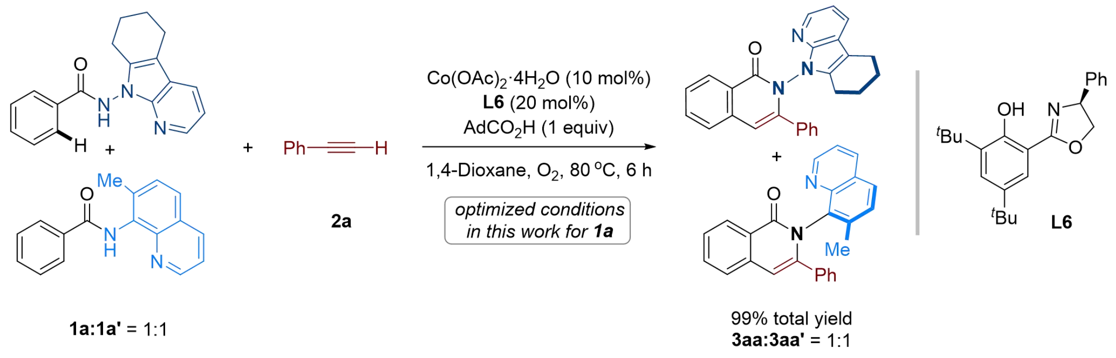

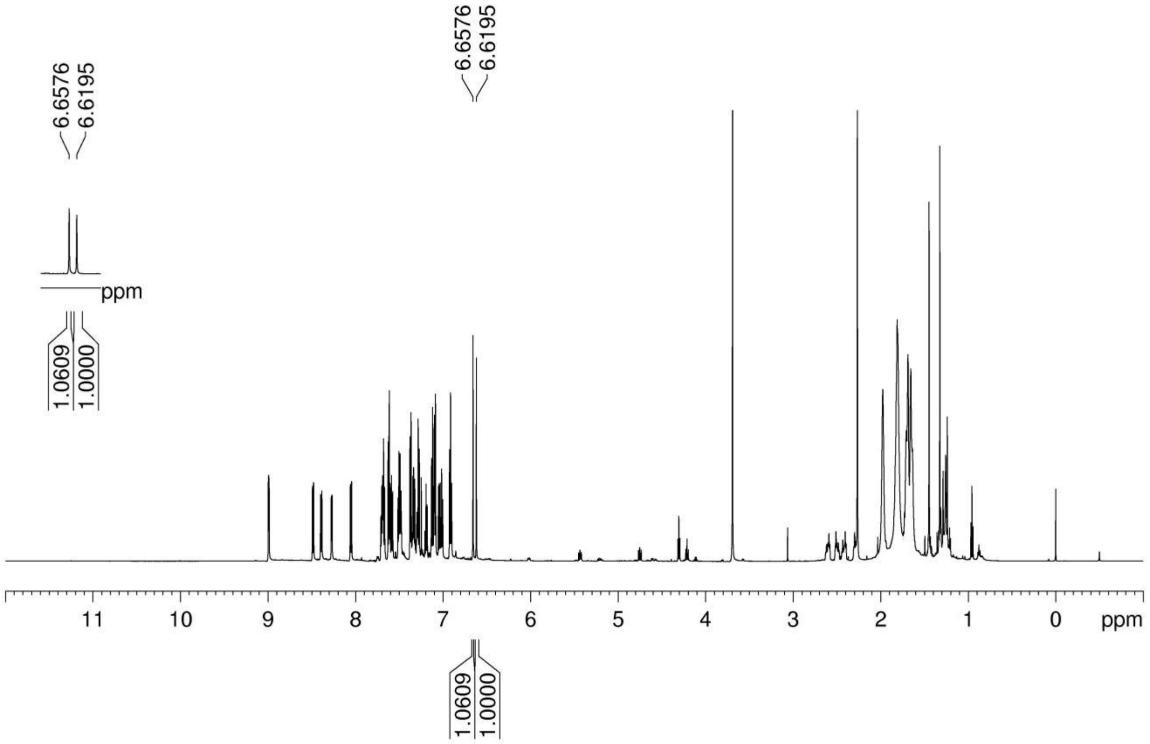
6.6576
6.6195
ppm
1.0609
1.0000
11
10
9
8
7
6
5
4
3
2
1
0
ppm

3. General procedure for the synthesis of substrates

3.1 General procedure for the synthesis of benzamides

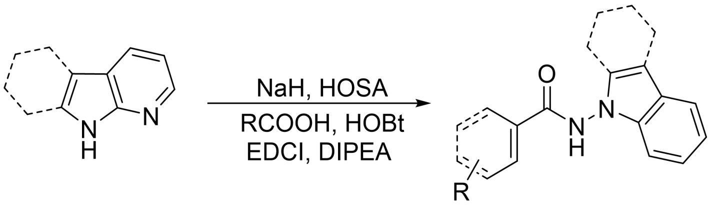

7-azindoles derivatives were synthesized according to literature procedures.[1]

To a stirred solution of NaH (10 equiv) in dry DMF was added a solution of 7-azindoles derivatives (3 mmol) in dry DMF dropwise at 0 ºC. Kept the reaction mixture stirred at 0 ºC for 30 min. Then, hydroxylamine-O-sulfonic acid (5 equiv) was slowly added at 0 ºC, and the mixture was stirred overnight at room temperature. Upon completion of the reaction by TLC, the mixture was quenched with H_2O and extracted with EA for three times. These extracts were dried over Na_2SO_4 and concentrated *in vacuo.* The residue was used in next step without further purification.

A solution of benzoic acid (1.3 equiv), *N*-amino-7-azaindole derivative, HOBt (1.2 equiv), EDCI (1.2 equiv) and DIPEA (2.5 equiv) in DMF was stirred at room temperature for 12 h. The mixture was quenched with saturated $NaHCO_3$ solution and extracted with EA for three times. These extracts were combined and dried over Na_2SO_4. The residue was purified by flash column chromatography on silica gel to give the amides.

N-(5,6,7,8-tetrahydro-9H-pyrido[2,3-b]indol-9-yl)benzamide (1a)

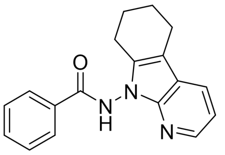

Yield:401.6 mg (46%). White solid, mp: 241-242 ˚C. **^{1}H NMR** ^{1}H NMR (600 MHz, $CDCl_3$) δ 12.62 (s, 1H), 8.14 – 8.13 (m, 1H), 7.92 (d, *J* = 7.4 Hz, 2H), 7.74 (dd, *J* = 7.7, 1.1 Hz, 1H), 7.38 (t, *J* = 7.4 Hz, 1H), 7.22 (t, *J* = 7.8 Hz, 2H), 7.05 (dd, *J* = 7.7, 4.9 Hz, 1H), 2.65 (d, *J* = 6.2 Hz, 4H), 1.90 – 1.85 (m, 4H). **^{13}C NMR** (151 MHz, $CDCl_3$) δ 166.8, 147.0, 140.1, 138.2, 132.0, 131.0, 128.4, 127.7, 126.6, 119.8, 116.2, 107.3, 22.8, 22.4, 21.0, 20.5. **HRMS** (ESI): m/z $[M+H]^+$ calcd for $[C_{18}H_{18}N_3O]^+$ required 292.1445, found 292.1448.

4-fluoro-N-(5,6,7,8-tetrahydro-9H-pyrido[2,3-b]indol-9-yl)benzamide (1b)

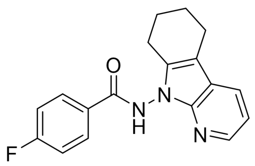

Yield: 215.7 mg (24%). White solid, mp: 236-237 ˚C. **^{1}H NMR** ^{1}H NMR (600 MHz, $CDCl_3$) δ 13.01 (s, 1H), 8.12 (d, *J* = 3.9 Hz, 1H), 7.99 (dd, *J* = 8.7, 5.3 Hz, 2H), 7.76 (dd, *J* = 7.7, 1.0 Hz, 1H), 7.06 (dd, *J* = 7.7, 4.9 Hz, 1H), 6.89 (t, *J* = 8.6 Hz, 2H), 2.65 (s, 4H), 1.88 (dd, *J* = 29.2, 5.0 Hz, 4H). **^{13}C NMR** (151 MHz, $CDCl_3$) δ 165.9, 165.6, 164.2, 146.9, 139.9, 138.2, 130.2 ($^3J_{C-F}$ = 8.9 Hz), 127.1, 126.9, 120.0, 116.3, 115.5 ($^2J_{C-F}$ = 21.9 Hz), 107.5, 22.8, 22.4, 21.0, 20.5. **^{19}F NMR** (565 MHz, $CDCl_3$) δ -107.1. **HRMS** (ESI): m/z $[M+H]^+$ calcd for $[C_{18}H_{17}FN_3O]^+$ required 310.1350, found 310.1357.

4-chloro-N-(5,6,7,8-tetrahydro-9H-pyrido[2,3-b]indol-9-yl)benzamide (1c)

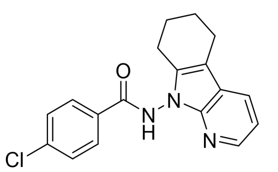

Yield: 350.9 mg (36%). White solid, mp: 221-222 ˚C. **^{1}H NMR** ^{1}H NMR (600 MHz, $CDCl_3$) δ 13.12 (s, 1H), 8.12 – 8.11 (m, 1H), 7.88 (d, *J* = 8.6 Hz, 2H), 7.75 (dd, *J* = 7.7, 1.2 Hz, 1H), 7.18 (d, *J* = 8.6 Hz, 2H), 7.06 (dd, *J* = 7.7, 4.9 Hz, 1H), 2.64 (d, *J* = 5.2 Hz, 4H), 1.88 (dd, *J* = 29.6, 4.9 Hz, 4H). **^{13}C NMR** (151 MHz, $CDCl_3$) δ 165.6, 146.8, 139.8, 138.4, 138.2, 129.2, 129.1, 128.6, 127.0, 120.0, 116.3, 107.5, 22.8, 22.4, 21.0, 20.5. **HRMS** (ESI): m/z $[M+H]^+$ calcd for $[C_{18}H_{17}ClN_3O]^+$ required 326.1055, found 326.1056.

4-bromo-N-(5,6,7,8-tetrahydro-9H-pyrido[2,3-b]indol-9-yl)benzamide (1d)

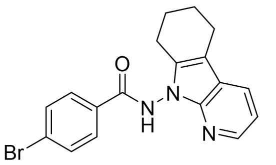

Yield: 332.3 mg (30%). White solid, mp: 269-270 ˚C. **^{1}H NMR** ^{1}H NMR (600 MHz, $CDCl_3$) δ 13.12 (s, 1H), 8.12 (d, *J* = 4.0 Hz, 1H), 7.80 (d, *J* = 8.5 Hz, 2H), 7.75 (dd, *J* = 7.7, 1.0 Hz, 1H), 7.35 (d, *J* = 8.5 Hz, 2H), 7.06 (dd, *J* = 7.7, 4.9 Hz, 1H), 2.64 (t, *J* = 5.3 Hz, 4H), 1.88 (dd, *J* = 29.8, 4.6 Hz, 4H). **^{13}C NMR** (151 MHz, $CDCl_3$) δ 165.8, 146.8, 139.8, 138.2, 131.6, 129.6, 129.2, 127.1, 127.0, 120.0, 116.3, 107.5, 22.8, 22.4, 21.0, 20.5. **HRMS** (ESI): m/z $[M+H]^+$ calcd for $[C_{18}H_{17}N_2O]^+$ required 370.0550, found 370.0552.

N-(5,6,7,8-tetrahydro-9H-pyrido[2,3-b]indol-9-yl)-4-(trifluoromethyl)benzamide (1e)

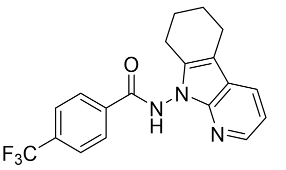

Yield: 279.3 mg (26%). White solid, mp: 270-271 ˚C. **^{1}H NMR** ^{1}H NMR (600 MHz, $CDCl_3$) δ 13.64 (s, 1H), 8.14 (d, *J* = 4.3 Hz, 1H), 8.02 (d, *J* = 8.2 Hz, 2H), 7.78 (d, *J* = 7.2 Hz, 1H), 7.41 (d, *J* = 8.3 Hz, 2H), 7.09 (dd, *J* = 7.7, 4.9 Hz, 1H), 2.66 (s, 4H), 1.89 (dd, *J* = 30.9, 4.7 Hz, 4H). **^{13}C NMR** (151 MHz, $CDCl_3$) δ 165.1, 146.8, 139.6, 138.2, 133.7, 133.4 ($^{2}J_{C\text{-}F}$ = 32.2 Hz), 128.0, 127.2, 125.4 ($^{1}J_{C\text{-}F}$ = 3.4 Hz), 123.6 ($^{3}J_{C\text{-}F}$ = 272.8 Hz), 120.8, 120.2, 116.4, 107.8, 22.8, 22.4, 21.0, 20.5. **^{19}F NMR** (565 MHz, $CDCl_3$) δ -63.3. **HRMS** (ESI): m/z $[M+H]^+$ calcd for $[C_{19}H_{17}F_3N_3O]^+$ required 360.1318, found 360.1328.

4-methoxy-N-(5,6,7,8-tetrahydro-9H-pyrido[2,3-b]indol-9-yl)benzamide (1f)

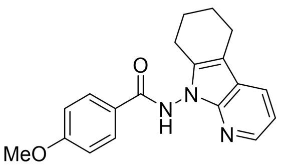

Yield: 289.7 mg (30%). White solid, mp: 221-222 ˚C. **^{1}H NMR** ^{1}H NMR (600 MHz, $CDCl_3$) δ 12.60 (s, 1H), 8.13 (d, *J* = 4.7 Hz, 1H), 7.92 (d, *J* = 8.7 Hz, 2H), 7.73 (d, *J* = 7.7 Hz, 1H), 7.03 (dd, *J* = 7.6, 4.9 Hz, 1H), 6.69 (d, *J* = 8.7 Hz, 2H), 3.79 (s, 3H), 2.66 – 2.64 (m, 4H), 1.87 (dd, *J* = 28.1, 5.2 Hz, 4H). **^{13}C NMR** (151 MHz, $CDCl_3$) δ 166.4, 162.5, 147.0, 140.0, 138.3, 129.6, 126.6, 123.5, 119.8, 116.1, 113.6, 107.2, 55.4, 22.9, 22.5, 21.1, 20.5. **HRMS** (ESI): m/z $[M+H]^+$ calcd for $[C_{19}H_{20}N_3O_2]^+$ required 322.1550, found 322.1550.

4-ethyl-N-(5,6,7,8-tetrahydro-9H-pyrido[2,3-b]indol-9-yl)benzamide (1g)

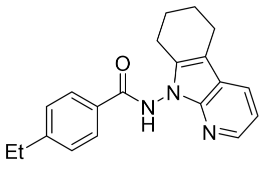

Yield: 220.2 mg (23%). White solid, mp: 224-125 ˚C. **^{1}H NMR** ^{1}H NMR (600 MHz, $CDCl_3$) δ 13.16 (s, 1H), 8.14 – 8.13 (m, 1H), 7.85 – 7.83 (m, 2H), 7.72 (d, *J* = 7.7 Hz, 1H), 7.03 (dd, *J* = 7.7, 4.9 Hz, 1H), 6.99 (d, *J* = 8.2 Hz, 2H), 2.65 (m, 4H), 2.60 (q, *J* = 7.6 Hz, 2H), 1.88 (dd, *J* = 28.1, 5.0 Hz, 4H), 1.24 – 1.21 (m, 3H). **^{13}C NMR** (151 MHz, $CDCl_3$) δ 166.7, 148.4, 147.0, 139.8, 138.4, 128.3, 127.8, 127.8, 126.6, 119.9, 116.1, 107.2, 28.9, 22.9, 22.5, 21.1, 20.5, 15.4. **HRMS** (ESI): m/z $[M+H]^+$ calcd for $[C_{20}H_{22}N_3O]^+$ required 320.1758, found 320.1758.

4-(tert-butyl)-N-(5,6,7,8-tetrahydro-9H-pyrido[2,3-b]indol-9-yl)benzamide (1h)

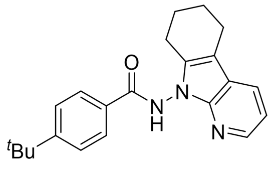

Yield: 405.6 mg (39%). White solid, mp: 241-242 ˚C. **^{1}H NMR** ^{1}H NMR (600 MHz, $CDCl_3$) δ 13.35 (d, *J* = 26.9 Hz, 1H), 8.12 (d, *J* = 4.4 Hz, 1H), 7.86 – 7.85 (m, 2H), 7.74 (d, *J* = 7.7 Hz, 1H), 7.15 (dd, *J* = 8.4, 4.9 Hz, 2H), 7.04 (dd, *J* = 7.6, 5.0 Hz, 1H), 2.66 – 2.65 (m, 4H), 1.88 (d, *J* = 27.3 Hz, 4H), 1.29 (s, 9H). **^{13}C NMR** (151 MHz, $CDCl_3$) δ 166.5, 155.0,

147.0, 139.9, 138.4, 127.9, 127.5, 126.7, 125.2, 119.9, 116.1, 107.2, 34.9, 31.1, 22.9, 22.5, 21.1, 20.5. **HRMS** (ESI): m/z $[M+H]^+$ calcd for $[C_{22}H_{26}N_3O]^+$ required 348.2071, found 348.2073.

N-(5,6,7,8-tetrahydro-9H-pyrido[2,3-b]indol-9-yl)-[1,1'-biphenyl]-4-carboxamide (1i)

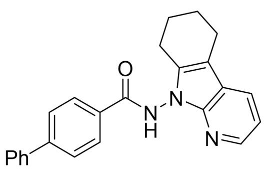

Yield: 451.4 mg (41%). White solid, mp: 226-227 ˚C. **^{1}H NMR** ^{1}H NMR (600 MHz, $CDCl_3$) δ 13.34 (s, 1H), 8.19 – 8.18 (m, 1H), 8.02 (dd, *J* = 8.2, 4.4 Hz, 2H), 7.76 (dd, *J* = 7.7, 1.2 Hz, 1H), 7.54 (d, *J* = 7.8 Hz, 2H), 7.44 (t, *J* = 7.6 Hz, 2H), 7.37 (dd, *J* = 11.7, 7.9 Hz, 3H), 7.07 (dd, *J* = 7.8, 4.9 Hz, 1H), 2.69 (d, *J* = 28.8 Hz, 4H), 1.91 (dd, *J* = 31.1, 4.3 Hz, 4H). **^{13}C NMR** (151 MHz, $CDCl_3$) δ 166.5, 147.0, 144.5, 140.1, 139.9, 138.4, 129.5, 128.9, 128.2, 127.9, 127.2, 126.9, 126.8, 120.0, 116.2, 107.3, 22.9, 22.5, 21.1, 20.5. **HRMS** (ESI): m/z $[M+H]^+$ calcd for $[C_{24}H_{22}N_3O]^+$ required 368.1758, found 368.1761.

3-bromo-N-(5,6,7,8-tetrahydro-9H-pyrido[2,3-b]indol-9-yl)benzamide (1j)

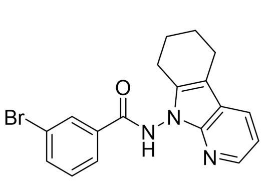

Yield: 219.9 mg (20%). White solid, mp: 223-224 ˚C. **^{1}H NMR** ^{1}H NMR (600 MHz, $CDCl_3$) δ 13.37 (s, 1H), 8.15 (d, *J* = 4.7 Hz, 1H), 8.00 (d, *J* = 7.9 Hz, 1H), 7.97 (s, 1H), 7.75 (d, *J* = 7.7 Hz, 1H), 7.54 (d, *J* = 7.9 Hz, 1H), 7.10 – 7.05 (m, 2H), 2.64 (s, 4H), 1.87 (dd, *J* = 30.9, 4.7 Hz, 4H). **^{13}C NMR** (151 MHz, $CDCl_3$) δ 165.2, 146.9, 139.7, 138.2, 134.9, 132.6, 131.3, 129.9, 127.0, 125.6, 122.9, 120.1, 116.3, 107.6, 22.8, 22.4, 21.0, 20.5. **HRMS** (ESI): m/z $[M+H]^+$ calcd for $[C_{18}H_{17}BrN_3O]^+$ required 370.0550, found 370.0558.

2-chloro-N-(5,6,7,8-tetrahydro-9H-pyrido[2,3-b]indol-9-yl)benzamide (1k)

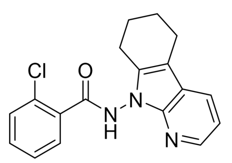

Yield: 253.5 mg (26%). White solid, mp: 216-217 ˚C. **^{1}H NMR** ^{1}H NMR (600 MHz, $CDCl_3$) δ 10.32 (s, 1H), 8.08 – 8.07 (m, 1H), 7.67 – 7.63 (m, 2H), 7.40 (d, *J* = 7.8 Hz, 1H), 7.35 (td, *J* = 7.8, 1.4 Hz, 1H), 7.22 (t, *J* = 7.1 Hz, 1H), 6.98 (dd, *J* = 7.7, 4.9 Hz, 1H), 2.73 (t, *J* = 5.8 Hz, 2H), 2.64 (t, *J* = 5.7 Hz, 2H), 1.93 (dt, *J* = 12.1, 6.1 Hz, 2H), 1.87 (dd, *J* = 11.2, 5.6 Hz, 2H). **^{13}C NMR** (151 MHz, $CDCl_3$) δ 166.4, 147.1, 141.6, 137.5, 132.8, 131.8, 131.3, 130.3, 130.2, 127.0, 126.2, 119.5, 116.5, 107.8, 22.8, 22.5, 21.3, 20.5. **HRMS** (ESI): m/z $[M+H]^+$ calcd for $[C_{18}H_{17}ClN_3O]^+$ required 326.1055, found 326.1060.

2-methyl-N-(5,6,7,8-tetrahydro-9H-pyrido[2,3-b]indol-9-yl)benzamide (1l)

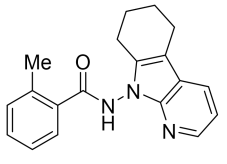

Yield: 328.9 mg (36%). White solid, mp:177-178 ˚C. **^{1}H NMR** ^{1}H NMR (600 MHz, $CDCl_3$) δ 10.27 (s, 1H), 8.12 (d, *J* = 4.6 Hz, 1H), 7.71 (t, *J* = 7.1 Hz, 1H), 7.69 (dd, *J* = 7.7, 1.0 Hz, 1H), 7.34 (t, *J* = 7.1 Hz, 1H), 7.22 – 7.18 (m, 2H), 7.01 (dd, *J* = 7.7, 4.8 Hz, 1H), 2.63 (d, *J* = 6.0 Hz, 4H), 2.36 (s, 3H), 1.87 (dd, *J* = 28.9, 5.3 Hz, 4H). **^{13}C NMR** (151 MHz, $CDCl_3$) δ 169.3, 147.2, 141.3, 137.9, 137.4, 132.5, 131.4, 130.9, 127.4, 126.2, 119.4, 116.4, 107.5, 22.8, 22.5, 21.0, 20.5, 20.2. **HRMS** (ESI): m/z $[M+H]^+$ calcd for $[C_{19}H_{20}N_3O]^+$ required 306.1601, found 306.1605.

3,5-dimethyl-N-(5,6,7,8-tetrahydro-9H-pyrido[2,3-b]indol-9-yl)benzamide (1m)

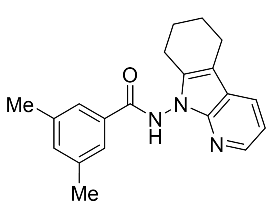

Yield: 134.6 mg (20%). White solid, mp: 233-234 ˚C. **^{1}H NMR** ^{1}H NMR (600 MHz, $CDCl_3$) δ 12.52 (s, 1H), 8.15 – 8.14 (m, 1H), 7.73 (dd, *J* = 7.7, 1.0 Hz, 1H), 7.63 (s, 2H), 7.04 (dd, *J* = 7.7, 4.9 Hz, 1H), 6.98 (s, 1H), 2.64 (d, *J* = 6.1 Hz, 4H), 2.18 (s, 6H), 1.87 (dd, *J* = 27.6, 5.4 Hz, 4H). **^{13}C NMR** (151 MHz, $CDCl_3$) δ 167.0, 147.1, 140.0, 138.3, 137.9, 133.3, 130.8, 126.5, 125.4, 119.8, 116.1, 107.2, 22.9, 22.4, 21.1, 21.1, 20.5,. **HRMS** (ESI): m/z $[M+H]^+$ calcd for $[C_{20}H_{22}N_3O]^+$ required 320.1758, found 320.1761.

3,5-dimethoxy-N-(5,6,7,8-tetrahydro-9H-pyrido[2,3-b]indol-9-yl)benzamide (1n)

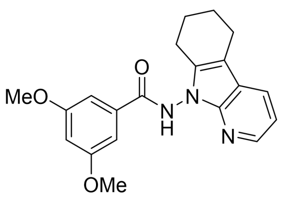

Yield: 315.9 mg (36%). White solid, mp: 227-228 ˚C. **^{1}H NMR** ^{1}H NMR (600 MHz, $CDCl_3$) δ 13.17 (s, 1H), 8.17 (d, *J* = 4.4 Hz, 1H), 7.73 (d, *J* = 7.4 Hz, 1H), 7.19 (d, *J* = 2.0 Hz, 2H), 7.05 (dd, *J* = 7.7, 4.9 Hz, 1H), 6.44 (s, 1H), 3.58 (s, 6H), 2.63 (d, *J* = 5.9 Hz, 4H), 1.86 (dd, *J* = 31.2, 3.8 Hz, 4H). **^{13}C NMR** (151 MHz, $CDCl_3$) δ 166.2, 160.5, 147.2, 139.6, 138.6, 132.6, 126.8, 120.1, 116.2, 107.4, 105.9, 104.9, 55.3, 22.8, 22.4, 21.0, 20.4,. **HRMS** (ESI): m/z $[M+H]^+$ calcd for $[C_{20}H_{22}N_3O_3]^+$ required 352.1656, found 352.1659.

3,4,5-trimethoxy-N-(5,6,7,8-tetrahydro-9H-pyrido[2,3-b]indol-9-yl)benzamide (1o)

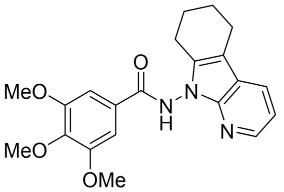

Yield: 297.2 mg (26%). White solid, mp: 257-258 ˚C. **^{1}H NMR** ^{1}H NMR (600 MHz, $CDCl_3$) δ 13.29 (s, 1H), 8.17 (d, *J* = 4.4 Hz, 1H), 7.75 (d, *J* = 7.6 Hz, 1H), 7.37 (s, 2H), 7.06 (dd, *J* = 7.7, 4.9 Hz, 1H), 3.81 (s, 3H), 3.61 (s, 6H), 2.63 (s, 4H), 1.86 (d, *J* = 29.1 Hz, 4H). **^{13}C NMR** (151 MHz, $CDCl_3$) δ 166.1, 152.7, 147.3, 141.0, 139.4, 138.7, 126.9, 125.8, 120.2, 116.2, 107.4, 104.9, 60.7, 56.0, 22.8, 22.4, 21.0, 20.4. **HRMS** (ESI): m/z $[M+H]^+$ calcd for $[C_{21}H_{24}N_3O_4]^+$ required 382.1762, found 382.1765.

N-(5,6,7,8-tetrahydro-9H-pyrido[2,3-b]indol-9-yl)thiophene-2-carboxamide (1p)

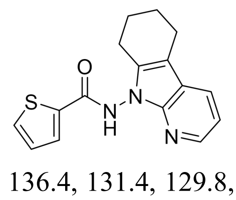

Yield: 249.9 mg (28%). White solid, mp: 216-217 ˚C. **^{1}H NMR** ^{1}H NMR (600 MHz, $CDCl_3$) δ 13.27 (s, 1H), 8.11 (d, *J* = 4.6 Hz, 1H), 7.99 (d, *J* = 3.3 Hz, 1H), 7.74 (d, *J* = 7.7 Hz, 1H), 7.37 (d, *J* = 4.9 Hz, 1H), 7.04 (dd, *J* = 7.7, 5.0 Hz, 1H), 6.91 (t, *J* = 4.3 Hz, 1H), 2.66 (d, *J* = 42.0 Hz, 4H), 1.88 (dd, *J* = 33.7, 4.9 Hz, 4H). **^{13}C NMR** (151 MHz, $CDCl_3$) δ 162.2, 146.7, 139.6, 138.6, 136.4, 131.4, 129.8, 128.0, 127.0, 120.1, 116.2, 107.4, 22.8, 22.4, 21.1, 20.5. **HRMS** (ESI): m/z $[M+H]^+$ calcd for $[C_{16}H_{16}N_3OS]^+$ required 298.1009, found 298.1014.

(*E*)-2-methyl-N-(5,6,7,8-tetrahydro-9H-pyrido[2,3-b]indol-9-yl)but-2-enamide (1q)

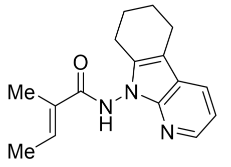

Yield: 153.5 mg (19%). White solid, mp: 153-154 ˚C. **^{1}H NMR** ^{1}H NMR (600 MHz, $CDCl_3$) δ 12.0 (s, 1H), 8.05 (t, *J* = 11.2 Hz, 1H), 7.65 (dd, *J* = 7.7, 1.1 Hz, 1H), 6.98 – 6.96 (m, 2H), 2.59 (d, *J* = 4.9 Hz, 4H), 1.96 (s, 3H), 1.84 (dd, *J* = 33.4, 5.5 Hz, 4H), 1.75 (d, *J* = 6.8 Hz, 3H). **^{13}C NMR** (151 MHz, $CDCl_3$) δ 168.7, 146.9, 140.0, 138.0, 134.1, 129.7, 126.3, 119.6, 115.9, 107.0, 22.8, 22.4, 21.0, 20.5. **HRMS** (ESI): m/z $[M+H]^+$ calcd for $[C_{16}H_{20}N_3O]^+$ required 270.1601, found 270.1608.

N-(5,6,7,8-tetrahydro-9H-pyrido[2,3-b]indol-9-yl)cyclohex-1-ene-1-carboxamide (1r)

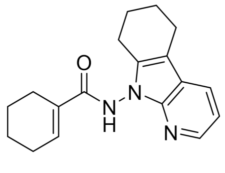

Yield: 230.1 mg (26%). White solid, mp: 173-174 ˚C. **^{1}H NMR** ^{1}H NMR (600 MHz, $CDCl_3$) δ 11.36 (s, 1H), 8.06 (d, *J* = 3.9 Hz, 1H), 7.68 (dd, *J* = 7.7, 1.2 Hz, 1H), 7.10 (s, 1H), 6.98 (dd, *J* = 7.7, 4.9 Hz, 1H), 2.61 (t, *J* = 5.7 Hz, 4H), 2.45 (s, 2H), 2.18 – 2.17 (m, 2H), 1.86 (dd, *J* = 31.3, 5.4 Hz, 4H), 1.68 – 1.59 (m, 4H). **^{13}C NMR** (151 MHz, $CDCl_3$) δ 168.1, 146.9, 140.3, 138.0, 136.9, 131.1, 126.3, 119.6, 116.0, 107.1, 25.7, 24.3, 22.9, 22.5, 21.4, 21.0, 20.5. **HRMS** (ESI): m/z $[M+H]^+$ calcd for $[C_{18}H_{22}N_3O]^+$ required 296.1758, found 296.1760.

N-(6,7,8,9-tetrahydrocyclohepta[4,5]pyrrolo[2,3-b]pyridin-10(5H)-yl)benzamide (1s)

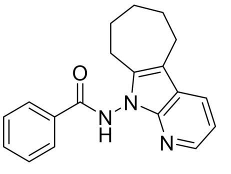

Yield: 420.9 mg (46%). White solid, mp: 196-197 ˚C. **^{1}H NMR** ^{1}H NMR (600 MHz, $CDCl_3$) δ 13.42 (s, 1H), 8.14 – 8.11 (m, 1H), 8.00 – 7.93 (m, 2H), 7.76 (d, *J* = 7.8 Hz, 1H), 7.36 (td, *J* = 7.4, 0.9 Hz, 1H), 7.18 (dd, *J* = 11.0, 4.5 Hz, 2H), 7.06 (ddd, *J* = 11.3, 5.1, 2.9 Hz, 1H), 5.29 (dd, *J* = 7.3, 4.1 Hz, 1H), 2.82 – 2.78 (m, 4H), 1.87 – 1.70 (m, 5H). **^{13}C NMR** (151 MHz, $CDCl_3$) δ 163.8, 145.9, 141.3, 149.7, 139.7, 131.9, 130.9, 128.4, 128.3, 127.8, 126.6, 126.5, 120.4, 116.2,

111.0, 31.5, 28.2, 26.8, 25.5, 24.5. **HRMS** (ESI): m/z $[M+H]^+$ calcd for $[C_{19}H_{20}N_3O]^+$ required 306.1601, found 306.1609.

N-(2-ethyl-3-methyl-1H-pyrrolo[2,3-b]pyridin-1-yl)benzamide (1t)

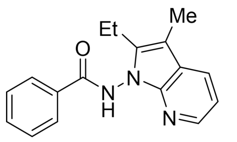

Yield: 326.6 mg (39%). White solid, mp: 217-218 ˚C. **^{1}H NMR** ^{1}H NMR (600 MHz, $CDCl_3$) δ 13.11 (s, 1H), 8.232– 8.21 (m, 2H), 7.96 – 7.94 (m, 1H), 7.79 (dd, *J* = 7.7, 1.3 Hz, 1H), 7.36 (t, *J* = 7.4 Hz, 1H), 7.19 (t, *J* = 7.7 Hz, 2H), 7.08 (dd, *J* = 7.7, 4.9 Hz, 1H), 2.71 (s, 2H), 2.22 (s, 3H), 1.21 (t, *J* = 7.6 Hz, 3H). **^{13}C NMR** (151 MHz, $CDCl_3$) δ 166.8, 147.0, 140.8, 140.4, 131.9, 130.9, 128.3, 127.8, 127.0, 121.2, 116.2, 104.0, 17.4, 14.0, 8.4. **HRMS** (ESI): m/z $[M+H]^+$ calcd for $[C_{18}H_{17}N_2O]^+$ required 280.1445, found 280.1453.

N-(2-methyl-1H-pyrrolo[2,3-b]pyridin-1-yl)benzamide (1u)

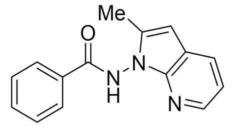

Yield: 271.0 mg (36%). White solid, mp: 169-170 ˚C. **^{1}H NMR** ^{1}H NMR (600 MHz, $CDCl_3$) δ 13.23 (s, 1H), 8.18 (dd, *J* = 4.9, 1.2 Hz, 1H), 7.97 – 7.95 (m, 2H), 7.79 (dd, *J* = 7.8, 1.3 Hz, 1H), 7.37 (t, *J* = 7.4 Hz, 1H), 7.21 (t, *J* = 7.8 Hz, 2H), 7.07 (dd, *J* = 7.8, 4.9 Hz, 1H), 6.23 (d, *J* = 1.0 Hz, 1H), 2.37 (s, 3H). **^{13}C NMR** (151 MHz, $CDCl_3$) δ 166.7, 147.0, 140.1, 139.7, 132.0, 130.8, 128.7, 128.4, 127.7, 120.3, 116.8, 97.2, 11.9. **HRMS** (ESI): m/z $[M+H]^+$ calcd for $[C_{15}H_{14}N_3O]^+$ required 252.1132, found 252.1140.

3.2 The procedure for the synthesis of 1v and 1w

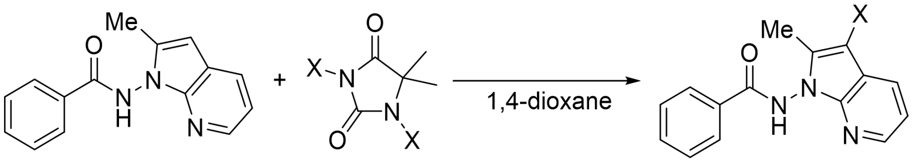

1u

1v, X = Br
1w, X = Cl

A flask charged with magnetic stirrer added **1u** (1 mmol), 1,3-dibromo-5,5-dimethylhydantoin or 1,3-dichloro-5,5-dimethylhydantoin (0.55 equiv) with subsequent addition of 1,4-dioxane (10 mL) as solvent. After the reaction was completed, the mixture was quenched with saturated $NaHSO_3$ solution and extracted with DCM for three times. These extracts were combined and dried over Na_2SO_4. The residue was purified by flash column chromatography on silica gel to give **1v** and **1w**.[2]

N-(3-bromo-2-methyl-1H-pyrrolo[2,3-b]pyridin-1-yl)benzamide (1v)

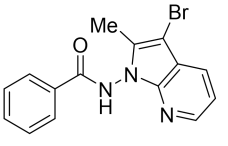

1v was obtained at room temperature for 15 min. Yield: 263.2 mg (80%). White solid, mp: 209-210 ˚C. **^{1}H NMR** ^{1}H NMR (600 MHz, $CDCl_3$) δ 12.95 (s, 1H), 8.21 (dd, *J* = 4.8, 1.0 Hz, 1H), 7.89 (d, *J* = 7.3 Hz, 2H), 7.79 (dd, *J* = 7.8, 1.1 Hz, 1H), 7.39 (t, *J* = 7.4 Hz, 1H), 7.21 (t, *J* = 7.9 Hz, 2H), 7.16 (dd, *J* = 7.8, 4.9 Hz, 1H), 2.37 (s, 3H). **^{13}C NMR** (151 MHz, $CDCl_3$) δ 166.5, 145.9, 141.5, 137.5, 132.3, 130.3, 128.5, 127.9, 127.6, 120.0, 117.5, 87.5, 10.5. **HRMS** (ESI): m/z $[M+H]^+$ calcd for $[C_{15}H_{13}BrN_3O]^+$ required 330.0237, found 330.0243.

N-(3-chloro-2-methyl-1H-pyrrolo[2,3-b]pyridin-1-yl)benzamide (1w)

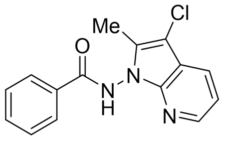

1w was obtained at 80 ºC for 3 h. Yield: 157.6 mg (55%). White solid, mp: 184-185 ˚C. **^{1}H NMR** ^{1}H NMR (600 MHz, $CDCl_3$) δ 12.42 (s, 1H), 8.22 (d, *J* = 4.3 Hz, 1H), 7.90 – 7.86 (m, 3H), 7.42 (t, *J* = 7.4 Hz, 1H), 7.24 (t, *J* = 7.9 Hz, 2H), 7.16 (dd, *J* = 7.8, 4.9 Hz, 1H), 2.37 (s, 3H). **^{13}C**

NMR (151 MHz, $CDCl_3$) δ 166.6, 145.3, 141.6, 135.6, 132.4, 130.4, 128.5, 127.7, 127.0, 118.5, 117.4, 101.2, 9.4. **HRMS** (ESI): m/z $[M+H]^+$ calcd for $[C_{15}H_{13}ClN_3O]^+$ required 286.0742, found 286.0745.

3.3 The procedure for the synthesis of 1x

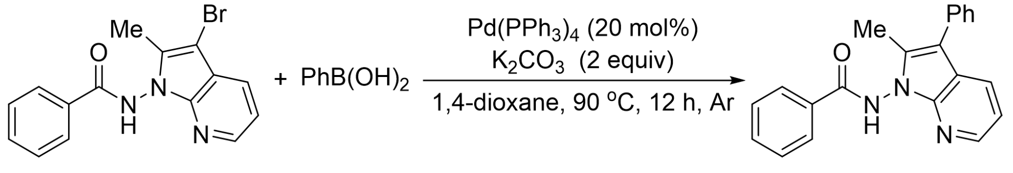

1v **1x**

A solution of **1v** (1 mmol), phenylboronic acid (1.5 equiv), $Pd(PPh_3)_4$ (20 mol%), K_2CO_3 (2 equiv) in 1,4-dioxane was stirred at 90 °C in Ar for 12 h. The mixture was quenched with saturated $NaHCO_3$ solution and extracted with DCM for three times. These extracts were combined and dried over Na_2SO_4. The residue was purified by flash column chromatography on silica gel to give **1x**.

N-(2-methyl-3-phenyl-1H-pyrrolo[2,3-b]pyridin-1-yl)benzamide (1x)

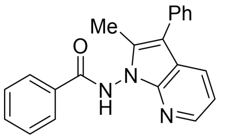

Yield: 281.2 mg (85%). White solid, mp: 213-214 ˚C. **^{1}H NMR** ^{1}H NMR (600 MHz, $CDCl_3$) δ 13.06 (s, 1H), 8.24 (dd, J = 4.8, 0.9 Hz, 1H), 7.98 – 7.95 (m, 3H), 7.44 (dd, J = 6.5, 4.9 Hz, 4H), 7.40 (t, J = 7.5 Hz, 1H), 7.34 – 7.30 (m, 1H), 7.24 (t, J = 7.4 Hz, 2H), 7.12 (dd, J = 7.8, 4.9 Hz, 1H), 2.45 (s, 3H). **^{13}C NMR** (151 MHz, $CDCl_3$) δ 166.8, 146.5, 140.8, 136.0, 133.9, 132.2, 130.8, 129.5, 128.7, 128.5, 128.0, 127.8, 127.7, 126.5, 119.7, 117.2, 111.6, 10.3. **HRMS** (ESI): m/z $[M+H]^+$ calcd for $[C_{21}H_{18}N_3O]^+$ required 328.1444, found 328.1446.

3.4 The procedure for the synthesis of 2n and 2o

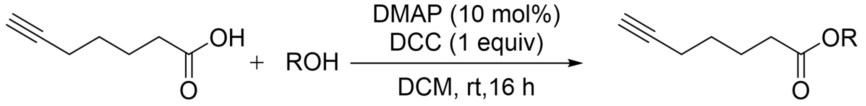

A solution of 6-heptynoic acid (1 mmol), estrone or dehydroepiandrosterone (1.1 equiv), DMAP (10 mol%), DCC (1 equiv) in DCM was stirred at room temperature for 12 h. The mixture was quenched with saturated $NaHCO_3$ solution and extracted with DCM for three times. These extracts were combined and dried over Na_2SO_4. The residue was purified by flash column chromatography on silica gel to give **2n** and **2o**.

(8R,9S,13S,14S)-13-methyl-17-oxo-7,8,9,11,12,13,14,15,16,17-decahydro-6H-cyclopenta[a]phenanthren-3-yl hept-6-ynoate (2n)

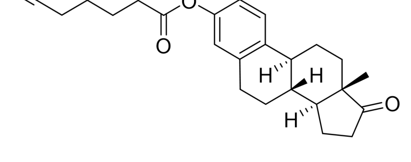

Yield: 347.8 mg (92%). White solid, mp: 107-108 ˚C. **^{1}H NMR** ^{1}H NMR (600 MHz, $CDCl_3$) δ 7.27 (d, J = 8.5 Hz, 1H), 6.84 (d, J = 8.5 Hz, 1H), 6.80 (s, 1H), 2.90 (d, J = 6.3 Hz, 2H), 2.57 (t, J = 7.4 Hz, 2H), 2.49 (dd, J = 19.1, 8.8 Hz, 1H), 2.41 – 2.37 (m, 1H), 2.29 – 2.24 (m, 3H), 2.16 – 2.10 (m, 1H), 2.07 – 1.94 (m, 4H), 1.89 – 1.84 (m, 2H), 1.67 – 1.40 (m, 8H), 0.90 (s, 3H). **^{13}C NMR** (151 MHz, $CDCl_3$) δ 172.1, 148.6, 138.0, 137.3, 126.4, 121.6, 118.7, 83.9, 68.8, 50.4, 47.9, 44.2, 38.0, 35.8, 31.6, 29.4, 27.8, 26.3, 25.8, 24.0, 21.6, 18.2, 13.8. **HRMS** (ESI): m/z $[M+Na]^+$ calcd for $[C_{25}H_{30}NaO_3]^+$ required 401.2087, found 401.2090.

(3S,8R,9S,10R,13S,14S)-10,13-dimethyl-17-oxo-2,3,4,7,8,9,10,11,12,13,14,15,16,17-tetradecahydro-1H-cyclopenta[a]phenanthren-3-yl hept-6-ynoate (2o)

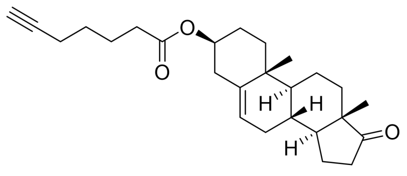

Yield: 356.6 mg (90%). White solid, mp: 115-116 ˚C. **^{1}H NMR** ^{1}H NMR (600 MHz, $CDCl_3$) δ 5.38 (s, 1H), 4.60 – 4.56 (m, 1H), 2.43 (dd, *J* = 19.1, 8.8 Hz, 1H), 2.32 – 2.27 (m, 4H), 2.21 – 2.17 (m, 2H), 2.10 – 2.02 (m, 2H), 1.94 – 1.90 (m, 2H), 1.86 – 1.81 (m, 3H), 1.76 – 1.42 (m, 10H), 1.29 – 1.24 (m, 2H), 1.15 – 1.10 (m, 1H), 1.04 – 1.02 (m, 4H), 0.88 – 0.85 (m, 3H). **^{13}C NMR** (151 MHz, $CDCl_3$) δ 172.7, 139.9, 121.8, 84.0, 73.6, 68.6, 51.7, 50.1, 47.5, 38.1, 36.9, 36.7, 35.8, 34.1, 31.5, 31.4, 30.8, 27.8, 27.7, 24.1, 21.9, 20.3, 19.3, 18.1, 13.5. **HRMS** (ESI): m/z $[M+Na]^+$ calcd for $[C_{26}H_{36}NaO_3]^+$ required 419.2557, found 419.2563.

4. General procedure for synthesis of products 3

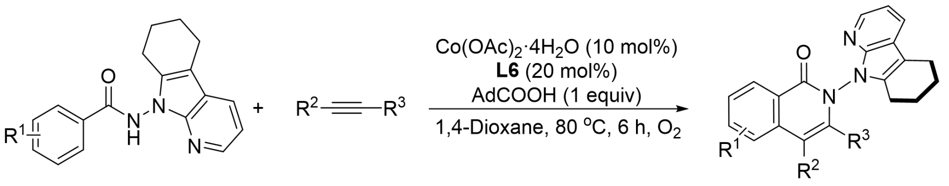

An oven dried schleck tube charged with magnetic stirrer added benzamide/vinylamide **1** (0.2 mmol), $Co(OAc)_2{\cdot}4H_2O$ (0.02 mmol, 10 mol%), **L6** (0.04 mmol, 20 mol%), 1-adamantanecarboxylic acid (0.2 mmol, 1.0 equiv) with subsequent addition of 1,4-dioxane (2 mL) as solvent. To this reaction mixture, **2** (0.24 mmol, 1.2 eq) was added under O_2. Then, the reaction system was stirred at 80 °C for 6 h. After the reaction was completed, the reaction mixture was quenched with $NaHCO_3$ saturated solution and extracted with CH_2Cl_2. The combined organic layer extracts were washed with brine, dried over Na_2SO_4, and concentrated under reduced pressure, and purified on silica gel chromatography (petroleum ether/ethyl acetate = 5:1) to afford the corresponding products.

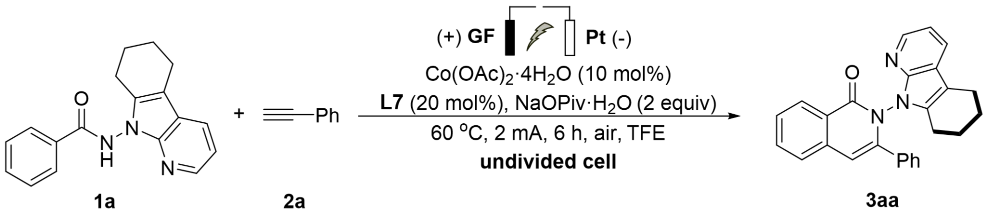

The electrolysis was carried out in an undivided cell with a graphite felt anode (10 mm × 15 mm × 6 mm) and a platinum cathode (10 mm × 10 mm × 0.1 mm). Corresponding substrate **1** (0.2 mmol), **2** (0.24 mmol, 1.2 equiv) $Co(OAc)_2{\cdot}4H_2O$ (0.02 mmol, 10 mol %), **L7** (0.04 mmol, 20 mol%), and $NaOPiv{\cdot}H_2O$ (0.4 mmol, 2.0 equiv) were dissolved in TFE (5 mL) and stirred for 6 hours at 60 °C. Then, the mixture was transferred to a flask and the electrodes were rinsed with DCM. The combined solvent was washed with $NaHCO_3$ saturated solution. The combined organic layer extracts were washed with brine, dried over Na_2SO_4, and concentrated under reduced pressure, and purified on silica gel chromatography (petroleum ether/ethyl acetate = 5:1) to afford the corresponding products

5. Mechanistic studies

5.1 H/D exchange experiments

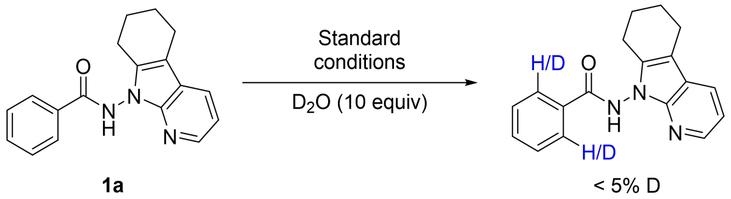

An oven dried schlenk tube charged with magnetic stirrer added benzamide **1a** (0.1 mmol), $Co(OAc)_2 \cdot 4H_2O$ (0.01 mmol, 10 mol%), **L6** (0.02 mmol, 20 mol%), D_2O (10 equiv) with subsequent addition of 1,4-dioxane (1 mL) as solvent. Then, the reaction system was stirred at 80 °C under O_2 for 6 h. After the reaction was completed, the reaction mixture was diluted with CH_2Cl_2 and filtered through a pad of Celite. The reaction solution was detected by TLC, and then concentrated in vacuum. The product was purified by flash column chromatography using petroleum ether/ethyl acetate (2:1) as eluent. ^1H NMR analysis showed that the D contents in the recovered amide were 4%.

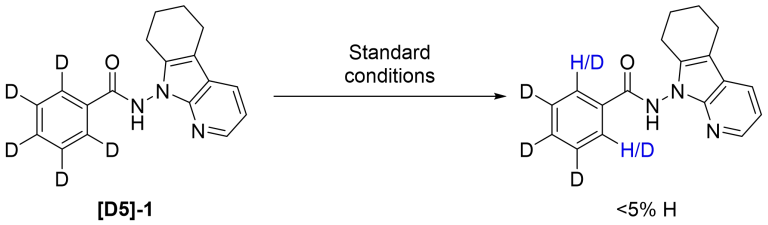

An oven dried schlenk tube charged with magnetic stirrer added Deuterated benzamide **[D$_5$]-1** (0.1 mmol), $Co(OAc)_2 \cdot 4H_2O$ (0.01 mmol, 10 mol%), **L6** (0.02 mmol, 20 mol%) with subsequent addition of 1,4-dioxane (1 mL) as solvent. Then, the reaction system was stirred at 80 °C under O_2 for 6 h. After the reaction was completed, the reaction mixture was diluted with CH_2Cl_2 and filtered through a pad of Celite. The reaction solution was detected by TLC, and then concentrated in vacuum. The product was purified by flash column chromatography using petroleum ether/ethyl acetate (2:1) as eluent. ^1H NMR analysis showed that the H contents in the recovered amide were 2%.

5.2 Parallel KIE experiments

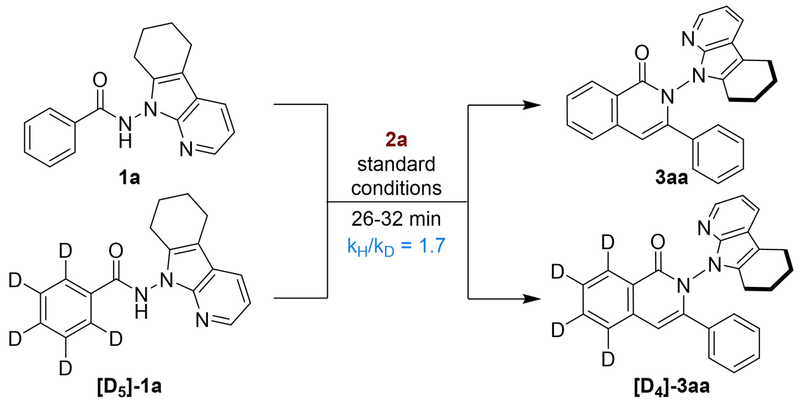

An oven dried schlenk tube charged with magnetic stirrer added Deuterated benzamide **[D5]-1** or benzamide **1a** (0.1 mmol), $Co(OAc)_2{\cdot}4H_2O$ (0.01 mmol, 10 mol%), **L6** (0.02 mmol, 20 mol%) with subsequent addition of 1,4-dioxane (1 mL) as solvent. To this reaction mixture, **2a** (0.24 mmol, 1.2 eq) was added under O_2. Then, the reaction system was stirred at 100 °C for 26 min, 28 min, 30 min, 32 min. After the reaction was completed, and immediately quenched with Ethyl acetate. and filtered through a pad of Celite. The reaction solution was removed under reduced pressure and ^{1}H NMR was taken using anisole (0.1 mmol, 10.8 mg) as the internal standard. The KIE was determined as k_H/k_D = 3.45/2.05 = 1.7.

5.3 Competitive KIE experiments

An oven dried schlenk tube charged with magnetic stirrer added Deuterated benzamide **[D5]-1** (0.05 mmol), benzamide **1a** (0.05 mmol), $Co(OAc)_2{\cdot}4H_2O$ (0.01 mmol, 10 mol%), **L6** (0.02 mmol, 20 mol%) with subsequent addition of 1,4-dioxane (1 mL) as solvent. To this reaction mixture, **2a** (0.24 mmol, 1.2 eq) was added under O_2. Then, the reaction system was stirred at 100 °C for 30 min. After the reaction was completed, the reaction mixture was diluted with CH_2Cl_2 and filtered through a pad of Celite. The reaction solution was detected by TLC, and then concentrated in vacuum. The product was purified by flash column chromatography using petroleum ether/ethyl acetate (5:1) as eluent. The KIE was determined as k_H/k_D = 0.65/0.35 = 1.9.

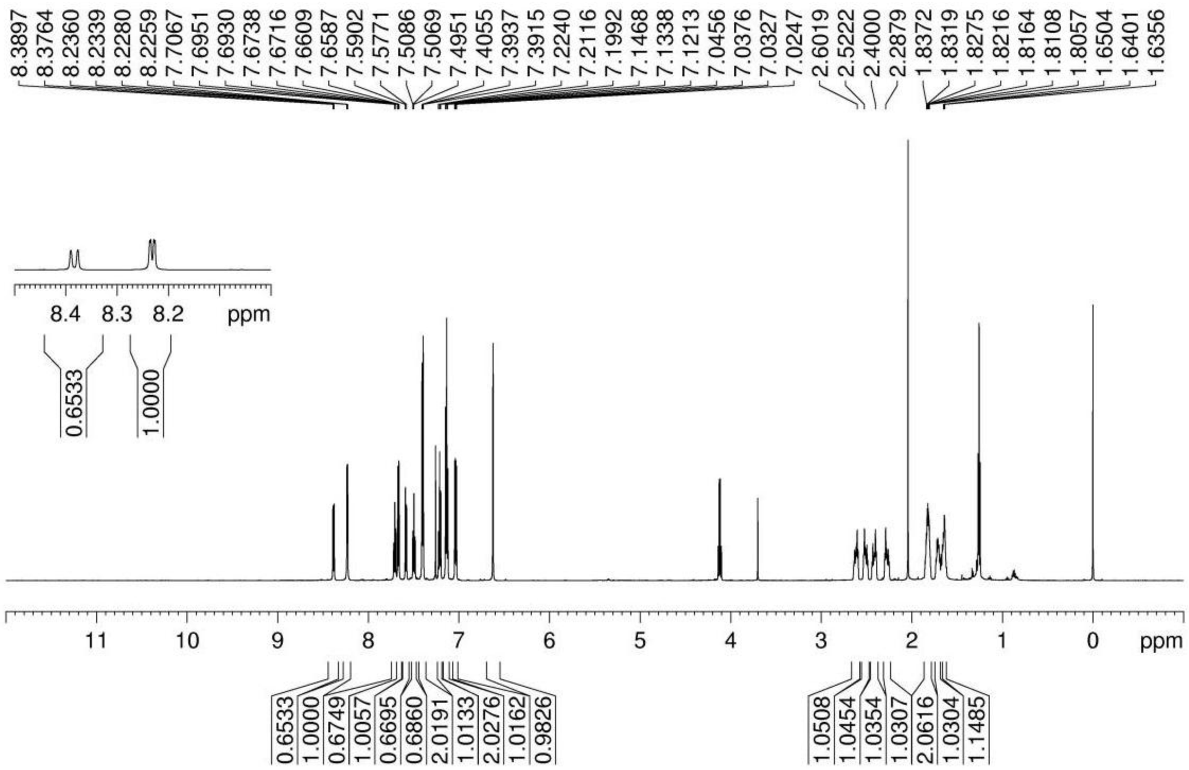
8.3897
8.3764
8.2360
8.2339
8.2280
8.2259
7.7067
7.6951
7.6930
7.6738
7.6716
7.6609
7.6587
7.5902
7.5771
7.5086
7.5069
7.4951
7.4055
7.3937
7.3915
7.2240
7.2116
7.1992
7.1468
7.1338
7.1213
7.0456
7.0376
7.0327
7.0247
2.6019
2.5222
2.4000
2.2879
1.8372
1.8319
1.8275
1.8216
1.8164
1.8108
1.8057
1.6504
1.6401
1.6356
8.4 8.3 8.2 ppm
0.6533
1.0000
11 10 9 8 7 6 5 4 3 2 1 0 ppm
0.6533
1.0000
0.6749
1.0057
0.6695
0.6860
2.0191
1.0133
2.0276
1.0162
0.9826
1.0508
1.0454
1.0354
1.0307
2.0616
1.0304
1.1485

6. Synthetic applications

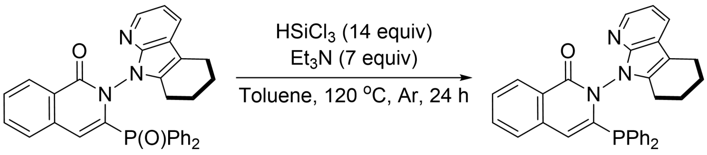

99% ee 83%, 99% ee

Et_3N (0.35 mmol, 14.0 equiv), $HSiCl_3$ (0.15 mmol, 6.0 equiv) were added sequentially to a solution of **3ai** (0.025 mmol, 1.0 equiv.) in dry toluene (5 mL) and the mixture was stirred under argon at 120 ºC for 12 h. The reaction mixture was quenched with aq. 1 N Na_2CO_3 solution and extracted with EA. The combined organic layer extracts were washed with brine, dried over Na_2SO_4, and concentrated under reduced pressure, and the residue was purified by silica gel chromatography (petroleum ether:ethyl acetate = 5:1) to give the **4**.[3]

(S)-3-(diphenylphosphaneyl)-2-(5,6,7,8-tetrahydro-9H-pyrido[2,3-b]indol-9-yl)isoquinolin-1(2H)-one (4)

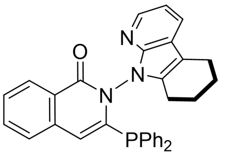

Yield: 10.4 mg (83%). White solid, mp: 216-217 ˚C. **^{1}H NMR** (600 MHz, $CDCl_3$) δ 8.32 (d, J = 7.9 Hz, 1H), 7.99 (dd, J = 4.7, 1.2 Hz, 1H), 7.69 (dd, J = 7.7, 1.3 Hz, 1H), 7.65 – 7.61 (m, 1H), 7.47 (dd, J = 11.6, 4.4 Hz, 1H), 7.43 – 7.38 (m, 5H), 7.30 (dt, J = 8.5, 6.8 Hz, 3H), 7.25 (dd, J = 10.6, 4.0 Hz, 2H), 6.97 (dd, J = 7.7, 4.8 Hz, 1H), 6.27 (s, 1H), 2.70 – 2.64 (m, 2H), 2.29– 2.25 (m, 1H), 1.87 – 1.80 (m, 2H), 1.74 – 1.72 (m, 2H), 1.59 – 1.54 (m, 1H). ^{13}C NMR (151 MHz, $CDCl_3$) δ 161.5, 147.6, 147.4 ($^2J_{C-P}$ = 9.2 Hz), 142.4, 137.4 ($^1J_{C-P}$ = 154.1 Hz), 134.9, 134.8, 134.2, 134.1, 133.3($^2J_{C-P}$ = 10.9 Hz), 130.0 ($^2J_{C-P}$ = 9.8 Hz), 132.9, 129.9, 129.3, 128.8, 128.7, 127.4, 126.5, 125.9, 125.6, 119.3, 116.6, 113.9, 108.5, 22.7, 22.3, 20.9 ($^2J_{C-P}$ = 4.4 Hz), 20.6. **^{31}P NMR** (243 MHz, $CDCl_3$) δ -13.8. **HRMS** (ESI): m/z $[M+H]^+$calcd for $[C_{32}H_{27}N_3OP]^+$ required 500.1886, found 500.1892. $[\alpha]_D^{25}$ = -69 (c = 0.1, CH_2Cl_2). The product was analyzed by HPLC to determine the enantiomeric excess: 99% ee (CHIRALPAK AS-H, hexane/i-PrOH =95/5, detector: 254 nm, T = 25 ºC, flow rate: 0.8 mL/min), t_1 (minor) = 10.565 min, t_2 (major) = 14.588 min.

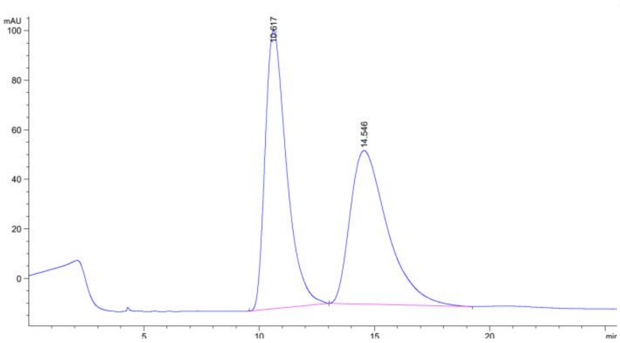

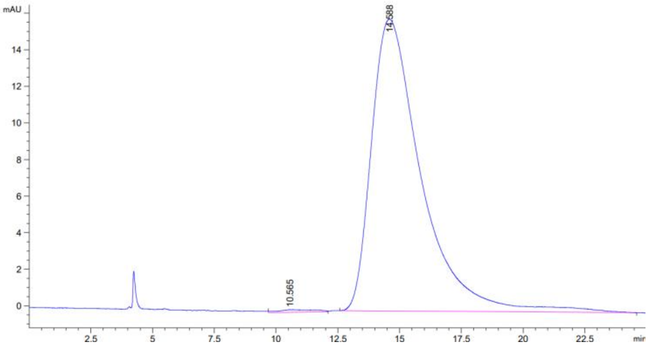

Peak	RetTime	Area	Height	Area
1	10.617	7245.56201	112.33806	50.9634
2	14.546	6971.61621	61.99229	49.0366

Peak	RetTime	Area	Height	Area
1	10.565	14.97165	1.51777e-1	0.6642
2	14.588	2239.10913	15.95400	99.3358

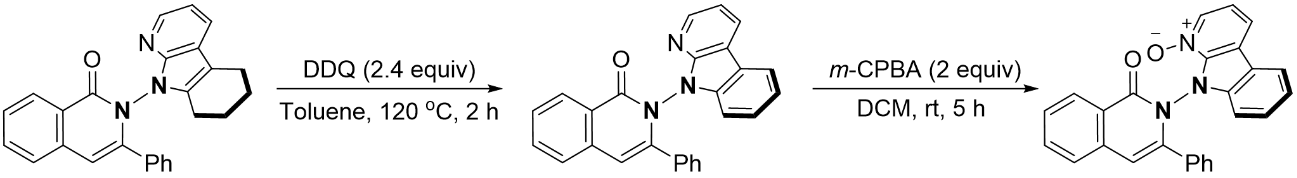

3aa, 96% ee **5**, 88%, 96% ee **6**, 42%, 96% ee

To a solution of **3aa** (0.1 mmol, 1.0 equiv) in toluene (1 mL) was added dropwise to a solution of DDQ (0.24 mmol, 2.4 equiv) in toluene (1 mL) at room temperature, and the mixture was stirred at 120 ºC for 2 h. After the reaction was completed, the hot solution was filtered, and concentrated under reduced pressure. The residue was purified by silica gel chromatography (petroleum ether:ethyl acetate = 5:1) to give the **5**.

To a solution of **7** (0.1 mmol, 1 equiv) in CH_2Cl_2 (1 mL) was added *m*-CPBA (0.2 mmol, 2.0 equiv) at 0 ºC. The mixture was stirred at room temperature for 5 h. After the reaction was completed, the saturated Na_2CO_3 aqueous solution was added slowly to quench the reaction. The mixture was extracted with CH_2Cl_2, dried over anhydrous Na_2SO_4, filtered, and concentrated under reduced pressure. The residue was purified by silica gel column chromatography (petroleum ether:ethyl acetate = 1:10) to afford product **6**.

(*R*)-3-phenyl-2-(9H-pyrido[2,3-b]indol-9-yl)isoquinolin-1(2H)-one (5)

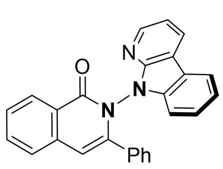

Yield: 34.6 mg (88%). White solid. mp: 197-198 ˚C. ^{1}H NMR (600 MHz, $CDCl_3$) δ 8.47 (dd, *J* = 4.9, 1.4 Hz, 1H), 8.39 (d, *J* = 8.0 Hz, 1H), 8.25 (dd, *J* = 7.7, 1.5 Hz, 1H), 7.97 (d, *J* = 7.8 Hz, 1H), 7.79 – 7.74 (m, 1H), 7.66 (d, *J* = 7.9 Hz, 1H), 7.54 – 7.51 (m, 1H), 7.50 – 7.49 (m, 2H), 7.44 – 7.41 (m, 1H), 7.28 (dd, *J* = 5.8, 2.0 Hz, 1H), 7.23 (dd, *J* = 7.7, 4.9 Hz, 1H), 7.19 (d, *J* = 8.1 Hz, 1H), 7.14 (t, *J* = 7.4 Hz, 1H), 7.07 (t, *J* = 7.6 Hz, 2H), 6.74 (s, 1H). ^{13}C NMR (151 MHz, $CDCl_3$) δ 161.0, 151.5, 146.6, 145.4, 139.7, 136.9, 133.7, 133.5, 129.0, 128.7, 128.6, 128.3, 127.7, 127.5, 127.1, 126.5, 125.5, 121.7, 121.2, 119.8, 117.2, 115.5, 109.1, 107.9. (ESI): m/z $[M+H]^+$calcd for $[C_{26}H_{18}N_3O]^+$ required 388.1444, found 388.1451. The product was analyzed by HPLC to determine the enantiomeric excess: 96% ee (CHIRALPAK AS-H, hexane/*i*-PrOH =85/15, detector: 254 nm, T = 25 ºC, flow rate: 1 mL/min), t_1 (minor = 8.581 min, t_2 (major) = 13.860 min.

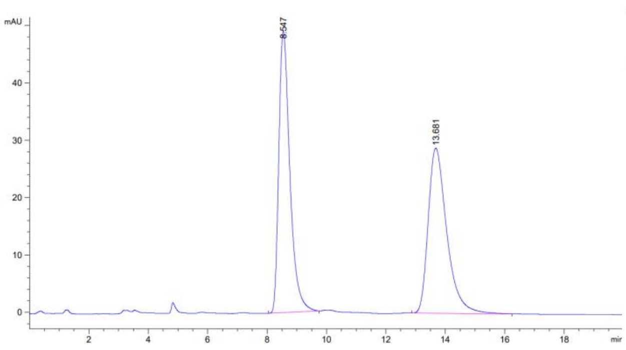

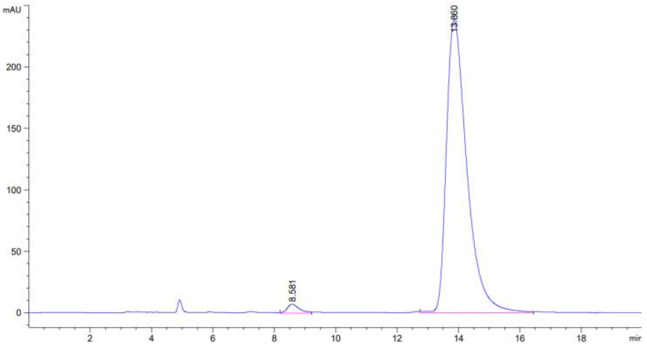

Peak	RetTime	Area	Height	Area
1	8.547	1266.48120	49.08044	50.3069
2	13.681	1251.02930	28.80413	49.6931

Peak	RetTime	Area	Height	Area
1	8.581	207.11267	7.34023	1.8454
2	13.860	1.10162e4	238.19600	98.1546

(*R*)-9-(1-oxo-3-phenylisoquinolin-2(1H)-yl)-9H-pyrido[2,3-b]indole 1-oxide (6)

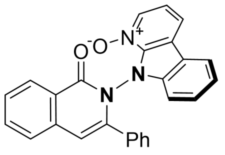

Yield: 17.0 mg (42%). White solid. mp: 266-267 ˚C. ^{1}H NMR (600 MHz, $CDCl_3$) δ 8.31 (d, *J* = 8.0 Hz, 1H), 8.18 (d, *J* = 6.4 Hz, 1H), 7.91 (d, *J* = 7.8 Hz, 1H), 7.85 (d, *J* = 7.8 Hz, 1H), 7.74 – 7.70 (m, 3H), 7.62 (d, *J* = 7.9 Hz, 1H), 7.47 (t, *J* = 7.5 Hz, 1H), 7.44 (t, *J* = 7.7 Hz, 1H), 7.30 (t, *J* = 7.5 Hz, 1H), 7.20 – 7.14 (m, 5H), 6.69 (s, 1H). ^{13}C NMR (151 MHz, $CDCl_3$) δ 161.0, 144.7, 141.7, 140.7, 137.3, 136.9, 134.3, 133.4, 129.6, 128.9, 128.8, 128.7,128.0, 127.1, 126.5, 124.3, 123.2, 121.7, 121.5, 120.0, 119.2, 118.3, 110.2, 107.6. (ESI): m/z $[M+H]^+$calcd for $[C_{26}H_{18}N_3O_2]^+$ required 404.1394, found 404.1399. The product was analyzed by HPLC to determine the enantiomeric excess: 96% ee

(CHIRALPAK AD-H, hexane/*i*-PrOH =70/30, detector: 254 nm, T = 25 °C, flow rate: 1 mL/min), t_1 (major) = 25.688 min, t_2 (minor) = 32.805 min.

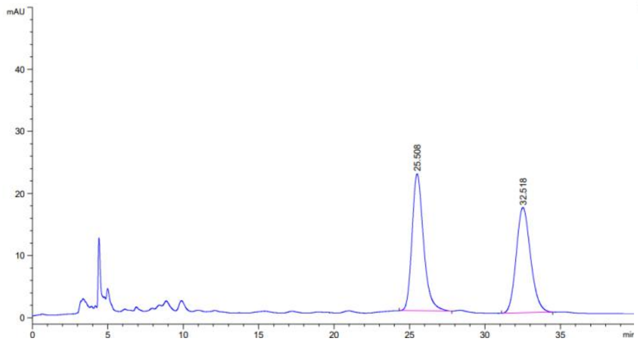

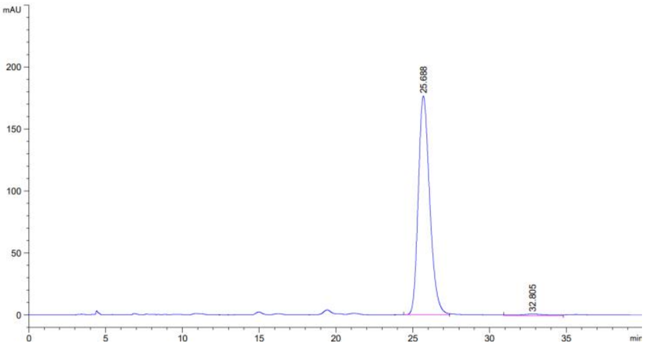

Peak	RetTime	Area	Height	Area
1	25.508	1172.60181	22.04536	51.0441
2	32.518	1124.62891	16.97393	48.959

Peak	RetTime	Area	Height	Area
1	25.688	9107.27832	176.42958	98.2243
2	32.805	164.63722	21.20850	1.7757

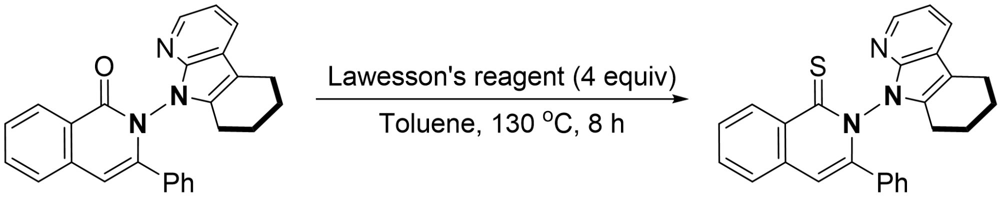

3aa 96% ee **7**, 75%, 96% ee

The Lawesson's reagent (0.4 mmol, 4.0 equiv) was added slowly to a solution of **3aa** (0.1 mmol, 1 equiv) in toluene (1 mL) at room temperature under Ar, and the mixture was stirred at 130 ºC for 8 h. After the reaction was completed, the reaction mixture was diluted with CH_2Cl_2 and filtered through a pad of Celite. The solvent was removed under reduced pressure and the residue was purified by silica gel column chromatography (petroleum ether:ethyl acetate = 5:1) to afford product **7**.

(*R*)-3-phenyl-2-(5,6,7,8-tetrahydro-9H-pyrido[2,3-b]indol-9-yl)isoquinoline-1(2H)-thione (7)

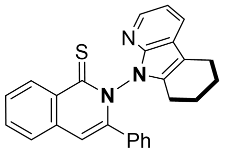

Yield: 30.6 mg (75%). Yellow solid. mp: 169-170 ˚C. ^{1}H NMR (600 MHz, $CDCl_3$) δ 8.98 (d, *J* = 8.3 Hz, 1H), 8.25 (dd, *J* = 4.8, 1.2 Hz, 1H), 7.74 – 7.71 (m, 1H), 7.68 (dd, *J* = 7.7, 1.2 Hz, 1H), 7.59 (t, *J* = 7.8 Hz, 1H), 7.56 – 7.53 (m, 1H), 7.38 – 7.37 (m, 2H), 7.21 (t, *J* = 7.5 Hz, 1H), 7.12 (t, *J* = 7.7 Hz, 2H), 7.04 (dd, *J* = 7.7, 4.8 Hz, 1H), 6.91 (s, 1H), 2.67 – 2.63 (m, 1H), 2.51 – 2.47 (m, 1H), 2.46 – 2.42 (m, 1H), 2.31 – 2.26 (m, 1H), 1.86 – 1.78 (m, 2H), 1.74 – 1.69 (m, 1H), 1.68 – 1.63 (m, 1H). ^{13}C NMR (151 MHz, $CDCl_3$) δ 187.1, 147.1, 146.6, 142.6, 135.4, 134.0, 133.7, 133.3, 132.2, 129.1, 128.6, 128.4, 127.7, 126.9, 126.1, 119.0, 116.6, 113.2, 108.8, 22.7, 22.2, 21.7, 20.4. (ESI): m/z $[M+H]^+$calcd for $[C_{26}H_{18}N_3O]^+$ required 408.1529, found 408.1541. The product was analyzed by HPLC to determine the enantiomeric excess: 96% ee (CHIRALPAK AS-H, hexane/*i*-PrOH =85/15, detector: 254 nm, T = 25 °C, flow rate: 1 mL/min), t_1 (minor) = 5.655 min, t_2 (major) = 6.911 min.

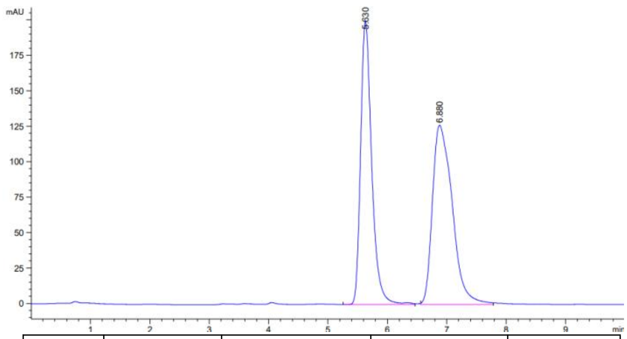

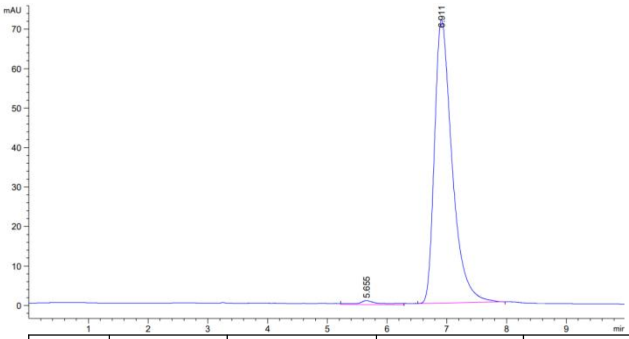

Peak	RetTime	Area	Height	Area
1	5.630	2598.27319	199.72209	46.9821
2	6.880	2932.07642	126.52087	53.0179

Peak	RetTime	Area	Height	Area
1	5.655	26.37694	9.99053e-1	1.7622
2	6.911	1470.47656	71.77865	98.2378

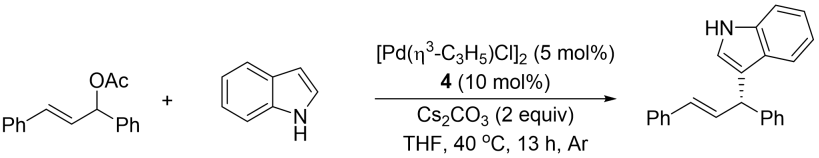

1.2 equiv

8, 92%, 73% ee

To a mixture of **4** (0.01 mmol, 10 mol%), $[Pd(C_3H_5)Cl]_2$ (0.005 mmol, 5 mol%), indole (0.1 mmol), 1,3-diphenyl-2-propenyl acetate (0.12 mmol, 1.2 equiv), and Cs_2CO_3 (0.2 mol, 2 equiv) was added THF (1 mL) at room temperature under an Ar atmosphere. After stirring for 13 h at 40 °C, the reaction mixture was quenched with water and extracted with CH_2Cl_2. The combined organic layer extracts were washed with brine, dried over Na_2SO_4, and concentrated under reduced pressure, and purified on silica gel chromatography (petroleum ether:ethyl acetate = 20:1) to afford **8**.[4]

(S,E)-3-(1,3-diphenylallyl)-1H-indole (8)

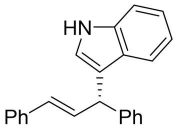

Yield: 28.5 mg (92%). **^{1}H NMR** (600 MHz, $CDCl_3$) δ 7.94 (s, 1H), 7.42 (d, *J* = 8.0 Hz, 1H), 7.37 – 7.34 (m, 5H), 7.31 – 7.26 (m, 4H), 7.24 – 7.15 (m, 3H), 7.04 – 6.99 (m, 1H), 6.88 (d, *J* = 1.8 Hz, 1H), 6.72 (dd, *J* = 15.8, 7.4 Hz, 1H), 6.44 (d, *J* = 15.8 Hz, 1H), 5.11 (d, *J* = 7.4 Hz, 1H). **^{13}C NMR** (151 MHz, $CDCl_3$) δ 143.4, 137.5, 136.7, 132.6, 130.6, 128.5, 128.5, 128.4, 127.2, 126.9, 126.4, 126.4, 122.6, 122.1, 119.9, 119.5, 118.8, 111.1, 46.2. The product was analyzed by HPLC to determine the enantiomeric excess: 73% ee (CHIRALPAK IB-H, hexane/*i*-PrOH =99/1, detector: 254 nm, T = 25 °C, flow rate: 0.7 mL/min), t_1 (minor) = 91.988 min, t_2 (major) = 101.020 min.

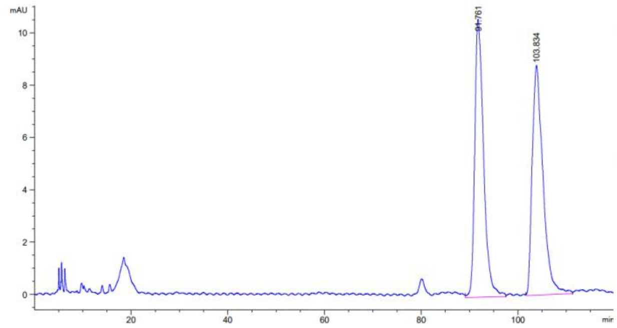

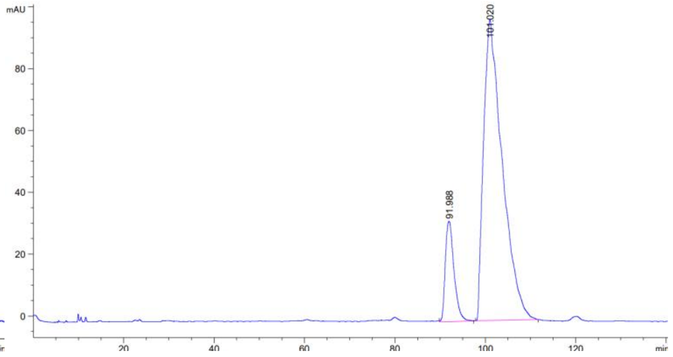

Peak	RetTime	Area	Height	Area

Peak	RetTime	Area	Height	Area

1	91.761	1348.84448	10.62305	49.7093
2	103.834	1364.61853	8.78623	50.2907

1	91.988	4365.51660	32.49841	13.4909
2	101.020	2.79934e4	97.3779	86.8091

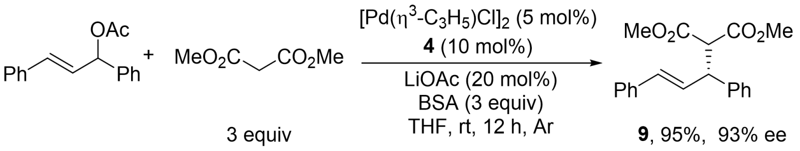

$[Pd(C_3H_5)Cl]_2$ (0.005 mmol, 5 mol%), LiOAc (0.02 mmol, 20 mol%) was added to a solution of **4** (0.01 mmol, 10mol%) in THF (0.4 mL) and the mixture was stirred under argon at room temperature for 30 min. Then 1,3-diphenyl-2-propenyl acetate (0.1 mmol, 1.0 equiv), dimethyl malonate (0.3 mmol, 3.0 equiv), *N*,*O*-bis(trimethylsilyl)-acetamide (BSA) (0.3 mmol, 3.0 equiv) were added subsequently, and the reaction mixture was stirred at room temperature for 12 h. The reaction mixture was quenched with NH_4Cl aqueous solution and extracted with CH_2Cl_2. The combined organic layer extracts were washed with brine, dried over Na_2SO_4, and concentrated under reduced pressure, and purified on silica gel chromatography (petroleum ether:ethyl acetate = 20:1) to afford **9**.[3]

Dimethyl (*S,E*)-2-(1,3-diphenylallyl)malonate (9)

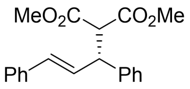

Yield: 30.7 mg (95%). Colorless oil.^{1}H NMR (600 MHz, $CDCl_3$) δ 7.34 – 7.24 (m, 8H), 7.24 – 7.18 (m, 2H), 6.48 (d, *J* = 15.7 Hz, 1H), 6.35 – 6.31 (m, 1H), 4.28 – 4.25 (m, 1H), 3.96 (d, *J* = 10.9 Hz, 1H), 3.70 (s, 3H), 3.51 (s, 3H). ^{13}C NMR (151 MHz, $CDCl_3$) δ 168.2, 167.8, 140.2, 136.9, 131.9, 129.2, 128.8, 128.5, 127.9, 127.6, 127.2, 126.4, 57.7, 52.6, 52.5, 49.2. The product was analyzed by HPLC to determine the enantiomeric excess: 93% ee (CHIRALPAK AD-H, hexane/*i*-PrOH =90/10, detector: 254 nm, T = 25 °C, flow rate: 1 mL/min), t_1 (major) = 11.74 min, t_2 (minor) = 16.34 min.

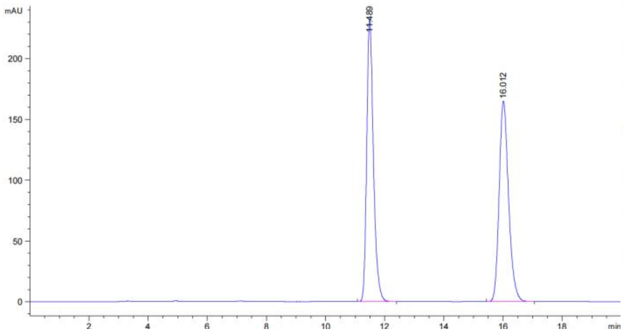

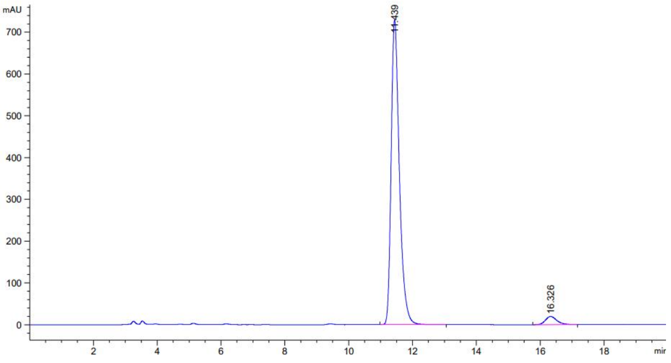

Peak	RetTime	Area	Height	Area
1	11.489	3651.54028	231.72360	50.0657
2	16.012	3641.95410	164.89719	49.9343

Peak	RetTime	Area	Height	Area
1	11.439	1.29972e4	729.60815	96.3903
2	16.326	486.73001	19.65545	3.6097

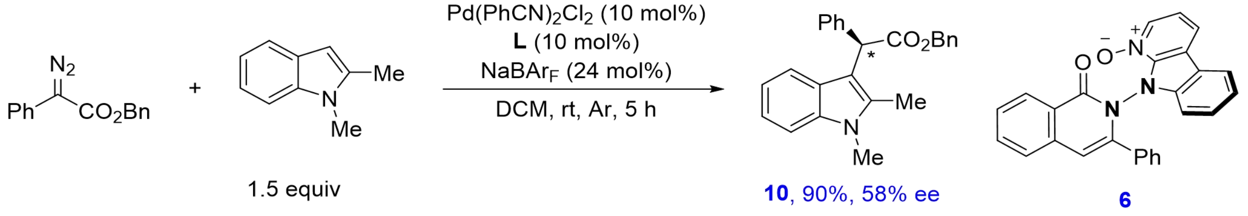

According to the reported literature[5], the $Pd(PhCN)_2Cl_2$ (3.8 mg, 0.01 mmol, 10 mol%), **8** (0.01 mmol, 10 mol%) and $NaBAr_F$ (21.2mg, 0.024 mmol, 24 mol%) were introduced into an 10 mL oven-dried Schlenk tube under Ar atmosphere. After dichloromethane (1.0 mL) was injected into the Schlenk tube, the solution was stirred at room temperature for 2 h. Indoles (21.7 mg, 0.15 mmol, 1.5 equiv) and α-aryl-α-diazoactates (25.2 mg, 0.1 mmol, 1.0 equiv) was then introduced in one portion under Ar atmosphere. The resulting mixture was stirred at room temperature for 5 h. The mixture was diluted with CH_2Cl_2 and filtered over a Celite. The reaction solution was concentrated in vacuum and purified by flash column chromatography to give the product (PE/EA = 30:1).

The product was analyzed by HPLC to determine the enantiomeric excess: 58% ee (CHIRALPAK AS-H, hexane/*i*-PrOH =90/10, detector: 254 nm, T = 25 °C, flow rate: 0.7 mL/min), t_1 (minor) = 11.254 min, t_2 (major) = 13.129 min.

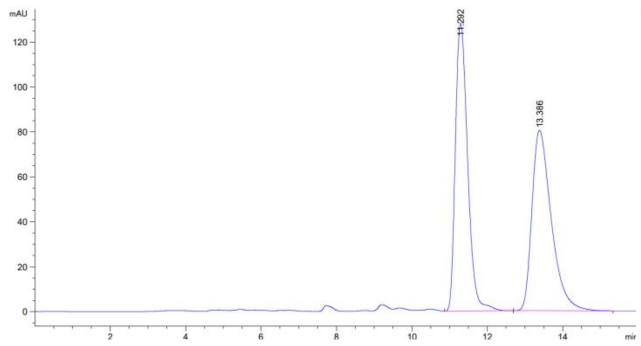

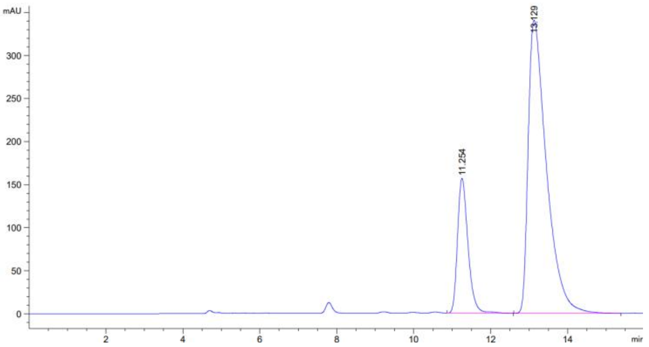

Peak	RetTime	Area	Height	Area
1	11.292	2895.48315	128.05927	50.2758
2	13.386	2863.71240	80.33157	49.7242

Peak	RetTime	Area	Height	Area
1	11.254	2967.18750	156.63911	20.8940
2	13.129	1.12339e4	340.25226	79.1060

7. Study on product stabilities

The enantiomerization barrier, corresponding to the barrier to rotation for the following atropisomers, was obtained by kinetic of racemization of an enantiomer. The slope of the firstorder kinetic line gives the racemization constant ($k_{racemization} = 2 \times k_{enantiomerization}$). According to the Eyring equation, the enantiomerization barrier ($\Delta G^{\neq}_{enantiomerization}$) can be obtained from enantiomerization constant ($k_{enantiomerization}$), R = 8.31451 J.K^{-1}.mol^{-1}, h = 6.62608 ×10^{-34} J·s and k_B = 1.38066 ×10^{-23} J·K^{-1}.[6,7,8]

$$\Delta G^{\neq}_{enantiomerization} = RT \cdot \ln \frac{Tk_B}{hk_{enantiomerization}}$$

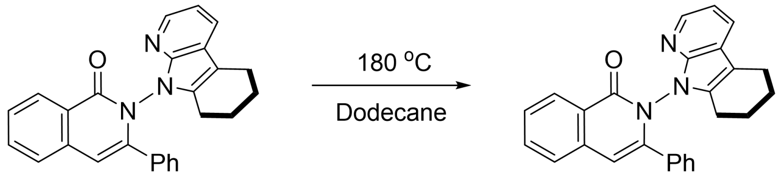

3aa, 98% ee   recovered **3aa**

A solution of (*R*)-**3aa** (10.0 mg, 98% ee) in dodecane (1 mL) was heated at the specific temperatures (Supplementary Table 11). The ee value was determined by chiral HPLC analysis at different intervals.

Supplementary Table 11. Thermal racemization of product **3aa** at 180 °C

Time (h)	0	1	4	6	8	10
ee (%)	97.520	97.440	97.292	97.150	97.012	96.992

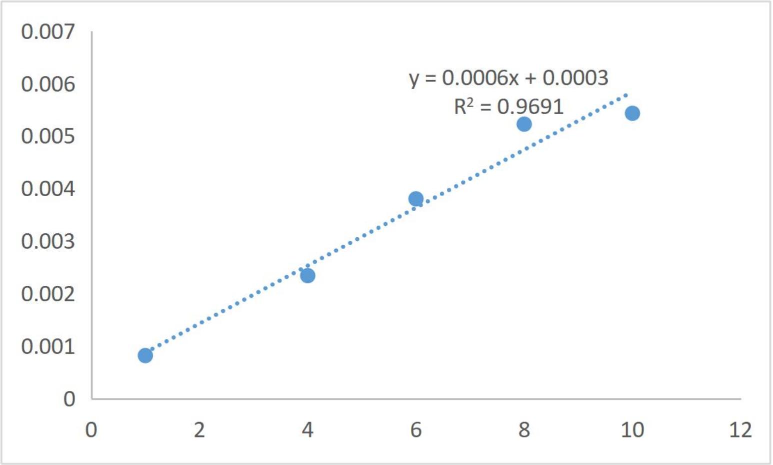

Supplementary Figure 1 The plot of ln(ee$_0$/ee$_t$) vs time of **3aa** at 180 °C

$k_{racemization}$ (180 °C) = 0.0006 h^{-1} = 1.7 × 10^{-7} s^{-1}; $k_{enantiomerization}$ (180 °C) = 8.3 × 10^{-8} s^{-1}; $\Delta G^{\neq}_{enantiomerization}$ = 174.0 kJ/mol = 41.6 kcal/mol; $t_{1/2}$ (25 °C) = 5.5 × 10^9 years.

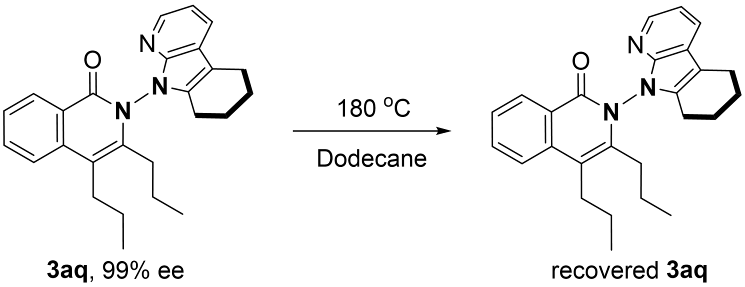

A solution of (*R*)-**3aq** (10.0 mg, 99% ee) in dodecane (1 mL) was heated at the specific temperatures (Supplementary Table 12). The ee value was determined by chiral HPLC analysis at different intervals.

Supplementary Table 12. Thermal racemization of product **3aq** at 180 °C

Time (h)	0	1	2	4	6
ee (%)	98.546	98.354	98.154	97.780	97.356

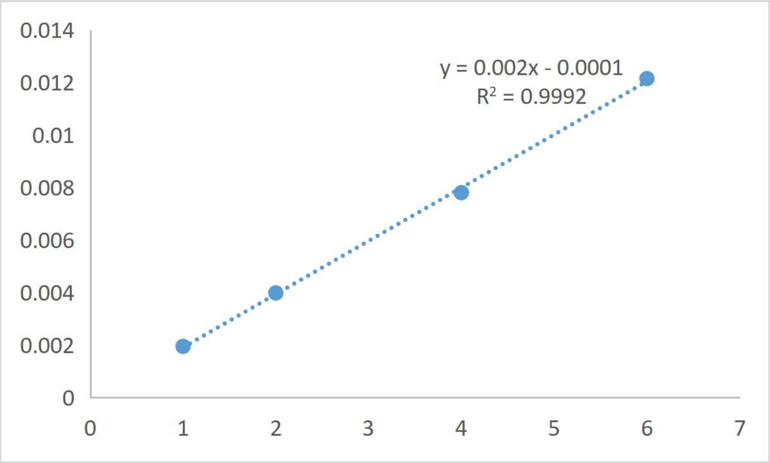

Supplementary Figure 2 The plot of ln(ee_0/ee_t) vs time of **3aq** at 180 °C

$k_{racemization}$ (180 °C) = 0.002 h^{-1} = 5.6 × 10^{-7} s^{-1}; $k_{enantiomerization}$ (180 °C) = 2.8 × 10^{-7} s^{-1}; $\Delta G^{\neq}_{enantiomerization}$ = 169.4 kJ/mol = 40.5 kcal/mol; $t_{1/2}$ (25 °C) = 9.2 × 10^8 years.

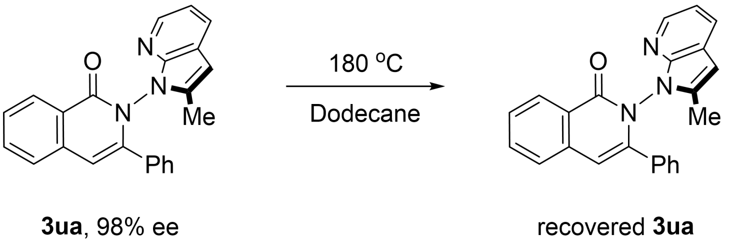

A solution of (*R*)-**3ua** (10.0 mg, 98% ee) in dodecane (1 mL) was heated at the specific temperatures (Supplementary Table 13). The ee value was determined by chiral HPLC analysis at different intervals.

Supplementary Table 13. Thermal racemization of product **3ua** at 180 °C

Time (h)	0	1	4	6	8	10
ee (%)	97.782	97.522	97.274	97.154	96.904	96.752

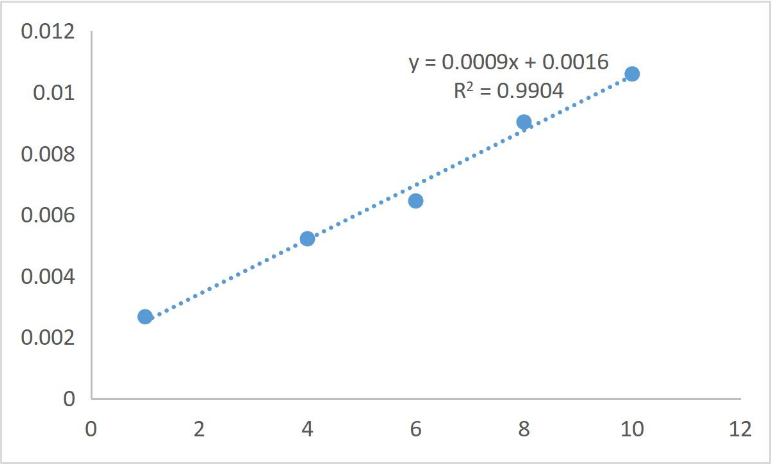

Supplementary Figure 3 The plot of ln(ee_0/ee_t) vs time of **3ua** at 180 ^oC

$k_{racemization}$ (180 ^oC) = 0.0009 h^{-1} = 2.5 × 10^{-7} s^{-1}; $k_{enantiomerization}$ (180 ^oC) = 1.3 × 10^{-7} s^{-1}; $\Delta G^{\neq}_{enantiomerization}$ = 172.5 kJ/mol = 41.2 kcal/mol; $t_{1/2}$ (25 ^oC) = 3.7 × 10^9 years.

8. The nonlinear effect studies

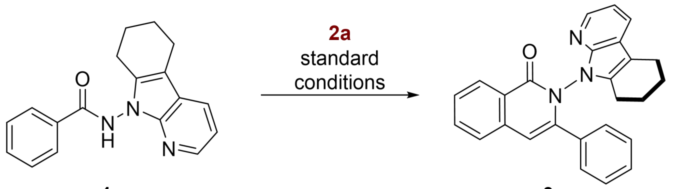

Supplementary Table 14. The nonlinear effect study between the ee of **L6** and the ee of **3aa**

ee of **L6**	0	20	40	60	80	100
ee of **3aa**	0	21	42	61	84	98

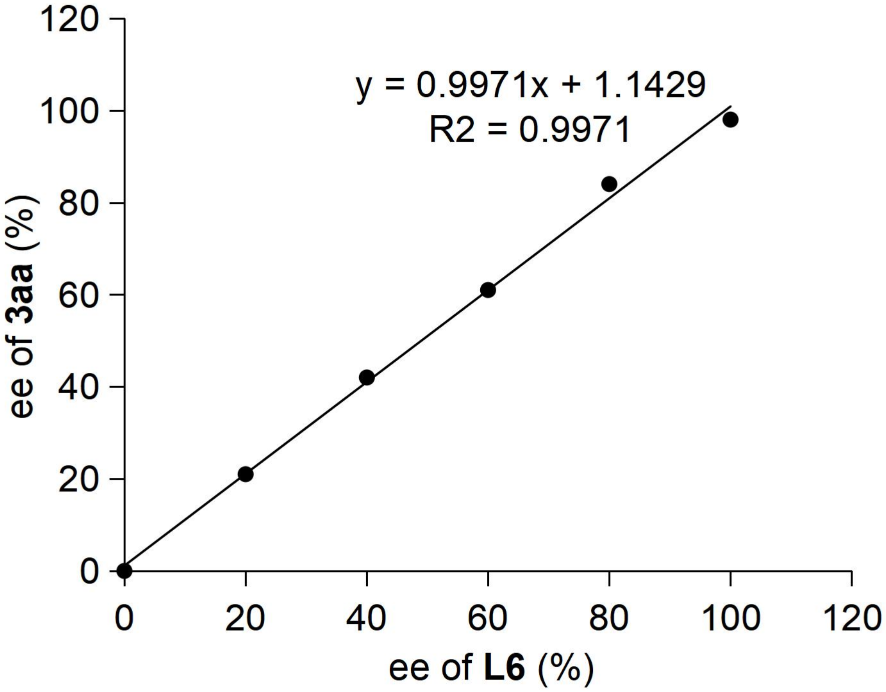

Supplementary Figure 4 The nonlinear effect study between the ee of **L6** and the ee of **3aa**

9. X-ray crystal structure of 3aa

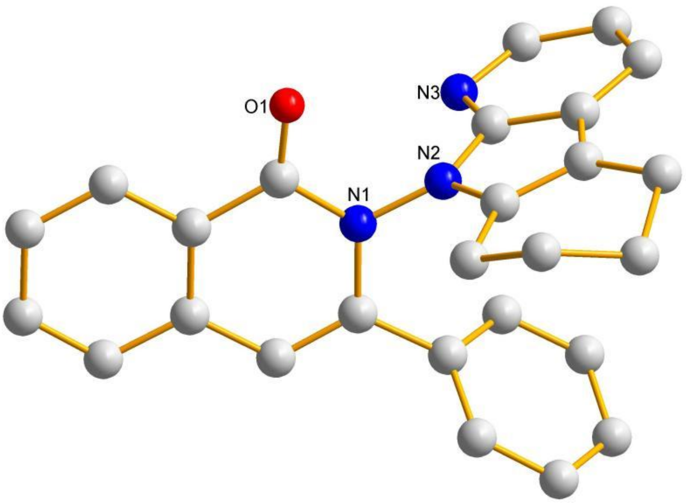

Supplementary Figure 5. X-ray molecular structure of **3aa** (CCDC 2257445).

Crystal data and structure refinement for 3aa

Identification code	**3aa**
Empirical formula	$C_{26}H_{21}N_3O$
Formula weight	36.51
Temperature/K	300.0
Crystal system	orthorhombic
Space group	$C222_1$
a/Å	11.6738(3)
b/Å	13.9355(3)
c/Å	25.6081(6)
α/°	90
β/°	90
γ/°	90
Volume/Å^3	4165.93(17)
Z	86
ρ_{calc}g/cm^3	1.2515
μ/mm^{-1}	0.078
F(000)	1656.7
Crystal size/mm^3	N/A × N/A × N/A
Radiation	Mo Kα (λ = 0.71073)
2Θ range for data collection/°	4.82 to 54.96
Index ranges	$-15 \le h \le 15$, $-18 \le k \le 18$, $-33 \le l \le 33$

Reflections collected	86348
Independent reflections	4768 [R_{int} = 0.0530, R_{sigma} = 0.0190]
Data/restraints/parameters	4768/0/271
Goodness-of-fit on F^2	1.044
Final R indexes [I>=2σ (I)]	R_1 = 0.0399, wR_2 = 0.1032
Final R indexes [all data]	R_1 = 0.0461, wR_2 = 0.1080
Largest diff. peak/hole / e $Å^{-3}$	0.13/-0.19
Flack parameter	-0.2(12)

Method for crystal growth：

The 10 mg of pure compound **3aa** was dissolved in 1 mL methanol in a little sample bottle. And 2 mL hexane was added dropwise in the bottle. Then, the bottle is sealed with plastic film, and two holes are made in the plastic film. The bottle was placed in a quiet environment.

Crystal measurement:

A methanol and hexane mixture of **3aa** (CCDC 2257445) were slowly evaporated at ambient temperature over a period of one day, to afford single crystals suitable for an X-ray crystallographic study. Single-crystal X-ray diffraction data were collected on a Rigaku XtaLAB Pro diffractometer with Cu-Kα radiation (λ =1.54184 Å) for compound **3aa**. The structure was solved by Direct Method of SHELXS-97 and refined by full-matrix least-squares techniques using the SHELXL-97 program. Non-hydrogen atoms were refined with anisotropic temperature parameters, and hydrogen atoms of the ligands were refined as rigid groups.

10. Computational studies.

All calculations were performed with the Gaussian 09[9] program by using density functional theory (DFT). All the geometries were optimized with the M06-2X[10] functional and 6-31G(d, p)[11,12] basis set. The solvent effect was considered using the implicit solvation model based on density (SMD) [13] and dodecane was selected as the solvent. Additionally, single-point energy calculations were performed at the M06-2X/6-311+G(d, p)[14,15]/$SMD_{dodecane}$ level. In summary, the discussed energies were obtained at the M06-2X/6-311+G(d, p)/$SMD_{dodecane}$//M06-2X/6-31G(d, p)/$SMD_{dodecane}$ level. The three-dimensional configurations involved in this work are all displayed through CYLview [16] software.

Calculational of rotation energy

For the racemization processes of the products **3aa** and **3ua**, the energy barriers of the dihedral Φ(C1−N1−N2−C2) rotation have been studied and calculated. As shown in Figure 1, the energy barriers of the dihedral rotation via transition states **TS^{3aa}** and **TS^{3ua}** are 40.3 kcal/mol and 41.2 kcal/mol, respectively, indicating the axially chiral products should be very stable in theory. All the calculations are in agreement with the experimental results.

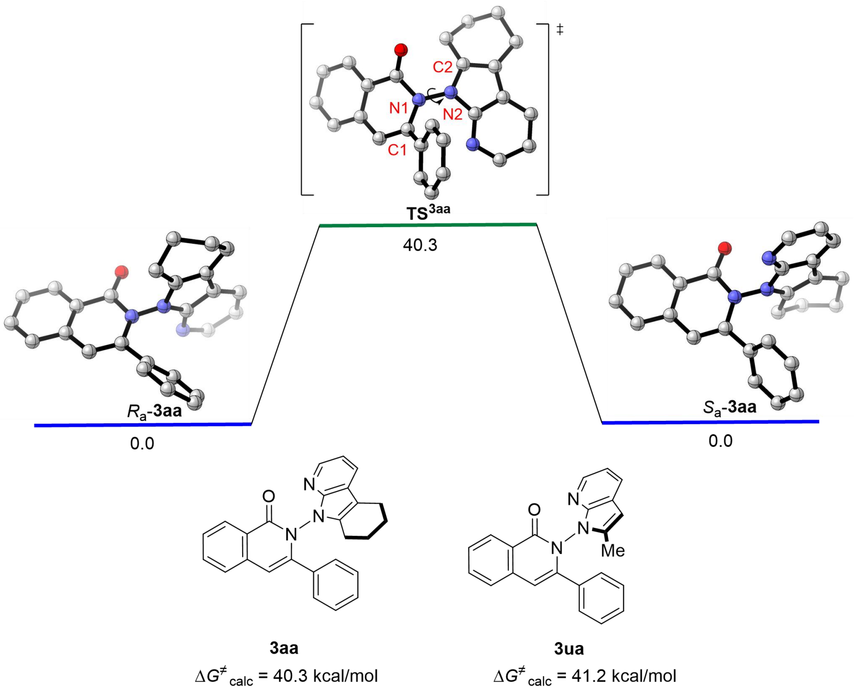

Supplementary Figure 6. Relative Gibbs free energy profiles associated with transition states **TS^A** and **TS^B**. (For clarity, we have hidden the hydrogens from the three-dimensional configurations.)

11. Characterization data and HPLC chromatograms

(*R*)-3-phenyl-2-(5,6,7,8-tetrahydro-9H-pyrido[2,3-b]indol-9-yl)isoquinolin-1(2H)-one (3aa)

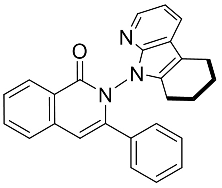

Yield: 70.4 mg (90%). White solid, mp: 150-151 ˚C. ^{1}**H NMR** (600 MHz, $CDCl_3$) δ 8.39 (d, *J* = 8.0 Hz, 1H), 8.25 (d, *J* = 4.7 Hz, 1H), 7.71 (dd, *J* = 14.1, 6.3 Hz, 1H), 7.67 (t, *J* = 11.6 Hz, 1H), 7.58 (d, *J* = 7.9 Hz, 1H), 7.50 (t, *J* = 7.6 Hz, 1H), 7.40 – 7.39 (m, 2H), 7.23 – 7.20 (m, 1H), 7.14 (t, *J* = 7.6 Hz, 2H), 7.05 (dd, *J* = 7.7, 4.9 Hz, 1H), 6.62 (s, 1H), 2.63 – 2.60 (m, 1H), 2.53 – 2.49 (m, 1H), 2.44 – 2.40 (m, 1H), 2.30 – 2.25 (m, 1H), 1.84 – 1.81 (m, 2H), 1.77 – 1.69 (m, 1H), 1.68 – 1.63 (m, 1H). ^{13}C NMR (151 MHz, $CDCl_3$) δ 161.4, 148.1, 145.1, 142.5, 136.8, 136.4, 133.9, 133.3, 128.9, 128.6, 128.5, 127.8, 127.0, 126.4, 126.1, 125.4, 119.3, 116.7, 108.8, 107.7, 22.6, 22.2, 21.1, 20.4. **HRMS** (ESI): m/z $[M+H]^+$calcd for $[C_{26}H_{22}N_3O]^+$ required 392.1758, found 392.1759. $[\alpha]_D^{25}$ = -97 (c = 0.1, CH_2Cl_2). The product was analyzed by HPLC to determine the enantiomeric excess: 98% ee (CHIRALPAK AS-H, hexane/*i*-PrOH =80/20, detector: 254 nm, T = 25 °C, flow rate: 1 mL/min), t_1 (minor) = 5.191 min, t_2 (major) = 6.262 min.

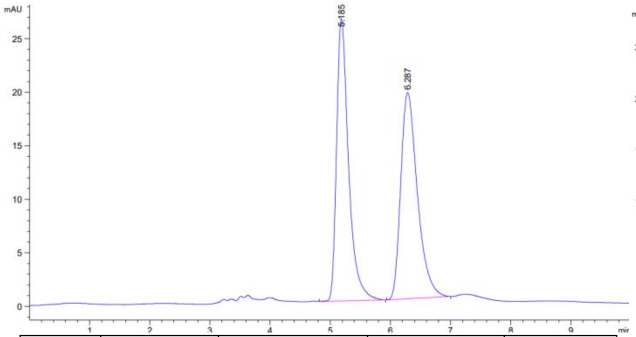

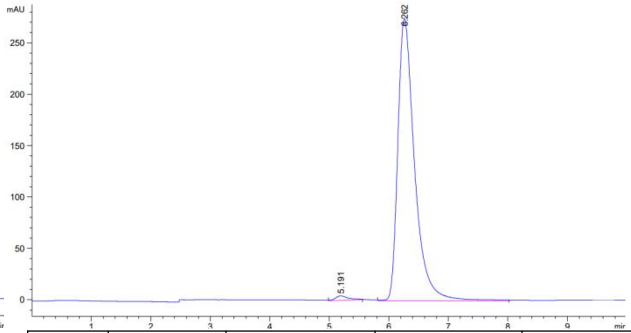

Peak	RetTime	Area	Height	Area
1	5.185	364.19531	26.34732	49.5701
2	6.287	370.51221	19.26932	50.4299

Peak	RetTime	Area	Height	Area
1	5.191	67.77026	4.43082	1.2438
2	6.262	5381.03711	274.56998	98.7562

(*R*)-6-fluoro-3-phenyl-2-(5,6,7,8-tetrahydro-9H-pyrido[2,3-b]indol-9-yl)isoquinolin-1(2H)-one (3ba)

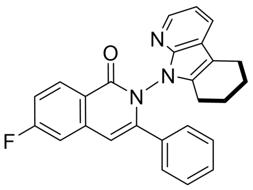

Yield: 51.5 mg (63%). White solid, mp: 190-191 ˚C. ^{1}**H NMR** (600 MHz, $CDCl_3$) δ 8.39 (dd, *J* = 8.7, 5.7 Hz, 1H), 8.24 – 8.23 (m, 1H), 7.67 (d, *J* = 7.7 Hz, 1H), 7.40 (d, *J* = 7.3 Hz, 2H), 7.24 – 7.17 (m, 3H), 7.14 (t, *J* = 7.7 Hz, 2H), 7.04 (dd, *J* = 7.7, 4.8 Hz, 1H), 6.56 (s, 1H), 2.64 – 2.59 (m, 1H), 2.52 – 2.49 (m, 1H), 2.43 – 2.39 (m, 1H), 2.30 – 2.25 (m, 1H), 1.85 – 1.80 (m, 2H), 1.73 – 1.68 (m, 1H), 1.67 – 1.62 (m, 1H). ^{13}C NMR (151 MHz, $CDCl_3$) δ 166.7, 165.0, 160.7, 147.4 ($^1J_{C-F}$ = 244.0 Hz), 142.7, 139.1 ($^3J_{C-F}$ = 10.3 Hz), 136.3, 133.5, 132.0 ($^3J_{C-F}$ = 10.1 Hz), 129.2, 128.4, 127.9, 126.1, 121.9, 119.3, 116.8, 115.6 ($^2J_{C-F}$ = 23.4 Hz), 111.4 ($^2J_{C-F}$ = 22.1 Hz), 108.9, 106.9 ($^3J_{C-F}$ = 3.3 Hz), 22.6, 22.2, 21.1, 20.3. ^{19}**F NMR** (565 MHz, $CDCl_3$) δ -140.8. **HRMS** (ESI): m/z $[M+H]^+$calcd for $[C_{26}H_{21}FN_3O]^+$ required 410.1663, found 410.1672. $[\alpha]_D^{25}$ = +41 (c = 0.1, CH_2Cl_2). The product was analyzed by HPLC to determine the enantiomeric excess: 96% ee (CHIRALPAK AS-H, hexane/*i*-PrOH =85/15, detector: 254 nm, T = 25 °C, flow rate: 1 mL/min), t_1 (minor) = 5.895 min, t_2 (major) = 7.842 min.

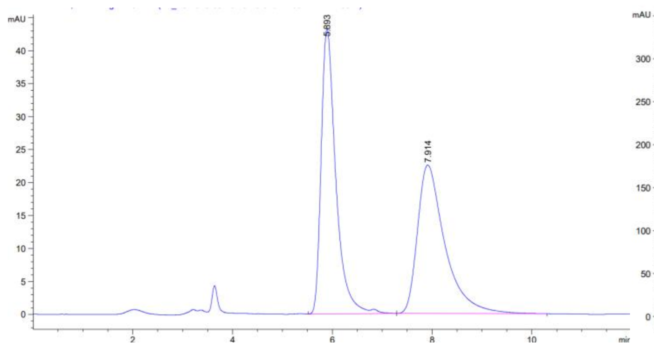

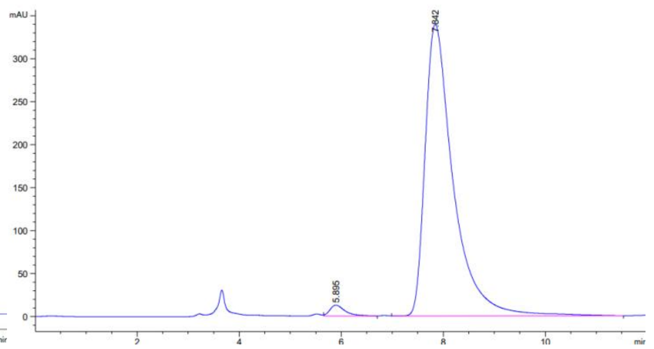

Peak	RetTime	Area	Height	Area
1	5.893	883.32672	43.21945	50.7137
2	7.914	858.46393	22.49776	49.2863

Peak	RetTime	Area	Height	Area
1	5.895	257.78522	12.61488	1.9400
2	7.842	1.30300e4	339.01884	98.0600

(*R*)-6-chloro-3-phenyl-2-(5,6,7,8-tetrahydro-9H-pyrido[2,3-b]indol-9-yl)isoquinolin-1(2H)-one (3ca)

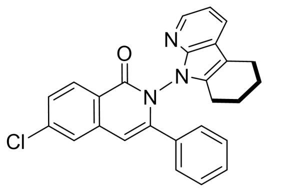

Yield: 72.2 mg (85%). White solid, mp: 140-141 ˚C. **^{1}H NMR** (600 MHz, $CDCl_3$) δ 8.30 (d, *J* = 8.6 Hz, 1H), 8.23 (d, *J* = 4.0 Hz, 1H), 7.67 (d, *J* = 7.7 Hz, 1H), 7.55 (t, *J* = 7.5 Hz, 1H), 7.43 (dd, *J* = 8.6, 1.9 Hz, 1H), 7.38 (t, *J* = 13.5 Hz, 2H), 7.23 – 7.21 (m, 1H), 7.15 – 7.13 (m, 2H), 7.05 – 7.03 (m, 1H), 6.54 (s, 1H), 2.62 – 2.59 (m, 1H), 2.51 – 2.48 (m, 1H), 2.42 – 2.37 (m, 1H), 2.29 – 2.24 (m, 1H), 1.83 – 1.79 (m, 2H), 1.74 – 1.68 (m, 1H), 1.67 – 1.63 (m, 1H). ^{13}C NMR (151 MHz, $CDCl_3$) δ 160.9, 148.1, 146.6, 142.6, 139.8, 138.0, 136.3, 133.5, 130.4, 129.2, 128.4, 127.9, 127.5, 126.2, 125.7, 123.6, 119.3, 116.8, 109.0, 106.6, 22.6, 22.2, 21.1, 20.3.**HRMS** (ESI): m/z $[M+H]^+$calcd for $[C_{26}H_{21}ClN_3O]^+$ required 426.1368, found 426.1370. $[\alpha]_D^{25}$ = -61(c = 0.1, CH_2Cl_2). The product was analyzed by HPLC to determine the enantiomeric excess: 95% ee (CHIRALPAK AS-H, hexane/*i*-PrOH =85/15, detector: 254 nm, T = 25 °C, flow rate: 1 mL/min), t_1 (minor) = 5.785 min, t_2 (major) = 7.931 min.

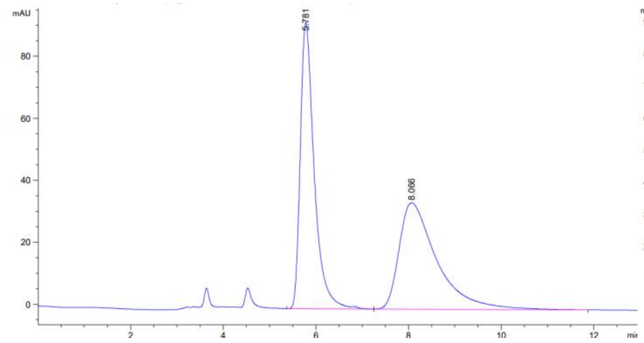

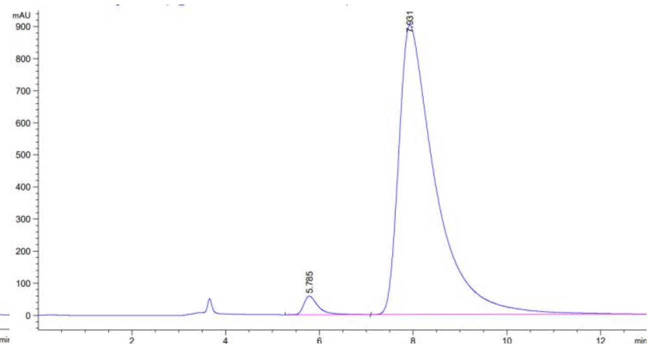

Peak	RetTime	Area	Height	Area
1	5.781	1928.41089	92.27707	49.8197
2	8.066	1942.37024	34.33122	50.1803

Peak	RetTime	Area	Height	Area
1	5.785	1241.77832	57.88111	2.4808
2	7.931	4.88141e4	902.77722	97.5192

(*R*)-6-bromo-3-phenyl-2-(5,6,7,8-tetrahydro-9H-pyrido[2,3-b]indol-9-yl)isoquinolin-1(2H)-one (3da)

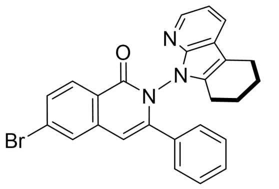

Yield: 81.6 mg (87%). White solid, mp: 156-157 ˚C. **^{1}H NMR** (600 MHz, $CDCl_3$) δ 8.22 (t, *J* = 6.0 Hz, 2H), 7.74 (s, 1H), 7.66 (t, *J* = 12.9 Hz, 1H), 7.58 (dd, *J* = 8.5, 1.4 Hz, 1H), 7.38 (d, *J* = 7.4 Hz, 2H), 7.23 (t, *J* = 7.4 Hz, 1H), 7.14 (t, *J* = 7.6 Hz, 2H), 7.04 (dd, *J* = 7.7, 4.8 Hz, 1H), 6.53 (s, 1H), 2.62 – 2.59 (m, 1H), 2.52 – 2.48 (m, 1H), 2.41 – 2.37 (m, 1H), 2.28 – 2.23 (m, 1H), 1.83 – 1.80 (m, 2H), 1.73 – 1.67 (m, 1H), 1.66 – 1.61 (m, 1H). ^{13}C NMR (151 MHz, $CDCl_3$) δ 161.0, 148.1, 146.6, 142.6, 138.1, 136.2, 133.5, 130.4, 129.2, 128.8, 128.5, 128.4, 127.9, 126.2, 124.0, 119.3, 116.8, 109.0, 106.4, 22.6, 22.2, 21.1, 20.3.**HRMS** (ESI): m/z $[M+H]^+$calcd for

$[C_{26}H_{21}BrN_3O]^+$ required 470.0863, found 470.0862. $[\alpha]_D^{25}$ = +78 (c = 0.1, CH_2Cl_2). The product was analyzed by HPLC to determine the enantiomeric excess: 96% ee (CHIRALPAK AS-H, hexane/*i*-PrOH =85/15, detector: 254 nm, T = 25 °C, flow rate: 1 mL/min), t_1 (minor) = 5.982 min, t_2 (major) = 8.505 min.

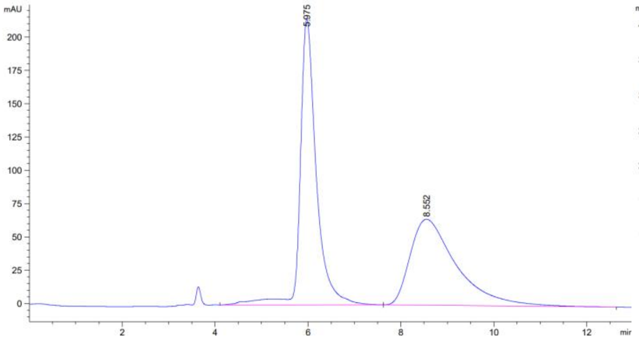

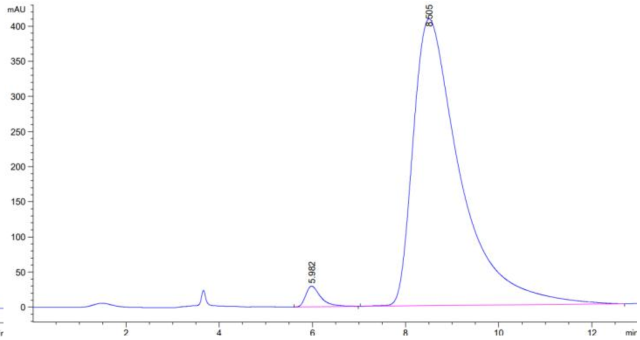

Peak	RetTime	Area	Height	Area
1	5.975	5168.78320	214.63582	53.6568
2	8.552	4464.26855	64.53683	46.3432

Peak	RetTime	Area	Height	Area
1	5.982	656.77502	29.42193	2.2451
2	8.505	2.85968e4	408.04346	97.7549

(*R*)-3-phenyl-2-(5,6,7,8-tetrahydro-9H-pyrido[2,3-b]indol-9-yl)-6-(trifluoromethyl)isoquinolin-1(2H)-one (3ea)

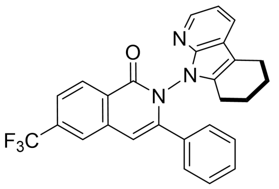

Yield: 58.8 mg (64%). White solid, mp: 169-170 ˚C. **^{1}H NMR** (600 MHz, $CDCl_3$) δ 8.49 (d, *J* = 8.3 Hz, 1H), 8.23 – 8.22 (m, 1H), 7.87 (s, 1H), 7.70 – 7.67 (m, 2H), 7.40 – 7.39 (m, 2H), 7.26 – 7.22 (m, 1H), 7.15 (t, *J* = 7.7 Hz, 2H), 7.05 (dd, *J* = 7.7, 4.8 Hz, 1H), 6.68 (s, 1H), 2.64 – 2.60 (m, 1H), 2.53 – 2.49 (m, 1H), 2.41 – 2.37 (m, 1H), 2.29 – 2.24 (m, 1H), 1.85 – 1.80 (m, 2H), 1.74 – 1.70 (m, 1H), 1.69 – 1.62 (m, 1H). ^{13}C NMR (151 MHz, $CDCl_3$) δ 160.7, 148.1, 146.9, 142.7, 136.8, 136.1 ($^2J_{C-F}$ = 32.0 Hz), 133.3, 129.8, 129.3, 128.5, 127.9, 127.4, 126.2, 123.6 ($^1J_{C-F}$ = 271.6 Hz), 123.7 ($^3J_{C-F}$ = 3.5 Hz), 123.0 ($^3J_{C-F}$ = 3.5 Hz), 119.3, 116.9, 109.2, 107.1, 22.6, 22.2, 21.1, 20.3. **^{19}F NMR** (565 MHz, $CDCl_3$) δ -63.1. **HRMS** (ESI): m/z $[M+H]^+$calcd for $[C_{27}H_{21}F_3N_3O]^+$ required 460.1631, found 460.1631. $[\alpha]_D^{25}$ = +30 (c = 0.1, CH_2Cl_2). The product was analyzed by HPLC to determine the enantiomeric excess: 95% ee (CHIRALPAK AS-H, hexane/*i*-PrOH =85/15, detector: 254 nm, T = 25 °C, flow rate: 1 mL/min), t_1 (minor) = 4.756 min, t_2 (major) = 6.282 min.

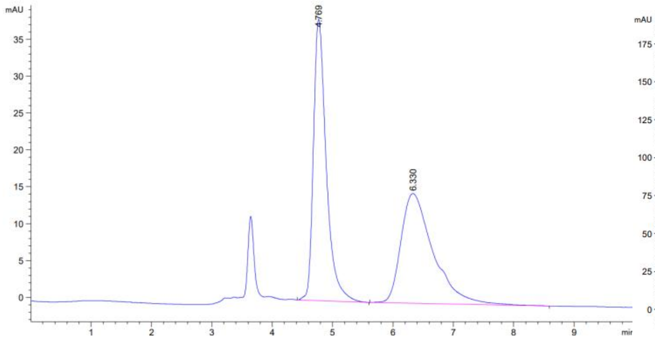

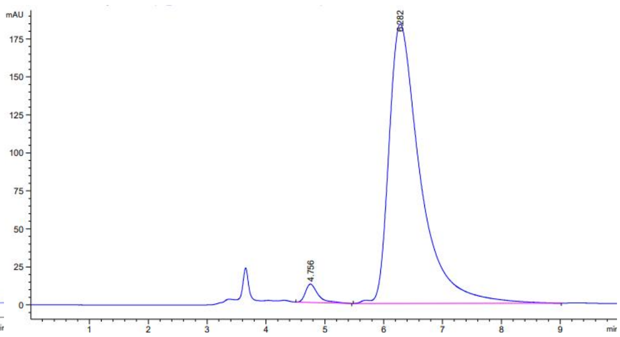

Peak	RetTime	Area	Height	Area
1	4.769	563.64697	38.14309	50.4798
2	6.330	552.93311	14.88607	49.5202

Peak	RetTime	Area	Height	Area
1	4.756	178.74133	12.03876	2.5609
2	6.282	6800.81494	183.75186	97.4391

(*R*)-6-methoxy-3-phenyl-2-(5,6,7,8-tetrahydro-9H-pyrido[2,3-b]indol-9-yl)isoquinolin-1(2H)-one (3fa)

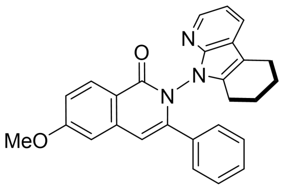

Yield: 80.8 mg (96%). White solid, mp: 132-133 °C. **^{1}H NMR** (600 MHz, $CDCl_3$) δ 8.29 (d, *J* = 8.9 Hz, 1H), 8.24 (dd, *J* = 4.7, 1.0 Hz, 1H), 7.65 (dd, *J* = 7.7, 1.1 Hz, 1H), 7.40 (d, *J* = 7.3 Hz, 2H), 7.19 (t, *J* = 7.4 Hz, 1H), 7.12 (t, *J* = 7.6 Hz, 2H), 7.05 – 7.01 (m, 2H), 6.94 (d, *J* = 2.4 Hz, 1H), 6.54 (s, 1H), 3.89 (s, 3H), 2.63 – 2.58 (m, 1H), 2.51 – 2.47 (m, 1H), 2.44 – 2.39 (m, 1H), 2.29 – 2.25 (m, 1H), 1.83 – 1.79 (m, 2H), 1.73 – 1.68 (m, 1H), 1.66 – 1.62 (m, 1H). ^{13}C NMR (151 MHz, $CDCl_3$) δ 163.6, 161.1, 148.3, 145.7, 142.6, 138.9, 136.5, 134.0, 130.7, 128.9, 128.5, 127.8, 126.0, 119.2, 119.0, 116.6, 116.3, 108.6, 107.5, 107.5, 55.6, 22.6, 22.2, 21.1, 20.4. **HRMS** (ESI): m/z [M+H]$^+$calcd for $[C_{27}H_{24}N_3O_2]^+$ required 422.1863, found 422.1860. $[\alpha]_D^{25}$ = +51 (c = 0.1, CH_2Cl_2). The product was analyzed by HPLC to determine the enantiomeric excess: 98% ee (CHIRALPAK AS-H, hexane/*i*-PrOH =85/15, detector: 254 nm, T = 25 °C, flow rate: 1 mL/min), t_1 (minor) = 7.824 min, t_2 (major) = 11.140 min.

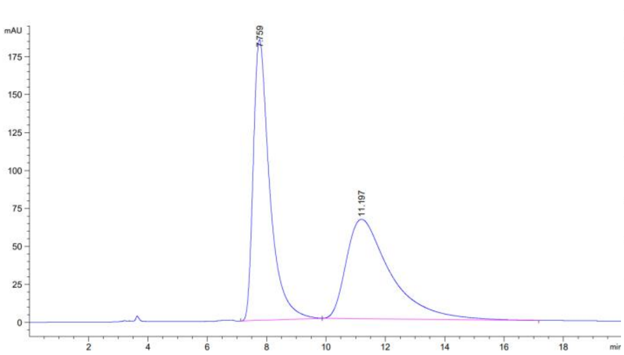

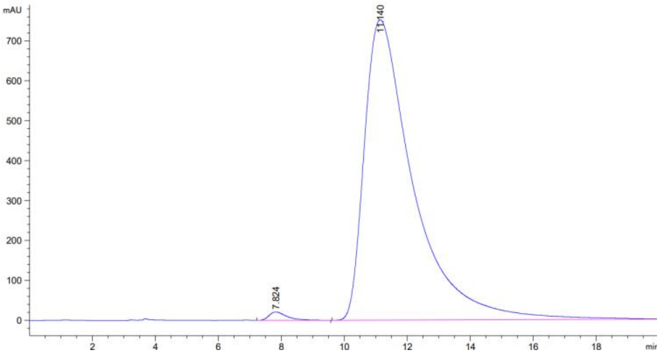

Peak	RetTime	Area	Height	Area
1	7.759	7123.31982	184.88353	51.4415
2	11.197	6724.08643	65.41141	48.5585

Peak	RetTime	Area	Height	Area
1	7.824	823.06061	20.99747	1.0040
2	11.140	8.11544e4	752.15204	98.9960

(*R*)-6-ethyl-3-phenyl-2-(5,6,7,8-tetrahydro-9H-pyrido[2,3-b]indol-9-yl)isoquinolin-1(2H)-one (3ga)

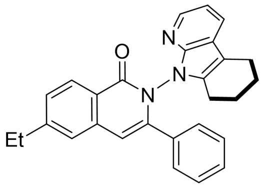

Yield: 77.1 mg (92%). White solid, 149-150. **^{1}H NMR** (600 MHz, $CDCl_3$) δ 8.31 (d, *J* = 8.2 Hz, 1H), 8.24 (d, *J* = 4.7 Hz, 1H), 7.66 (d, *J* = 7.7 Hz, 1H), 7.42 – 7.39 (m, 3H), 7.32 (dd, *J* = 28.5, 7.8 Hz, 1H), 7.20 (t, *J* = 7.4 Hz, 1H), 7.13 (t, *J* = 7.6 Hz, 2H), 7.03 (dd, *J* = 7.7, 4.8 Hz, 1H), 6.59 (s, 1H), 2.80 (q, *J* = 7.6 Hz, 2H), 2.63 – 2.59 (m, 1H), 2.52 – 2.48 (m, 1H), 2.44 – 2.40 (m, 1H), 2.30 – 2.25 (m, 1H), 1.84 – 1.81 (m, 2H), 1.72 – 1.69 (m, 1H), 1.67 – 1.63 (m, 1H), 1.32 (t, *J* = 7.6 Hz, 3H). ^{13}C NMR (151 MHz, $CDCl_3$) δ 163.6, 161.1, 148.3, 145.7, 142.6, 138.9, 136.5, 134.0, 130.7, 128.9, 128.5, 127.8, 126.0, 119.2, 119.0, 116.6, 116.3, 108.6, 107.5, 107.5, 55.6, 22.6, 22.2, 21.1, 20.4. **HRMS** (ESI): m/z [M+H]$^+$calcd for $[C_{28}H_{26}N_3O]^+$ required 420.2071, found 420.2070. $[\alpha]_D^{25}$ = +131 (c = 0.1, CH_2Cl_2). The product was analyzed by HPLC to determine the enantiomeric excess: 98% ee (CHIRALPAK AS-H, hexane/*i*-PrOH =85/15, detector: 254 nm, T = 25 °C, flow rate: 1 mL/min), t_1 (minor) = 5.121 min, t_2 (major) = 6.908 min.

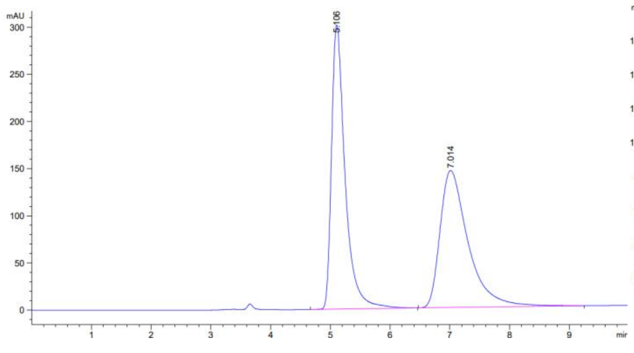

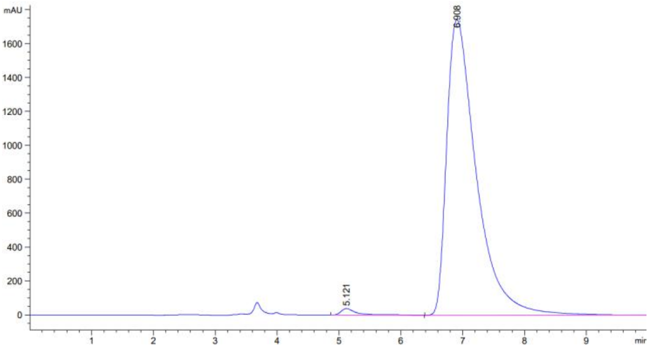

Peak	RetTime	Area	Height	Area
1	5.106	4758.43115	301.08920	50.0336
2	7.014	4752.04834	145.13837	49.9664

Peak	RetTime	Area	Height	Area
1	5.121	673.13916	37.82708	1.1487
2	6.908	5.79193e4	1741.39648	98.8513

(*R*)-6-(tert-butyl)-3-phenyl-2-(5,6,7,8-tetrahydro-9H-pyrido[2,3-b]indol-9-yl)isoquinolin-1(2H)-one (3ha)

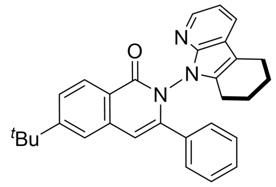

Yield: 85.8 mg (96%). White solid, mp: 155-156 ˚C. **^{1}H NMR** (600 MHz, $CDCl_3$) δ 8.35 (d, *J* = 9.0 Hz, 1H), 8.24 – 8.23 (m, 1H), 7.64 (t, *J* = 16.2 Hz, 1H), 7.58 (d, *J* = 7.2 Hz, 2H), 7.42 (d, *J* = 7.3 Hz, 2H), 7.19 (t, *J* = 7.4 Hz, 1H), 7.13 (t, *J* = 7.6 Hz, 2H), 7.02 (dd, *J* = 19.3, 9.7 Hz, 1H), 6.64 (s, 1H), 2.63 – 2.60 (m, 1H), 2.52 – 2.49 (m, 1H), 2.46 – 2.41 (m, 1H), 2.31 – 2.27 (m, 1H), 1.84 – 1.82 (m, 2H), 1.73 – 1.70 (m, 1H), 1.68 – 1.64 (m, 1H), 1.42 (s, 9H). ^{13}C NMR (151 MHz, $CDCl_3$) δ 161.3, 157.0, 148.3, 145.0, 142.6, 136.8, 136.5, 134.1, 128.9, 128.6, 128.5, 127.8, 126.0, 125.1, 123.1, 122.5, 119.2, 116.6, 108.6, 108.1, 35.3, 31.2, 22.7, 22.3, 21.2, 20.4. **HRMS** (ESI): m/z $[M+H]^+$calcd for $[C_{30}H_{30}N_3O]^+$ required 448.2384, found 448.2383. $[\alpha]_D^{25}$ = -125 (c = 0.1, CH_2Cl_2). The product was analyzed by HPLC to determine the enantiomeric excess: 99% ee (CHIRALPAK AS-H, hexane/*i*-PrOH =85/15, detector: 254 nm, T = 25 °C, flow rate: 1 mL/min), t_1 (minor) = 4.567 min, t_2 (major) = 6.318 min.

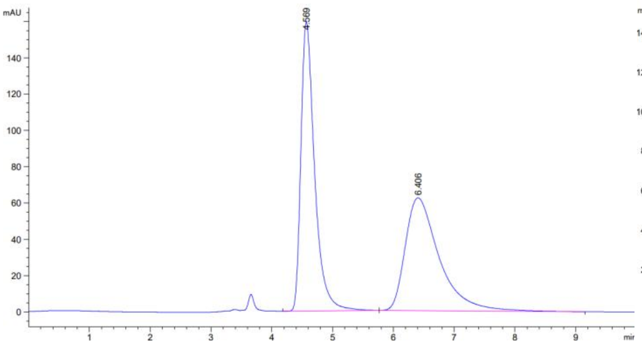

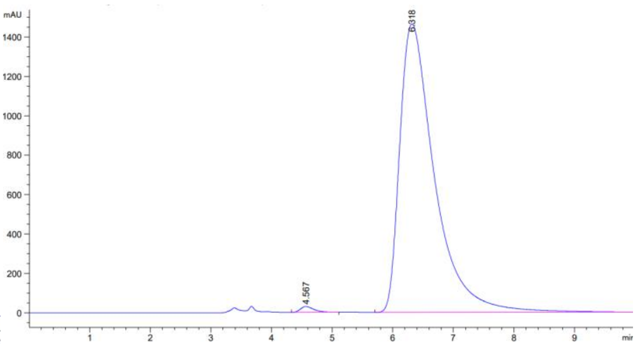

Peak	RetTime	Area	Height	Area
1	4.569	2497.99194	159.20494	50.8177
2	6.406	2417.60327	62.13045	49.1823

Peak	RetTime	Area	Height	Area
1	4.567	436.76187	29.08740	0.7421
2	6.318	5.84144e4	1464.00330	99.2579

(*R*)-3,6-diphenyl-2-(5,6,7,8-tetrahydro-9H-pyrido[2,3-b]indol-9-yl)isoquinolin-1(2H)-one (3ia)

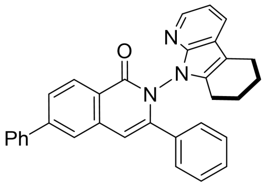

Yield: 90.6 mg (97%). White solid, mp: 140-141 ˚C. **^{1}H NMR** (600 MHz, $CDCl_3$) δ 8.47 (d, *J* = 8.3 Hz, 1H), 8.27 (dd, *J* = 4.7, 1.1 Hz, 1H), 7.78 (t, *J* = 8.7 Hz, 1H), 7.74 (dd, *J* = 8.3, 1.6 Hz, 1H), 7.71 (d, *J* = 7.3 Hz, 2H), 7.68 (dd, *J* = 7.7, 1.1 Hz, 1H), 7.53 – 7.48 (m, 2H), 7.48 – 7.39 (m, 3H), 7.23 (t, *J* = 7.4 Hz, 1H), 7.16 (t, *J* = 7.6 Hz, 2H), 7.05 (dd, *J* = 7.7, 4.8 Hz, 1H), 6.69 (s, 1H), 2.66 – 2.61 (m, 1H), 2.55 – 2.50 (m, 1H), 2.48 – 2.44 (m, 1H), 2.34 – 2.30 (m, 1H), 1.87 – 1.82 (m, 2H), 1.76 – 1.70 (m, 1H), 1.69 – 1.65 (m, 1H). ^{13}C NMR (151 MHz,

$CDCl_3$) δ 161.4, 146.1, 145.6, 142.6, 140.0, 137.2, 136.5, 133.9, 129.3, 129.1, 129.0, 128.6, 128.4, 127.8, 127.5, 126.2, 126.1, 124.6, 124.2, 119.3, 116.7, 108.8, 107.9, 22.7, 22.3, 21.2, 20.4. **HRMS** (ESI): m/z $[M+H]^+$calcd for $[C_{32}H_{26}N_3O]^+$ required 468.2071, found 468.2071. $[\alpha]_D^{25}$ = +55 (c = 0.1, CH_2Cl_2). The product was analyzed by HPLC to determine the enantiomeric excess: 98% ee (CHIRALPAK AS-H, hexane/*i*-PrOH =85/15, detector: 254 nm, T = 25 °C, flow rate: 1 mL/min), t_1 (minor) = 6.716 min, t_2 (major) = 8.273 min.

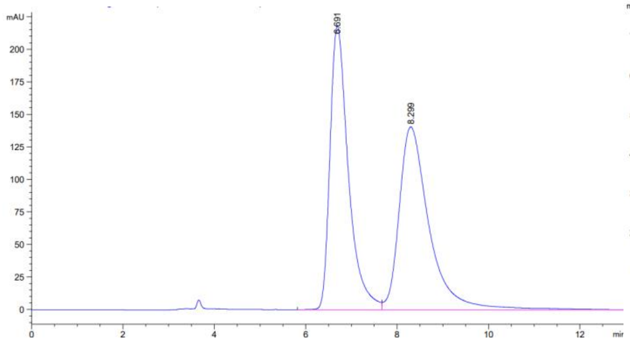

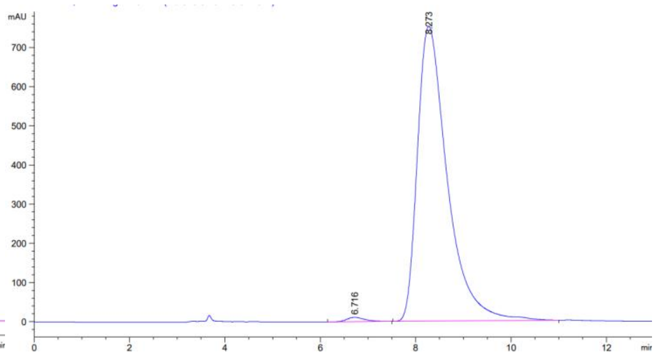

Peak	RetTime	Area	Height	Area
1	6.691	6086.98145	217.82425	48.7531
2	8.299	6398.34277	140.49390	51.2469

Peak	RetTime	Area	Height	Area
1	6.716	286.69348	10.96583	0.8669
2	8.273	3.27848e4	752.84906	99.1331

(*R*)-5-bromo-3-phenyl-2-(5,6,7,8-tetrahydro-9H-pyrido[2,3-b]indol-9-yl)isoquinolin-1(2H)-one (3ja)

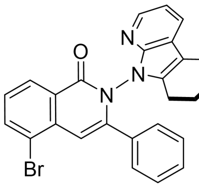

Yield: 65.6 mg (70%). White solid, mp: 160-161 ˚C. **^{1}H NMR** (600 MHz, $CDCl_3$) δ 8.51 (d, *J* = 1.9 Hz, 1H), 8.22 (dd, *J* = 4.7, 1.0 Hz, 1H), 7.78 (dd, *J* = 8.5, 2.1 Hz, 1H), 7.67 (dd, *J* = 7.7, 1.0 Hz, 1H), 7.45 (d, *J* = 8.5 Hz, 1H), 7.38 (d, *J* = 7.3 Hz, 2H), 7.22 (t, *J* = 7.5 Hz, 1H), 7.13 (t, *J* = 7.7 Hz, 2H), 7.04 (dd, *J* = 7.7, 4.8 Hz, 1H), 6.58 (s, 1H), 2.63 – 2.59 (m, 1H), 2.53 – 2.48 (m, 1H), 2.42 – 2.37 (m, 1H), 2.29 – 2.24 (m, 1H), 1.85 – 1.80 (m, 2H), 1.74 – 1.70 (m, 1H), 1.68 – 1.64 (m, 1H). ^{13}C NMR (151 MHz, $CDCl_3$) δ 160.3, 148.0, 145.7, 142.6, 136.4, 136.2, 135.4, 133.5, 131.1, 129.1, 128.4, 128.0, 127.8, 126.6, 126.1, 120.7, 119.3, 116.8, 109.0, 107.0, 22.6, 22.2, 21.1, 20.3.**HRMS** (ESI): m/z $[M+H]^+$calcd for $[C_{26}H_{21}BrN_3O]^+$ required 470.0863, found 470.0864. $[\alpha]_D^{25}$ = +31 (c = 0.1, CH_2Cl_2). The product was analyzed by HPLC to determine the enantiomeric excess: 94% ee (CHIRALPAK AS-H, hexane/*i*-PrOH =85/15, detector: 254 nm, T = 25 °C, flow rate: 1 mL/min), t_1 (minor) = 5.929 min, t_2 (major) = 7.477 min.

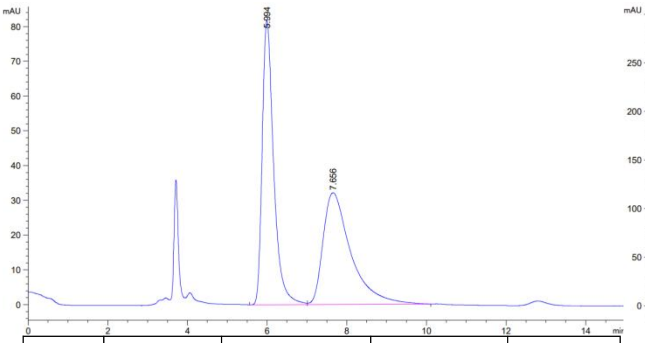

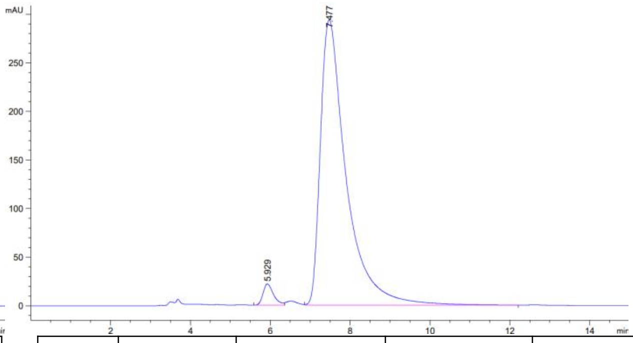

Peak	RetTime	Area	Height	Area
1	5.994	1636.80725	81.72443	51.1974
2	7.656	1560.24280	32.14521	48.8026

Peak	RetTime	Area	Height	Area
1	5.929	398.66956	21.76585	2.9336
2	7.477	1.31911e4	293.60129	97.0664

(*R*)-8-chloro-3-phenyl-2-(5,6,7,8-tetrahydro-9H-pyrido[2,3-b]indol-9-yl)isoquinolin-1(2H)-one (3ka)

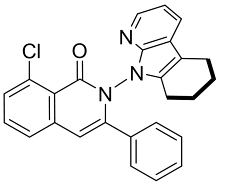

Yield: 55.2 mg (65%). White solid, mp: 136-137 °C. **^{1}H NMR** (600 MHz, $CDCl_3$) δ 8.23 (dd, *J* = 4.7, 1.0 Hz, 1H), 7.65 (dd, *J* = 7.7, 1.1 Hz, 1H), 7.52 (t, *J* = 7.8 Hz, 1H), 7.47 – 7.44 (m, 2H), 7.41 (d, *J* = 7.2 Hz, 2H), 7.22 (t, *J* = 7.4 Hz, 1H), 7.14 (t, *J* = 7.6 Hz, 2H), 7.03 (dd, *J* = 7.7, 4.8 Hz, 1H), 6.55 (s, 1H), 2.62 – 2.57 (m, 1H), 2.52 – 2.48 (m, 1H), 2.44 – 2.40 (m, 1H), 2.29 – 2.24 (m, 1H), 1.85 – 1.80 (m, 2H), 1.73 – 1.69 (m, 1H), 1.68 – 1.61 (m, 1H). ^{13}C NMR (151 MHz, $CDCl_3$) δ 159.3, 148.1, 146.1, 142.6, 139.7, 136.4, 136.3, 133.6, 133.0, 130.1, 129.1, 128.3, 127.9, 126.1, 125.6, 121.8, 119.4, 116.7, 108.9, 107.1, 22.6, 22.2, 21.1, 20.4. **HRMS** (ESI): m/z $[M+H]^+$calcd for $[C_{26}H_{21}ClN_3O]^+$ required 426.1368, found 426.1368. $[\alpha]_D^{25}$ = -48 (c = 0.1, CH_2Cl_2). The product was analyzed by HPLC to determine the enantiomeric excess: 96% ee (CHIRALPAK AS-H, hexane/*i*-PrOH =85/15, detector: 254 nm, T = 25 °C, flow rate: 1 mL/min), t_1 (minor) = 6.421 min, t_2 (major) = 7.685 min.

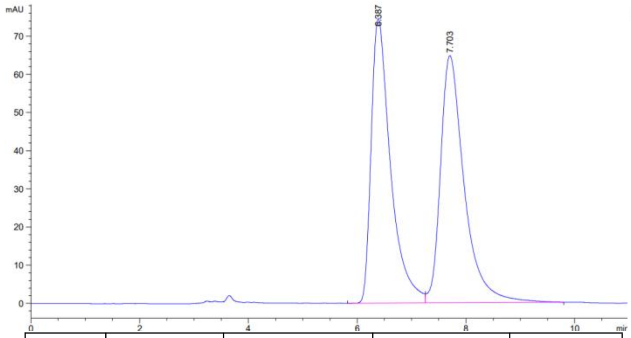

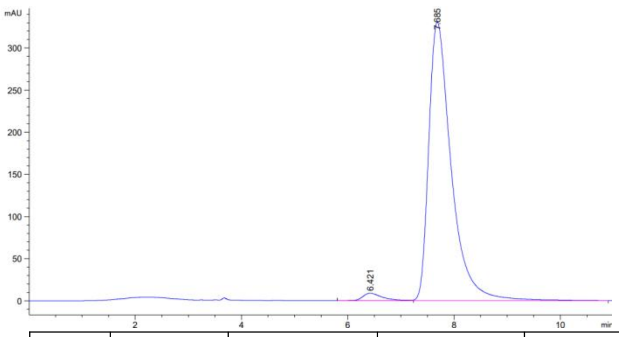

Peak	RetTime	Area	Height	Area
1	6.387	1846.94214	74.53737	48.4519
2	7.703	1964.97021	64.76451	51.5481

Peak	RetTime	Area	Height	Area
1	6.421	221.05559	8.75075	2.2224
2	7.685	9725.84473	330.45645	97.7776

(*R*)-8-methyl-3-phenyl-2-(5,6,7,8-tetrahydro-9H-pyrido[2,3-b]indol-9-yl)isoquinolin-1(2H)-one (3la)

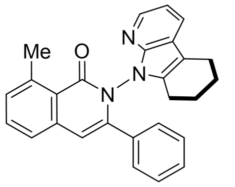

Yield: 55.1 mg (68%). White solid, mp: 115-116 °C. **^{1}H NMR** (600 MHz, $CDCl_3$) δ 8.25 (dd, *J* = 4.8, 1.1 Hz, 1H), 7.67 (dd, *J* = 7.7, 1.2 Hz, 1H), 7.53 (t, *J* = 7.6 Hz, 1H), 7.40 (dd, *J* = 8.8, 7.7 Hz, 3H), 7.26 – 7.24 (m, 1H), 7.20 (d, *J* = 7.4 Hz, 1H), 7.13 (t, *J* = 7.6 Hz, 2H), 7.04 (dd, *J* = 7.7, 4.8 Hz, 1H), 6.55 (s, 1H), 2.84 (s, 3H), 2.65 – 2.60 (m, 1H), 2.53 – 2.49 (m, 1H), 2.44 – 2.40 (m, 1H), 2.31 – 2.27 (m, 1H), 1.87 – 1.81 (m, 2H), 1.73 – 1.70 (m, 1H), 1.67 – 1.63 (m, 1H). ^{13}C NMR (151 MHz, $CDCl_3$) δ 161.8, 148.1, 144.8, 142.8, 142.6, 138.4, 136.5, 134.0, 132.5, 130.1, 128.8, 128.4, 127.8, 126.1, 124.8, 123.8, 119.2, 116.6, 108.5, 107.9, 23.6, 22.7, 22.3, 21.2, 20.4. **HRMS** (ESI): m/z $[M+H]^+$calcd for $[C_{27}H_{24}N_3O]^+$ required 406.1914, found 406.1920. $[\alpha]_D^{25}$ = -94 (c = 0.1, CH_2Cl_2). The product was analyzed by HPLC to determine the enantiomeric excess: 96% ee (CHIRALPAK AS-H, hexane/*i*-PrOH =85/15, detector: 254 nm, T = 25 °C, flow rate: 1 mL/min), t_1 (minor) = 4.933 min, t_2 (major) = 5.398 min.

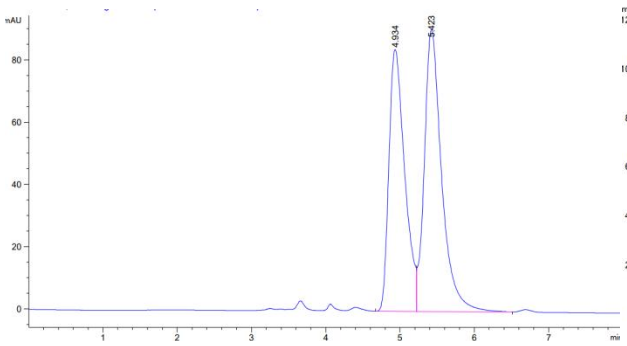

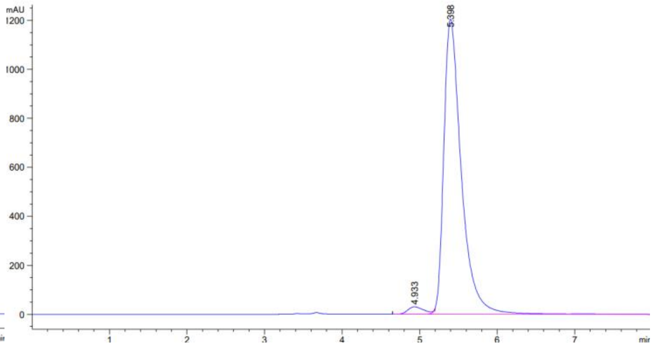

Peak	RetTime	Area	Height	Area

Peak	RetTime	Area	Height	Area

1	4.934	1243.06262	84.09695	45.8102
2	5.423	1470.44263	90.60947	54.1898

1	4.933	426.68329	30.13373	2.2032
2	5.398	1.89396e4	1204.07751	97.7968

(*R*)-5,7-dimethyl-3-phenyl-2-(5,6,7,8-tetrahydro-9H-pyrido[2,3-b]indol-9-yl)isoquinolin-1(2H)-one (3ma)

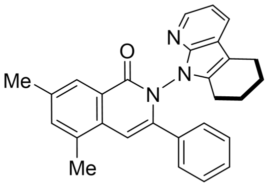

Yield: 62.0 mg (74%). White solid, mp: 150-151 ˚C. **^{1}H NMR** (600 MHz, $CDCl_3$) δ 8.22 (dd, *J* = 4.8, 1.2 Hz, 1H), 8.07 (s, 1H), 7.66 (dd, *J* = 7.7, 1.3 Hz, 1H), 7.41 – 7.40 (m, 2H), 7.38 (s, 1H), 7.21 (t, *J* = 7.4 Hz, 1H), 7.14 (t, *J* = 7.6 Hz, 2H), 7.03 (dd, *J* = 7.7, 4.8 Hz, 1H), 6.70 (s, 1H), 2.65 – 2.560 (m, 1H), 2.54 (s, 3H), 2.52 – 2.49 (m, 1H), 2.44 (s, 3H), 2.43 – 2.40 (m, 1H), 2.31 – 2.26 (m, 1H), 1.86 – 1.80 (m, 2H), 1.74 – 1.69 (m, 1H), 1.68 – 1.64 (m, 1H). ^{13}C NMR (151 MHz, $CDCl_3$) δ 161.6, 148.1, 143.6, 142.6, 136.9, 136.5, 135.7, 134.4, 133.6, 133.2, 128.8, 128.6, 127.7, 126.2, 126.0, 125.6, 119.2, 116.6, 108.6, 104.5, 22.7, 22.3, 21.4, 21.2, 20.4, 19.0. **HRMS** (ESI): m/z $[M+H]^+$calcd for $[C_{28}H_{26}N_3O]^+$ required 420.2071, found 420.2076. $[\alpha]_D^{25}$ = -96 (c = 0.1, CH_2Cl_2). The product was analyzed by HPLC to determine the enantiomeric excess: 99% ee (CHIRALPAK AD-H, hexane/*i*-PrOH =85/15, detector: 254 nm, T = 25 ºC, flow rate: 1 mL/min), t_1 (minor) = 5.746 min, t_2 (major) = 7.957 min.

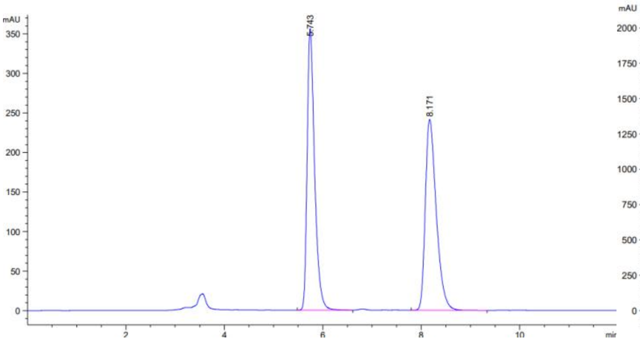

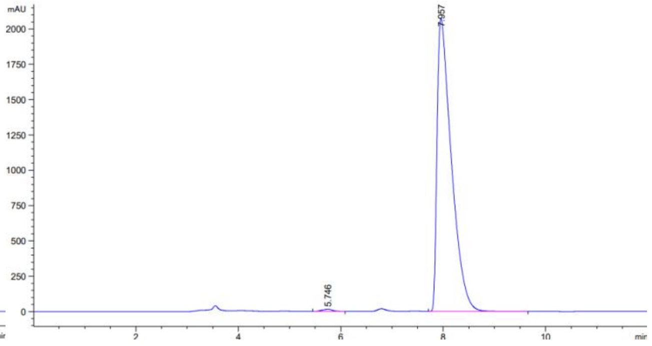

Peak	RetTime	Area	Height	Area
1	5.743	3743.98340	355.89410	50.1027
2	8.171	3728.64038	241.42502	49.8973

Peak	RetTime	Area	Height	Area
1	5.746	221.07347	16.13316	0.5465
2	7.685	4.02345e4	2071.71289	99.4535

(*R*)-5,7-dimethoxy-3-phenyl-2-(5,6,7,8-tetrahydro-9H-pyrido[2,3-b]indol-9-yl)isoquinolin-1(2H)-one (3na)

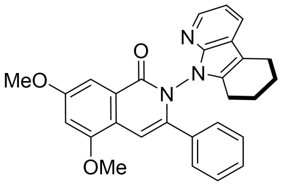

Yield: 76.6 mg (85%). White solid, mp: 166-167 ˚C. **^{1}H NMR** (600 MHz, $CDCl_3$) δ 8.25 (dd, *J* = 4.8, 1.3 Hz, 1H), 7.67 (dd, *J* = 7.7, 1.4 Hz, 1H), 7.40 – 7.39 (m, 3H), 7.20 – 7.17 (m, 1H), 7.12 (t, *J* = 7.6 Hz, 2H), 7.04 (dd, *J* = 7.7, 4.8 Hz, 1H), 6.97 (s, 1H), 6.75 (d, *J* = 2.4 Hz, 1H), 3.93 (s, 3H), 3.88 (s, 3H), 2.64 – 2.5 (m, 1H), 2.54 – 2.49 (m, 1H), 2.41 – 2.36 (m, 1H), 2.38 – 2.24 (m, 1H), 1.85 – 1.78 (m, 2H), 1.73 – 1.68 (m, 1H), 1.67 – 1.61 (m, 1H). ^{13}C NMR (151 MHz, $CDCl_3$) δ 161.0, 159.6, 156.1, 148.2, 142.5, 141.9, 136.4, 134.3, 128.7, 128.6, 127.7, 127.1, 126.0, 122.5, 119.3, 116.6, 108.7, 103.9, 102.2, 99.8, 55.9, 55.7, 22.7, 22.2, 21.1, 20.4. **HRMS** (ESI): m/z $[M+H]^+$calcd for $[C_{28}H_{26}N_3O_3]^+$ required 452.1969, found 452.1970. $[\alpha]_D^{25}$ =+66 (c = 0.1, CH_2Cl_2). The product was analyzed by HPLC to determine the enantiomeric excess: 99% ee (CHIRALPAK AD-H, hexane/*i*-PrOH =85/15, detector: 254 nm, T = 25 ºC, flow rate: 1 mL/min), t_1 (minor) = 5.746 min, t_2 (major) = 7.685 min.

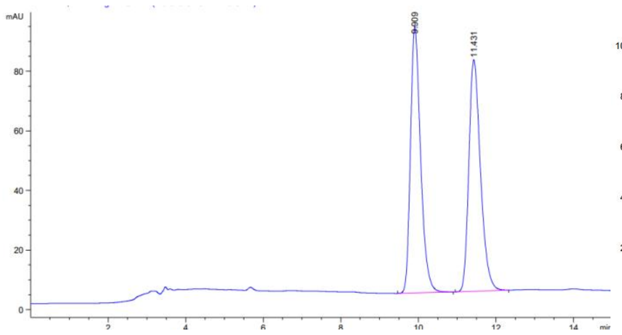

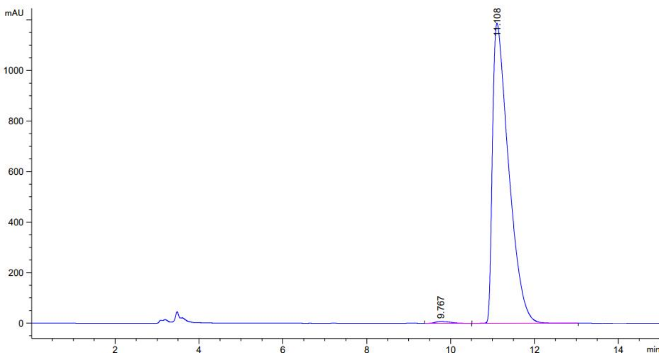

Peak	RetTime	Area	Height	Area
1	9.909	1621.15466	89.57498	50.0057
2	11.431	1620.78809	77.71816	49.9943

Peak	RetTime	Area	Height	Area
1	9.767	187.37732	7.48221	0.5865
2	11.108	3.17605e4	1188.02417	99.4135

(*R*)-5,6,7-trimethoxy-3-phenyl-2-(5,6,7,8-tetrahydro-9H-pyrido[2,3-b]indol-9-yl)isoquinolin-1(2H)-one (3oa)

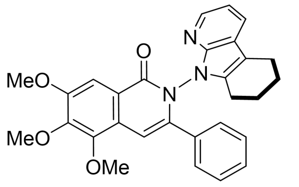

Yield: 66.4 mg (69%). White solid, mp: 184-185 ˚C. **^{1}H NMR** (600 MHz, $CDCl_3$) δ 8.23 (dd, *J* = 4.8, 1.2 Hz, 1H), 7.67 (dd, *J* = 7.7, 1.3 Hz, 1H), 7.63 (s, 1H), 7.42 – 7.37 (m, 2H), 7.21 (t, *J* = 7.4 Hz, 1H), 7.14 (t, *J* = 7.6 Hz, 2H), 7.04 (dd, *J* = 7.7, 4.8 Hz, 1H), 6.87 (s, 1H), 4.01 (s, 3H), 3.98 (s, 3H), 3.93 (s, 3H), 2.64 – 2.59 (m, 1H), 2.53 – 2.49 (m, 1H), 2.41 – 2.36 (m, 1H), 2.28 – 2.23 (m, 1H), 1.84 – 1.78 (m, 2H), 1.72 – 1.67 (m, 1H), 1.66 – 1.62 (m, 1H). ^{13}C NMR (151 MHz, $CDCl_3$) δ 160.7, 153.4, 148.3, 148.0, 146.7, 143.1, 142.6, 136.5, 134.2, 128.7, 127.8, 126.5, 126.0, 121.3, 119.3, 116.6, 108.7, 105.0, 102.0, 61.6, 61.1, 56.1, 22.7, 22.2, 21.1, 20.4. **HRMS** (ESI): m/z $[M+H]^+$calcd for $[C_{29}H_{28}N_3O_4]^+$ required 482.2075, found 482.2083. $[\alpha]_D^{25}$ =+28 (c = 0.1, CH_2Cl_2). The product was analyzed by HPLC to determine the enantiomeric excess: 97% ee (CHIRALPAK AD-H, hexane/*i*-PrOH =85/15, detector: 254 nm, T = 25 ºC, flow rate: 1 mL/min), t_1 (minor) = 7.611 min, t_2 (major) = 9.456 min.

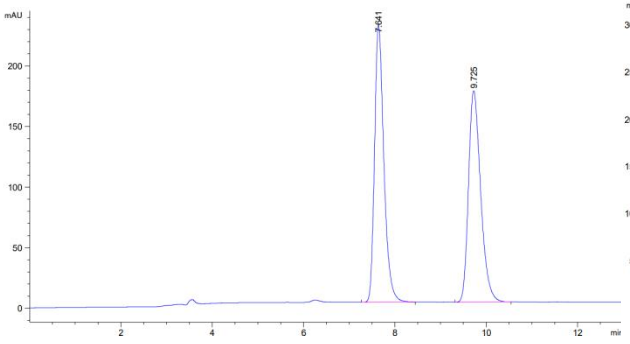

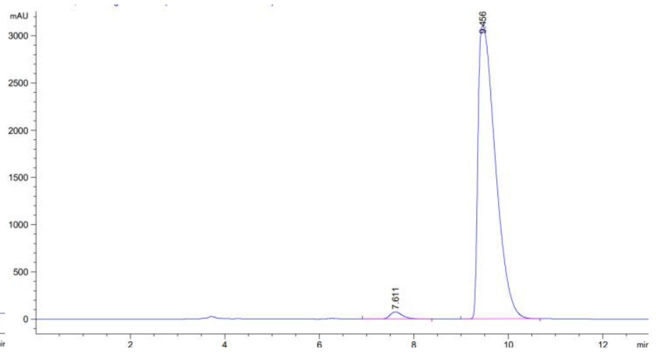

Peak	RetTime	Area	Height	Area
1	7.641	3247.80786	228.94188	50.1904
2	9.725	3223.16064	174.44185	49.8096

Peak	RetTime	Area	Height	Area
1	7.611	1386.74011	75.45431	1.7247
2	9.456	7.90173e4	3103.45264	98.2753

(*R*)-5-phenyl-6-(5,6,7,8-tetrahydro-9H-pyrido[2,3-b]indol-9-yl)thieno[2,3-c]pyridin-7(6H)-one (3pa)

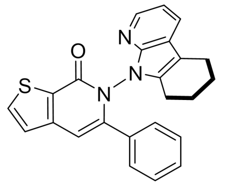

Yield: 55.6 mg (70%). White solid, mp: 137-138 ˚C. **^{1}H NMR** (600 MHz, $CDCl_3$) δ 8.23 (d, *J* = 4.8 Hz, 1H), 7.77 (dd, *J* = 5.1, 1.5 Hz, 1H), 7.65 (dd, *J* = 7.7, 1.2 Hz, 1H), 7.38 – 7.37 (m, 2H), 7.27 (d, *J* = 5.2 Hz, 1H), 7.21 (t, *J* = 7.4 Hz, 1H), 7.13 (t, *J* = 7.7 Hz, 2H), 7.03 (dd, *J* = 7.7, 4.8 Hz, 1H), 6.76 (s, 1H), 2.63 – 2.58 (m, 1H), 2.53 – 2.47 (m, 1H), 2.46 – 2.42 (m, 1H), 2.30 – 2.25 (m, 1H), 1.86 – 1.80 (m, 2H), 1.74 – 1.69 (m, 1H), 1.68 – 1.62 (m, 1H). ^{13}C NMR (151 MHz, $CDCl_3$) δ 157.3, 148.2, 146.9, 145.2, 142.6, 136.5, 134.9, 133.9, 129.0, 128.6, 127.8, 126.1, 124.7,

119.3, 116.7, 108.8, 104.4, 22.6, 22.2, 21.2, 20.4. **HRMS** (ESI): m/z $[M+H]^+$calcd for $[C_{24}H_{20}N_3OS]^+$ required 398.1322, found 398.1327. $[\alpha]_D^{25}$ = +94 (c = 0.1, CH_2Cl_2). The product was analyzed by HPLC to determine the enantiomeric excess: 99% ee (CHIRALPAK AS-H, hexane/*i*-PrOH =85/15, detector: 254 nm, T = 25 ^oC, flow rate: 1 mL/min), t_1 (minor) = 7.936 min, t_2 (major) = 9.864 min.

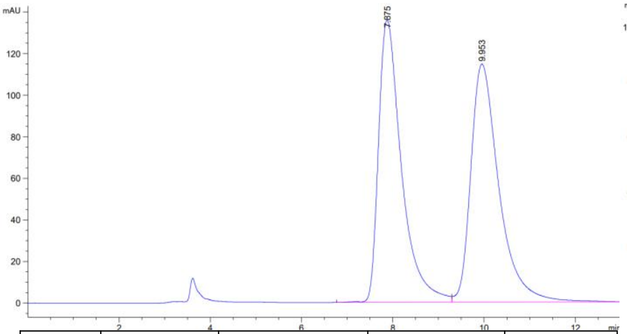

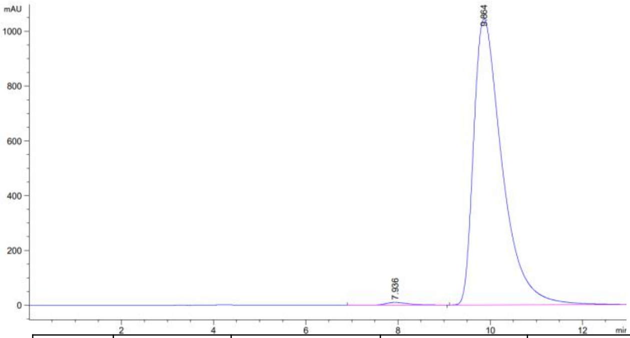

Peak	RetTime	Area	Height	Area
1	7.875	4752.28467	135.81160	49.1726
2	9.953	4912.21875	114.54189	50.8274

Peak	RetTime	Area	Height	Area
1	7.936	330.91605	9.93288	0.7323
2	9.864	4.48594e4	1045.06067	99.2677

(*R*)-3,4-dimethyl-6-phenyl-1-(5,6,7,8-tetrahydro-9H-pyrido[2,3-b]indol-9-yl)pyridin-2(1H)-one (3qa)

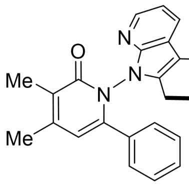

Yield: 48.7 mg (66%). White solid, mp: 112-113 ˚C. **^{1}H NMR** (600 MHz, $CDCl_3$) δ 8.23 (d, *J* = 4.6 Hz, 1H), 7.65 (d, *J* = 7.6 Hz, 1H), 7.32 (d, *J* = 7.5 Hz, 2H), 7.19 (t, *J* = 7.4 Hz, 1H), 7.12 (t, *J* = 7.6 Hz, 2H), 7.02 (dd, *J* = 7.6, 4.9 Hz, 1H), 6.15 (s, 1H), 2.62 – 2.57 (m, 1H), 2.52 – 2.48 (m, 1H), 2.42 – 2.37 (m, 1H), 2.25 (s, 3H), 2.23 – 2.18 (m, 1H), 2.14 (s, 3H), 1.85 – 1.81(m, 2H), 1.73 – 1.70 (m, 1H), 1.65 – 1.61 (m, 1H). ^{13}C NMR (151 MHz, $CDCl_3$) δ 161.5, 148.1, 146.7, 142.6, 136.4, 133.6, 128.9, 128.3, 127.8, 126.0, 125.8, 119.4, 116.6, 110.7, 108.8, 36.6, 28.0, 22.6, 22.2, 21.1, 20.3, 20.1, 13.0. **HRMS** (ESI): m/z $[M+H]^+$calcd for $[C_{24}H_{24}N_3O]^+$ required 370.1914, found 370.1915. $[\alpha]_D^{25}$ =+181 (c = 0.1, CH_2Cl_2). The product was analyzed by HPLC to determine the enantiomeric excess: 98% ee (CHIRALPAK AS-H, hexane/*i*-PrOH =85/15, detector: 254 nm, T = 25 ^oC, flow rate: 1 mL/min), t_1 (minor) = 4.843 min, t_2 (major) = 5.369 min.

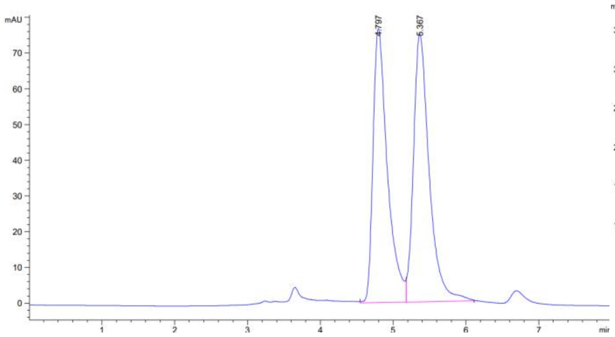

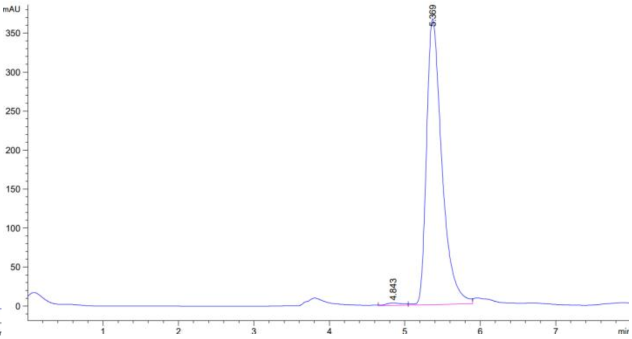

Peak	RetTime	Area	Height	Area
1	4.797	1050.56055	76.66224	48.3086
2	5.367	1124.12390	74.99065	51.6914

Peak	RetTime	Area	Height	Area
1	4.843	50.10641	3.34079	0.9583
2	5.369	5178.56494	366.41577	99.0417

(*R*)-3-phenyl-2-(5,6,7,8-tetrahydro-9H-pyrido[2,3-b]indol-9-yl)-5,6,7,8-tetrahydroisoquinolin-1(2H)-one (3ra)

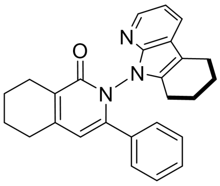

Yield: 56.8 mg (72%). White solid, mp: 122-123 ˚C. **^{1}H NMR** (600 MHz, $CDCl_3$) δ 8.24 – 8.23 (m, 1H), 7.64 (dd, *J* = 7.7, 1.1 Hz, 1H), 7.32 (d, *J* = 7.3 Hz, 2H), 7.19 (t, *J* = 7.4 Hz, 1H), 7.11 (t, *J* = 7.6 Hz, 2H), 7.01 (dd, *J* = 7.7, 4.9 Hz, 1H), 6.06 (s, 1H), 2.62 – 2.54 (m, 6H), 2.44 – 2.39 (m, 1H), 2.25 – 2.20 (m, 1H), 1.88 – 1.74 (m, 6H), 1.73 – 1.68 (m, 1H), 1.66 –

1.60 (m, 1H). ^{13}C NMR (151 MHz, $CDCl_3$) δ 161.2, 148.1, 147.8, 146.6, 142.5, 136.4, 133.7, 128.9, 128.3, 127.8, 127.0, 126.0, 119.3, 116.6, 109.6, 108.7, 36.6, 29.6, 23.7, 22.6, 22.2, 21.9, 21.1, 20.4. **HRMS** (ESI): m/z $[M+H]^+$calcd for $[C_{26}H_{26}N_3O]^+$ required 396.2071, found 396.2078. $[\alpha]_D^{25}$ = +87 (c = 0.1, CH_2Cl_2). The product was analyzed by HPLC to determine the enantiomeric excess: 99% ee (CHIRALPAK AS-H, hexane/*i*-PrOH =85/15, detector: 254 nm, T = 25 °C, flow rate: 1 mL/min), t_1 (minor) = 5.352 min, t_2 (major) = 6.104 min.

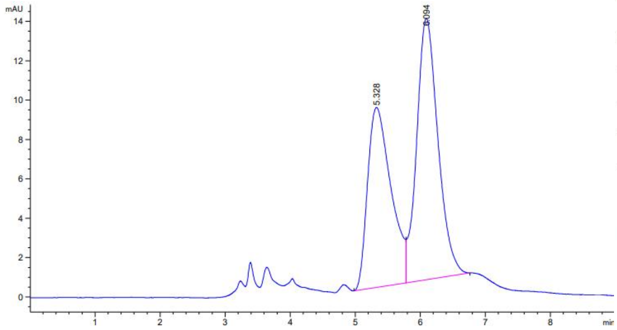

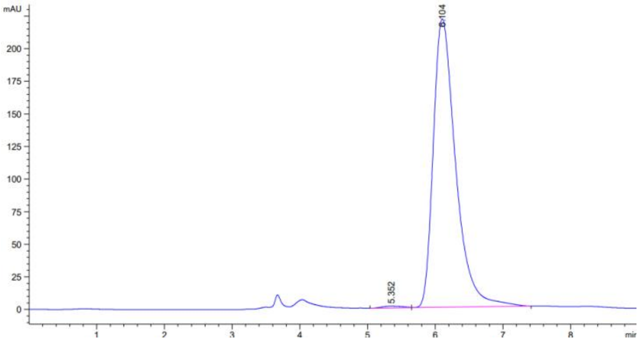

Peak	RetTime	Area	Height	Area
1	5.328	228.25340	9.14020	42.9773
2	6.094	302.84906	13.26953	57.0227

Peak	RetTime	Area	Height	Area
1	5.352	27.77756	1.40769	0.5634
2	6.104	5078.95117	221.12482	99.4366

(*R*)-3-phenyl-2-(6,7,8,9-tetrahydrocyclohepta[4,5]pyrrolo[2,3-b]pyridin-10(5H)-yl)isoquinolin-1(2H)-one (3sa)

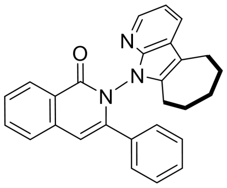

Yield: 68.9 mg (85%). White solid, mp: 126-127 ˚C. **^1H NMR** (600 MHz, $CDCl_3$) δ 8.40 (d, *J* = 8.0 Hz, 1H), 8.22 (dd, *J* = 4.7, 1.1 Hz, 1H), 7.74 – 7.67 (m, 2H), 7.59 (d, *J* = 7.9 Hz, 1H), 7.50 (t, *J* = 7.6 Hz, 1H), 7.33 (d, *J* = 7.2 Hz, 2H), 7.21 (t, *J* = 7.5 Hz, 1H), 7.12 (t, *J* = 7.7 Hz, 2H), 7.03 (dd, *J* = 7.8, 4.8 Hz, 1H), 6.64 (s, 1H), 2.73 – 2.66 (m, 2H), 2.59 – 2.55 (m, 1H), 2.46 – 2.42 (m, 1H), 1.79 – 1.68 (m, 4H), 1.52 – 1.48 (m, 1H), 1.36 – 1.30 (m, 1H). ^{13}C NMR (151 MHz, $CDCl_3$) δ 161.5, 146.8, 145.3, 142.6, 139.1, 136.8, 133.8, 133.3, 128.9, 128.7, 128.6, 127.7, 127.0, 126.4, 125.9, 125.3, 119.6, 116.7, 112.2, 107.6, 31.3, 28.1, 26.5, 25.7, 24.5. **HRMS** (ESI): m/z $[M+H]^+$calcd for $[C_{27}H_{24}N_3O]^+$ required 406.1914, found 406.1920. $[\alpha]_D^{25}$ = -30 (c = 0.1, CH_2Cl_2). The product was analyzed by HPLC to determine the enantiomeric excess: 97% ee (CHIRALPAK AS-H, hexane/*i*-PrOH =85/15, detector: 254 nm, T = 25 °C, flow rate: 1 mL/min), t_1 (minor) = 5.692 min, t_2 (major) = 6.936 min.

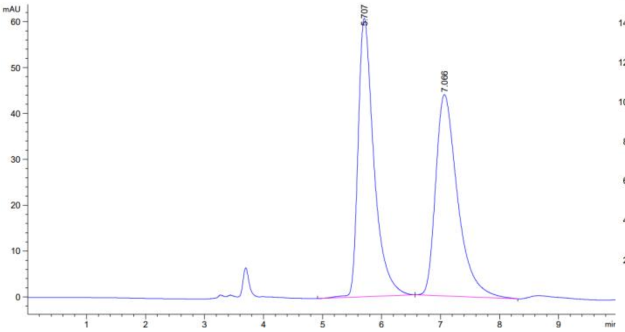

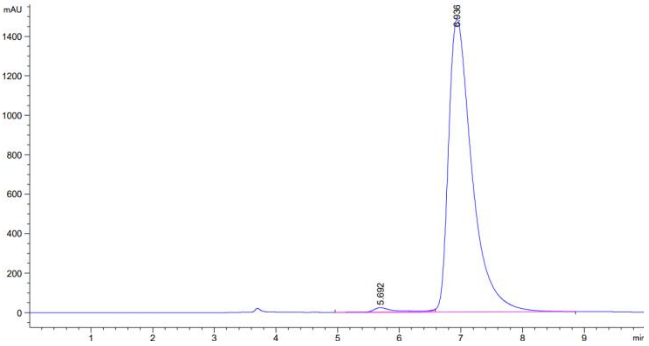

Peak	RetTime	Area	Height	Area
1	5.707	1133.96826	60.74480	50.0148
2	7.066	1133.29932	43.92148	49.9852

Peak	RetTime	Area	Height	Area
1	5.692	613.10510	22.93920	1.5417
2	6.936	3.91559e4	1484.78809	98.4583

(*R*)-2-(2-ethyl-3-methyl-1H-pyrrolo[2,3-b]pyridin-1-yl)-3-phenylisoquinolin-1(2H)-one (3ta)

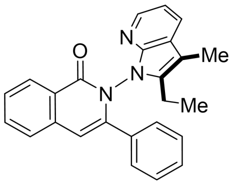

Yield: 59.1 mg (78%). White solid, mp: 153-154 ˚C. **^{1}H NMR** (600 MHz, $CDCl_3$) δ 8.38 (d, *J* = 8.0 Hz, 1H), 8.28 (dd, *J* = 4.7, 1.0 Hz, 1H), 7.74 – 7.70 (m, 2H), 7.60 (d, *J* = 7.9 Hz, 1H), 7.50 (t, *J* = 7.5 Hz, 1H), 7.44 (d, *J* = 7.4 Hz, 2H), 7.23 (t, *J* = 7.4 Hz, 1H), 7.14 (t, *J* = 7.7 Hz, 2H), 7.08 (dd, *J* = 7.7, 4.8 Hz, 1H), 6.66 (s, 1H), 2.46 – 2.33 (m, 2H), 2.14 (s, 3H), 0.93 (t, *J* = 7.6 Hz, 3H). ^{13}C NMR (151 MHz, $CDCl_3$) δ 161.5, 148.3, 145.2, 142.9, 138.9, 136.8, 133.9, 133.3, 129.0, 128.7, 128.7, 127.8, 127.0, 126.4, 126.3, 125.3, 120.6, 116.8, 107.9, 105.9, 17.4, 13.0, 8.6. **HRMS** (ESI): m/z $[M+H]^+$calcd for $[C_{25}H_{22}N_3O]^+$ required 380.1758, found 380.1761. $[\alpha]_D^{25}$ = +62 (c = 0.1, CH_2Cl_2). The product was analyzed by HPLC to determine the enantiomeric excess: 98% ee (CHIRALPAK AS-H, hexane/*i*-PrOH =85/15, detector: 254 nm, T = 25 ºC, flow rate: 1 mL/min), t_1 (minor) = 5.376 min, t_2 (major) = 5.888 min.

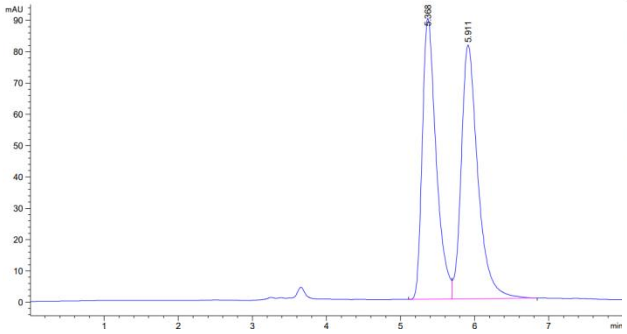

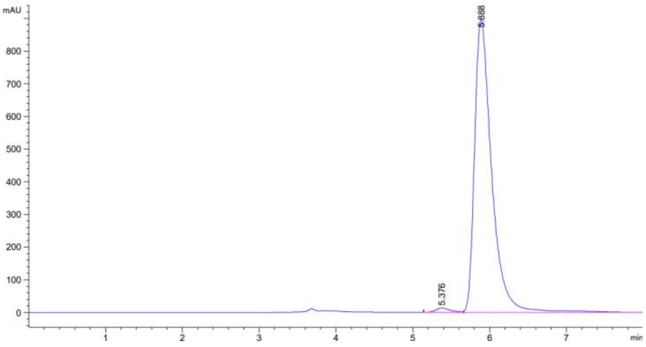

Peak	RetTime	Area	Height	Area
1	5.368	1170.81519	89.68768	48.0912
2	5.911	1263.75671	81.09327	51.9088

Peak	RetTime	Area	Height	Area
1	5.376	162.57121	12.79231	1.1587
2	5.888	1.38674e4	895.73071	98.8413

(*R*)-2-(2-methyl-1H-pyrrolo[2,3-b]pyridin-1-yl)-3-phenylisoquinolin-1(2H)-one (3ua)

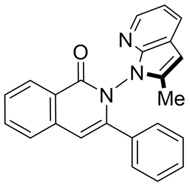

Yield: 56.1 mg (80%). White solid, mp: 160-161 ˚C. **^{1}H NMR** (600 MHz, $CDCl_3$) δ 8.41 (d, *J* = 8.0 Hz, 1H), 8.26 (d, *J* = 4.6 Hz, 1H), 7.74 – 7.72 (m, 2H), 7.61 (d, *J* = 7.9 Hz, 1H), 7.52 (t, *J* = 7.6 Hz, 1H), 7.39 (d, *J* = 7.6 Hz, 2H), 7.22 (t, *J* = 7.4 Hz, 1H), 7.14 (t, *J* = 7.7 Hz, 2H), 7.06 (dd, *J* = 7.7, 4.8 Hz, 1H), 6.66 (s, 1H), 6.17 (s, 1H), 2.13 (s, 3H). ^{13}C NMR (151 MHz, $CDCl_3$) δ 161.4, 148.1, 144.9, 142.9, 137.6, 136.8, 133.7, 133.4, 129.1, 128.7, 128.5, 127.9, 127.2, 126.5, 125.3, 119.4, 117.3, 107.8, 98.6, 12.0. **HRMS** (ESI): m/z $[M+H]^+$calcd for $[C_{23}H_{18}N_3O]^+$ required 352.1445, found 352.1449. $[\alpha]_D^{25}$ = -52 (c = 0.1, CH_2Cl_2). The product was analyzed by HPLC to determine the enantiomeric excess: 97% ee (CHIRALPAK AS-H, hexane/*i*-PrOH =85/15, detector: 254 nm, T = 25 ºC, flow rate: 1 mL/min), t_1 (minor) = 7.241 min, t_2 (major) = 8.472 min.

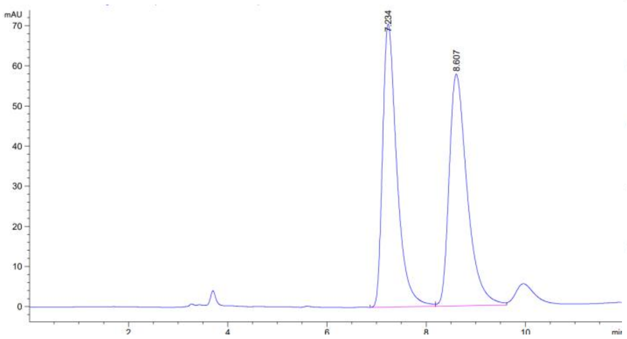

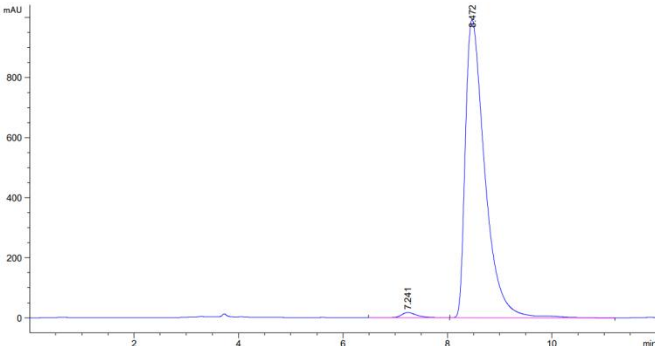

Peak	RetTime	Area	Height	Area

Peak	RetTime	Area	Height	Area

1	7.234	1406.89575	70.46046	49.0588
2	8.607	1460.77135	57.71819	50.9412

1	7.241	349.82452	16.93112	1.3420
2	8.472	2.57182e4	993.23236	98.6580

(*R*)-2-(3-bromo-2-methyl-1H-pyrrolo[2,3-b]pyridin-1-yl)-3-phenylisoquinolin-1(2H)-one (3va)

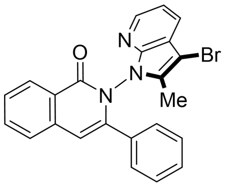

Yield: 69.5 mg (81%). White solid, mp: 177-178 ˚C. **^{1}H NMR** (600 MHz, $CDCl_3$) δ 8.39 (d, *J* = 8.0 Hz, 1H), 8.30 (dd, *J* = 4.8, 1.2 Hz, 1H), 7.76 – 7.72 (m, 2H), 7.61 (d, *J* = 7.9 Hz, 1H), 7.54 – 7.51 (m, 1H), 7.39 – 7.38 (m, 2H), 7.24 (t, *J* = 7.5 Hz, 1H), 7.17 – 7.14 (m, 3H), 6.67 (s, 1H), 2.14 (s, 3H). ^{13}C NMR (151 MHz, $CDCl_3$) δ 161.2, 147.1, 144.6, 144.2, 136.7, 135.7, 133.6, 133.4, 129.3, 128.7, 128.5, 128.0, 127.3, 127.1, 126.5, 125.1, 119.2, 117.9, 108.1, 89.2, 10.7. **HRMS** (ESI): m/z $[M+H]^+$calcd for $[C_{23}H_{17}BrN_3O]^+$ required 430.0550, found 430.0555. $[\alpha]_D^{25}$ = -21 (c = 0.1, CH_2Cl_2). The product was analyzed by HPLC to determine the enantiomeric excess: 97% ee (CHIRALPAK AS-H, hexane/*i*-PrOH =85/15, detector: 254 nm, T = 25 ºC, flow rate: 1 mL/min), t_1 (minor) = 6.581 min, t_2 (major) = 8.595 min.

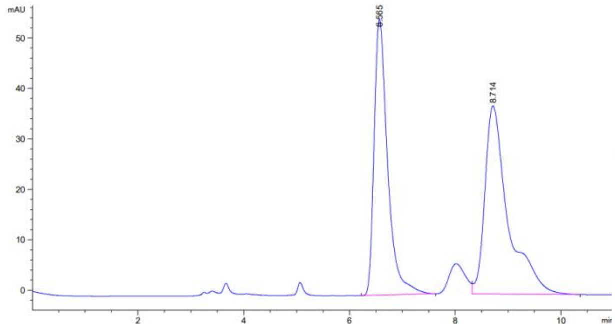

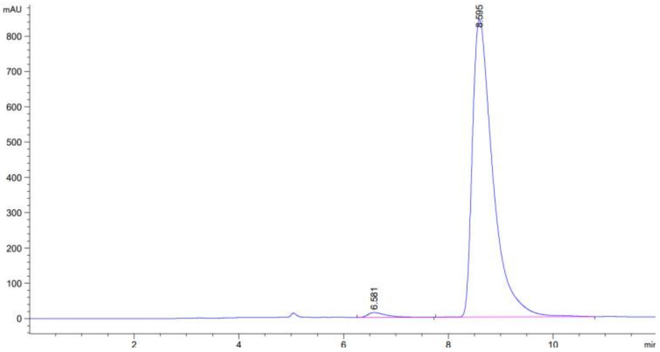

Peak	RetTime	Area	Height	Area
1	6.565	971.17480	54.71373	46.0831
2	8.714	1136.26978	37.30009	53.9169

Peak	RetTime	Area	Height	Area
1	6.581	333.78323	13.80532	1.4375
2	8.595	2.28864e4	842.55981	98.5625

(*R*)-2-(3-chloro-2-methyl-1H-pyrrolo[2,3-b]pyridin-1-yl)-3-phenylisoquinolin-1(2H)-one (3wa)

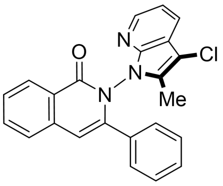

Yield: 57.7 mg (75%). White solid, mp: 157-158 ˚C. **^{1}H NMR** (600 MHz, $CDCl_3$) δ 8.39 (d, *J* = 8.0 Hz, 1H), 8.31 (dd, *J* = 4.8, 1.1 Hz, 1H), 7.80 (dd, *J* = 7.8, 1.2 Hz, 1H), 7.76 – 7.73 (m, 1H), 7.61 (d, *J* = 7.9 Hz, 1H), 7.54 – 7.51 (m, 1H), 7.40 – 7.39 (m, 2H), 7.24 (t, *J* = 7.5 Hz, 1H), 7.17 – 7.14 (m, 3H), 6.66 (s, 1H), 2.13 (s, 3H). **^{13}C NMR** (151 MHz, $CDCl_3$) δ 161.3, 146.6, 144.7, 144.1, 136.7, 133.8, 133.6, 133.5, 129.3, 128.7, 128.5, 128.0, 127.3, 126.5, 126.2, 125.1, 117.8, 117.5, 108.0, 102.7, 9.5. **HRMS** (ESI): m/z $[M+H]^+$calcd for $[C_{23}H_{17}ClN_3O]^+$ required 386.1055, found 386.1059. $[\alpha]_D^{25}$ = -50 (c = 0.1, CH_2Cl_2). The product was analyzed by HPLC to determine the enantiomeric excess: 98% ee (CHIRALPAK AS-H, hexane/*i*-PrOH =85/15, detector: 254 nm, T = 25 ºC, flow rate: 1 mL/min), t_1 (minor) = 6.350 min, t_2 (major) = 8.187 min.

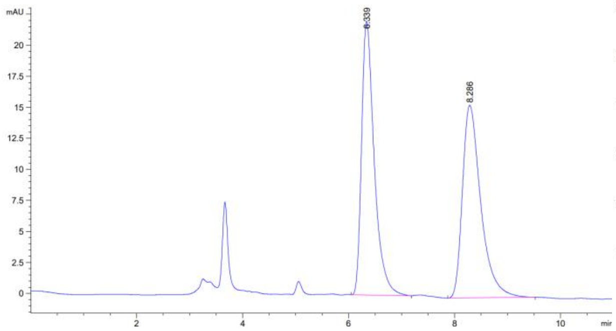

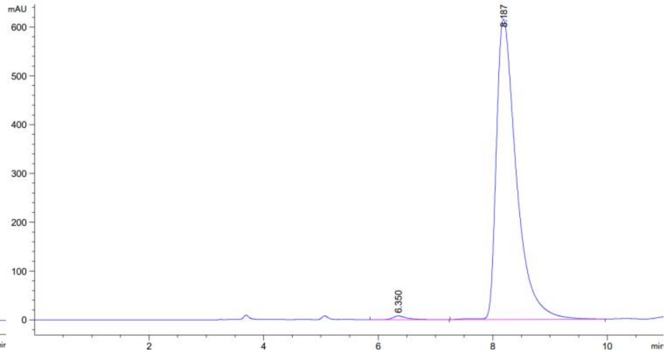

Peak	RetTime	Area	Height	Area
1	6.339	366.62561	22.05909	49.5491
2	8.286	373.29761	15.51106	50.4509

Peak	RetTime	Area	Height	Area
1	6.350	131.34586	7.41284	0.8727
2	8.187	1.49190e4	615.36865	99.1273

(*R*)-2-(3-bromo-2-methyl-1H-pyrrolo[2,3-b]pyridin-1-yl)-3-phenylisoquinolin-1(2H)-one (3xa)

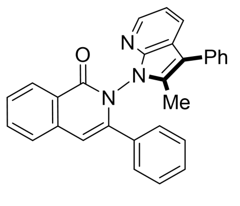

Yield: 59.7 mg (70%). White solid, mp: 187-188 ˚C. **^{1}H NMR** (600 MHz, $CDCl_3$) δ 8.44 (d, *J* = 8.0 Hz, 1H), 8.31 (d, *J* = 4.4 Hz, 1H), 7.87 (d, *J* = 7.7 Hz, 1H), 7.75 (t, *J* = 7.5 Hz, 1H), 7.63 (d, *J* = 7.9 Hz, 1H), 7.54 (t, *J* = 7.6 Hz, 1H), 7.43 – 7.35 (m, 4H), 7.35 (d, *J* = 7.4 Hz, 2H), 7.30 (t, *J* = 7.3 Hz, 1H), 7.22 (t, *J* = 7.4 Hz, 1H), 7.13 (dd, *J* = 14.2, 6.6 Hz, 2H), 7.10 (dd, *J* = 7.7, 4.8 Hz, 1H), 6.68 (d, *J* = 14.1 Hz, 1H), 2.22 (s, 3H). ^{13}C NMR (151 MHz, $CDCl_3$) δ 161.4, 147.5, 144.9, 143.4, 136.8, 133.8, 133.6, 133.5, 129.4, 129.1, 128.7, 128.6, 128.5, 127.9, 127.2, 127.2, 126.5, 125.4, 118.7, 117.5, 112.7, 108.0, 10.4. **HRMS** (ESI): m/z $[M+H]^+$calcd for $[C_{29}H_{22}N_3O]^+$ required 428.1758, found 428.1772. $[\alpha]_D^{25}$ = -90 (c = 0.1, CH_2Cl_2). The product was analyzed by HPLC to determine the enantiomeric excess: 98% ee (CHIRALPAK AS-H, hexane/*i*-PrOH =85/15, detector: 254 nm, T = 25 °C, flow rate: 1 mL/min), t_1 (minor) = 7.366 min, t_2 (major) = 11.327 min.

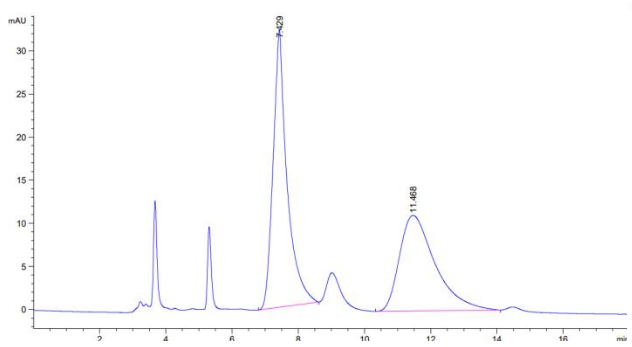

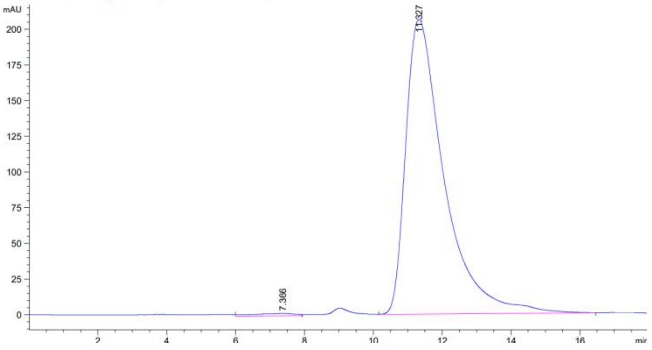

Peak	RetTime	Area	Height	Area
1	7.429	939.52032	32.15431	53.1288
2	11.468	828.86353	110.8840	46.8712

Peak	RetTime	Area	Height	Area
1	7.366	151.81314	1.62914	0.9405
2	11.327	1.59897e4	206.41219	99.0595

(*R*)-2-(5,6,7,8-tetrahydro-9H-pyrido[2,3-b]indol-9-yl)-3-(4-(trifluoromethyl)phenyl)isoquinolin-1(2H)-one (3ab)

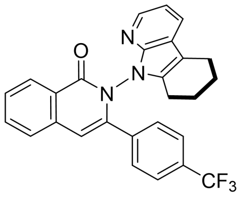

Yield: 82.6 mg (90%). White solid, mp: 172-173 ˚C. **^{1}H NMR** (600 MHz, $CDCl_3$) δ 8.38 (d, *J* = 8.0 Hz, 1H), 8.23 (d, *J* = 4.7 Hz, 1H), 7.75 – 7.69 (m, 2H), 7.61 – 7.58 (m, 3H), 7.53 (t, *J* = 7.6 Hz, 1H), 7.43 (d, *J* = 8.1 Hz, 2H), 7.07 (dd, *J* = 7.7, 4.8 Hz, 1H), 6.63 (s, 1H), 2.65 – 2.61 (m, 1H), 2.57 – 2.52 (m, 1H), 2.43 – 2.39 (m, 1H), 2.26 – 2.21 (m, 1H), 1.86 – 1.81 (m, 2H), 1.75 – 1.72 (m, 1H), 1.70 – 1.64 (m, 1H). ^{13}C NMR (151 MHz, $CDCl_3$) δ 161.2, 148.4, 143.6, 142.7, 137.5, 136.4, 136.3, 133.5, 130.9 ($^2J_{C\text{-}F}$ = 62.0 Hz), 129.0, 128.7, 127.5, 126.5, 126.3, 125.5, 124.9, 124.9, 124.7, 122.9, 119.4, 117.0, 109.4, 108.2, 22.6, 22.2, 21.1, 20.3. **^{19}F NMR** (565 MHz, $CDCl_3$) δ -62.8. **HRMS** (ESI): m/z $[M+H]^+$calcd for $[C_{27}H_{20}F_3N_3O]^+$ required 460.1631, found 460.1645. $[\alpha]_D^{25}$ = +140 (c = 0.1, CH_2Cl_2). The product was analyzed by HPLC to determine the enantiomeric excess: 98% ee (CHIRALPAK AS-H, hexane/*i*-PrOH =85/15, detector: 254 nm, T = 25 °C, flow rate: 1 mL/min), t_1 (minor) = 4.971 min, t_2 (major) = 5.809 min.

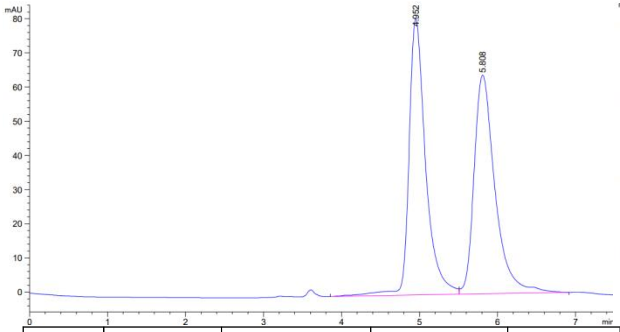

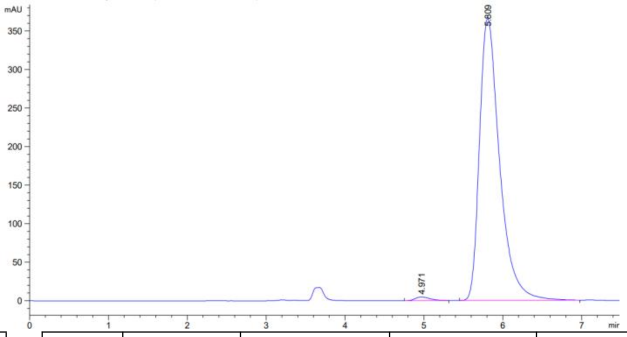

Peak	RetTime	Area	Height	Area
1	4.952	1208.52380	80.89925	50.2749
2	5.808	1195.30798	64.02271	49.7251

Peak	RetTime	Area	Height	Area
1	4.971	58.84403	4.72220	0.9009
2	5.809	6472.81396	365.44846	99.0991

(*R*)-2-(5,6,7,8-tetrahydro-9H-pyrido[2,3-b]indol-9-yl)-3-(4-(trifluoromethyl)phenyl)isoquinolin-1(2H)-one (3ac)

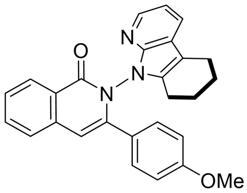

Yield: 74.1 mg (88%). White solid, mp: 143-144 ˚C. **^{1}H NMR** (600 MHz, $CDCl_3$) δ 8.36 (d, *J* = 7.9 Hz, 1H), 8.23 (d, *J* = 3.0 Hz, 1H), 7.66 (t, *J* = 8.3 Hz, 2H), 7.54 (d, *J* = 7.8 Hz, 1H), 7.45 (t, *J* = 7.4 Hz, 1H), 7.32 (d, *J* = 7.4 Hz, 2H), 7.02 (t, *J* = 5.1 Hz, 1H), 6.64 (d, *J* = 7.5 Hz, 2H), 6.58 (s, 1H), 3.67 (s, 3H), 2.63 – 2.60 (m, 1H), 2.53 – 2.51 (m, 1H), 2.39 – 2.36 (m, 1H), 2.26 – 2.23 (m, 1H), 1.81 – 1.80 (m, 2H), 1.71 – 1.68 (m, 1H), 1.67 – 1.65 (m, 1H). ^{13}C NMR (151 MHz, $CDCl_3$) δ 161.5, 160.0, 148.3, 144.9, 142.6, 136.9, 136.5, 133.3, 129.9, 128.6, 126.8, 126.4, 126.1, 125.2, 119.3, 116.7, 113.3, 108.7, 107.5, 55.2, 22.7, 22.2, 21.1, 20.4. **HRMS** (ESI): m/z $[M+H]^{+}$calcd for $[C_{27}H_{24}N_3O]^{+}$ required 422.1863, found 422.1861. $[\alpha]_D^{25}$ = +171 (c = 0.1, CH_2Cl_2). The product was analyzed by HPLC to determine the enantiomeric excess: 98% ee (CHIRALPAK AS-H, hexane/*i*-PrOH =85/15, detector: 254 nm, T = 25 ºC, flow rate: 1 mL/min), t_1 (minor) = 6.953 min, t_2 (major) = 9.388 min.

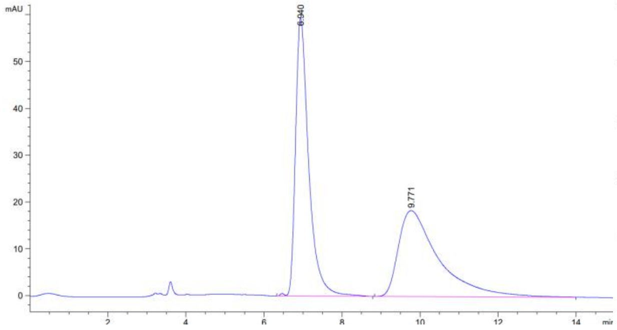

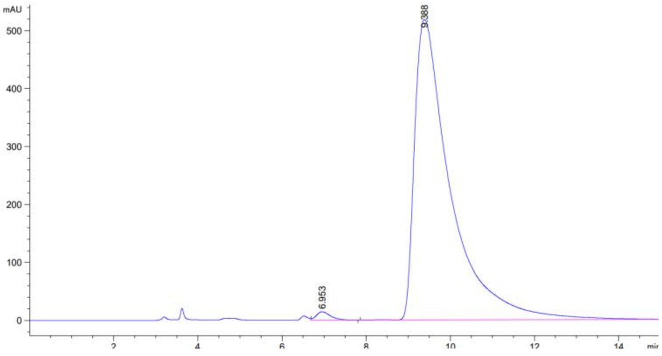

Peak	RetTime	Area	Height	Area
1	6.940	1397.16260	59.31247	51.5482
2	9.771	1313.23901	18.36338	48.4518

Peak	RetTime	Area	Height	Area
1	6.953	340.12744	14.61207	1.0911
2	9.388	3.08340e4	518.26056	98.9089

(*R*)-2-(5,6,7,8-tetrahydro-9H-pyrido[2,3-b]indol-9-yl)-3-(4-(trifluoromethyl)phenyl)isoquinolin-1(2H)-one (3ad)

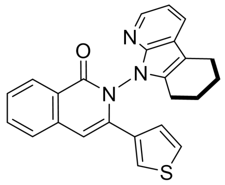

Yield: 75.4 mg (95%). White solid, mp: 165-166 ˚C. **^{1}H NMR** (600 MHz, $CDCl_3$) δ 8.36 (d, *J* = 8.0 Hz, 1H), 8.21 (d, *J* = 4.5 Hz, 1H), 7.72 (d, *J* = 7.6 Hz, 1H), 7.67 (t, *J* = 7.5 Hz, 1H), 7.55 (d, *J* = 7.9 Hz, 1H), 7.46 (t, *J* = 7.5 Hz, 1H), 7.19 (d, *J* = 2.1 Hz, 1H), 7.06 – 7.03 (m, 2H), 6.92 (d, *J* = 4.9 Hz, 1H), 6.73 (s, 1H), 2.68 – 2.63 (m, 1H), 2.61 – 2.56 (m, 1H), 2.43 – 2.38 (m, 1H), 2.26 – 2.22 (m, 1H), 1.85 – 1.81 (m, 2H), 1.73 – 1.68 (m, 2H). ^{13}C NMR (151

MHz, $CDCl_3$) δ 161.5, 147.9, 142.7, 140.0, 136.7, 136.5, 133.9, 133.3, 128.6, 127.5, 127.1, 126.4, 126.3, 125.5, 125.3, 125.2, 119.3, 116.8, 108.8, 107.5, 22.7, 22.3, 21.0, 20.5. **HRMS** (ESI): m/z $[M+H]^+$calcd for $[C_{24}H_{20}N_3OS]^+$ required 398.1322, found 398.1329. $[\alpha]_D^{25}$ = +46 (c = 0.1, CH_2Cl_2). The product was analyzed by HPLC to determine the enantiomeric excess: 98% ee (CHIRALPAK AS-H, hexane/*i*-PrOH =85/15, detector: 254 nm, T = 25 °C, flow rate: 1 mL/min), t_1 (minor) = 6.822 min, t_2 (major) = 8.458 min.

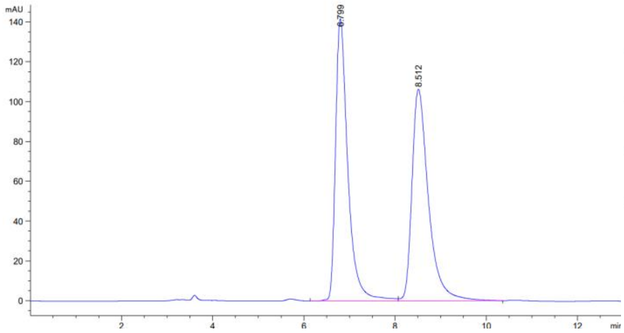

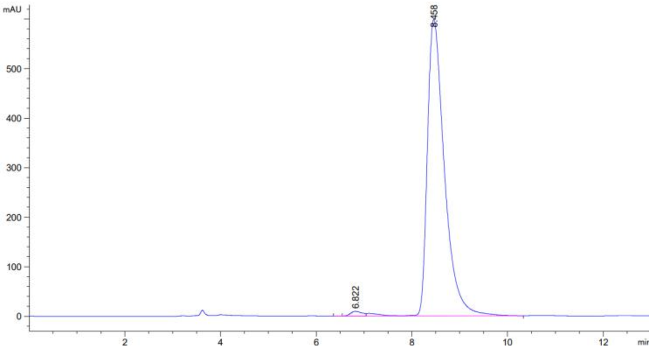

Peak	RetTime	Area	Height	Area
1	6.799	2659.50146	141.67094	49.9471
2	8.512	2665.13037	106.27389	50.0529

Peak	RetTime	Area	Height	Area
1	6.822	157.59790	9.34363	1.0491
2	8.458	1.48656e4	598.35583	98.9509

(*R*)-3-(pyridin-4-yl)-2-(5,6,7,8-tetrahydro-9H-pyrido[2,3-b]indol-9-yl)isoquinolin-1(2H)-one(3ae)

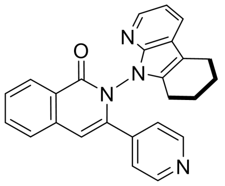

Yield: 35.3 mg (45%). White solid, mp: 155-156 ˚C. **^{1}H NMR** (600 MHz, $CDCl_3$) δ 8.42 (d, *J* = 5.9 Hz, 2H), 8.37 (d, *J* = 8.0 Hz, 1H), 8.22 (dd, *J* = 4.7, 1.1 Hz, 1H), 7.77 – 7.71 (m, 1H), 7.69 (dd, *J* = 7.7, 1.1 Hz, 1H), 7.61 (d, *J* = 7.9 Hz, 1H), 7.55 – 7.52 (m, 1H), 7.35 (dd, *J* = 4.6, 1.5 Hz, 2H), 7.06 (dd, *J* = 7.7, 4.8 Hz, 1H), 6.66 (s, 1H), 2.66 – 2.61 (m, 1H), 2.56 – 2.52 (m, 1H), 2.44 – 2.39 (m, 1H), 2.27 – 2.22 (m, 1H), 1.86 – 1.81 (m, 2H), 1.78 – 1.73 (m, 1H), 1.71 – 1.67 (m, 1H). ^{13}C NMR (151 MHz, $CDCl_3$) δ 161.1, 149.6, 148.2, 142.8, 142.5, 141.6, 136.3, 136.2, 128.7, 127.9, 127.8, 126.7, 126.4, 125.7, 122.9, 119.4, 117.1, 109.5, 108.3, 22.6, 22.2, 21.1, 20.3. **HRMS** (ESI): m/z $[M+H]^+$calcd for $[C_{25}H_{21}N_4O]^+$ required 393.1710, found 393.1717. $[\alpha]_D^{25}$ = -44 (c = 0.1, CH_2Cl_2). The product was analyzed by HPLC to determine the enantiomeric excess: 95% ee (CHIRALPAK AS-H, hexane/*i*-PrOH =75/25, detector: 254 nm, T = 25 °C, flow rate: 1 mL/min), t_1 (minor) = 7.21 min, t_2 (major) = 8.94 min.

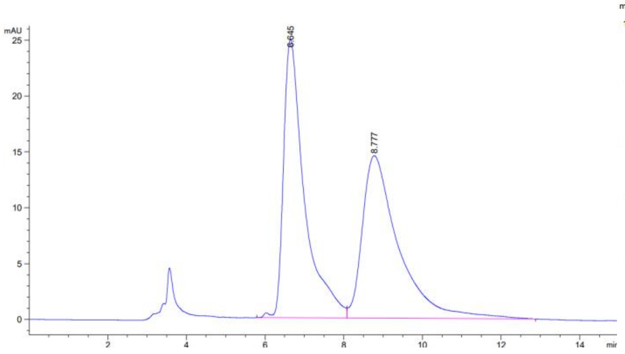

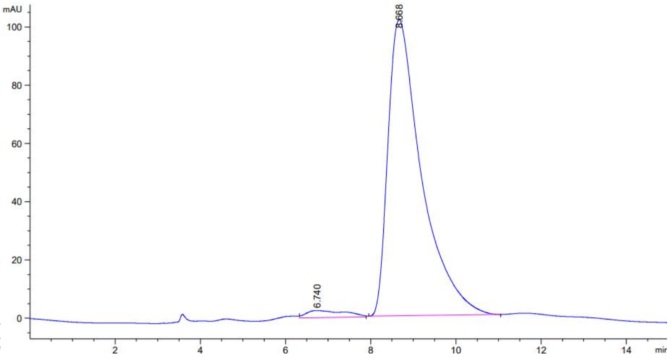

Peak	RetTime	Area	Height	Area
1	6.645	921.88953	24.89529	49.7279
2	8.777	931.97845	14.54086	50.2721

Peak	RetTime	Area	Height	Area
1	6.740	156.72627	2.47741	2.7330
2	8.668	5577.93164	101.72075	97.2670

(*R*)-3-(cyclohex-1-en-1-yl)-2-(5,6,7,8-tetrahydro-9H-pyrido[2,3-b]indol-9-yl)isoquinolin-1(2H)-one (3af)

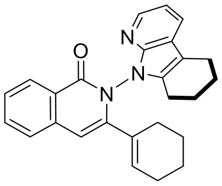

Yield: 65.6 mg (83%). White solid, mp: 140-141 ˚C. **^{1}H NMR** (600 MHz, $CDCl_3$) δ 8.32 (d, *J* = 13.4 Hz, 1H), 8.17 (d, *J* = 10.5 Hz, 1H), 7.77 (d, *J* = 11.8 Hz, 1H), 7.66 (t, *J* = 7.5 Hz, 1H), 7.52 (d, *J* = 7.9 Hz, 1H), 7.43 (t, *J* = 7.6 Hz, 1H), 7.05 (dd, *J* = 7.6, 4.8 Hz, 1H), 6.47 (s, 1H), 5.93 (s, 1H), 2.77 – 2.70 (m, 2H), 2.47 (t, *J* = 5.7 Hz, 2H), 1.94 – 1.78 (m, 8H), 1.35 – 1.21 (m, 4H). ^{13}C NMR (151 MHz, $CDCl_3$) δ 161.6, 147.8, 147.6, 142.6, 137.3, 136.8, 133.0, 132.7, 130.8, 128.5, 126.45, 126.1, 125.9, 119.2, 116.5, 108.5, 105.5, 28.3, 25.3, 22.9, 22.5, 22.4, 21.4, 21.4, 20.6. **HRMS** (ESI): m/z $[M+H]^+$calcd for $[C_{26}H_{26}N_3O]^+$ required 396.2071, found 396.2073. $[\alpha]_D^{25}$ = +145 (c = 0.1, CH_2Cl_2). The product was analyzed by HPLC to determine the enantiomeric excess: 99% ee (CHIRALPAK AS-H, hexane/*i*-PrOH =85/15, detector: 254 nm, T = 25 °C, flow rate: 1 mL/min), t_1 (minor) = 4.652 min, t_2 (major) = 5.127 min.

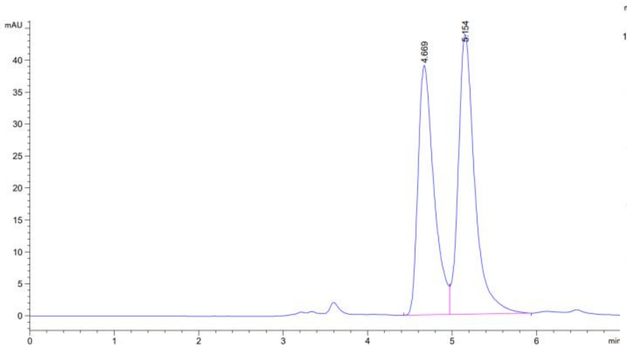

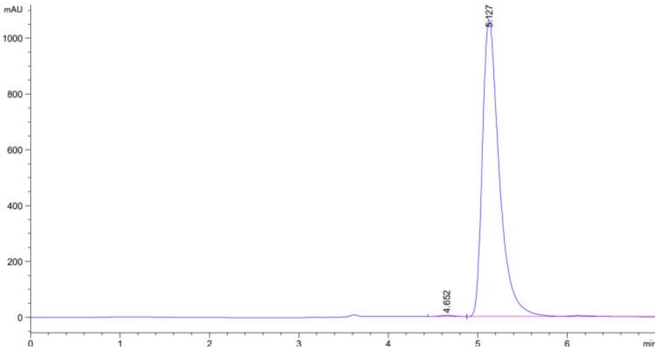

Peak	RetTime	Area	Height	Area
1	4.669	511.40735	39.02634	46.3515
2	5.154	591.91730	43.77682	53.6485

Peak	RetTime	Area	Height	Area
1	4.652	50.89102	4.53086	0.3618
2	5.127	1.40155e4	1064.51941	99.6382

(*R*)-3-cyclopropyl-2-(5,6,7,8-tetrahydro-9H-pyrido[2,3-b]indol-9-yl)isoquinolin-1(2H)-one (3ag)

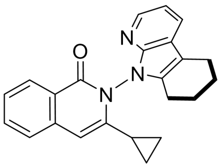

Yield: 49.7 mg (70%). White solid, mp: 165-166 ˚C. **^{1}H NMR** (600 MHz, $CDCl_3$) δ 8.33 (d, *J* = 8.0 Hz, 1H), 8.19 (d, *J* = 3.9 Hz, 1H), 7.79 (dd, *J* = 11.0, 10.3 Hz, 1H), 7.64 (dd, *J* = 11.1, 4.0 Hz, 1H), 7.47 (t, *J* = 11.6 Hz, 1H), 7.42 (t, *J* = 7.4 Hz, 1H), 7.08 (dd, *J* = 7.7, 4.8 Hz, 1H), 6.32 (s, 1H), 2.80 – 2.72 (m, 2H), 2.28 – 2.47 (m, 2H), 1.99 – 1.94 (m, 2H), 1.93 – 1.87 (m, 2H), 1.53 – 1.46 (m, 1H), 0.88 – 0.83 (m, 1H), 0.71 – 0.64 (m, 2H), 0.56 – 0.52 (m, 1H). ^{13}C NMR (151 MHz, $CDCl_3$) δ 161.8, 147.1, 146.5, 142.7, 137.1, 136.7, 133.1, 128.5, 126.4, 126.1, 125.9, 124.9, 119.3, 116.7, 108.5, 102.9, 22.9, 22.4, 21.1, 20.6, 11.9, 7.1, 5.9. **HRMS** (ESI): m/z $[M+H]^+$calcd for $[C_{23}H_{22}N_3O]^+$ required 356.1758, found 356.1761. $[\alpha]_D^{25}$ = -75 (c = 0.1, CH_2Cl_2). The product was analyzed by HPLC to determine the enantiomeric excess: 96% ee (CHIRALPAK AD-H, hexane/*i*-PrOH =90/10, detector: 254 nm, T = 25 °C, flow rate: 1 mL/min), t_1 (minor) = 7.21 min, t_2 (major) = 8.94 min.

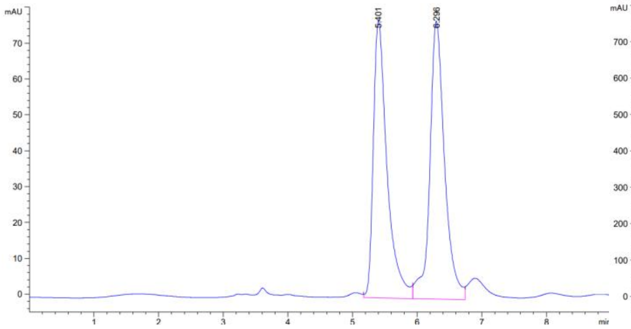

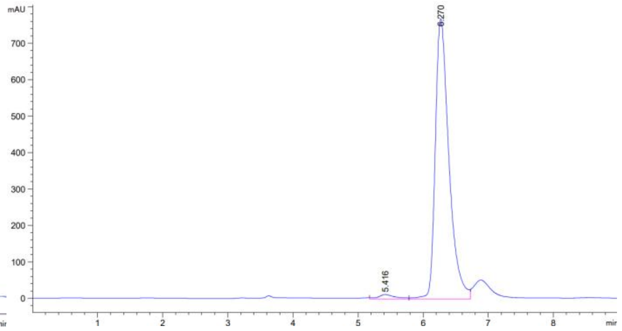

Peak	RetTime	Area	Height	Area

Peak	RetTime	Area	Height	Area

1	5.401	1142.21289	77.31690	48.1297
2	6.296	1230.98279	77.50382	51.8703

1	5.416	232.94180	12.28231	2.0374
2	6.270	1.12005e4	768.93353	97.9626

(*R*)-3-(cyclohex-1-en-1-yl)-2-(5,6,7,8-tetrahydro-9H-pyrido[2,3-b]indol-9-yl)isoquinolin-1(2H)-one (3ah)

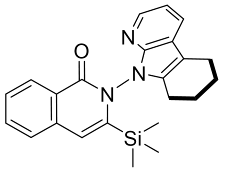

Yield: 60.4 mg (78%). White solid, mp: 167-168 °C. **^{1}H NMR** (600 MHz, $CDCl_3$) δ 8.31 (d, *J* = 8.0 Hz, 1H), 8.18 (d, *J* = 4.6 Hz, 1H), 7.78 (d, *J* = 7.7 Hz, 1H), 7.69 (t, *J* = 7.5 Hz, 1H), 7.58 (d, *J* = 7.9 Hz, 1H), 7.48 (t, *J* = 7.6 Hz, 1H), 7.07 (dd, *J* = 7.6, 4.8 Hz, 1H), 6.81 (s, 1H), 2.79 – 2.71 (m, 2H), 2.49 – 2.42 (m, 2H), 1.95 – 1.85 (m, 4H), 0.05 (s, 9H). ^{13}C NMR (151 MHz, $CDCl_3$) δ 162.1, 149.2, 148.9, 143.1, 138.8, 137.2, 133.4, 128.7, 127.9, 126.9, 126.8, 126.6, 120.2, 117.6, 116.0, 109.8, 23.3, 22.9, 21.9, 21.1, 0.0. **HRMS** (ESI): m/z $[M+H]^+$calcd for $[C_{23}H_{26}N_3OSi]^+$ required 388.1840, found 388.1841. $[\alpha]_D^{25}$ =+62 (c = 0.1, CH_2Cl_2). The product was analyzed by HPLC to determine the enantiomeric excess: 98% ee (CHIRALPAK AS-H, hexane/*i*-PrOH =85/15, detector: 254 nm, T = 25 °C, flow rate: 1 mL/min), t_1 (minor) = 6.551 min, t_2 (major) = 7.025 min.

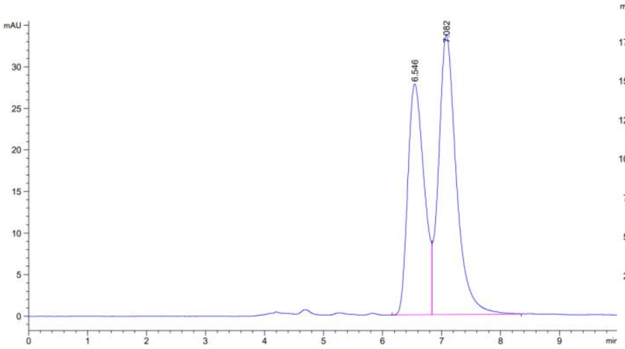

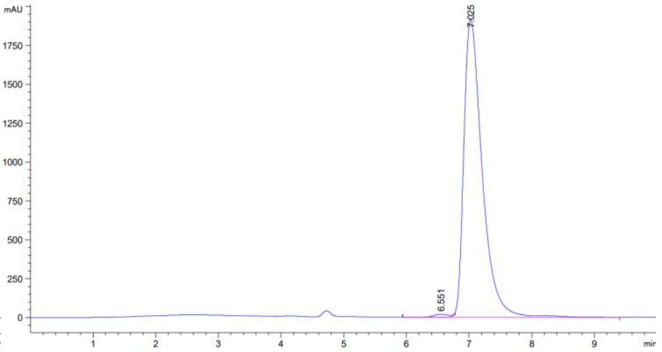

Peak	RetTime	Area	Height	Area
1	6.546	534.48279	27.79096	43.1032
2	7.082	705.52460	33.64652	56.8968

Peak	RetTime	Area	Height	Area
1	6.551	318.34769	19.05519	0.8097
2	7.025	3.89989e4	1914.74121	99.1903

(*S*)-3-(diphenylphosphoryl)-2-(5,6,7,8-tetrahydro-9H-pyrido[2,3-b]indol-9-yl)isoquinolin-1(2H)-one (3ai)

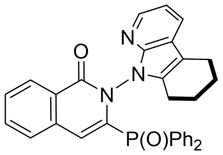

Yield: 69.0 mg (67%). White solid, mp: 190-191 °C. **^{1}H NMR** (600 MHz, $CDCl_3$) δ 8.31 (d, *J* = 7.9 Hz, 1H), 7.94 (dt, *J* = 21.1, 10.5 Hz, 2H), 7.68 (dt, *J* = 15.0, 8.9 Hz, 2H), 7.61 – 7.54 (m, 2H), 7.54 – 7.46 (m, 5H), 7.42 (dd, *J* = 7.6, 1.0 Hz, 1H), 7.20 – 7.10 (m, 1H), 7.06 (td, *J* = 7.7, 3.2 Hz, 2H), 6.85 – 6.75 (m, 2H), 3.09 – 3.05 (m, 1H), 2.60 – 2.59 (m, 2H), 2.34 – 2.31 (m, 1H), 1.94 – 1.90 (m, 1H), 1.89 – 1.83 (m, 3H). ^{13}C NMR (151 MHz, $CDCl_3$) δ 161.2, 161.1, 148.0, 141.4, 139.9 ($^1J_{C-P}$ = 106.4 Hz), 139.5, 134.9 ($^2J_{C-P}$ = 13.2 Hz), 133.4, 132.5, 132.5, 131.4 ($^3J_{C-F}$ = 2.3 Hz), 131.1, 131.0, 130.3, 129.3, 128.6, 128.6, 128.6, 127.8, 127.6, 127.5, 127.1, 125.1, 119.6, 117.7, 117.6, 116.5, 109.2, 22.7, 22.4, 21.1, 20.5. **^{31}P NMR** (243 MHz, $CDCl_3$) δ 20.9. **HRMS** (ESI): m/z $[M+H]^+$calcd for $[C_{32}H_{27}N_3O_2P]^+$ required 516.1836, found 516.1840. $[\alpha]_D^{25}$ = -154 (c = 0.1, CH_2Cl_2). The product was analyzed by HPLC to determine the enantiomeric excess: 99% ee (CHIRALPAK AS-H, hexane/*i*-PrOH =95/5, detector: 254 nm, T = 25 °C, flow rate: 0.8 mL/min), t_1 (minor) = 19.417 min, t_2 (major) = 24.173 min.

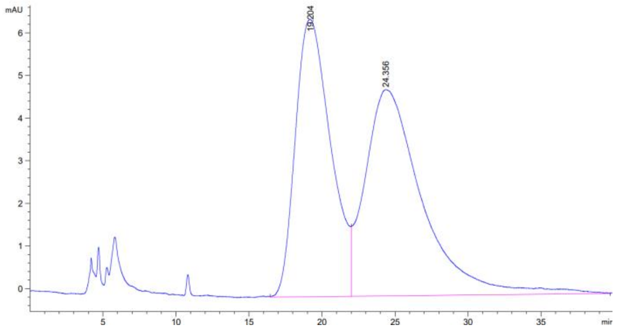

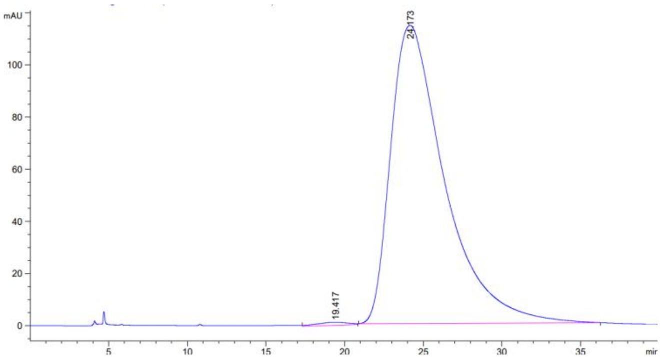

Peak	RetTime	Area	Height	Area
1	19.204	1094.51074	6.51018	45.3983
2	24.356	1316.39709	4.83772	54.6017

Peak	RetTime	Area	Height	Area
1	19.417	164.49036	1.09958	0.5999
2	24.173	2.72548e4	114.31062	99.4001

(*R*)-3-(pyridin-4-yl)-2-(5,6,7,8-tetrahydro-9H-pyrido[2,3-b]indol-9-yl)isoquinolin-1(2H)-one(3aj)

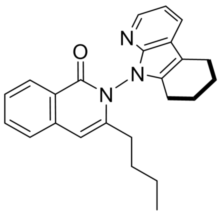

Yield: 59.3 mg (80%). White solid, mp: 141-142 ˚C. **^{1}H NMR** (600 MHz, $CDCl_3$) δ 8.30 (t, *J* = 8.4 Hz, 1H), 8.19 – 8.18 (m, 1H), 7.80 (dd, *J* = 7.7, 1.0 Hz, 1H), 7.67 – 7.65 (m, 1H), 7.50 (t, *J* = 14.0 Hz, 1H), 7.42 (t, *J* = 7.3 Hz, 1H), 7.08 (dd, *J* = 7.7, 4.8 Hz, 1H), 6.47 (d, *J* = 14.6 Hz, 1H), 2.80 – 2.68 (m, 2H), 2.53 – 2.49 (m, 1H), 2.46 – 2.35 (m, 3H), 1.98 – 1.93 (m, 2H), 1.92 – 1.87 (m, 2H), 1.63 – 1.54 (m, 2H), 1.31 – 1.24 (m, 3H), 0.81 (t, *J* = 7.3 Hz, 3H). ^{13}C NMR (151 MHz, $CDCl_3$) δ 161.8, 147.4, 145.2, 142.8, 137.1, 136.8, 133.1, 128.4, 126.2, 126.2, 125.8, 124.8, 119.4, 116.9, 108.9, 104.5, 30.9, 29.2, 22.8, 22.4, 22.2, 21.1, 20.6, 13.8. **HRMS** (ESI): m/z $[M+H]^+$calcd for $[C_{24}H_{26}N_3O]^+$ required 372.2071, found 372.2076. $[\alpha]_D^{25}$ = -44 (c = 0.1, CH_2Cl_2). The product was analyzed by HPLC to determine the enantiomeric excess: 98% ee (CHIRALPAK AS-H, hexane/*i*-PrOH =85/15, detector: 254 nm, T = 25 ºC, flow rate: 1 mL/min), t_1 (minor) = 4.674 min, t_2 (major) = 5.289 min.

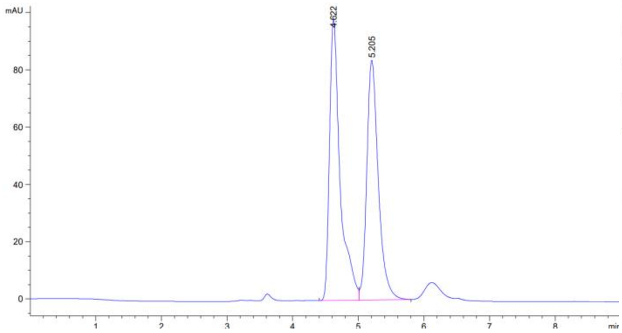

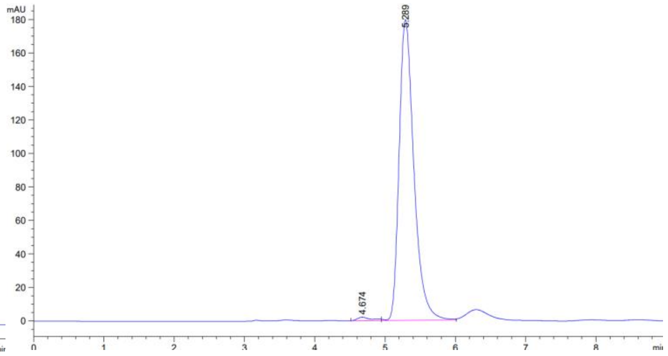

Peak	RetTime	Area	Height	Area
1	4.622	1108.44983	97.89529	52.0330
2	5.205	1021.83063	83.67129	47.9670

Peak	RetTime	Area	Height	Area
1	4.674	30.97710	2.15031	1.1696
2	5.289	2617.50513	179.60805	98.8304

(*R*)-3-(3-hydroxypropyl)-2-(5,6,7,8-tetrahydro-9H-pyrido[2,3-b]indol-9-yl)isoquinolin-1(2H)-one (3ak)

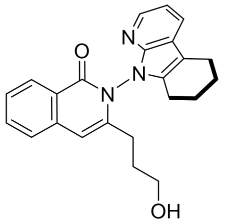

Yield: 52.2 mg (70%). White solid, mp: 122-123 ˚C. **^{1}H NMR** (600 MHz, $CDCl_3$) δ 8.27 (d, *J* = 8.0 Hz, 1H), 8.12 (d, *J* = 4.6 Hz, 1H), 7.78 (d, *J* = 7.7 Hz, 1H), 7.62 (t, *J* = 7.5 Hz, 1H), 7.49 (d, *J* = 7.9 Hz, 1H), 7.39 (dd, *J* = 16.9, 9.4 Hz, 1H), 7.06 (dd, *J* = 7.7, 4.8 Hz, 1H), 6.44 (s, 1H), 3.48 (t, *J* = 5.9 Hz, 2H), 2.83 (s, 1H), 2.77 – 2.68 (m, 2H), 2.51 (t, *J* = 7.4 Hz, 2H), 2.45 – 2.36 (m, 2H), 1.94 – 1.84 (m, 4H), 1.77 – 1.73 (m, 2H). ^{13}C NMR (151 MHz, $CDCl_3$) δ 161.7, 147.6, 144.6, 142.6, 137.0, 136.9, 133.2, 128.3, 126.5, 125.9, 126.7, 119.7, 117.1, 109.3, 104.7, 61.2, 30.1, 27.7, 22.8, 22.3, 21.0, 20.6. **HRMS** (ESI): m/z $[M+H]^+$calcd for $[C_{23}H_{24}N_3O_2]^+$ required

374.1863, found 374.1867. $[\alpha]_D^{25}$ = -158 (c = 0.1, CH_2Cl_2). The product was analyzed by HPLC to determine the enantiomeric excess: 99% ee (CHIRALPAK AS-H, hexane/*i*-PrOH =90/10, detector: 254 nm, T = 25 ºC, flow rate: 1 mL/min), t_1 (minor) = 7.942 min, t_2 (major) = 9.903 min.

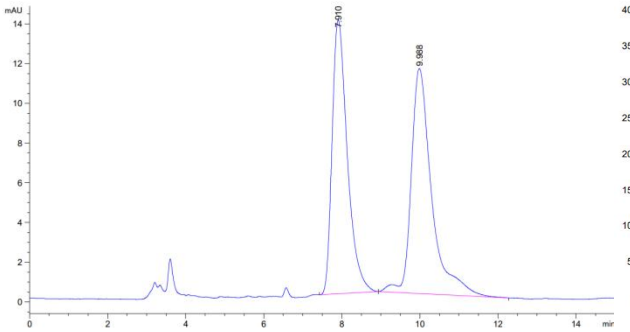

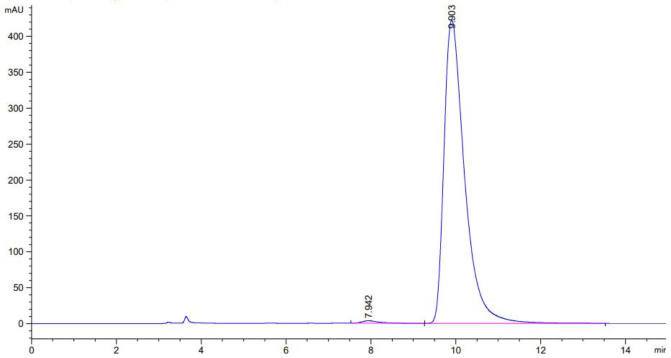

Peak	RetTime	Area	Height	Area
1	7.910	373.01480	13.78905	47.5397
2	9.988	411.62381	11.32807	52.4603

Peak	RetTime	Area	Height	Area
1	7.942	100.61913	3.41924	0.7014
2	9.903	1.42447e4	422.21384	99.2986

(*R*)-3-(hex-5-yn-1-yl)-2-(5,6,7,8-tetrahydro-9H-pyrido[2,3-b]indol-9-yl)isoquinolin-1(2H)-one (3al)

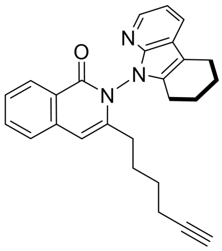

Yield: 48.2 mg (61%). White solid, mp: 135-136 ˚C. **^{1}H NMR** (600 MHz, $CDCl_3$) δ 8.30 (d, *J* = 8.0 Hz, 1H), 8.18 (d, *J* = 4.7 Hz, 1H), 7.81 (d, *J* = 7.7 Hz, 1H), 7.66 (t, *J* = 7.5 Hz, 1H), 7.52 (d, *J* = 7.9 Hz, 1H), 7.43 (t, *J* = 7.5 Hz, 1H), 7.09 (dd, *J* = 7.5, 4.6 Hz, 1H), 6.48 (s, 1H), 2.80 – 2.71 (m, 2H), 2.53 – 2.39 (m, 4H), 2.13 – 2.11 (m, 2H), 1.97 – 1.89 (m, 5H), 1.78 – 1.69 (m, 2H), 1.53 – 1.47 (m, 2H). ^{13}C NMR (151 MHz, $CDCl_3$) δ 161.7, 147.6, 144.6, 142.8, 137.0, 136.8, 133.2, 128.4, 126.4, 125.8, 124.8, 119.5, 117.0, 109.0, 104.6, 83.9, 68.7, 30.7, 27.8, 26.1, 22.8, 22.4, 21.1, 20.6, 18.2. **HRMS** (ESI): m/z $[M+H]^+$calcd for $[C_{26}H_{26}N_3O]^+$ required 396.2071, found 396.2075. $[\alpha]_D^{25}$ = -121 (c = 0.1, CH_2Cl_2). The product was analyzed by HPLC to determine the enantiomeric excess: 98% ee (CHIRALPAK AS-H, hexane/*i*-PrOH =85/15, detector: 254 nm, T = 25 ºC, flow rate: 1 mL/min), t_1 (minor) = 5.986 min, t_2 (major) = 7.456 min.

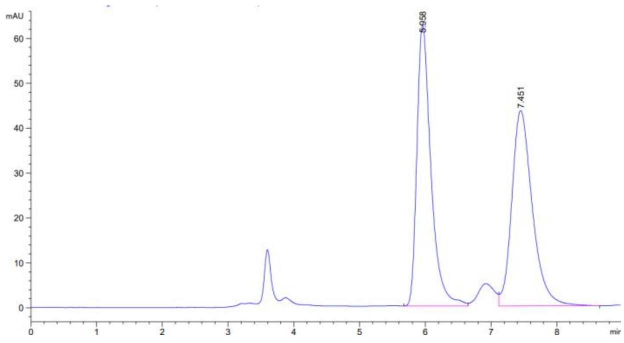

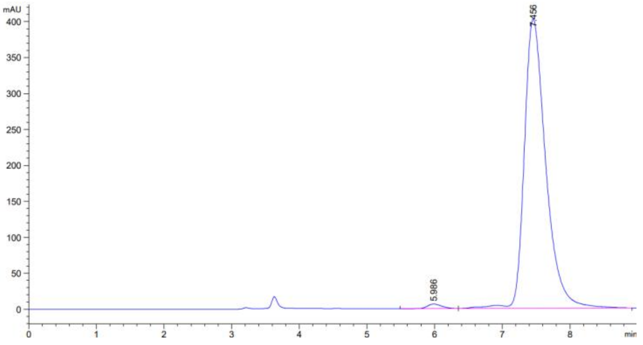

Peak	RetTime	Area	Height	Area
1	5.958	929.19775	62.54620	49.5992
2	7.451	944.21570	43.50178	50.4008

Peak	RetTime	Area	Height	Area
1	5.986	95.92433	6.43610	1.0687
2	7.456	8879.74023	402.55170	98.9313

Ethyl (*R*)-1-oxo-2-(5,6,7,8-tetrahydro-9H-pyrido[2,3-b]indol-9-yl)-1,2-dihydroisoquinoline-3-carboxylate (3am)

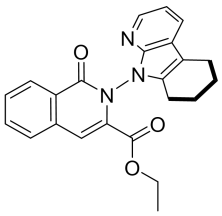

Yield: 73.5 mg (95%). White solid, mp: 172-173 ˚C. **^{1}H NMR** (600 MHz, $CDCl_3$) δ 8.41 (d, *J* = 7.9 Hz, 1H), 8.14 (d, *J* = 4.7 Hz, 1H), 7.76 (t, *J* = 7.4 Hz, 2H), 7.69 (d, *J* = 7.8 Hz, 1H), 7.61 (t, *J* = 7.6 Hz, 1H), 7.36 (s, 1H), 7.03 (dd, *J* = 7.7, 4.8 Hz, 1H), 4.02 (q, *J* = 7.0 Hz, 2H), 2.78 – 2.71 (m, 2H), 2.66 – 2.56 (m, 2H), 1.97 – 1.90 (m, 4H), 0.97 (t, *J* = 7.1 Hz, 3H). ^{13}C NMR (151 MHz, $CDCl_3$) δ 161.3, 160.7, 147.1, 142.4, 137.6, 134.9, 134.1, 133.6, 129.3, 128.8, 127.8, 127.3, 126.0, 119.3, 116.6, 112.7, 107.9, 62.0, 22.9, 22.4, 21.0, 20.6, 13.6. **HRMS** (ESI): m/z $[M+H]^+$calcd for $[C_{23}H_{22}N_3O_3]^+$ required 388.1656, found 388.1655. $[\alpha]_D^{25}$ = -109 (c = 0.1, CH_2Cl_2). The product was analyzed by HPLC to determine the enantiomeric excess: 98% ee (CHIRALPAK AS-H, hexane/*i*-PrOH =85/15, detector: 254 nm, T = 25 °C, flow rate: 1 mL/min), t_1 (minor) = 6.887 min, t_2 (major) = 7.310 min.

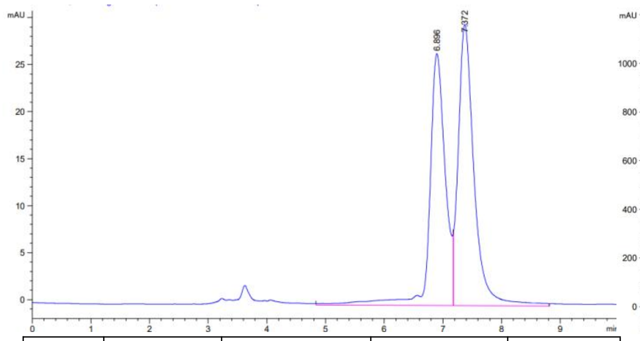

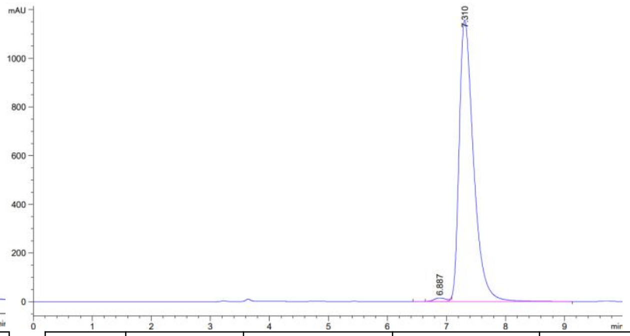

Peak	RetTime	Area	Height	Area
1	6.896	501.85797	26.80707	47.2699
2	7.372	559.82837	29.9061	52.7301

Peak	RetTime	Area	Height	Area
1	6.887	205.62155	14.86232	1.0391
2	7.310	1.95828e4	1156.98254	98.9609

(8R,9S,13S,14S)-13-methyl-17-oxo-7,8,9,11,12,13,14,15,16,17-decahydro-6H-cyclopenta[a]phenanthren-3-yl 5-(1-oxo-2-((R)-5,6,7,8-tetrahydro-9H-pyrido[2,3-b]indol-9-yl)-1,2-dihydroisoquinolin-3-yl)pentanoate (3an)

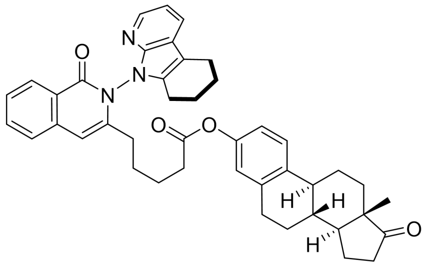

Yield: 108.0 mg (81%). White solid, mp: 158-159 ˚C. **^{1}H NMR** (600 MHz, $CDCl_3$) δ 8.30 (d, *J* = 7.9 Hz, 1H), 8.18 (d, *J* = 4.3 Hz, 1H), 7.80 (d, *J* = 7.6 Hz, 1H), 7.65 (t, *J* = 7.4 Hz, 1H), 7.51 (d, *J* = 7.9 Hz, 1H), 7.42 (t, *J* = 7.5 Hz, 1H), 7.26 (d, *J* = 8.2 Hz, 1H), 7.08 (dd, *J* = 7.7, 4.8 Hz, 1H), 6.79 (dd, *J* = 9.1, 7.3 Hz, 1H), 6.77 (s, 1H), 6.49 (s, 1H). 2.89 – 2.87 (m, 2H), 2.79 – 2.72 (m, 2H), 2.51 – 2.37 (m, 7H), 2.28 – 2.25 (m, 1H), 2.16 – 2.10 (m, 1H), 2.06 – 1.89 (m, 8H), 1.74 – 1.69 (m, 4H), 1.64 – 1.56 (m, 2H), 1.54 – 1.47 (m, 4H), 0.89 (s, 3H). ^{13}C NMR (151 MHz, $CDCl_3$) δ 172.0, 161.7, 148.5, 147.5, 144.4, 142.9, 138.0, 137.4, 137.0, 136.8, 133.2, 128.4 126.4, 126.4 , 126.3, 125.9, 124.8, 121.5, 119.5, 118.7, 117.0, 109.1, 104.6, 50.4, 47.9, 44.2, 38.0, 35.9, 34.0, 31.6, 30.9, 29.4, 26.5, 26.3, 25.8, 24.4, 22.8, 22.4, 21.6, 21.1, 20.6, 13.9. **HRMS** (ESI): m/z $[M+H]^+$calcd for $[C_{43}H_{46}N_3O_4]^+$ required 668.3483, found 668.3478. $[\alpha]_D^{25}$ = -64 (c = 0.1, CH_2Cl_2). The product was analyzed by HPLC to determine the enantiomeric excess: 97% de (CHIRALPAK AD-H, hexane/*i*-PrOH =80/20, detector: 254 nm, T = 25 °C, flow rate: 1 mL/min), t_1 (major) = 26.485 min, t_2 (minor) = 31.726 min.

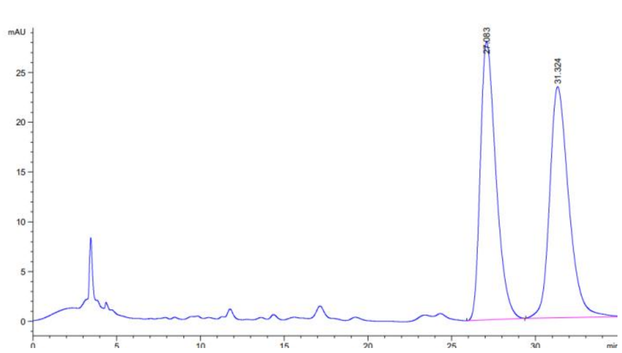

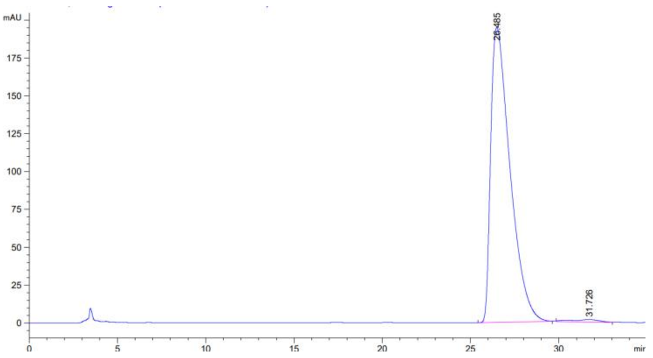

Peak	RetTime	Area	Height	Area
1	27.083	1812.87378	37.95967	49.7129
2	31.324	1833.81653	23.21653	50.2871

Peak	RetTime	Area	Height	Area
1	26.485	1.47960e4	194.73929	98.7025
2	31.726	194.50836	1.69779	1.2975

(3S,8R,9S,10R,13S,14S)-10,13-dimethyl-17-oxo-2,3,4,7,8,9,10,11,12,13,14,15,16,17-tetradecahydro-1H-cyclopenta[a]phenanthren-3-yl-5-(1-oxo-2-((R)-5,6,7,8-tetrahydro-9H-pyrido[2,3-b]indol-9-yl)-1,2-dihydroisoquinolin-3-yl)pentanoate (3ao)

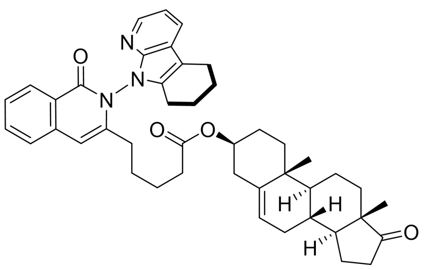

Yield: 117.5 mg (86%). White solid, mp: 177-178 ˚C. **^{1}H NMR** (600 MHz, $CDCl_3$) δ 8.28 (d, *J* = 7.9 Hz, 1H), 8.16 – 8.15 (m, 1H), 7.79 (dd, *J* = 7.7, 1.1 Hz, 1H), 7.65 – 7.62 (m, 1H), 7.50 (d, *J* = 7.9 Hz, 1H), 7.41 (t, *J* = 7.6 Hz, 1H), 7.07 (dd, *J* = 7.7, 4.8 Hz, 1H), 6.45 (s, 1H), 5.38 (d, *J* = 4.9 Hz, 1H), 4.59 – 4.54 (m, 1H), 2.78 – 2.71 (m, 2H), 2.50 – 2.37 (m, 5H), 2.32 – 2.26 (m, 2H), 2.21 – 2.18 (m, 2H), 2.12 – 2.04 (m, 2H), 1.95 – 1.82 (m, 8H), 1.66 – 1.43 (m, 10H), 1.29 – 1.25 (m, 2H), 1.15 – 1.10 (m, 1H), 1.02 – 0.99 (m, 4H), 0.87 (S, 3H), . ^{13}C NMR (151 MHz, $CDCl_3$) δ 172.6, 161.7, 147.5, 144.5, 142.8, 139.9, 137.0, 136.7, 133.1, 128.4, 126.4, 126.3, 125.8, 124.8, 121.9, 119.4, 117.0, 109.0, 104.5, 73.7, 51.7, 50.1, 47.5, 38.1, 36.9, 36.7, 35.8, 34.2, 31.5, 31.4, 30.9, 30.8, 27.7, 26.5, 24.4, 22.8, 22.4, 21.9, 21.1, 20.6, 20.3, 19.3, 13.6. **HRMS** (ESI): m/z $[M+Na]^+$calcd for $[C_{44}H_{51}N_3O_4]^+$ required 686.3952, found 686.3967. $[\alpha]_D^{25}$ = -131 (c = 0.1, CH_2Cl_2). The product was analyzed by HPLC to determine the enantiomeric excess: 98% de (CHIRALPAK AD-H, hexane/*i*-PrOH =80/20, detector: 254 nm, T = 25 °C, flow rate: 1 mL/min), t_1 (minor) = 12.567 min, t_2 (major) = 13.965 min.

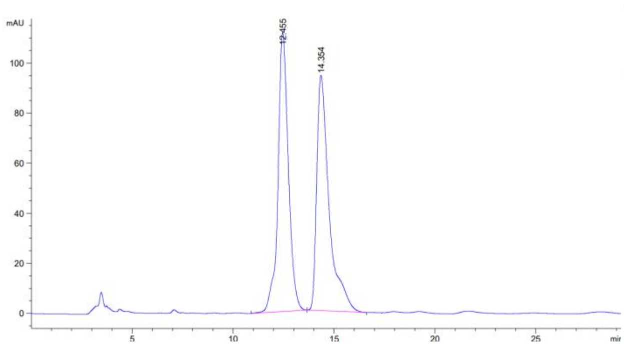

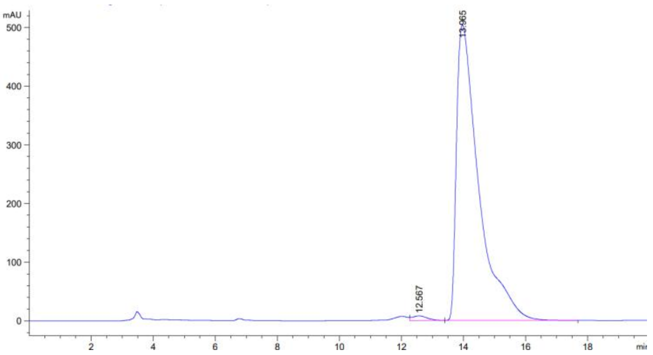

Peak	RetTime	Area	Height	Area
1	12.455	3994.55566	111.53780	49.9262
2	14.354	4006.36328	94.02797	50.0738

Peak	RetTime	Area	Height	Area
1	12.567	262.66266	7.87349	1.0410
2	13.965	249691e4	503.52420	98.9590

(*R*)-3,4-diethyl-2-(5,6,7,8-tetrahydro-9H-pyrido[2,3-b]indol-9-yl)isoquinolin-1(2H)-one (3ap)

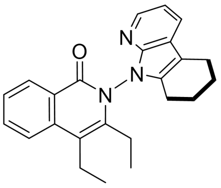

Yield: 60.1 mg (81%). White solid, mp: 145-146 ˚C. **^{1}H NMR** (600 MHz, $CDCl_3$) δ 8.38 (d, *J* = 7.9 Hz, 1H), 8.20 (d, *J* = 4.6 Hz, 1H), 7.81 (d, *J* = 7.7 Hz, 1H), 7.76 – 7.70 (m, 2H), 7.44 (t, *J* = 7.4 Hz, 1H), 7.09 (dd, *J* = 7.5, 4.9 Hz, 1H), 2.86 (q, *J* = 7.4 Hz, 2H), 2.80 – 2.72 (m, 2H), 2.65 – 2.56 (m, 2H), 2.48 – 2.41 (m, 2H), 1.97 – 1.87 (m, 2H), 1.68 – 1.59 (m, 2H), 1.32 (t, *J* = 7.5 Hz, 3H), 1.07 (t, *J* = 7.5 Hz, 3H). ^{13}C NMR (151 MHz, $CDCl_3$) δ 161.2, 147.8, 142.7, 142.6, 137.3, 137.0, 133.0, 128.9, 126.3, 126.1, 125.5, 123.0, 119.5, 116.9, 115.4, 109.0, 38.6, 36.5, 27.9, 22.8, 22.4, 22.3, 21.3, 20.9, 20.6, 14.7, 13.8. **HRMS** (ESI): m/z $[M+H]^+$calcd for $[C_{24}H_{26}N_3O]^+$ required 372.2071, found 372.2078. $[\alpha]_D^{25}$ = -134 (c = 0.1, CH_2Cl_2). The product was analyzed by HPLC to determine the enantiomeric excess: 98% ee (CHIRALPAK AS-H, hexane/*i*-PrOH =95/5, detector: 254 nm, T = 25 ºC, flow rate: 0.8 mL/min), t_1 (minor) = 7.757 min, t_2 (major) = 9.929 min.

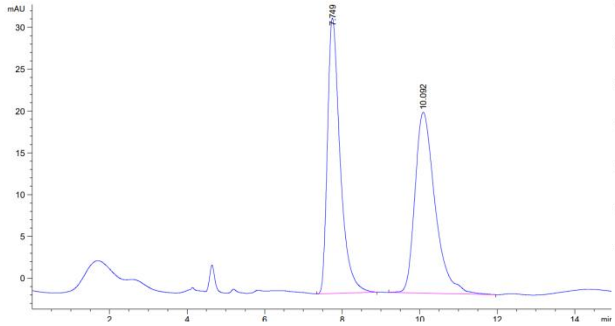

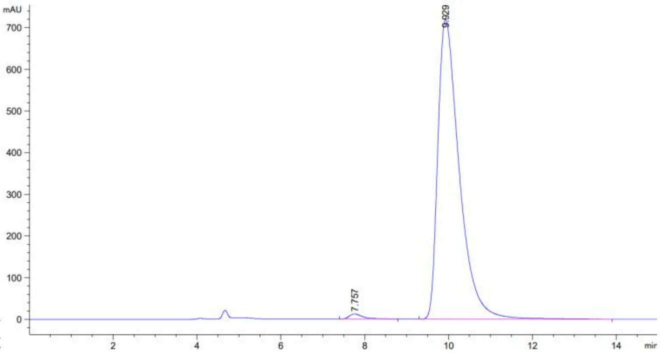

Peak	RetTime	Area	Height	Area
1	7.749	747.81598	32.92284	49.0422
2	10.092	777.02734	21.62253	50.9578

Peak	RetTime	Area	Height	Area
1	7.757	263.70605	11.77184	1.0239
2	9.929	2.54906e4	718.31799	98.9761

(*R*)-3,4-dipropyl-2-(5,6,7,8-tetrahydro-9H-pyrido[2,3-b]indol-9-yl)isoquinolin-1(2H)-one (3aq)

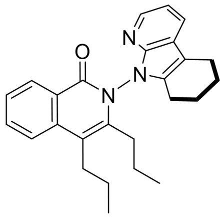

Yield: 59.8 mg (75%). White solid, mp: 147-148 ˚C. **^{1}H NMR** (600 MHz, $CDCl_3$) δ 8.37 (d, *J* = 7.9 Hz, 1H), 8.18 (dd, *J* = 4.7, 1.1 Hz, 1H), 7.80 (dd, *J* = 7.7, 1.2 Hz, 1H), 7.71 (d, *J* = 3.6 Hz, 2H), 7.42 (dd, *J* = 8.0, 4.1 Hz, 1H), 7.08 (dd, *J* = 7.7, 4.8 Hz, 1H), 2.81 – 2.73 (m, 4H), 2.60 – 2.54 (m, 1H), 2.47 – 2.42 (m, 3H), 1.97 – 1.87 (m, 4H), 1.77 – 1.65 (m, 2H), 1.57 – 1.45 (m, 2H), 1.11 (t, *J* = 7.3 Hz, 3H), 0.74 (t, *J* = 7.4 Hz, 3H). ^{13}C NMR (151 MHz, $CDCl_3$) δ 161.2, 147.9, 142.7, 142.0, 137.3, 137.2, 132.9, 128.9, 126.1, 126.1, 125.4, 123.2, 119.4, 116.8, 114.3, 109.0, 31.2, 30.1, 23.5, 22.8, 22.6, 22.4, 21.3, 20.6, 14.5, 14.4. **HRMS** (ESI): m/z $[M+H]^+$calcd for $[C_{26}H_{30}N_3O]^+$ required 400.2384, found 400.2393. $[\alpha]_D^{25}$ = -61 (c = 0.1, CH_2Cl_2). The product was analyzed by HPLC to determine the enantiomeric excess: 99% ee (CHIRALPAK AS-H, hexane/*i*-PrOH =95/5, detector: 254 nm, T = 25 ºC, flow rate: 0.8 mL/min), t_1 (minor) = 6.624 min, t_2 (major) = 7.499 min.

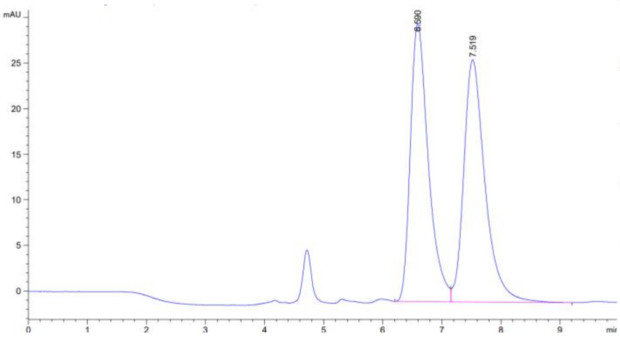

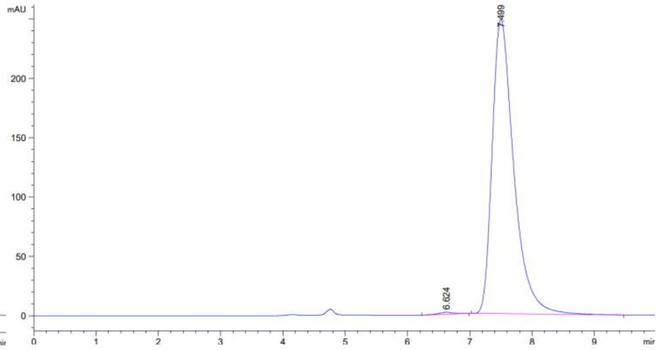

Peak	RetTime	Area	Height	Area
1	6.590	644.18988	30.44156	49.1752
2	7.519	665.79889	26.56727	50.8248

Peak	RetTime	Area	Height	Area
1	6.624	33.79324	1.77855	0.5604
2	7.499	5995.98828	247.92296	99.4396

(*R*)-3,4-dibutyl-2-(5,6,7,8-tetrahydro-9H-pyrido[2,3-b]indol-9-yl)isoquinolin-1(2H)-one (3ar)

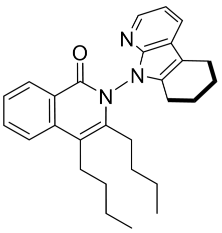

Yield: 62.3 mg (73%). White solid, mp: 159-160 °C. ^{1}**H NMR** (600 MHz, $CDCl_3$) δ 8.37 (d, *J* = 7.9 Hz, 1H), 8.18 (dd, *J* = 4.7, 1.2 Hz, 1H), 7.79 (dd, *J* = 7.7, 1.3 Hz, 1H), 7.71 (d, *J* = 3.6 Hz, 2H), 7.44 – 7.42 (m, 1H), 7.07 (dd, *J* = 7.7, 4.8 Hz, 1H), 2.81 – 2.72 (m, 4H), 2.57 – 2.52 (m, 1H), 2.49 – 2.38 (m, 3H), 1.96 – 1.92 (m, 2H), 1.91 – 1.86 (m, 2H), 1.71 – 1.67 (m, 1H), 1.66 – 1.62 (m, 2H), 1.56 – 1.47 (m, 4H), 1.46 – 1.38 (m, 2H), 1.14 – 1.05 (m, 2H), 1.02 (t, *J* = 7.3 Hz, 3H), 0.61 (t, *J* = 7.4 Hz, 3H). ^{13}C NMR (151 MHz, $CDCl_3$) δ 161.2, 147.7, 142.7, 142.0, 137.2, 137.1, 132.9, 128.9, 126.1, 126.0, 125.4, 123.1, 119.4, 116.8, 114.4, 108.9, 32.4, 31.0, 28.7, 27.7, 23.2, 22.9, 22.7, 22.4, 21.3, 20.6, 14.0, 13.2. **HRMS** (ESI): m/z $[M+H]^+$calcd for $[C_{28}H_{34}N_3O]^+$ required 428.2697, found 428.2700. $[\alpha]_D^{25}$ = -56 (c = 0.1, CH_2Cl_2). The product was analyzed by HPLC to determine the enantiomeric excess: 98% ee (CHIRALPAK AS-H, hexane/*i*-PrOH =95/5, detector: 254 nm, T = 25 °C, flow rate: 0.8 mL/min), t_1 (minor) = 5.957 min, t_2 (major) = 6.450 min.

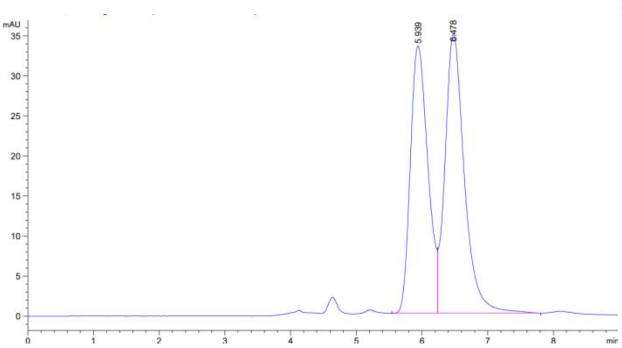

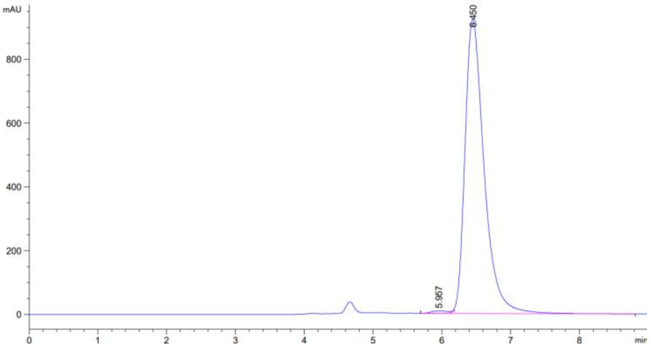

Peak	RetTime	Area	Height	Area
1	5.939	615.13147	33.35830	46.1862
2	6.478	716.71948	34.84469	53.8138

Peak	RetTime	Area	Height	Area
1	5.957	138.01343	8.72177	0.7664
2	6.450	1.78701e4	922.61829	99.2336

(*R*)-3,4-bis(hydroxymethyl)-2-(5,6,7,8-tetrahydro-9H-pyrido[2,3-b]indol-9-yl)isoquinolin-1(2H)-one (3as)

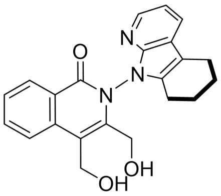

Yield: 67.5 mg (90%). White solid, mp: 137-138 °C. ^{1}**H NMR** (600 MHz, $CDCl_3$) δ 8.33 (d, *J* = 7.7 Hz, 1H), 7.91 (t, *J* = 6.2 Hz, 2H), 7.80 (d, *J* = 7.4 Hz, 1H), 7.70 (t, *J* = 7.3 Hz, 1H), 7.47 (t, *J* = 7.5 Hz, 1H), 7.05 (dd, *J* = 7.7, 4.9 Hz, 1H), 4.78 (s, 2H), 4.40 (q, *J* = 13.3 Hz, 2H), 2.76 – 2.66 (m, 2H), 2.43 – 2.33 (m, 2H), 1.92 – 1.77 (m, 4H). ^{13}C NMR (151 MHz, $CDCl_3$) δ 161.0, 147.8, 142.1, 142.0, 138.0, 136.2, 133.6, 128.6, 127.6, 127.0, 125.9, 124.2, 120.2, 117.4, 116.2, 109.5, 57.5, 57.3, 22.6, 22.2, 21.0, 20.5. **HRMS** (ESI): m/z $[M+H]^+$calcd for $[C_{22}H_{22}N_3O_3]^+$ required 376.1656, found 376.1659. $[\alpha]_D^{25}$ = -40 (c = 0.1, CH_2Cl_2). The product was analyzed by HPLC to determine the enantiomeric excess: 97% ee (CHIRALPAK AS-H, hexane/*i*-PrOH =85/15, detector: 254 nm, T = 25 °C, flow rate: 1 mL/min), t_1 (minor) = 6.227 min, t_2 (major) = 11.290 min.

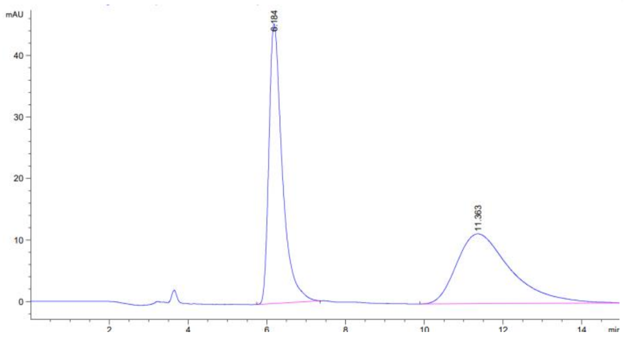

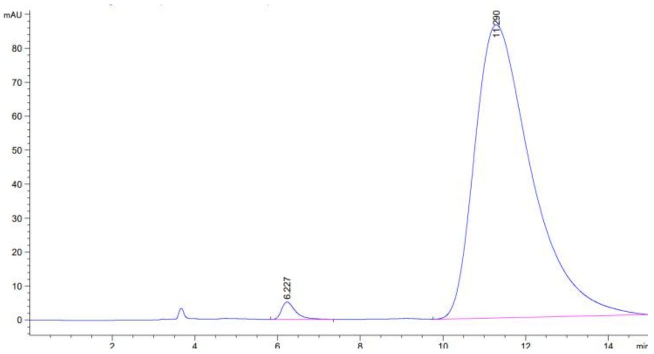

Peak	RetTime	Area	Height	Area
1	6.184	1071.43384	45.44476	49.7528
2	11.363	1082.08069	11.34843	50.2472

Peak	RetTime	Area	Height	Area
1	6.227	120.53471	5.12764	1.4472
2	11.290	8208.35254	86.25107	98.5528

(*R*)-4-methyl-3-phenyl-2-(5,6,7,8-tetrahydro-9H-pyrido[2,3-b]indol-9-yl)isoquinolin-1(2H)-one (3at)

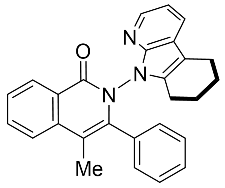

Yield: 40.5 mg (50%). White solid, mp: 162-163 °C. ^{1}**H NMR** (600 MHz, $CDCl_3$) δ 8.49 (d, J = 7.9 Hz, 1H), 8.19 (d, J = 4.1 Hz, 1H), 7.78 – 7.77 (m, 2H), 7.58 (d, J = 7.2 Hz, 1H), 7.55 – 7.53 (m, 1H), 7.30 (d, J = 7.7 Hz, 1H), 7.25 – 7.23 (m, 2H), 7.15 (t, J = 6.8 Hz, 1H), 7.00 – 6.93 (m, 2H), 2.60 – 2.55 (m, 1H), 2.52 – 2.48 (m, 1H), 2.47 – 2.38 (m, 2H), 2.11 (s, 3H), 1.87 – 1.79 (m, 2H), 1.75 – 1.71 (m, 1H), 1.69 – 1.64 (m, 1H). ^{13}C NMR (151 MHz, $CDCl_3$) δ 161.1, 147.6, 142.3, 141.5, 137.7, 136.4, 133.2, 133.1, 129.7, 129.1, 128.9, 128.6, 127.6, 127.4, 127.0, 125.7, 123.8, 119.0, 116.5, 111.3, 108.1, 22.7, 22.3, 21.4, 20.3, 15.0. **HRMS** (ESI): m/z $[M+H]^+$calcd for $[C_{27}H_{24}N_3O]^+$ required 406.1914, found 406.1923. $[\alpha]_D^{25}$ = -43 (c = 0.1, CH_2Cl_2). The product was analyzed by HPLC to determine the enantiomeric excess: 98% ee (CHIRALPAK AS-H, hexane/*i*-PrOH =95/5, detector: 254 nm, T = 25 °C, flow rate: 0.8 mL/min), t_1 (minor) = 9.630 min, t_2 (major) = 12.896 min.

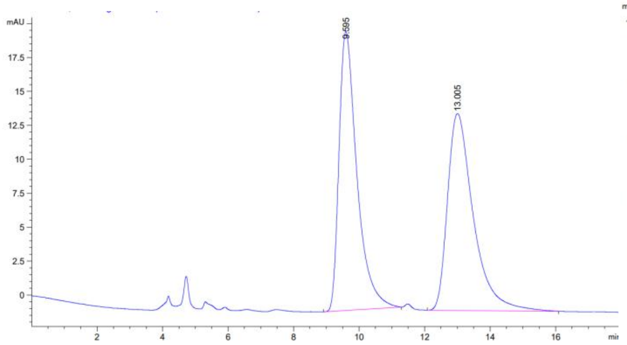

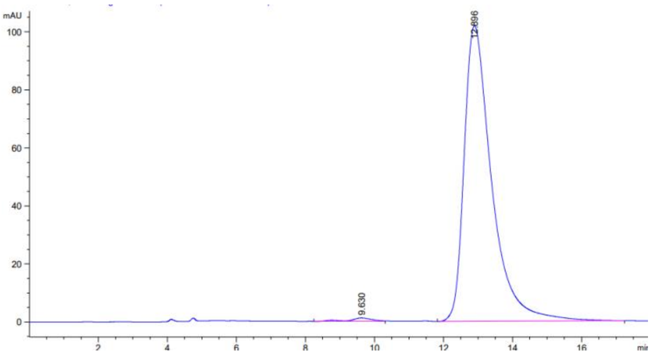

Peak	RetTime	Area	Height	Area
1	9.595	781.19989	20.56304	49.5593
2	13.005	795.09222	14.51331	50.4407

Peak	RetTime	Area	Height	Area
1	9.630	50.34890	1.14020	0.8844
2	12.896	5642.57031	101.85859	99.1156

12. NMR spectra for new compounds

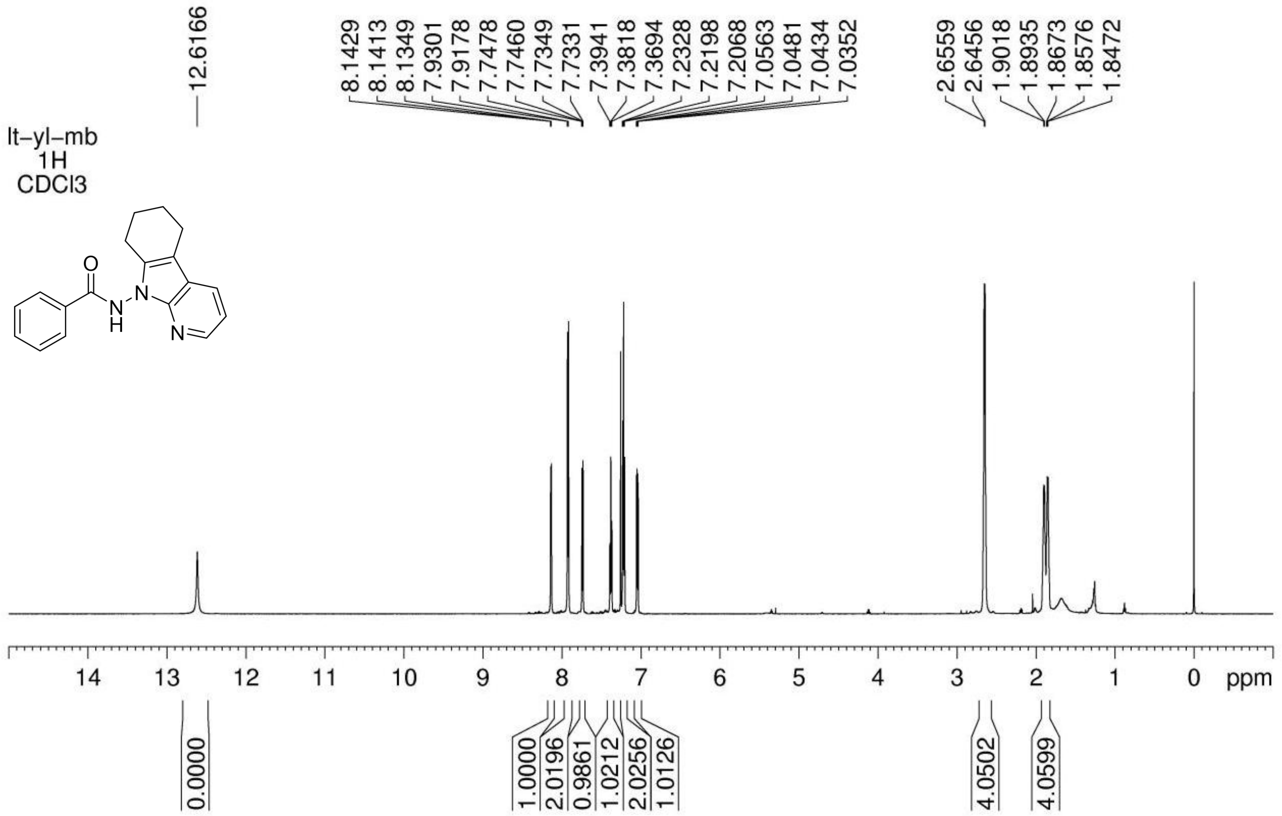

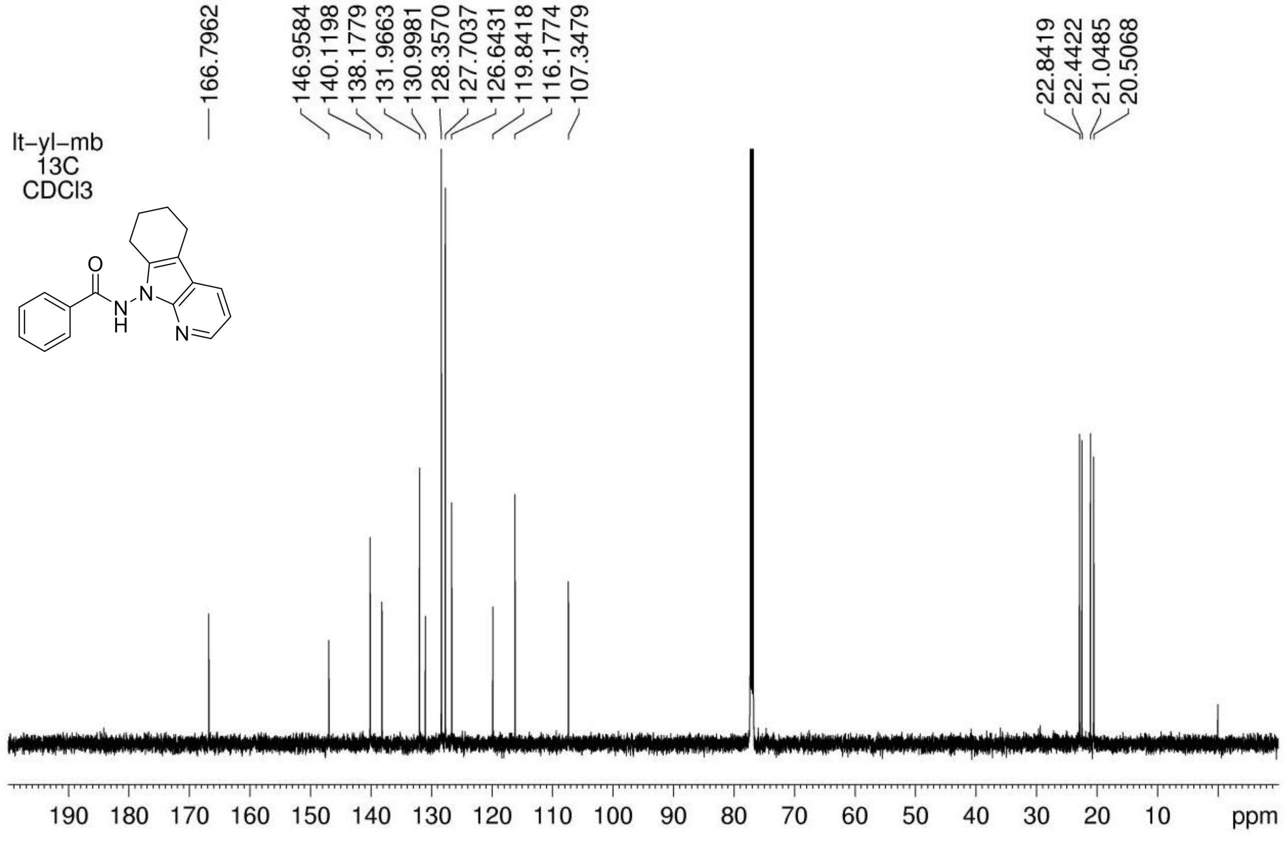

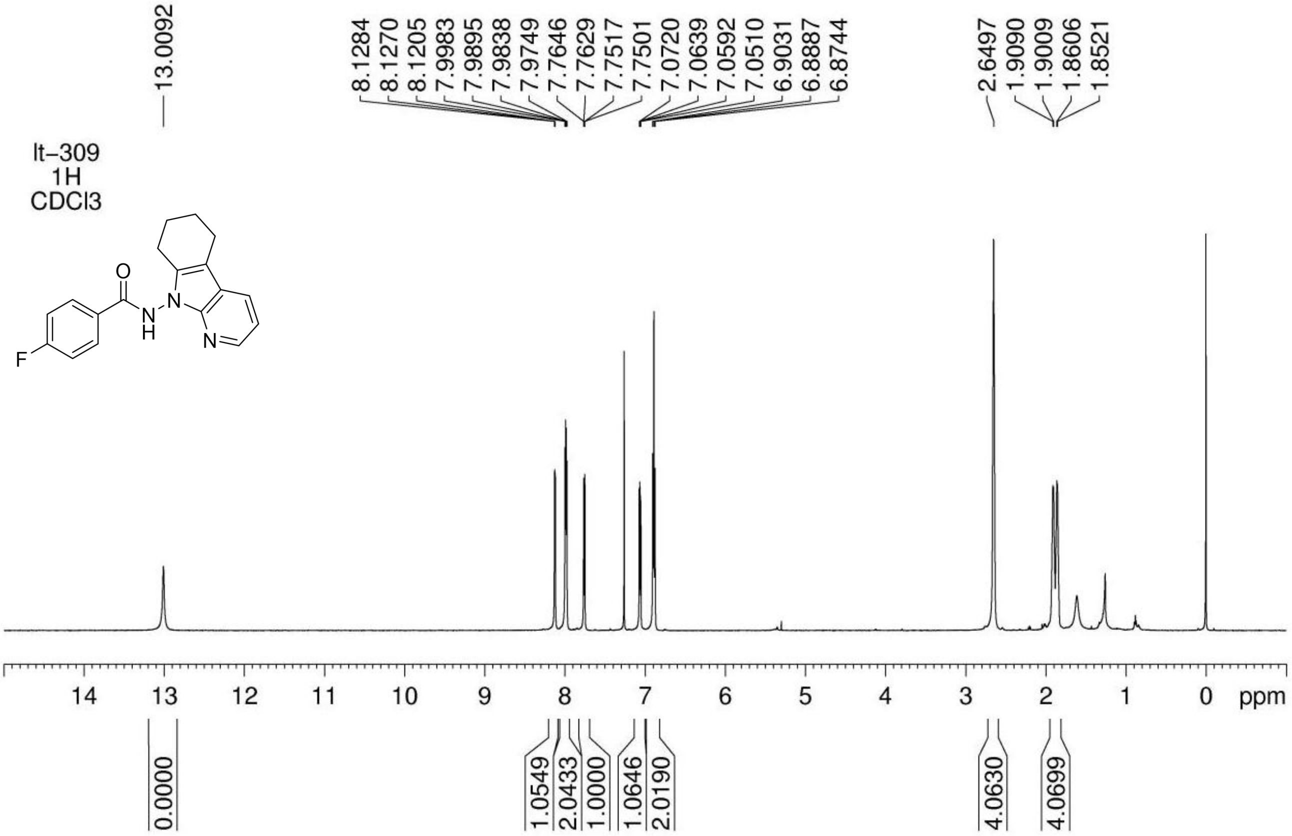
It–309
1H
CDCl3
O
N
H
N
F
N
13.0092
8.1284
8.1270
8.1205
7.9983
7.9895
7.9838
7.9749
7.7646
7.7629
7.7517
7.7501
7.0720
7.0639
7.0592
7.0510
6.9031
6.8887
6.8744
2.6497
1.9090
1.9009
1.8606
1.8521
14 13 12 11 10 9 8 7 6 5 4 3 2 1 0 ppm
0.0000
1.0549
2.0433
1.0000
1.0646
2.0190
4.0630
4.0699

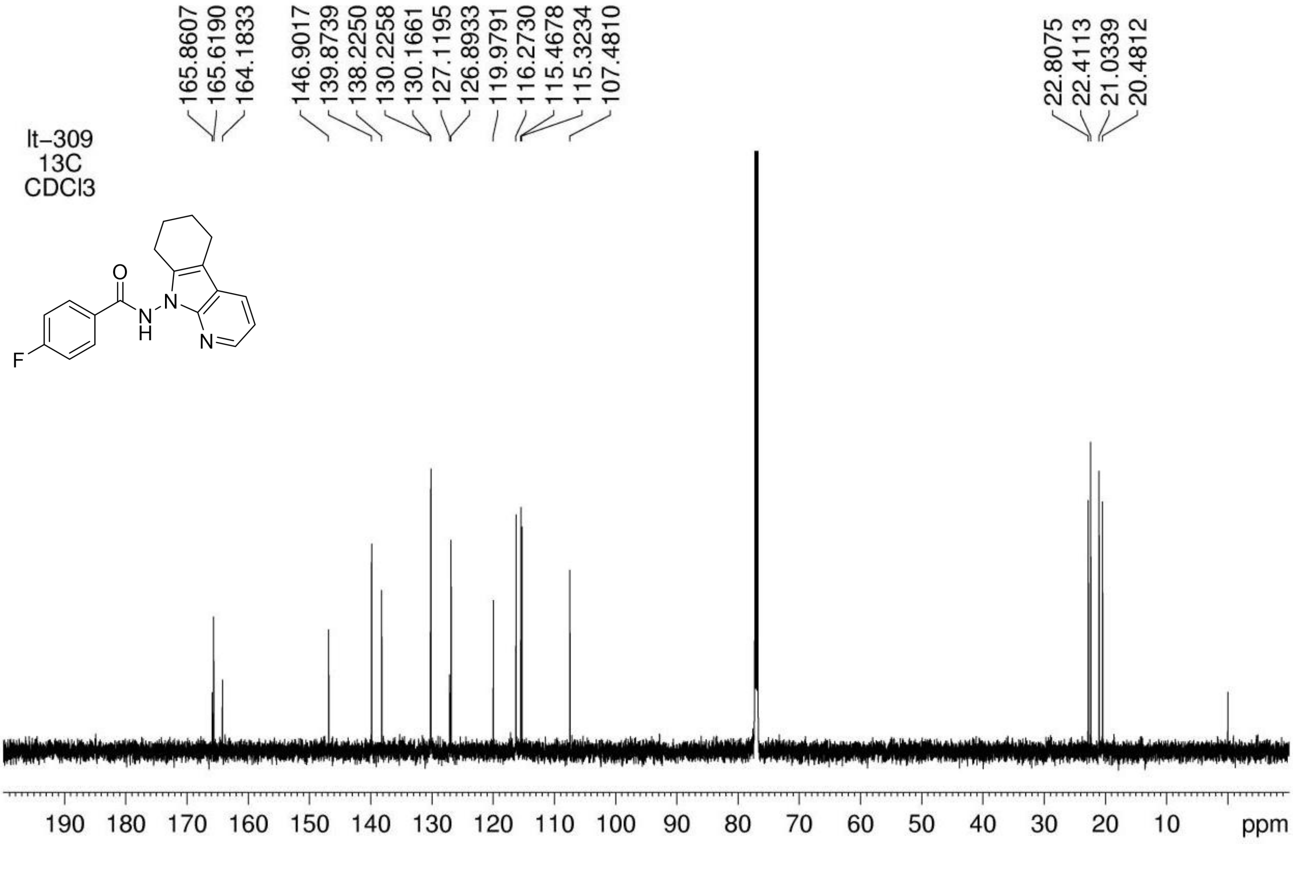
It–309
13C
CDCl3
O
N
H
N
F
N
165.8607
165.6190
164.1833
146.9017
139.8739
138.2250
130.2258
130.1661
127.1195
126.8933
119.9791
116.2730
115.4678
115.3234
107.4810
22.8075
22.4113
21.0339
20.4812
190 180 170 160 150 140 130 120 110 100 90 80 70 60 50 40 30 20 10 ppm

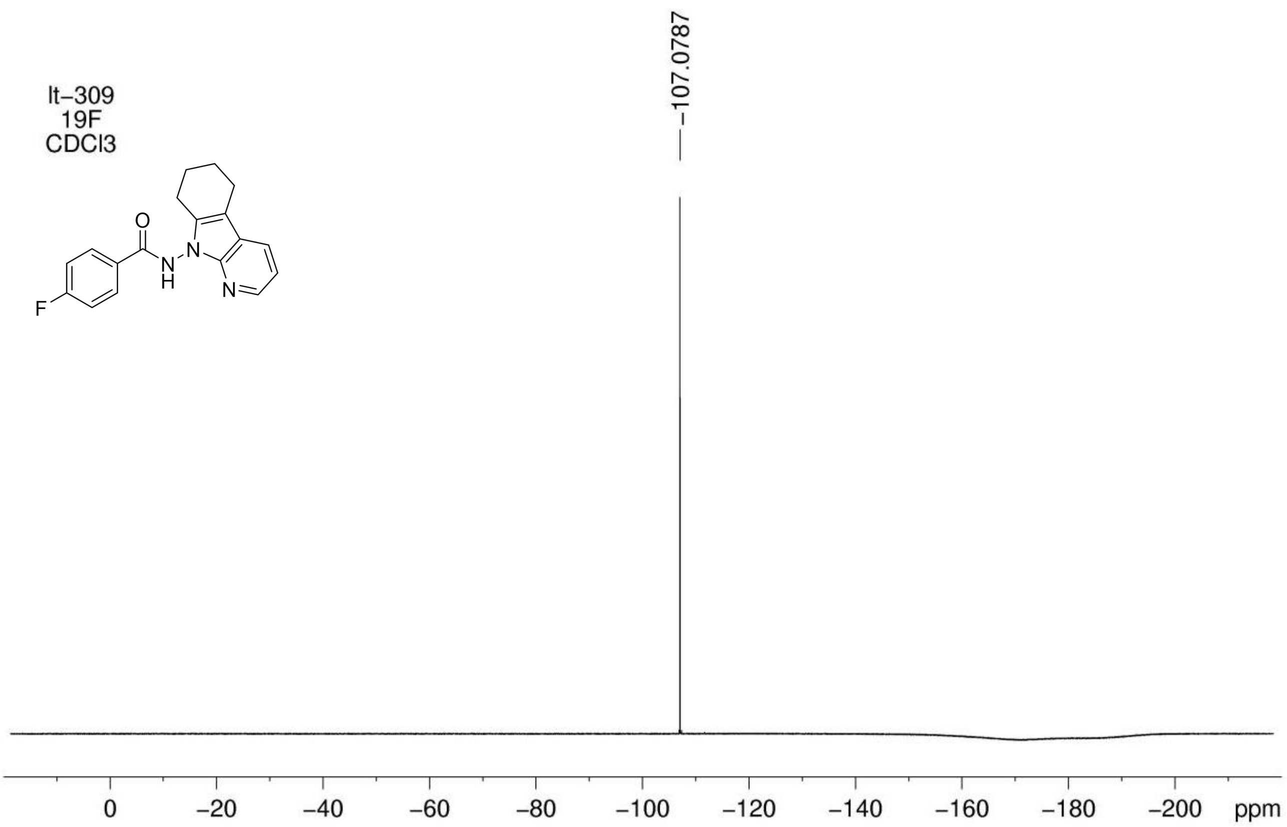
lt–309
19F
CDCl3
–107.0787
0
–20
–40
–60
–80
–100
–120
–140
–160
–180
–200
ppm

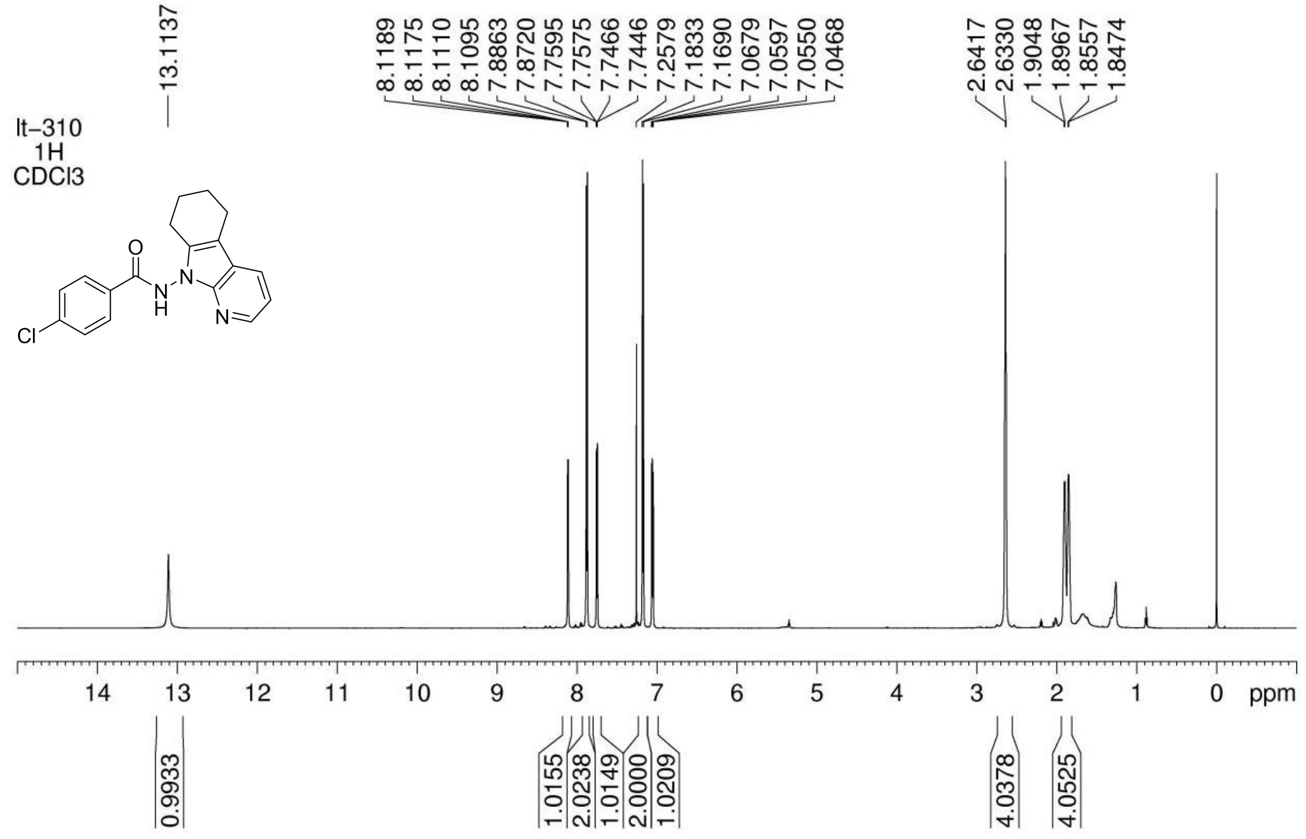
lt–310
1H
CDCl3
13.1137
8.1189
8.1175
8.1110
8.1095
7.8863
7.8720
7.7595
7.7575
7.7466
7.7446
7.2579
7.1833
7.1690
7.0679
7.0597
7.0550
7.0468
2.6417
2.6330
1.9048
1.8967
1.8557
1.8474
14 13 12 11 10 9 8 7 6 5 4 3 2 1 0 ppm
0.9933
1.0155
2.0238
1.0149
2.0000
1.0209
4.0378
4.0525

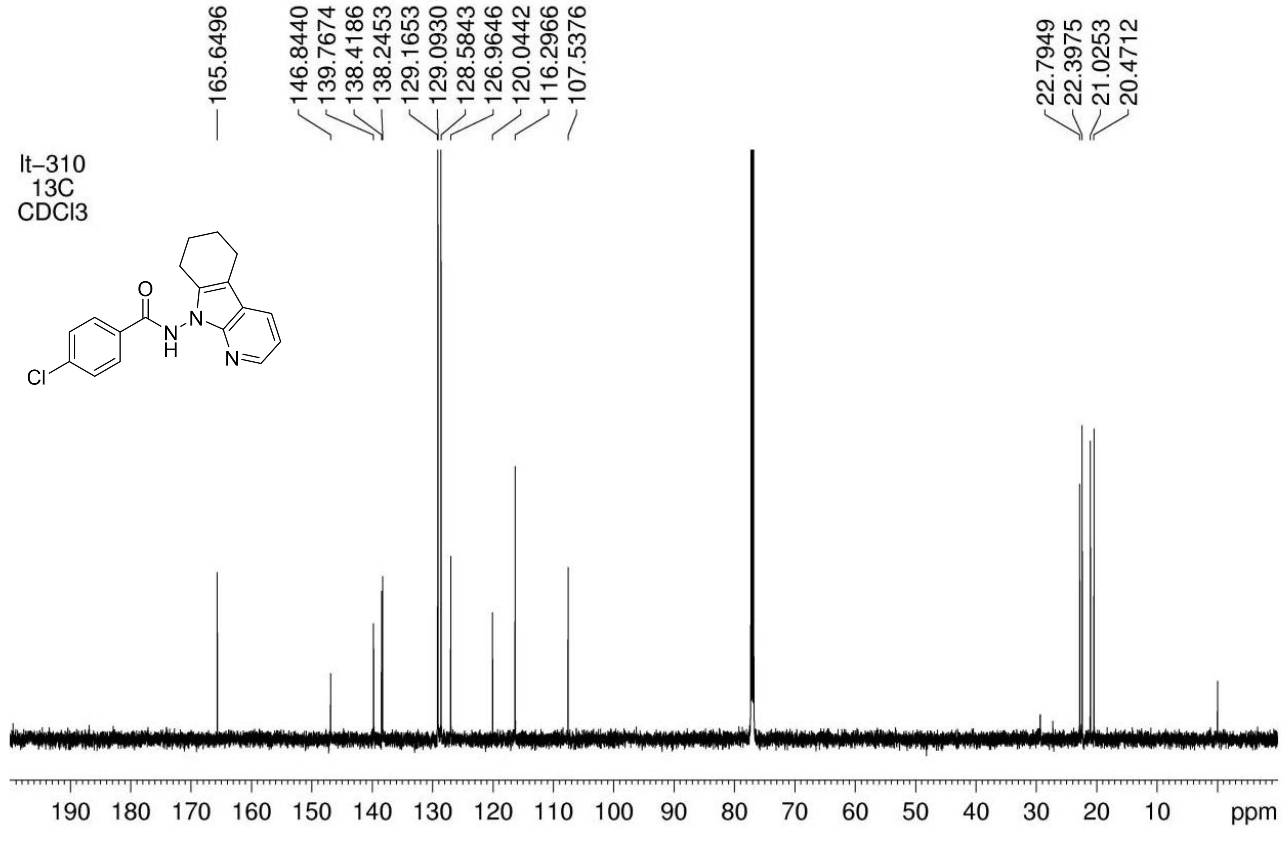
lt–310
13C
CDCl3
165.6496
146.8440
139.7674
138.4186
138.2453
129.1653
129.0930
128.5843
126.9646
120.0442
116.2966
107.5376
22.7949
22.3975
21.0253
20.4712
190 180 170 160 150 140 130 120 110 100 90 80 70 60 50 40 30 20 10 ppm

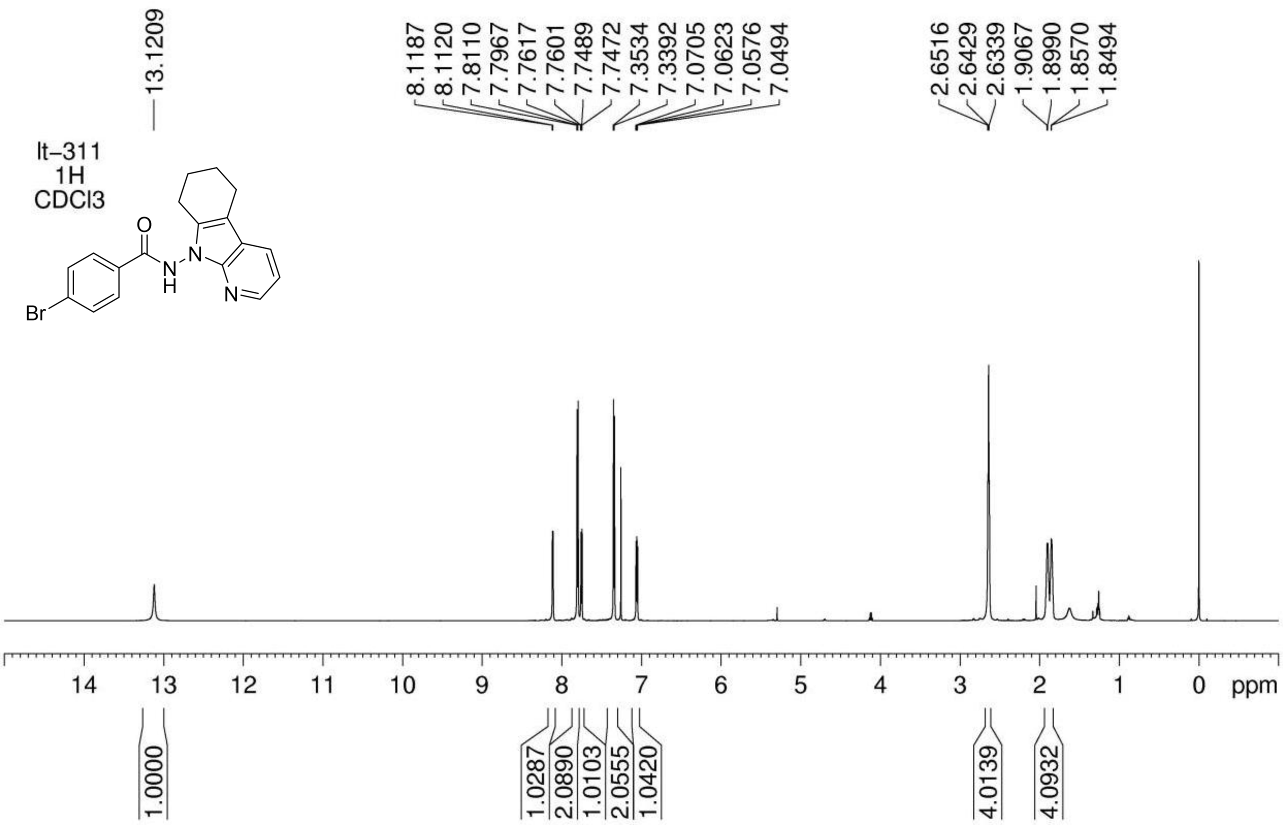
lt–311
1H
CDCl3
O
N
N
H
N
Br
13.1209
8.1187
8.1120
7.8110
7.7967
7.7617
7.7601
7.7489
7.7472
7.3534
7.3392
7.0705
7.0623
7.0576
7.0494
2.6516
2.6429
2.6339
1.9067
1.8990
1.8570
1.8494
14 13 12 11 10 9 8 7 6 5 4 3 2 1 0 ppm
1.0000
1.0287
2.0890
1.0103
2.0555
1.0420
4.0139
4.0932

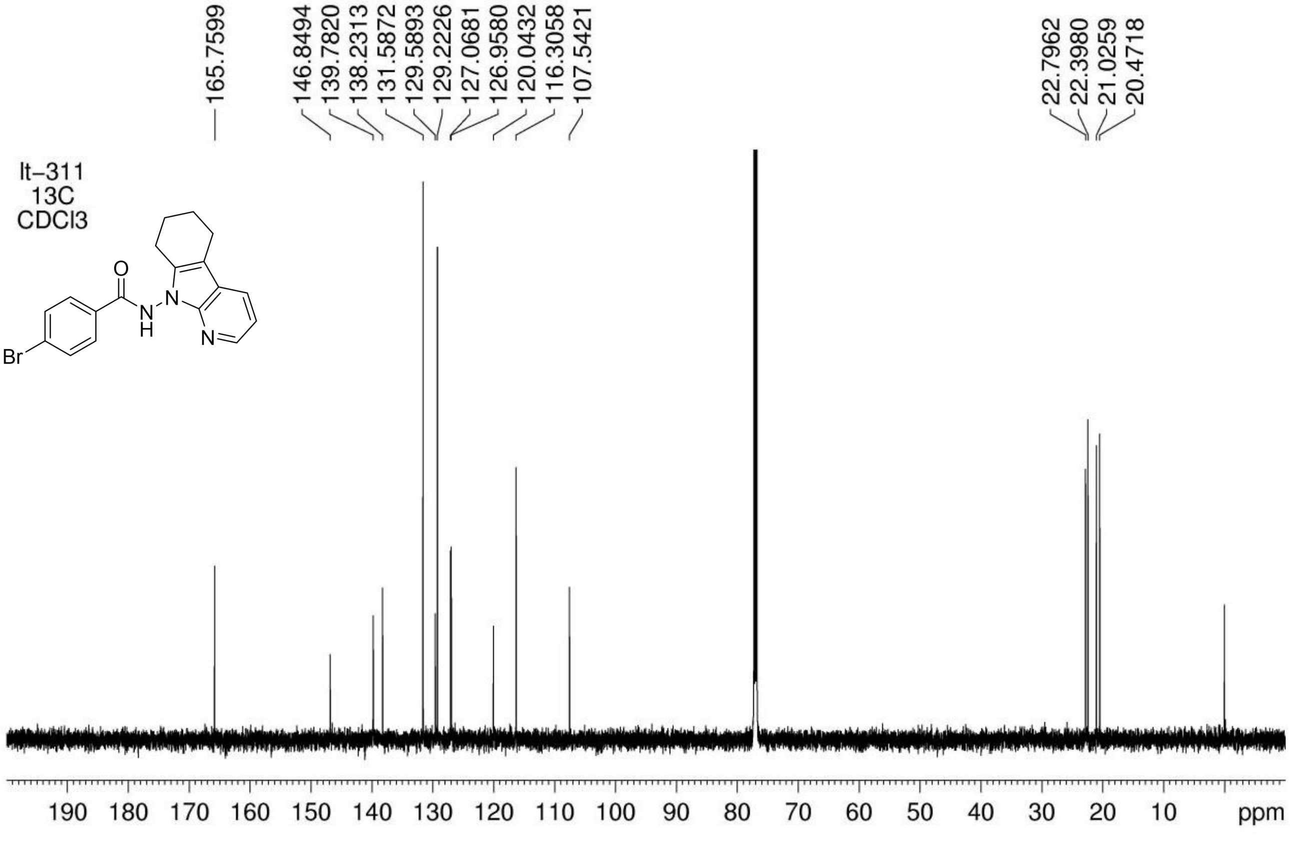
lt–311
13C
CDCl3
O
N
N
H
N
Br
165.7599
146.8494
139.7820
138.2313
131.5872
129.5893
129.2226
127.0681
126.9580
120.0432
116.3058
107.5421
22.7962
22.3980
21.0259
20.4718
190 180 170 160 150 140 130 120 110 100 90 80 70 60 50 40 30 20 10 ppm

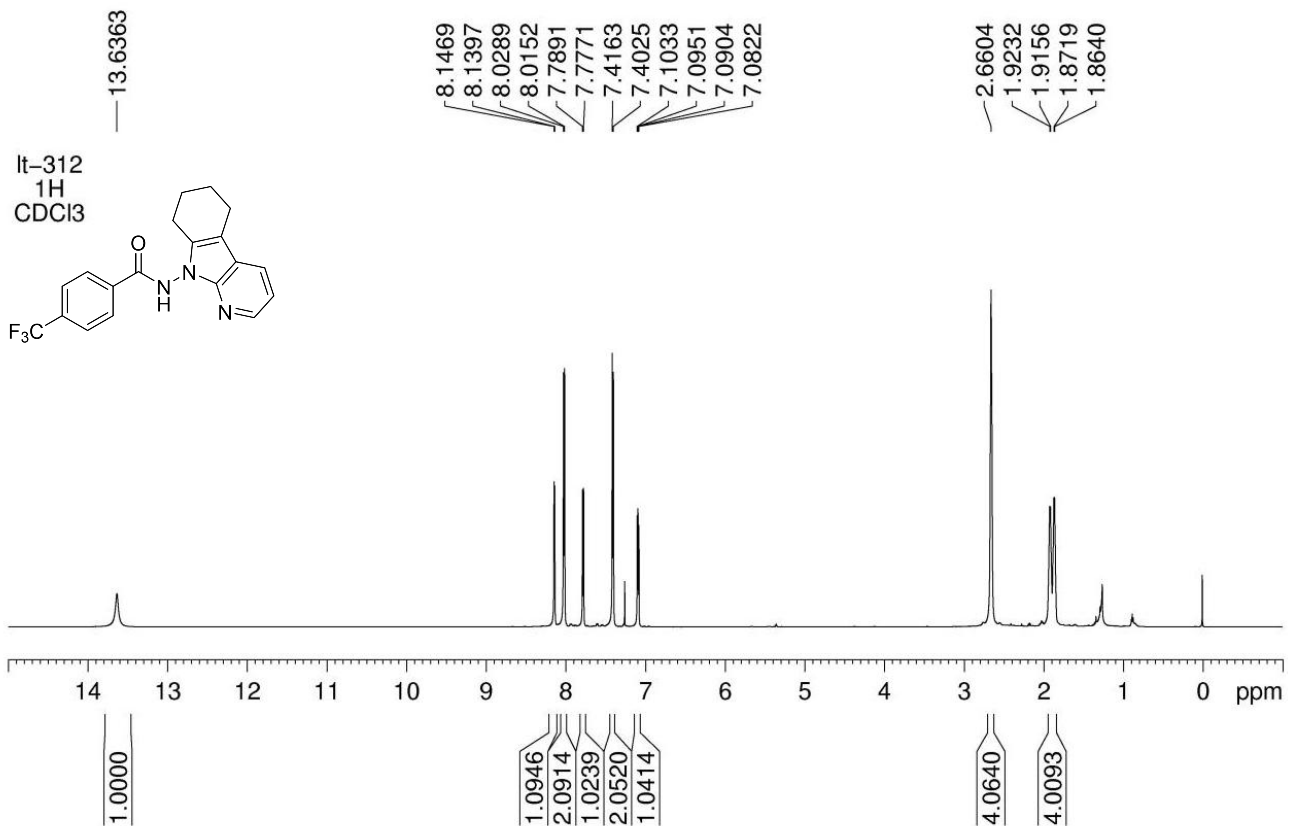
13.6363
8.1469
8.1397
8.0289
8.0152
7.7891
7.7771
7.4163
7.4025
7.1033
7.0951
7.0904
7.0822
2.6604
1.9232
1.9156
1.8719
1.8640
lt–312
1H
CDCl3
O
N
N
H
N
F3C
14 13 12 11 10 9 8 7 6 5 4 3 2 1 0 ppm
1.0000
1.0946
2.0914
1.0239
2.0520
1.0414
4.0640
4.0093

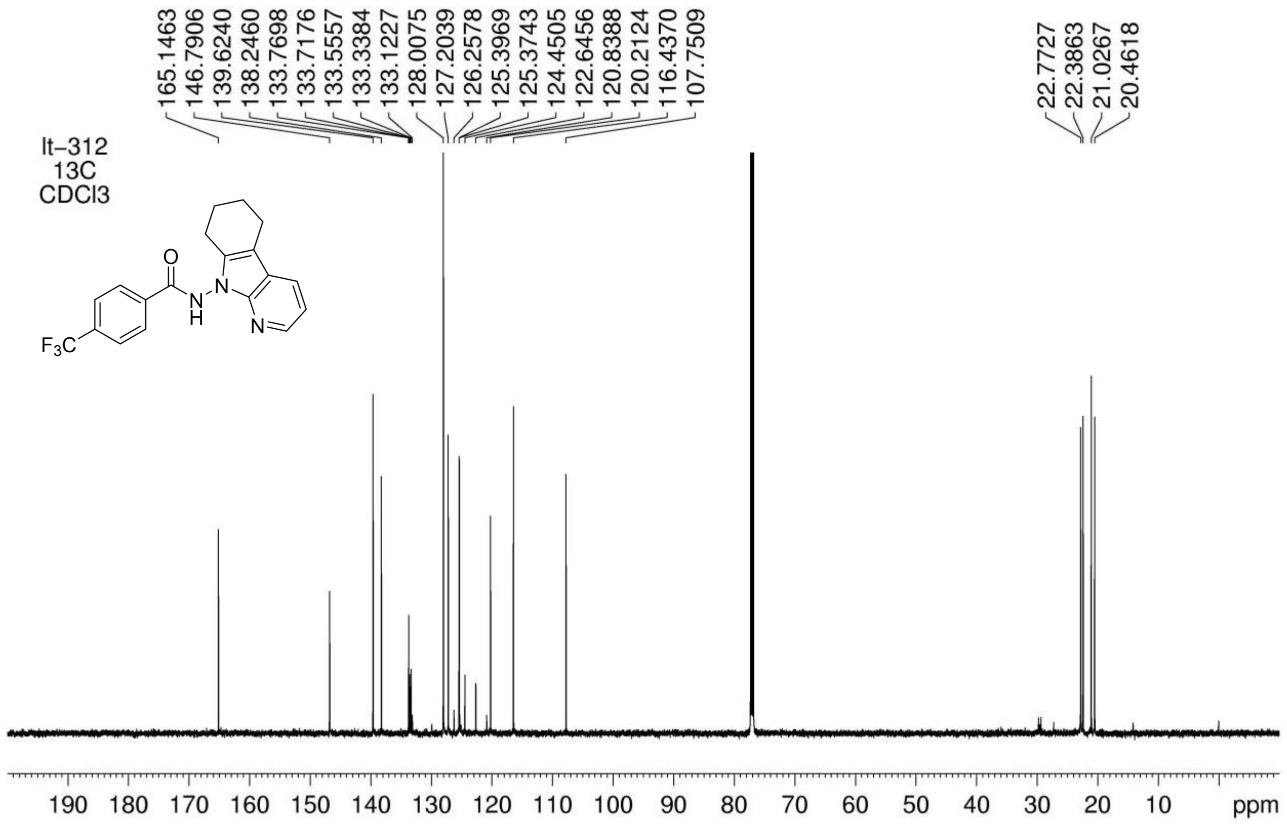
165.1463
146.7906
139.6240
138.2460
133.7698
133.7176
133.5557
133.3384
133.1227
128.0075
127.2039
126.2578
125.3969
125.3743
124.4505
122.6456
120.8388
120.2124
116.4370
107.7509
22.7727
22.3863
21.0267
20.4618
lt–312
13C
CDCl3
O
N
N
H
N
F3C
190 180 170 160 150 140 130 120 110 100 90 80 70 60 50 40 30 20 10 ppm

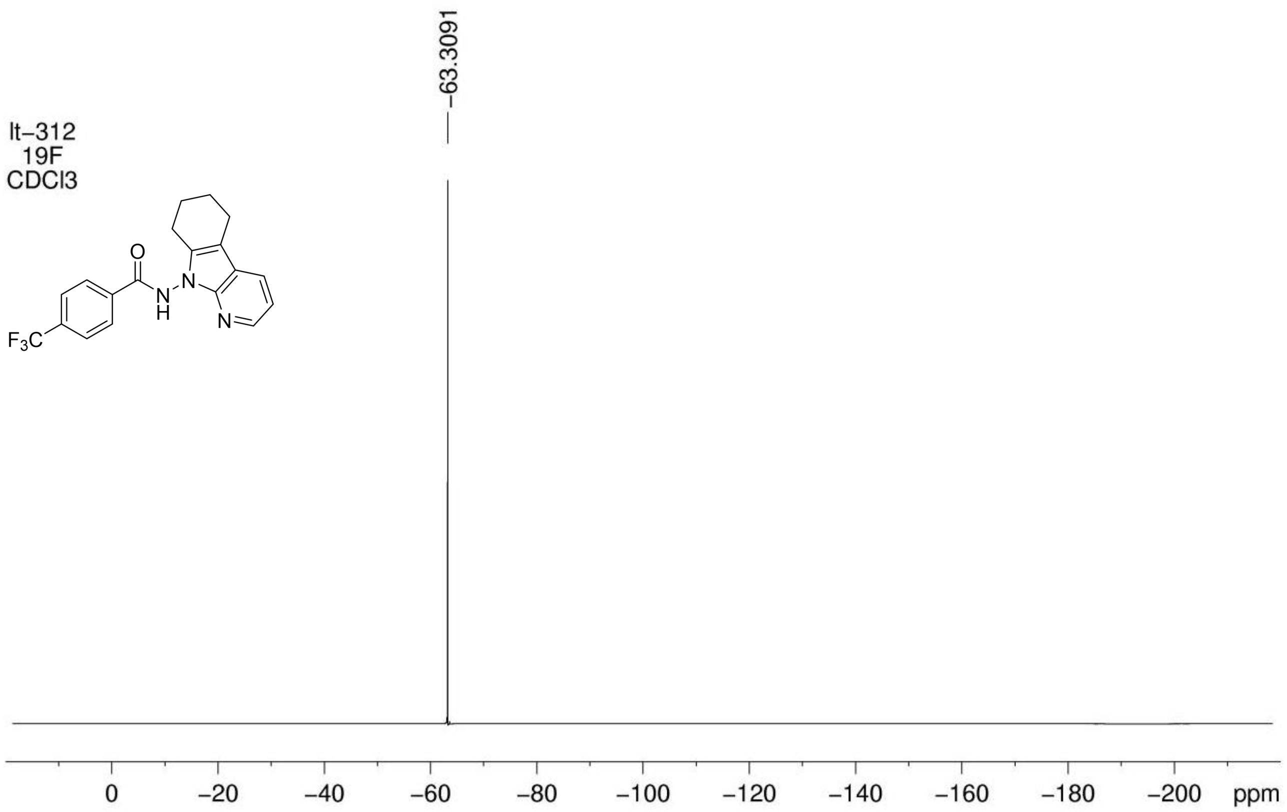

−63.3091
lt−312
19F
CDCl3
O
N
N
H
N
F_3C
0
−20
−40
−60
−80
−100
−120
−140
−160
−180
−200
ppm

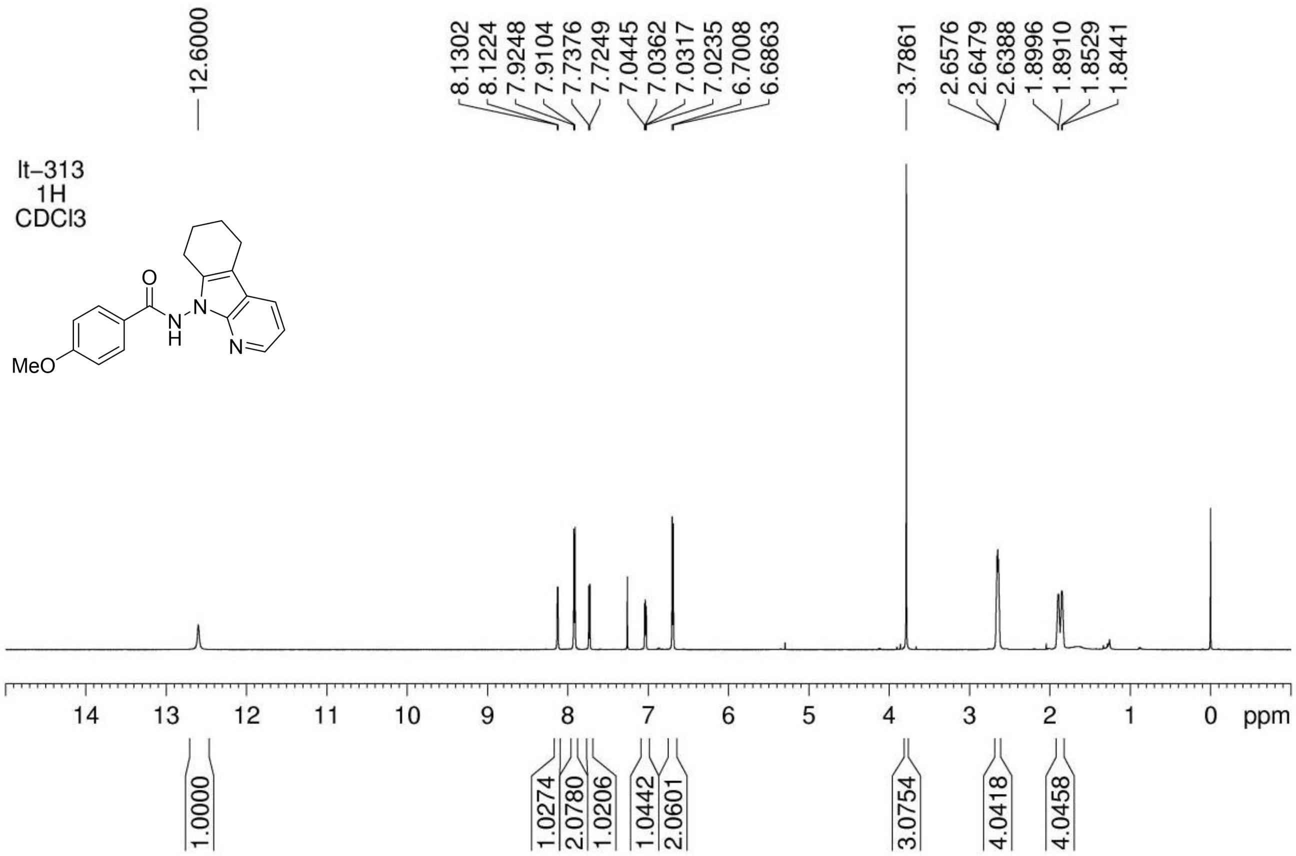
It–313
1H
CDCl3
MeO
O
N
H
N
N
12.6000
8.1302
8.1224
7.9248
7.9104
7.7376
7.7249
7.0445
7.0362
7.0317
7.0235
6.7008
6.6863
3.7861
2.6576
2.6479
2.6388
1.8996
1.8910
1.8529
1.8441
14 13 12 11 10 9 8 7 6 5 4 3 2 1 0 ppm
1.0000
1.0274
2.0780
1.0206
1.0442
2.0601
3.0754
4.0418
4.0458

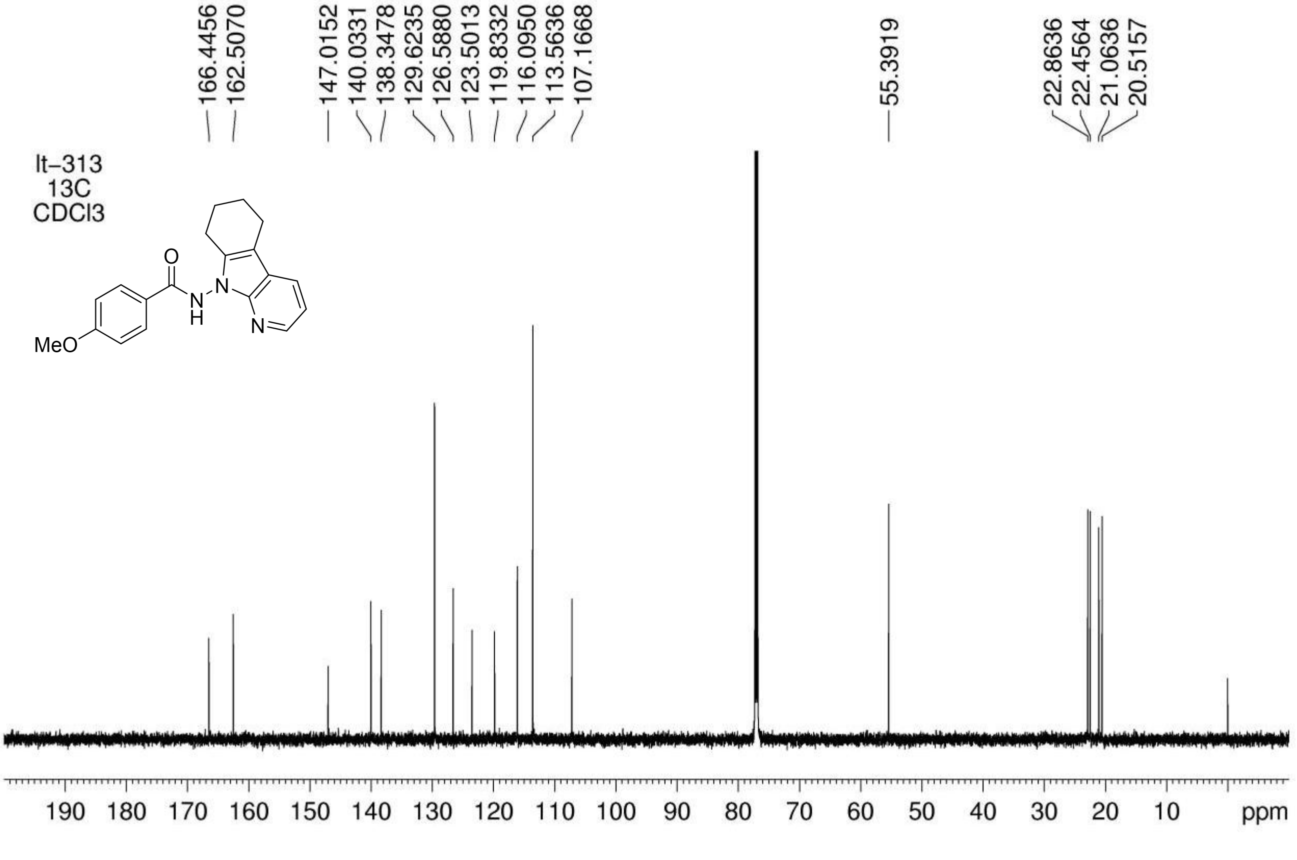
It–313
13C
CDCl3
MeO
O
N
H
N
N
166.4456
162.5070
147.0152
140.0331
138.3478
129.6235
126.5880
123.5013
119.8332
116.0950
113.5636
107.1668
55.3919
22.8636
22.4564
21.0636
20.5157
190 180 170 160 150 140 130 120 110 100 90 80 70 60 50 40 30 20 10 ppm

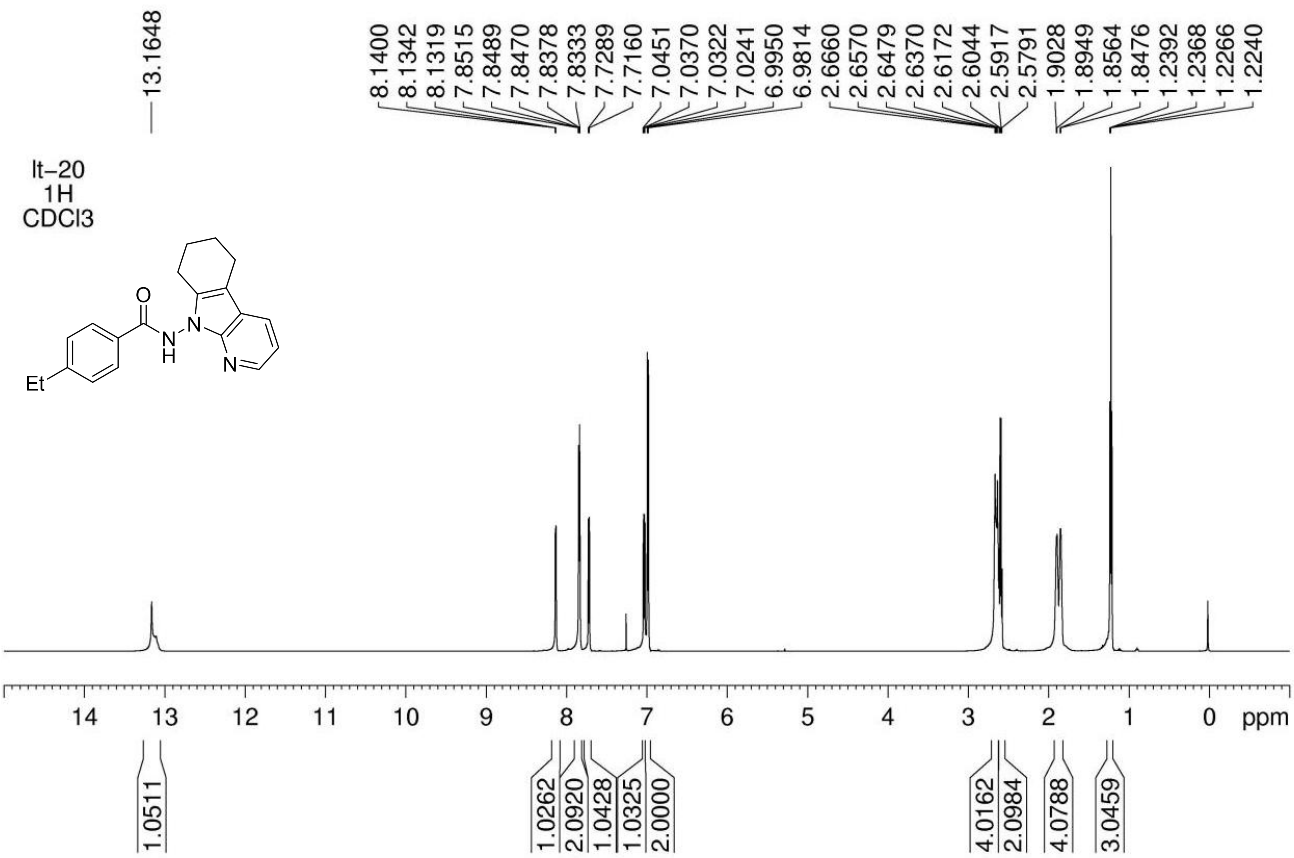
13.1648
8.1400
8.1342
8.1319
7.8515
7.8489
7.8470
7.8378
7.8333
7.7289
7.7160
7.0451
7.0370
7.0322
7.0241
6.9950
6.9814
2.6660
2.6570
2.6479
2.6370
2.6172
2.6044
2.5917
2.5791
1.9028
1.8949
1.8564
1.8476
1.2392
1.2368
1.2266
1.2240
It–20
1H
CDCl3
O
N
N
H
N
Et
14 13 12 11 10 9 8 7 6 5 4 3 2 1 0 ppm
1.0511
1.0262
2.0920
1.0428
1.0325
2.0000
4.0162
2.0984
4.0788
3.0459

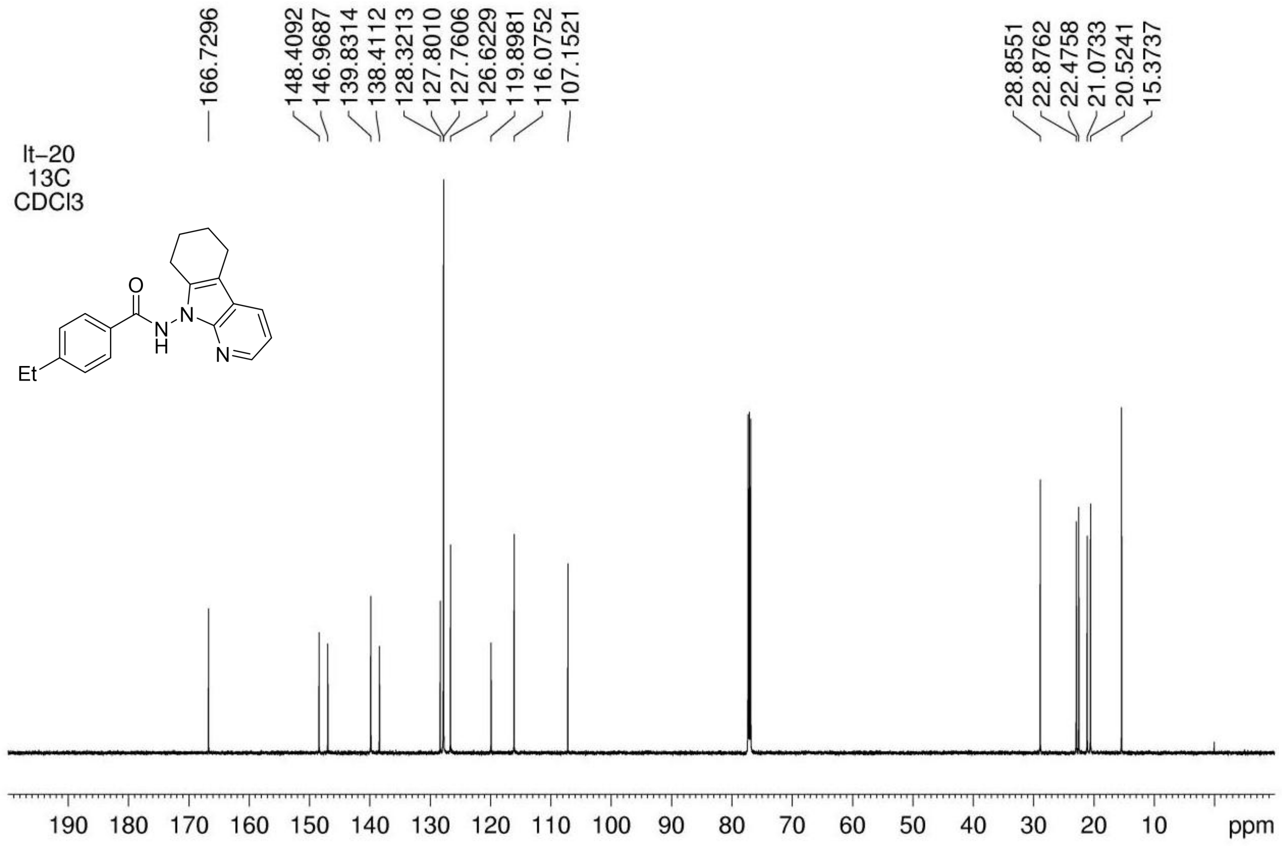
166.7296
148.4092
146.9687
139.8314
138.4112
128.3213
127.8010
127.7606
126.6229
119.8981
116.0752
107.1521
28.8551
22.8762
22.4758
21.0733
20.5241
15.3737
It–20
13C
CDCl3
O
N
N
H
N
Et
190 180 170 160 150 140 130 120 110 100 90 80 70 60 50 40 30 20 10 ppm

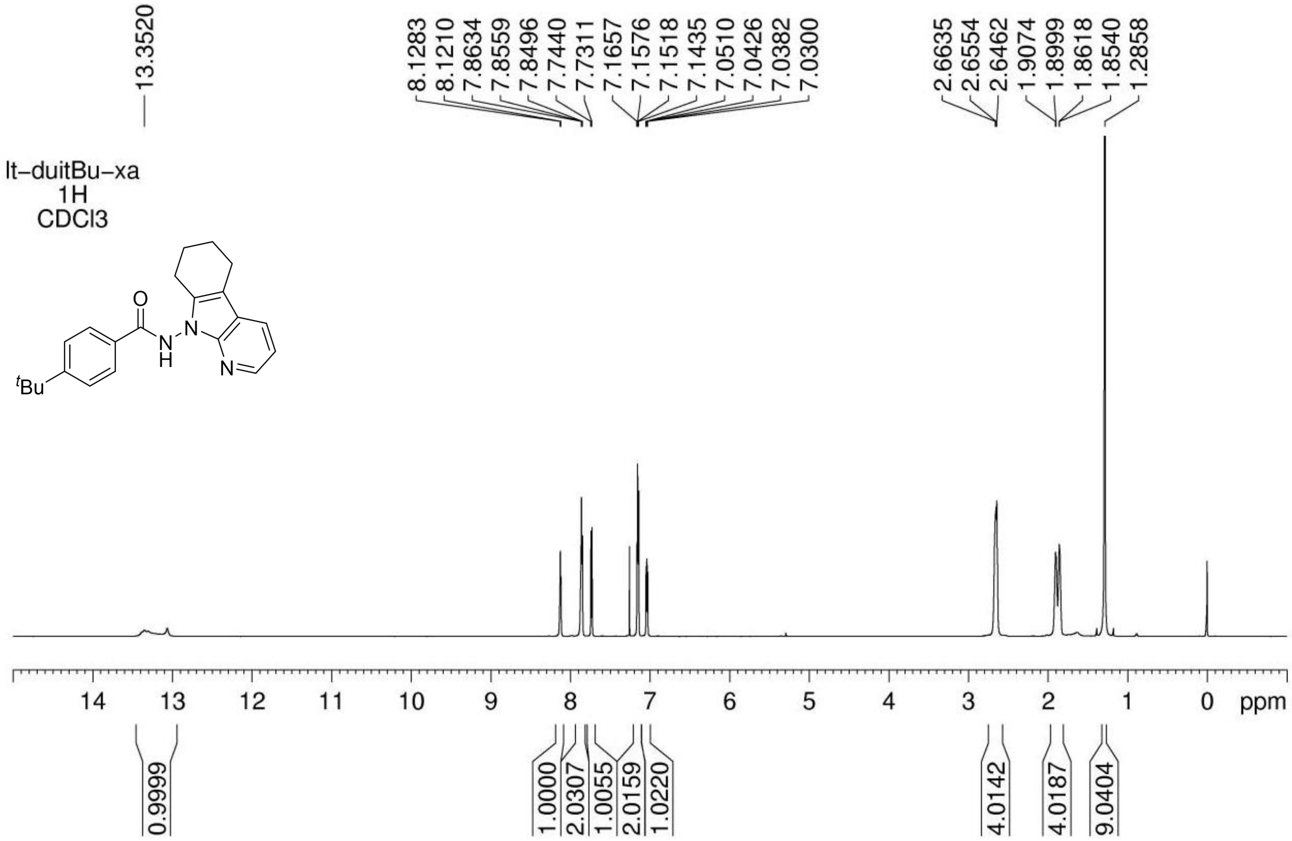
13.3520
8.1283
8.1210
7.8634
7.8559
7.8496
7.7440
7.7311
7.1657
7.1576
7.1518
7.1435
7.0510
7.0426
7.0382
7.0300
2.6635
2.6554
2.6462
1.9074
1.8999
1.8618
1.8540
1.2858
lt−duitBu−xa
1H
CDCl3
O
N
N
H
N
tBu
14 13 12 11 10 9 8 7 6 5 4 3 2 1 0 ppm
0.9999
1.0000
2.0307
1.0055
2.0159
1.0220
4.0142
4.0187
9.0404

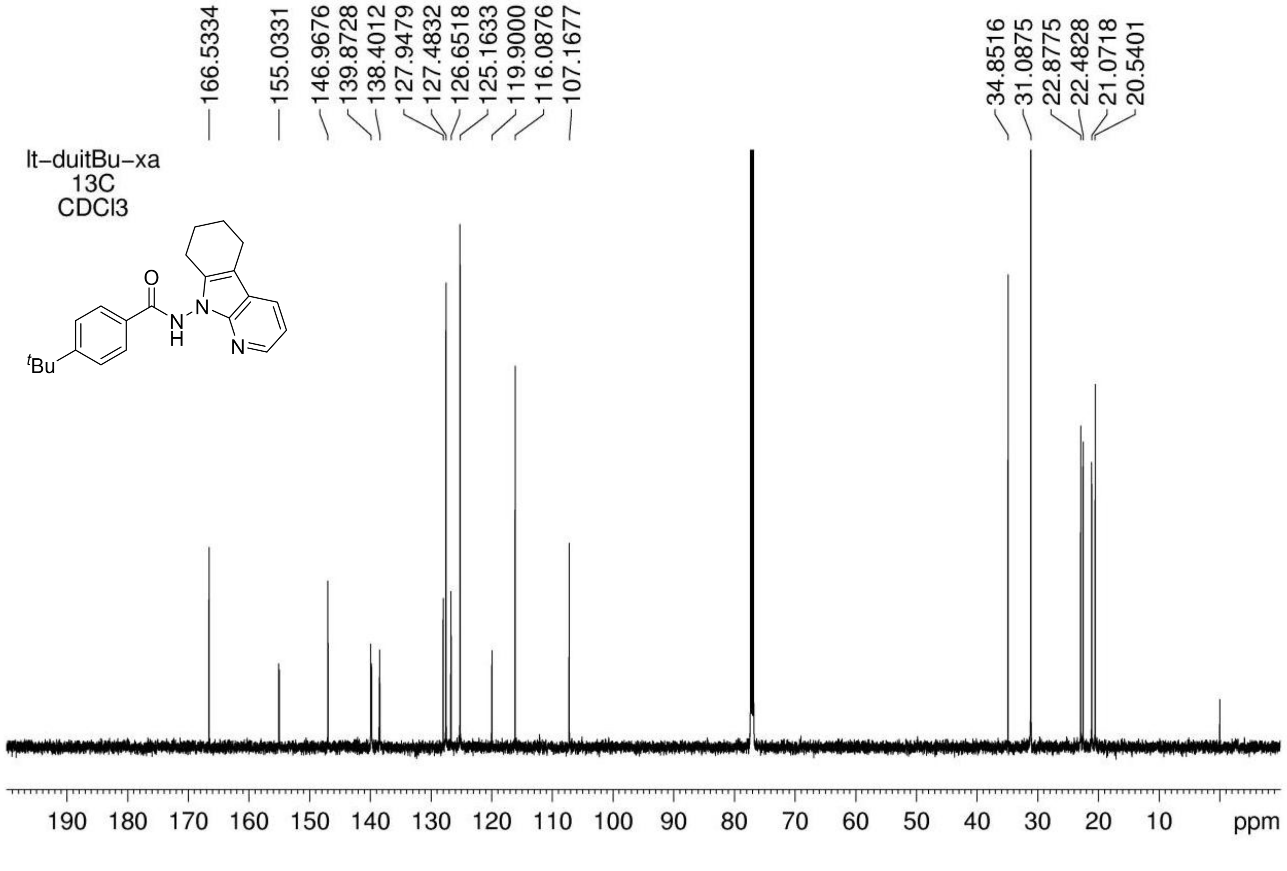
166.5334
155.0331
146.9676
139.8728
138.4012
127.9479
127.4832
126.6518
125.1633
119.9000
116.0876
107.1677
34.8516
31.0875
22.8775
22.4828
21.0718
20.5401
lt−duitBu−xa
13C
CDCl3
O
N
N
H
N
tBu
190 180 170 160 150 140 130 120 110 100 90 80 70 60 50 40 30 20 10 ppm

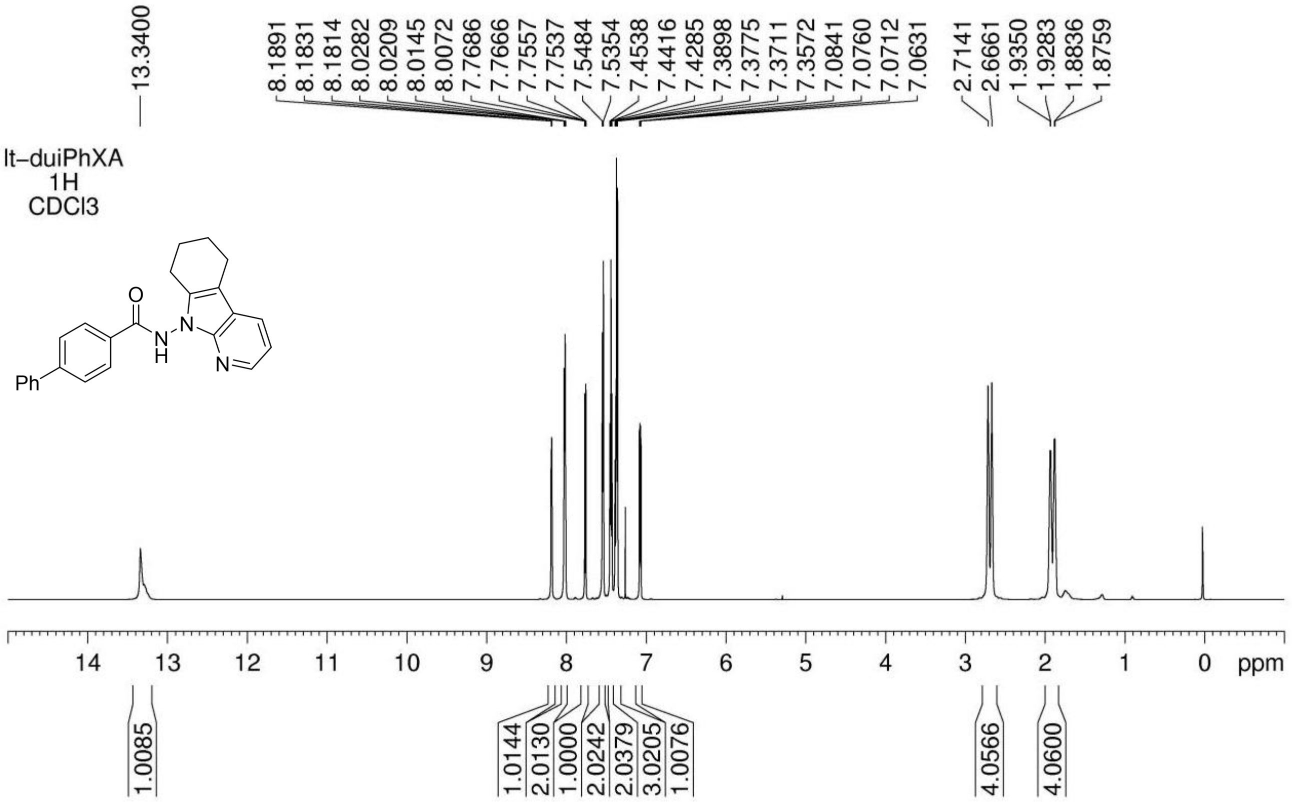
lt-duiPhXA
1H
CDCl3
O
N
H
N
Ph
13.3400
8.1891
8.1831
8.1814
8.0282
8.0209
8.0145
8.0072
7.7686
7.7666
7.7557
7.7537
7.5484
7.5354
7.4538
7.4416
7.4285
7.3898
7.3775
7.3711
7.3572
7.0841
7.0760
7.0712
7.0631
2.7141
2.6661
1.9350
1.9283
1.8836
1.8759
14 13 12 11 10 9 8 7 6 5 4 3 2 1 0 ppm
1.0085
1.0144
2.0130
1.0000
2.0242
2.0379
3.0205
1.0076
4.0566
4.0600

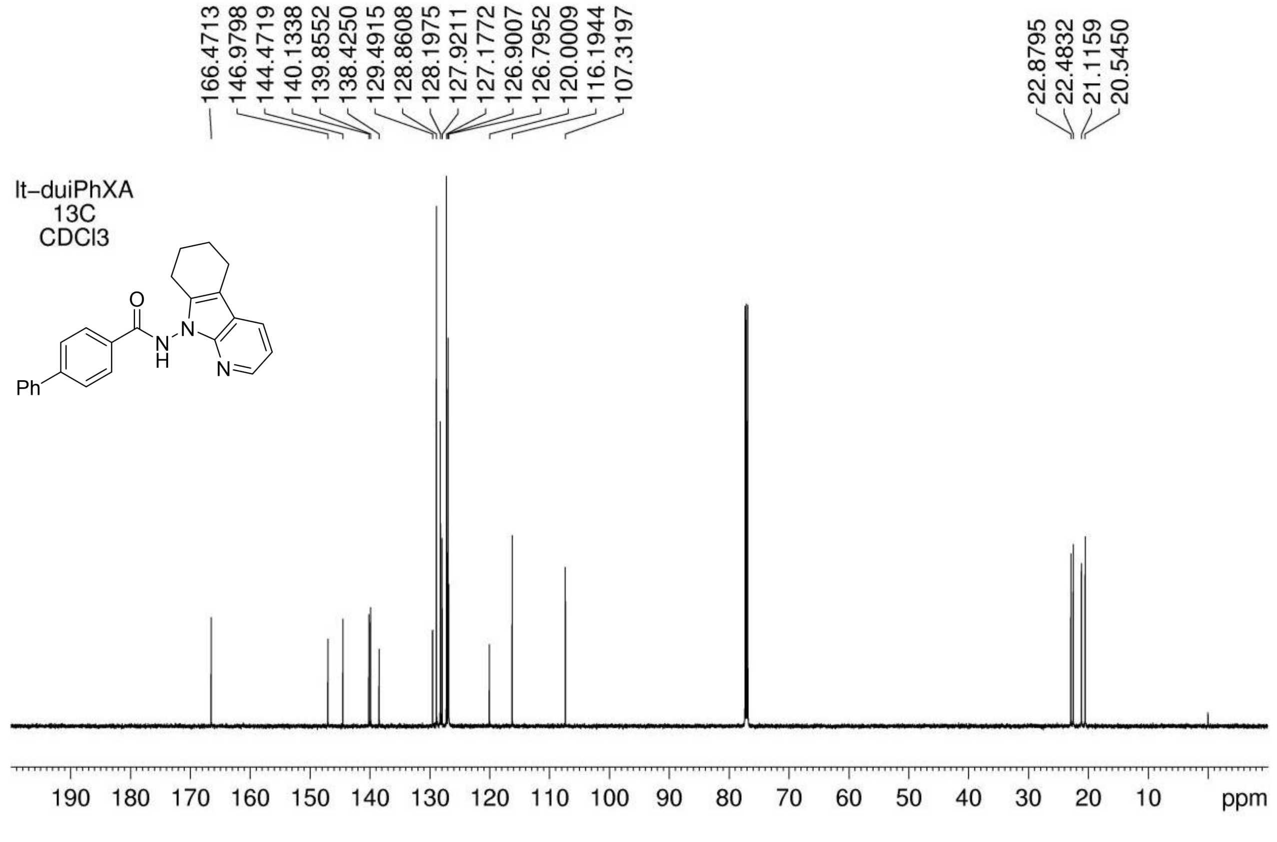
lt-duiPhXA
13C
CDCl3
O
N
H
N
Ph
166.4713
146.9798
144.4719
140.1338
139.8552
138.4250
129.4915
128.8608
128.1975
127.9211
127.1772
126.9007
126.7952
120.0009
116.1944
107.3197
22.8795
22.4832
21.1159
20.5450
190 180 170 160 150 140 130 120 110 100 90 80 70 60 50 40 30 20 10 ppm

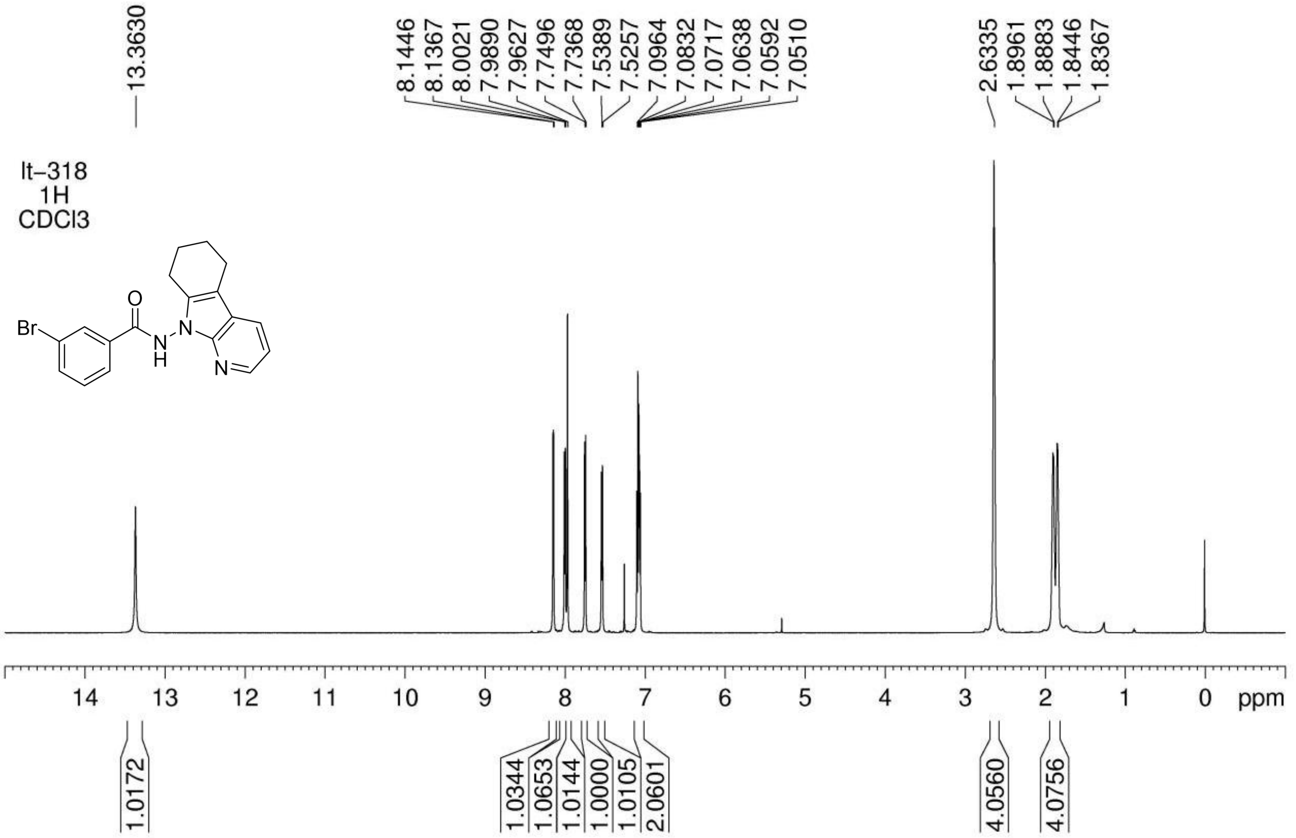
lt–318
1H
CDCl3
13.3630
8.1446
8.1367
8.0021
7.9890
7.9627
7.7496
7.7368
7.5389
7.5257
7.0964
7.0832
7.0717
7.0638
7.0592
7.0510
2.6335
1.8961
1.8883
1.8446
1.8367
1.0172
1.0344
1.0653
1.0144
1.0000
1.0105
2.0601
4.0560
4.0756
ppm

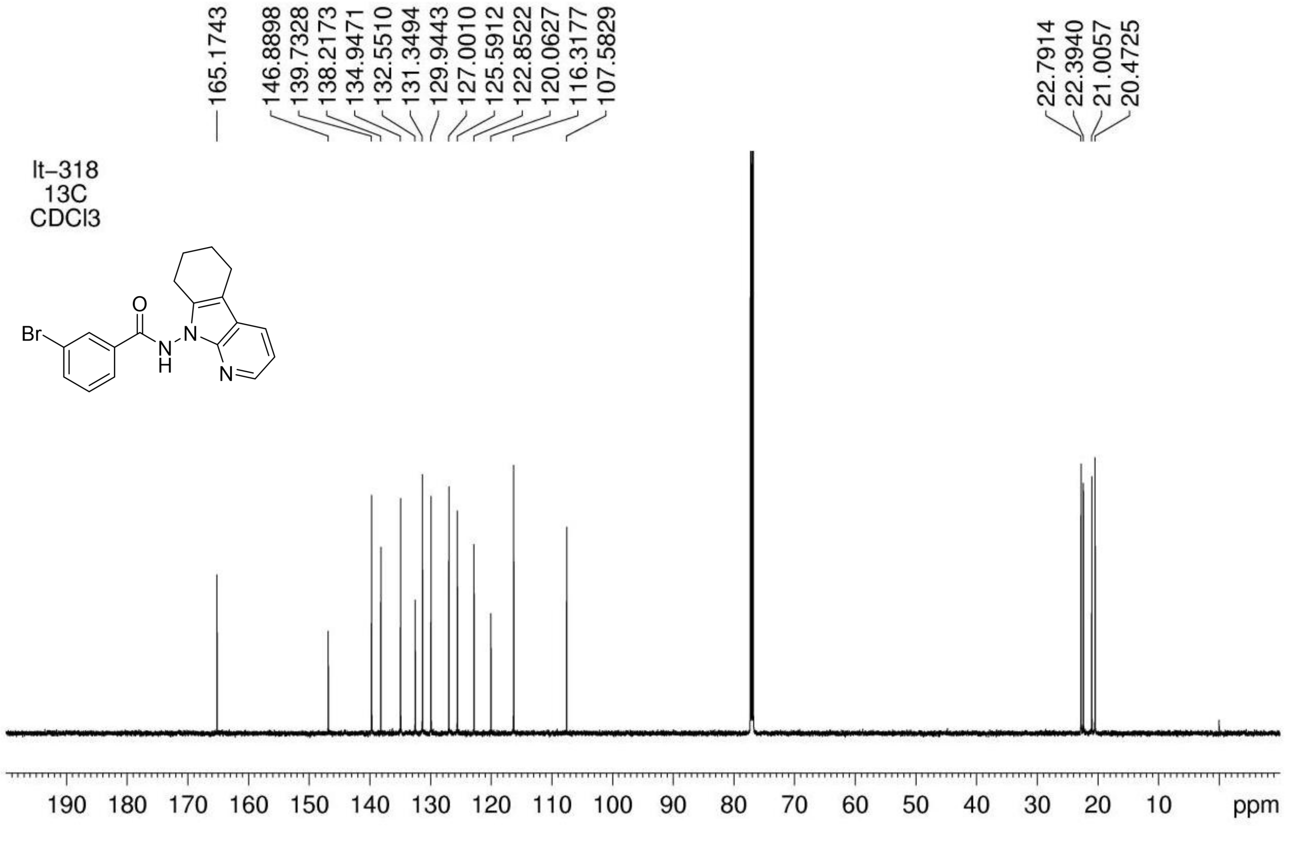
lt–318
13C
CDCl3
165.1743
146.8898
139.7328
138.2173
134.9471
132.5510
131.3494
129.9443
127.0010
125.5912
122.8522
120.0627
116.3177
107.5829
22.7914
22.3940
21.0057
20.4725
ppm

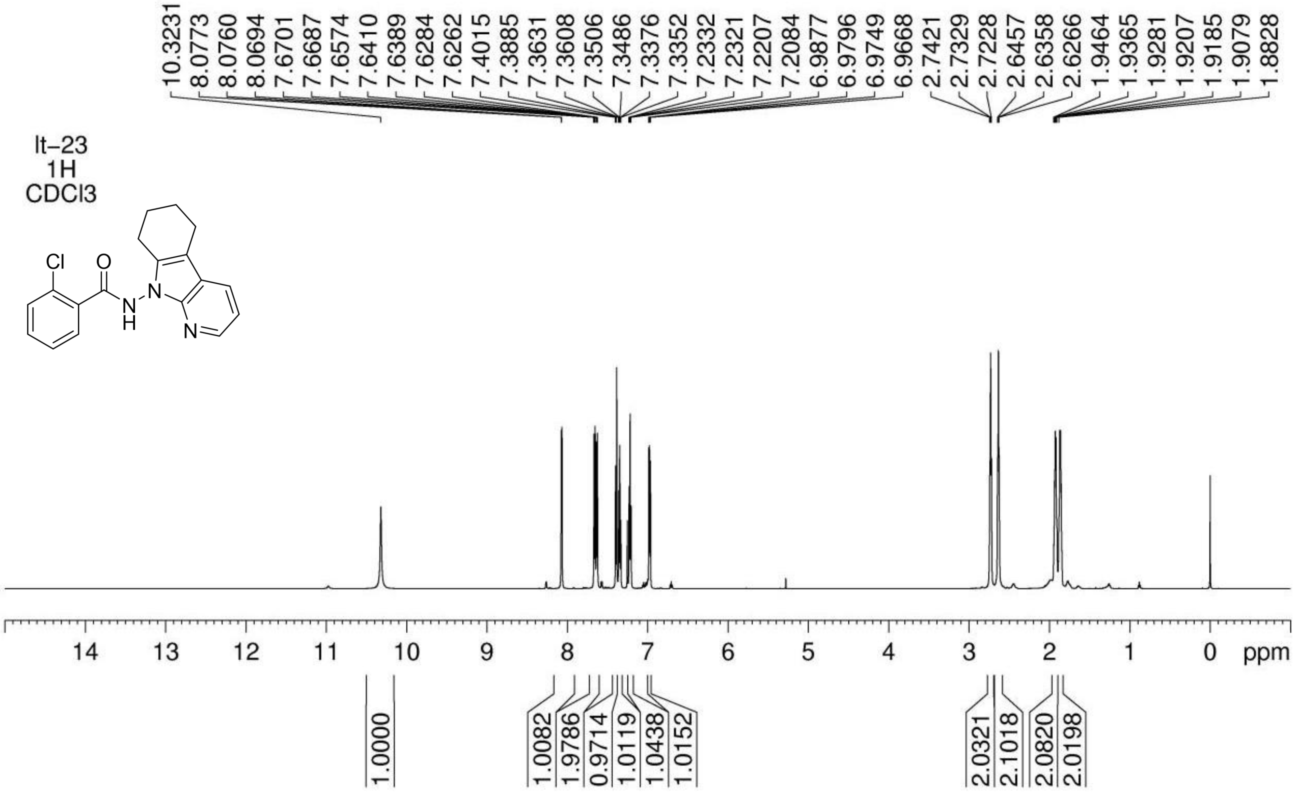
10.3231 8.0773 8.0760 8.0694 7.6701 7.6687 7.6574 7.6410 7.6389 7.6284 7.6262 7.4015 7.3885 7.3631 7.3608 7.3506 7.3486 7.3376 7.3352 7.2332 7.2321 7.2207 7.2084 6.9877 6.9796 6.9749 6.9668 2.7421 2.7329 2.7228 2.6457 2.6358 2.6266 1.9464 1.9365 1.9281 1.9207 1.9185 1.9079 1.8828
It–23
1H
CDCl3
Cl
O
N
H
N
N
14 13 12 11 10 9 8 7 6 5 4 3 2 1 0 ppm
1.0000
1.0082 1.9786 0.9714 1.0119 1.0438 1.0152
2.0321 2.1018 2.0820 2.0198

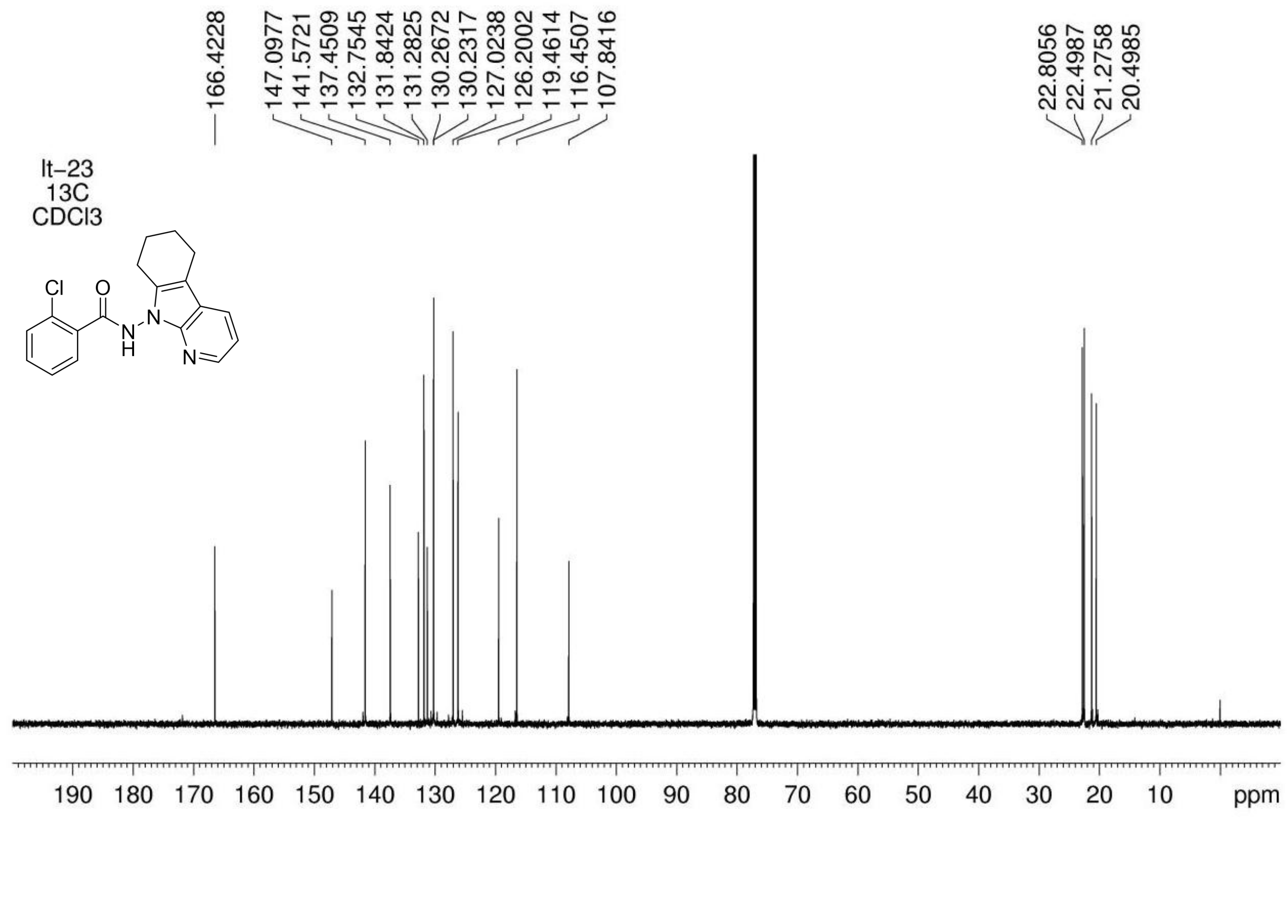
166.4228
147.0977 141.5721 137.4509 132.7545 131.8424 131.2825 130.2672 130.2317 127.0238 126.2002 119.4614 116.4507 107.8416
22.8056 22.4987 21.2758 20.4985
It–23
13C
CDCl3
Cl
O
N
H
N
N
190 180 170 160 150 140 130 120 110 100 90 80 70 60 50 40 30 20 10 ppm

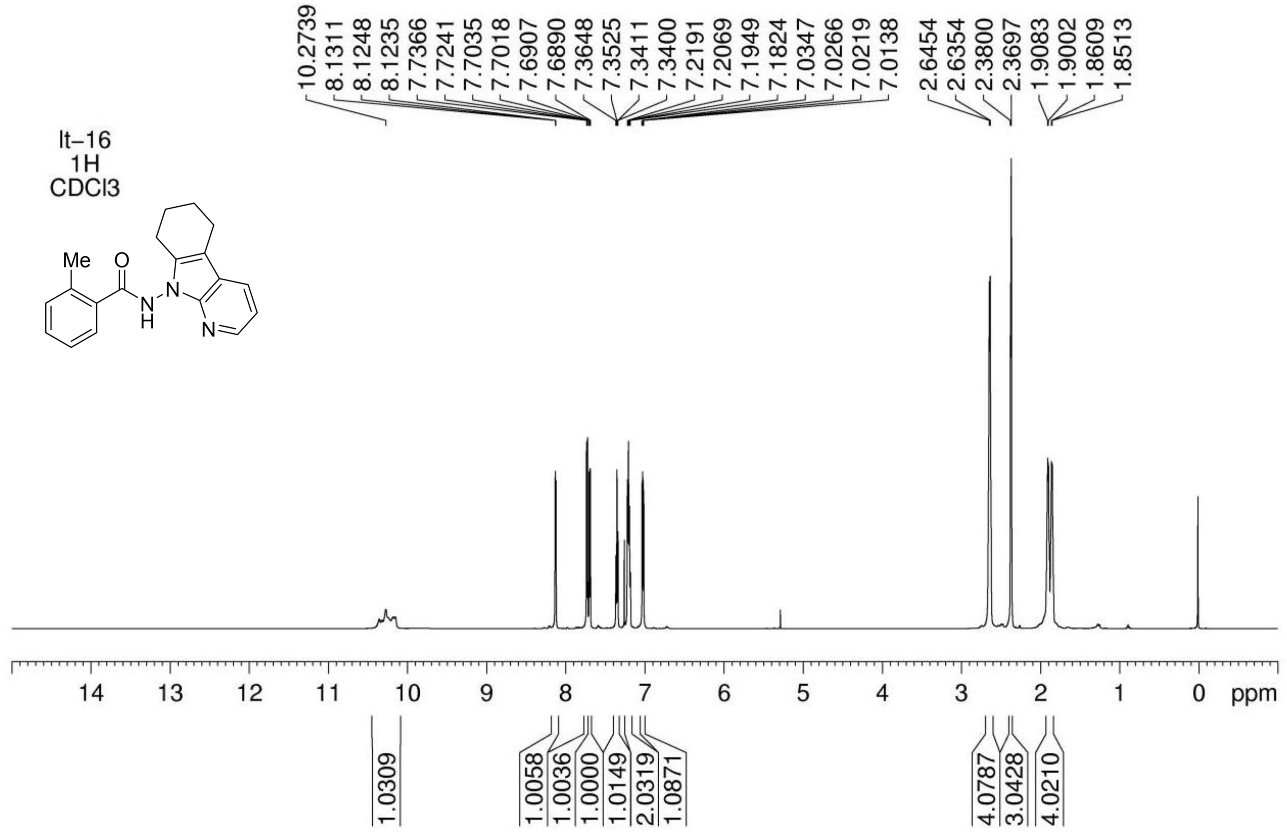

It–16
1H
CDCl3
Me
O
N
H
N
N
10.2739
8.1311
8.1248
8.1235
7.7366
7.7241
7.7035
7.7018
7.6907
7.6890
7.3648
7.3525
7.3411
7.3400
7.2191
7.2069
7.1949
7.1824
7.0347
7.0266
7.0219
7.0138
2.6454
2.6354
2.3800
2.3697
1.9083
1.9002
1.8609
1.8513
14
13
12
11
10
9
8
7
6
5
4
3
2
1
0
ppm
1.0309
1.0058
1.0036
1.0000
1.0149
2.0319
1.0871
4.0787
3.0428
4.0210

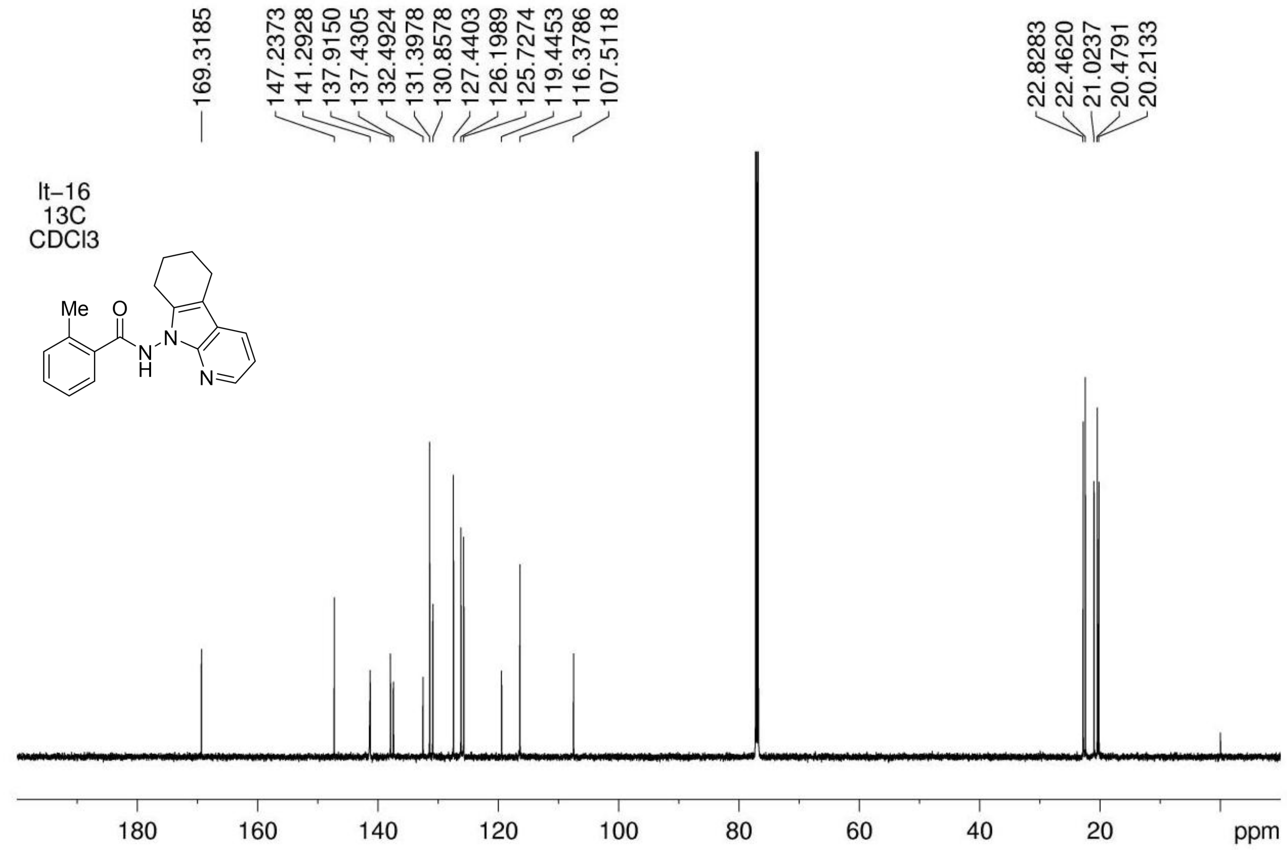

169.3185
147.2373
141.2928
137.9150
137.4305
132.4924
131.3978
130.8578
127.4403
126.1989
125.7274
119.4453
116.3786
107.5118
22.8283
22.4620
21.0237
20.4791
20.2133
It–16
13C
CDCl3
Me
O
N
H
N
N
180
160
140
120
100
80
60
40
20
ppm

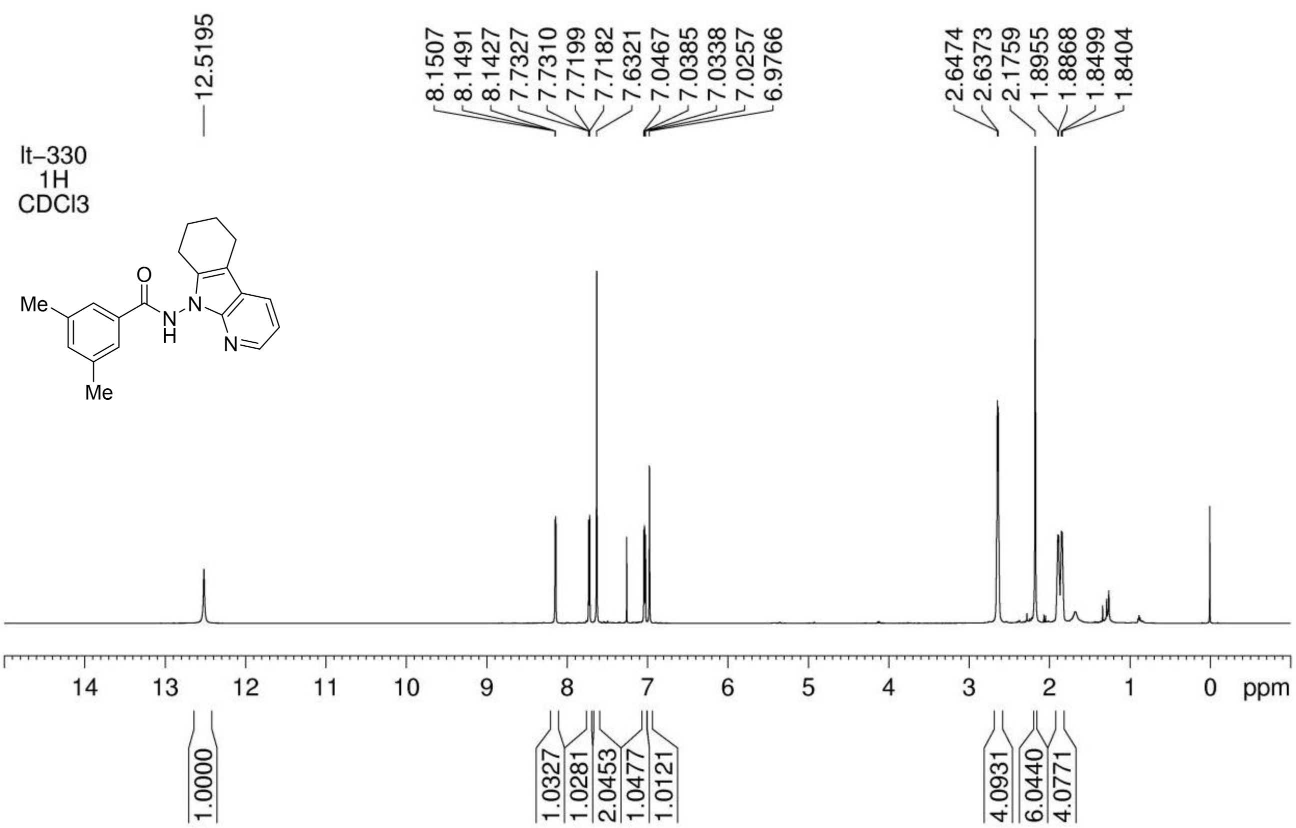
lt–330
1H
CDCl3
Me
O
N
H
N
N
Me
12.5195
8.1507
8.1491
8.1427
7.7327
7.7310
7.7199
7.7182
7.6321
7.0467
7.0385
7.0338
7.0257
6.9766
2.6474
2.6373
2.1759
1.8955
1.8868
1.8499
1.8404
14 13 12 11 10 9 8 7 6 5 4 3 2 1 0 ppm
1.0000
1.0327
1.0281
2.0453
1.0477
1.0121
4.0931
6.0440
4.0771

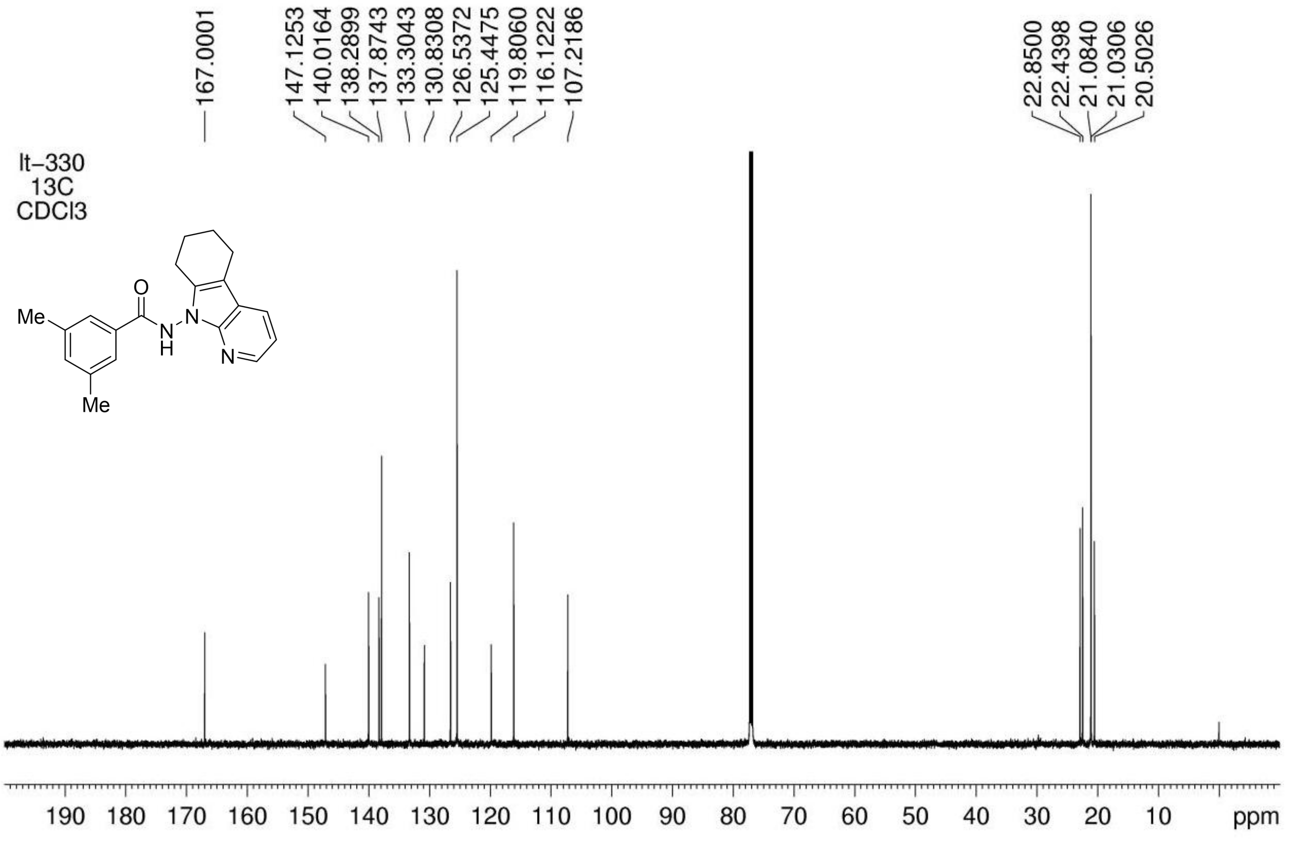
lt–330
13C
CDCl3
Me
O
N
H
N
N
Me
167.0001
147.1253
140.0164
138.2899
137.8743
133.3043
130.8308
126.5372
125.4475
119.8060
116.1222
107.2186
22.8500
22.4398
21.0840
21.0306
20.5026
190 180 170 160 150 140 130 120 110 100 90 80 70 60 50 40 30 20 10 ppm

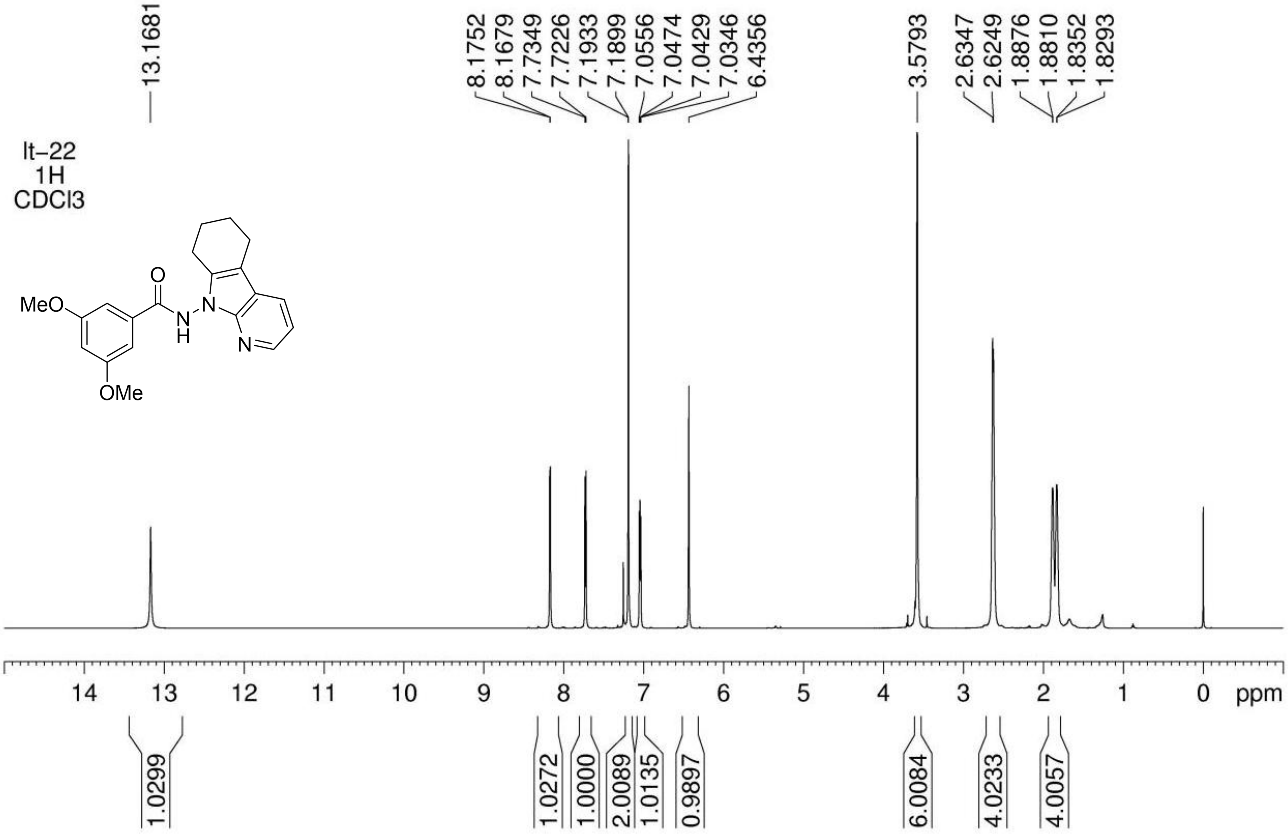

13.1681
8.1752
8.1679
7.7349
7.7226
7.1933
7.1899
7.0556
7.0474
7.0429
7.0346
6.4356
3.5793
2.6347
2.6249
1.8876
1.8810
1.8352
1.8293
It–22
1H
CDCl3
MeO
O
N
H
N
N
OMe
14 13 12 11 10 9 8 7 6 5 4 3 2 1 0 ppm
1.0299
1.0272
1.0000
2.0089
1.0135
0.9897
6.0084
4.0233
4.0057

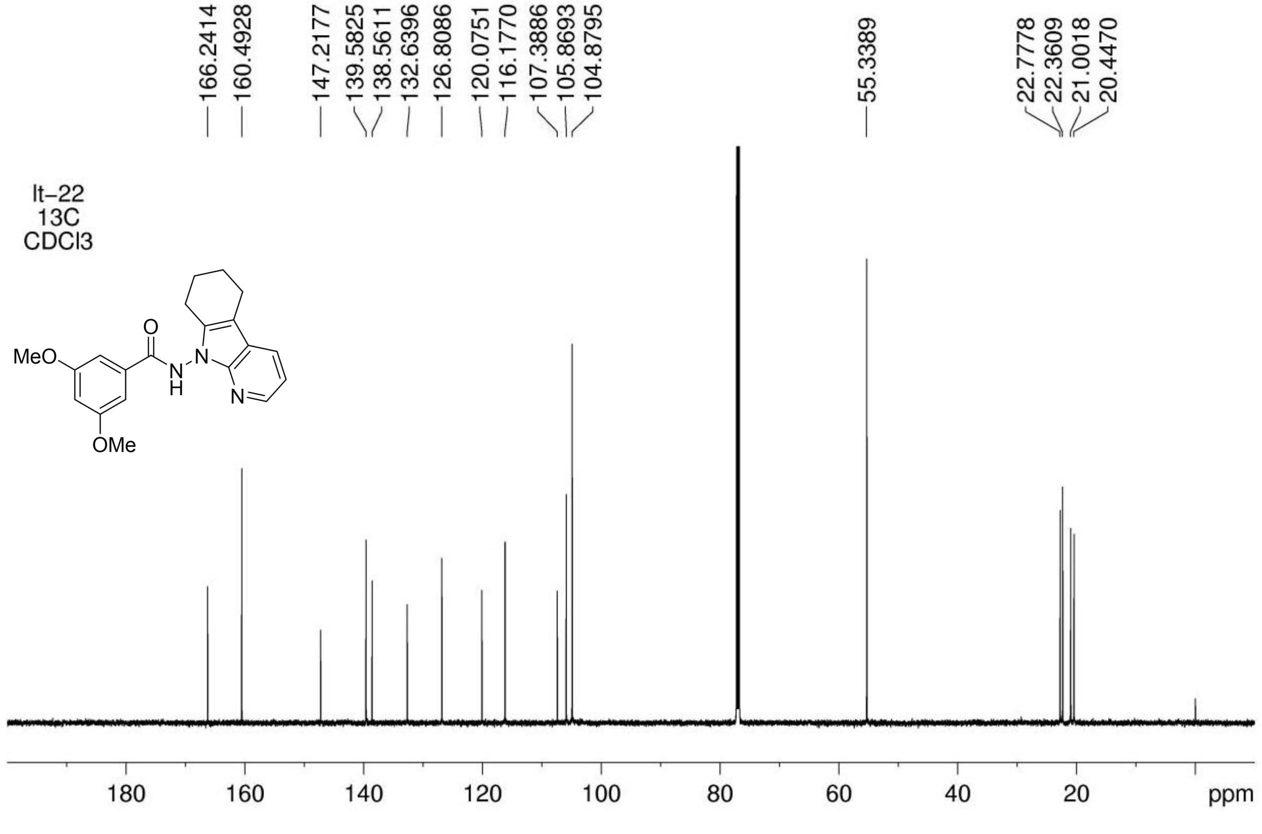

166.2414
160.4928
147.2177
139.5825
138.5611
132.6396
126.8086
120.0751
116.1770
107.3886
105.8693
104.8795
55.3389
22.7778
22.3609
21.0018
20.4470
It–22
13C
CDCl3
MeO
O
N
H
N
N
OMe
180 160 140 120 100 80 60 40 20 ppm

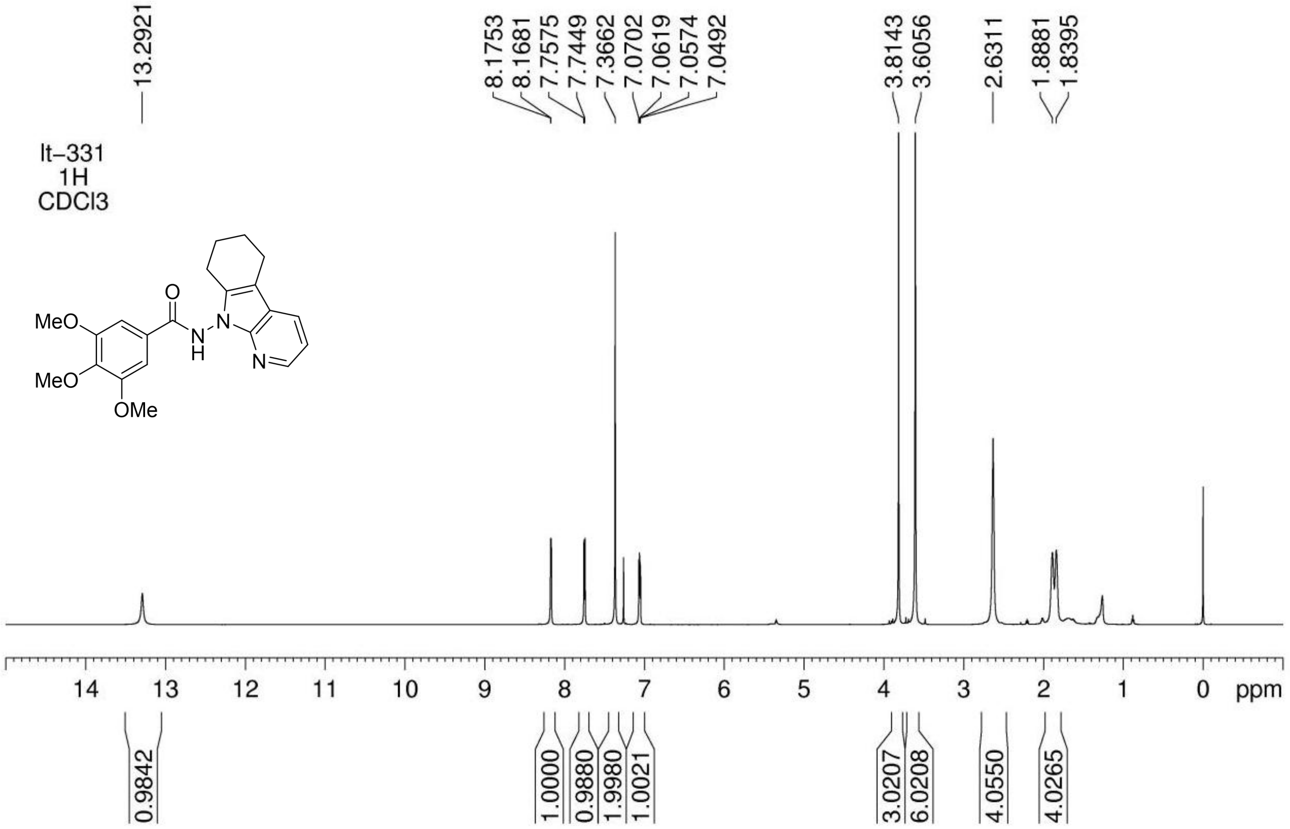
lt–331
1H
CDCl3
MeO
MeO
OMe
O
N
H
N
N
13.2921
8.1753
8.1681
7.7575
7.7449
7.3662
7.0702
7.0619
7.0574
7.0492
3.8143
3.6056
2.6311
1.8881
1.8395
14 13 12 11 10 9 8 7 6 5 4 3 2 1 0 ppm
0.9842
1.0000
0.9880
1.9980
1.0021
3.0207
6.0208
4.0550
4.0265

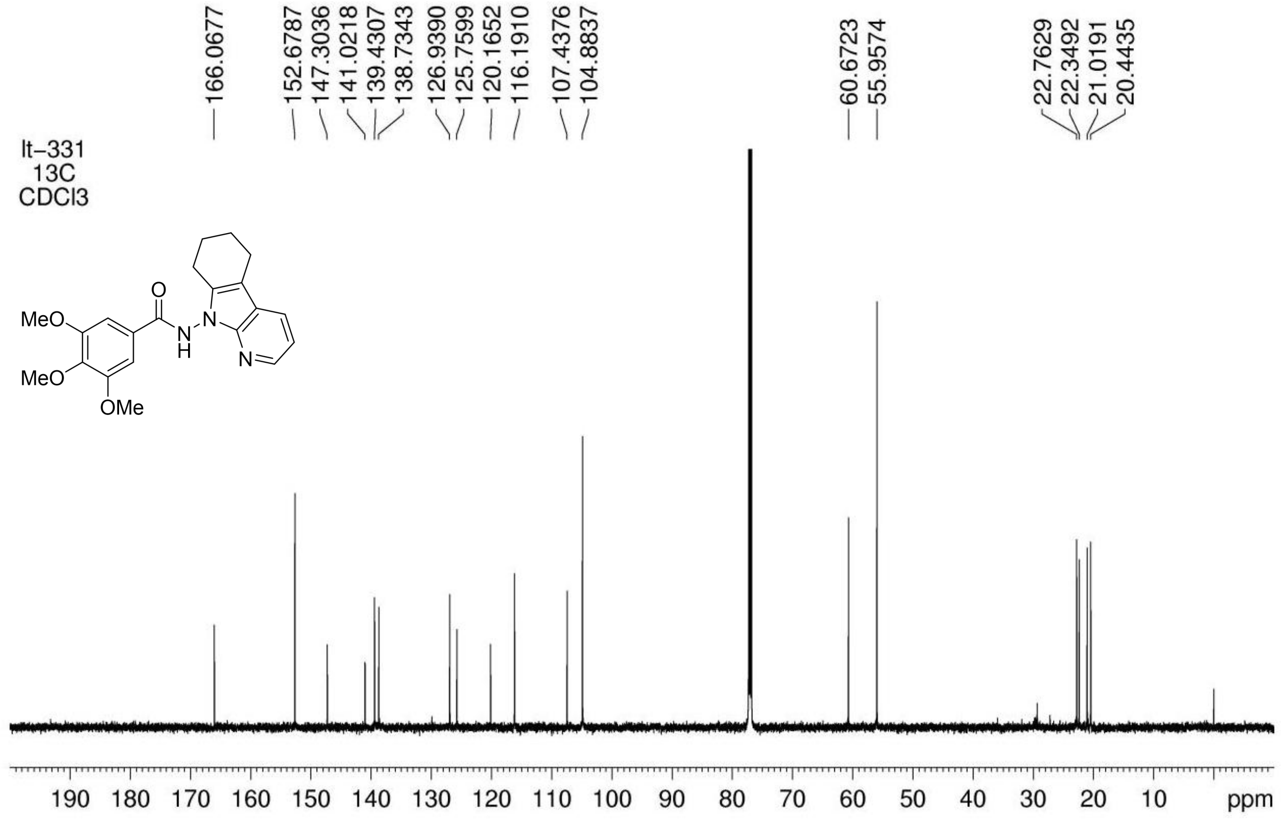
lt–331
13C
CDCl3
MeO
MeO
OMe
O
N
H
N
N
166.0677
152.6787
147.3036
141.0218
139.4307
138.7343
126.9390
125.7599
120.1652
116.1910
107.4376
104.8837
60.6723
55.9574
22.7629
22.3492
21.0191
20.4435
190 180 170 160 150 140 130 120 110 100 90 80 70 60 50 40 30 20 10 ppm

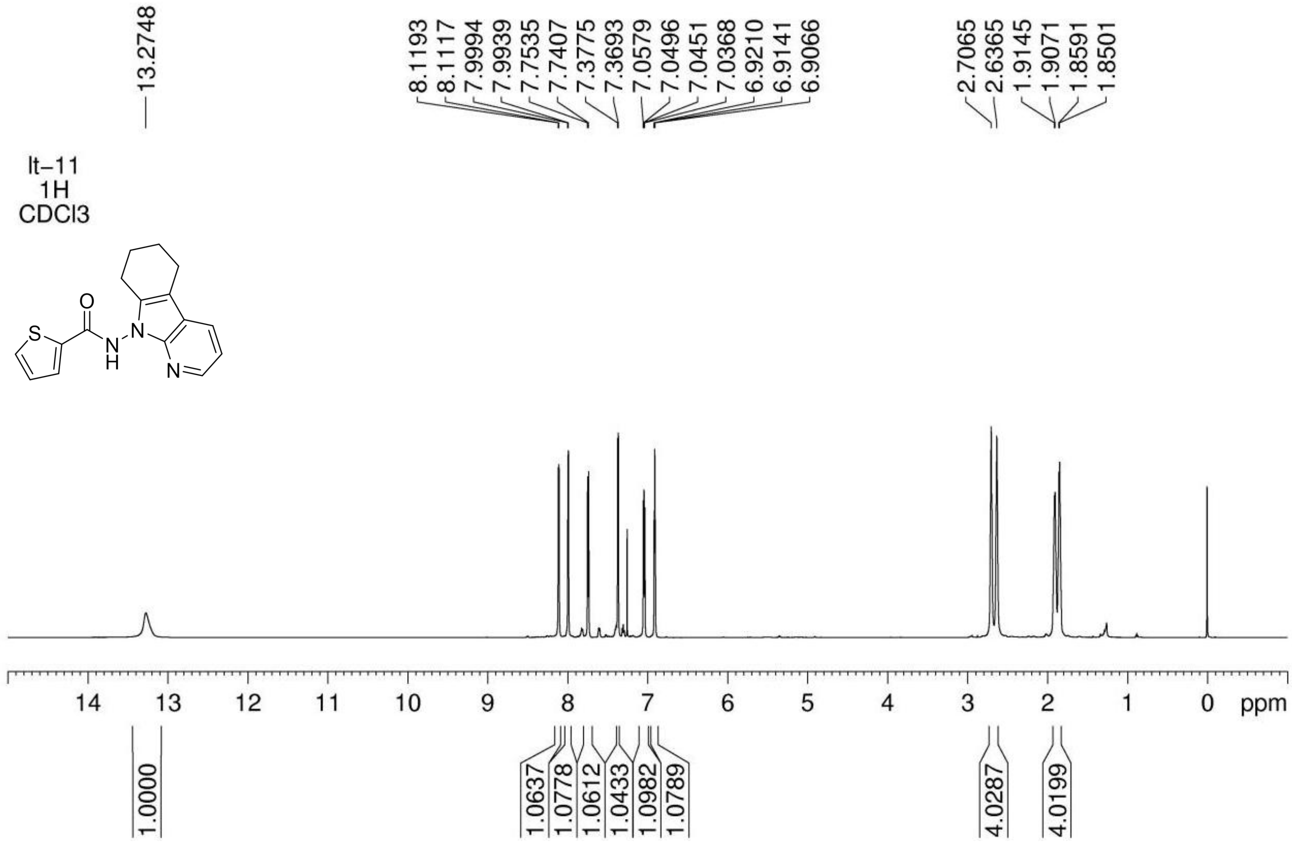
lt–11
1H
CDCl3
13.2748
8.1193
8.1117
7.9994
7.9939
7.7535
7.7407
7.3775
7.3693
7.0579
7.0496
7.0451
7.0368
6.9210
6.9141
6.9066
2.7065
2.6365
1.9145
1.9071
1.8591
1.8501
14 13 12 11 10 9 8 7 6 5 4 3 2 1 0 ppm
1.0000
1.0637
1.0778
1.0612
1.0433
1.0982
1.0789
4.0287
4.0199

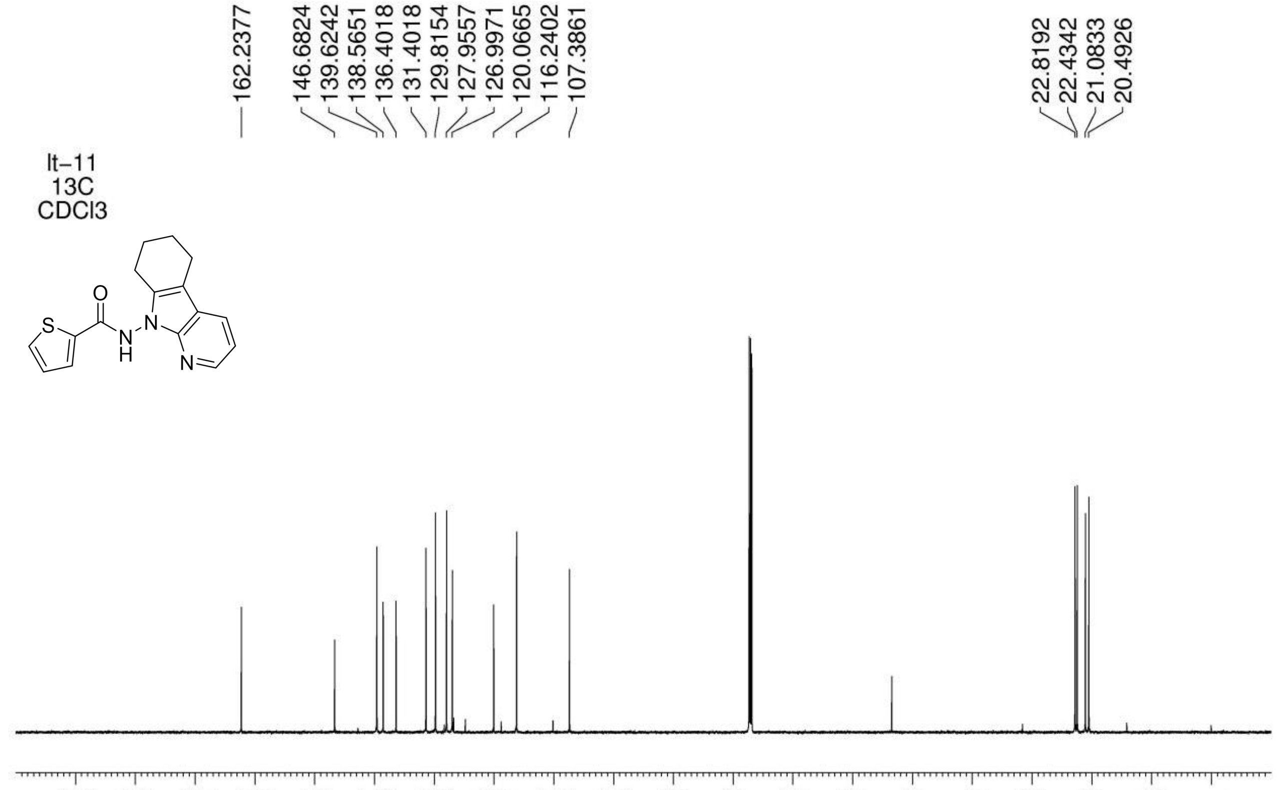
lt–11
13C
CDCl3
162.2377
146.6824
139.6242
138.5651
136.4018
131.4018
129.8154
127.9557
126.9971
120.0665
116.2402
107.3861
22.8192
22.4342
21.0833
20.4926
190 180 170 160 150 140 130 120 110 100 90 80 70 60 50 40 30 20 10 ppm

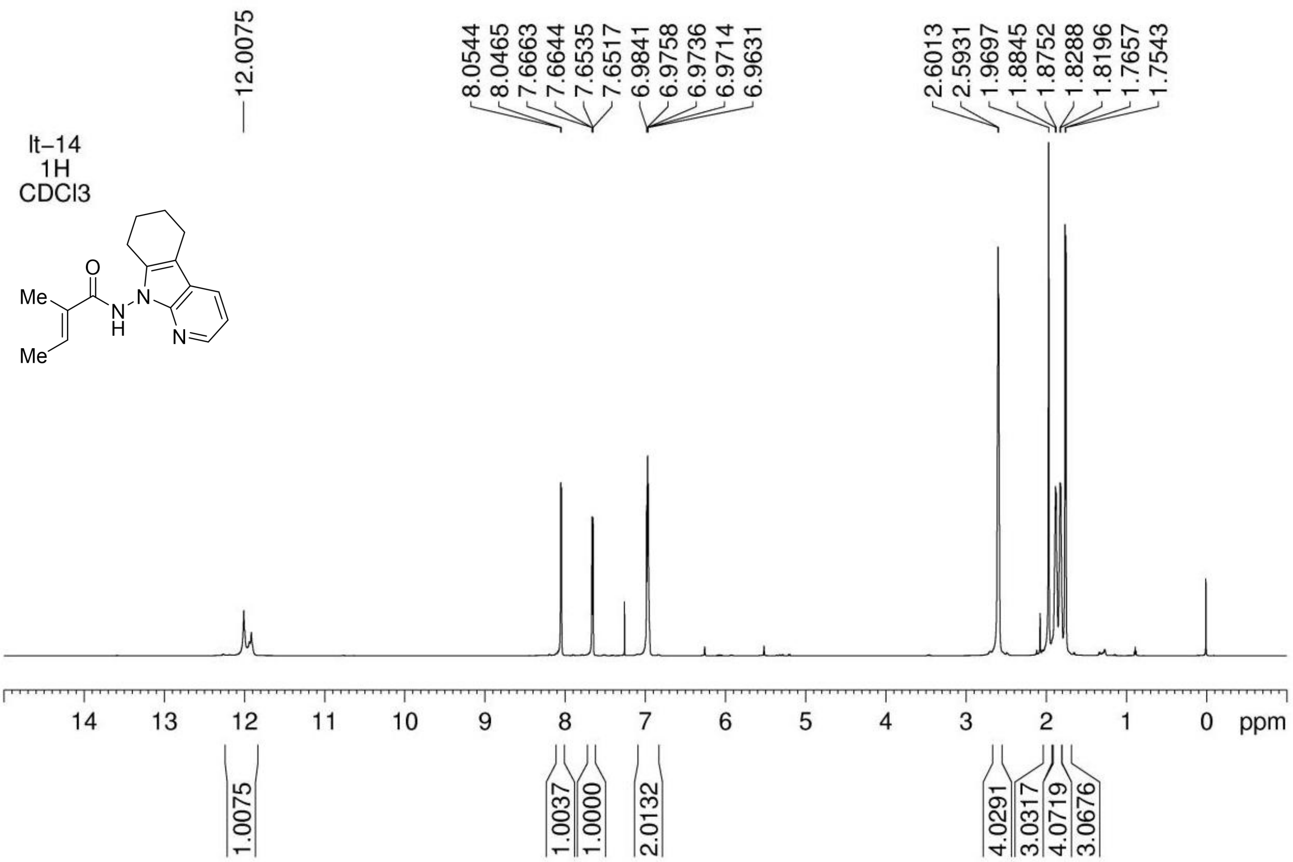
It–14
1H
CDCl3
Me
O
N
H
N
N
Me
12.0075
8.0544
8.0465
7.6663
7.6644
7.6535
7.6517
6.9841
6.9758
6.9736
6.9714
6.9631
2.6013
2.5931
1.9697
1.8845
1.8752
1.8288
1.8196
1.7657
1.7543
14 13 12 11 10 9 8 7 6 5 4 3 2 1 0 ppm
1.0075
1.0037
1.0000
2.0132
4.0291
3.0317
4.0719
3.0676

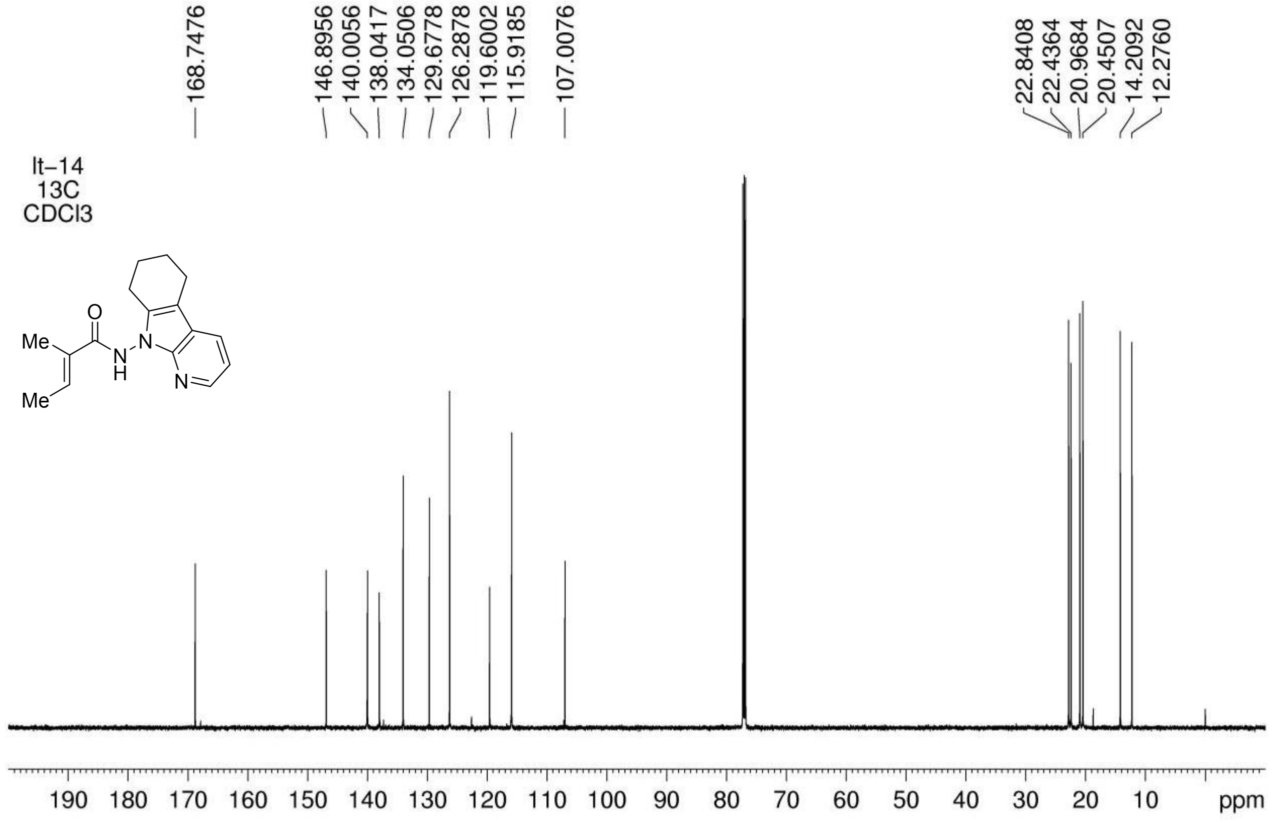
It–14
13C
CDCl3
Me
O
N
H
N
N
Me
168.7476
146.8956
140.0056
138.0417
134.0506
129.6778
126.2878
119.6002
115.9185
107.0076
22.8408
22.4364
20.9684
20.4507
14.2092
12.2760
190 180 170 160 150 140 130 120 110 100 90 80 70 60 50 40 30 20 10 ppm

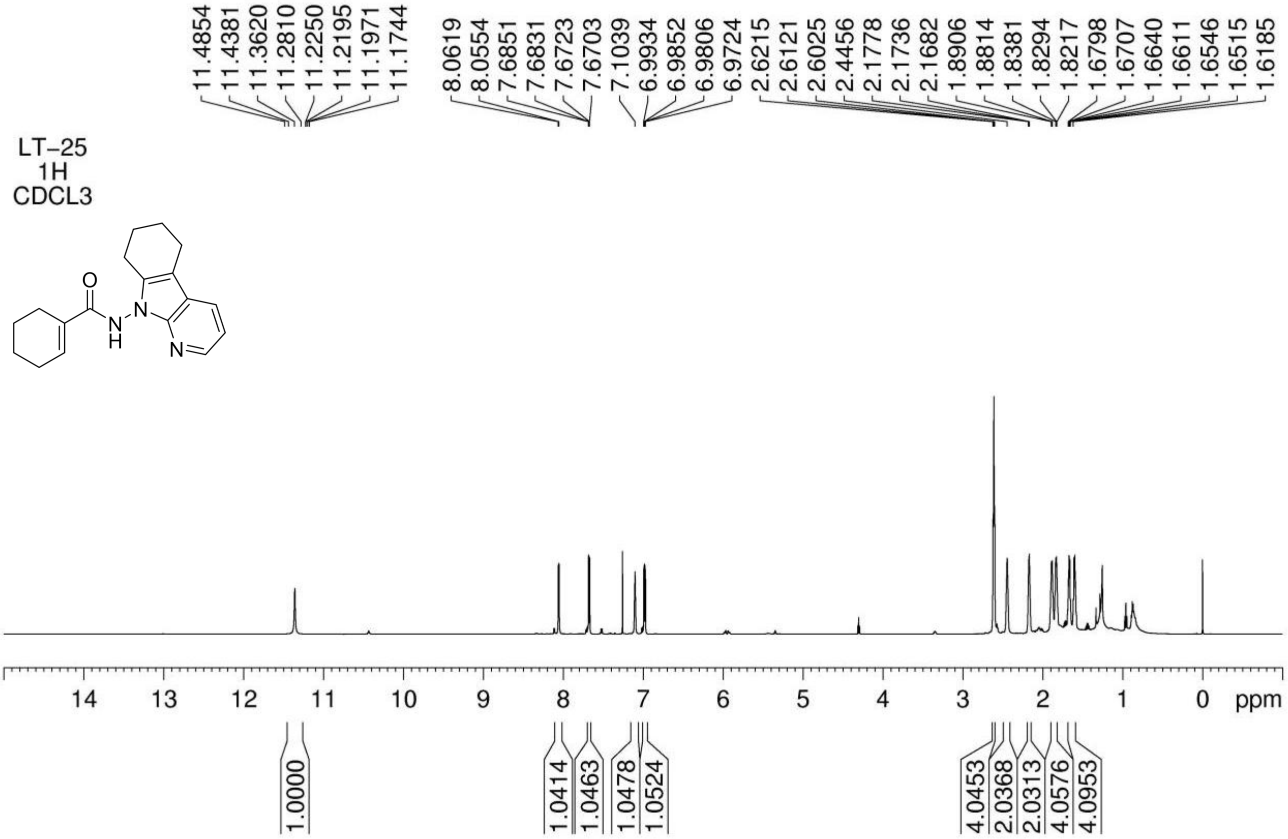
11.4854
11.4381
11.3620
11.2810
11.2250
11.2195
11.1971
11.1744
8.0619
8.0554
7.6851
7.6831
7.6723
7.6703
7.1039
6.9934
6.9852
6.9806
6.9724
2.6215
2.6121
2.6025
2.4456
2.1778
2.1736
2.1682
1.8906
1.8814
1.8381
1.8294
1.8217
1.6798
1.6707
1.6640
1.6611
1.6546
1.6515
1.6185
LT−25
1H
CDCL3
O
N
H
N
N
14 13 12 11 10 9 8 7 6 5 4 3 2 1 0 ppm
1.0000
1.0414
1.0463
1.0478
1.0524
4.0453
2.0368
2.0313
4.0576
4.0953

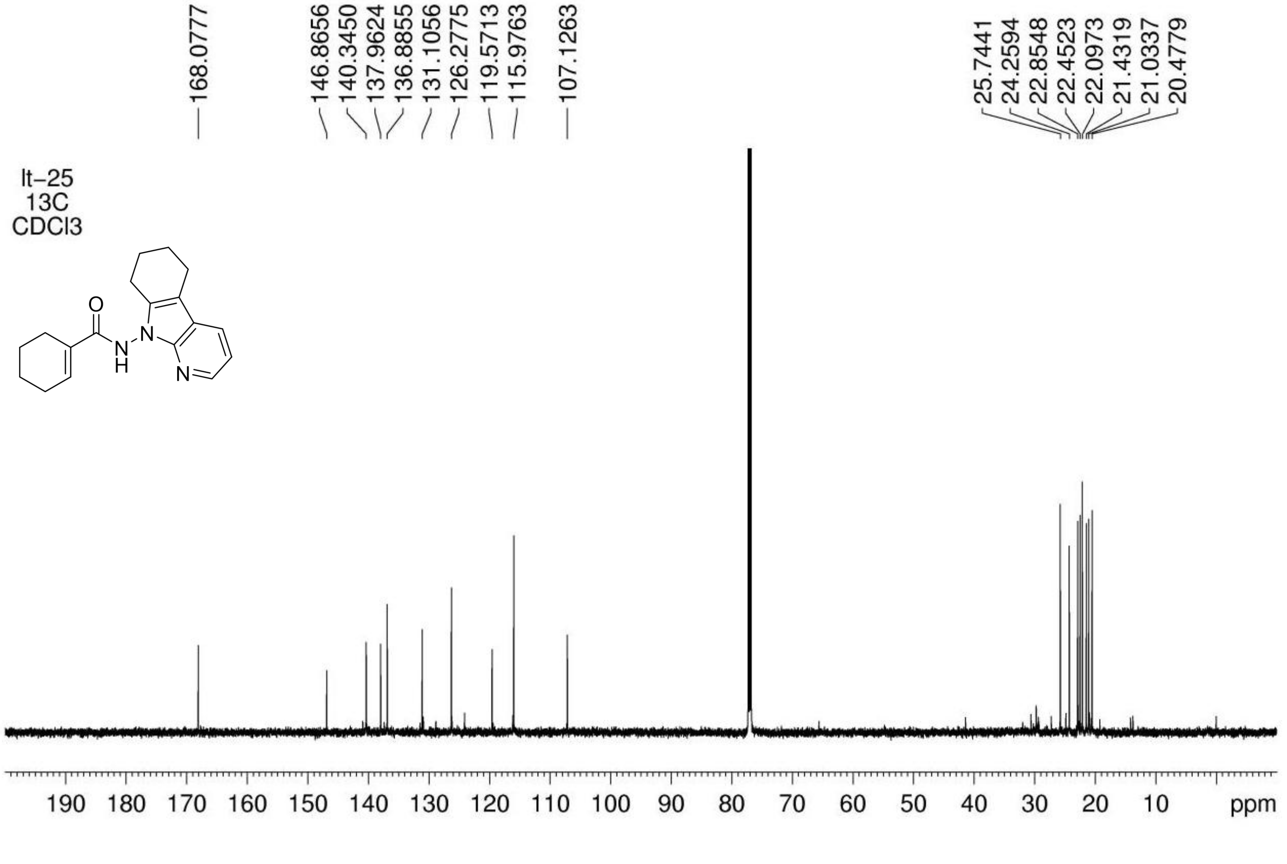
168.0777
146.8656
140.3450
137.9624
136.8855
131.1056
126.2775
119.5713
115.9763
107.1263
25.7441
24.2594
22.8548
22.4523
22.0973
21.4319
21.0337
20.4779
lt−25
13C
CDCl3
O
N
H
N
N
190 180 170 160 150 140 130 120 110 100 90 80 70 60 50 40 30 20 10 ppm

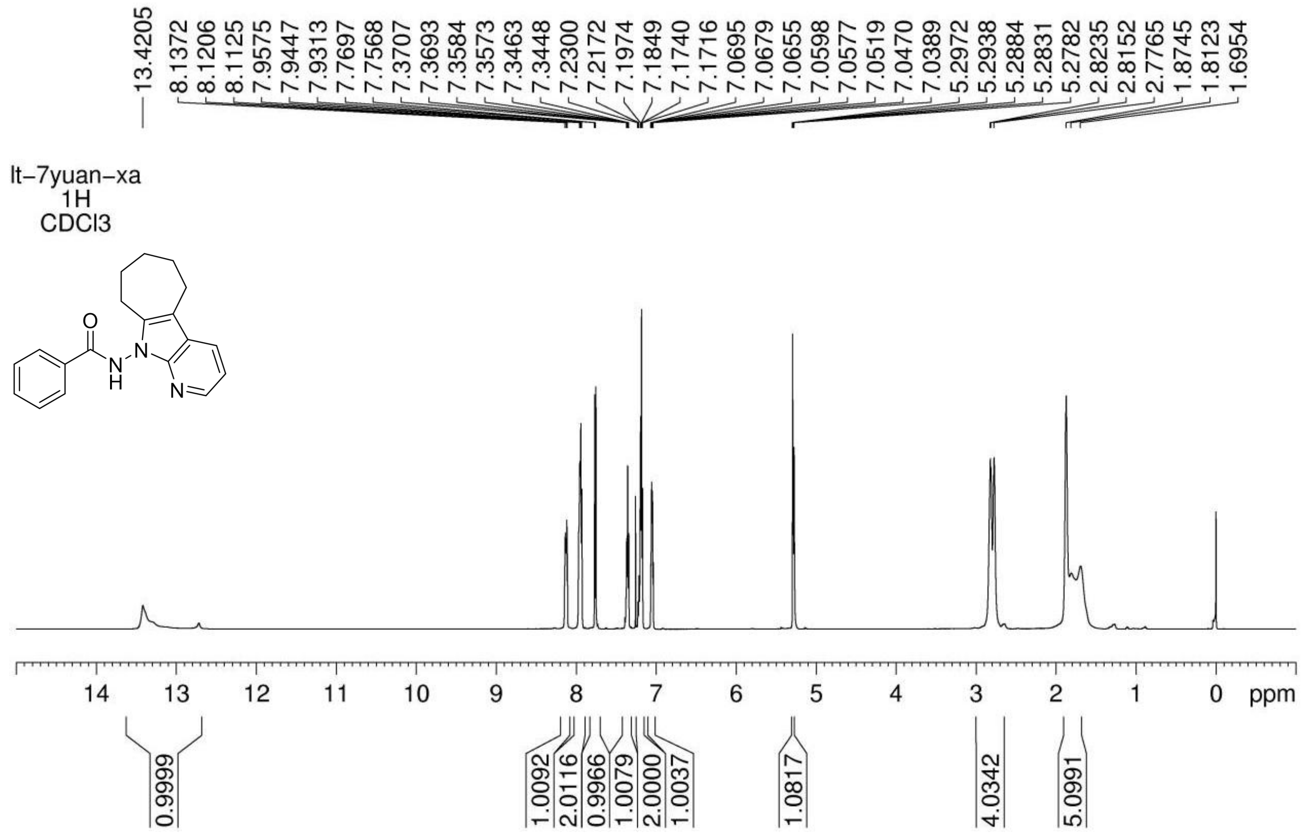
13.4205
8.1372
8.1206
8.1125
7.9575
7.9447
7.9313
7.7697
7.7568
7.3707
7.3693
7.3584
7.3573
7.3463
7.3448
7.2300
7.2172
7.1974
7.1849
7.1740
7.1716
7.0695
7.0679
7.0655
7.0598
7.0577
7.0519
7.0470
7.0389
5.2972
5.2938
5.2884
5.2831
5.2782
2.8235
2.8152
2.7765
1.8745
1.8123
1.6954
lt−7yuan−xa
1H
CDCl3
O
N
H
N
N
14 13 12 11 10 9 8 7 6 5 4 3 2 1 0 ppm
0.9999
1.0092
2.0116
0.9966
1.0079
2.0000
1.0037
1.0817
4.0342
5.0991

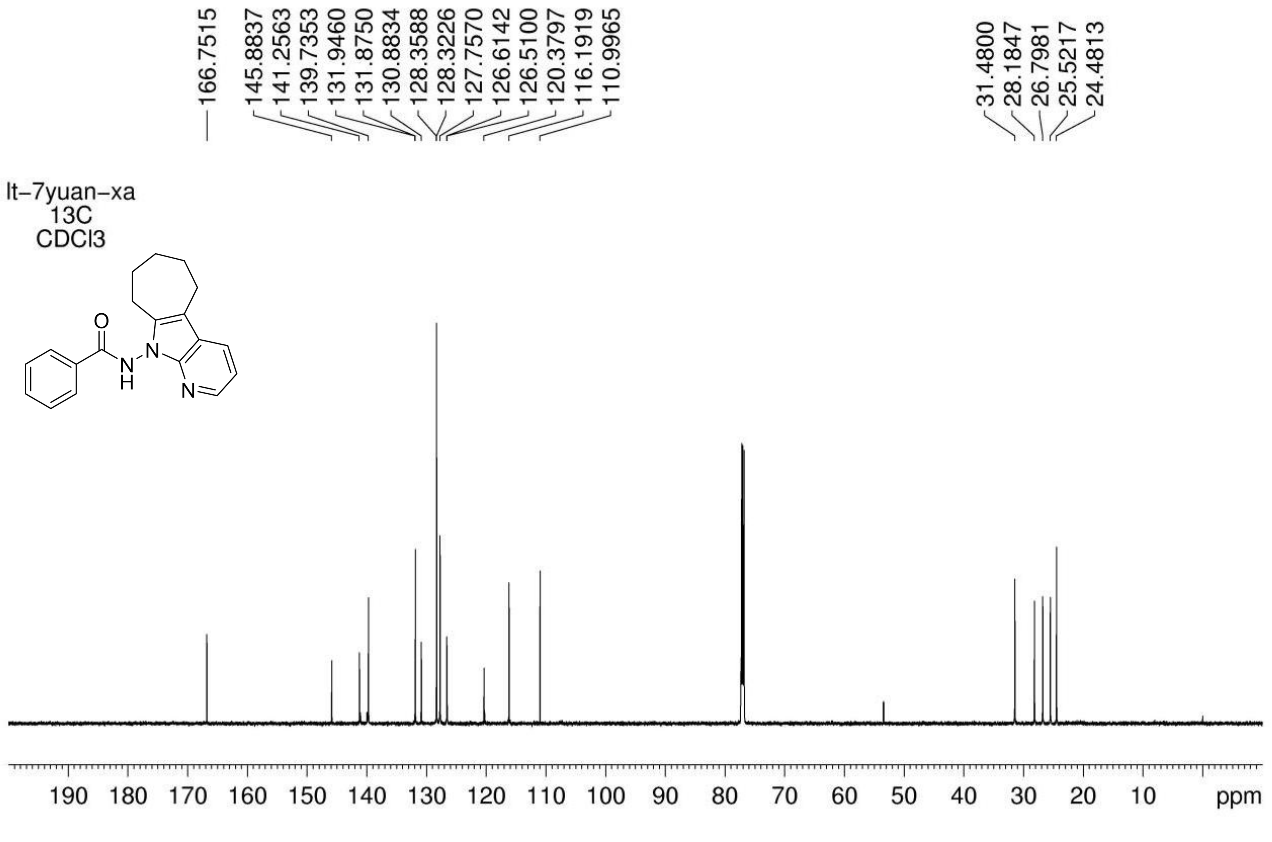
166.7515
145.8837
141.2563
139.7353
131.9460
131.8750
130.8834
128.3588
128.3226
127.7570
126.6142
126.5100
120.3797
116.1919
110.9965
31.4800
28.1847
26.7981
25.5217
24.4813
lt−7yuan−xa
13C
CDCl3
O
N
H
N
N
190 180 170 160 150 140 130 120 110 100 90 80 70 60 50 40 30 20 10 ppm

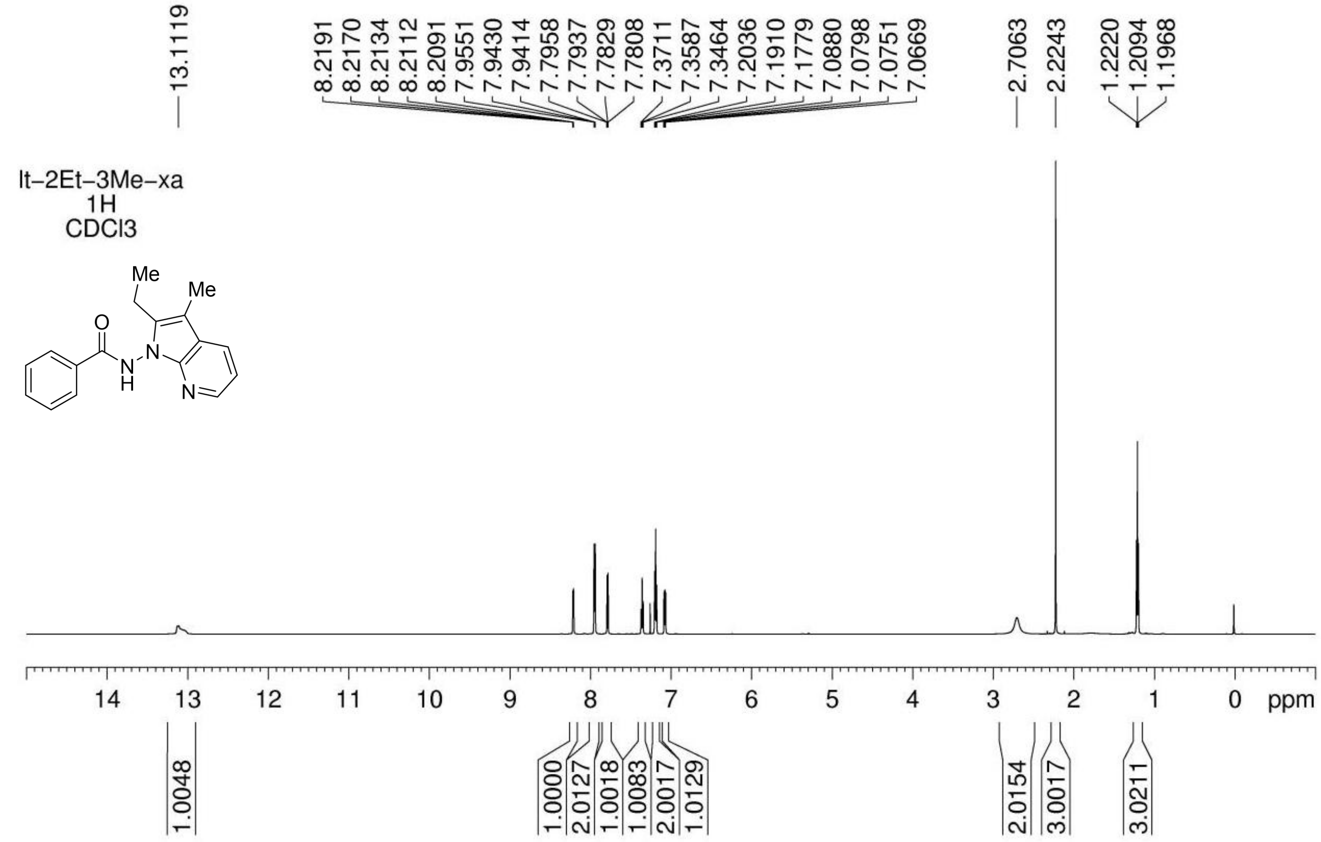
It–2Et–3Me–xa
1H
CDCl3
Me
Me
O
N
N
H
N
13.1119
8.2191
8.2170
8.2134
8.2112
8.2091
7.9551
7.9430
7.9414
7.7958
7.7937
7.7829
7.7808
7.3711
7.3587
7.3464
7.2036
7.1910
7.1779
7.0880
7.0798
7.0751
7.0669
2.7063
2.2243
1.2220
1.2094
1.1968
14
13
12
11
10
9
8
7
6
5
4
3
2
1
0
ppm
1.0048
1.0000
2.0127
1.0018
1.0083
2.0017
1.0129
2.0154
3.0017
3.0211

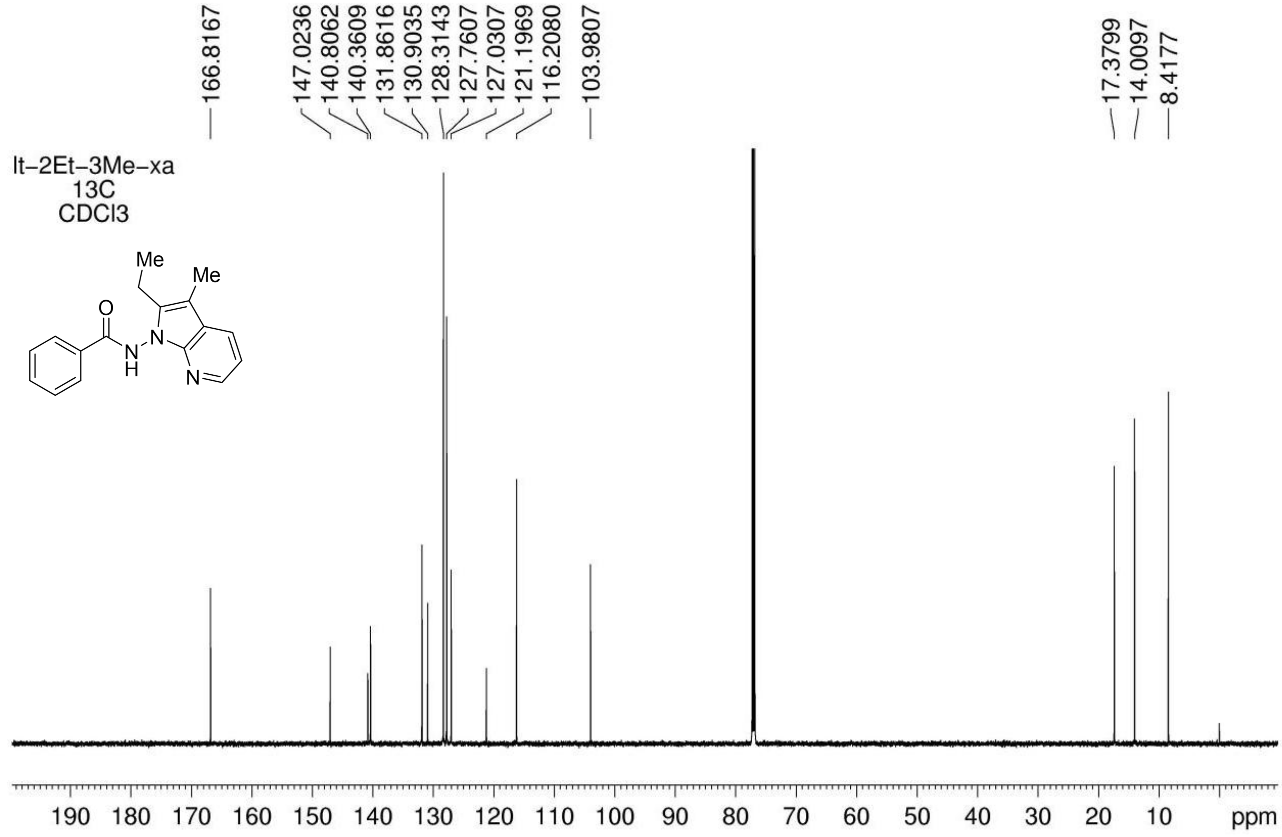
It–2Et–3Me–xa
13C
CDCl3
Me
Me
O
N
N
H
N
166.8167
147.0236
140.8062
140.3609
131.8616
130.9035
128.3143
127.7607
127.0307
121.1969
116.2080
103.9807
17.3799
14.0097
8.4177
190
180
170
160
150
140
130
120
110
100
90
80
70
60
50
40
30
20
10
ppm

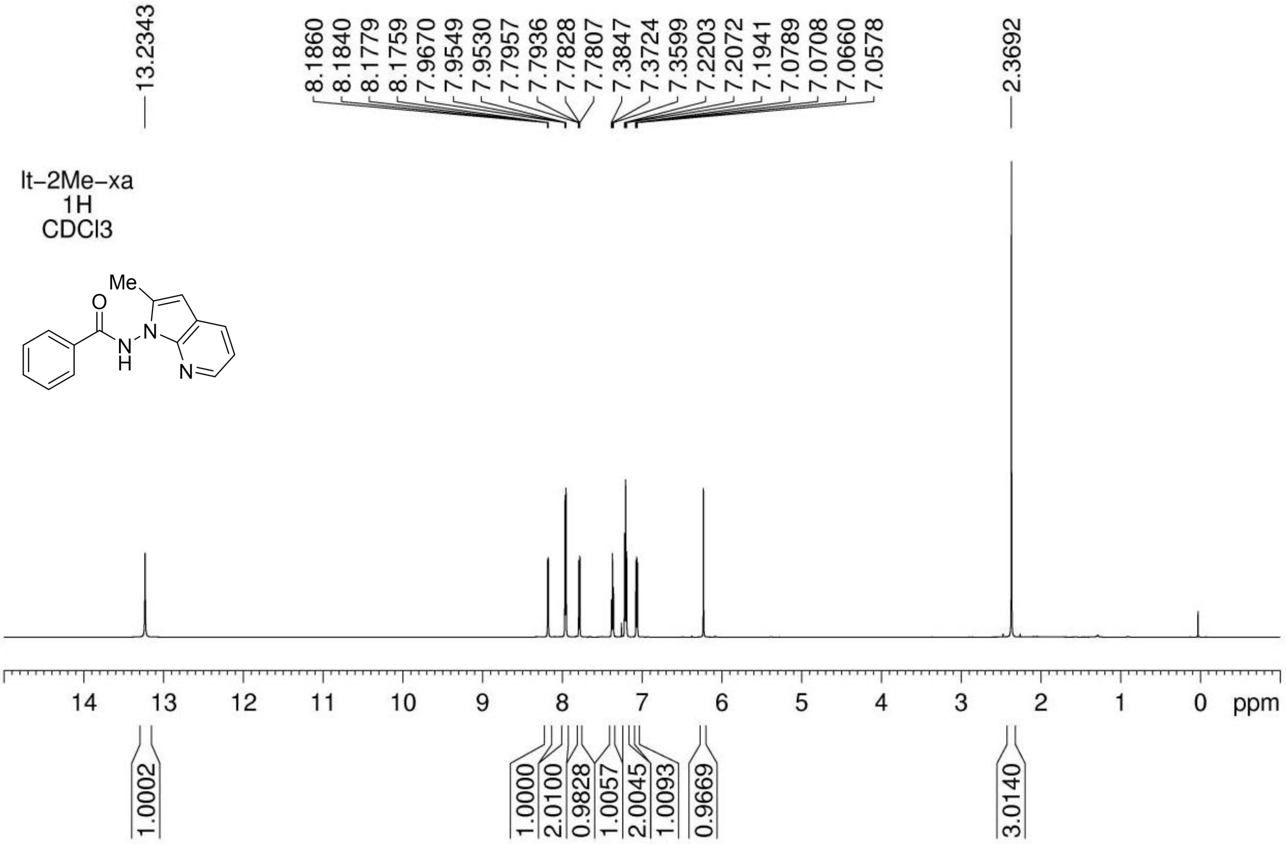
It−2Me−xa
1H
CDCl3
Me
O
N
H
N
N
13.2343
8.1860
8.1840
8.1779
8.1759
7.9670
7.9549
7.9530
7.7957
7.7936
7.7828
7.7807
7.3847
7.3724
7.3599
7.2203
7.2072
7.1941
7.0789
7.0708
7.0660
7.0578
2.3692
14 13 12 11 10 9 8 7 6 5 4 3 2 1 0 ppm
1.0002
1.0000
2.0100
0.9828
1.0057
2.0045
1.0093
0.9669
3.0140

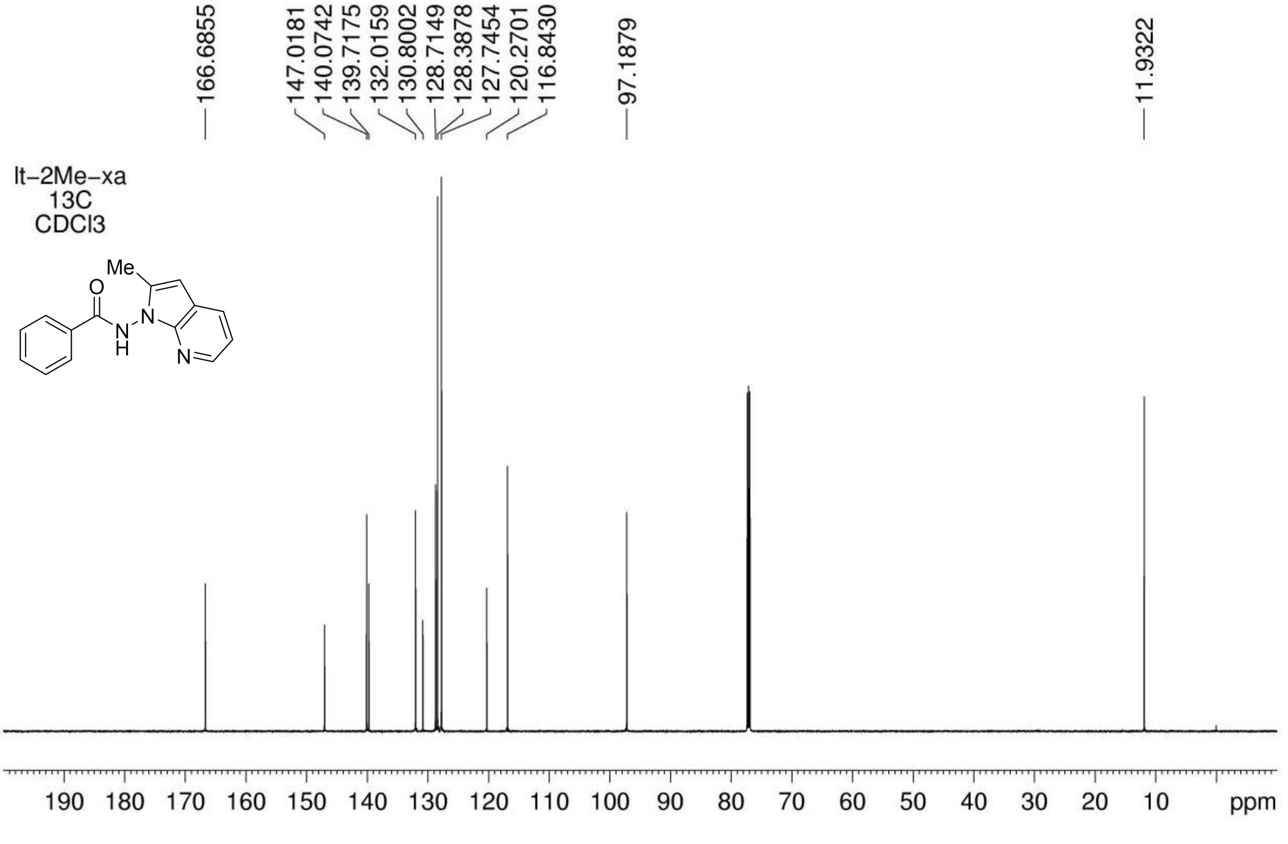
It−2Me−xa
13C
CDCl3
Me
O
N
H
N
N
166.6855
147.0181
140.0742
139.7175
132.0159
130.8002
128.7149
128.3878
127.7454
120.2701
116.8430
97.1879
11.9322
190 180 170 160 150 140 130 120 110 100 90 80 70 60 50 40 30 20 10 ppm

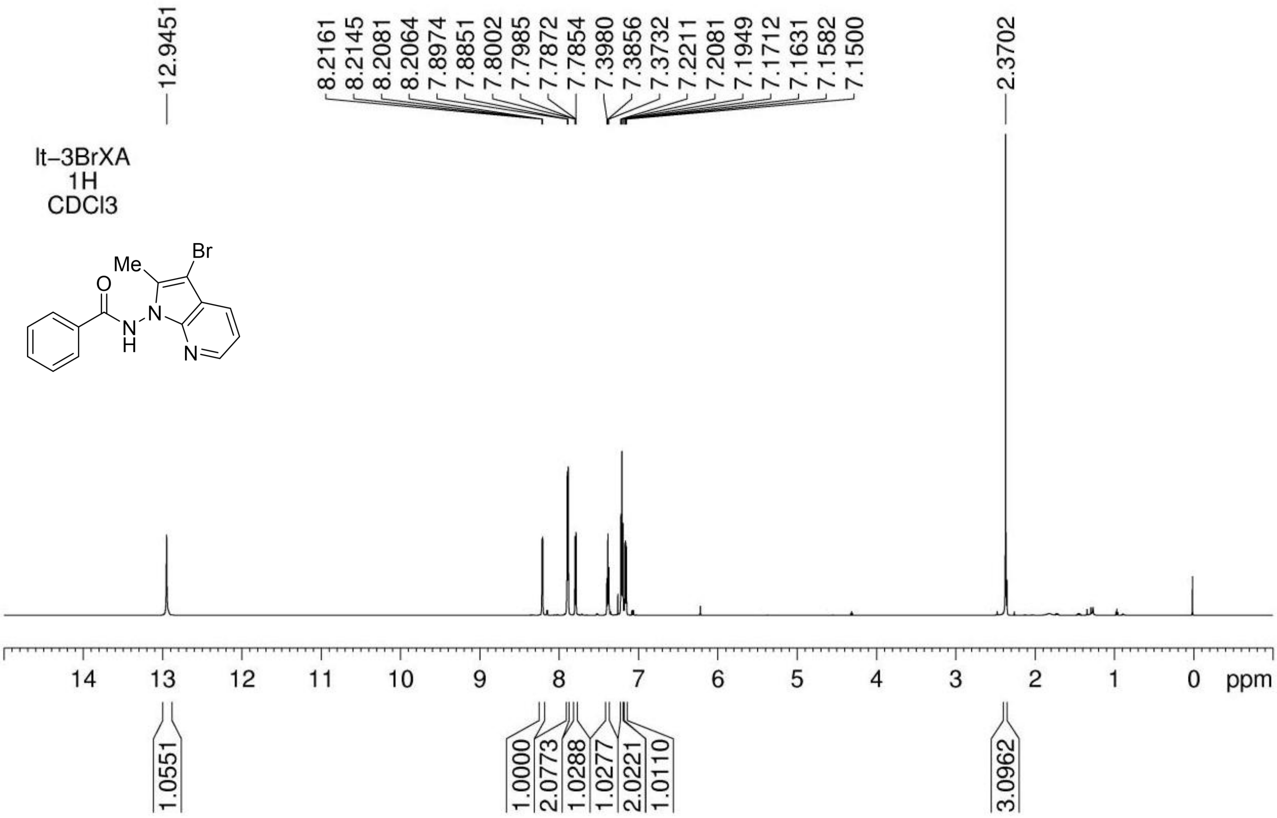
It−3BrXA
1H
CDCl3
Me
Br
O
N
H
N
N
12.9451
8.2161
8.2145
8.2081
8.2064
7.8974
7.8851
7.8002
7.7985
7.7872
7.7854
7.3980
7.3856
7.3732
7.2211
7.2081
7.1949
7.1712
7.1631
7.1582
7.1500
2.3702
14 13 12 11 10 9 8 7 6 5 4 3 2 1 0 ppm
1.0551
1.0000
2.0773
1.0288
1.0277
2.0221
1.0110
3.0962

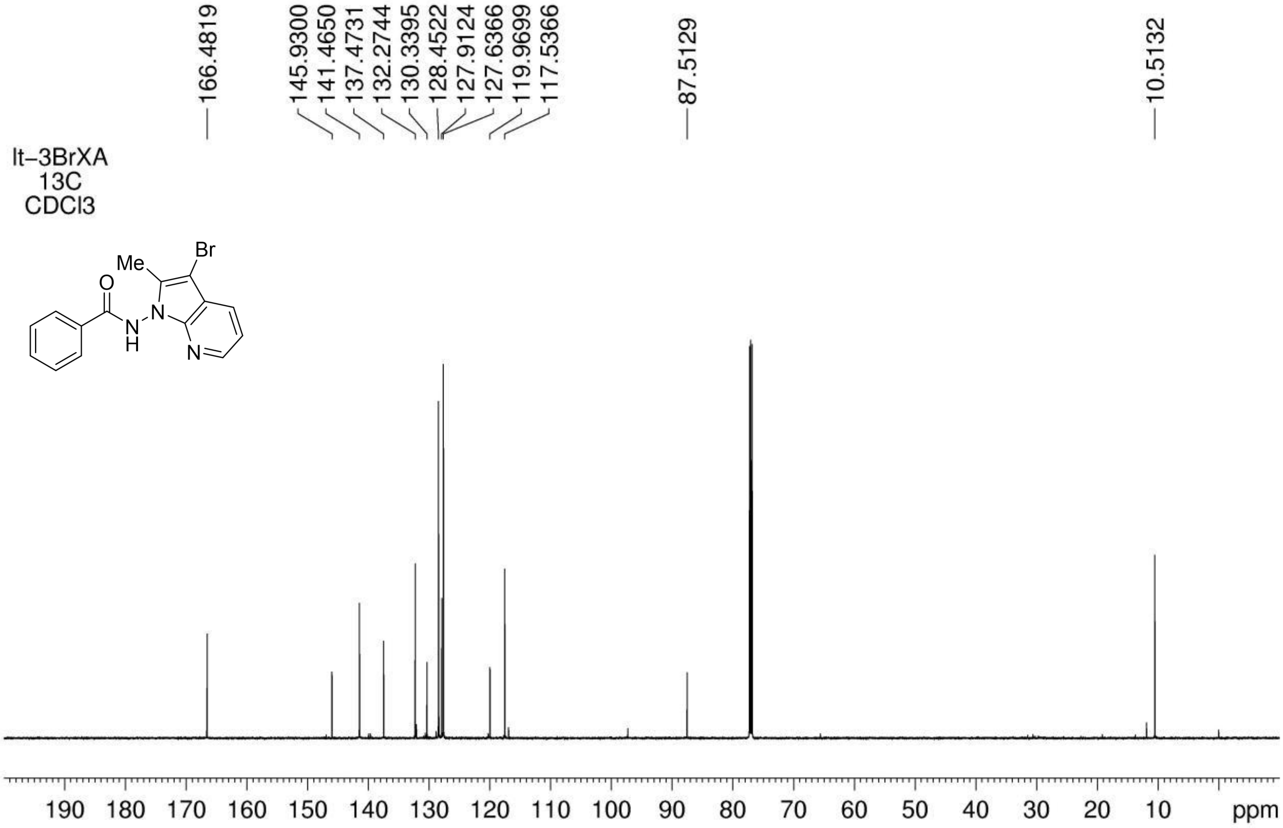
It−3BrXA
13C
CDCl3
Me
Br
O
N
H
N
N
166.4819
145.9300
141.4650
137.4731
132.2744
130.3395
128.4522
127.9124
127.6366
119.9699
117.5366
87.5129
10.5132
190 180 170 160 150 140 130 120 110 100 90 80 70 60 50 40 30 20 10 ppm

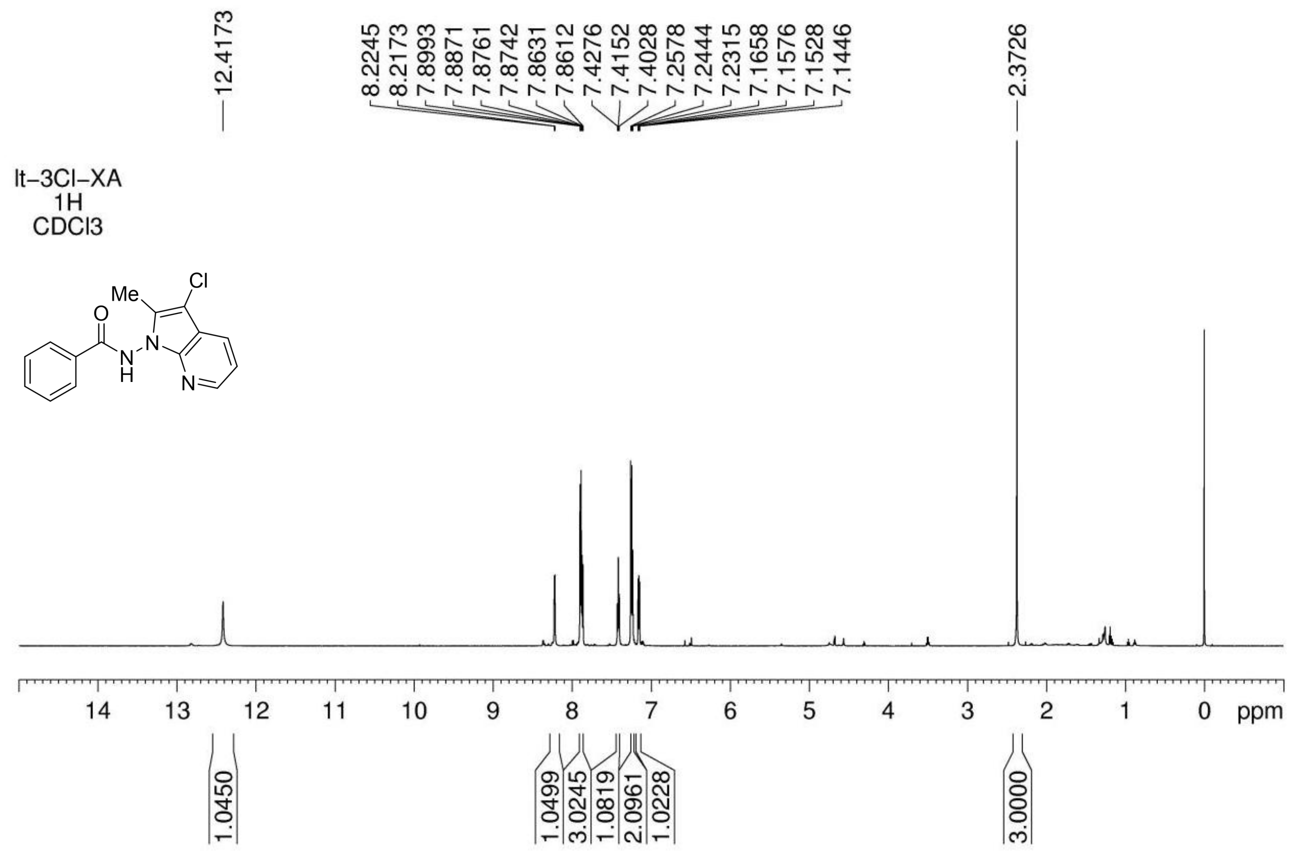
12.4173
8.2245
8.2173
7.8993
7.8871
7.8761
7.8742
7.8631
7.8612
7.4276
7.4152
7.4028
7.2578
7.2444
7.2315
7.1658
7.1576
7.1528
7.1446
2.3726
lt–3Cl–XA
1H
CDCl3
Me
Cl
O
N
N
H
N
14 13 12 11 10 9 8 7 6 5 4 3 2 1 0 ppm
1.0450
1.0499
3.0245
1.0819
2.0961
1.0228
3.0000

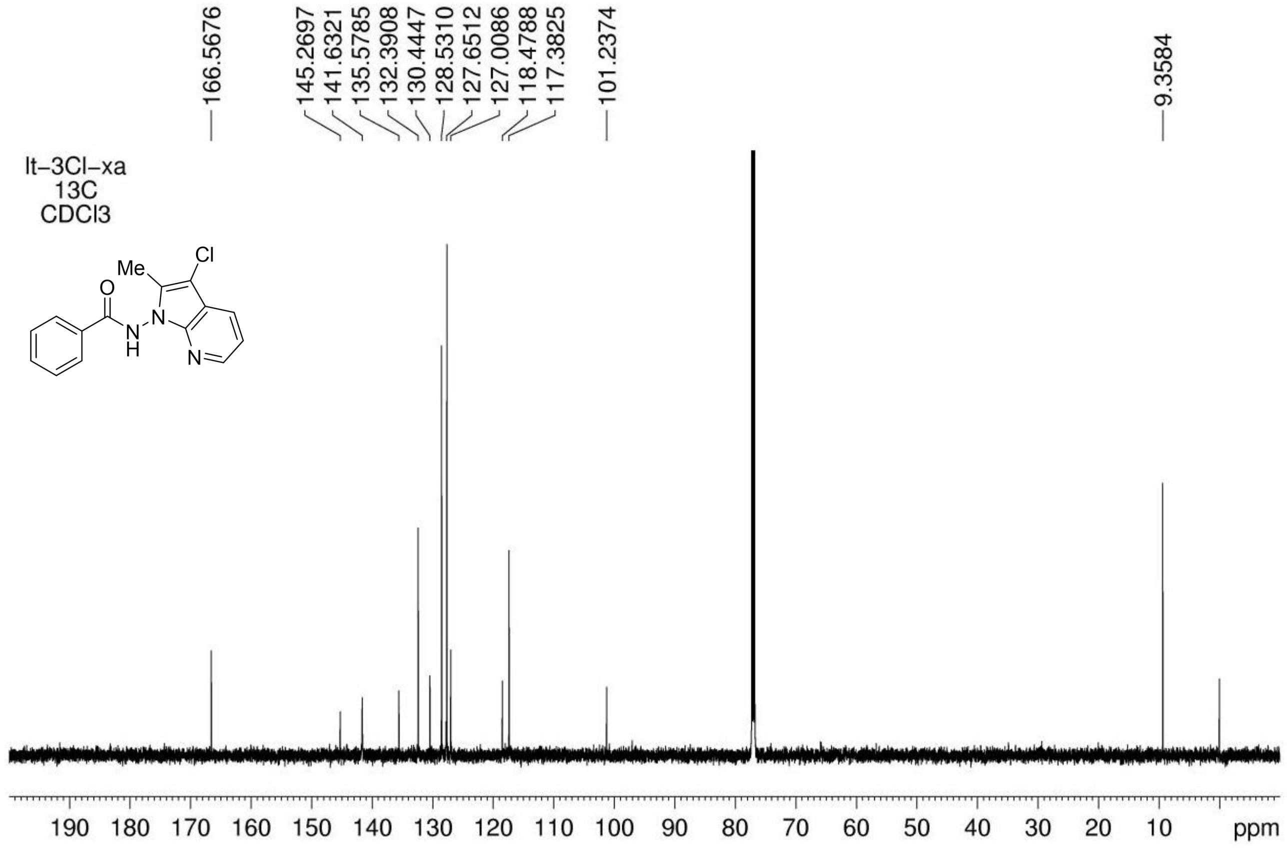
166.5676
145.2697
141.6321
135.5785
132.3908
130.4447
128.5310
127.6512
127.0086
118.4788
117.3825
101.2374
9.3584
lt–3Cl–xa
13C
CDCl3
Me
Cl
O
N
N
H
N
190 180 170 160 150 140 130 120 110 100 90 80 70 60 50 40 30 20 10 ppm

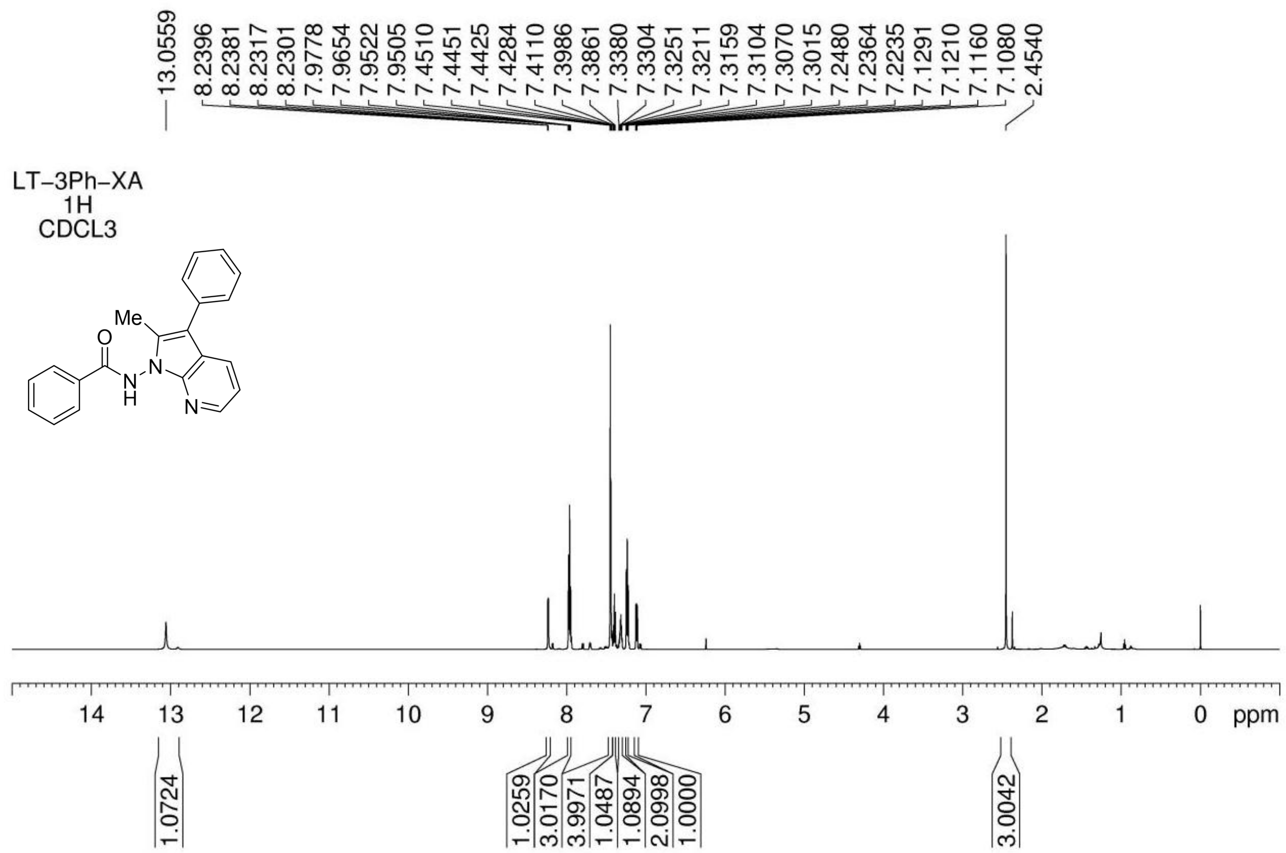
LT−3Ph−XA
1H
CDCL3
Me
O
N
H
N
N
13.0559
8.2396
8.2381
8.2317
8.2301
7.9778
7.9654
7.9522
7.9505
7.4510
7.4451
7.4425
7.4284
7.4110
7.3986
7.3861
7.3380
7.3304
7.3251
7.3211
7.3159
7.3104
7.3070
7.3015
7.2480
7.2364
7.2235
7.1291
7.1210
7.1160
7.1080
2.4540
14 13 12 11 10 9 8 7 6 5 4 3 2 1 0 ppm
1.0724
1.0259
3.0170
3.9971
1.0487
1.0894
2.0998
1.0000
3.0042

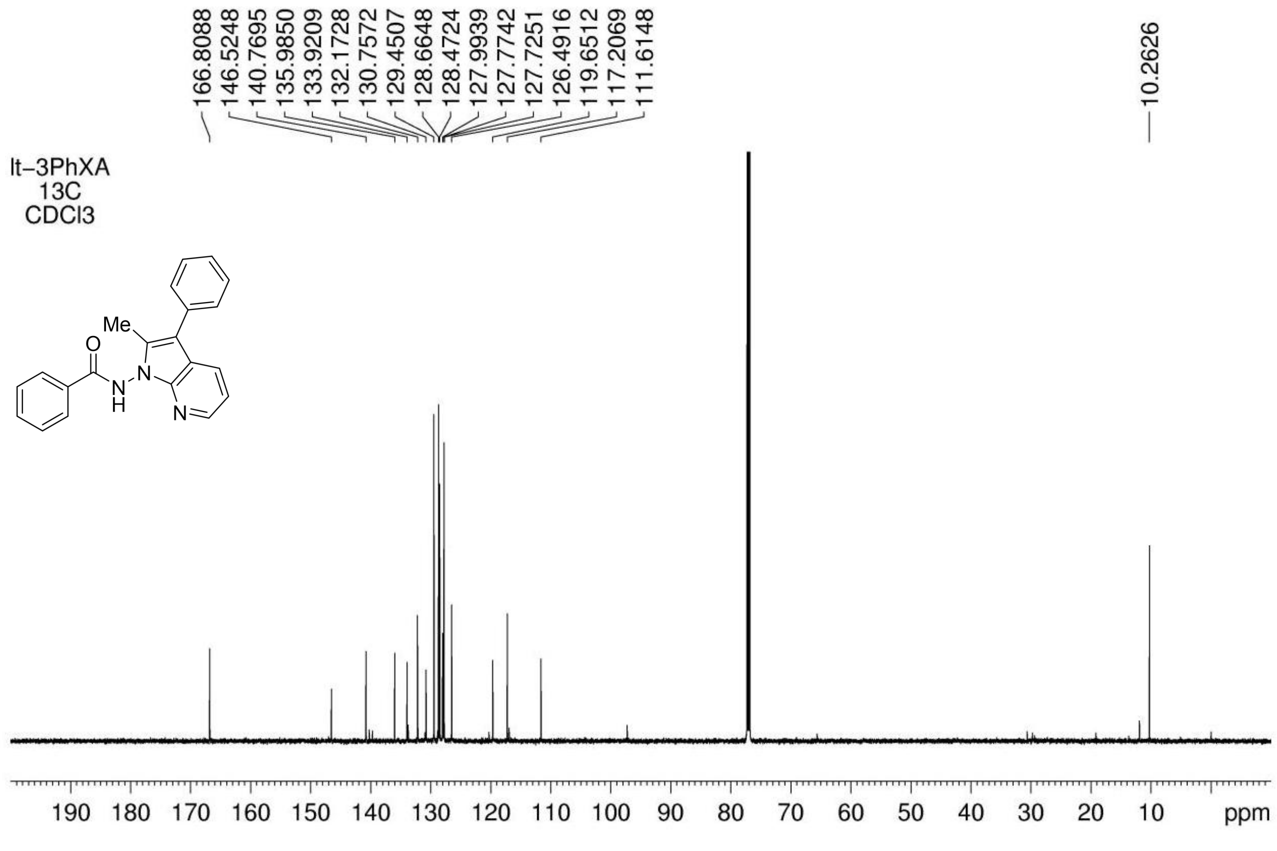
lt−3PhXA
13C
CDCl3
Me
O
N
H
N
N
166.8088
146.5248
140.7695
135.9850
133.9209
132.1728
130.7572
129.4507
128.6648
128.4724
127.9939
127.7742
127.7251
126.4916
119.6512
117.2069
111.6148
10.2626
190 180 170 160 150 140 130 120 110 100 90 80 70 60 50 40 30 20 10 ppm

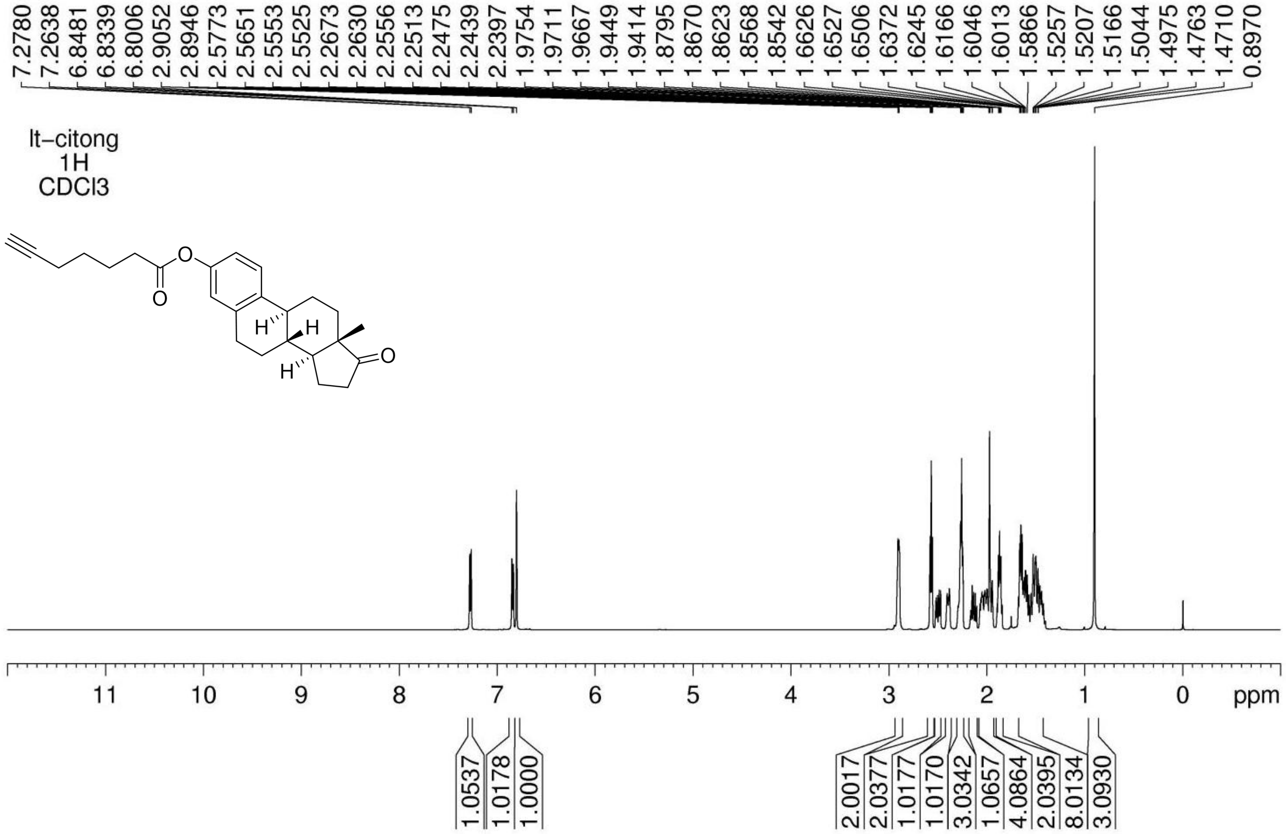
7.2780 7.2638 6.8481 6.8339 6.8006 2.9052 2.8946 2.5773 2.5651 2.5553 2.5525 2.2673 2.2630 2.2556 2.2513 2.2475 2.2439 2.2397 1.9754 1.9711 1.9667 1.9449 1.9414 1.8795 1.8670 1.8623 1.8568 1.8542 1.6626 1.6527 1.6506 1.6372 1.6245 1.6166 1.6046 1.6013 1.5866 1.5257 1.5207 1.5166 1.5044 1.4975 1.4763 1.4710 0.8970
lt–citong
1H
CDCl3
O
H
H
H
O
11 10 9 8 7 6 5 4 3 2 1 0 ppm
1.0537 1.0178 1.0000
2.0017 2.0377 1.0177 1.0170 3.0342 1.0657 4.0864 2.0395 8.0134 3.0930

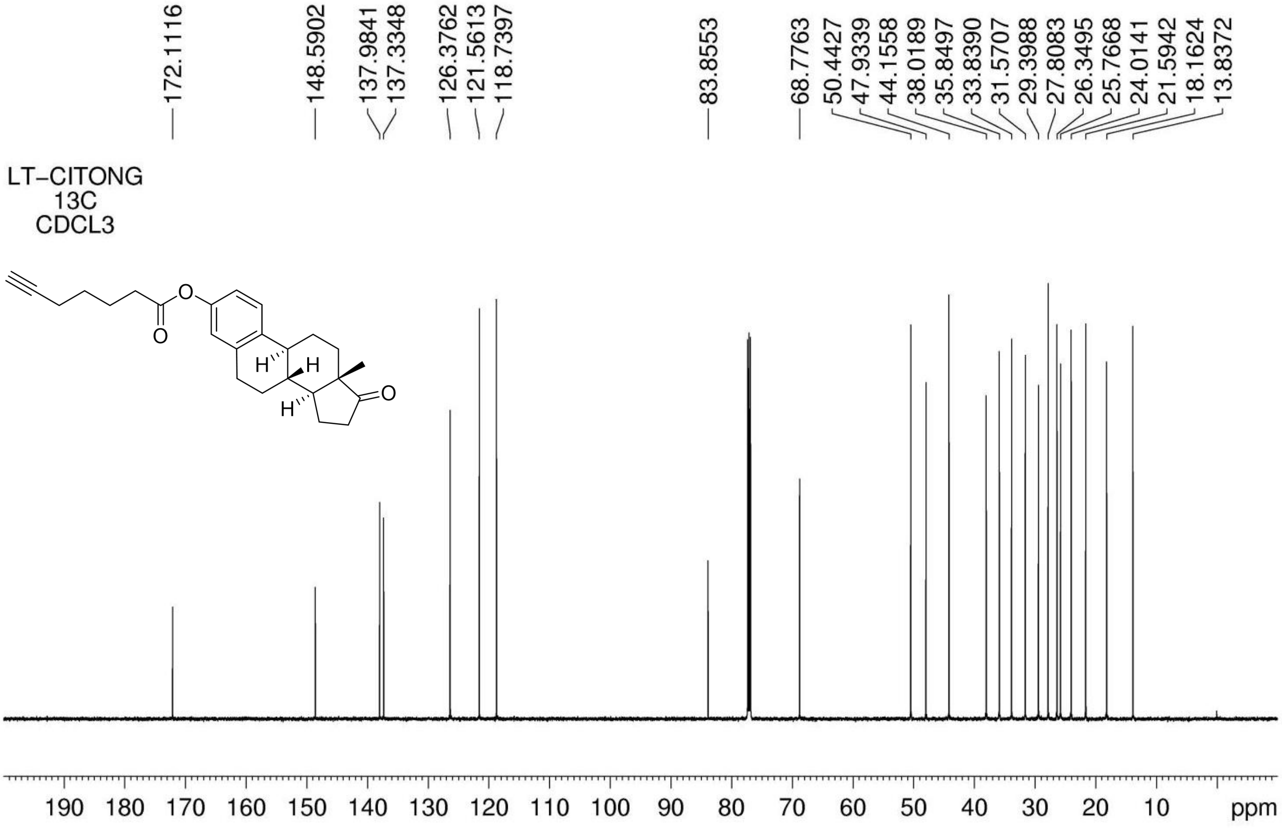
172.1116
148.5902
137.9841 137.3348
126.3762 121.5613 118.7397
83.8553
68.7763 50.4427 47.9339 44.1558 38.0189 35.8497 33.8390 31.5707 29.3988 27.8083 26.3495 25.7668 24.0141 21.5942 18.1624 13.8372
LT–CITONG
13C
CDCL3
O
H
H
H
O
190 180 170 160 150 140 130 120 110 100 90 80 70 60 50 40 30 20 10 ppm

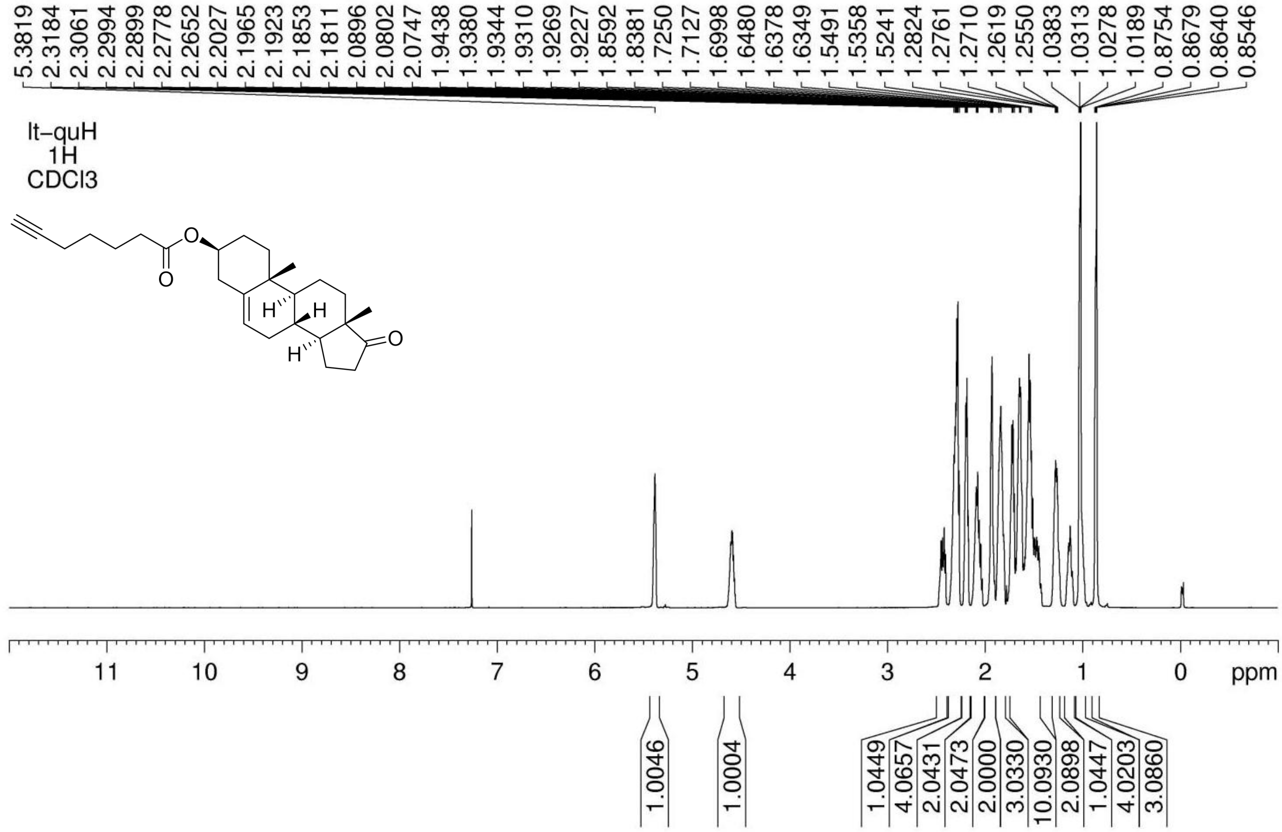

5.3819 2.3184 2.3061 2.2994 2.2899 2.2778 2.2652 2.2027 2.1965 2.1923 2.1853 2.1811 2.0896 2.0802 2.0747 1.9438 1.9380 1.9344 1.9310 1.9269 1.9227 1.8592 1.8381 1.7250 1.7127 1.6998 1.6480 1.6378 1.6349 1.5491 1.5358 1.5241 1.2824 1.2761 1.2710 1.2619 1.2550 1.0383 1.0313 1.0278 1.0189 0.8754 0.8679 0.8640 0.8546
lt–quH
1H
CDCl3
O
H
H
H
O
11 10 9 8 7 6 5 4 3 2 1 0 ppm
1.0046 1.0004 1.0449 4.0657 2.0431 2.0473 2.0000 3.0330 10.0930 2.0898 1.0447 4.0203 3.0860

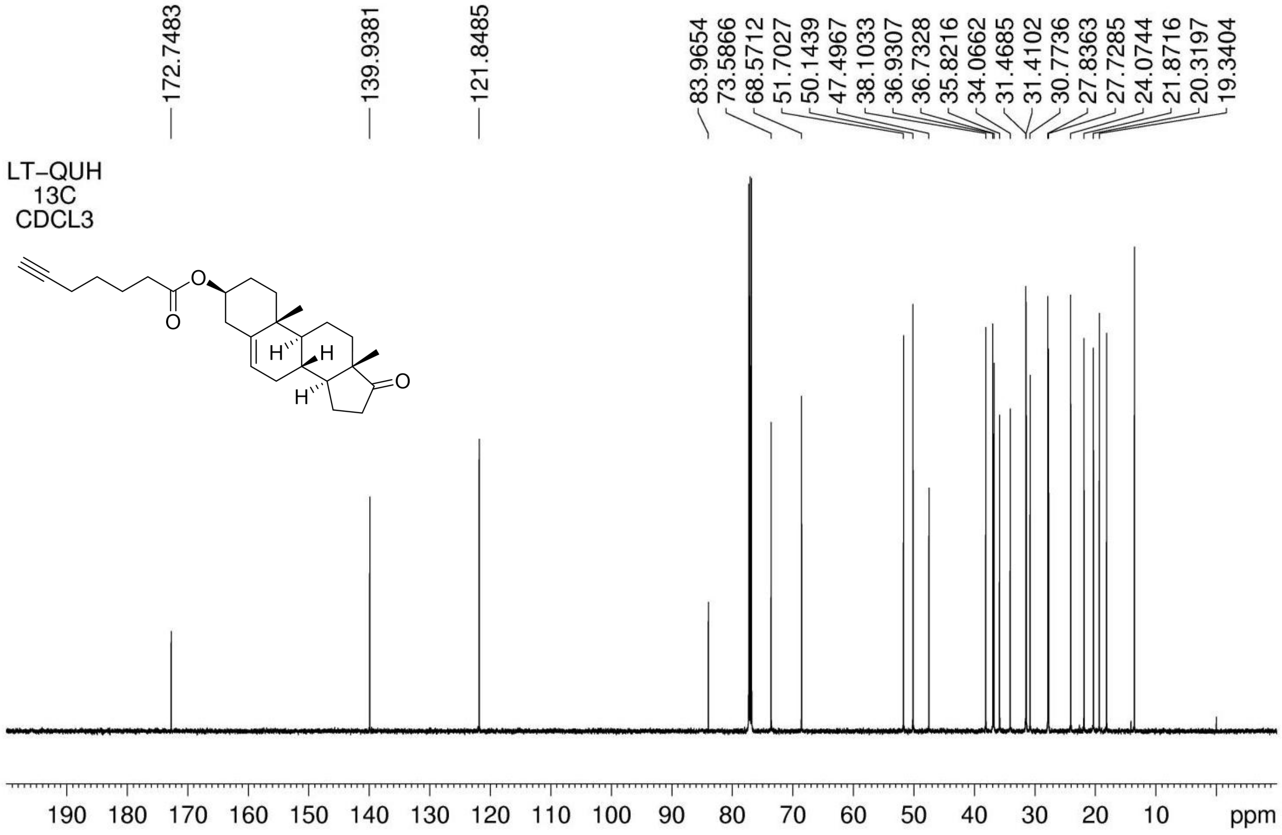

172.7483 139.9381 121.8485 83.9654 73.5866 68.5712 51.7027 50.1439 47.4967 38.1033 36.9307 36.7328 35.8216 34.0662 31.4685 31.4102 30.7736 27.8363 27.7285 24.0744 21.8716 20.3197 19.3404
LT–QUH
13C
CDCL3
O
H
H
H
O
190 180 170 160 150 140 130 120 110 100 90 80 70 60 50 40 30 20 10 ppm

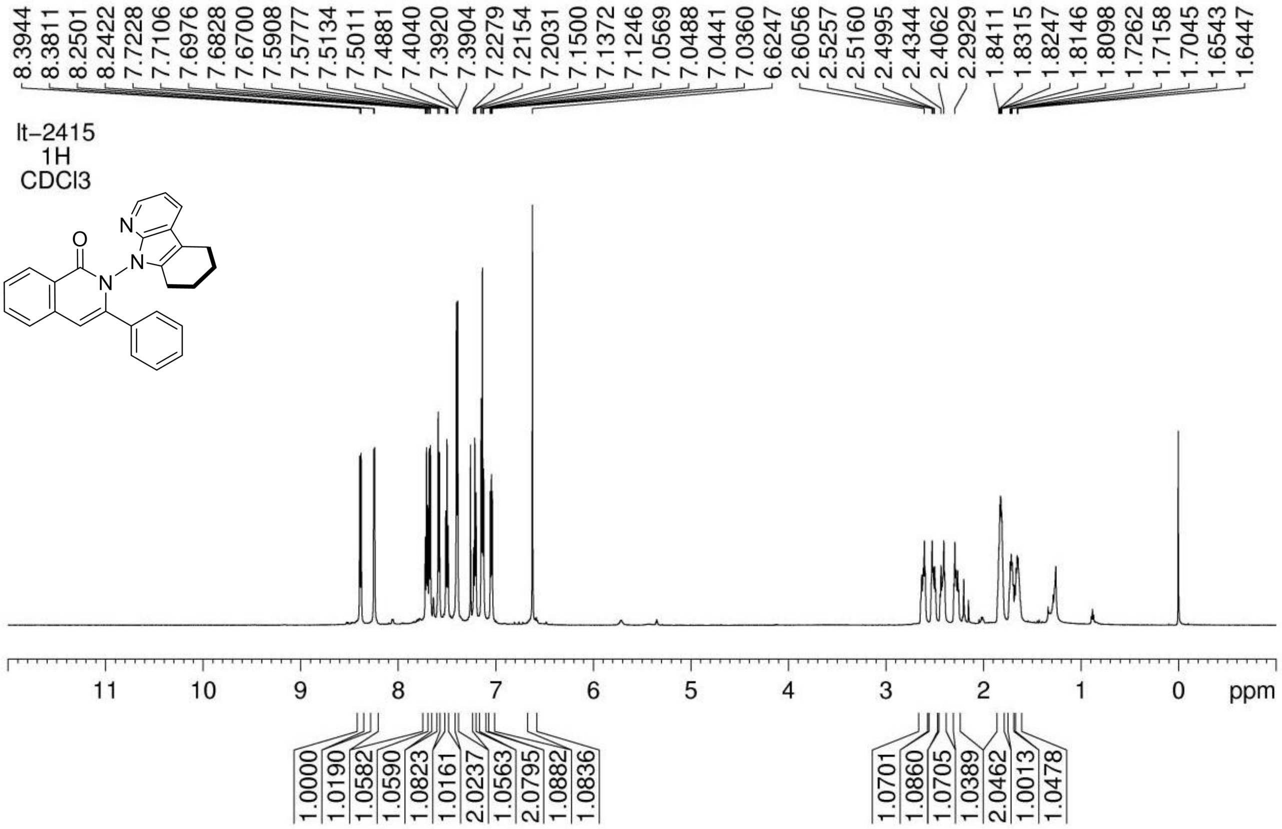
8.3944
8.3811
8.2501
8.2422
7.7228
7.7106
7.6976
7.6828
7.6700
7.5908
7.5777
7.5134
7.5011
7.4881
7.4040
7.3920
7.3904
7.2279
7.2154
7.2031
7.1500
7.1372
7.1246
7.0569
7.0488
7.0441
7.0360
6.6247
2.6056
2.5257
2.5160
2.4995
2.4344
2.4062
2.2929
1.8411
1.8315
1.8247
1.8146
1.8098
1.7262
1.7158
1.7045
1.6543
1.6447
lt-2415
1H
CDCl3
O
N
N
N
11
10
9
8
7
6
5
4
3
2
1
0
ppm
1.0000
1.0190
1.0582
1.0590
1.0823
1.0161
2.0237
1.0563
2.0795
1.0882
1.0836
1.0701
1.0860
1.0705
1.0389
2.0462
1.0013
1.0478

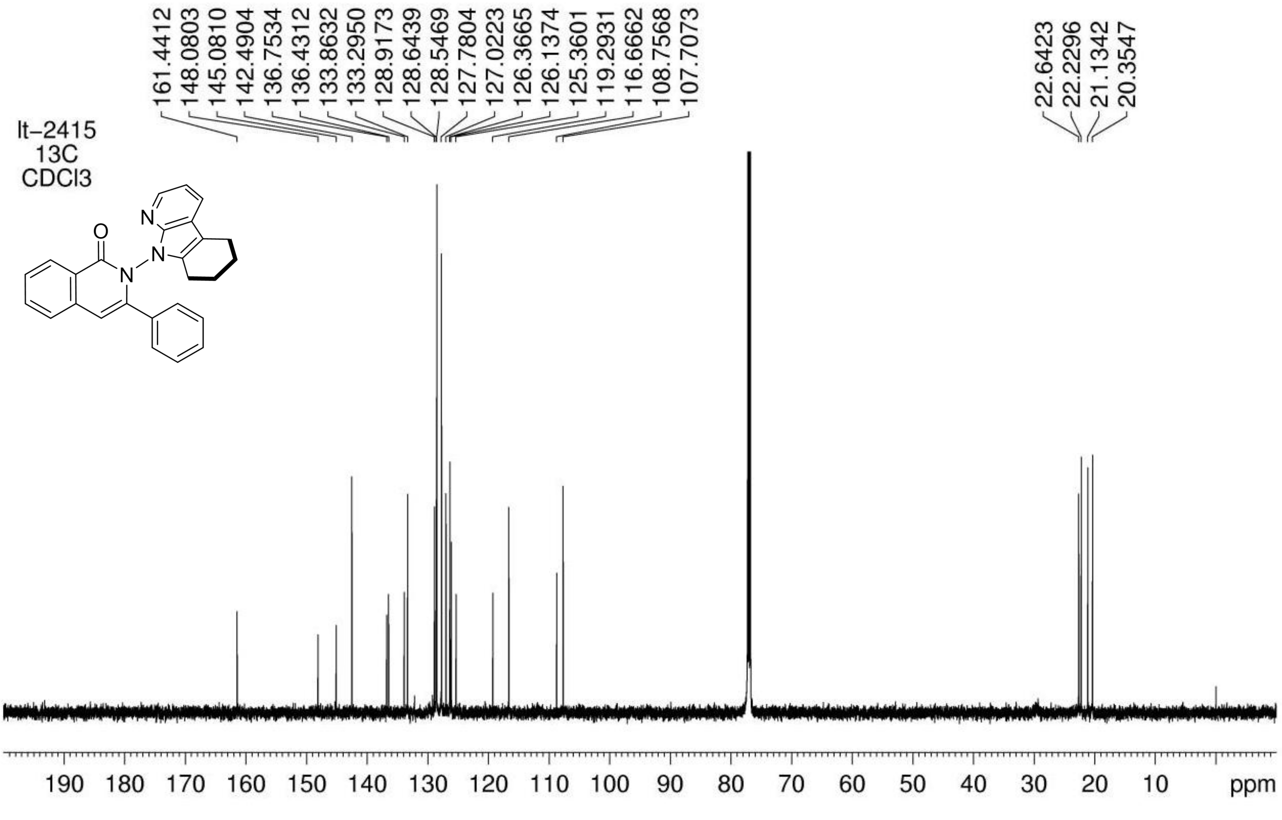
161.4412
148.0803
145.0810
142.4904
136.7534
136.4312
133.8632
133.2950
128.9173
128.6439
128.5469
127.7804
127.0223
126.3665
126.1374
125.3601
119.2931
116.6662
108.7568
107.7073
22.6423
22.2296
21.1342
20.3547
lt-2415
13C
CDCl3
O
N
N
N
190
180
170
160
150
140
130
120
110
100
90
80
70
60
50
40
30
20
10
ppm

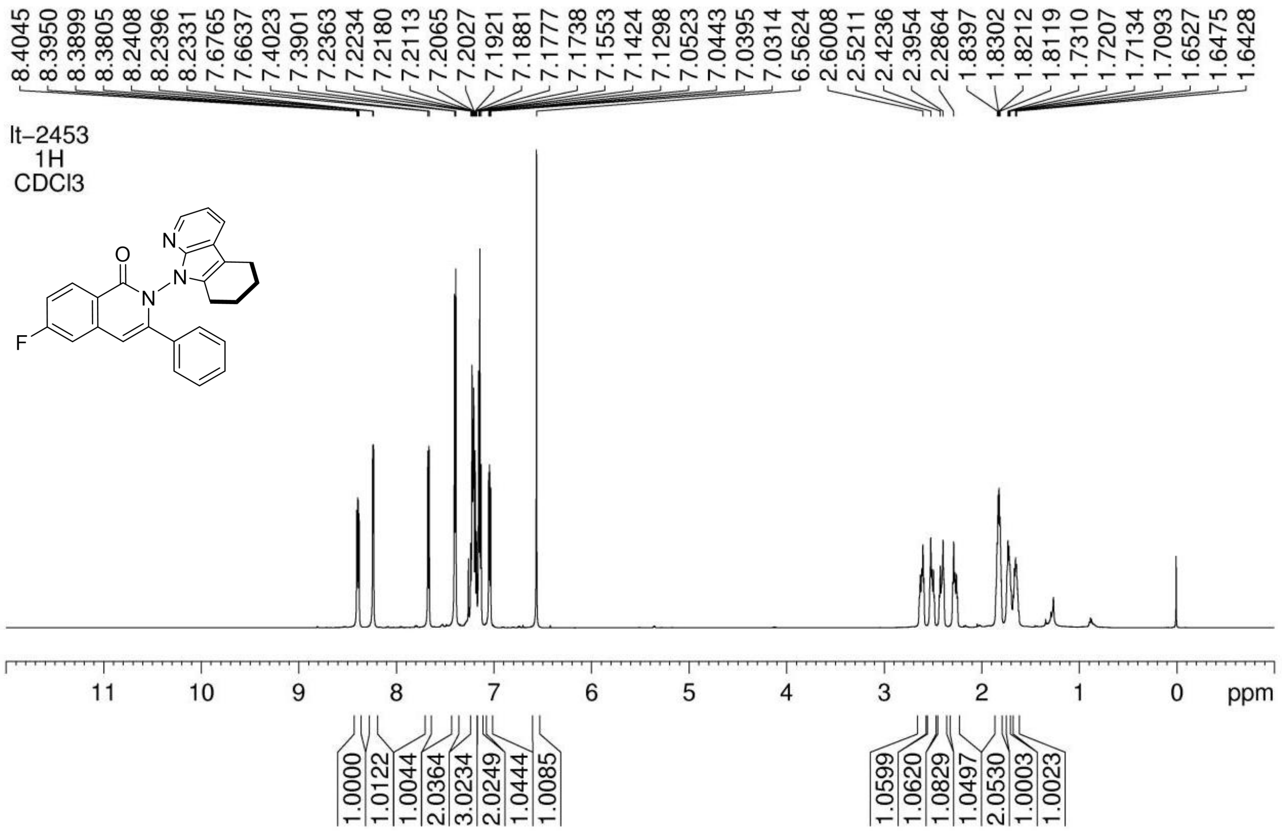
lt–2453
1H
CDCl3
N
O
N
N
F
8.4045
8.3950
8.3899
8.3805
8.2408
8.2396
8.2331
7.6765
7.6637
7.4023
7.3901
7.2363
7.2234
7.2180
7.2113
7.2065
7.2027
7.1921
7.1881
7.1777
7.1738
7.1553
7.1424
7.1298
7.0523
7.0443
7.0395
7.0314
6.5624
2.6008
2.5211
2.4236
2.3954
2.2864
1.8397
1.8302
1.8212
1.8119
1.7310
1.7207
1.7134
1.7093
1.6527
1.6475
1.6428
11 10 9 8 7 6 5 4 3 2 1 0 ppm
1.0000
1.0122
1.0044
2.0364
3.0234
2.0249
1.0444
1.0085
1.0599
1.0620
1.0829
1.0497
2.0530
1.0003
1.0023

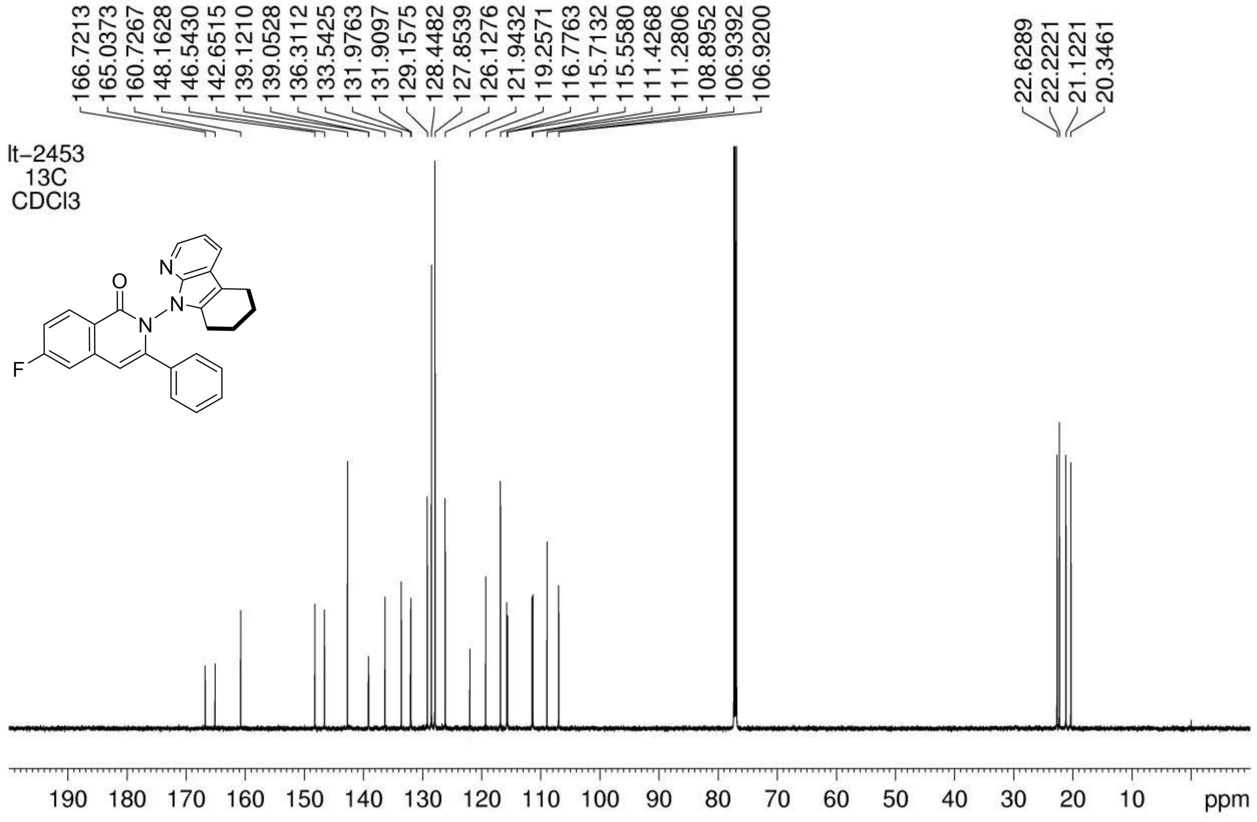
lt–2453
13C
CDCl3
N
O
N
N
F
166.7213
165.0373
160.7267
148.1628
146.5430
142.6515
139.1210
139.0528
136.3112
133.5425
131.9763
131.9097
129.1575
128.4482
127.8539
126.1276
121.9432
119.2571
116.7763
115.7132
115.5580
111.4268
111.2806
108.8952
106.9392
106.9200
22.6289
22.2221
21.1221
20.3461
190 180 170 160 150 140 130 120 110 100 90 80 70 60 50 40 30 20 10 ppm

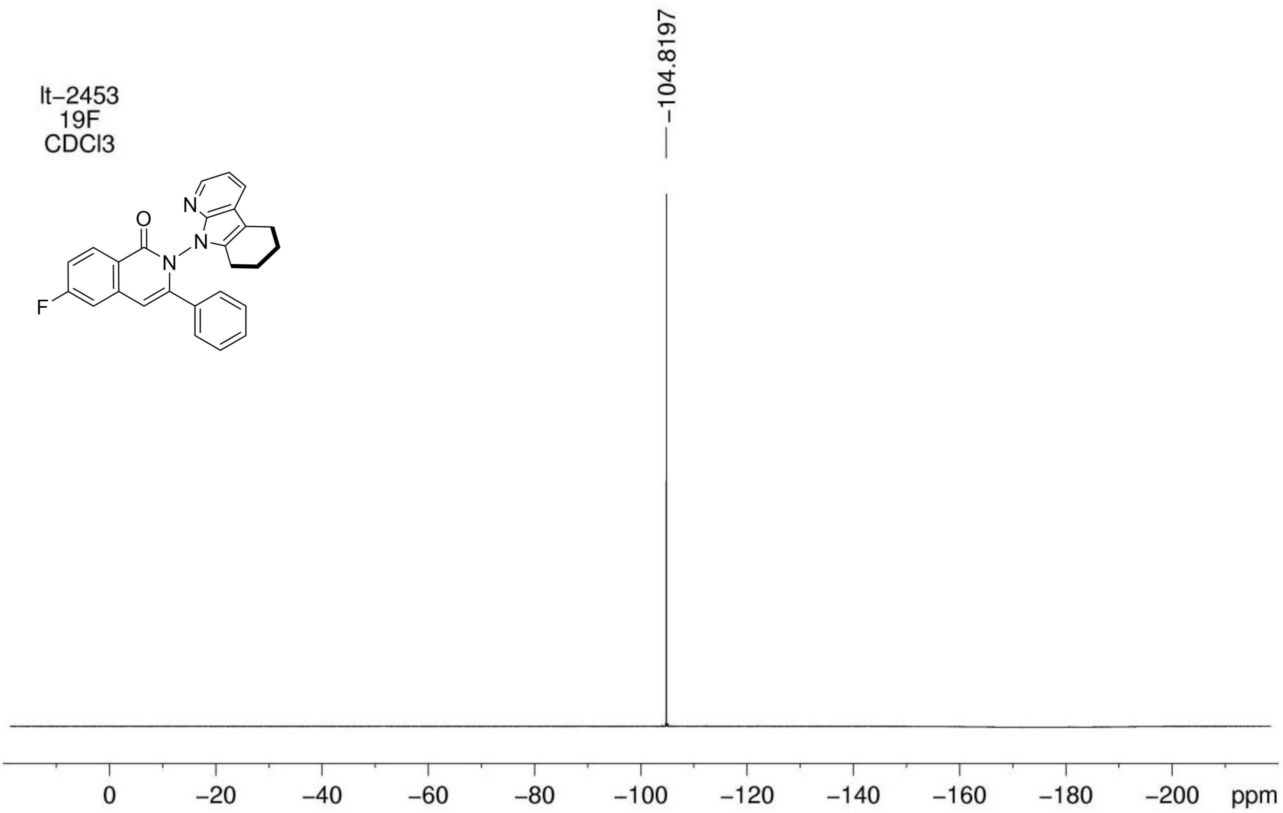

lt−2453
19F
CDCl3
−104.8197
O
N
N
N
F
0
−20
−40
−60
−80
−100
−120
−140
−160
−180
−200
ppm

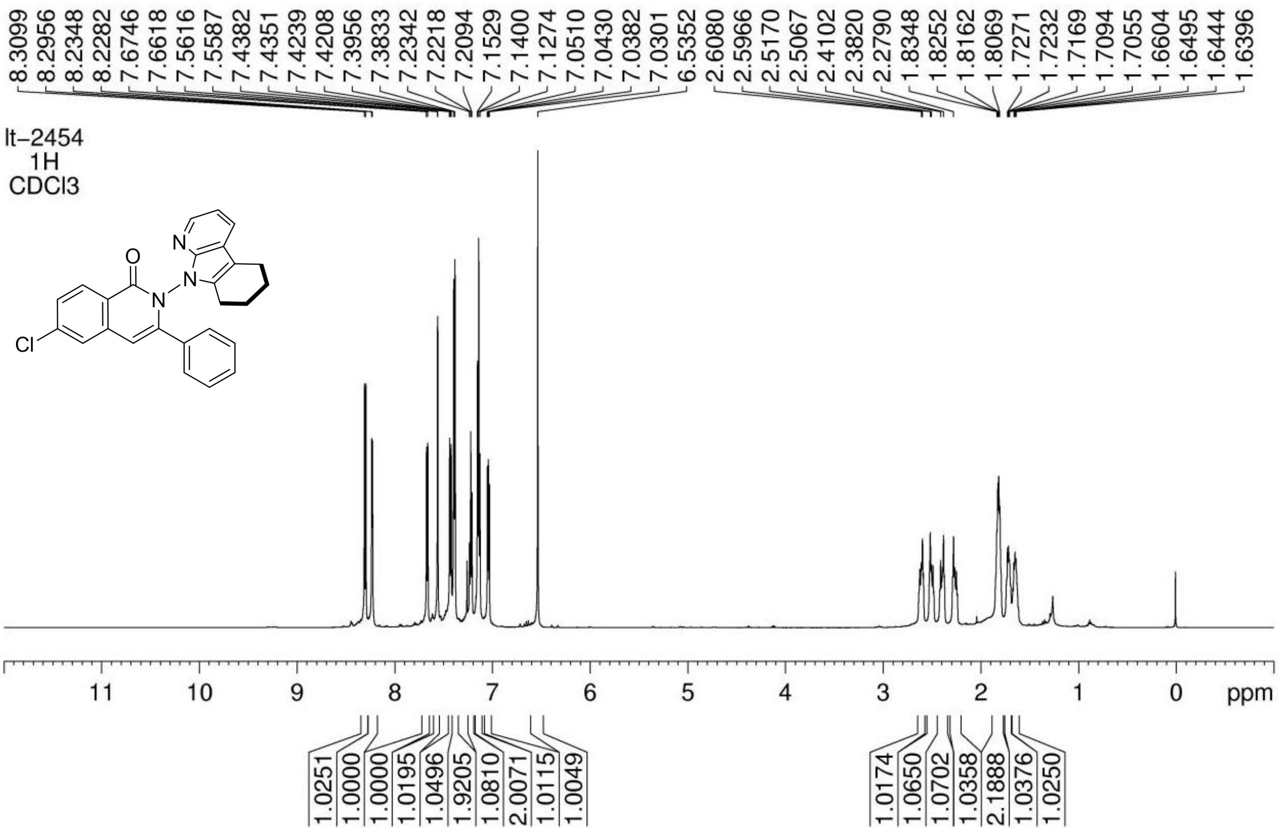
8.3099
8.2956
8.2348
8.2282
7.6746
7.6618
7.5616
7.5587
7.4382
7.4351
7.4239
7.4208
7.3956
7.3833
7.2342
7.2218
7.2094
7.1529
7.1400
7.1274
7.0510
7.0430
7.0382
7.0301
6.5352
2.6080
2.5966
2.5170
2.5067
2.4102
2.3820
2.2790
1.8348
1.8252
1.8162
1.8069
1.7271
1.7232
1.7169
1.7094
1.7055
1.6604
1.6495
1.6444
1.6396
lt–2454
1H
CDCl3
O
N
N
N
Cl
11 10 9 8 7 6 5 4 3 2 1 0 ppm
1.0251
1.0000
1.0000
1.0195
1.0496
1.9205
1.0810
2.0071
1.0115
1.0049
1.0174
1.0650
1.0702
1.0358
2.1888
1.0376
1.0250

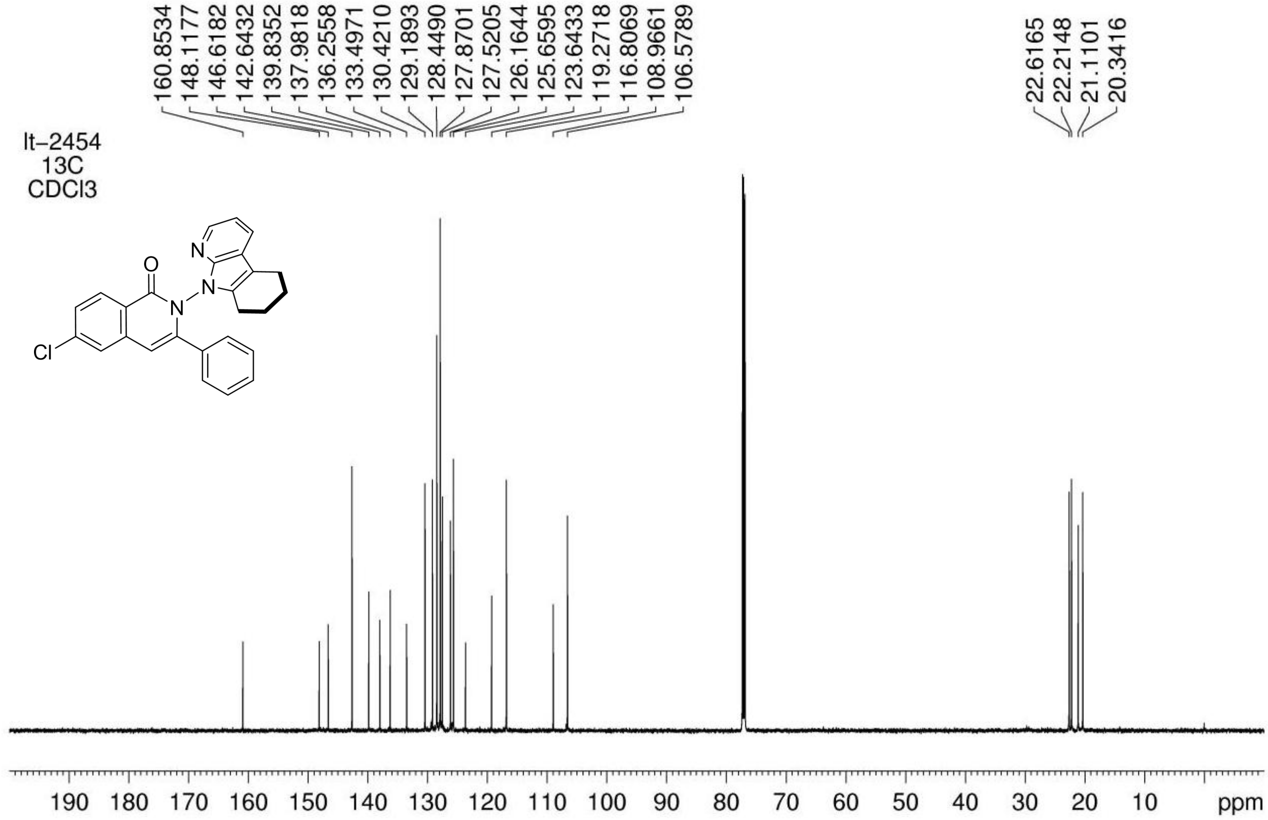
160.8534
148.1177
146.6182
142.6432
139.8352
137.9818
136.2558
133.4971
130.4210
129.1893
128.4490
127.8701
127.5205
126.1644
125.6595
123.6433
119.2718
116.8069
108.9661
106.5789
22.6165
22.2148
21.1101
20.3416
lt–2454
13C
CDCl3
O
N
N
N
Cl
190 180 170 160 150 140 130 120 110 100 90 80 70 60 50 40 30 20 10 ppm

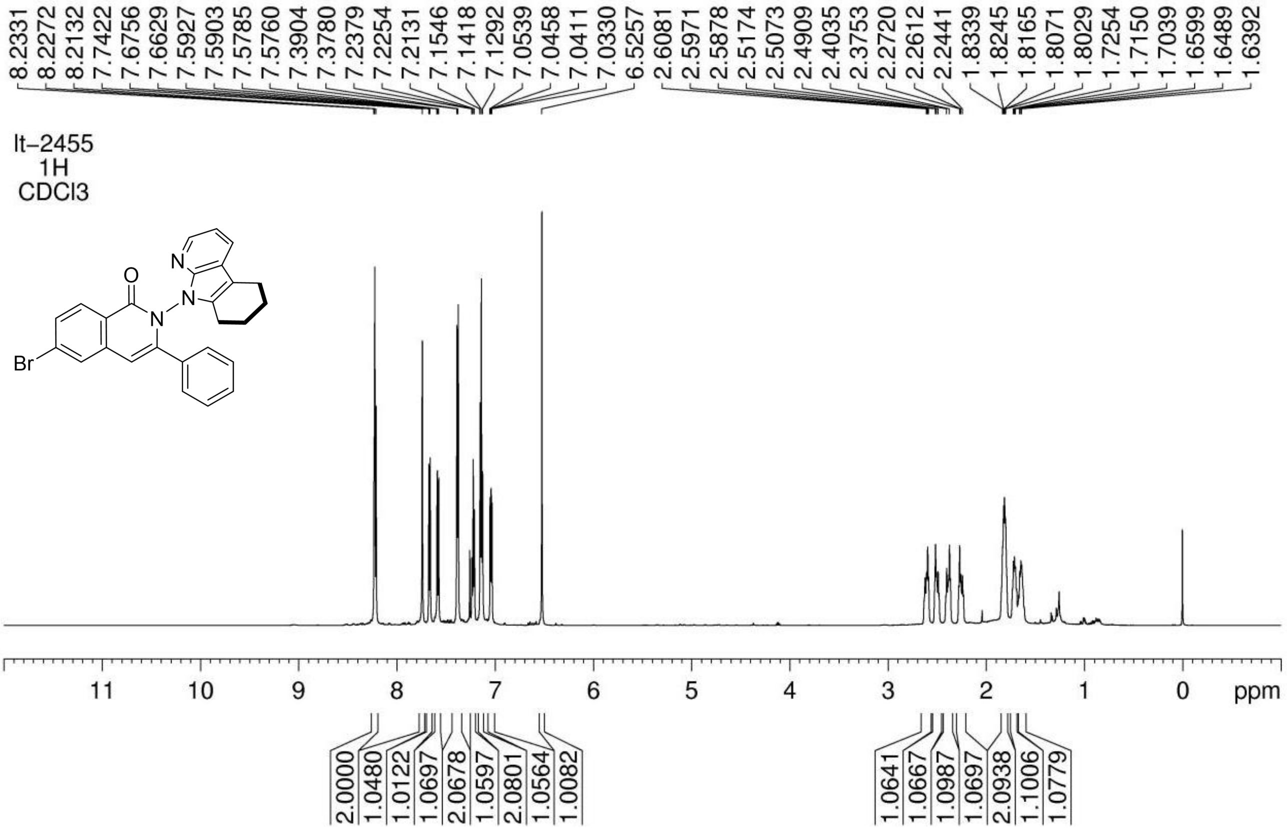
8.2331
8.2272
8.2132
7.7422
7.6756
7.6629
7.5927
7.5903
7.5785
7.5760
7.3904
7.3780
7.2379
7.2254
7.2131
7.1546
7.1418
7.1292
7.0539
7.0458
7.0411
7.0330
6.5257
2.6081
2.5971
2.5878
2.5174
2.5073
2.4909
2.4035
2.3753
2.2720
2.2612
2.2441
1.8339
1.8245
1.8165
1.8071
1.8029
1.7254
1.7150
1.7039
1.6599
1.6489
1.6392
lt−2455
1H
CDCl3
O
N
N
N
Br
11
10
9
8
7
6
5
4
3
2
1
0
ppm
2.0000
1.0480
1.0122
1.0697
2.0678
1.0597
2.0801
1.0564
1.0082
1.0641
1.0667
1.0987
1.0697
2.0938
1.1006
1.0779

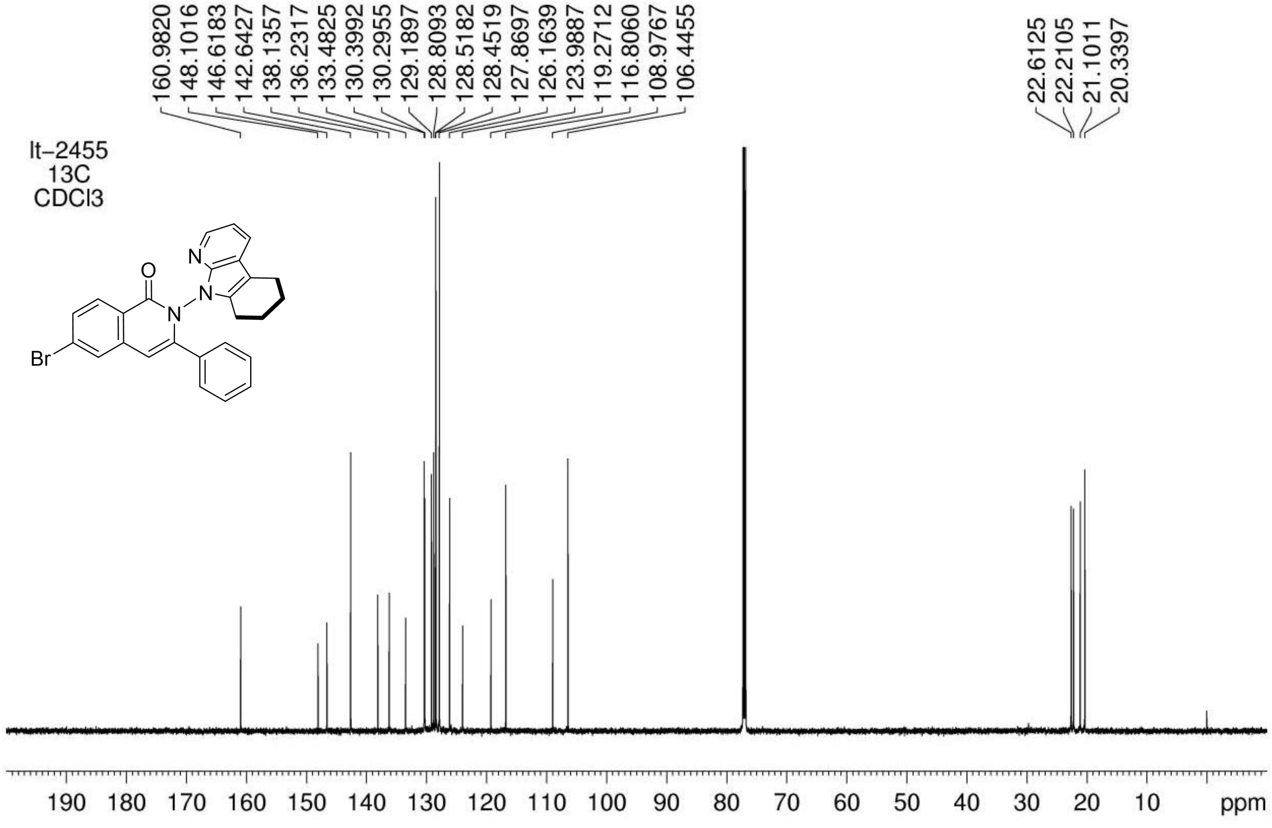
160.9820
148.1016
146.6183
142.6427
138.1357
136.2317
133.4825
130.3992
130.2955
129.1897
128.8093
128.5182
128.4519
127.8697
126.1639
123.9887
119.2712
116.8060
108.9767
106.4455
22.6125
22.2105
21.1011
20.3397
lt−2455
13C
CDCl3
O
N
N
N
Br
190
180
170
160
150
140
130
120
110
100
90
80
70
60
50
40
30
20
10
ppm

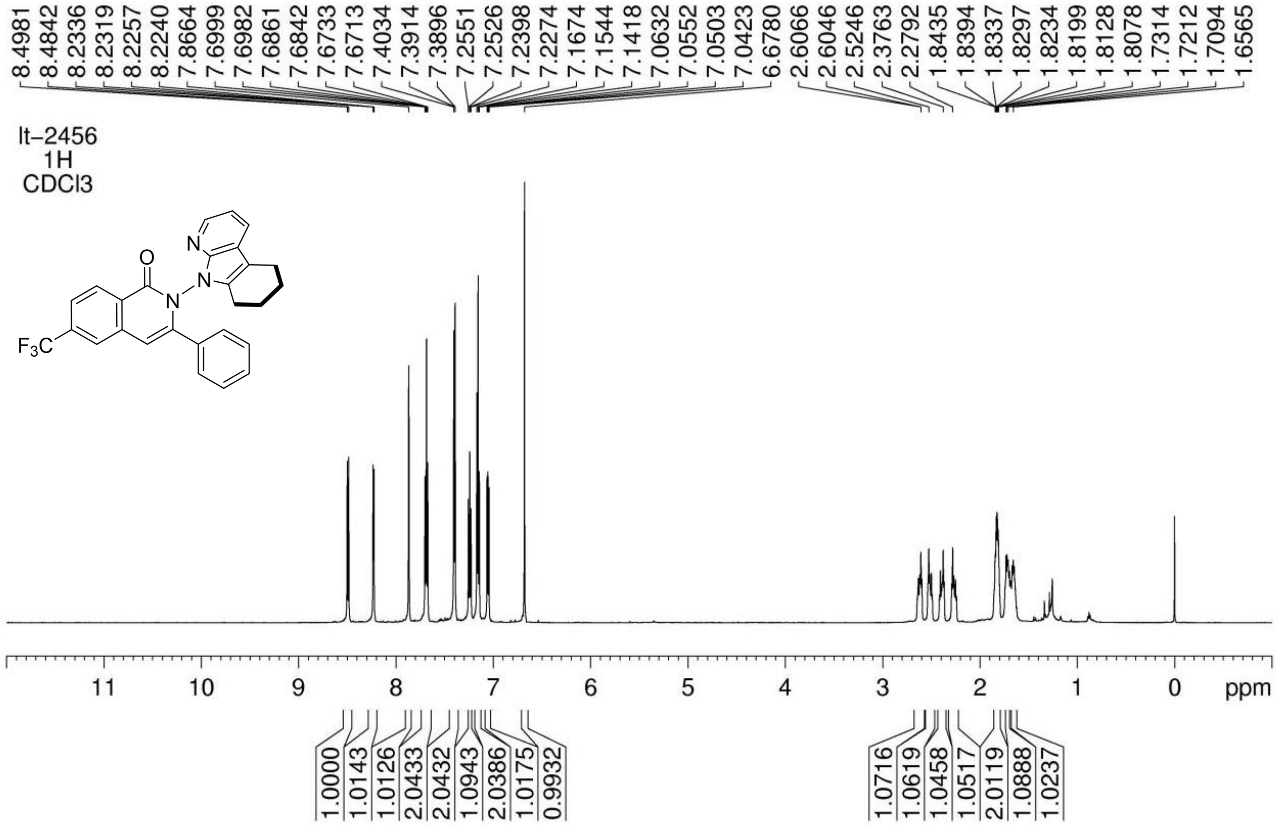
8.4981 8.4842 8.2336 8.2319 8.2257 8.2240 7.8664 7.6999 7.6982 7.6861 7.6842 7.6733 7.6713 7.4034 7.3914 7.3896 7.2551 7.2526 7.2398 7.2274 7.1674 7.1544 7.1418 7.0632 7.0552 7.0503 7.0423 6.6780 2.6066 2.6046 2.5246 2.3763 2.2792 1.8435 1.8394 1.8337 1.8297 1.8234 1.8199 1.8128 1.8078 1.7314 1.7212 1.7094 1.6565
lt–2456
1H
CDCl3
O
N
N
N
F_3C
11 10 9 8 7 6 5 4 3 2 1 0 ppm
1.0000 1.0143 1.0126 2.0433 2.0432 1.0943 2.0386 1.0175 0.9932
1.0716 1.0619 1.0458 1.0517 2.0119 1.0888 1.0237

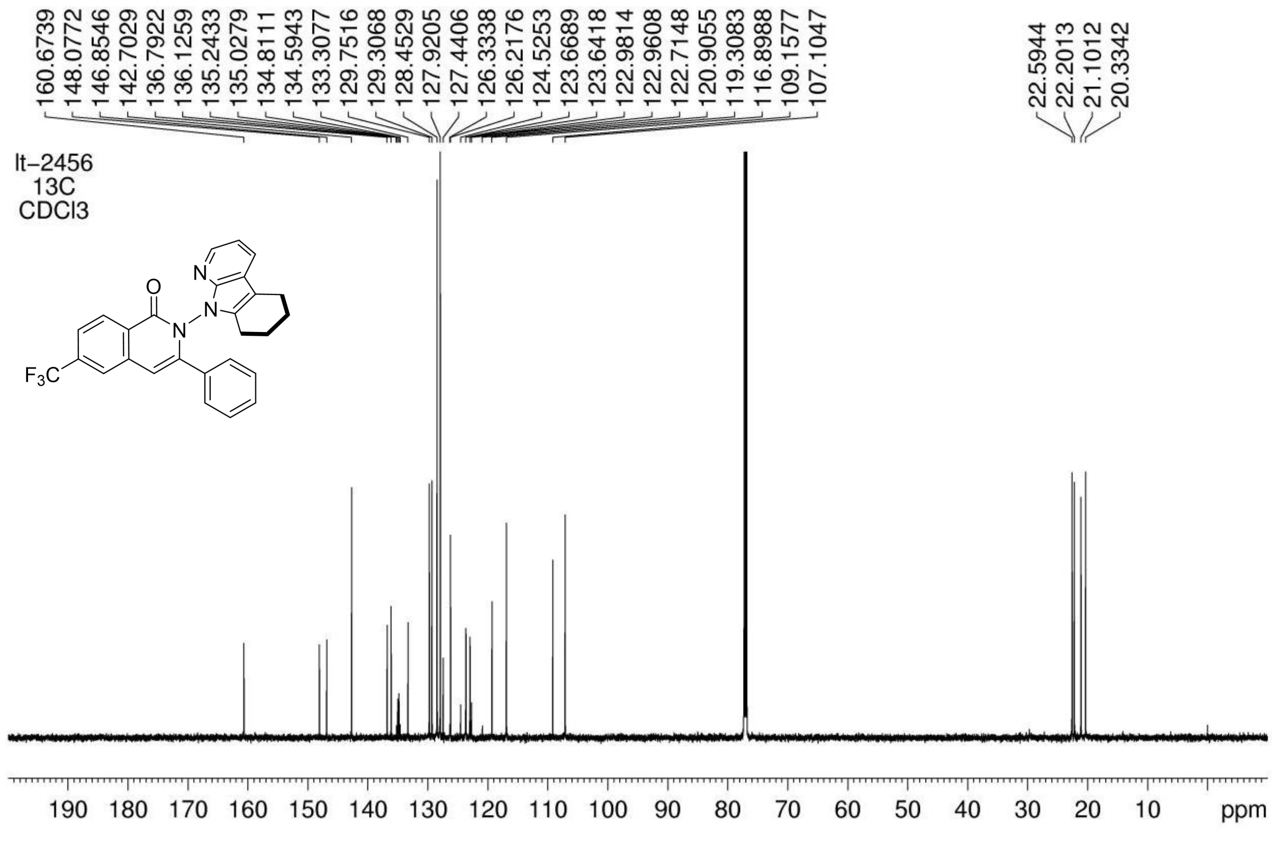
160.6739 148.0772 146.8546 142.7029 136.7922 136.1259 135.2433 135.0279 134.8111 134.5943 133.3077 129.7516 129.3068 128.4529 127.9205 127.4406 126.3338 126.2176 124.5253 123.6689 123.6418 122.9814 122.9608 122.7148 120.9055 119.3083 116.8988 109.1577 107.1047
22.5944 22.2013 21.1012 20.3342
lt–2456
13C
CDCl3
O
N
N
N
F_3C
190 180 170 160 150 140 130 120 110 100 90 80 70 60 50 40 30 20 10 ppm

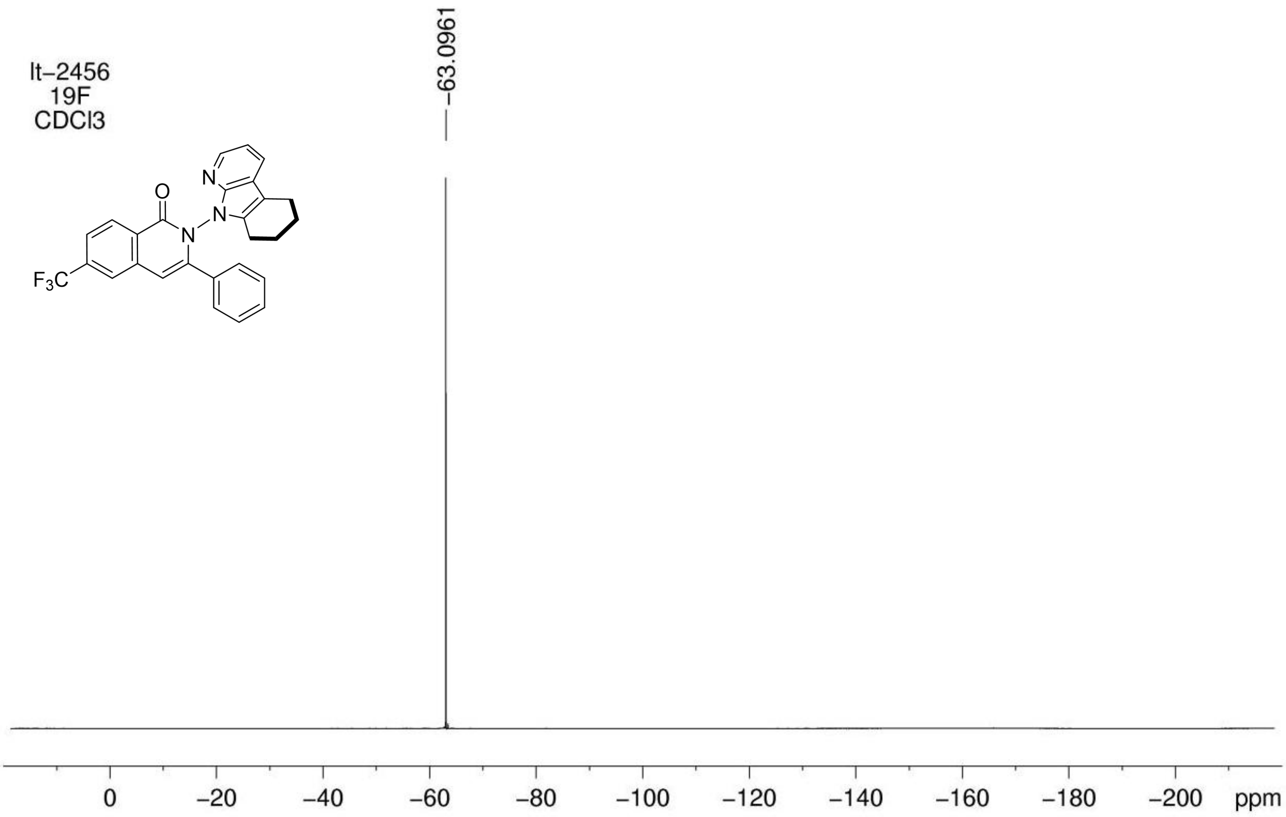

lt–2456
19F
CDCl3
O
N
N
N
F3C
–63.0961
0
–20
–40
–60
–80
–100
–120
–140
–160
–180
–200
ppm

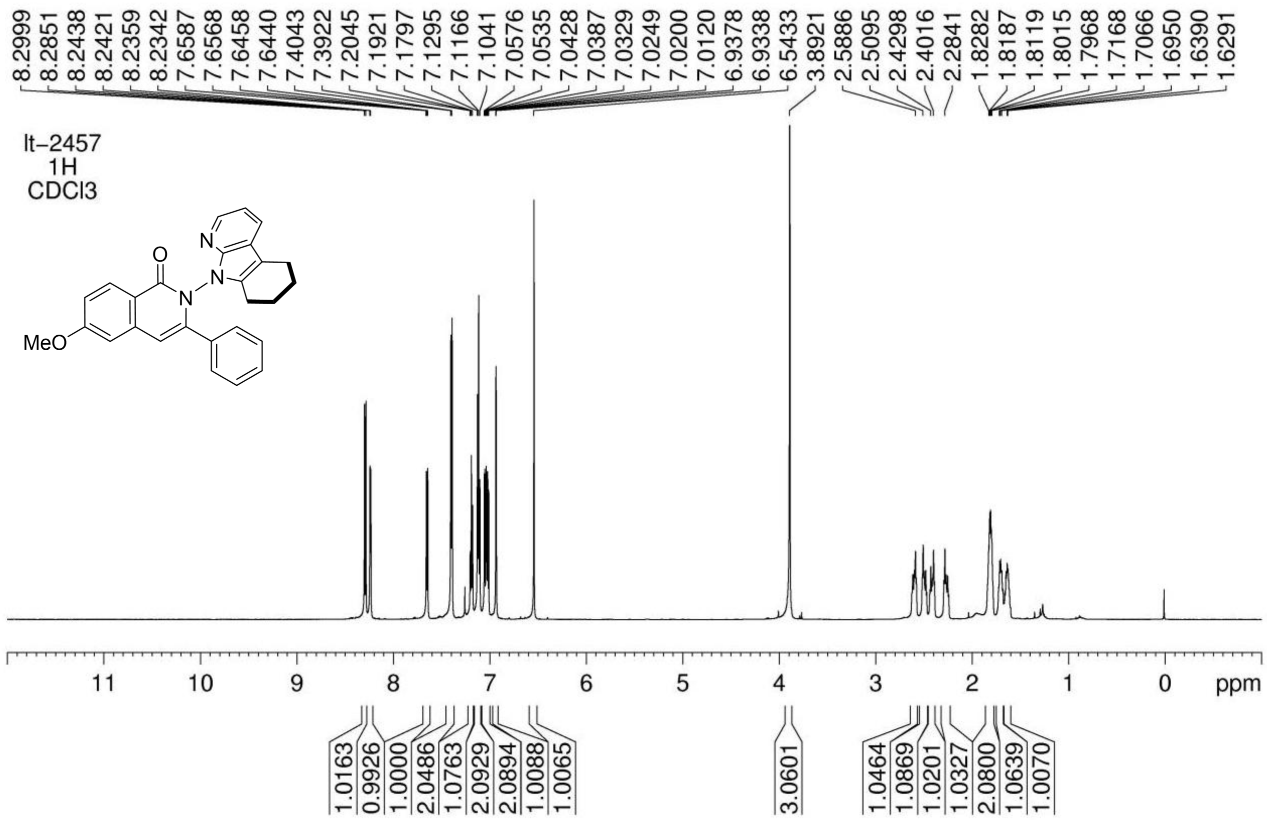

8.2999
8.2851
8.2438
8.2421
8.2359
8.2342
7.6587
7.6568
7.6458
7.6440
7.4043
7.3922
7.2045
7.1921
7.1797
7.1295
7.1166
7.1041
7.0576
7.0535
7.0428
7.0387
7.0329
7.0249
7.0200
7.0120
6.9378
6.9338
6.5433
3.8921
2.5886
2.5095
2.4298
2.4016
2.2841
1.8282
1.8187
1.8119
1.8015
1.7968
1.7168
1.7066
1.6950
1.6390
1.6291
lt–2457
1H
CDCl3
N
O
N
N
MeO
11
10
9
8
7
6
5
4
3
2
1
0
ppm
1.0163
0.9926
1.0000
2.0486
1.0763
2.0929
2.0894
1.0088
1.0065
3.0601
1.0464
1.0869
1.0201
1.0327
2.0800
1.0639
1.0070

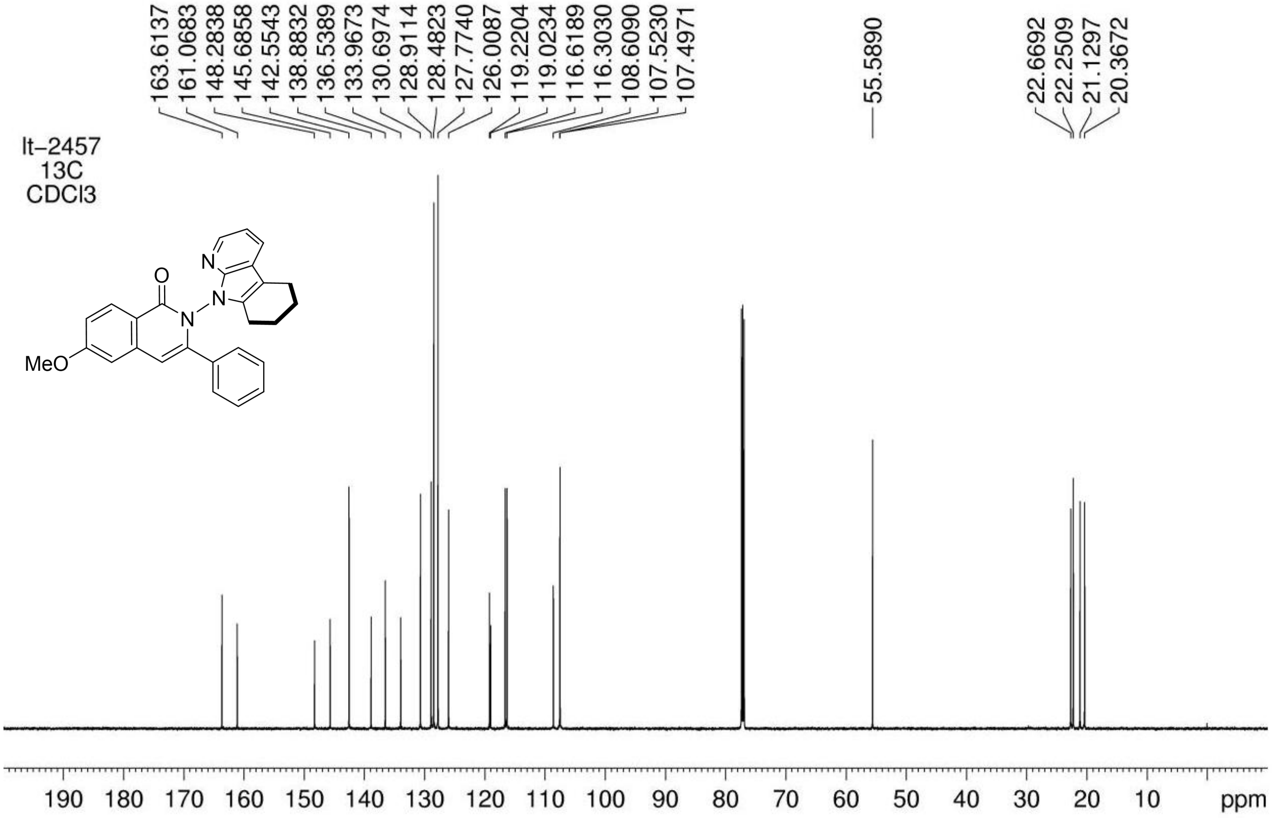

163.6137
161.0683
148.2838
145.6858
142.5543
138.8832
136.5389
133.9673
130.6974
128.9114
128.4823
127.7740
126.0087
119.2204
119.0234
116.6189
116.3030
108.6090
107.5230
107.4971
55.5890
22.6692
22.2509
21.1297
20.3672
lt–2457
13C
CDCl3
N
O
N
N
MeO
190
180
170
160
150
140
130
120
110
100
90
80
70
60
50
40
30
20
10
ppm

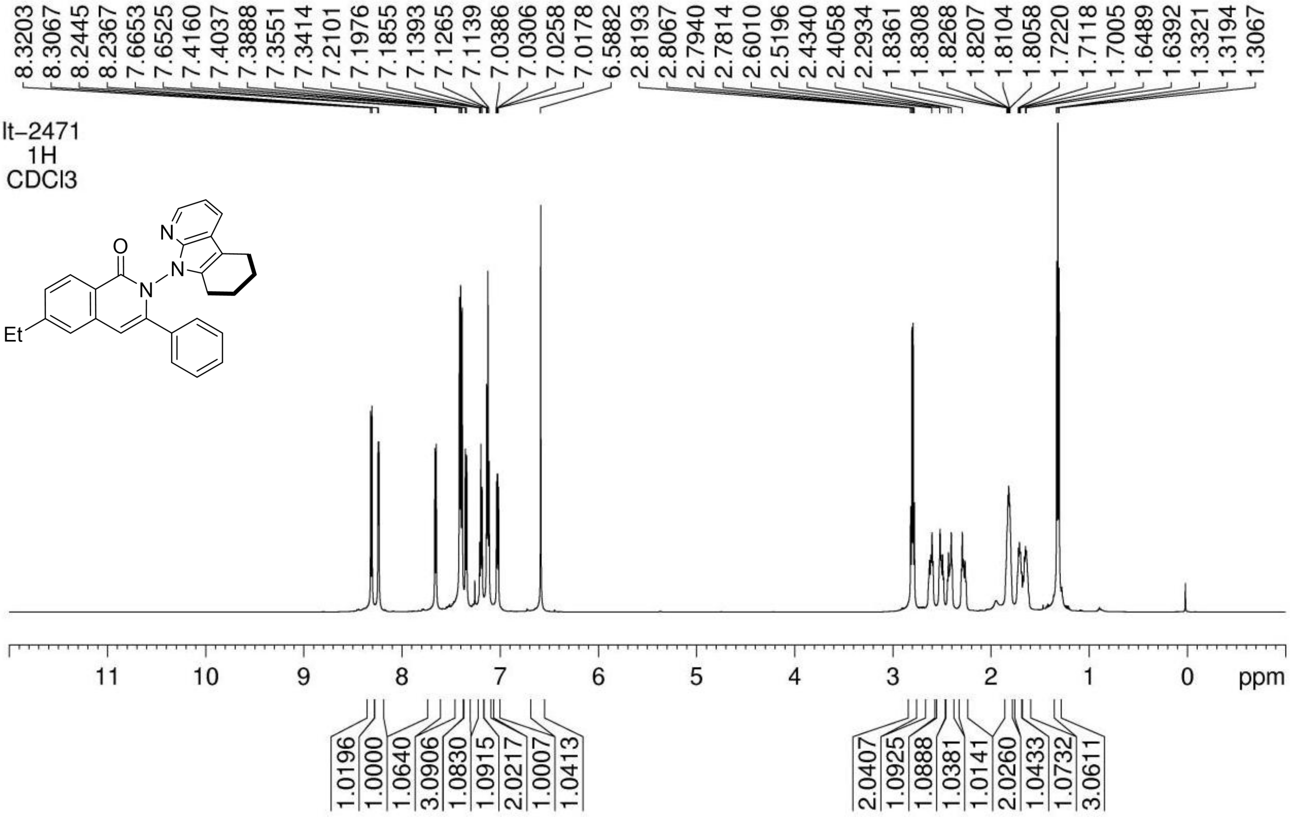
8.3203
8.3067
8.2445
8.2367
7.6653
7.6525
7.4160
7.4037
7.3888
7.3551
7.3414
7.2101
7.1976
7.1855
7.1393
7.1265
7.1139
7.0386
7.0306
7.0258
7.0178
6.5882
2.8193
2.8067
2.7940
2.7814
2.6010
2.5196
2.4340
2.4058
2.2934
1.8361
1.8308
1.8268
1.8207
1.8104
1.8058
1.7220
1.7118
1.7005
1.6489
1.6392
1.3321
1.3194
1.3067
lt–2471
1H
CDCl3
O
N
N
N
Et
11
10
9
8
7
6
5
4
3
2
1
0
ppm
1.0196
1.0000
1.0640
3.0906
1.0830
1.0915
2.0217
1.0007
1.0413
2.0407
1.0925
1.0888
1.0381
1.0141
2.0260
1.0433
1.0732
3.0611

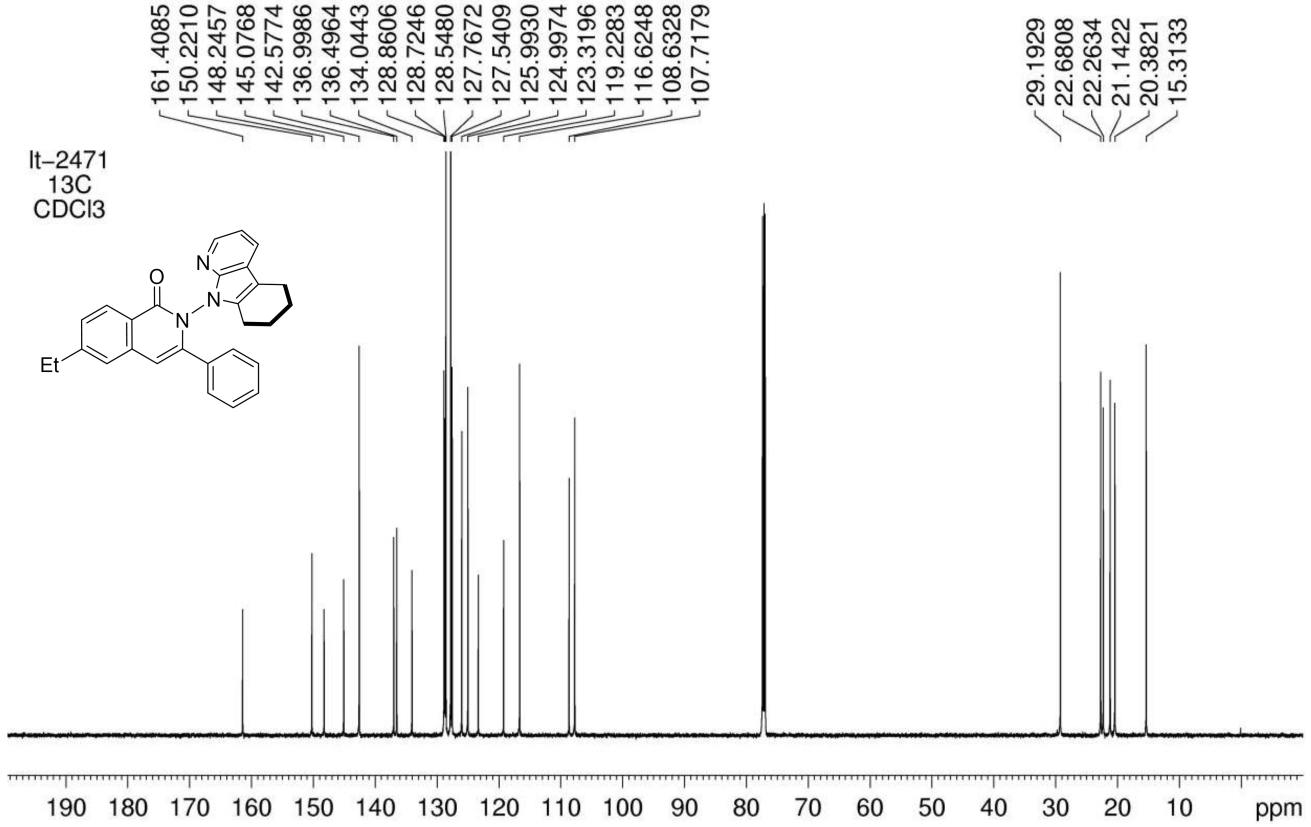
161.4085
150.2210
148.2457
145.0768
142.5774
136.9986
136.4964
134.0443
128.8606
128.7246
128.5480
127.7672
127.5409
125.9930
124.9974
123.3196
119.2283
116.6248
108.6328
107.7179
29.1929
22.6808
22.2634
21.1422
20.3821
15.3133
lt–2471
13C
CDCl3
O
N
N
N
Et
190
180
170
160
150
140
130
120
110
100
90
80
70
60
50
40
30
20
10
ppm

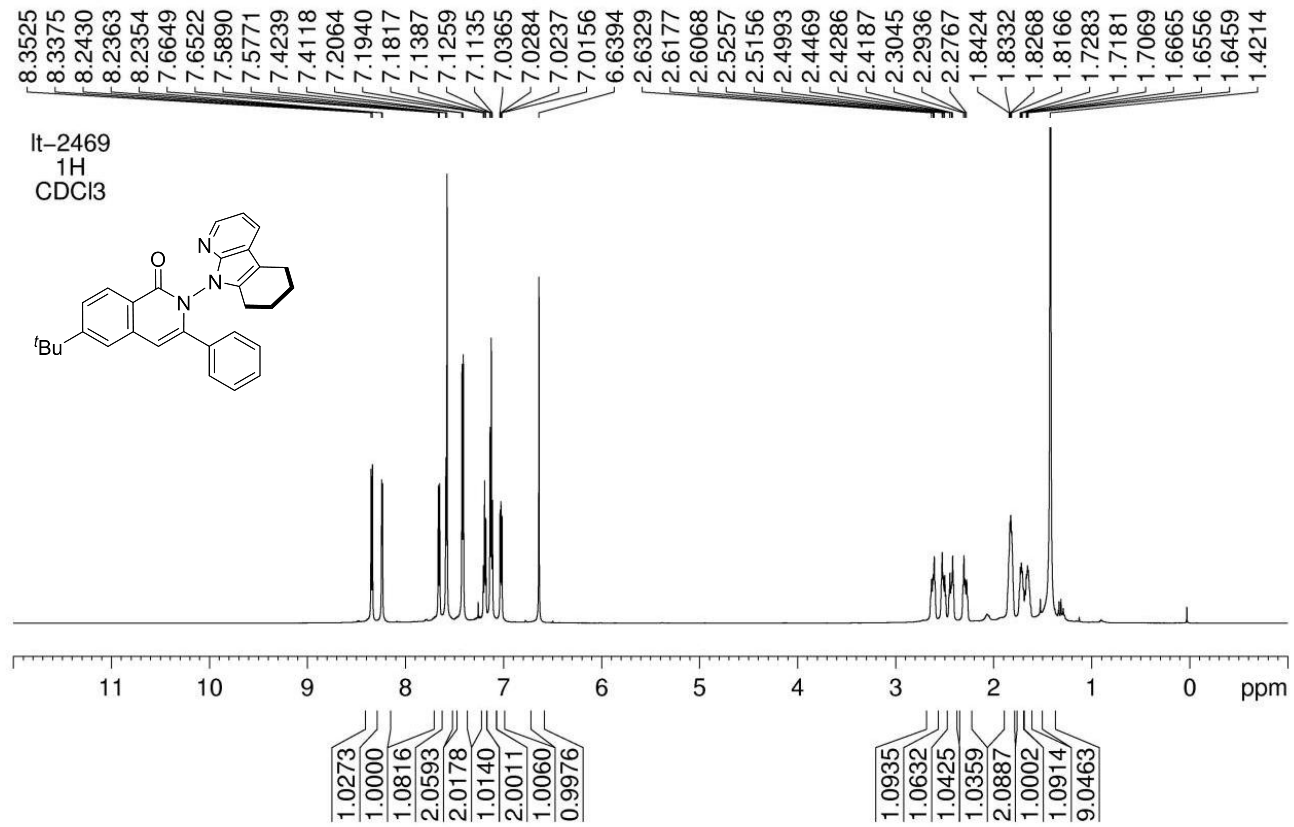
lt−2469
1H
CDCl3
O
N
N
N
tBu
8.3525
8.3375
8.2430
8.2363
8.2354
7.6649
7.6522
7.5890
7.5771
7.4239
7.4118
7.2064
7.1940
7.1817
7.1387
7.1259
7.1135
7.0365
7.0284
7.0237
7.0156
6.6394
2.6329
2.6177
2.6068
2.5257
2.5156
2.4993
2.4469
2.4286
2.4187
2.3045
2.2936
2.2767
1.8424
1.8332
1.8268
1.8166
1.7283
1.7181
1.7069
1.6665
1.6556
1.6459
1.4214
1.0273
1.0000
1.0816
2.0593
2.0178
1.0140
2.0011
1.0060
0.9976
1.0935
1.0632
1.0425
1.0359
2.0887
1.0002
1.0914
9.0463
ppm

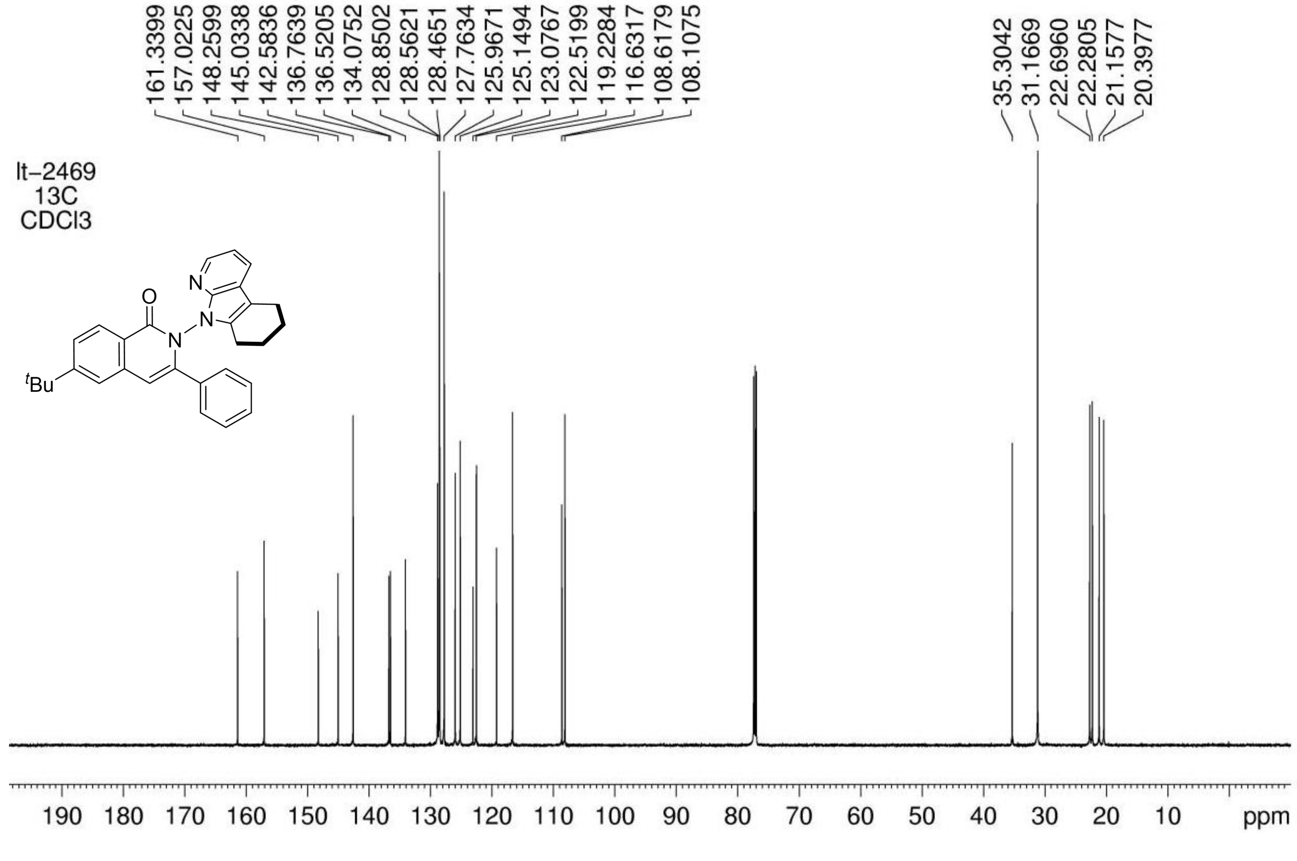
lt−2469
13C
CDCl3
O
N
N
N
tBu
161.3399
157.0225
148.2599
145.0338
142.5836
136.7639
136.5205
134.0752
128.8502
128.5621
128.4651
127.7634
125.9671
125.1494
123.0767
122.5199
119.2284
116.6317
108.6179
108.1075
35.3042
31.1669
22.6960
22.2805
21.1577
20.3977
ppm

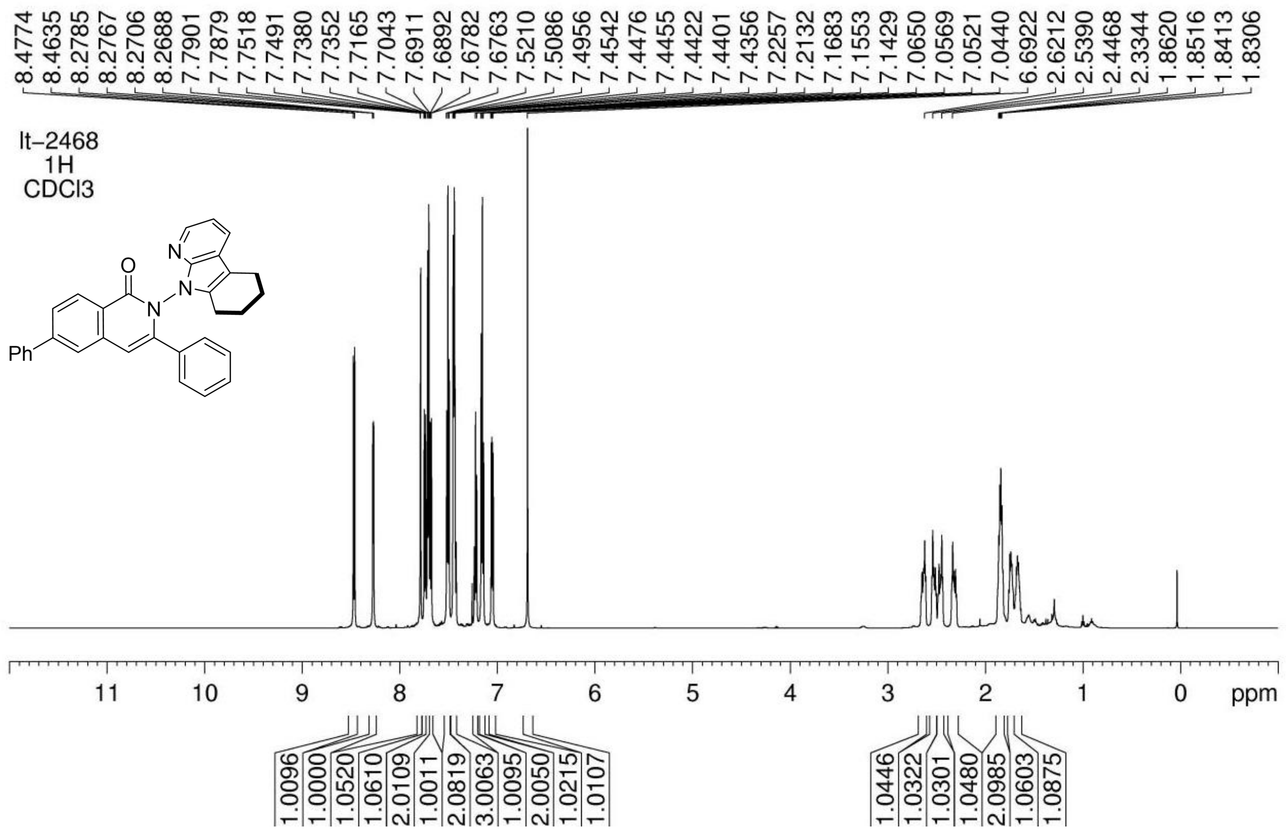
8.4774
8.4635
8.2785
8.2767
8.2706
8.2688
7.7901
7.7879
7.7518
7.7491
7.7380
7.7352
7.7165
7.7043
7.6911
7.6892
7.6782
7.6763
7.5210
7.5086
7.4956
7.4542
7.4476
7.4455
7.4422
7.4401
7.4356
7.2257
7.2132
7.1683
7.1553
7.1429
7.0650
7.0569
7.0521
7.0440
6.6922
2.6212
2.5390
2.4468
2.3344
1.8620
1.8516
1.8413
1.8306
lt–2468
1H
CDCl3
O
N
N
N
Ph
11 10 9 8 7 6 5 4 3 2 1 0 ppm
1.0096
1.0000
1.0520
1.0610
2.0109
1.0011
2.0819
3.0063
1.0095
2.0050
1.0215
1.0107
1.0446
1.0322
1.0301
1.0480
2.0985
1.0603
1.0875

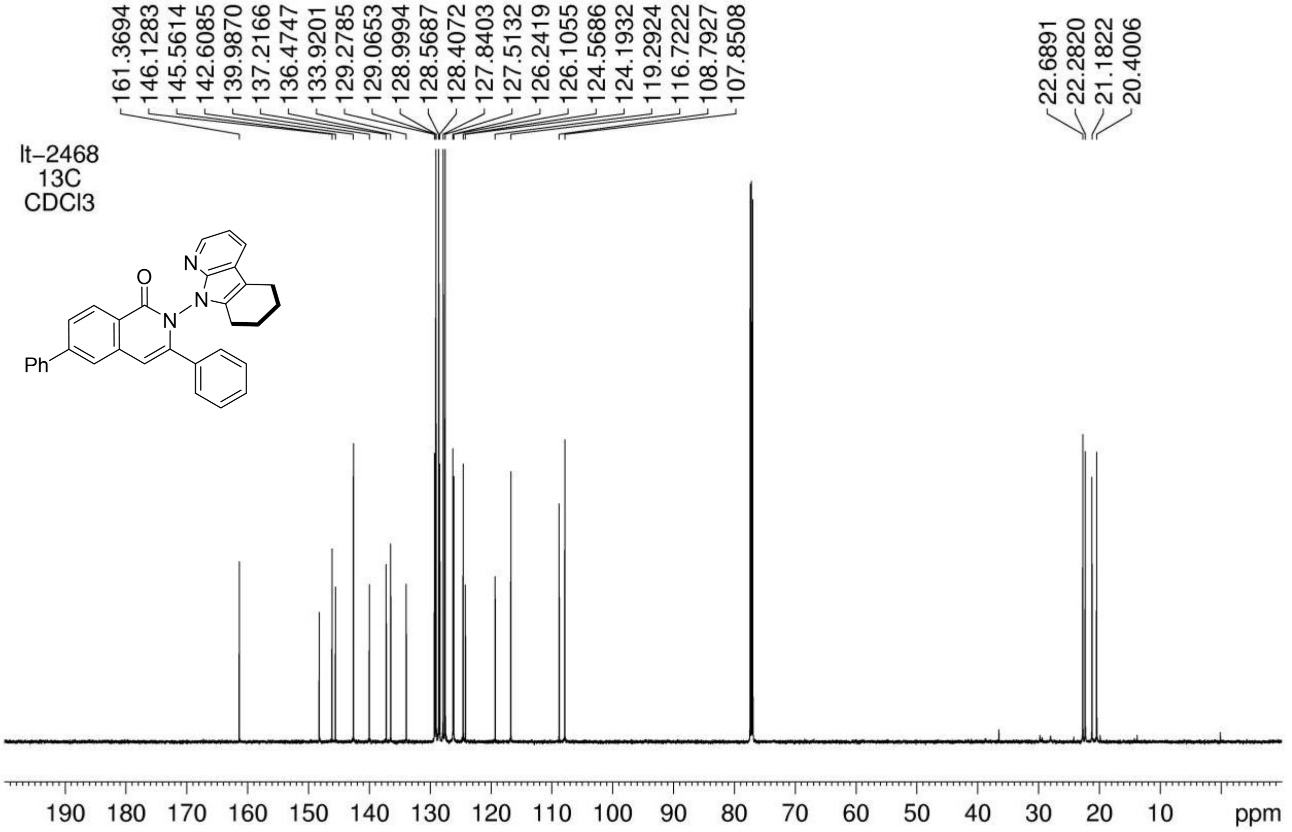
161.3694
146.1283
145.5614
142.6085
139.9870
137.2166
136.4747
133.9201
129.2785
129.0653
128.9994
128.5687
128.4072
127.8403
127.5132
126.2419
126.1055
124.5686
124.1932
119.2924
116.7222
108.7927
107.8508
22.6891
22.2820
21.1822
20.4006
lt–2468
13C
CDCl3
O
N
N
N
Ph
190 180 170 160 150 140 130 120 110 100 90 80 70 60 50 40 30 20 10 ppm

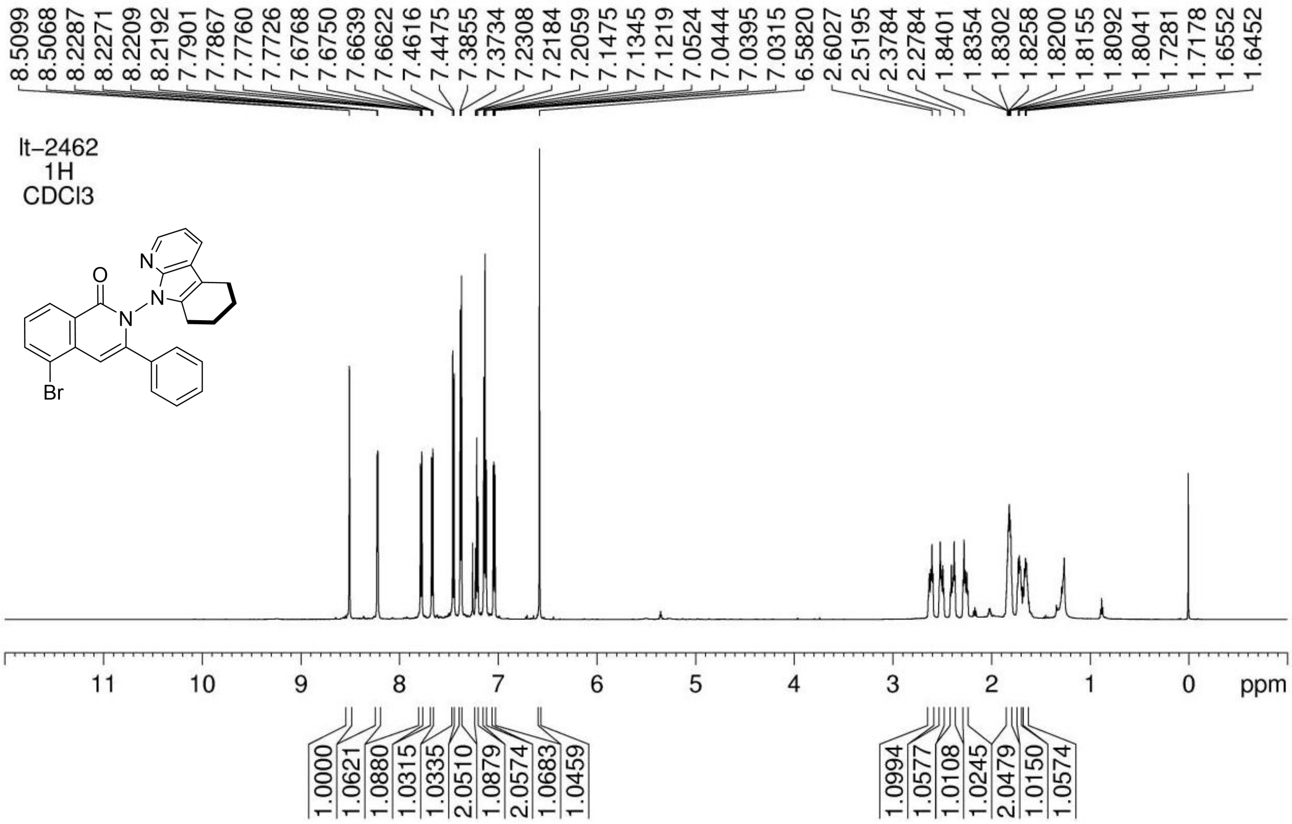
8.5099
8.5068
8.2287
8.2271
8.2209
8.2192
7.7901
7.7867
7.7760
7.7726
7.6768
7.6750
7.6639
7.6622
7.4616
7.4475
7.3855
7.3734
7.2308
7.2184
7.2059
7.1475
7.1345
7.1219
7.0524
7.0444
7.0395
7.0315
6.5820
2.6027
2.5195
2.3784
2.2784
1.8401
1.8354
1.8302
1.8258
1.8200
1.8155
1.8092
1.8041
1.7281
1.7178
1.6552
1.6452
lt–2462
1H
CDCl3
N
O
N
N
Br
11
10
9
8
7
6
5
4
3
2
1
0
ppm
1.0000
1.0621
1.0880
1.0315
1.0335
2.0510
1.0879
2.0574
1.0683
1.0459
1.0994
1.0577
1.0108
1.0245
2.0479
1.0150
1.0574

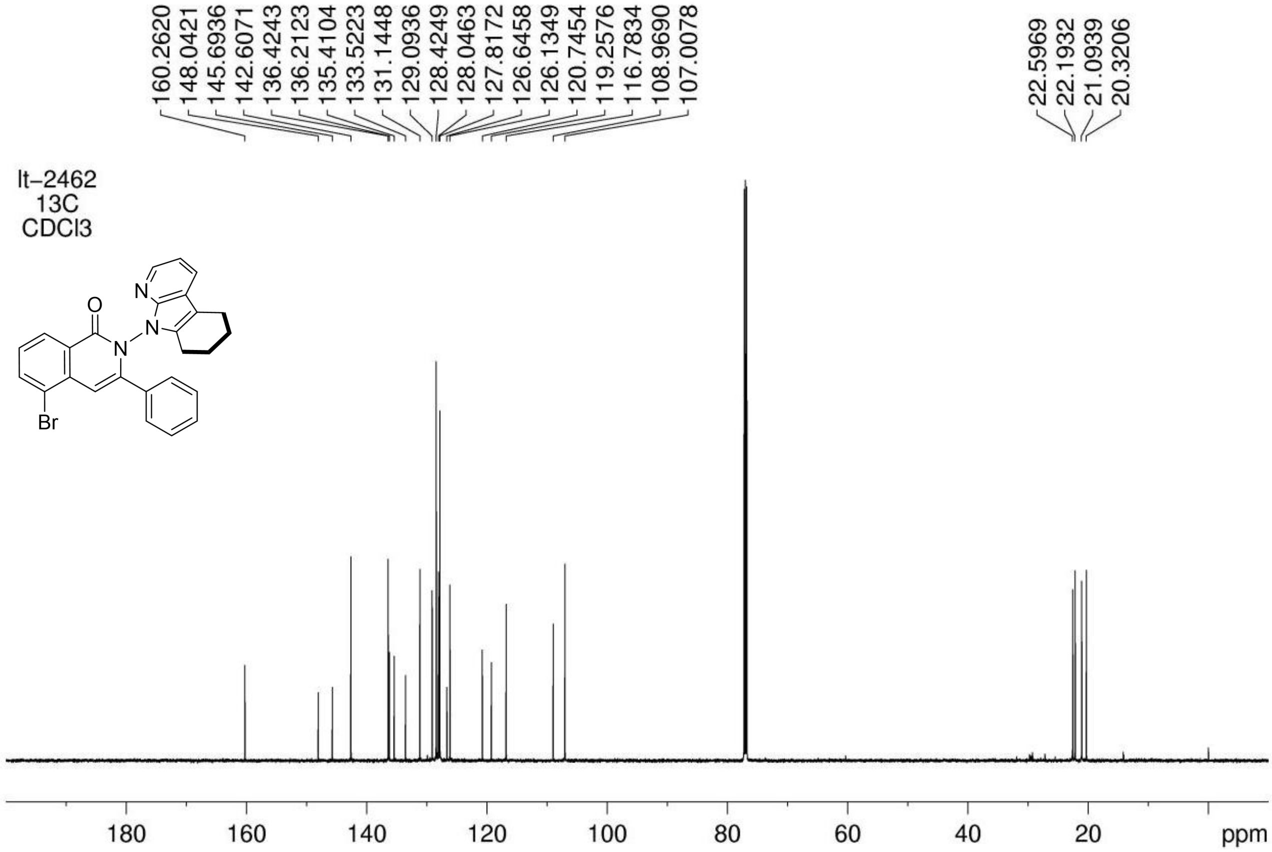
160.2620
148.0421
145.6936
142.6071
136.4243
136.2123
135.4104
133.5223
131.1448
129.0936
128.4249
128.0463
127.8172
126.6458
126.1349
120.7454
119.2576
116.7834
108.9690
107.0078
22.5969
22.1932
21.0939
20.3206
lt–2462
13C
CDCl3
N
O
N
N
Br
180
160
140
120
100
80
60
40
20
ppm

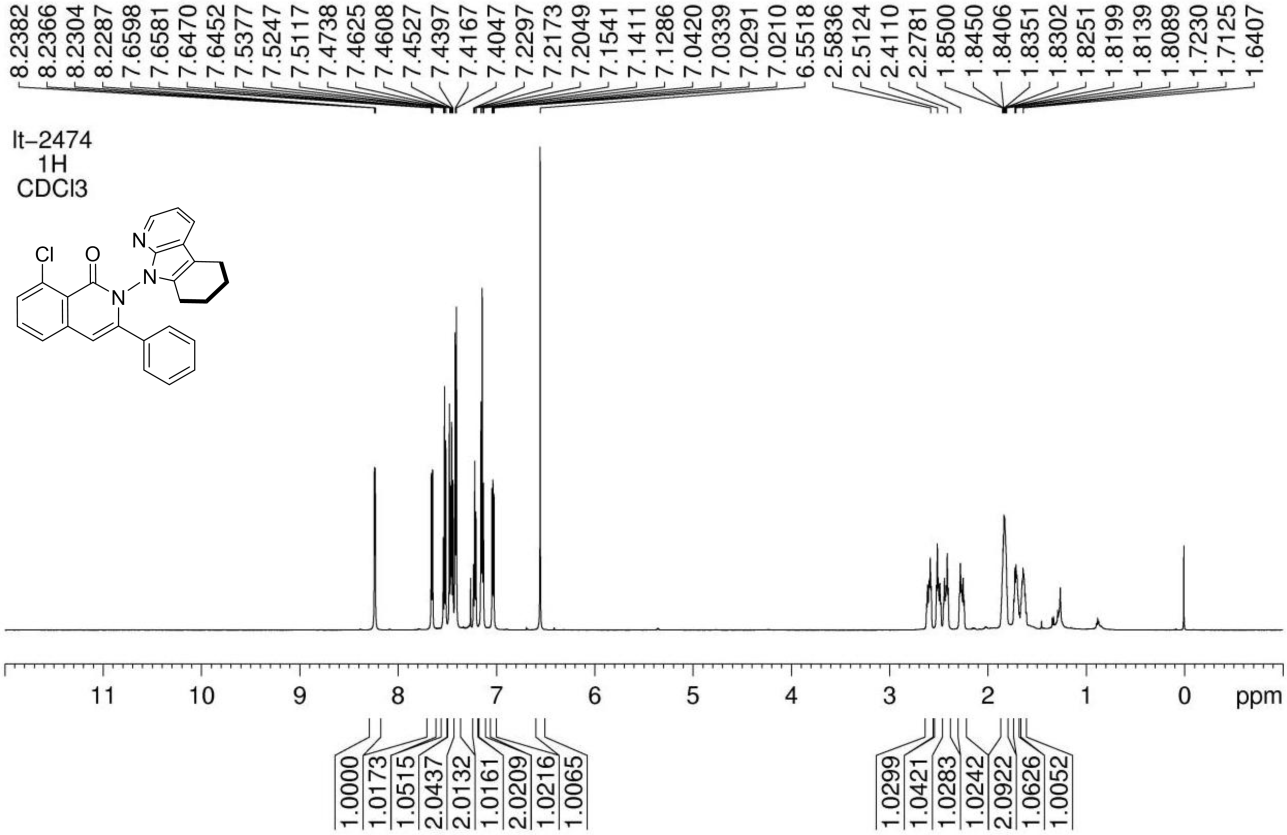
8.2382 8.2366 8.2304 8.2287 7.6598 7.6581 7.6470 7.6452 7.5377 7.5247 7.5117 7.4738 7.4625 7.4608 7.4527 7.4397 7.4167 7.4047 7.2297 7.2173 7.2049 7.1541 7.1411 7.1286 7.0420 7.0339 7.0291 7.0210 6.5518 2.5836 2.5124 2.4110 2.2781 1.8500 1.8450 1.8406 1.8351 1.8302 1.8251 1.8199 1.8139 1.8089 1.7230 1.7125 1.6407
lt–2474
1H
CDCl3
Cl
O
N
N
N
11 10 9 8 7 6 5 4 3 2 1 0 ppm
1.0000 1.0173 1.0515 2.0437 2.0132 1.0161 2.0209 1.0216 1.0065
1.0299 1.0421 1.0283 1.0242 2.0922 1.0626 1.0052

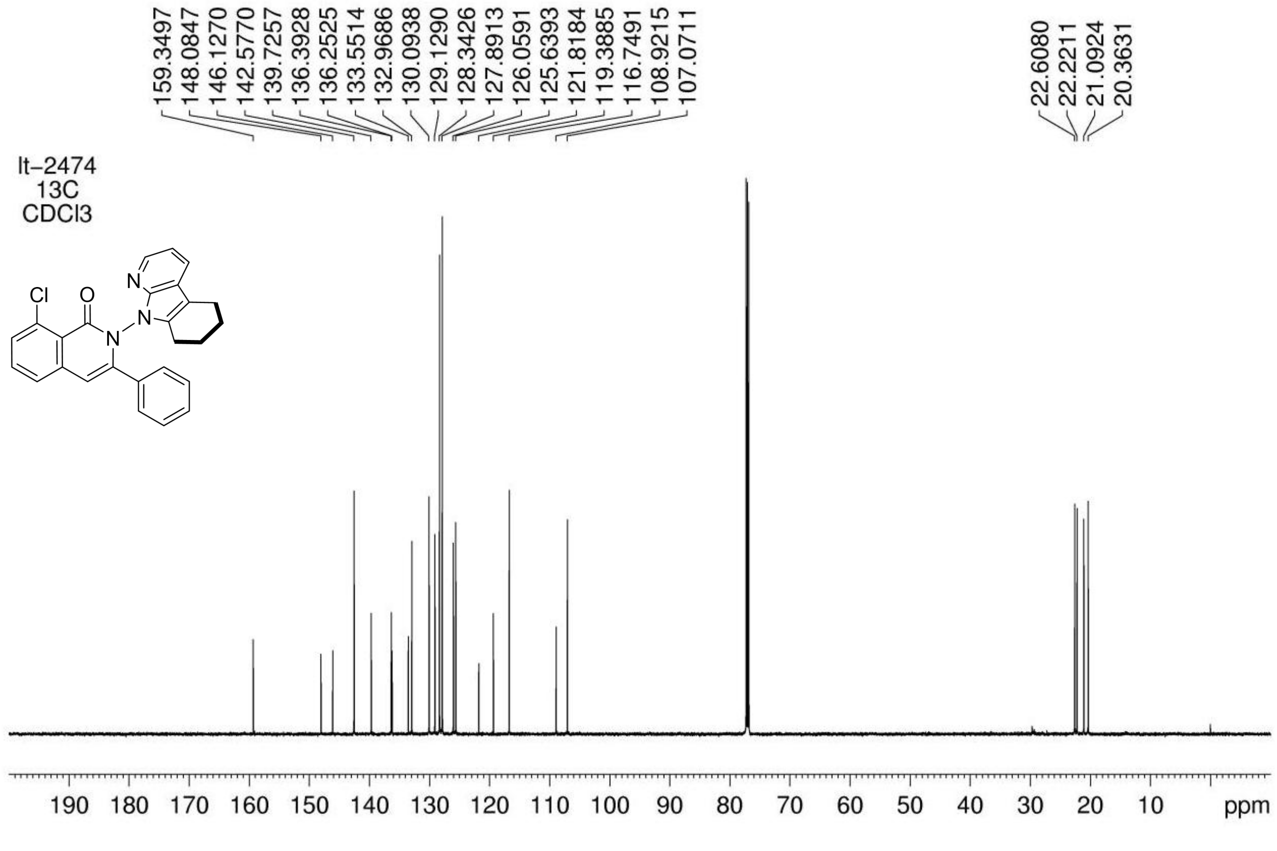
159.3497 148.0847 146.1270 142.5770 139.7257 136.3928 136.2525 133.5514 132.9686 130.0938 129.1290 128.3426 127.8913 126.0591 125.6393 121.8184 119.3885 116.7491 108.9215 107.0711
22.6080 22.2211 21.0924 20.3631
lt–2474
13C
CDCl3
Cl
O
N
N
N
190 180 170 160 150 140 130 120 110 100 90 80 70 60 50 40 30 20 10 ppm

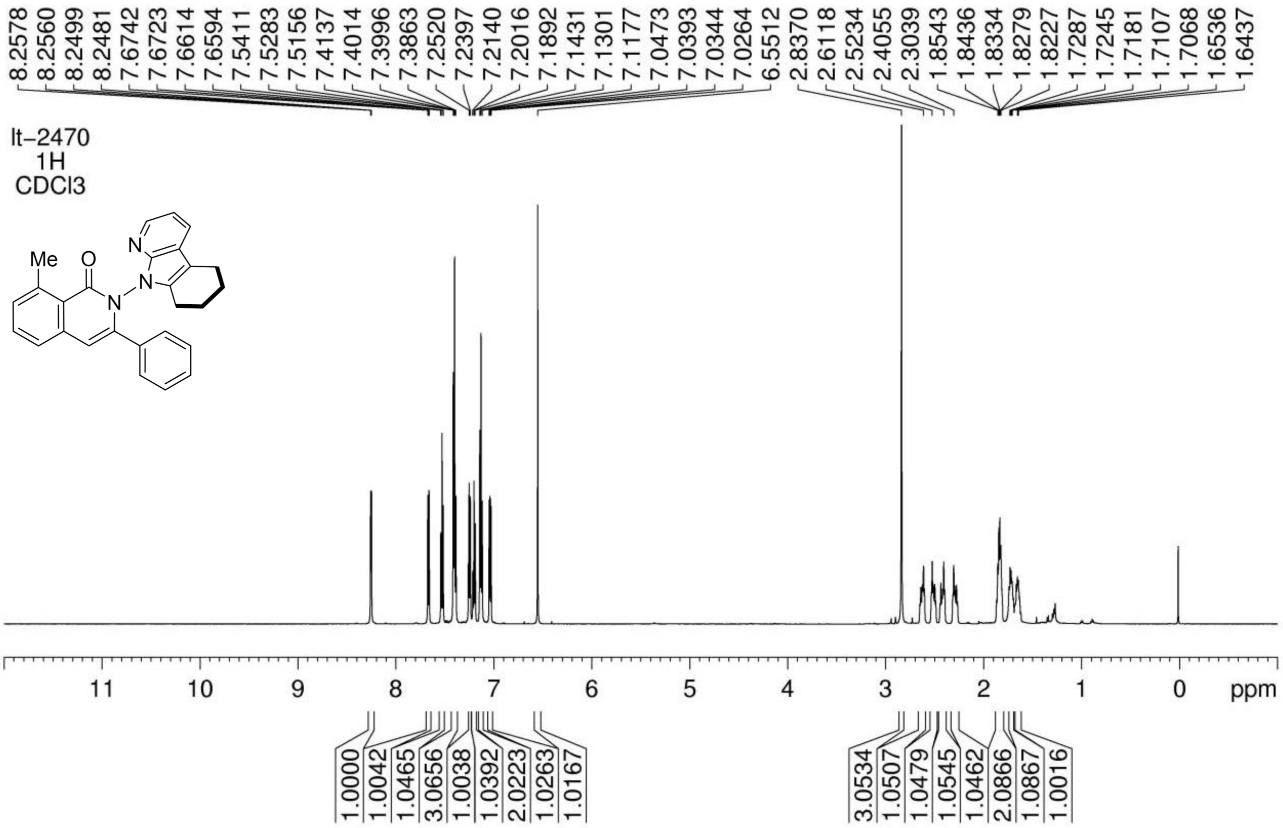
8.2578
8.2560
8.2499
8.2481
7.6742
7.6723
7.6614
7.6594
7.5411
7.5283
7.5156
7.4137
7.4014
7.3996
7.3863
7.2520
7.2397
7.2140
7.2016
7.1892
7.1431
7.1301
7.1177
7.0473
7.0393
7.0344
7.0264
6.5512
2.8370
2.6118
2.5234
2.4055
2.3039
1.8543
1.8436
1.8334
1.8279
1.8227
1.7287
1.7245
1.7181
1.7107
1.7068
1.6536
1.6437
lt–2470
1H
CDCl3
Me
O
N
N
N
11
10
9
8
7
6
5
4
3
2
1
0
ppm
1.0000
1.0042
1.0465
3.0656
1.0038
1.0392
2.0223
1.0263
1.0167
3.0534
1.0507
1.0479
1.0545
1.0462
2.0866
1.0867
1.0016

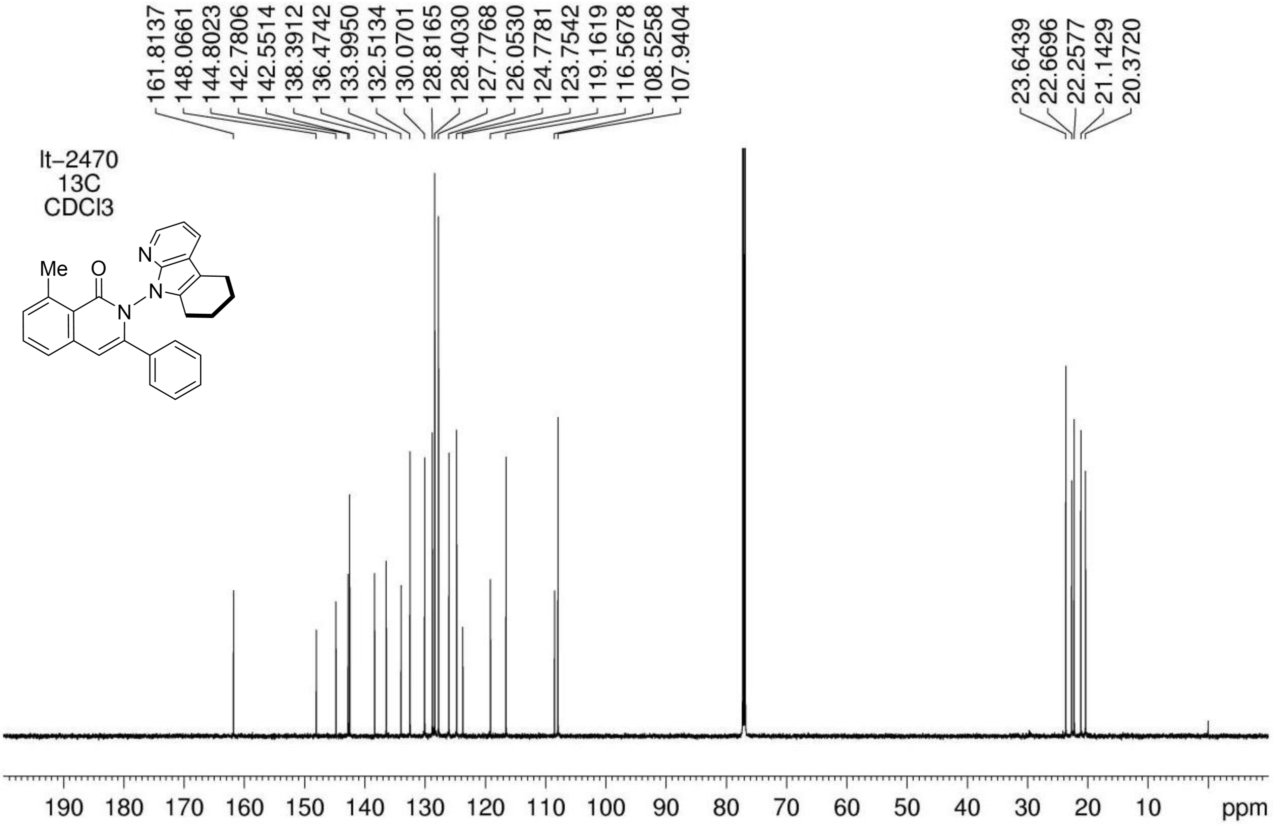
161.8137
148.0661
144.8023
142.7806
142.5514
138.3912
136.4742
133.9950
132.5134
130.0701
128.8165
128.4030
127.7768
126.0530
124.7781
123.7542
119.1619
116.5678
108.5258
107.9404
23.6439
22.6696
22.2577
21.1429
20.3720
lt–2470
13C
CDCl3
Me
O
N
N
N
190
180
170
160
150
140
130
120
110
100
90
80
70
60
50
40
30
20
10
ppm

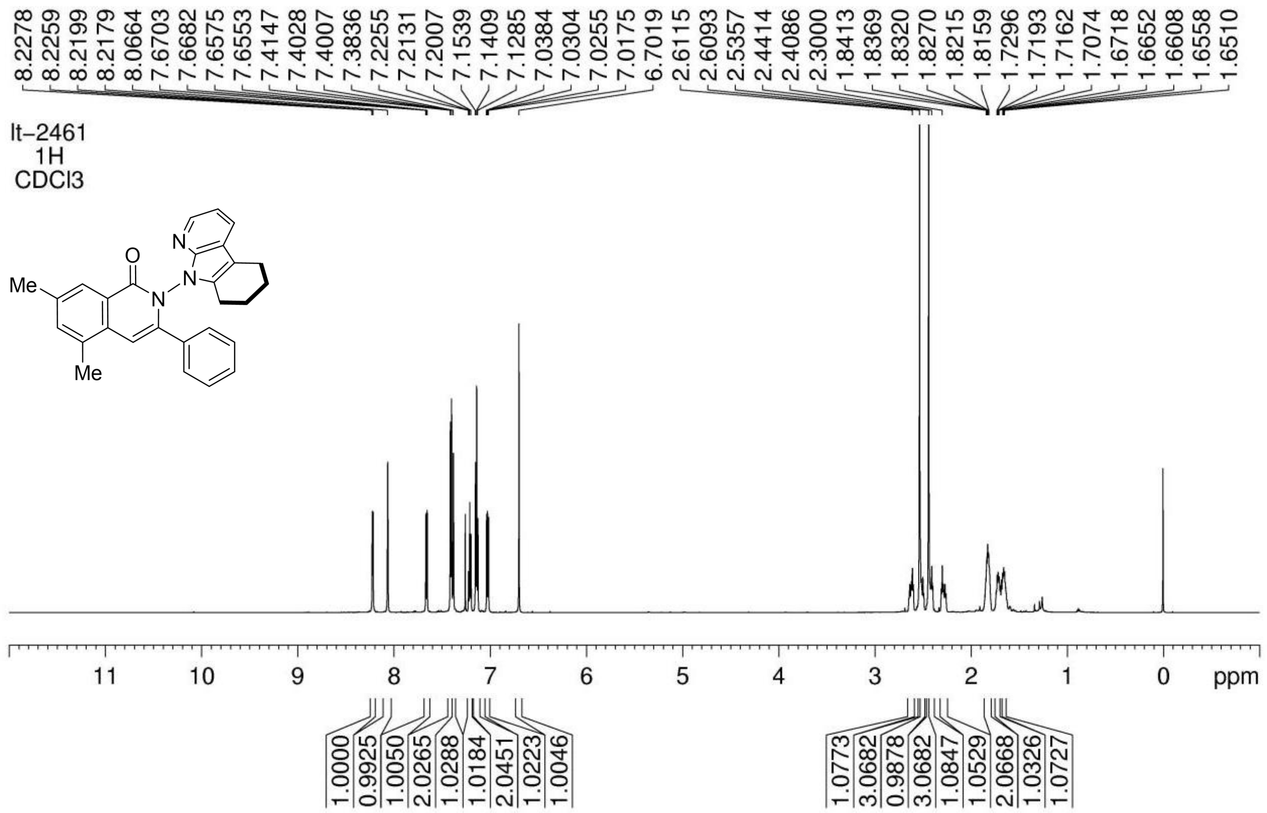
8.2278
8.2259
8.2199
8.2179
8.0664
7.6703
7.6682
7.6575
7.6553
7.4147
7.4028
7.4007
7.3836
7.2255
7.2131
7.2007
7.1539
7.1409
7.1285
7.0384
7.0304
7.0255
7.0175
6.7019
2.6115
2.6093
2.5357
2.4414
2.4086
2.3000
1.8413
1.8369
1.8320
1.8270
1.8215
1.8159
1.7296
1.7193
1.7162
1.7074
1.6718
1.6652
1.6608
1.6558
1.6510
lt–2461
1H
CDCl3
Me
Me
O
N
N
N
11 10 9 8 7 6 5 4 3 2 1 0 ppm
1.0000
0.9925
1.0050
2.0265
1.0288
1.0184
2.0451
1.0223
1.0046
1.0773
3.0682
0.9878
3.0682
1.0847
1.0529
2.0668
1.0326
1.0727

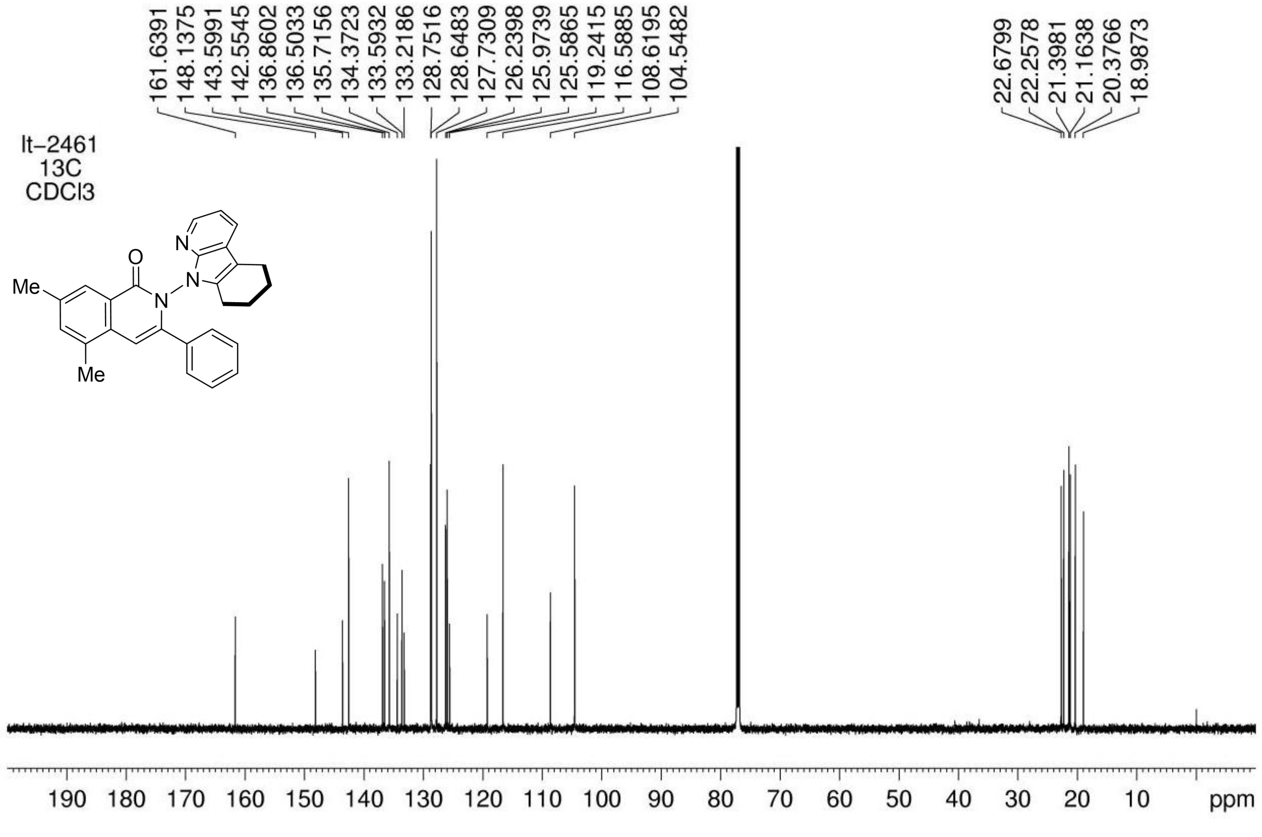
161.6391
148.1375
143.5991
142.5545
136.8602
136.5033
135.7156
134.3723
133.5932
133.2186
128.7516
128.6483
127.7309
126.2398
125.9739
125.5865
119.2415
116.5885
108.6195
104.5482
22.6799
22.2578
21.3981
21.1638
20.3766
18.9873
lt–2461
13C
CDCl3
Me
Me
O
N
N
N
190 180 170 160 150 140 130 120 110 100 90 80 70 60 50 40 30 20 10 ppm

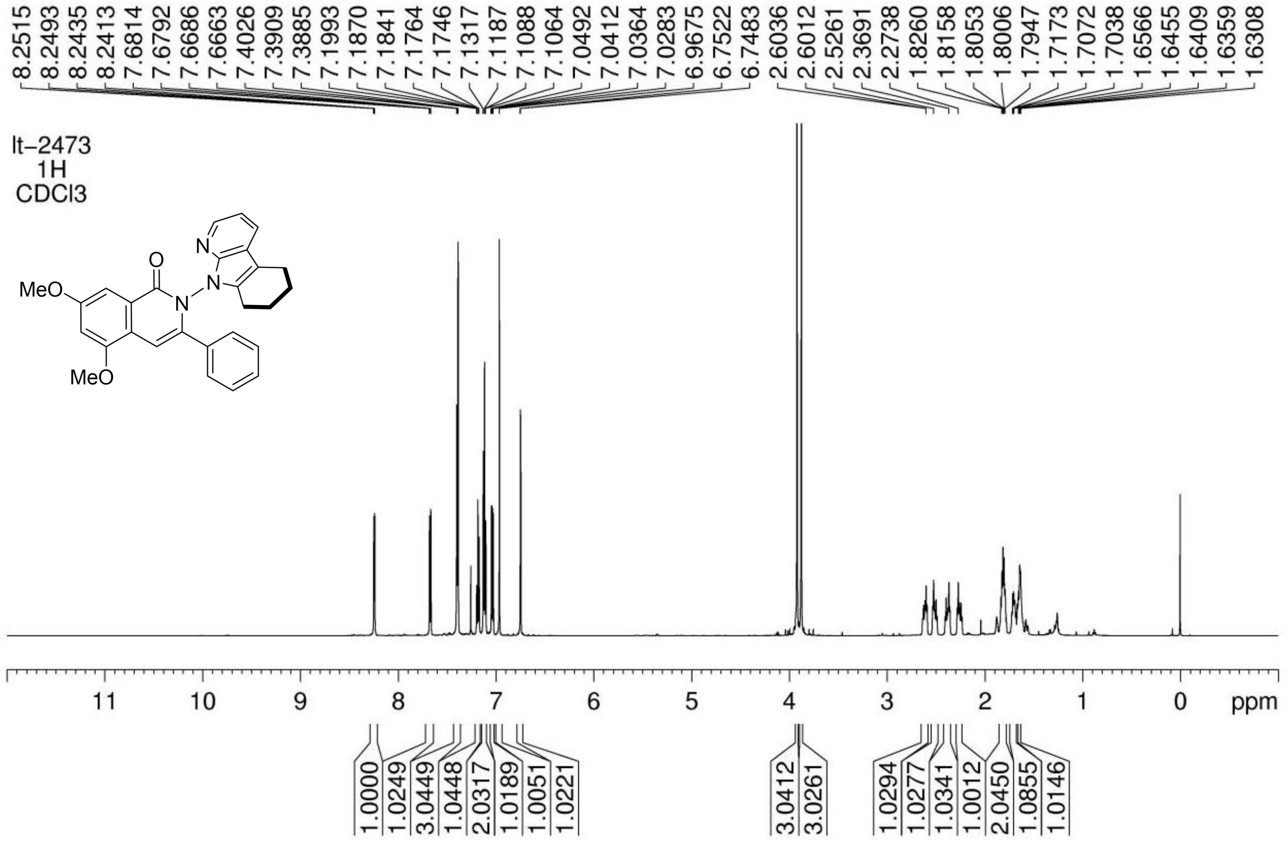
8.2515
8.2493
8.2435
8.2413
7.6814
7.6792
7.6686
7.6663
7.4026
7.3909
7.3885
7.1993
7.1870
7.1841
7.1764
7.1746
7.1317
7.1187
7.1088
7.1064
7.0492
7.0412
7.0364
7.0283
6.9675
6.7522
6.7483
2.6036
2.6012
2.5261
2.3691
2.2738
1.8260
1.8158
1.8053
1.8006
1.7947
1.7173
1.7072
1.7038
1.6566
1.6455
1.6409
1.6359
1.6308
lt-2473
1H
CDCl3
O
N
N
N
MeO
MeO
11
10
9
8
7
6
5
4
3
2
1
0
ppm
1.0000
1.0249
3.0449
1.0448
2.0317
1.0189
1.0051
1.0221
3.0412
3.0261
1.0294
1.0277
1.0341
1.0012
2.0450
1.0855
1.0146

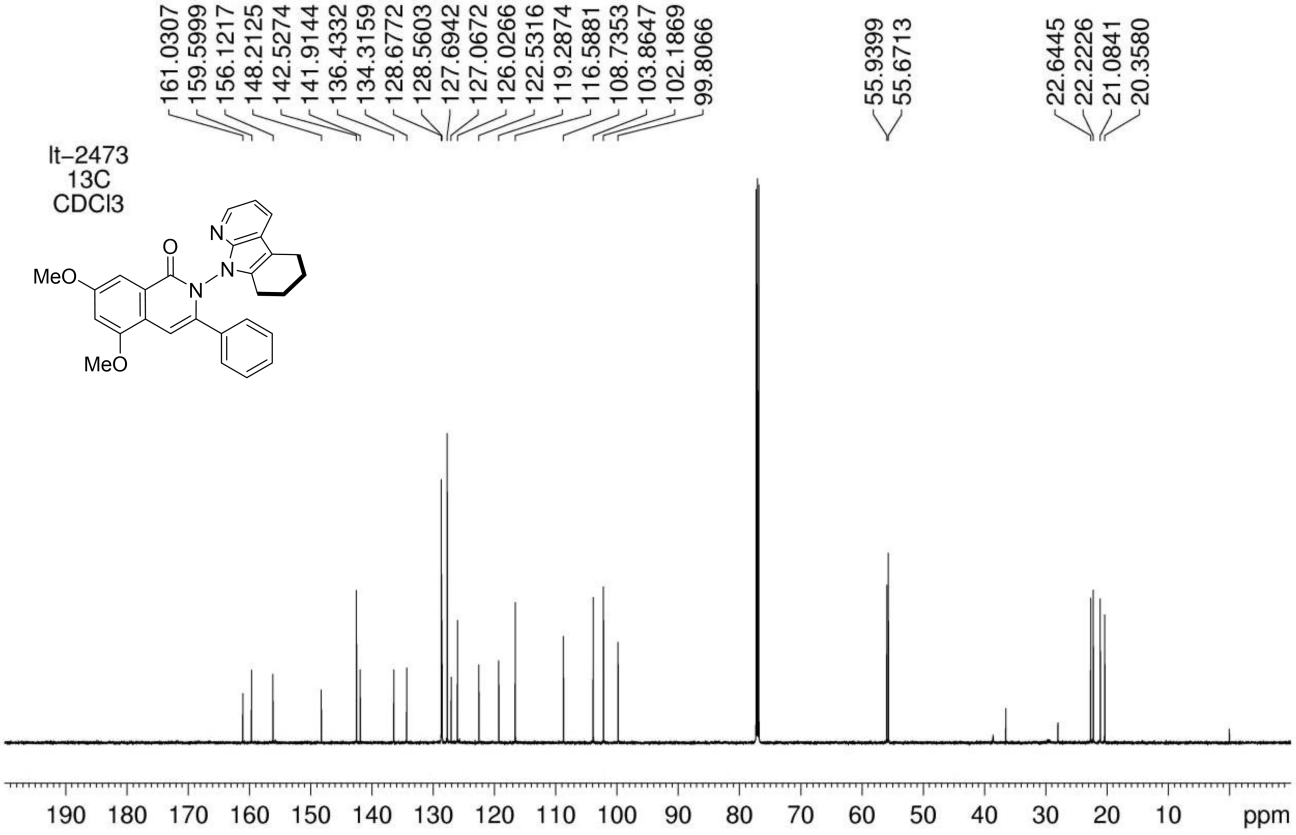
161.0307
159.5999
156.1217
148.2125
142.5274
141.9144
136.4332
134.3159
128.6772
128.5603
127.6942
127.0672
126.0266
122.5316
119.2874
116.5881
108.7353
103.8647
102.1869
99.8066
55.9399
55.6713
22.6445
22.2226
21.0841
20.3580
lt-2473
13C
CDCl3
O
N
N
N
MeO
MeO
190
180
170
160
150
140
130
120
110
100
90
80
70
60
50
40
30
20
10
ppm

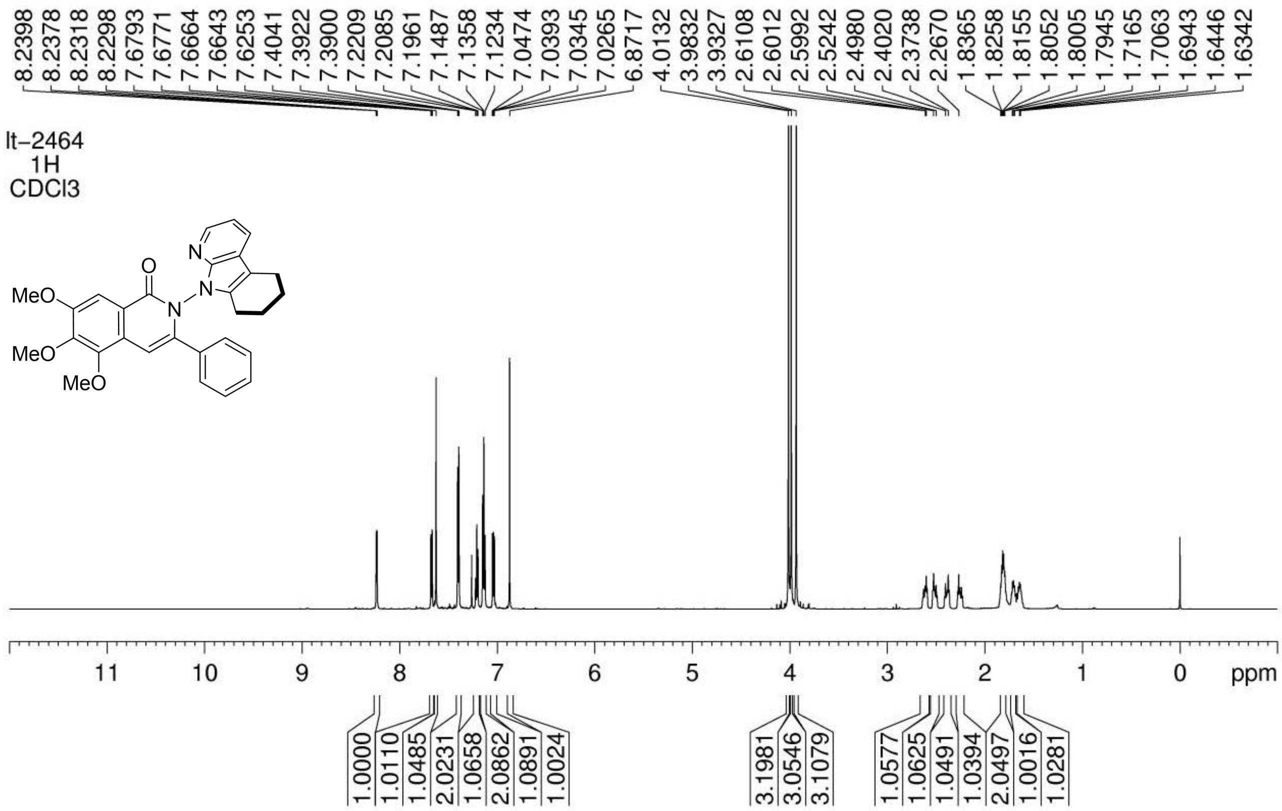
8.2398
8.2378
8.2318
8.2298
7.6793
7.6771
7.6664
7.6643
7.6253
7.4041
7.3922
7.3900
7.2209
7.2085
7.1961
7.1487
7.1358
7.1234
7.0474
7.0393
7.0345
7.0265
6.8717
4.0132
3.9832
3.9327
2.6108
2.6012
2.5992
2.5242
2.4980
2.4020
2.3738
2.2670
1.8365
1.8258
1.8155
1.8052
1.8005
1.7945
1.7165
1.7063
1.6943
1.6446
1.6342
lt–2464
1H
CDCl3
MeO
MeO
MeO
O
N
N
N
11 10 9 8 7 6 5 4 3 2 1 0 ppm
1.0000
1.0110
1.0485
2.0231
1.0658
2.0862
1.0891
1.0024
3.1981
3.0546
3.1079
1.0577
1.0625
1.0491
1.0394
2.0497
1.0016
1.0281

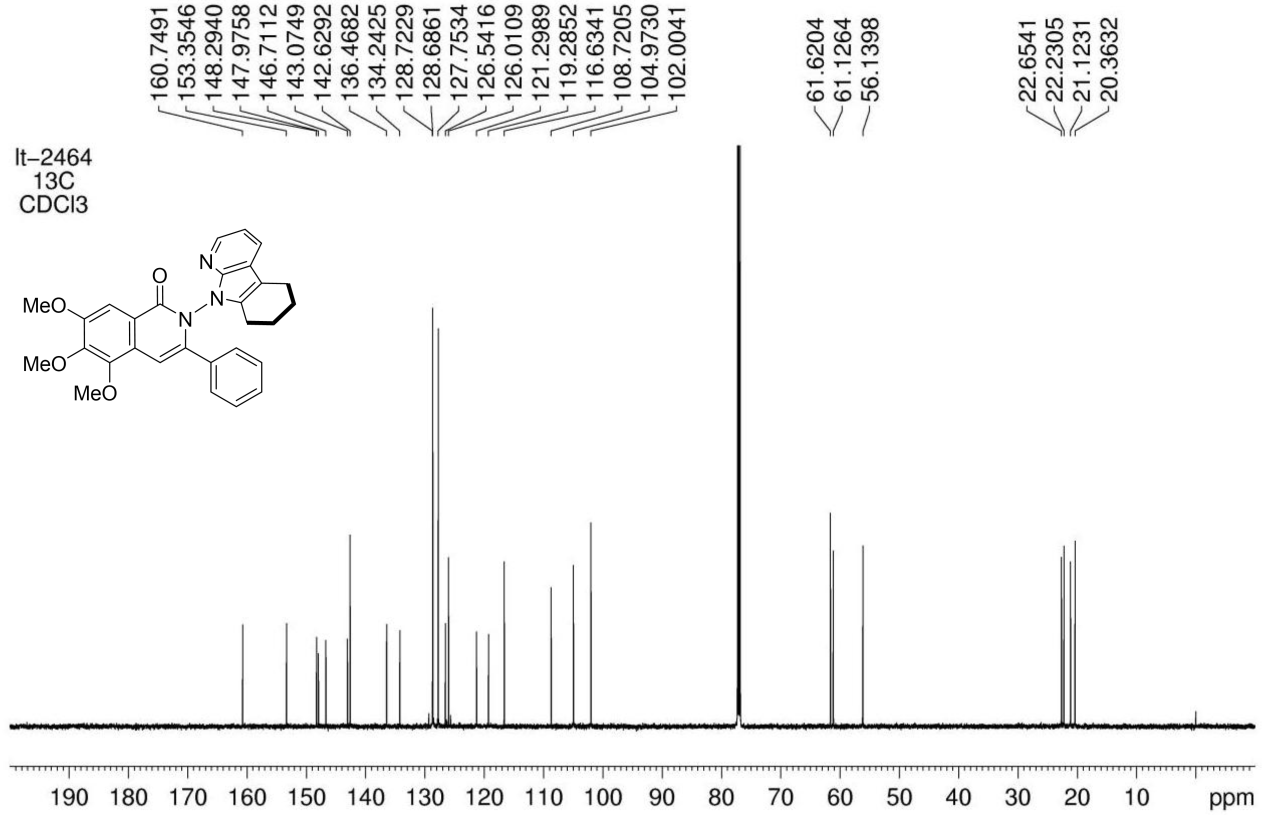
160.7491
153.3546
148.2940
147.9758
146.7112
143.0749
142.6292
136.4682
134.2425
128.7229
128.6861
127.7534
126.5416
126.0109
121.2989
119.2852
116.6341
108.7205
104.9730
102.0041
61.6204
61.1264
56.1398
22.6541
22.2305
21.1231
20.3632
lt–2464
13C
CDCl3
MeO
MeO
MeO
O
N
N
N
190 180 170 160 150 140 130 120 110 100 90 80 70 60 50 40 30 20 10 ppm

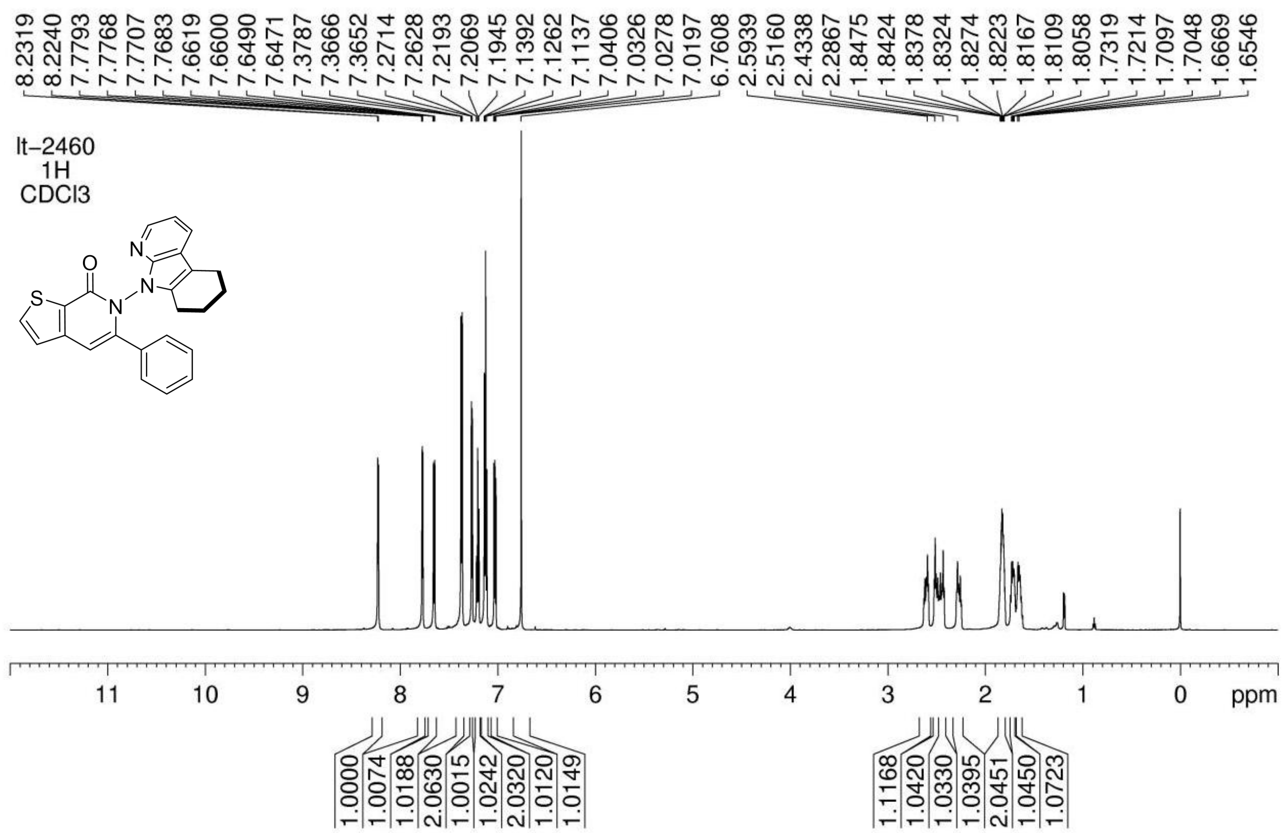
8.2319
8.2240
7.7793
7.7768
7.7707
7.7683
7.6619
7.6600
7.6490
7.6471
7.3787
7.3666
7.3652
7.2714
7.2628
7.2193
7.2069
7.1945
7.1392
7.1262
7.1137
7.0406
7.0326
7.0278
7.0197
6.7608
2.5939
2.5160
2.4338
2.2867
1.8475
1.8424
1.8378
1.8324
1.8274
1.8223
1.8167
1.8109
1.8058
1.7319
1.7214
1.7097
1.7048
1.6669
1.6546
lt−2460
1H
CDCl3
O
S
N
N
N
11
10
9
8
7
6
5
4
3
2
1
0
ppm
1.0000
1.0074
1.0188
2.0630
1.0015
1.0242
2.0320
1.0120
1.0149
1.1168
1.0420
1.0330
1.0395
2.0451
1.0450
1.0723

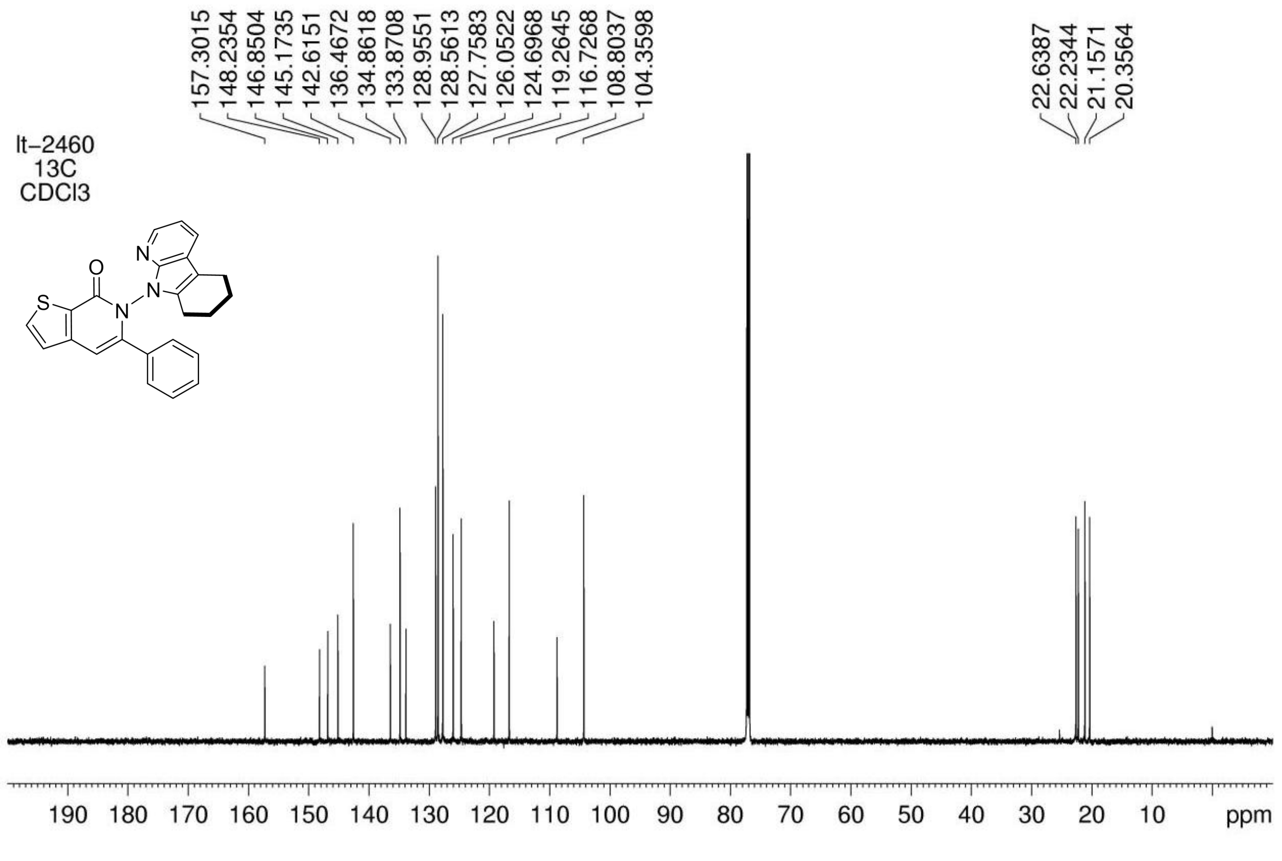
157.3015
148.2354
146.8504
145.1735
142.6151
136.4672
134.8618
133.8708
128.9551
128.5613
127.7583
126.0522
124.6968
119.2645
116.7268
108.8037
104.3598
22.6387
22.2344
21.1571
20.3564
lt−2460
13C
CDCl3
O
S
N
N
N
190
180
170
160
150
140
130
120
110
100
90
80
70
60
50
40
30
20
10
ppm

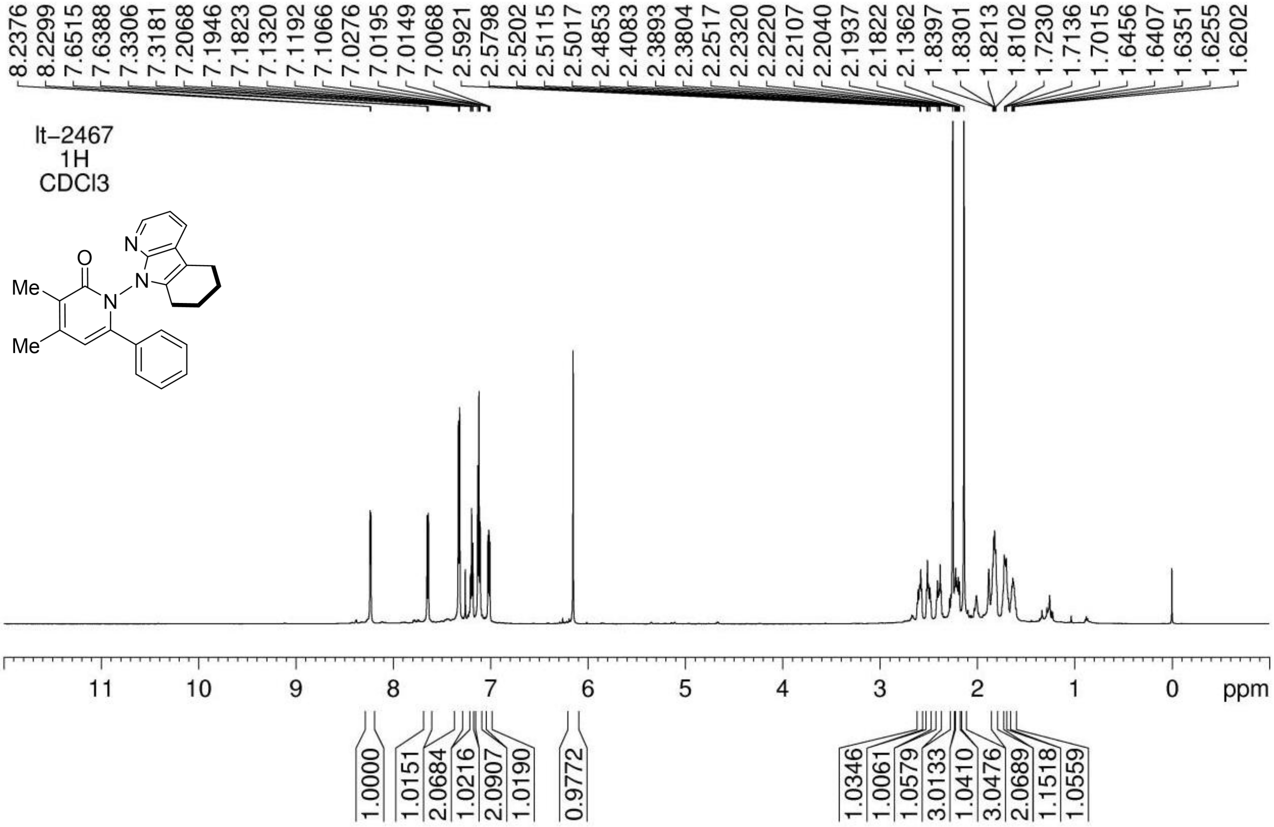
8.2376
8.2299
7.6515
7.6388
7.3306
7.3181
7.2068
7.1946
7.1823
7.1320
7.1192
7.1066
7.0276
7.0195
7.0149
7.0068
2.5921
2.5798
2.5202
2.5115
2.5017
2.4853
2.4083
2.3893
2.3804
2.2517
2.2320
2.2220
2.2107
2.2040
2.1937
2.1822
2.1362
1.8397
1.8301
1.8213
1.8102
1.7230
1.7136
1.7015
1.6456
1.6407
1.6351
1.6255
1.6202
lt–2467
1H
CDCl3
O
N
Me
Me
N
N
11 10 9 8 7 6 5 4 3 2 1 0 ppm
1.0000
1.0151
2.0684
1.0216
2.0907
1.0190
0.9772
1.0346
1.0061
1.0579
3.0133
1.0410
3.0476
2.0689
1.1518
1.0559

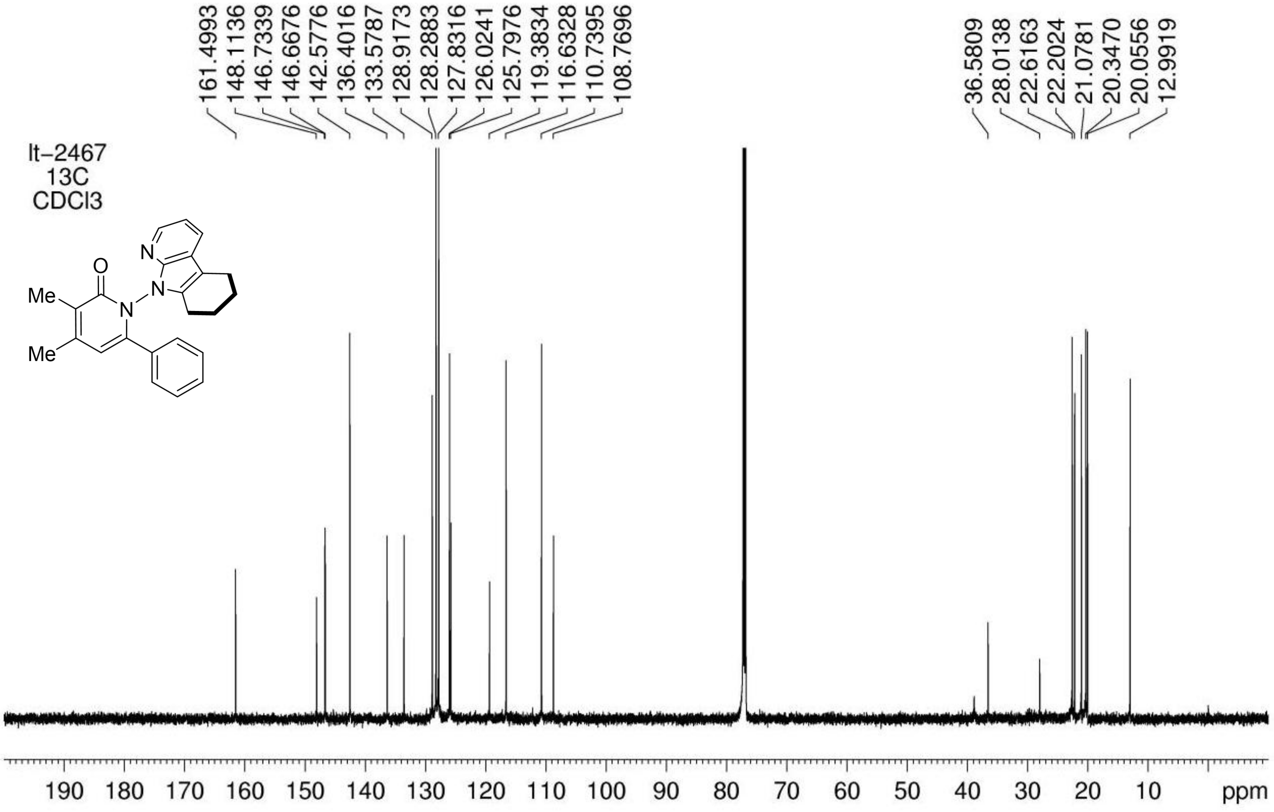
161.4993
148.1136
146.7339
146.6676
142.5776
136.4016
133.5787
128.9173
128.2883
127.8316
126.0241
125.7976
119.3834
116.6328
110.7395
108.7696
36.5809
28.0138
22.6163
22.2024
21.0781
20.3470
20.0556
12.9919
lt–2467
13C
CDCl3
O
N
Me
Me
N
N
190 180 170 160 150 140 130 120 110 100 90 80 70 60 50 40 30 20 10 ppm

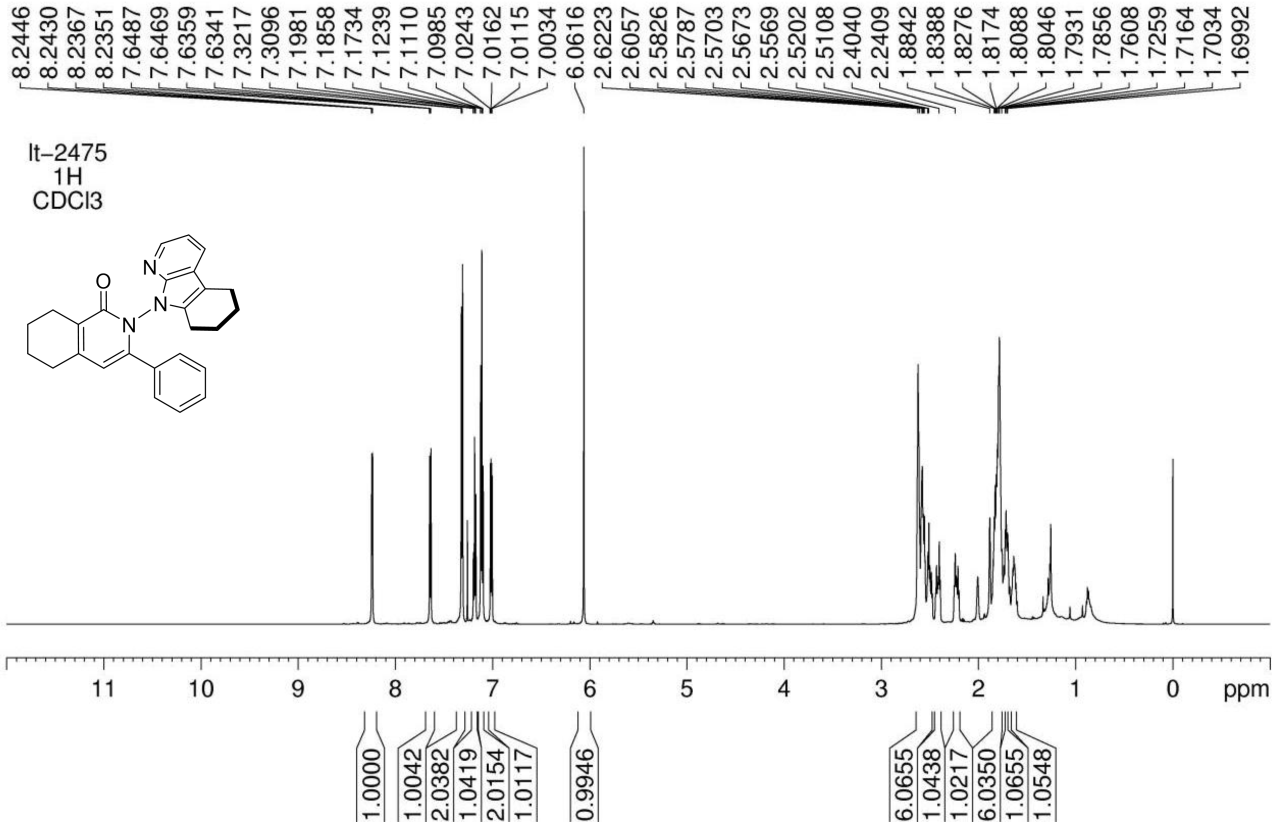
8.2446 8.2430 8.2367 8.2351 7.6487 7.6469 7.6359 7.6341 7.3217 7.3096 7.1981 7.1858 7.1734 7.1239 7.1110 7.0985 7.0243 7.0162 7.0115 7.0034 6.0616 2.6223 2.6057 2.5826 2.5787 2.5703 2.5673 2.5569 2.5202 2.5108 2.4040 2.2409 1.8842 1.8388 1.8276 1.8174 1.8088 1.8046 1.7931 1.7856 1.7608 1.7259 1.7164 1.7034 1.6992
lt–2475
1H
CDCl3
O
N
N
N
11 10 9 8 7 6 5 4 3 2 1 0 ppm
1.0000 1.0042 2.0382 1.0419 2.0154 1.0117 0.9946 6.0655 1.0438 1.0217 6.0350 1.0655 1.0548

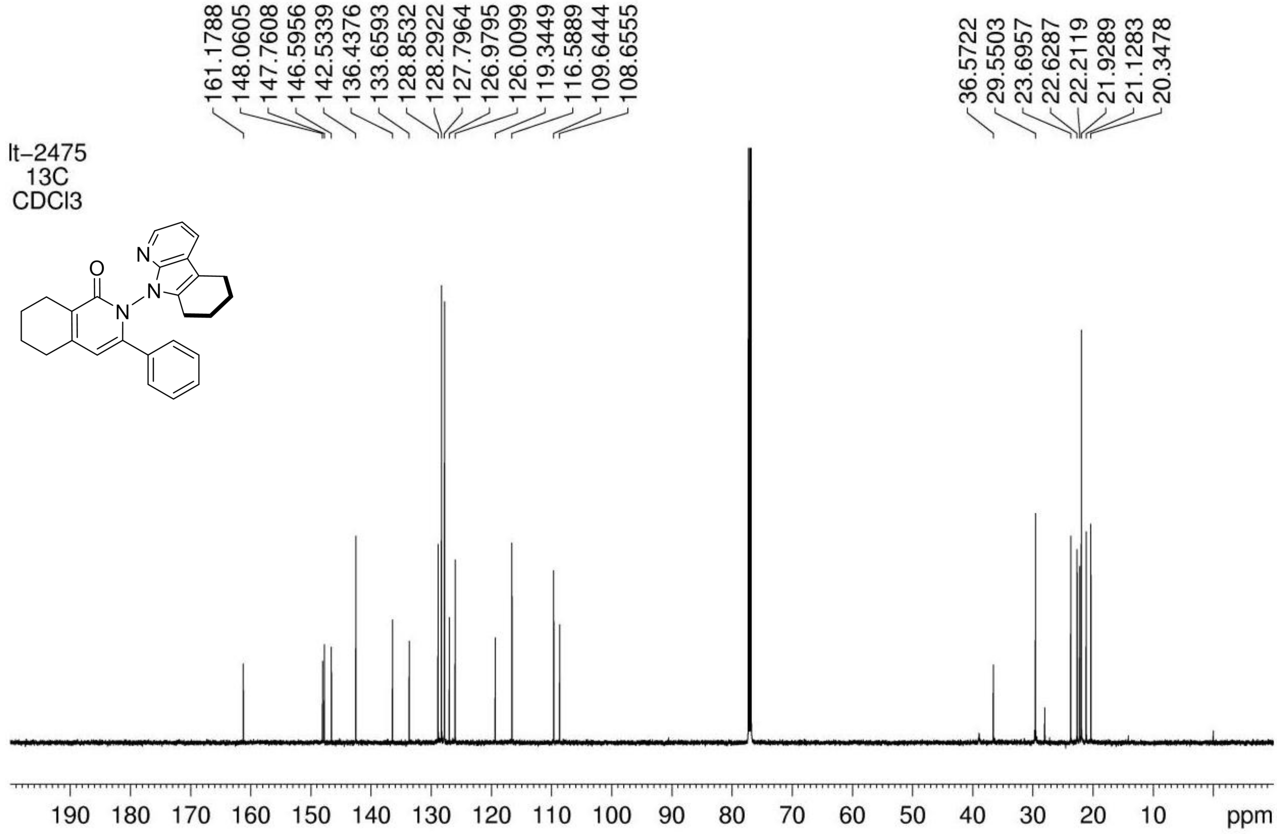
161.1788 148.0605 147.7608 146.5956 142.5339 136.4376 133.6593 128.8532 128.2922 127.7964 126.9795 126.0099 119.3449 116.5889 109.6444 108.6555
36.5722 29.5503 23.6957 22.6287 22.2119 21.9289 21.1283 20.3478
lt–2475
13C
CDCl3
O
N
N
N
190 180 170 160 150 140 130 120 110 100 90 80 70 60 50 40 30 20 10 ppm

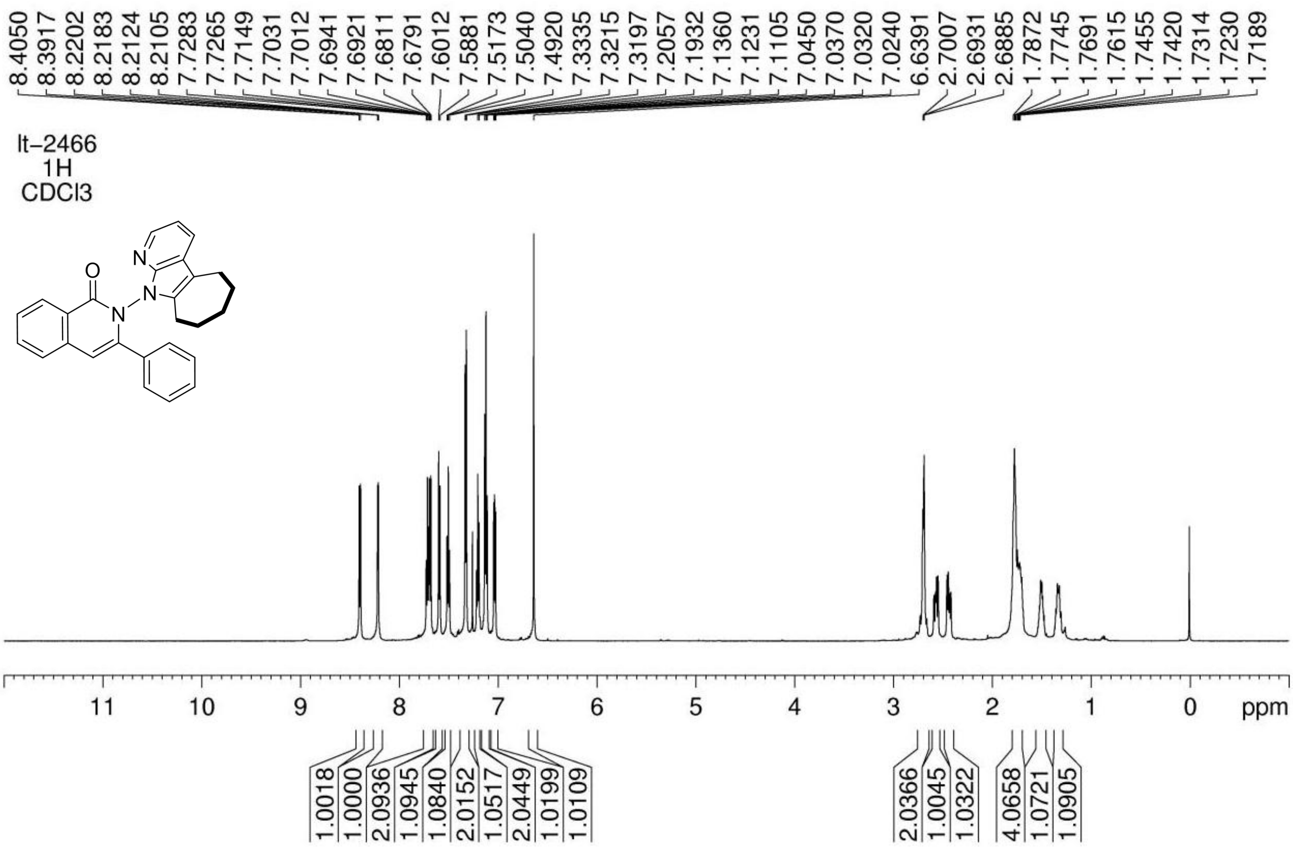

8.4050
8.3917
8.2202
8.2183
8.2124
8.2105
7.7283
7.7265
7.7149
7.7031
7.7012
7.6941
7.6921
7.6811
7.6791
7.6012
7.5881
7.5173
7.5040
7.4920
7.3335
7.3215
7.3197
7.2057
7.1932
7.1360
7.1231
7.1105
7.0450
7.0370
7.0320
7.0240
6.6391
2.7007
2.6931
2.6885
1.7872
1.7745
1.7691
1.7615
1.7455
1.7420
1.7314
1.7230
1.7189
lt–2466
1H
CDCl3
O
N
N
N
11
10
9
8
7
6
5
4
3
2
1
0
ppm
1.0018
1.0000
2.0936
1.0945
1.0840
2.0152
1.0517
2.0449
1.0199
1.0109
2.0366
1.0045
1.0322
4.0658
1.0721
1.0905

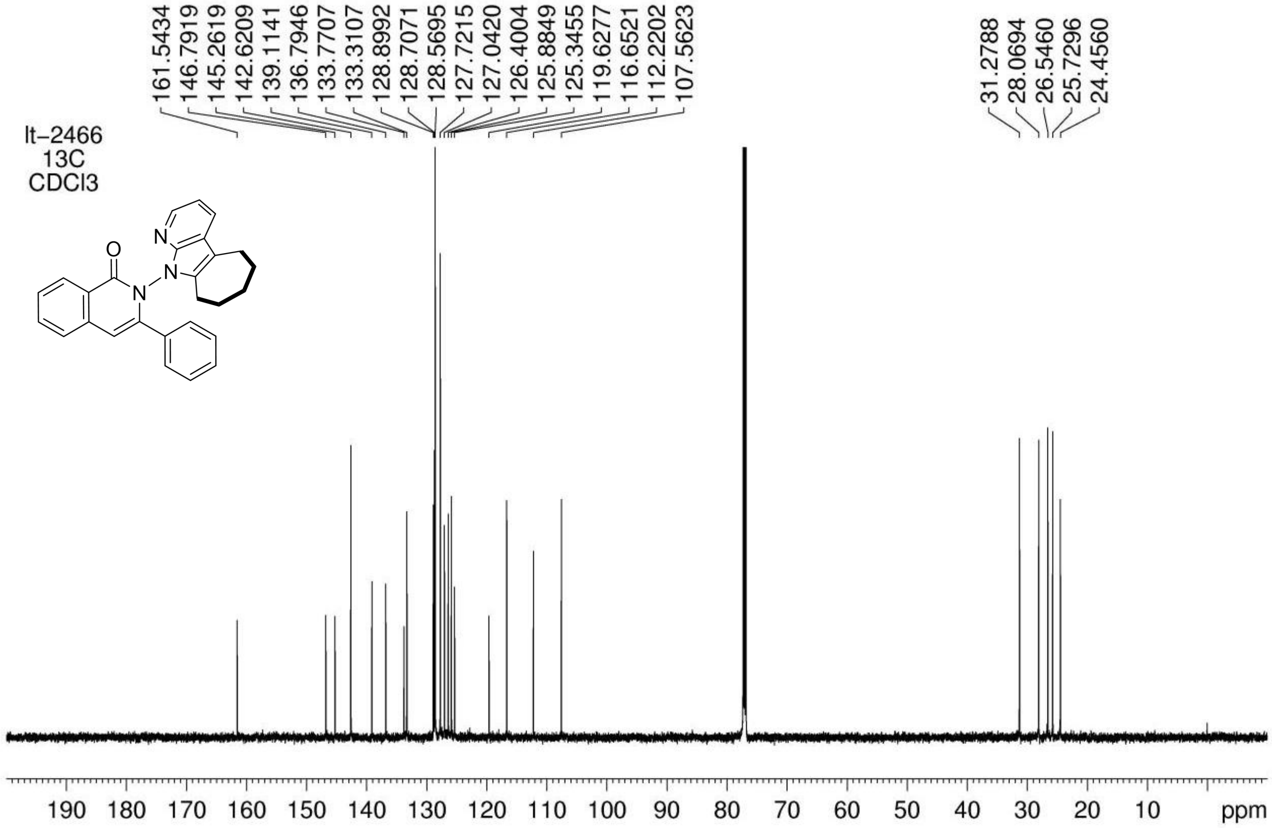

161.5434
146.7919
145.2619
142.6209
139.1141
136.7946
133.7707
133.3107
128.8992
128.7071
128.5695
127.7215
127.0420
126.4004
125.8849
125.3455
119.6277
116.6521
112.2202
107.5623
31.2788
28.0694
26.5460
25.7296
24.4560
lt–2466
13C
CDCl3
O
N
N
N
190
180
170
160
150
140
130
120
110
100
90
80
70
60
50
40
30
20
10
ppm

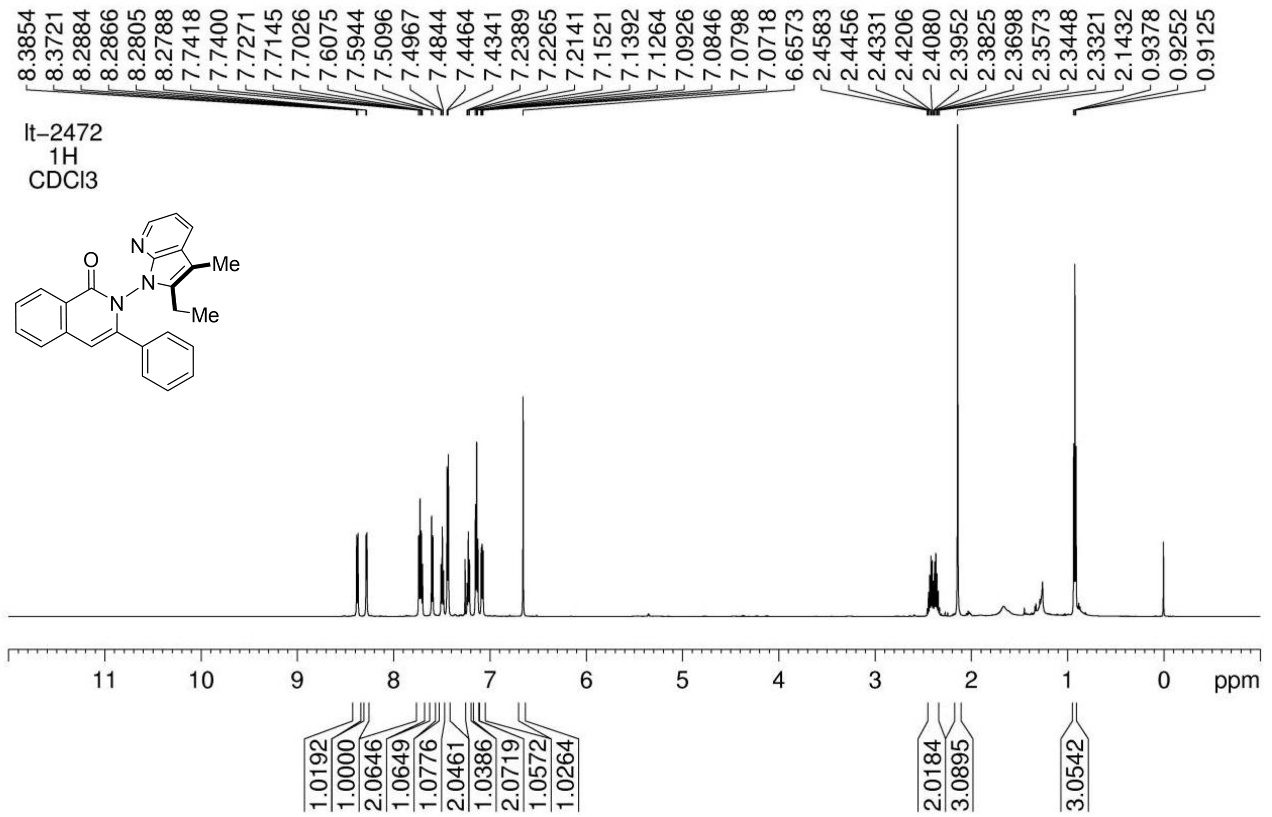
8.3854
8.3721
8.2884
8.2866
8.2805
8.2788
7.7418
7.7400
7.7271
7.7145
7.7026
7.6075
7.5944
7.5096
7.4967
7.4844
7.4464
7.4341
7.2389
7.2265
7.2141
7.1521
7.1392
7.1264
7.0926
7.0846
7.0798
7.0718
6.6573
2.4583
2.4456
2.4331
2.4206
2.4080
2.3952
2.3825
2.3698
2.3573
2.3448
2.3321
2.1432
0.9378
0.9252
0.9125
lt-2472
1H
CDCl3
O
N
N
N
Me
Me
11
10
9
8
7
6
5
4
3
2
1
0
ppm
1.0192
1.0000
2.0646
1.0649
1.0776
2.0461
1.0386
2.0719
1.0572
1.0264
2.0184
3.0895
3.0542

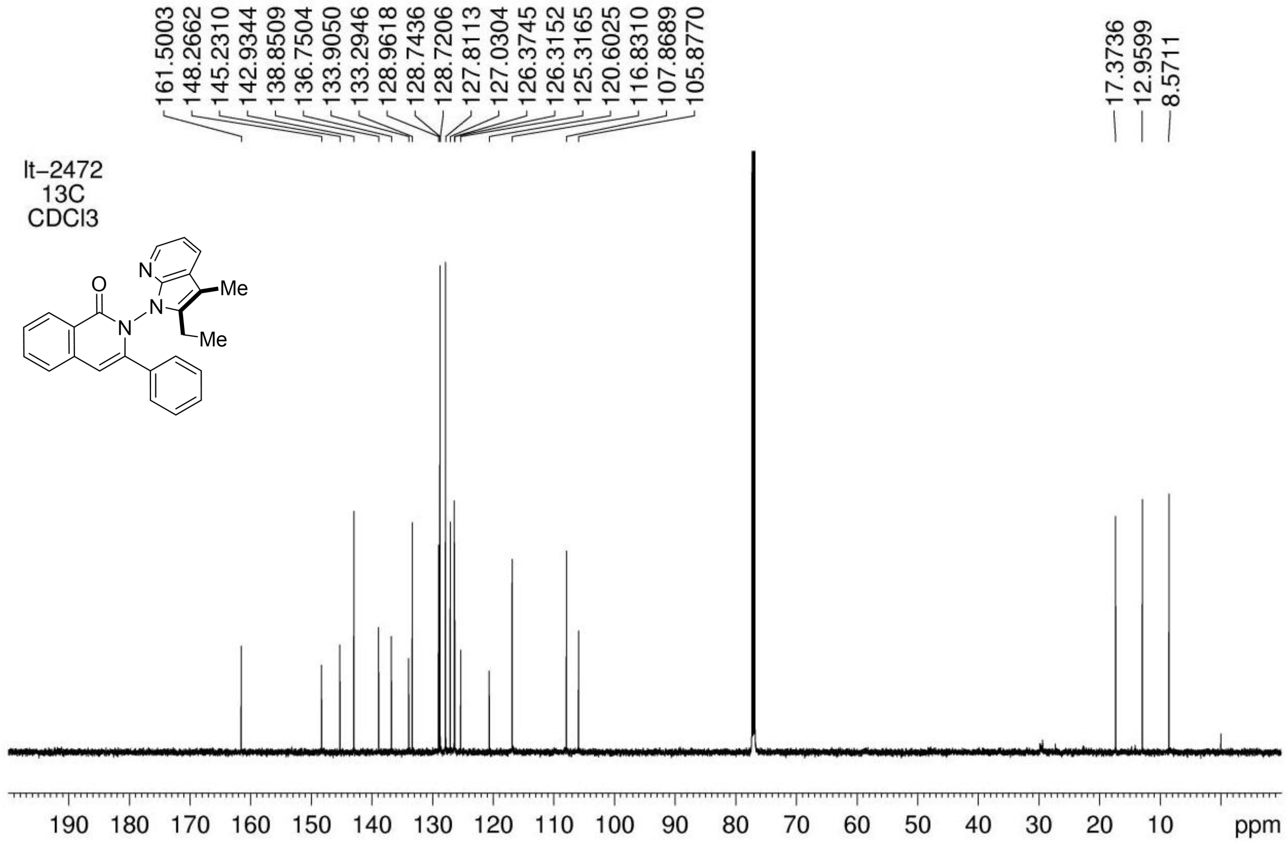
161.5003
148.2662
145.2310
142.9344
138.8509
136.7504
133.9050
133.2946
128.9618
128.7436
128.7206
127.8113
127.0304
126.3745
126.3152
125.3165
120.6025
116.8310
107.8689
105.8770
17.3736
12.9599
8.5711
lt-2472
13C
CDCl3
O
N
N
N
Me
Me
190
180
170
160
150
140
130
120
110
100
90
80
70
60
50
40
30
20
10
ppm

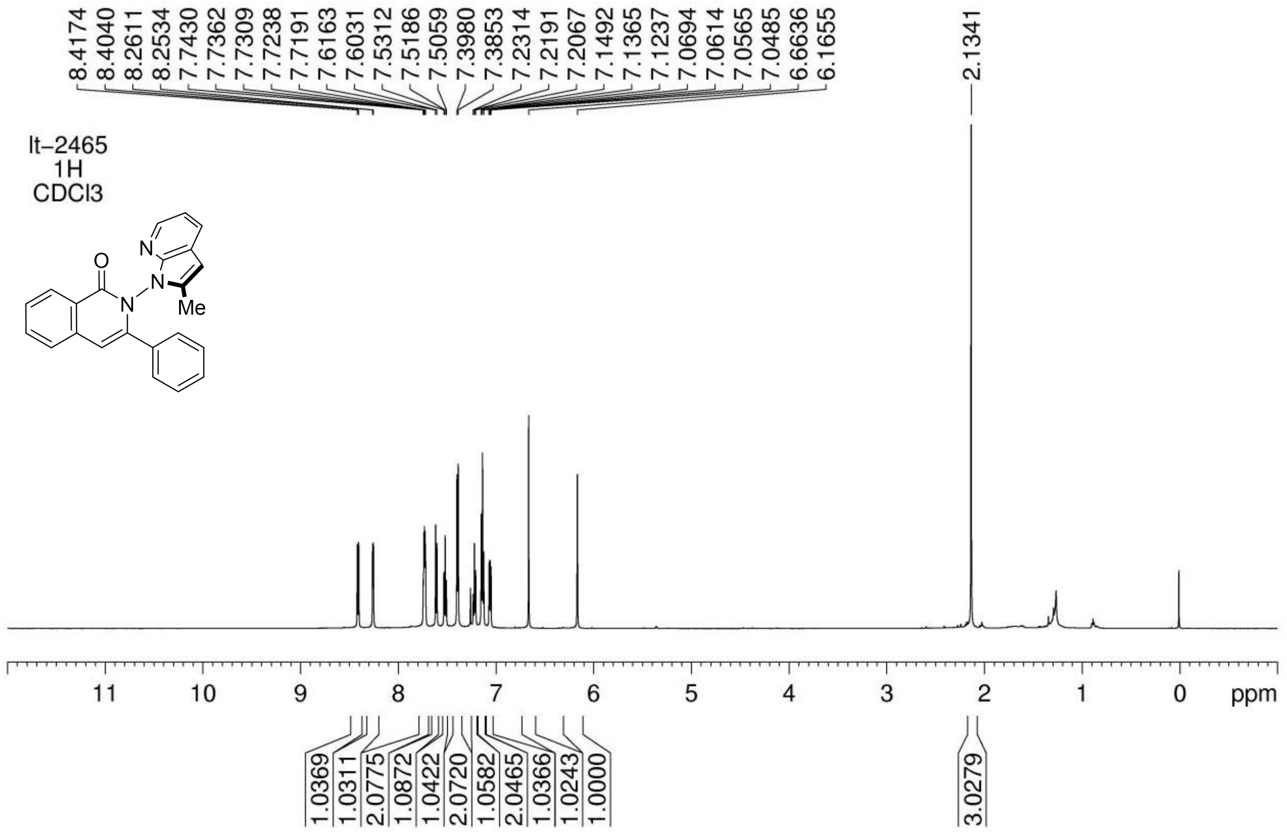

8.4174
8.4040
8.2611
8.2534
7.7430
7.7362
7.7309
7.7238
7.7191
7.6163
7.6031
7.5312
7.5186
7.5059
7.3980
7.3853
7.2314
7.2191
7.2067
7.1492
7.1365
7.1237
7.0694
7.0614
7.0565
7.0485
6.6636
6.1655
2.1341
lt–2465
1H
CDCl3
O
N
N
N
Me
11 10 9 8 7 6 5 4 3 2 1 0 ppm
1.0369
1.0311
2.0775
1.0872
1.0422
2.0720
1.0582
2.0465
1.0366
1.0243
1.0000
3.0279

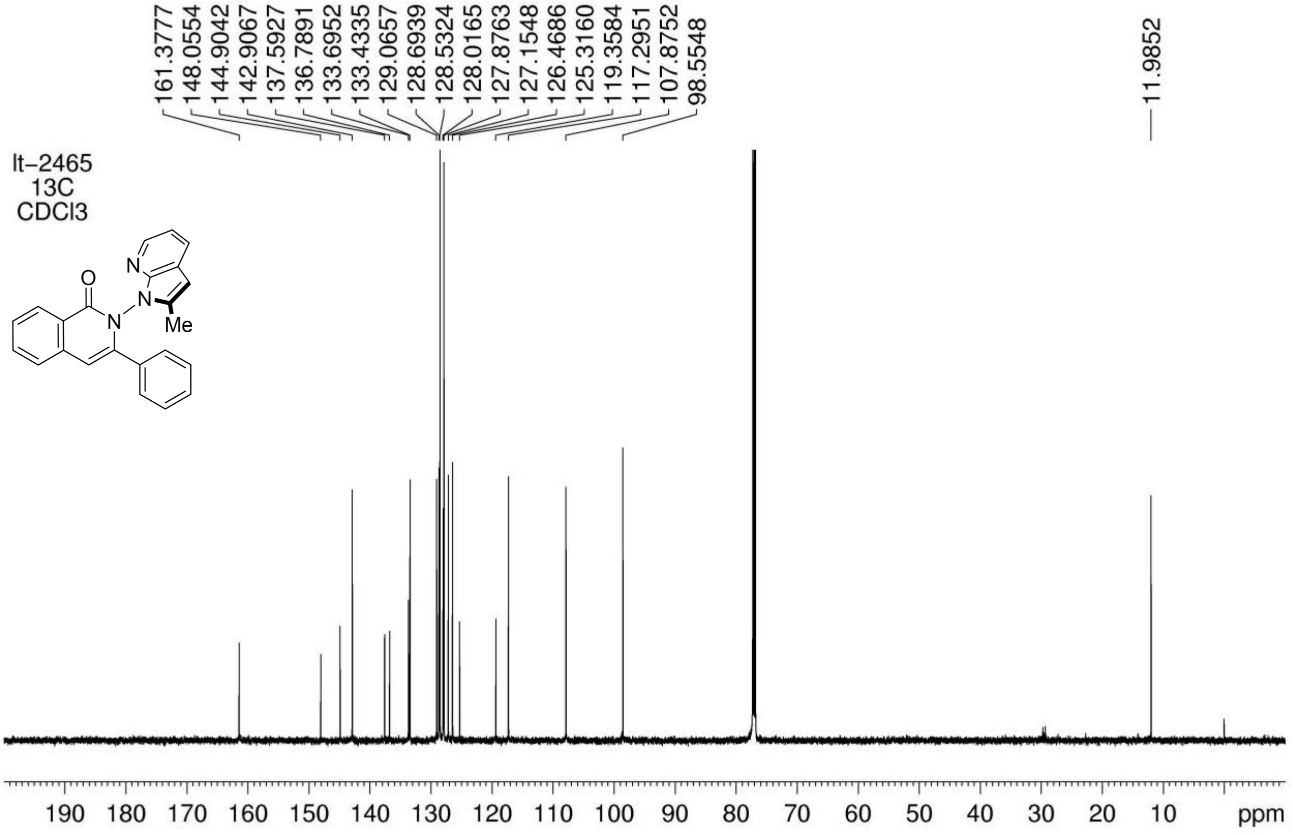

161.3777
148.0554
144.9042
142.9067
137.5927
136.7891
133.6952
133.4335
129.0657
128.6939
128.5324
128.0165
127.8763
127.1548
126.4686
125.3160
119.3584
117.2951
107.8752
98.5548
11.9852
lt–2465
13C
CDCl3
O
N
N
N
Me
190 180 170 160 150 140 130 120 110 100 90 80 70 60 50 40 30 20 10 ppm

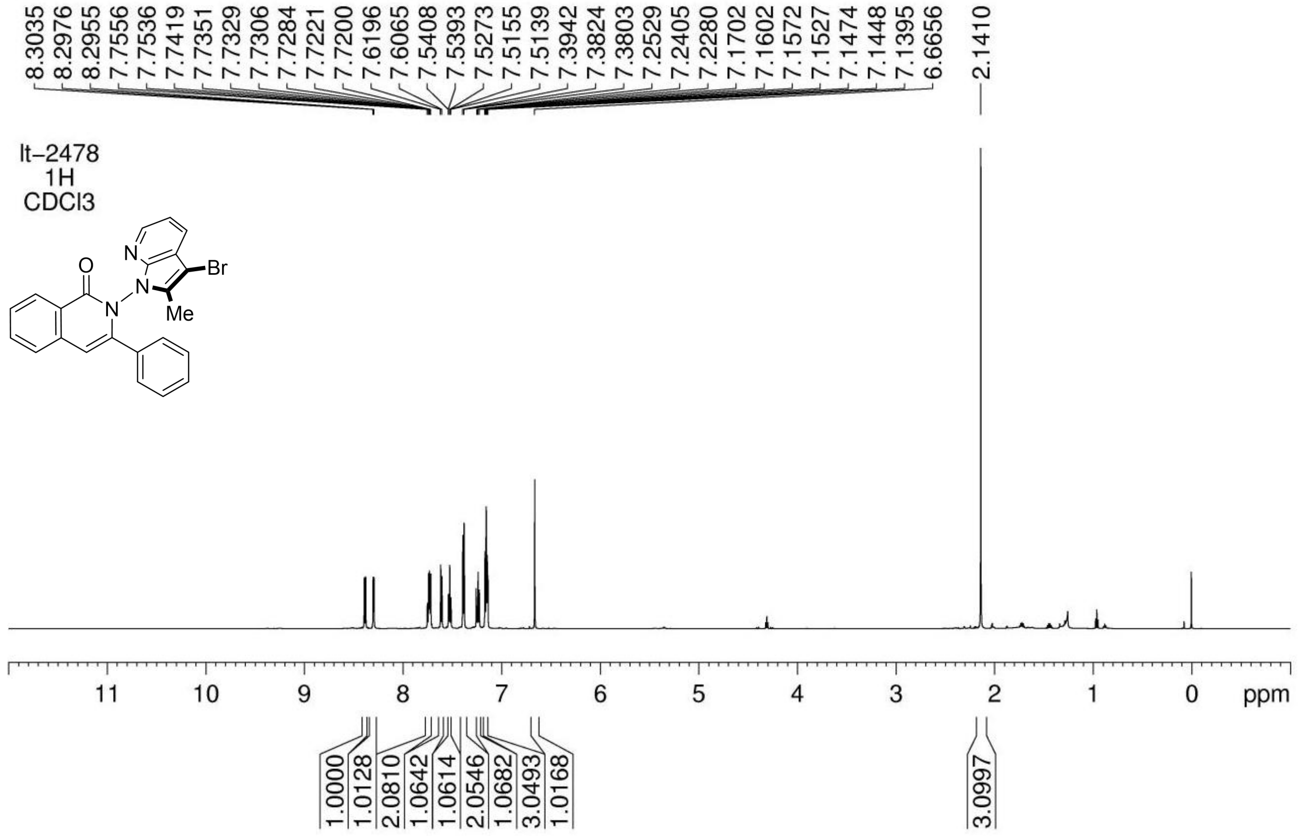

8.3035
8.2976
8.2955
7.7556
7.7536
7.7419
7.7351
7.7329
7.7306
7.7284
7.7221
7.7200
7.6196
7.6065
7.5408
7.5393
7.5273
7.5155
7.5139
7.3942
7.3824
7.3803
7.2529
7.2405
7.2280
7.1702
7.1602
7.1572
7.1527
7.1474
7.1448
7.1395
6.6656
2.1410
lt–2478
1H
CDCl3
O
N
N
N
Br
Me
11
10
9
8
7
6
5
4
3
2
1
0
ppm
1.0000
1.0128
2.0810
1.0642
1.0614
2.0546
1.0682
3.0493
1.0168
3.0997

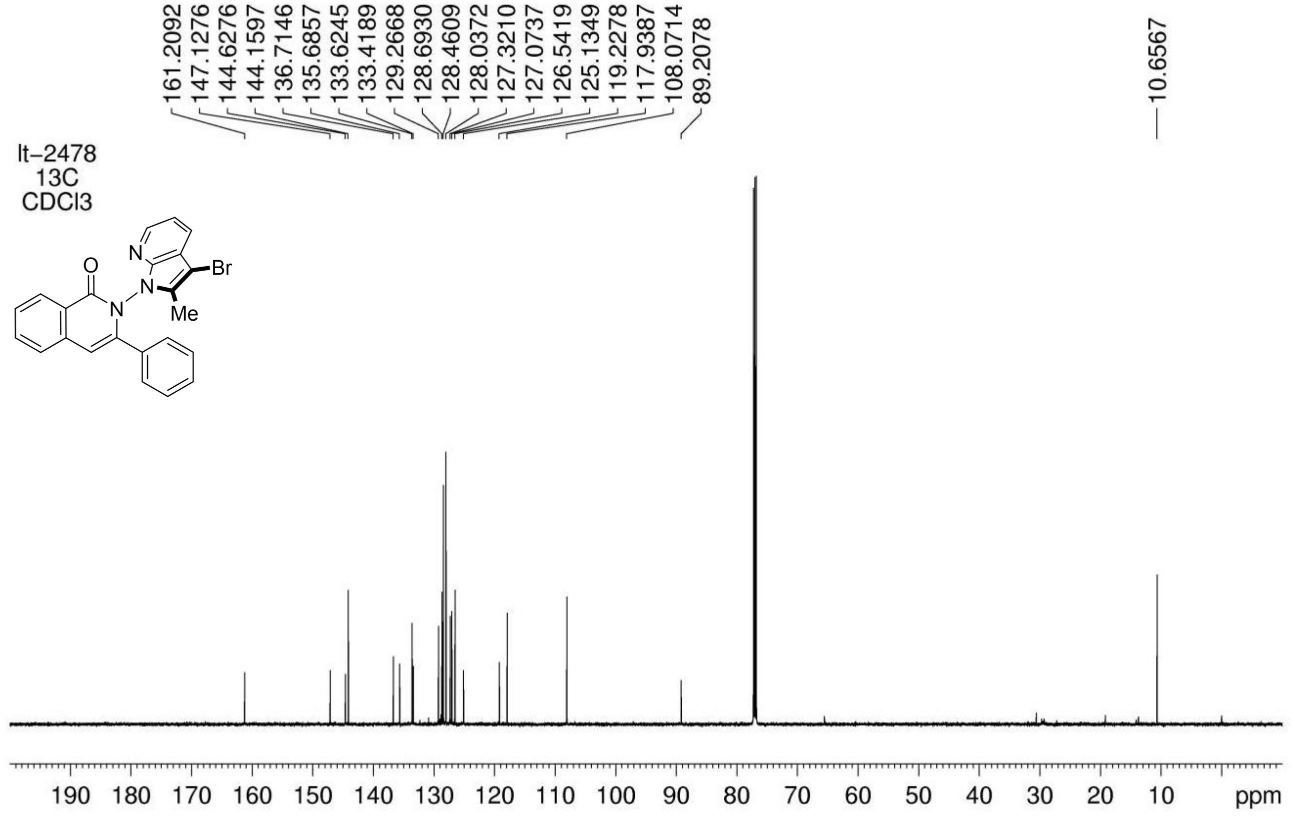

161.2092
147.1276
144.6276
144.1597
136.7146
135.6857
133.6245
133.4189
129.2668
128.6930
128.4609
128.0372
127.3210
127.0737
126.5419
125.1349
119.2278
117.9387
108.0714
89.2078
10.6567
lt–2478
13C
CDCl3
O
N
N
N
Br
Me
190
180
170
160
150
140
130
120
110
100
90
80
70
60
50
40
30
20
10
ppm

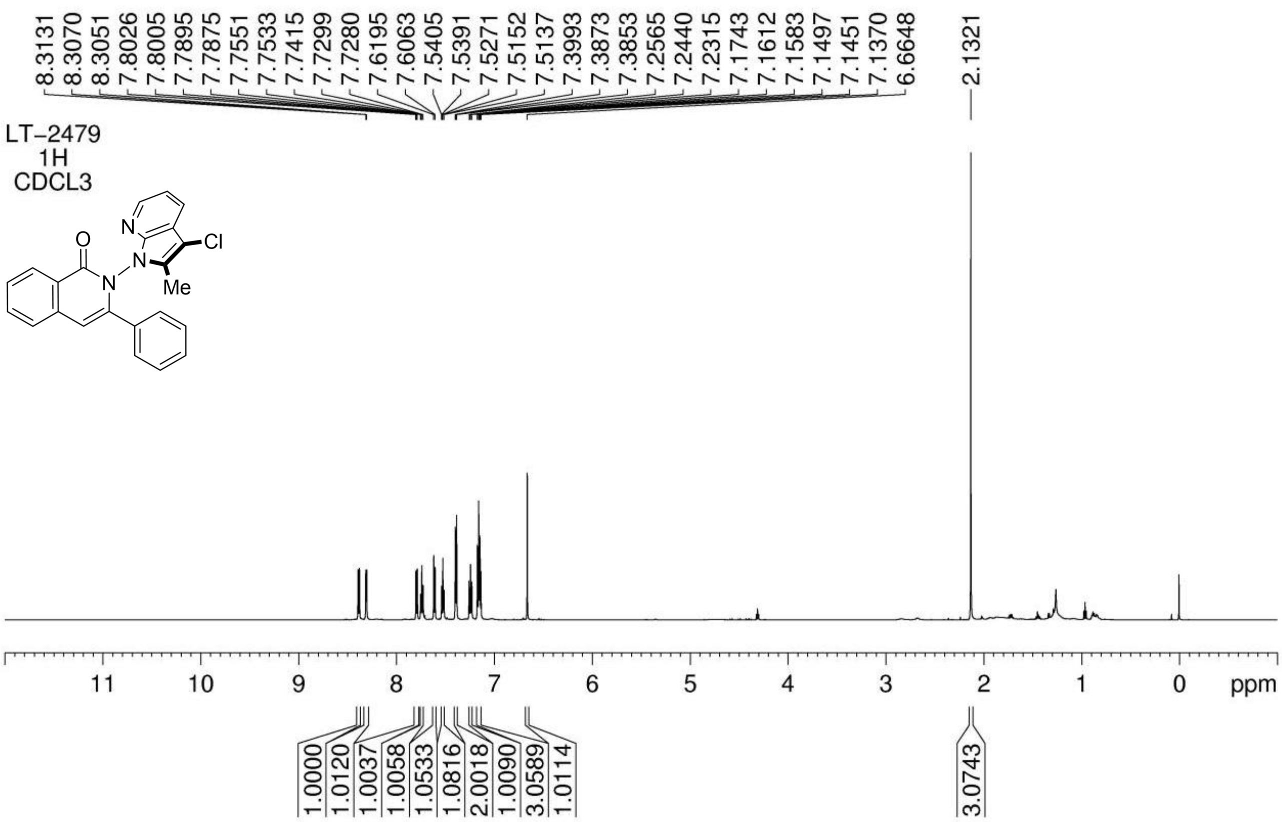
8.3131
8.3070
8.3051
7.8026
7.8005
7.7895
7.7875
7.7551
7.7533
7.7415
7.7299
7.7280
7.6195
7.6063
7.5405
7.5391
7.5271
7.5152
7.5137
7.3993
7.3873
7.3853
7.2565
7.2440
7.2315
7.1743
7.1612
7.1583
7.1497
7.1451
7.1370
6.6648
2.1321
LT–2479
1H
CDCL3
N
O
N
N
Cl
Me
11 10 9 8 7 6 5 4 3 2 1 0 ppm
1.0000
1.0120
1.0037
1.0058
1.0533
1.0816
2.0018
1.0090
3.0589
1.0114
3.0743

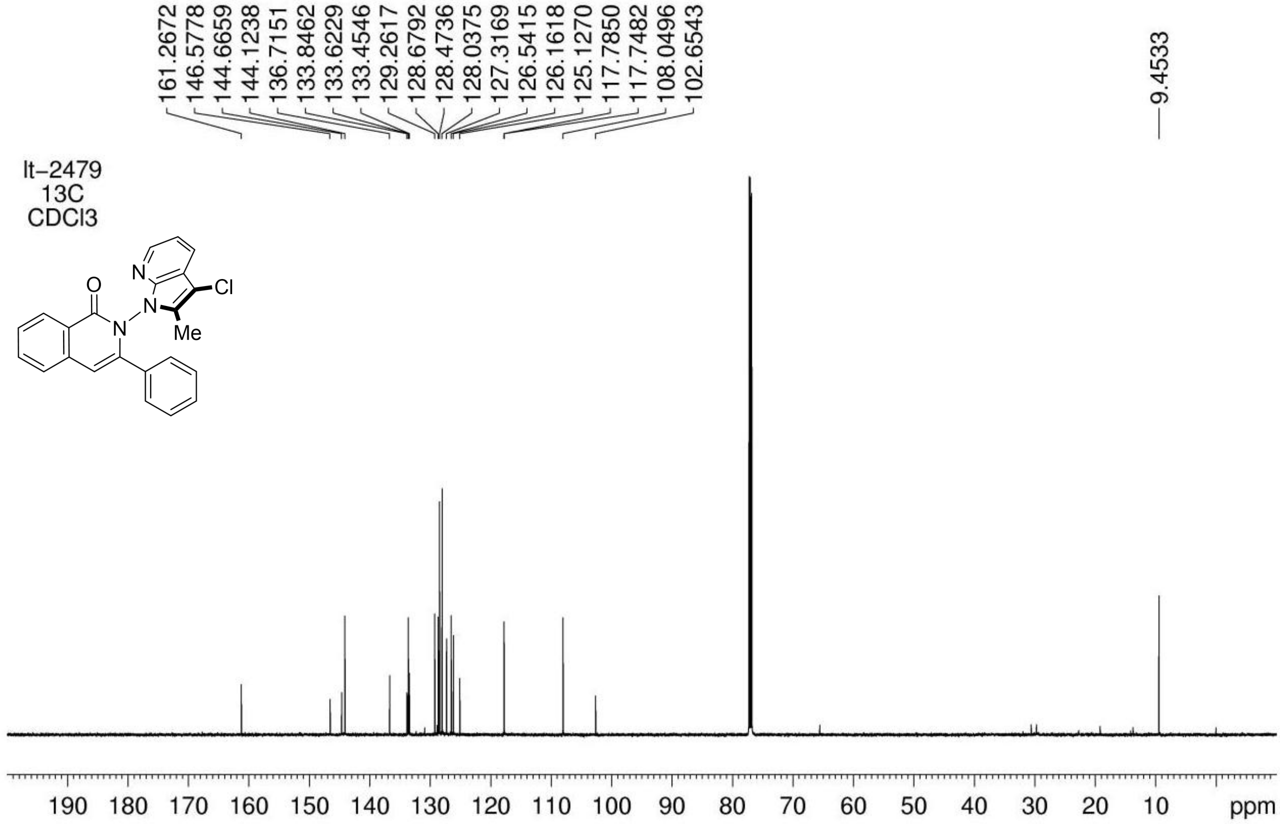
161.2672
146.5778
144.6659
144.1238
136.7151
133.8462
133.6229
133.4546
129.2617
128.6792
128.4736
128.0375
127.3169
126.5415
126.1618
125.1270
117.7850
117.7482
108.0496
102.6543
9.4533
lt–2479
13C
CDCl3
N
O
N
N
Cl
Me
190 180 170 160 150 140 130 120 110 100 90 80 70 60 50 40 30 20 10 ppm

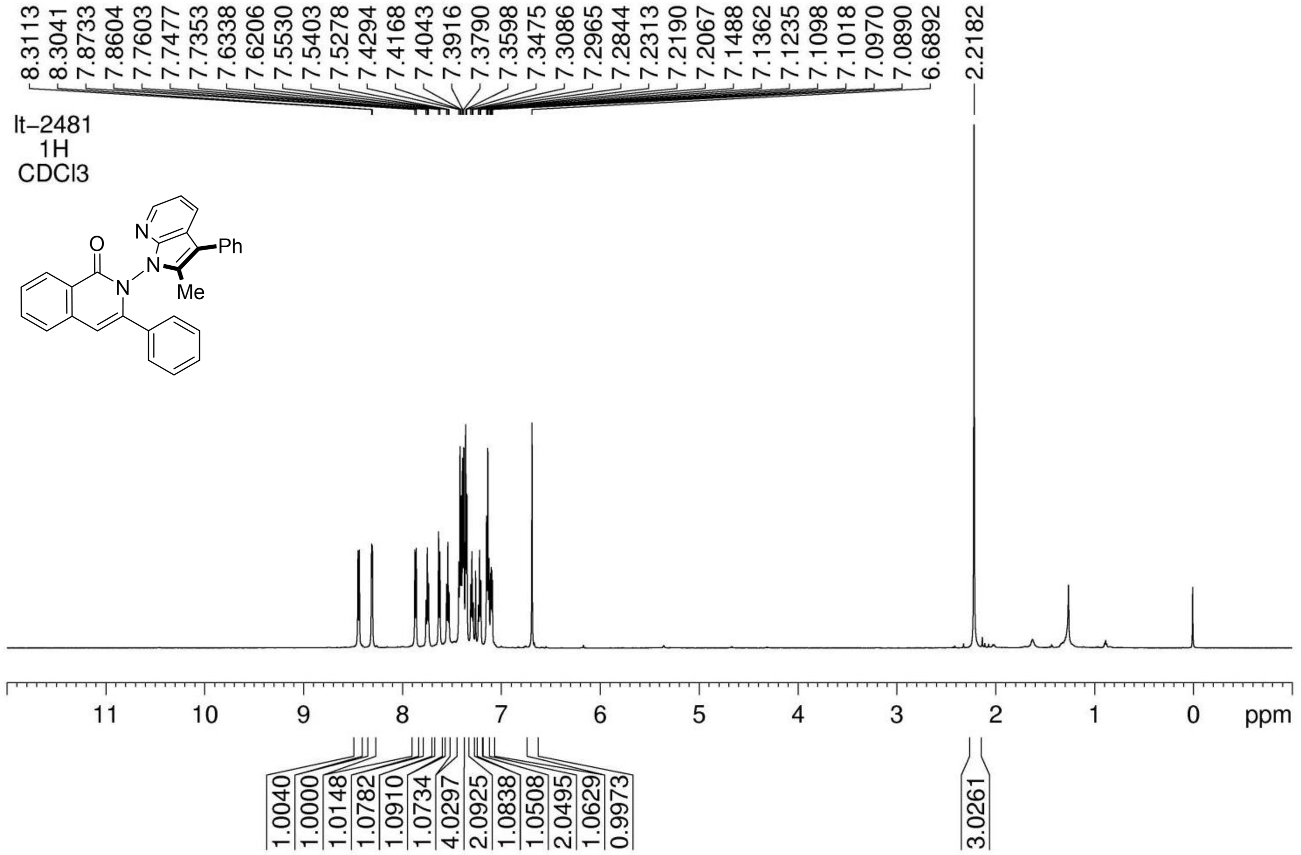
lt–2481
1H
CDCl3
Ph
Me
ppm

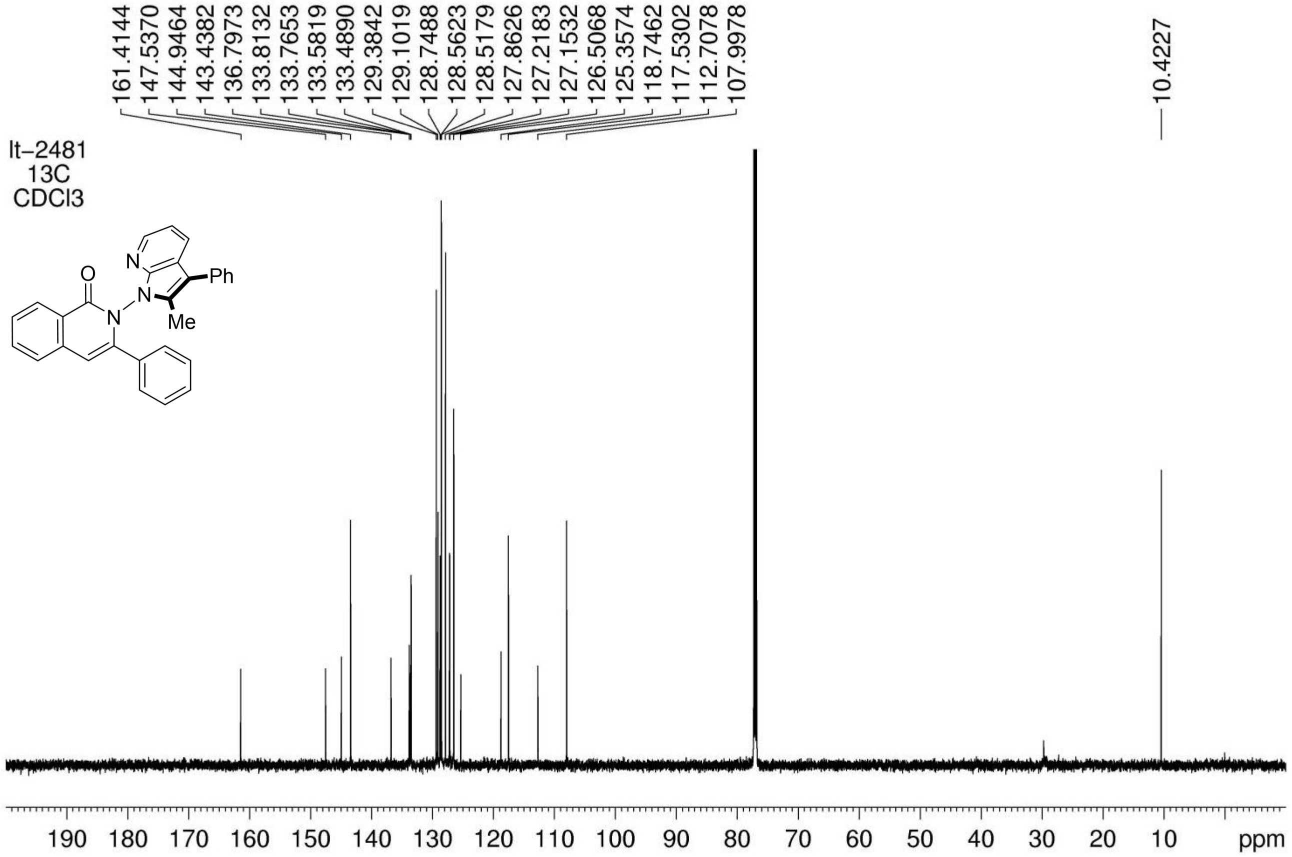
lt–2481
13C
CDCl3
Ph
Me
ppm

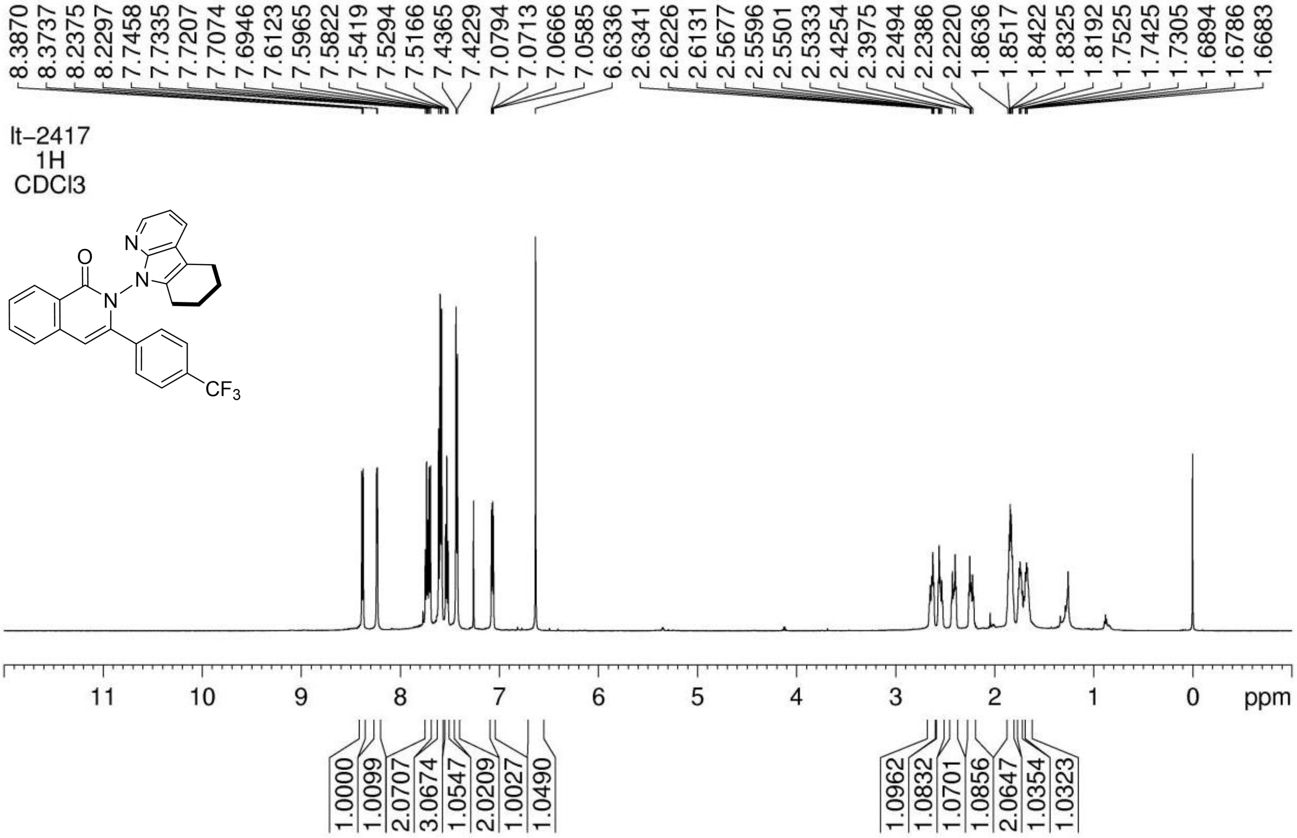
8.3870
8.3737
8.2375
8.2297
7.7458
7.7335
7.7207
7.7074
7.6946
7.6123
7.5965
7.5822
7.5419
7.5294
7.5166
7.4365
7.4229
7.0794
7.0713
7.0666
7.0585
6.6336
2.6341
2.6226
2.6131
2.5677
2.5596
2.5501
2.5333
2.4254
2.3975
2.2494
2.2386
2.2220
1.8636
1.8517
1.8422
1.8325
1.8192
1.7525
1.7425
1.7305
1.6894
1.6786
1.6683
lt–2417
1H
CDCl3
O
N
N
N
CF_3
11
10
9
8
7
6
5
4
3
2
1
0
ppm
1.0000
1.0099
2.0707
3.0674
1.0547
2.0209
1.0027
1.0490
1.0962
1.0832
1.0701
1.0856
2.0647
1.0354
1.0323

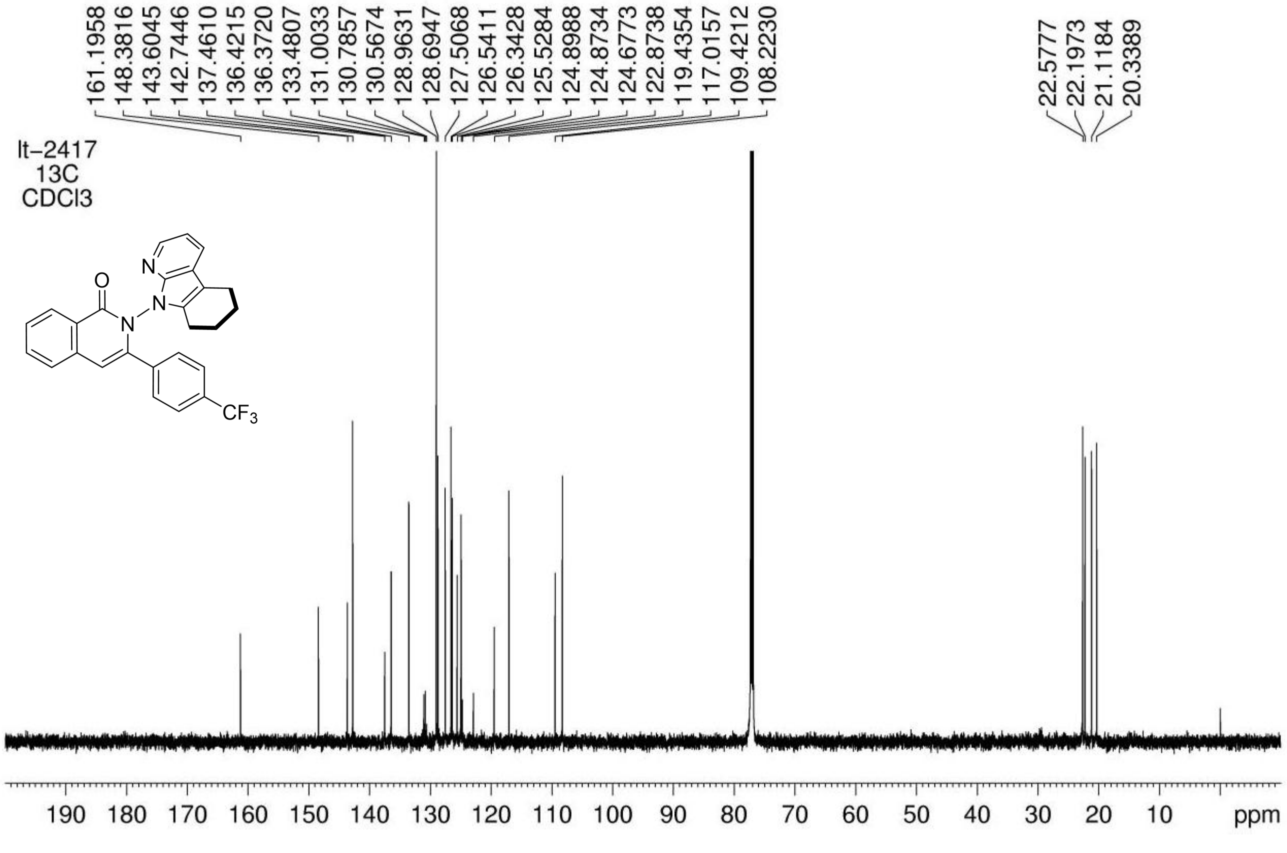
161.1958
148.3816
143.6045
142.7446
137.4610
136.4215
136.3720
133.4807
131.0033
130.7857
130.5674
128.9631
128.6947
127.5068
126.5411
126.3428
125.5284
124.8988
124.8734
124.6773
122.8738
119.4354
117.0157
109.4212
108.2230
22.5777
22.1973
21.1184
20.3389
lt–2417
13C
CDCl3
O
N
N
N
CF_3
190
180
170
160
150
140
130
120
110
100
90
80
70
60
50
40
30
20
10
ppm

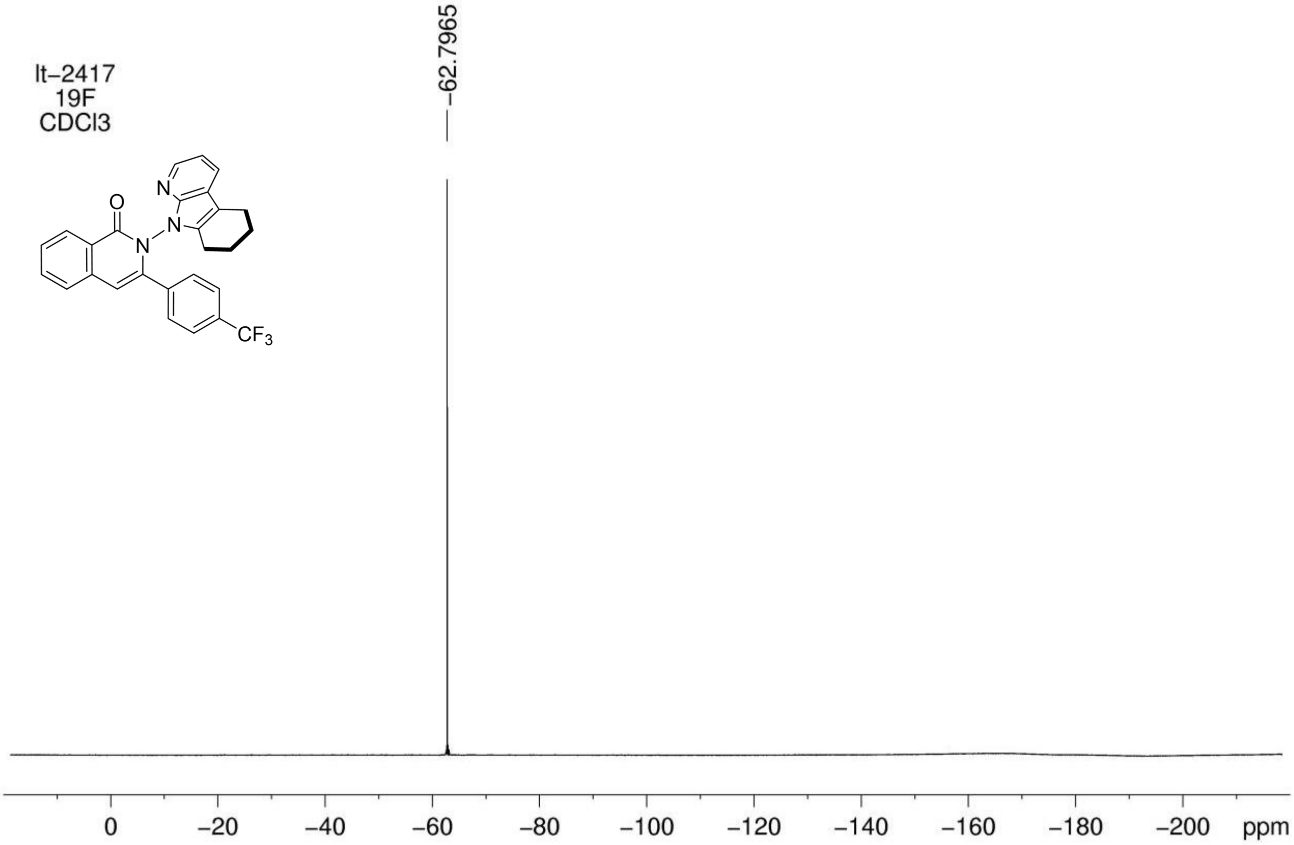
lt–2417
19F
CDCl3
CF3
–62.7965
0
–20
–40
–60
–80
–100
–120
–140
–160
–180
–200
ppm

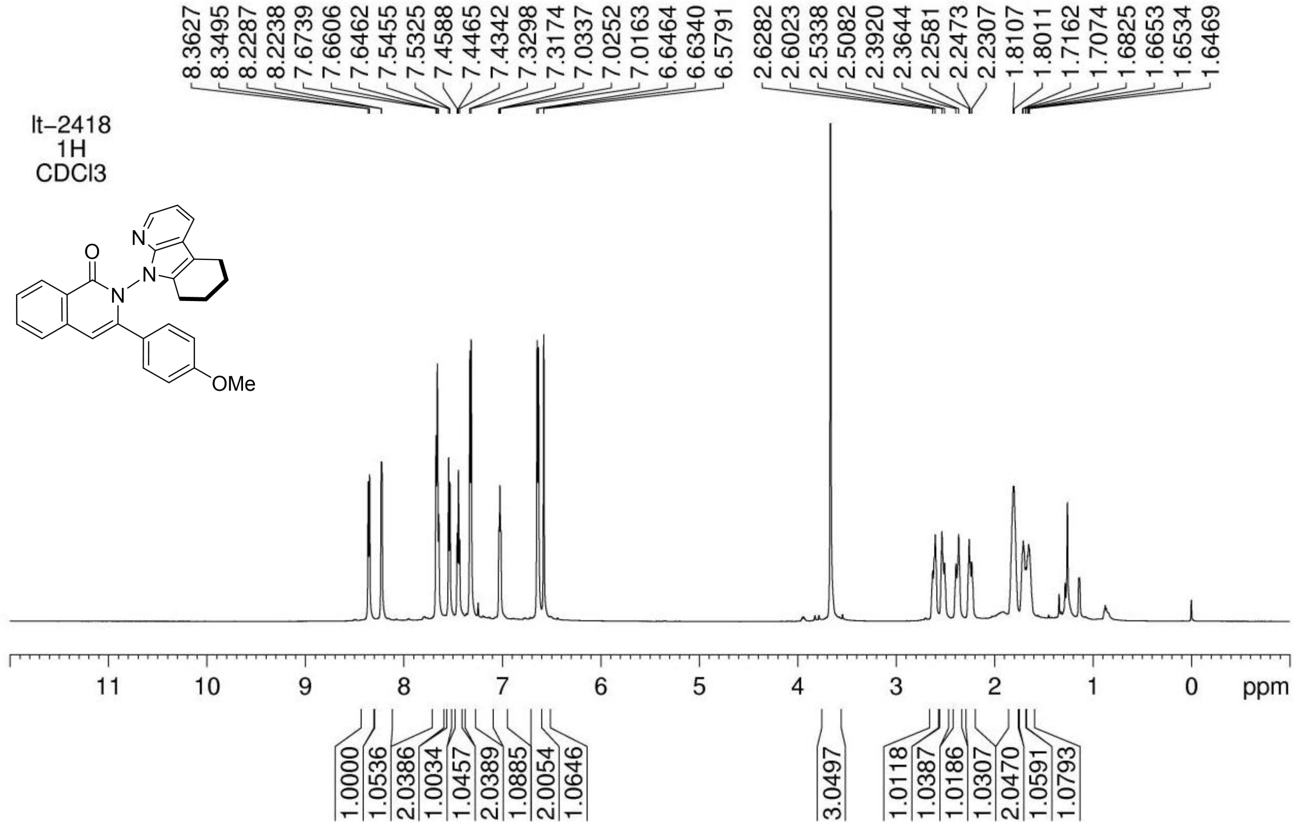
lt–2418
1H
CDCl3
O
N
N
N
OMe
8.3627
8.3495
8.2287
8.2238
7.6739
7.6606
7.6462
7.5455
7.5325
7.4588
7.4465
7.4342
7.3298
7.3174
7.0337
7.0252
7.0163
6.6464
6.6340
6.5791
2.6282
2.6023
2.5338
2.5082
2.3920
2.3644
2.2581
2.2473
2.2307
1.8107
1.8011
1.7162
1.7074
1.6825
1.6653
1.6534
1.6469
1.0000
1.0536
2.0386
1.0034
1.0457
2.0389
1.0885
2.0054
1.0646
3.0497
1.0118
1.0387
1.0186
1.0307
2.0470
1.0591
1.0793
ppm

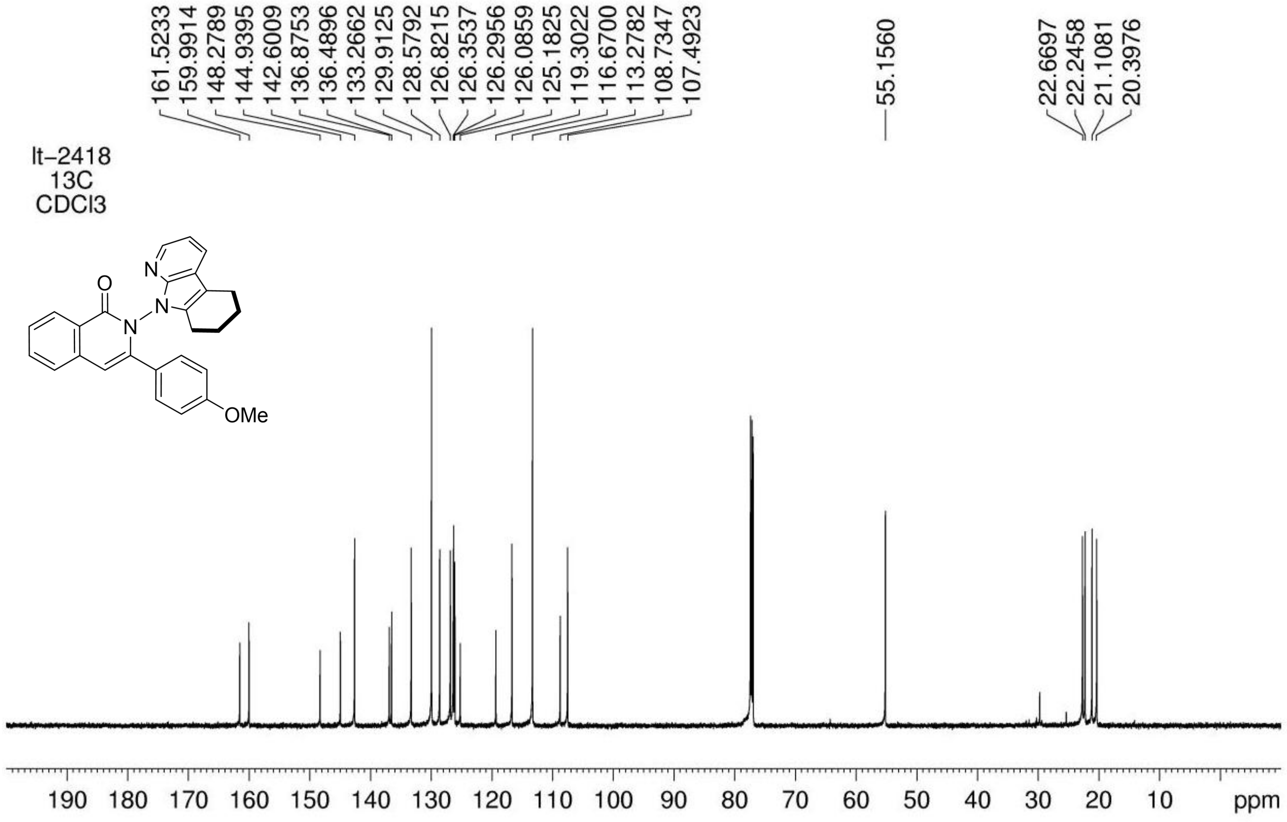
lt–2418
13C
CDCl3
O
N
N
N
OMe
161.5233
159.9914
148.2789
144.9395
142.6009
136.8753
136.4896
133.2662
129.9125
128.5792
126.8215
126.3537
126.2956
126.0859
125.1825
119.3022
116.6700
113.2782
108.7347
107.4923
55.1560
22.6697
22.2458
21.1081
20.3976
ppm

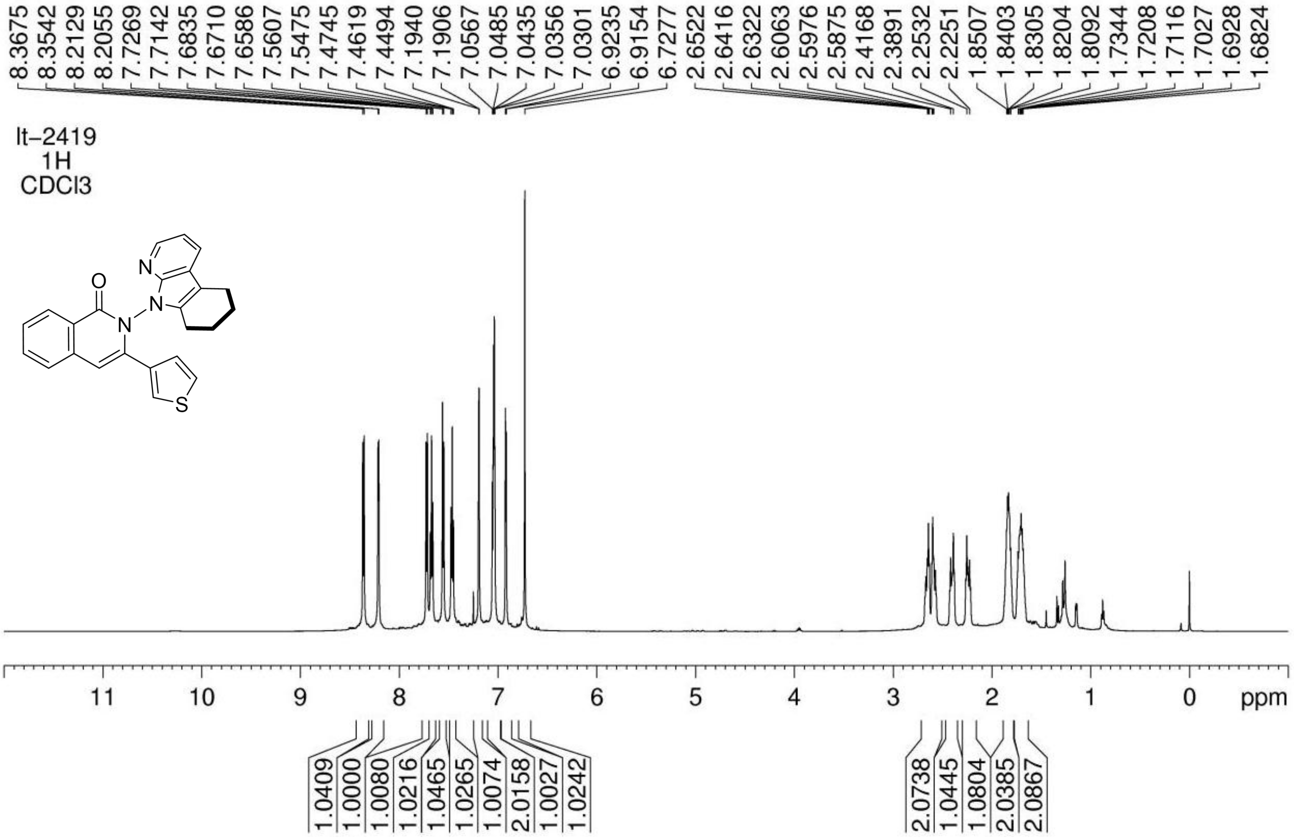
8.3675
8.3542
8.2129
8.2055
7.7269
7.7142
7.6835
7.6710
7.6586
7.5607
7.5475
7.4745
7.4619
7.4494
7.1940
7.1906
7.0567
7.0485
7.0435
7.0356
7.0301
6.9235
6.9154
6.7277
2.6522
2.6416
2.6322
2.6063
2.5976
2.5875
2.4168
2.3891
2.2532
2.2251
1.8507
1.8403
1.8305
1.8204
1.8092
1.7344
1.7208
1.7116
1.7027
1.6928
1.6824
lt–2419
1H
CDCl3
O
N
N
N
S
11
10
9
8
7
6
5
4
3
2
1
0
ppm
1.0409
1.0000
1.0080
1.0216
1.0465
1.0265
1.0074
2.0158
1.0027
1.0242
2.0738
1.0445
1.0804
2.0385
2.0867

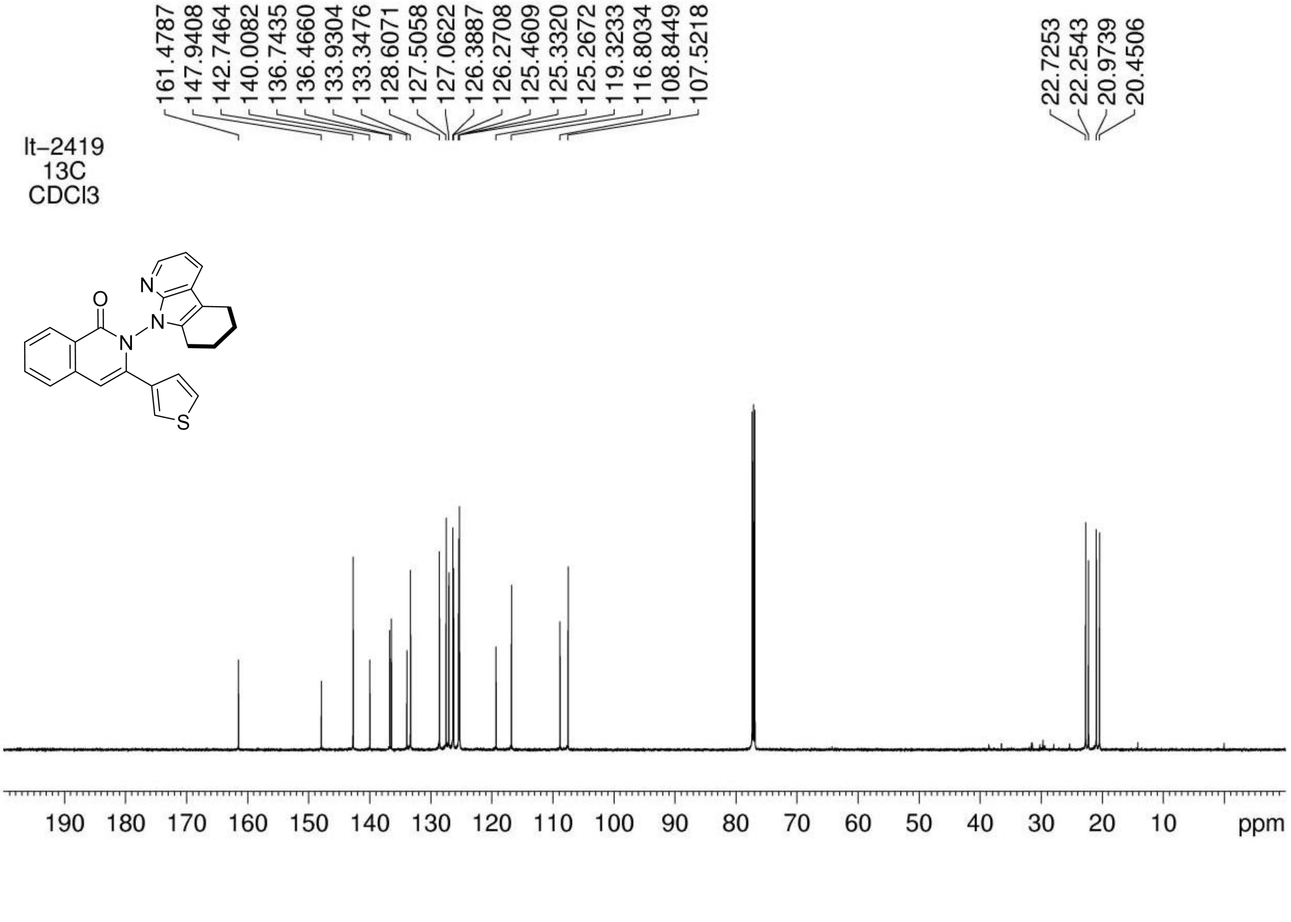
161.4787
147.9408
142.7464
140.0082
136.7435
136.4660
133.9304
133.3476
128.6071
127.5058
127.0622
126.3887
126.2708
125.4609
125.3320
125.2672
119.3233
116.8034
108.8449
107.5218
22.7253
22.2543
20.9739
20.4506
lt–2419
13C
CDCl3
O
N
N
N
S
190
180
170
160
150
140
130
120
110
100
90
80
70
60
50
40
30
20
10
ppm

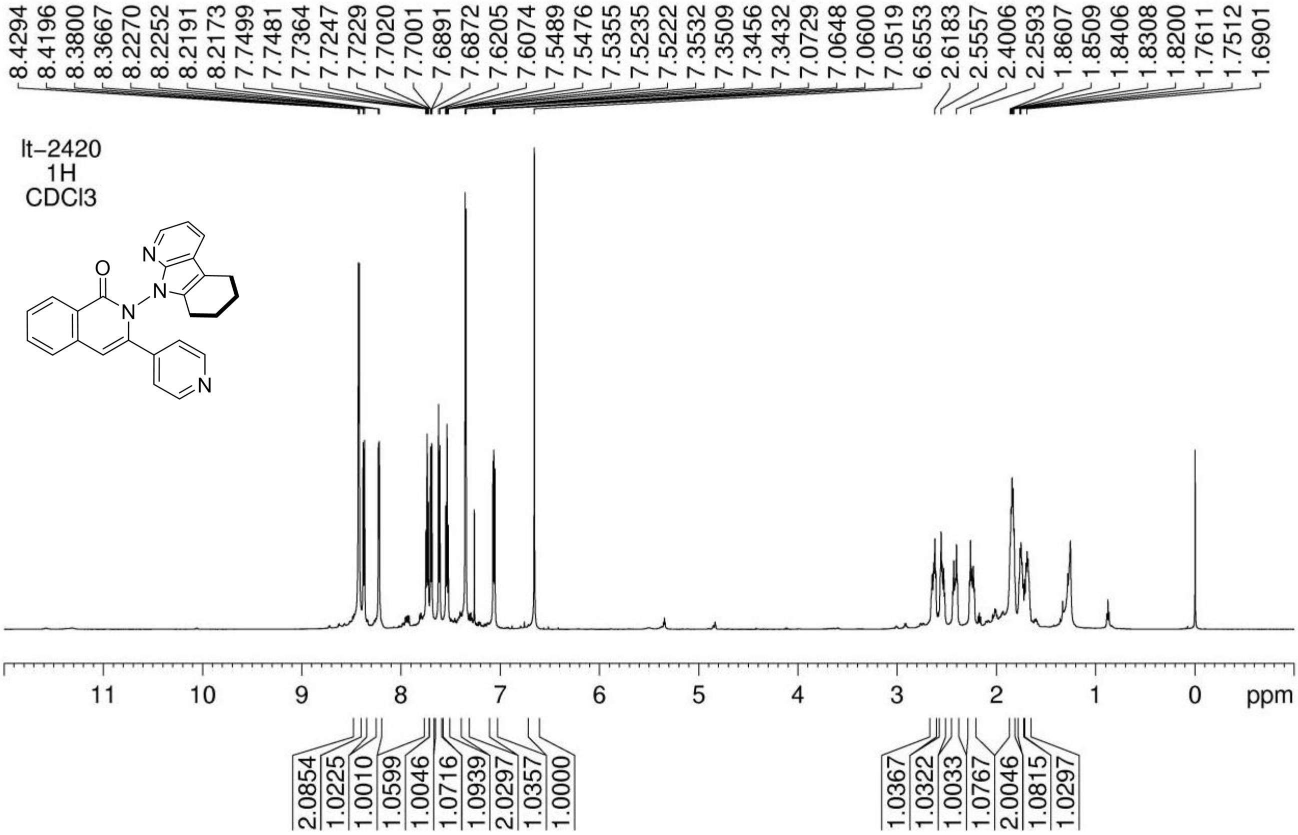
8.4294
8.4196
8.3800
8.3667
8.2270
8.2252
8.2191
8.2173
7.7499
7.7481
7.7364
7.7247
7.7229
7.7020
7.7001
7.6891
7.6872
7.6205
7.6074
7.5489
7.5476
7.5355
7.5235
7.5222
7.3532
7.3509
7.3456
7.3432
7.0729
7.0648
7.0600
7.0519
6.6553
2.6183
2.5557
2.4006
2.2593
1.8607
1.8509
1.8406
1.8308
1.8200
1.7611
1.7512
1.6901
lt–2420
1H
CDCl3
O
N
N
N
N
11
10
9
8
7
6
5
4
3
2
1
0
ppm
2.0854
1.0225
1.0010
1.0599
1.0046
1.0716
1.0939
2.0297
1.0357
1.0000
1.0367
1.0322
1.0033
1.0767
2.0046
1.0815
1.0297

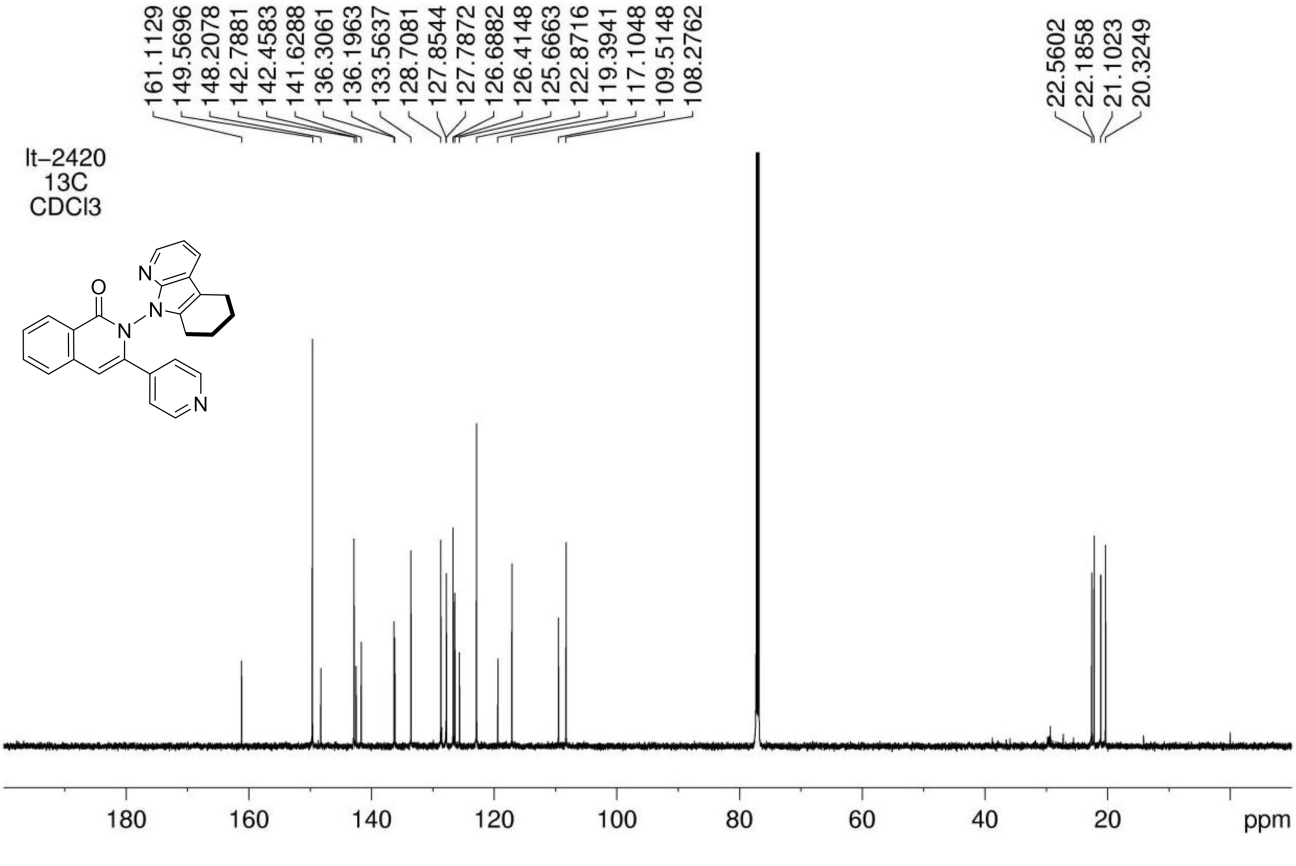
161.1129
149.5696
148.2078
142.7881
142.4583
141.6288
136.3061
136.1963
133.5637
128.7081
127.8544
127.7872
126.6882
126.4148
125.6663
122.8716
119.3941
117.1048
109.5148
108.2762
22.5602
22.1858
21.1023
20.3249
lt–2420
13C
CDCl3
O
N
N
N
N
180
160
140
120
100
80
60
40
20
ppm

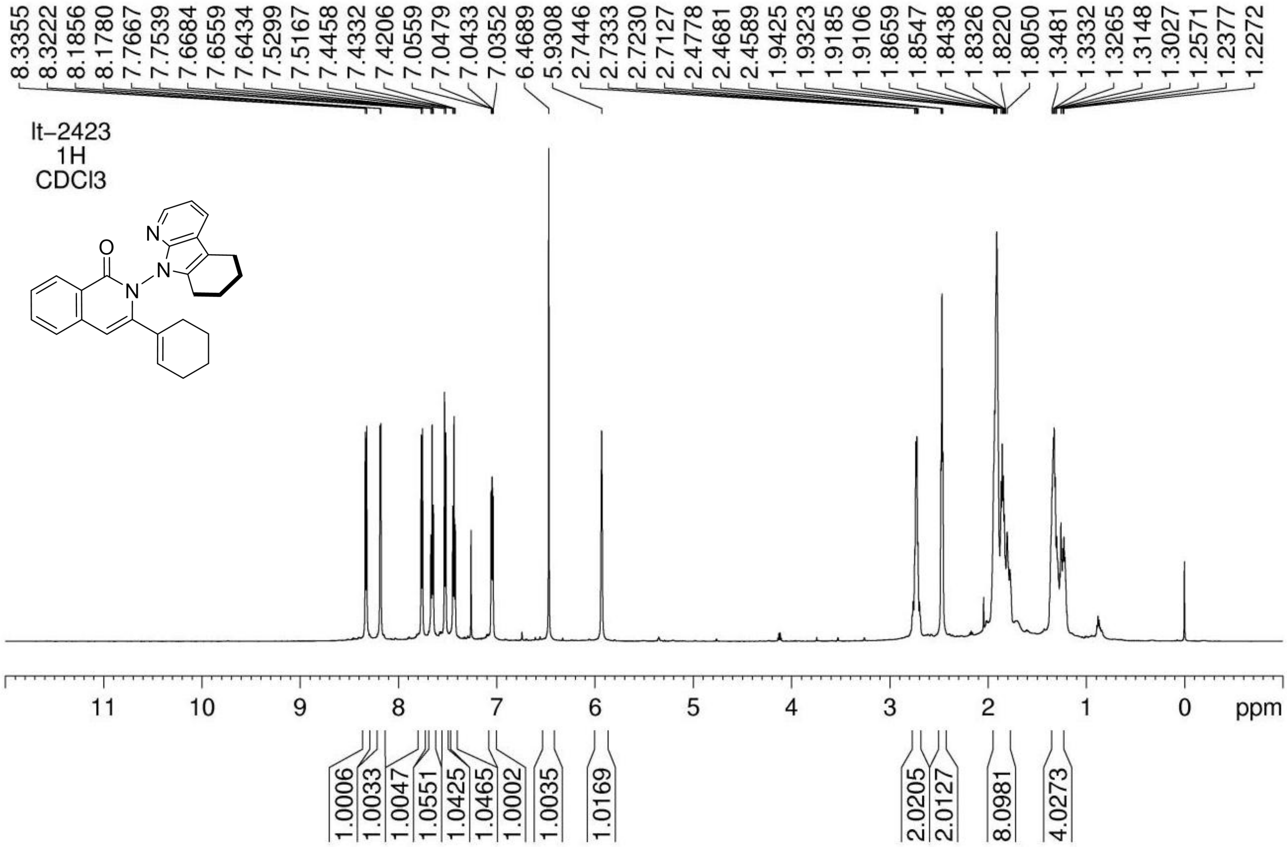
8.3355
8.3222
8.1856
8.1780
7.7667
7.7539
7.6684
7.6559
7.6434
7.5299
7.5167
7.4458
7.4332
7.4206
7.0559
7.0479
7.0433
7.0352
6.4689
5.9308
2.7446
2.7333
2.7230
2.7127
2.4778
2.4681
2.4589
1.9425
1.9323
1.9185
1.9106
1.8659
1.8547
1.8438
1.8326
1.8220
1.8050
1.3481
1.3332
1.3265
1.3148
1.3027
1.2571
1.2377
1.2272
lt–2423
1H
CDCl3
O
N
N
N
11
10
9
8
7
6
5
4
3
2
1
0
ppm
1.0006
1.0033
1.0047
1.0551
1.0425
1.0465
1.0002
1.0035
1.0169
2.0205
2.0127
8.0981
4.0273

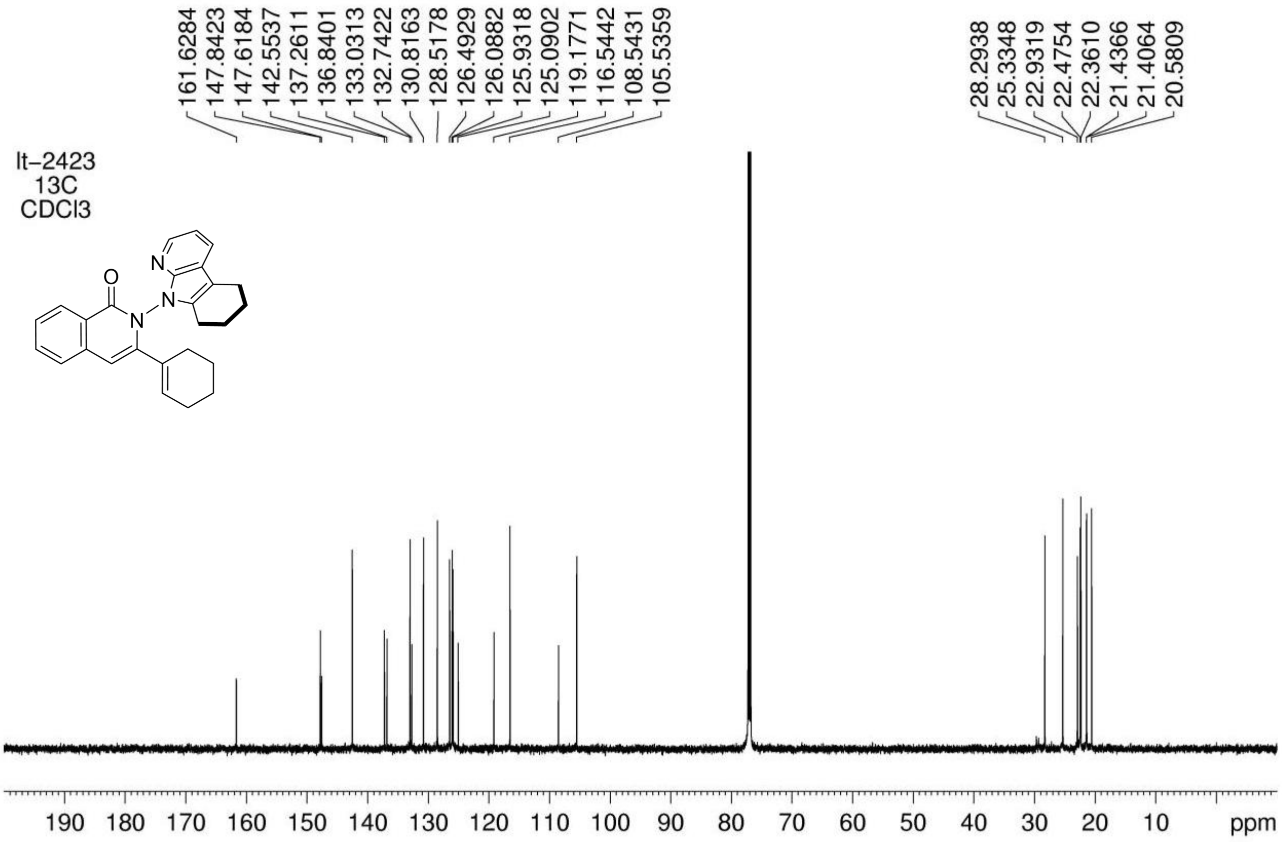
161.6284
147.8423
147.6184
142.5537
137.2611
136.8401
133.0313
132.7422
130.8163
128.5178
126.4929
126.0882
125.9318
125.0902
119.1771
116.5442
108.5431
105.5359
28.2938
25.3348
22.9319
22.4754
22.3610
21.4366
21.4064
20.5809
lt–2423
13C
CDCl3
O
N
N
N
190
180
170
160
150
140
130
120
110
100
90
80
70
60
50
40
30
20
10
ppm

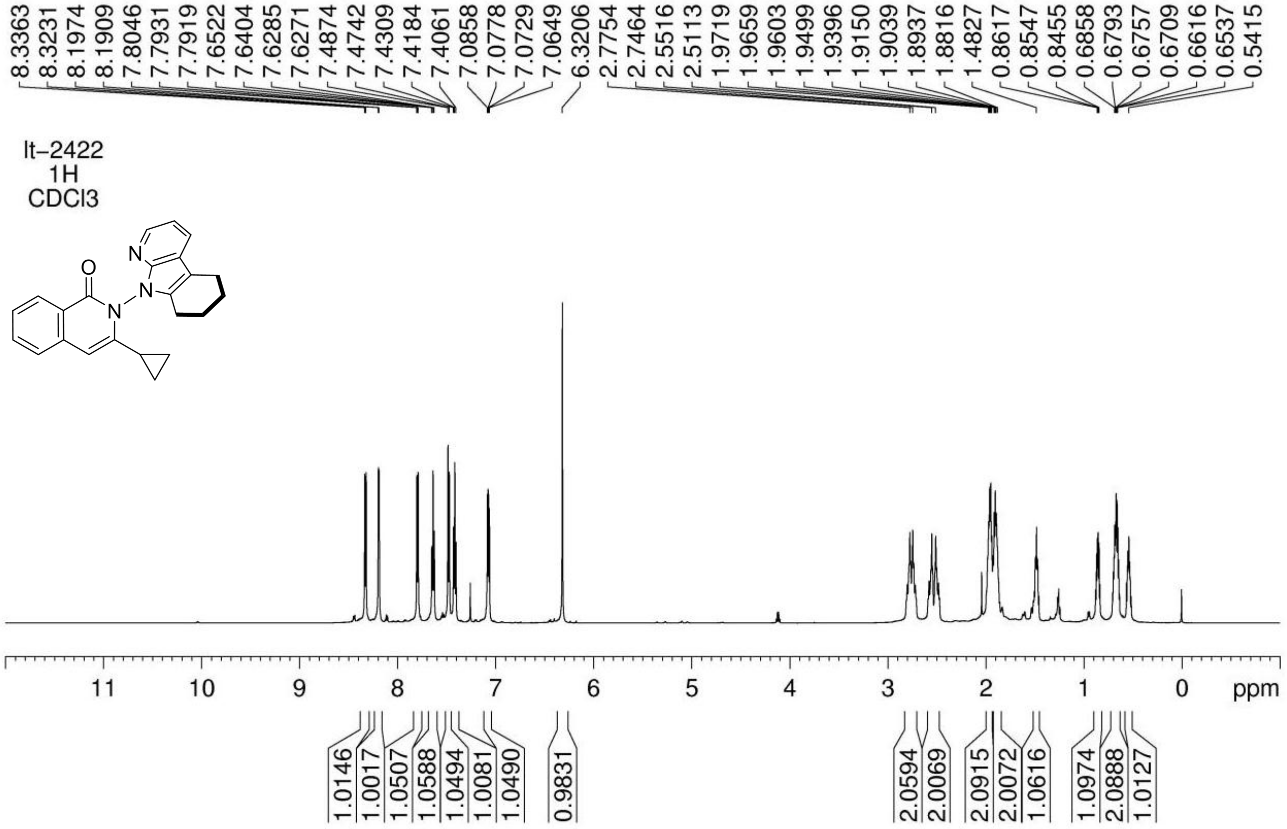
8.3363
8.3231
8.1974
8.1909
7.8046
7.7931
7.7919
7.6522
7.6404
7.6285
7.6271
7.4874
7.4742
7.4309
7.4184
7.4061
7.0858
7.0778
7.0729
7.0649
6.3206
2.7754
2.7464
2.5516
2.5113
1.9719
1.9659
1.9603
1.9499
1.9396
1.9150
1.9039
1.8937
1.8816
1.4827
0.8617
0.8547
0.8455
0.6858
0.6793
0.6757
0.6709
0.6616
0.6537
0.5415
lt–2422
1H
CDCl3
N
O
N
N
11
10
9
8
7
6
5
4
3
2
1
0
ppm
1.0146
1.0017
1.0507
1.0588
1.0494
1.0081
1.0490
0.9831
2.0594
2.0069
2.0915
2.0072
1.0616
1.0974
2.0888
1.0127

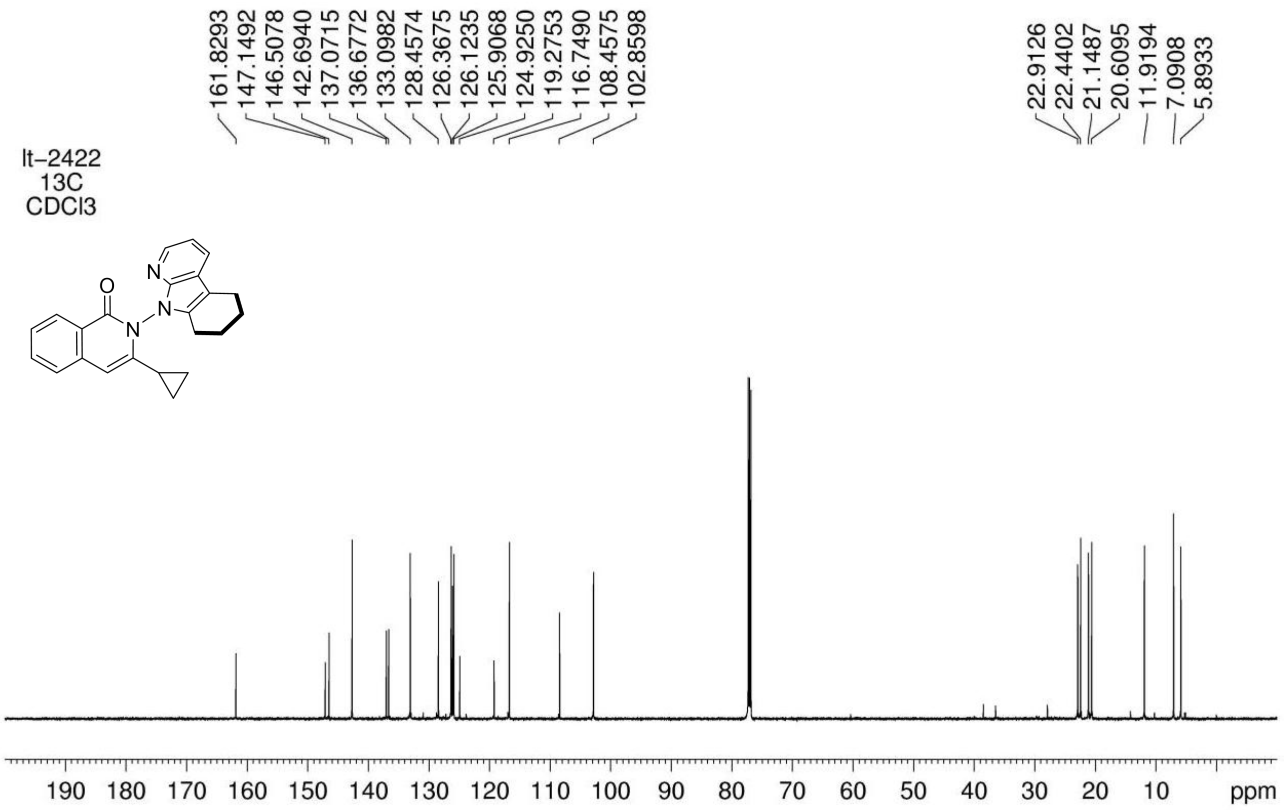
161.8293
147.1492
146.5078
142.6940
137.0715
136.6772
133.0982
128.4574
126.3675
126.1235
125.9068
124.9250
119.2753
116.7490
108.4575
102.8598
22.9126
22.4402
21.1487
20.6095
11.9194
7.0908
5.8933
lt–2422
13C
CDCl3
N
O
N
N
190
180
170
160
150
140
130
120
110
100
90
80
70
60
50
40
30
20
10
ppm

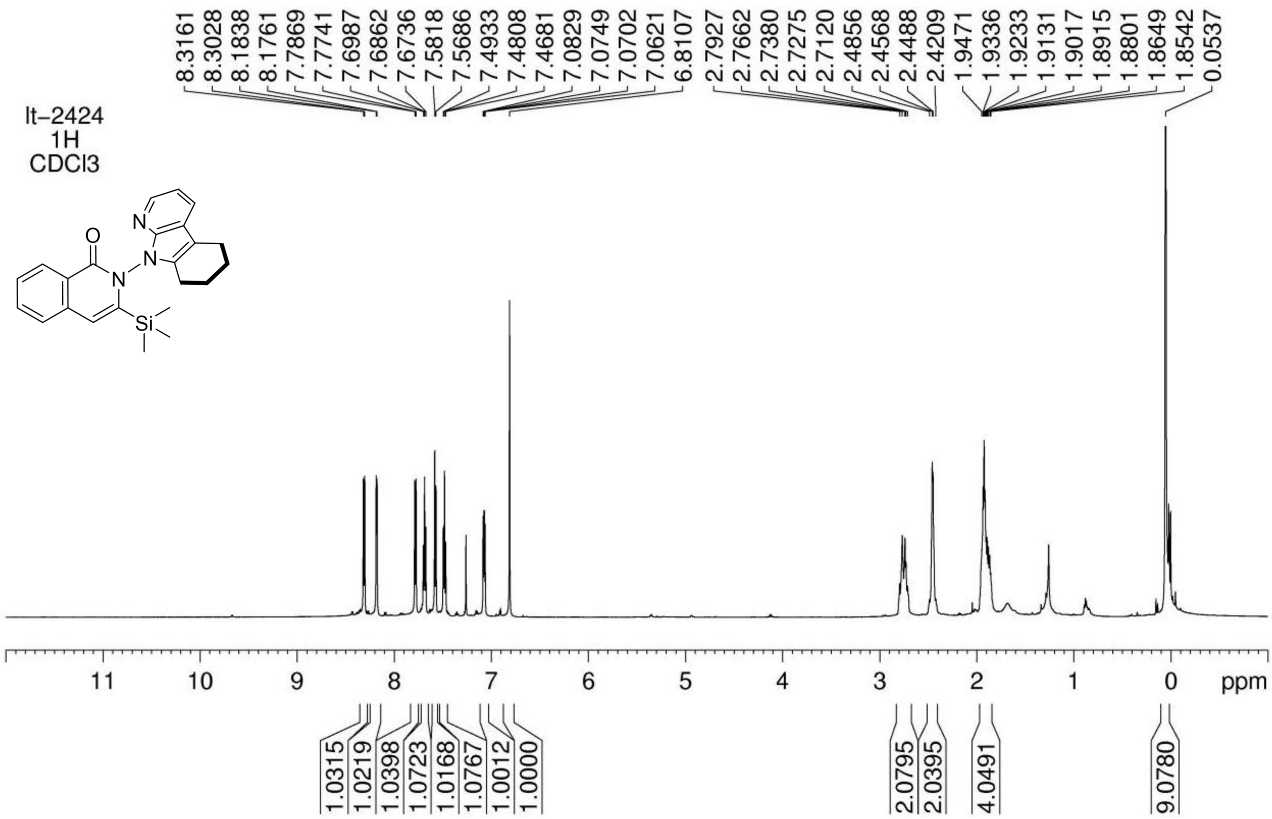
lt-2424
1H
CDCl3
O
N
N
N
Si
8.3161 8.3028 8.1838 8.1761 7.7869 7.7741 7.6987 7.6862 7.6736 7.5818 7.5686 7.4933 7.4808 7.4681 7.0829 7.0749 7.0702 7.0621 6.8107 2.7927 2.7662 2.7380 2.7275 2.7120 2.4856 2.4568 2.4488 2.4209 1.9471 1.9336 1.9233 1.9131 1.9017 1.8915 1.8801 1.8649 1.8542 0.0537
11 10 9 8 7 6 5 4 3 2 1 0 ppm
1.0315 1.0219 1.0398 1.0723 1.0168 1.0767 1.0012 1.0000 2.0795 2.0395 4.0491 9.0780

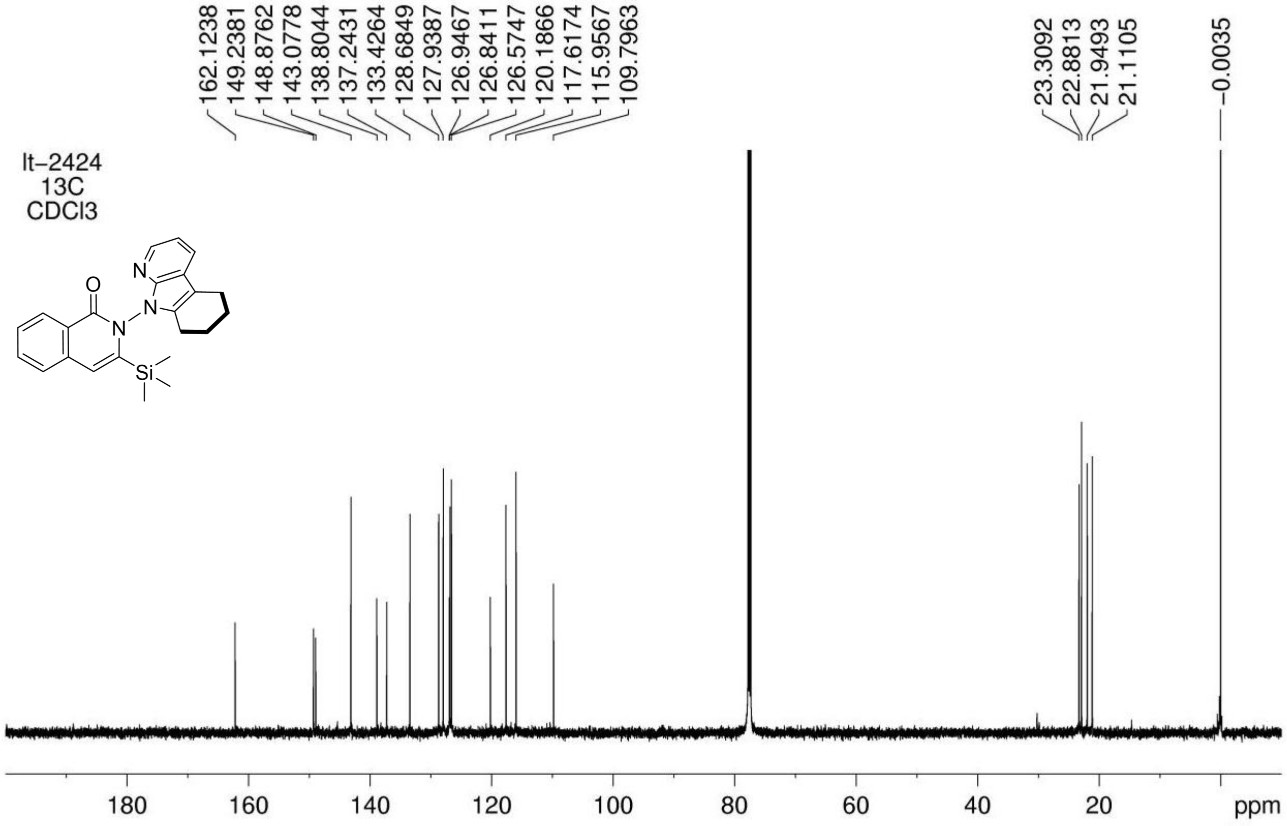
lt-2424
13C
CDCl3
O
N
N
N
Si
162.1238 149.2381 148.8762 143.0778 138.8044 137.2431 133.4264 128.6849 127.9387 126.9467 126.8411 126.5747 120.1866 117.6174 115.9567 109.7963 23.3092 22.8813 21.9493 21.1105 -0.0035
180 160 140 120 100 80 60 40 20 ppm

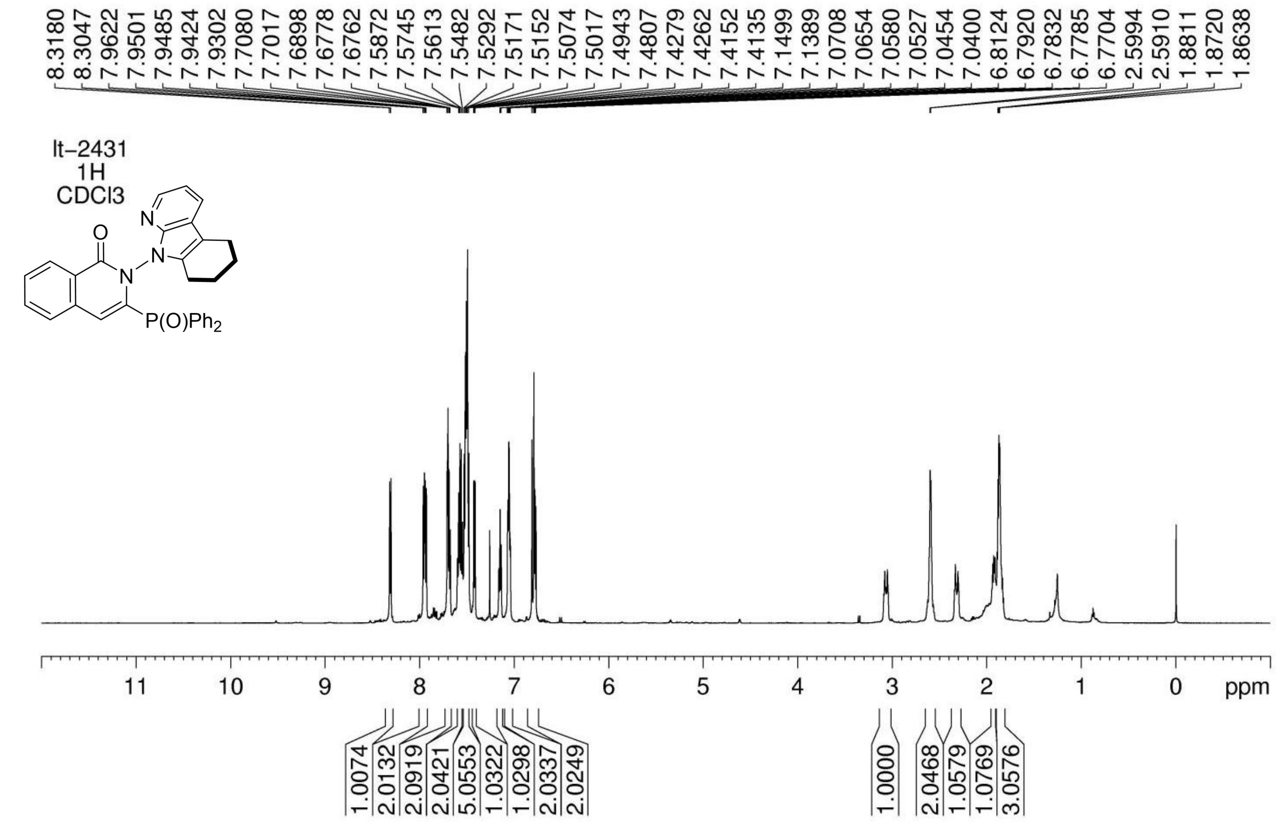
8.3180
8.3047
7.9622
7.9501
7.9485
7.9424
7.9302
7.7080
7.7017
7.6898
7.6778
7.6762
7.5872
7.5745
7.5613
7.5482
7.5292
7.5171
7.5152
7.5074
7.5017
7.4943
7.4807
7.4279
7.4262
7.4152
7.4135
7.1499
7.1389
7.0708
7.0654
7.0580
7.0527
7.0454
7.0400
6.8124
6.7920
6.7832
6.7785
6.7704
2.5994
2.5910
1.8811
1.8720
1.8638
lt–2431
1H
CDCl3
O
N
N
N
P(O)Ph2
11
10
9
8
7
6
5
4
3
2
1
0
ppm
1.0074
2.0132
2.0919
2.0421
5.0553
1.0322
1.0298
2.0337
2.0249
1.0000
2.0468
1.0579
1.0769
3.0576

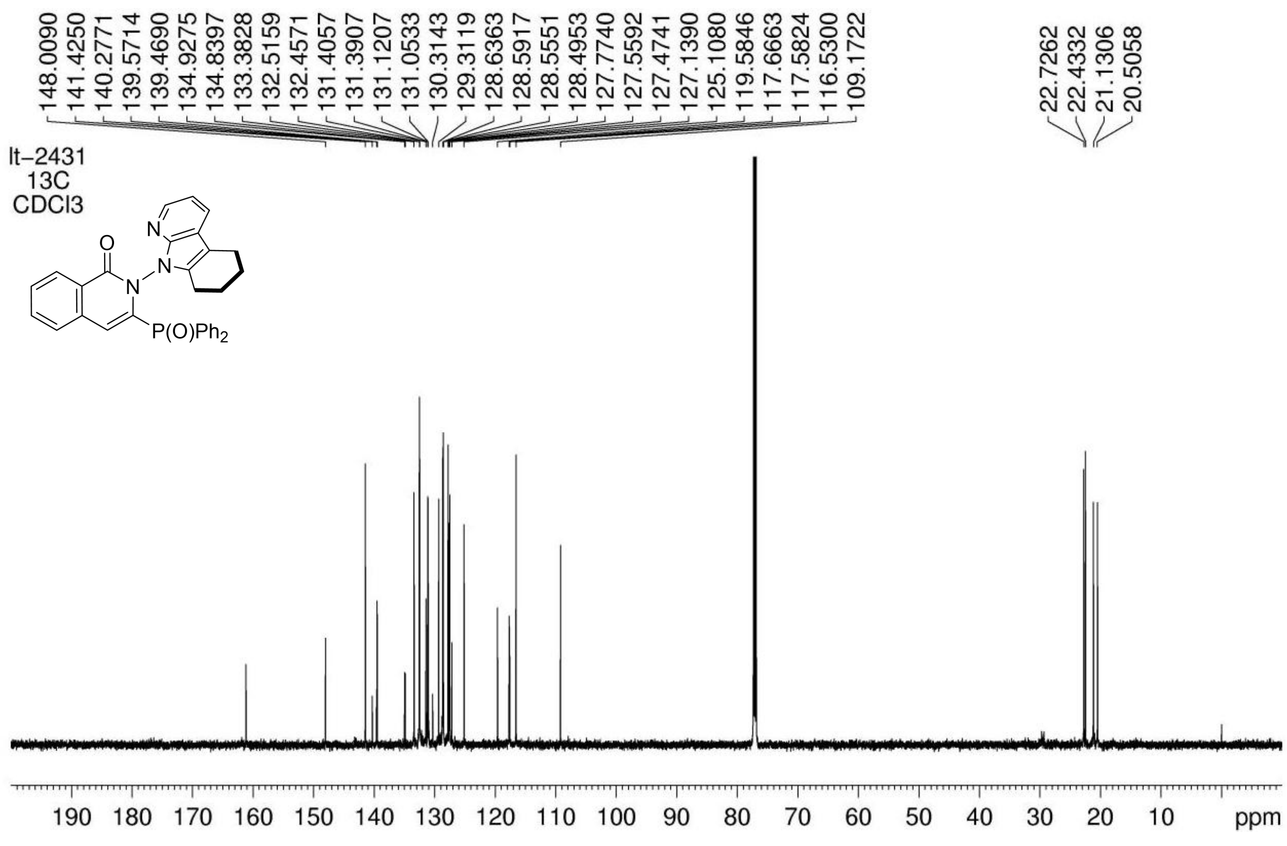
148.0090
141.4250
140.2771
139.5714
139.4690
134.9275
134.8397
133.3828
132.5159
132.4571
131.4057
131.3907
131.1207
131.0533
130.3143
129.3119
128.6363
128.5917
128.5551
128.4953
127.7740
127.5592
127.4741
127.1390
125.1080
119.5846
117.6663
117.5824
116.5300
109.1722
22.7262
22.4332
21.1306
20.5058
lt–2431
13C
CDCl3
O
N
N
N
P(O)Ph2
190
180
170
160
150
140
130
120
110
100
90
80
70
60
50
40
30
20
10
ppm

lt–2431
31P
CDCl3

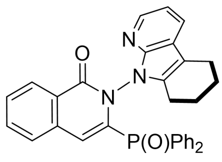

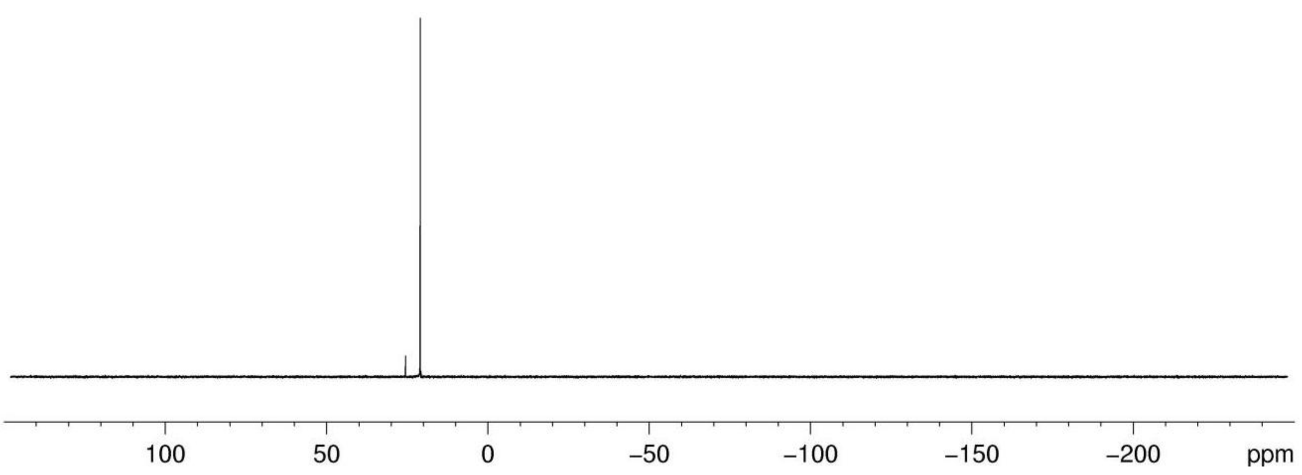

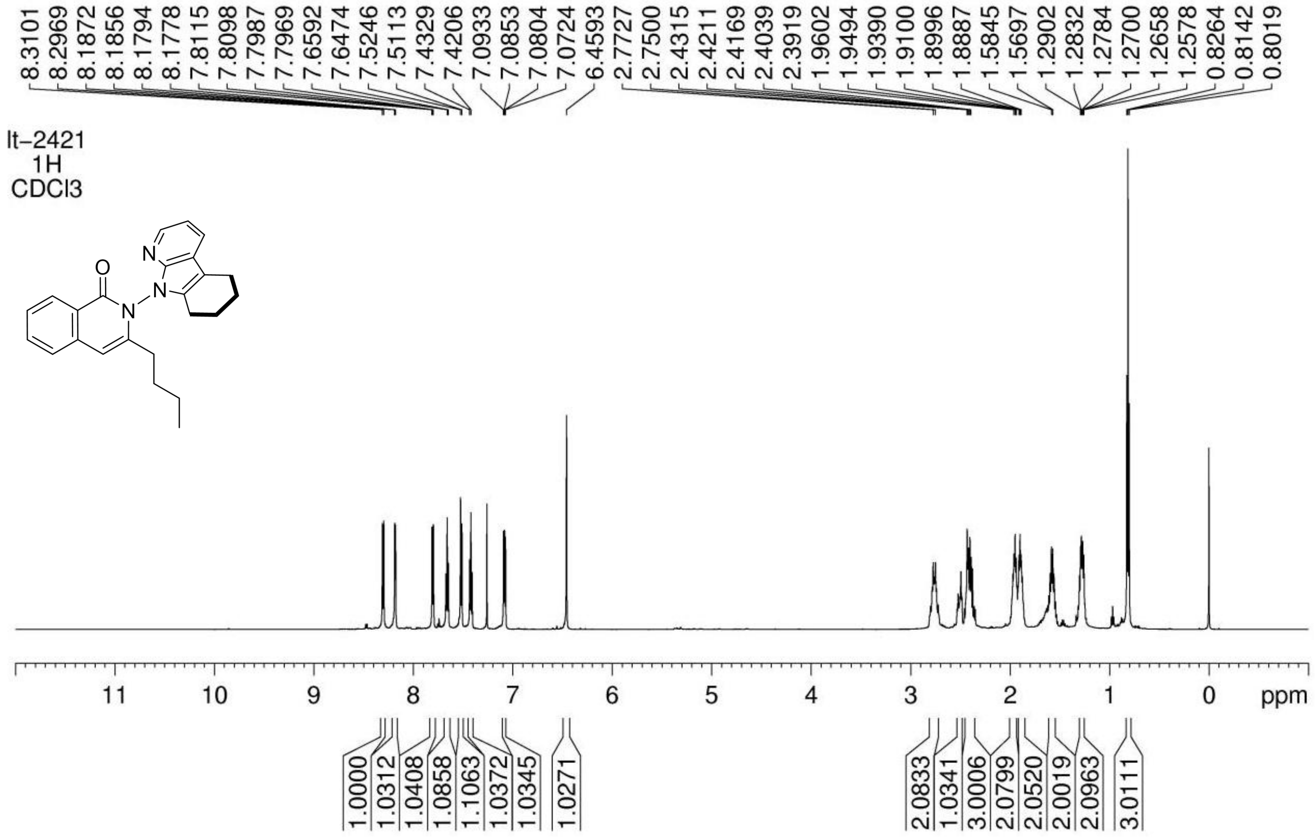
lt−2421
1H
CDCl3
8.3101
8.2969
8.1872
8.1856
8.1794
8.1778
7.8115
7.8098
7.7987
7.7969
7.6592
7.6474
7.5246
7.5113
7.4329
7.4206
7.0933
7.0853
7.0804
7.0724
6.4593
2.7727
2.7500
2.4315
2.4211
2.4169
2.4039
2.3919
1.9602
1.9494
1.9390
1.9100
1.8996
1.8887
1.5845
1.5697
1.2902
1.2832
1.2784
1.2700
1.2658
1.2578
0.8264
0.8142
0.8019
11
10
9
8
7
6
5
4
3
2
1
0
ppm
1.0000
1.0312
1.0408
1.0858
1.1063
1.0372
1.0345
1.0271
2.0833
1.0341
3.0006
2.0799
2.0520
2.0019
2.0963
3.0111

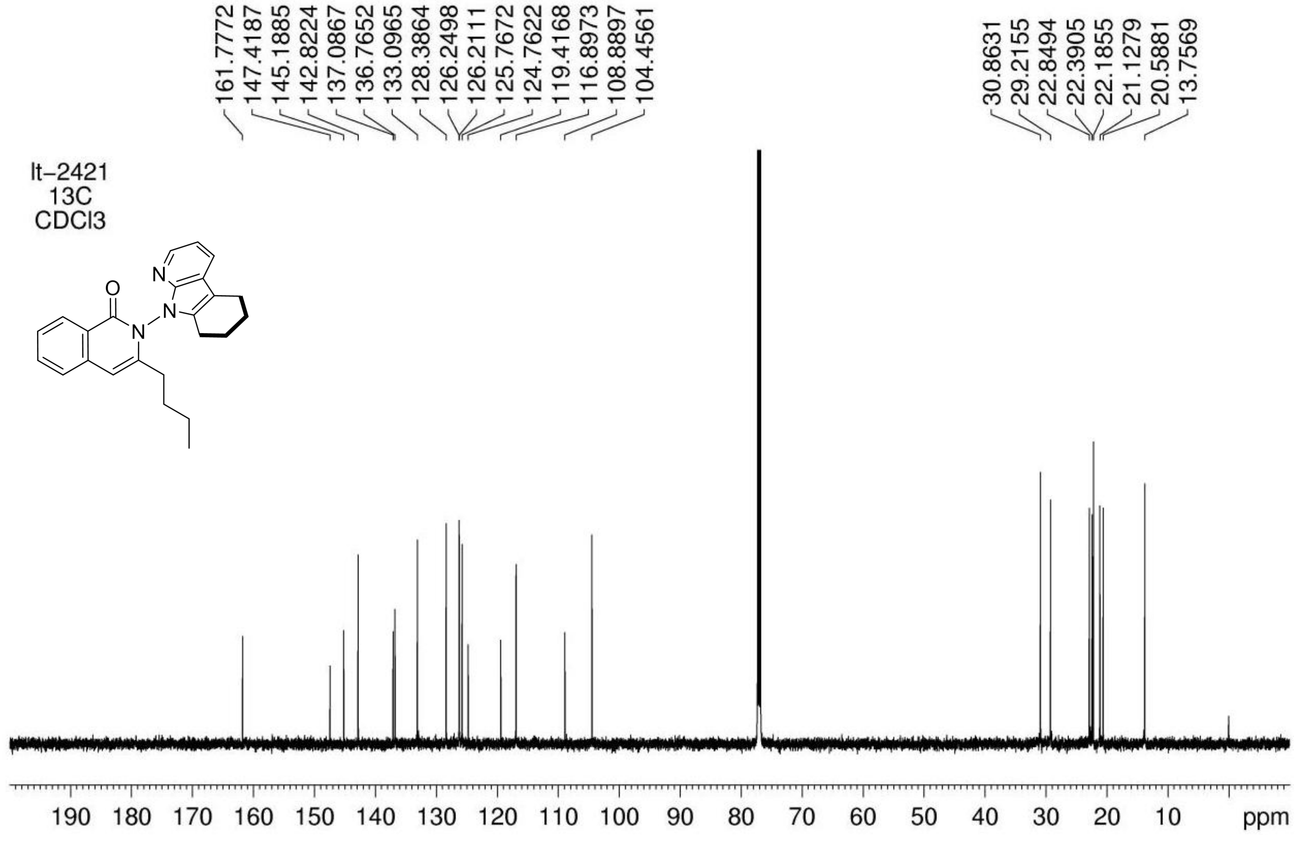
lt−2421
13C
CDCl3
161.7772
147.4187
145.1885
142.8224
137.0867
136.7652
133.0965
128.3864
126.2498
126.2111
125.7672
124.7622
119.4168
116.8973
108.8897
104.4561
30.8631
29.2155
22.8494
22.3905
22.1855
21.1279
20.5881
13.7569
190
180
170
160
150
140
130
120
110
100
90
80
70
60
50
40
30
20
10
ppm

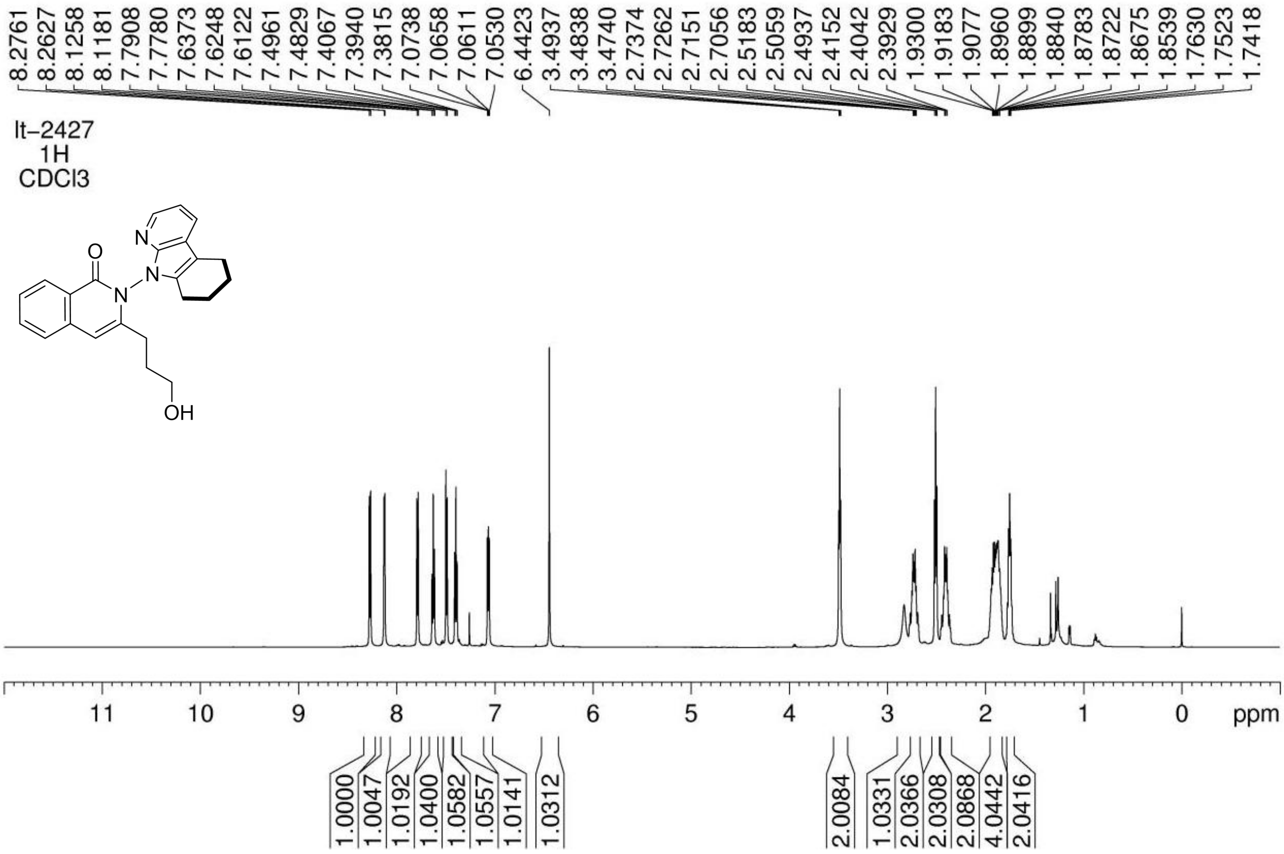
8.2761
8.2627
8.1258
8.1181
7.7908
7.7780
7.6373
7.6248
7.6122
7.4961
7.4829
7.4067
7.3940
7.3815
7.0738
7.0658
7.0611
7.0530
6.4423
3.4937
3.4838
3.4740
2.7374
2.7262
2.7151
2.7056
2.5183
2.5059
2.4937
2.4152
2.4042
2.3929
1.9300
1.9183
1.9077
1.8960
1.8899
1.8840
1.8783
1.8722
1.8675
1.8539
1.7630
1.7523
1.7418
lt–2427
1H
CDCl3
O
N
N
N
OH
11 10 9 8 7 6 5 4 3 2 1 0 ppm
1.0000
1.0047
1.0192
1.0400
1.0582
1.0557
1.0141
1.0312
2.0084
1.0331
2.0366
2.0308
2.0868
4.0442
2.0416

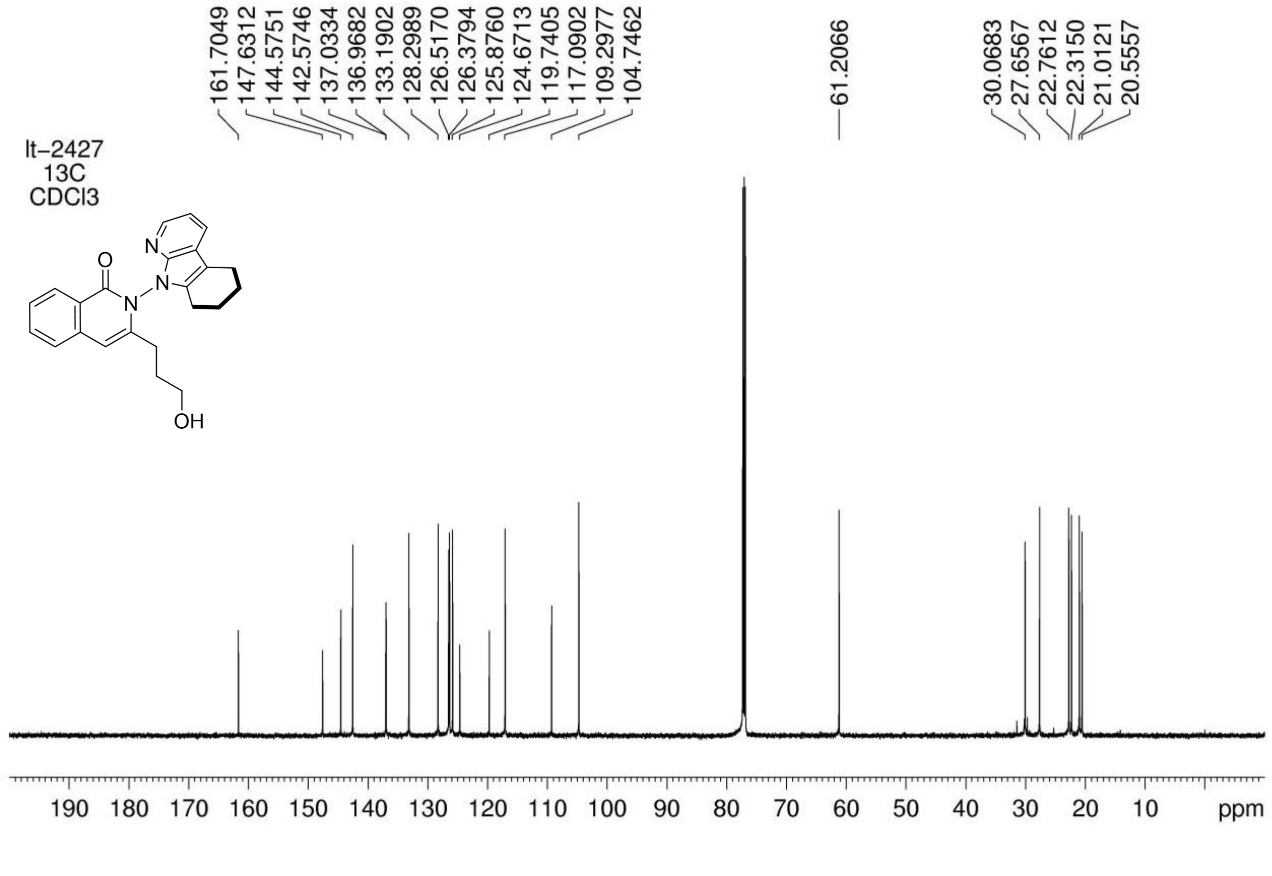
161.7049
147.6312
144.5751
142.5746
137.0334
136.9682
133.1902
128.2989
126.5170
126.3794
125.8760
124.6713
119.7405
117.0902
109.2977
104.7462
61.2066
30.0683
27.6567
22.7612
22.3150
21.0121
20.5557
lt–2427
13C
CDCl3
O
N
N
N
OH
190 180 170 160 150 140 130 120 110 100 90 80 70 60 50 40 30 20 10 ppm

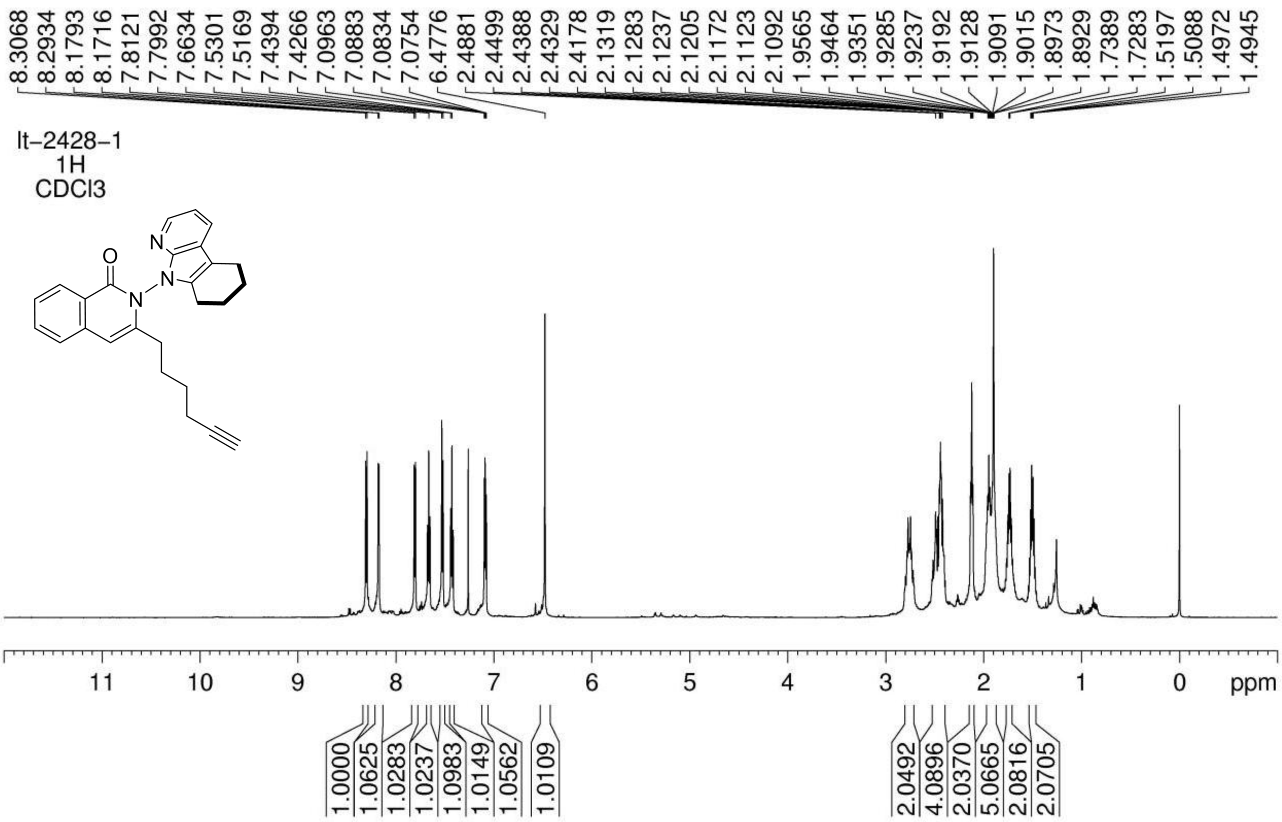
lt−2428−1
1H
CDCl3
N
O
N
N
8.3068
8.2934
8.1793
8.1716
7.8121
7.7992
7.6634
7.5301
7.5169
7.4394
7.4266
7.0963
7.0883
7.0834
7.0754
6.4776
2.4881
2.4499
2.4388
2.4329
2.4178
2.1319
2.1283
2.1237
2.1205
2.1172
2.1123
2.1092
1.9565
1.9464
1.9351
1.9285
1.9237
1.9192
1.9128
1.9091
1.9015
1.8973
1.8929
1.7389
1.7283
1.5197
1.5088
1.4972
1.4945
11 10 9 8 7 6 5 4 3 2 1 0 ppm
1.0000
1.0625
1.0283
1.0237
1.0983
1.0149
1.0562
1.0109
2.0492
4.0896
2.0370
5.0665
2.0816
2.0705

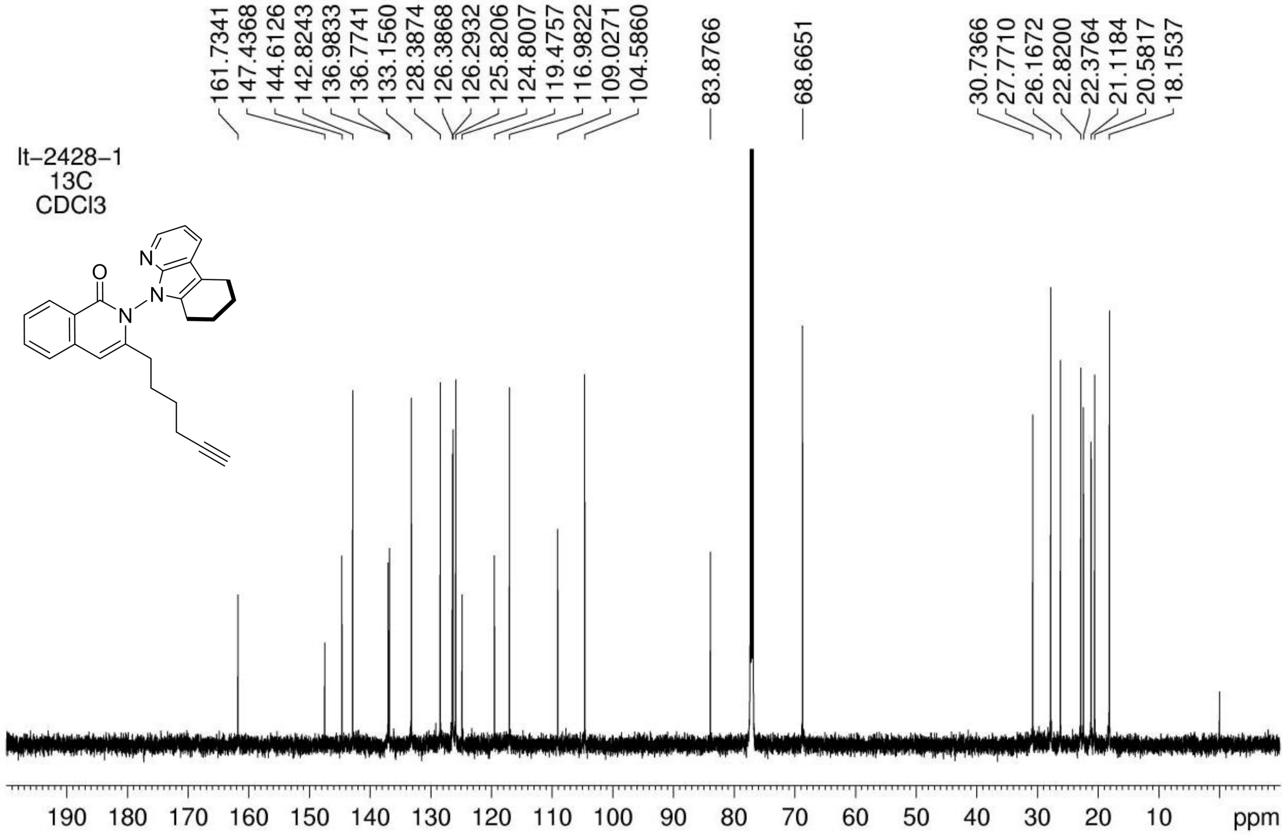
lt−2428−1
13C
CDCl3
N
O
N
N
161.7341
147.4368
144.6126
142.8243
136.9833
136.7741
133.1560
128.3874
126.3868
126.2932
125.8206
124.8007
119.4757
116.9822
109.0271
104.5860
83.8766
68.6651
30.7366
27.7710
26.1672
22.8200
22.3764
21.1184
20.5817
18.1537
190 180 170 160 150 140 130 120 110 100 90 80 70 60 50 40 30 20 10 ppm

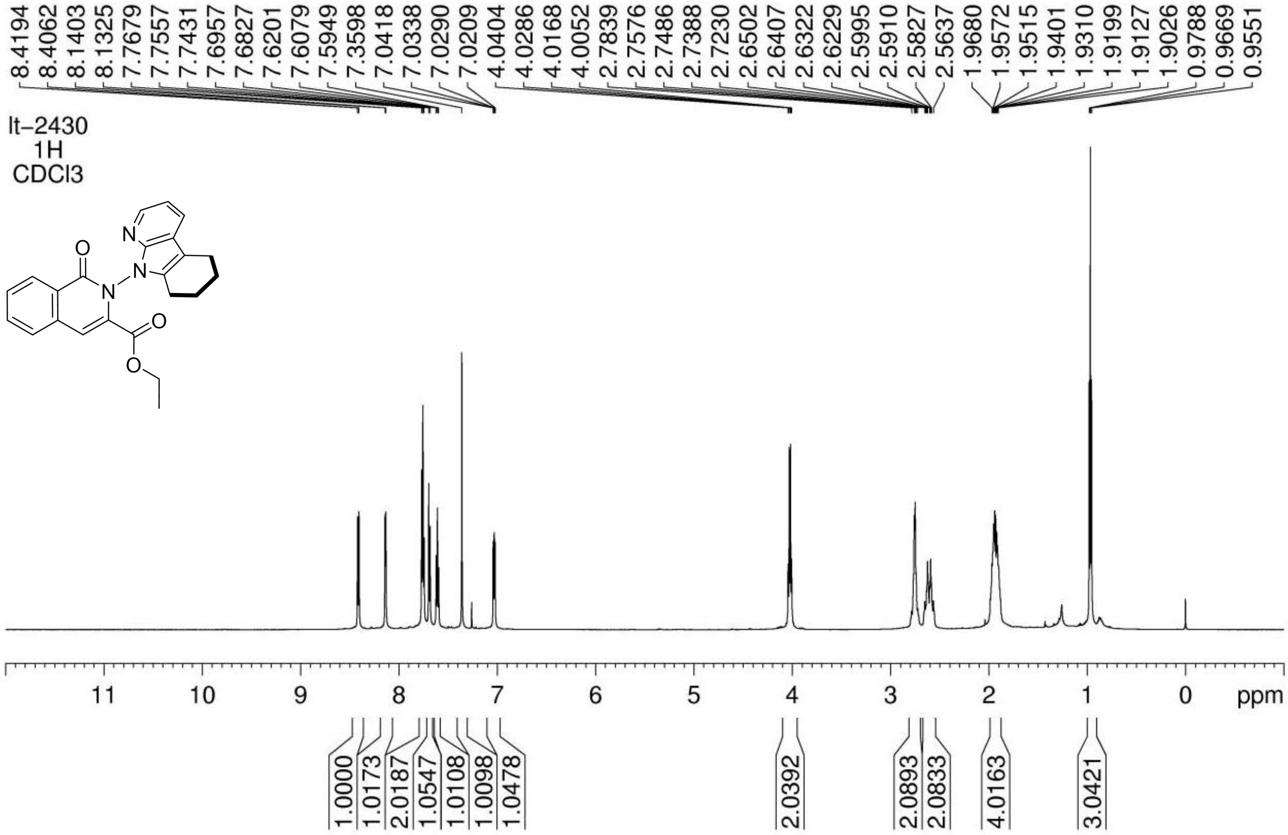
8.4194 8.4062 8.1403 8.1325 7.7679 7.7557 7.7431 7.6957 7.6827 7.6201 7.6079 7.5949 7.3598 7.0418 7.0338 7.0290 7.0209 4.0404 4.0286 4.0168 4.0052 2.7839 2.7576 2.7486 2.7388 2.7230 2.6502 2.6407 2.6322 2.6229 2.5995 2.5910 2.5827 2.5637 1.9680 1.9572 1.9515 1.9401 1.9310 1.9199 1.9127 1.9026 0.9788 0.9669 0.9551
lt−2430
1H
CDCl3
O
N
N
N
O
O
11 10 9 8 7 6 5 4 3 2 1 0 ppm
1.0000 1.0173 2.0187 1.0547 1.0108 1.0098 1.0478 2.0392 2.0893 2.0833 4.0163 3.0421

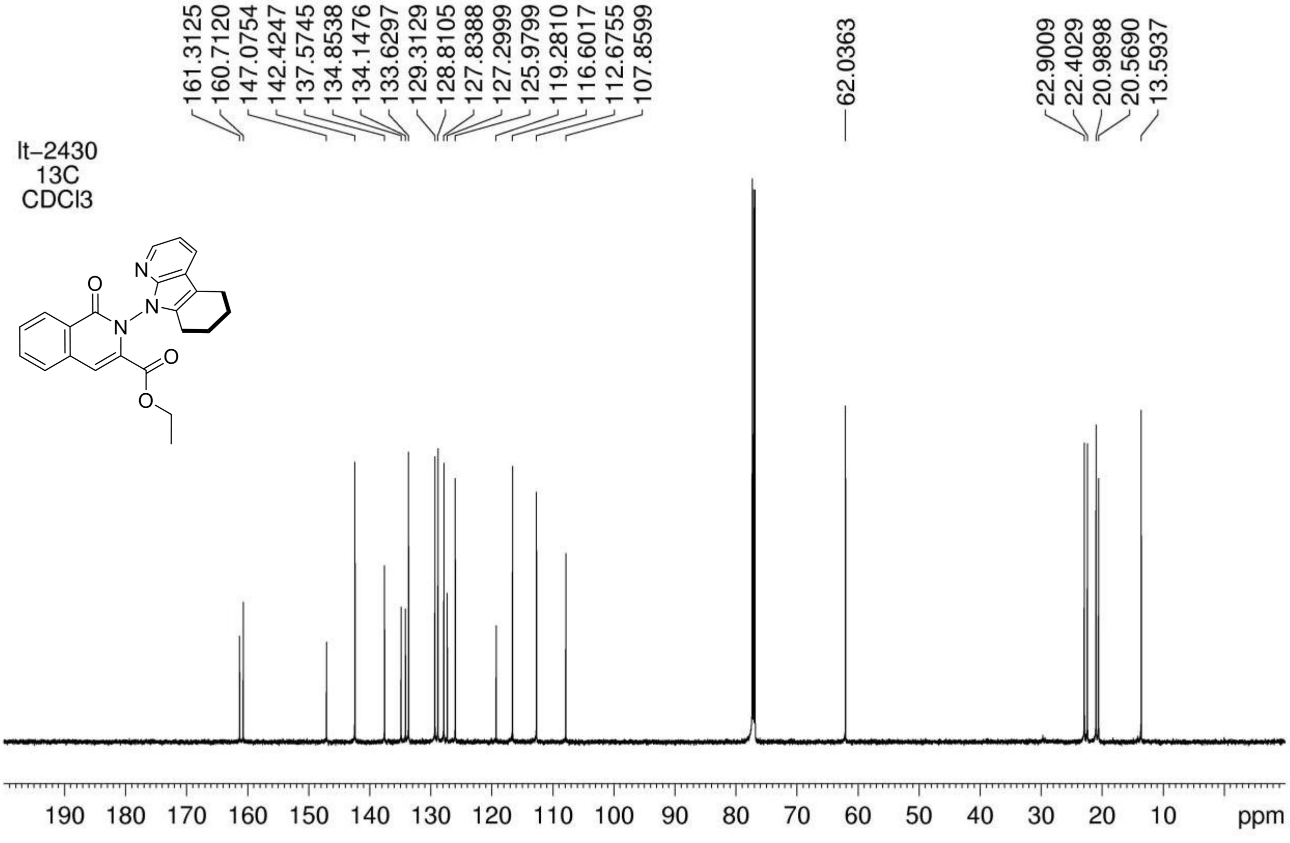
161.3125 160.7120 147.0754 142.4247 137.5745 134.8538 134.1476 133.6297 129.3129 128.8105 127.8388 127.2999 125.9799 119.2810 116.6017 112.6755 107.8599
62.0363
22.9009 22.4029 20.9898 20.5690 13.5937
lt−2430
13C
CDCl3
O
N
N
N
O
O
190 180 170 160 150 140 130 120 110 100 90 80 70 60 50 40 30 20 10 ppm

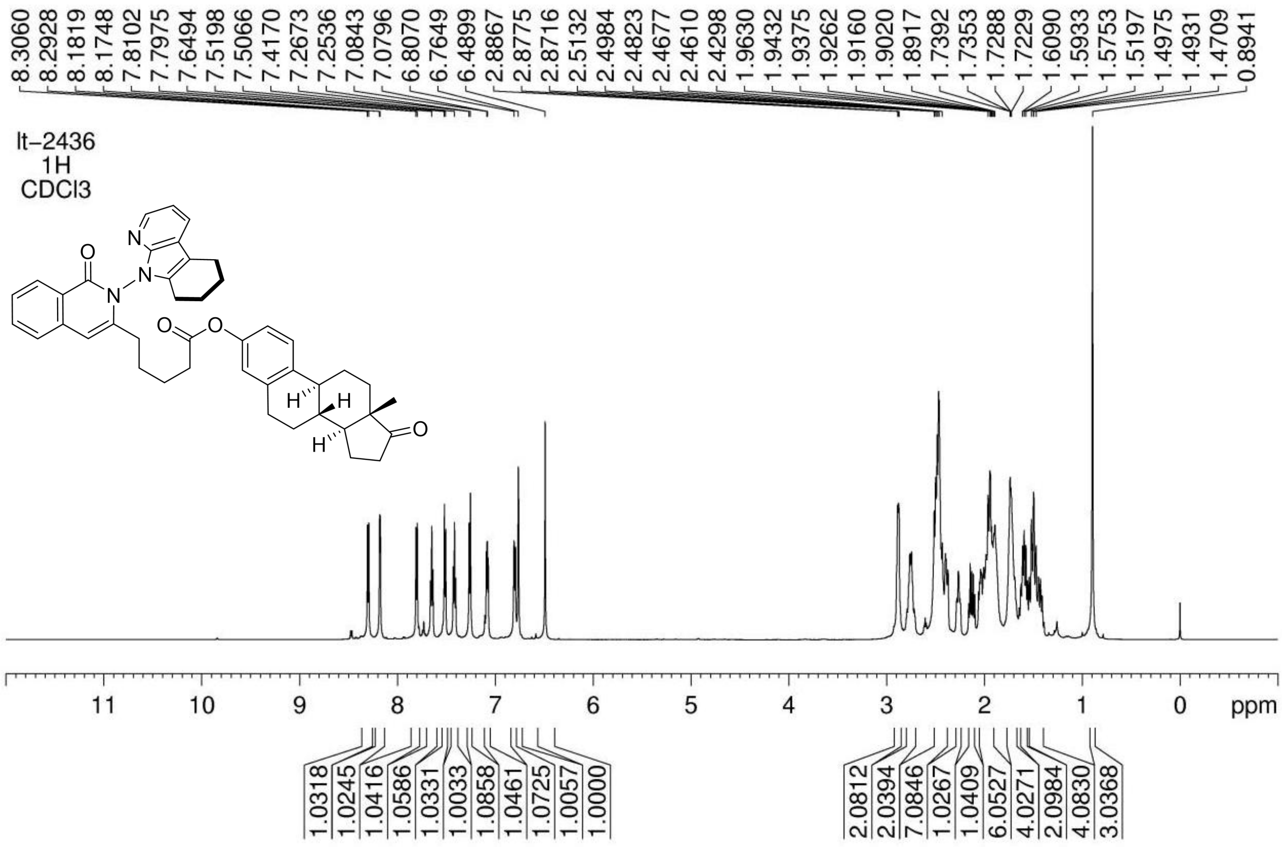
lt–2436
1H
CDCl3
8.3060
8.2928
8.1819
8.1748
7.8102
7.7975
7.6494
7.5198
7.5066
7.4170
7.2673
7.2536
7.0843
7.0796
6.8070
6.7649
6.4899
2.8867
2.8775
2.8716
2.5132
2.4984
2.4823
2.4677
2.4610
2.4298
1.9630
1.9432
1.9375
1.9262
1.9160
1.9020
1.8917
1.7392
1.7353
1.7288
1.7229
1.6090
1.5933
1.5753
1.5197
1.4975
1.4931
1.4709
0.8941
1.0318
1.0245
1.0416
1.0586
1.0331
1.0033
1.0858
1.0461
1.0725
1.0057
1.0000
2.0812
2.0394
7.0846
1.0267
1.0409
6.0527
4.0271
2.0984
4.0830
3.0368
ppm

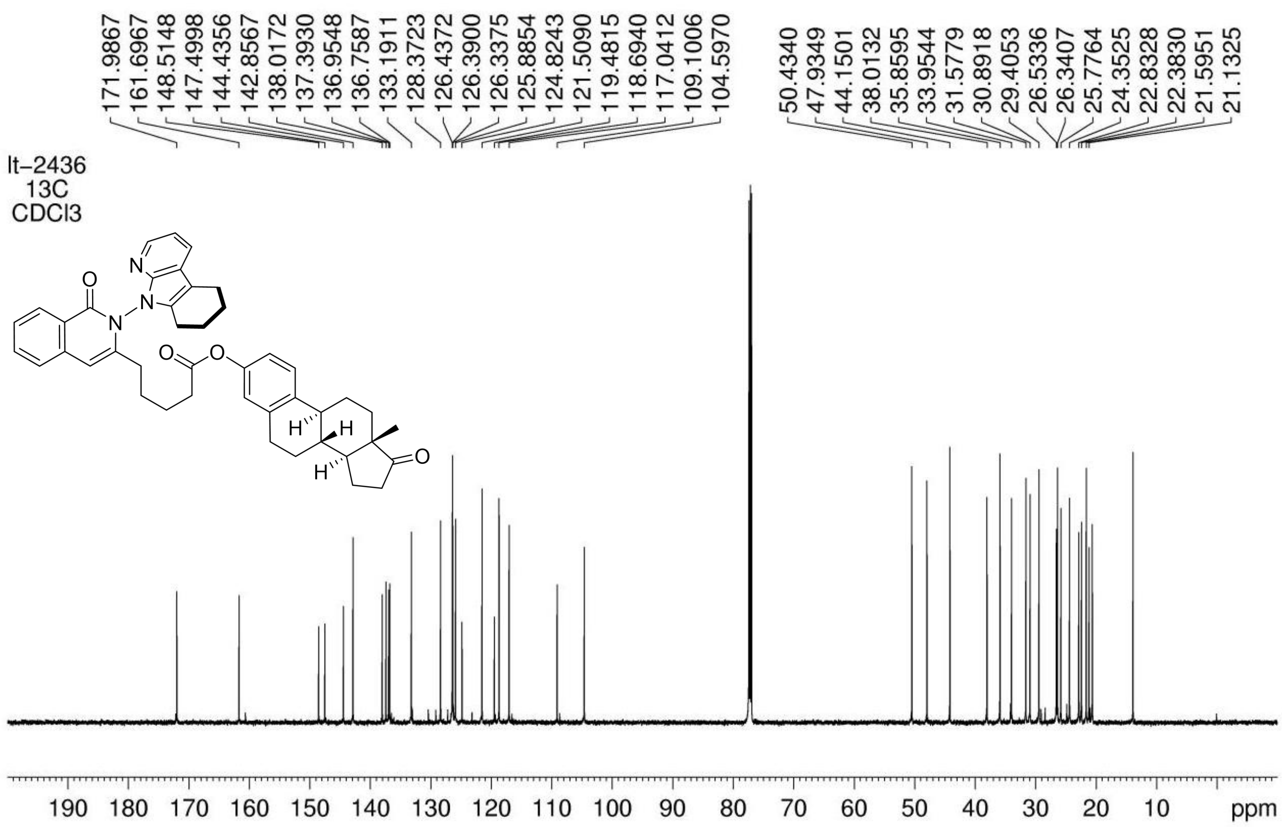
lt–2436
13C
CDCl3
171.9867
161.6967
148.5148
147.4998
144.4356
142.8567
138.0172
137.3930
136.9548
136.7587
133.1911
128.3723
126.4372
126.3900
126.3375
125.8854
124.8243
121.5090
119.4815
118.6940
117.0412
109.1006
104.5970
50.4340
47.9349
44.1501
38.0132
35.8595
33.9544
31.5779
30.8918
29.4053
26.5336
26.3407
25.7764
24.3525
22.8328
22.3830
21.5951
21.1325
ppm

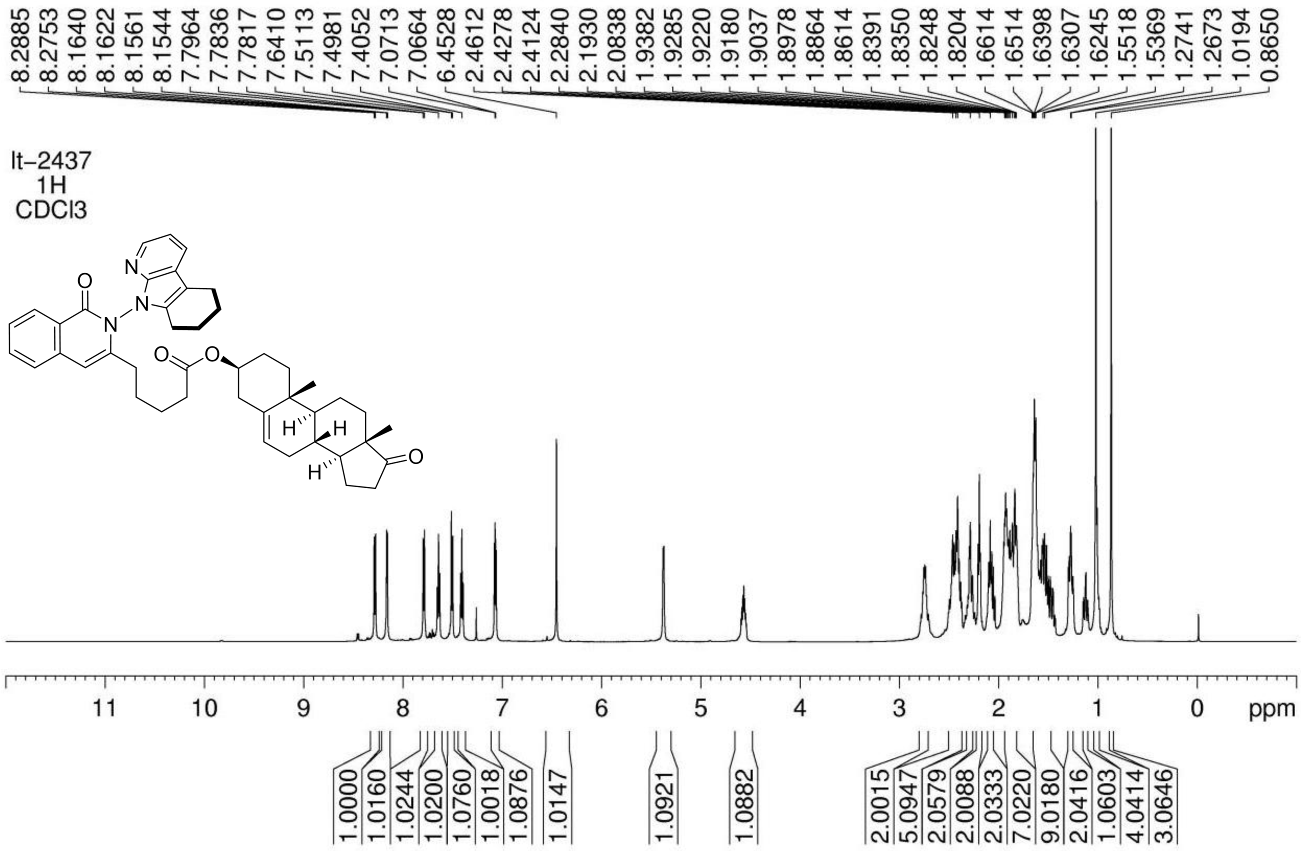
lt-2437
1H
CDCl3
8.2885 8.2753 8.1640 8.1622 8.1561 8.1544 7.7964 7.7836 7.7817 7.6410 7.5113 7.4981 7.4052 7.0713 7.0664 6.4528 2.4612 2.4278 2.4124 2.2840 2.1930 2.0838 1.9382 1.9285 1.9220 1.9180 1.9037 1.8978 1.8864 1.8614 1.8391 1.8350 1.8248 1.8204 1.6614 1.6514 1.6398 1.6307 1.6245 1.5518 1.5369 1.2741 1.2673 1.0194 0.8650
1.0000 1.0160 1.0244 1.0200 1.0760 1.0018 1.0876 1.0147 1.0921 1.0882 2.0015 5.0947 2.0579 2.0088 2.0333 7.0220 9.0180 2.0416 1.0603 4.0414 3.0646
11 10 9 8 7 6 5 4 3 2 1 0 ppm

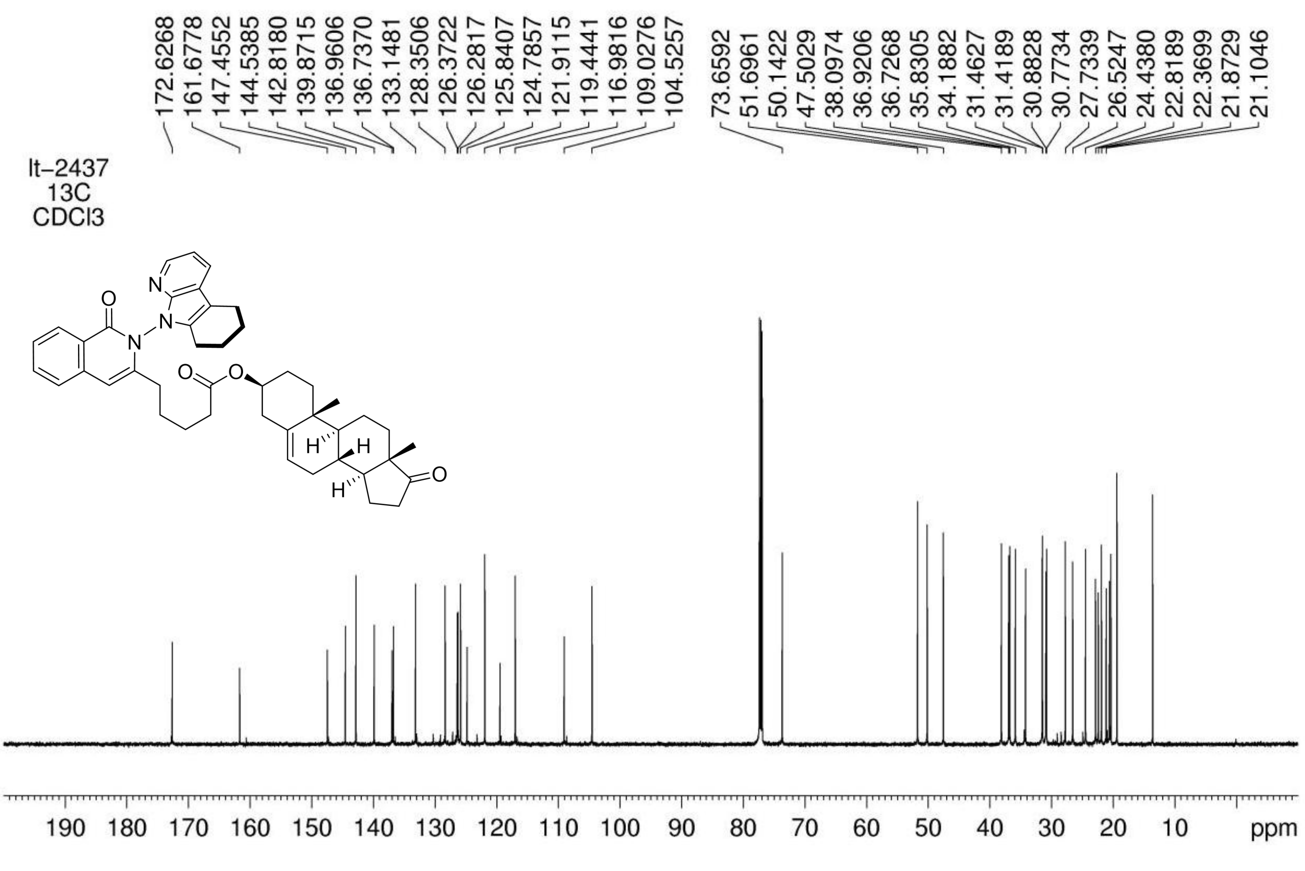
lt-2437
13C
CDCl3
172.6268 161.6778 147.4552 144.5385 142.8180 139.8715 136.9606 136.7370 133.1481 128.3506 126.3722 126.2817 125.8407 124.7857 121.9115 119.4441 116.9816 109.0276 104.5257 73.6592 51.6961 50.1422 47.5029 38.0974 36.9206 36.7268 35.8305 34.1882 31.4627 31.4189 30.8828 30.7734 27.7339 26.5247 24.4380 22.8189 22.3699 21.8729 21.1046
190 180 170 160 150 140 130 120 110 100 90 80 70 60 50 40 30 20 10 ppm

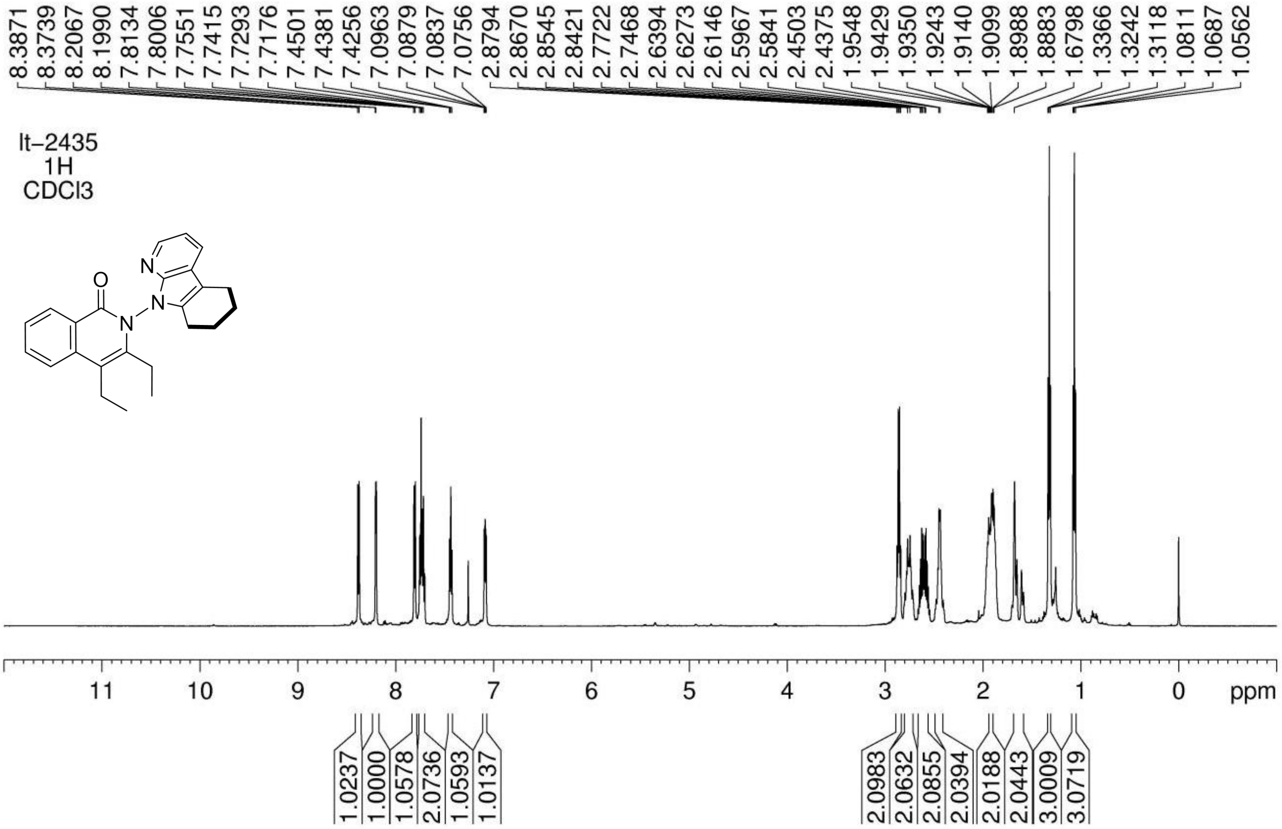
8.3871
8.3739
8.2067
8.1990
7.8134
7.8006
7.7551
7.7415
7.7293
7.7176
7.4501
7.4381
7.4256
7.0963
7.0879
7.0837
7.0756
2.8794
2.8670
2.8545
2.8421
2.7722
2.7468
2.6394
2.6273
2.6146
2.5967
2.5841
2.4503
2.4375
1.9548
1.9429
1.9350
1.9243
1.9140
1.9099
1.8988
1.8883
1.6798
1.3366
1.3242
1.3118
1.0811
1.0687
1.0562
lt–2435
1H
CDCl3
O
N
N
N
11
10
9
8
7
6
5
4
3
2
1
0
ppm
1.0237
1.0000
1.0578
2.0736
1.0593
1.0137
2.0983
2.0632
2.0855
2.0394
2.0188
2.0443
3.0009
3.0719

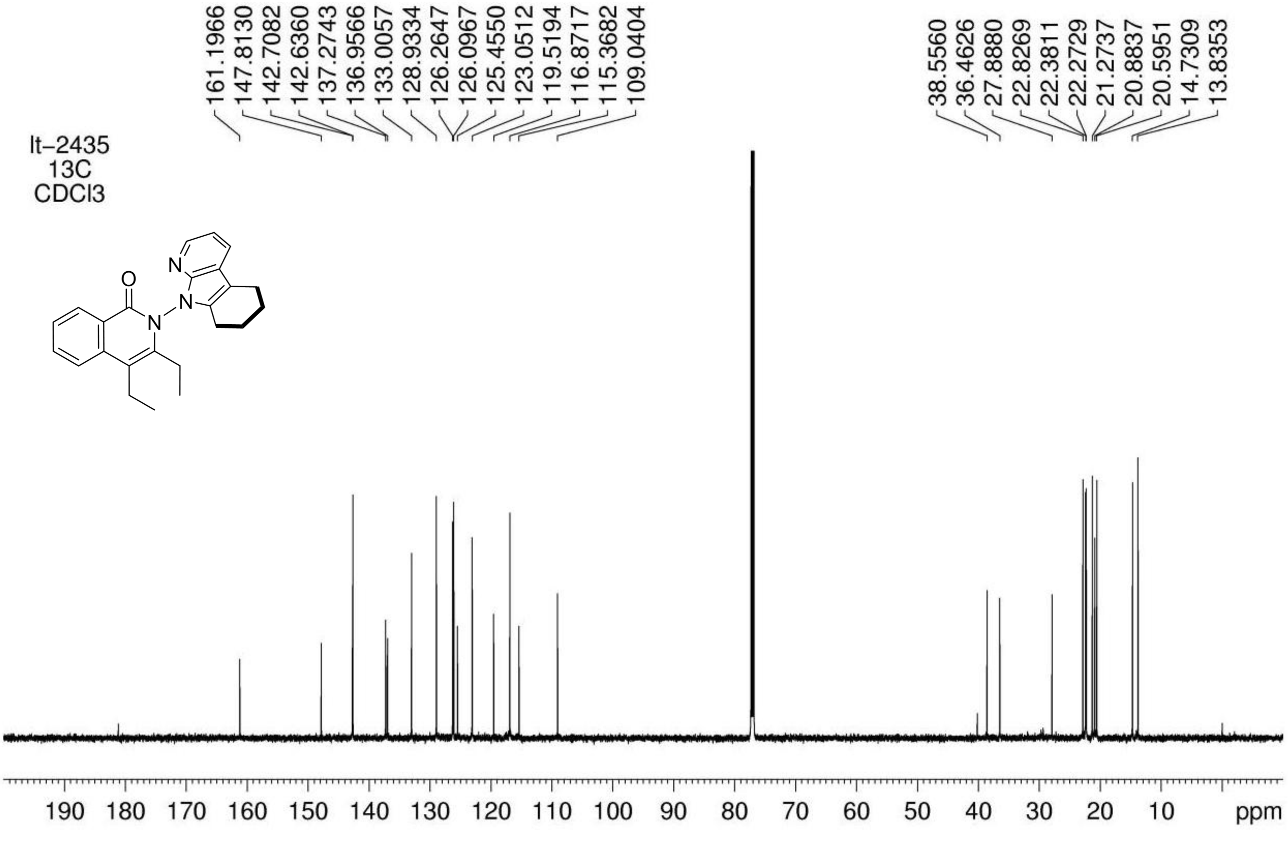
161.1966
147.8130
142.7082
142.6360
137.2743
136.9566
133.0057
128.9334
126.2647
126.0967
125.4550
123.0512
119.5194
116.8717
115.3682
109.0404
38.5560
36.4626
27.8880
22.8269
22.3811
22.2729
21.2737
20.8837
20.5951
14.7309
13.8353
lt–2435
13C
CDCl3
O
N
N
N
190
180
170
160
150
140
130
120
110
100
90
80
70
60
50
40
30
20
10
ppm

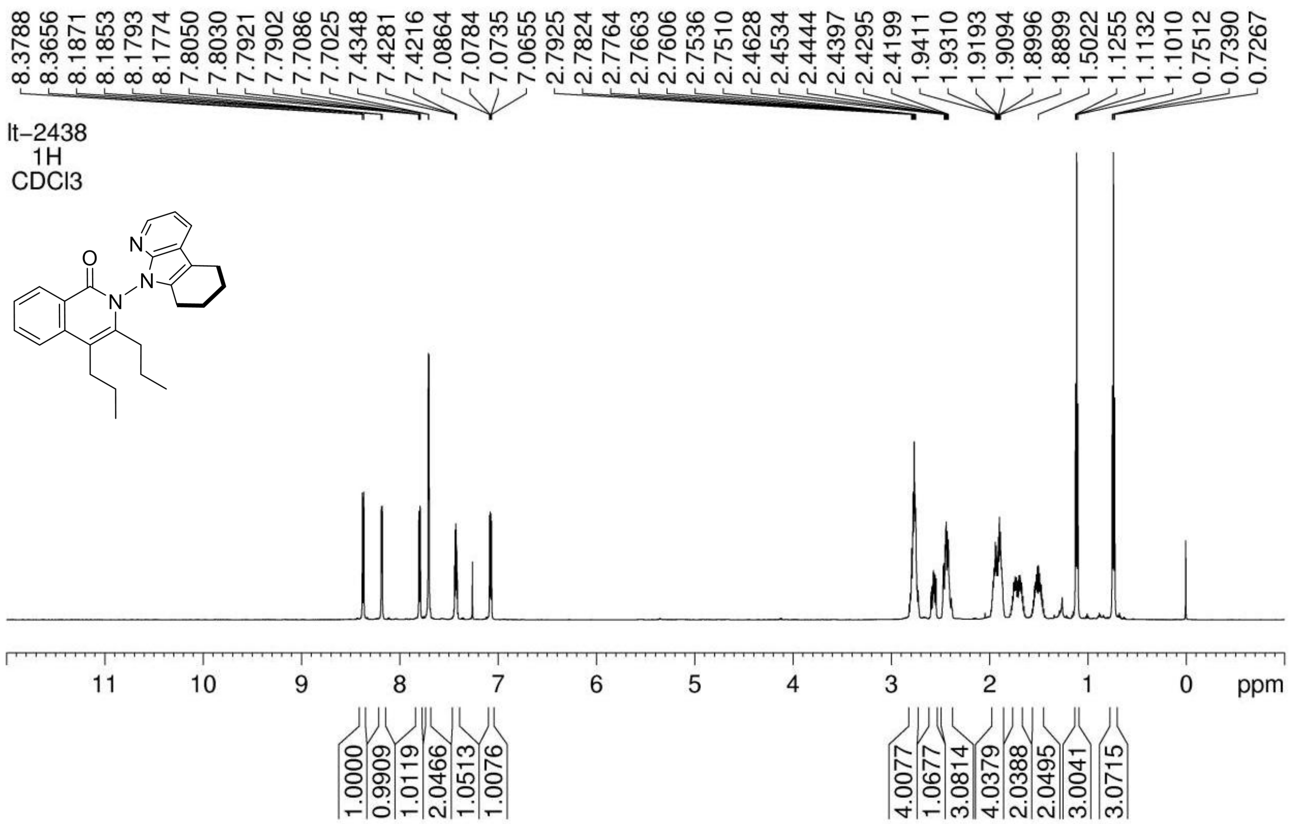
lt–2438
1H
CDCl3
N
O
N
N
8.3788 8.3656 8.1871 8.1853 8.1793 8.1774 7.8050 7.8030 7.7921 7.7902 7.7086 7.7025 7.4348 7.4281 7.4216 7.0864 7.0784 7.0735 7.0655 2.7925 2.7824 2.7764 2.7663 2.7606 2.7536 2.7510 2.4628 2.4534 2.4444 2.4397 2.4295 2.4199 1.9411 1.9310 1.9193 1.9094 1.8996 1.8899 1.5022 1.1255 1.1132 1.1010 0.7512 0.7390 0.7267
11 10 9 8 7 6 5 4 3 2 1 0 ppm
1.0000 0.9909 1.0119 2.0466 1.0513 1.0076
4.0077 1.0677 3.0814 4.0379 2.0388 2.0495 3.0041 3.0715

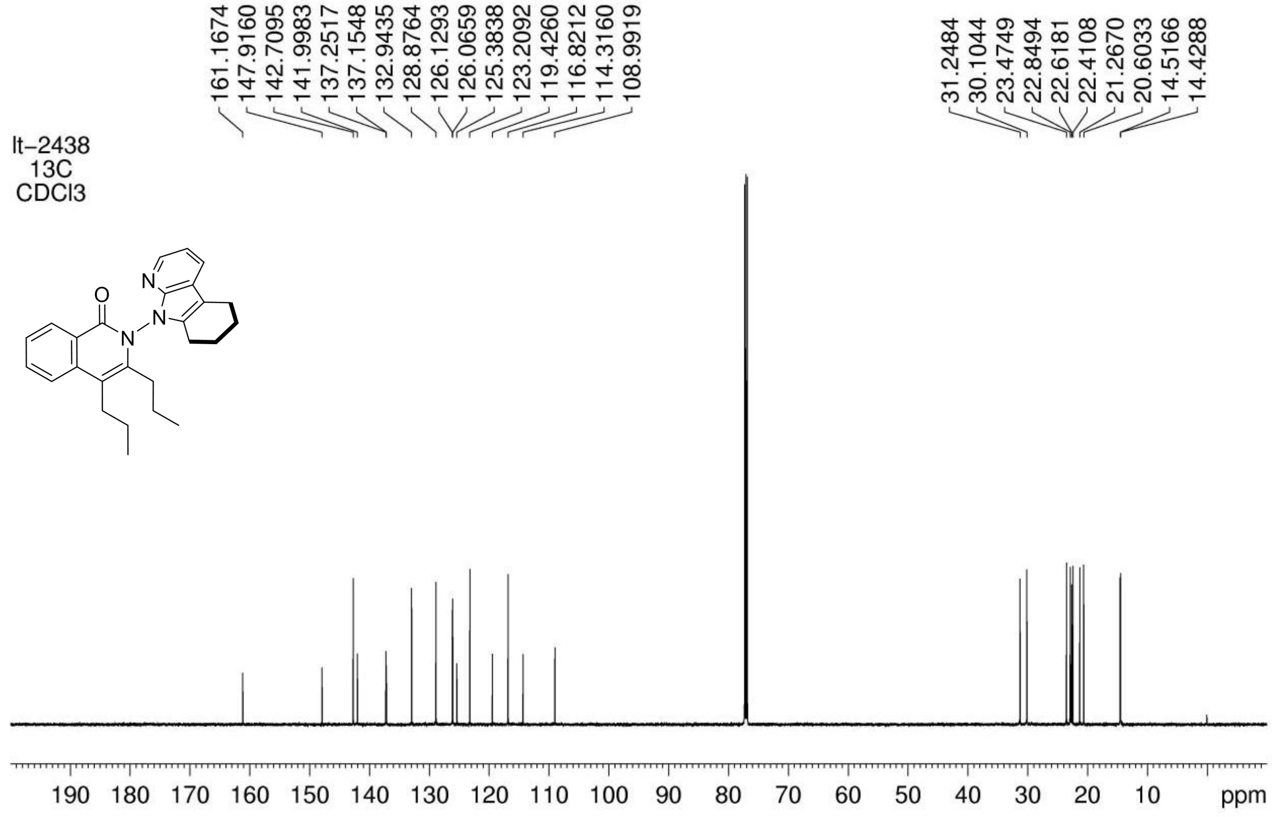
lt–2438
13C
CDCl3
N
O
N
N
161.1674 147.9160 142.7095 141.9983 137.2517 137.1548 132.9435 128.8764 126.1293 126.0659 125.3838 123.2092 119.4260 116.8212 114.3160 108.9919
31.2484 30.1044 23.4749 22.8494 22.6181 22.4108 21.2670 20.6033 14.5166 14.4288
190 180 170 160 150 140 130 120 110 100 90 80 70 60 50 40 30 20 10 ppm

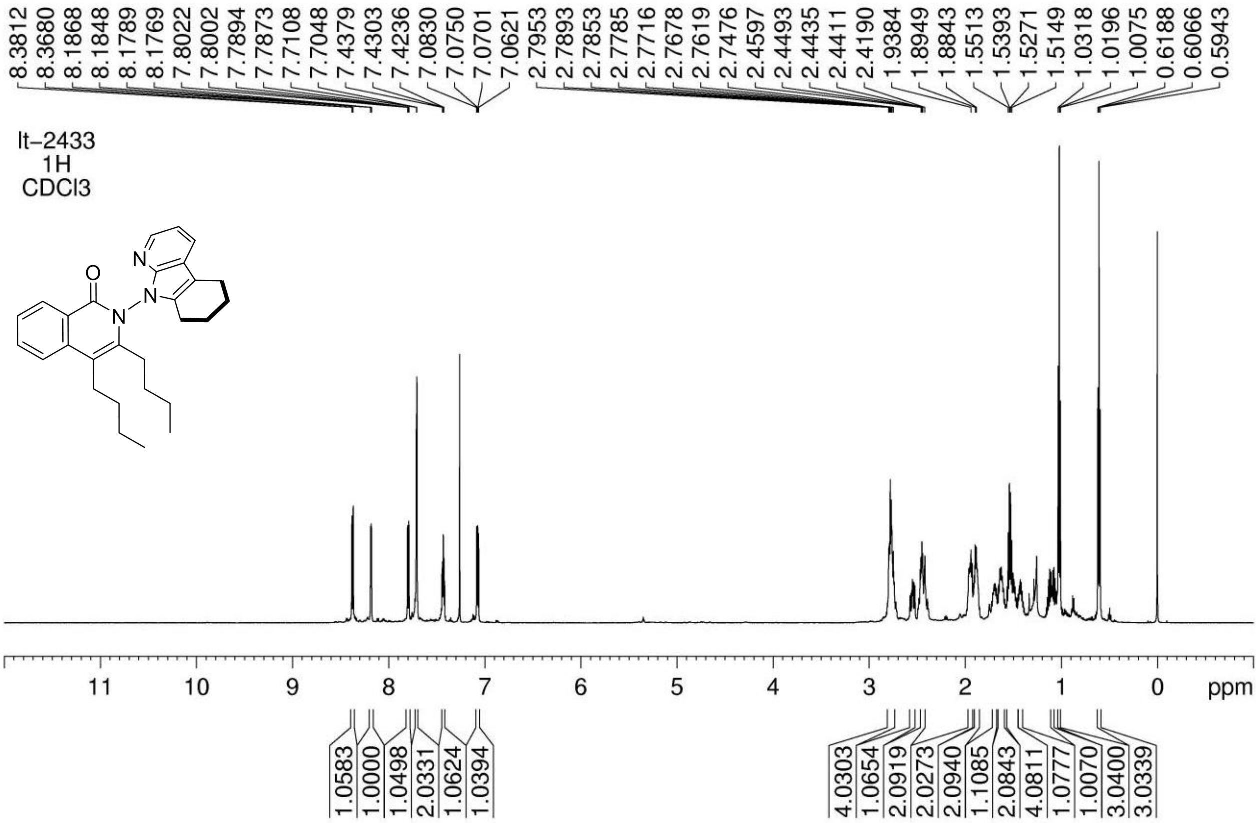

lt-2433
1H
CDCl3
8.3812
8.3680
8.1868
8.1848
8.1789
8.1769
7.8022
7.8002
7.7894
7.7873
7.7108
7.7048
7.4379
7.4303
7.4236
7.0830
7.0750
7.0701
7.0621
2.7953
2.7893
2.7853
2.7785
2.7716
2.7678
2.7619
2.7476
2.4597
2.4493
2.4435
2.4411
2.4190
1.9384
1.8949
1.8843
1.5513
1.5393
1.5271
1.5149
1.0318
1.0196
1.0075
0.6188
0.6066
0.5943
1.0583
1.0000
1.0498
2.0331
1.0624
1.0394
4.0303
1.0654
2.0919
2.0273
2.0940
1.1085
2.0843
4.0811
1.0777
1.0070
3.0400
3.0339
ppm

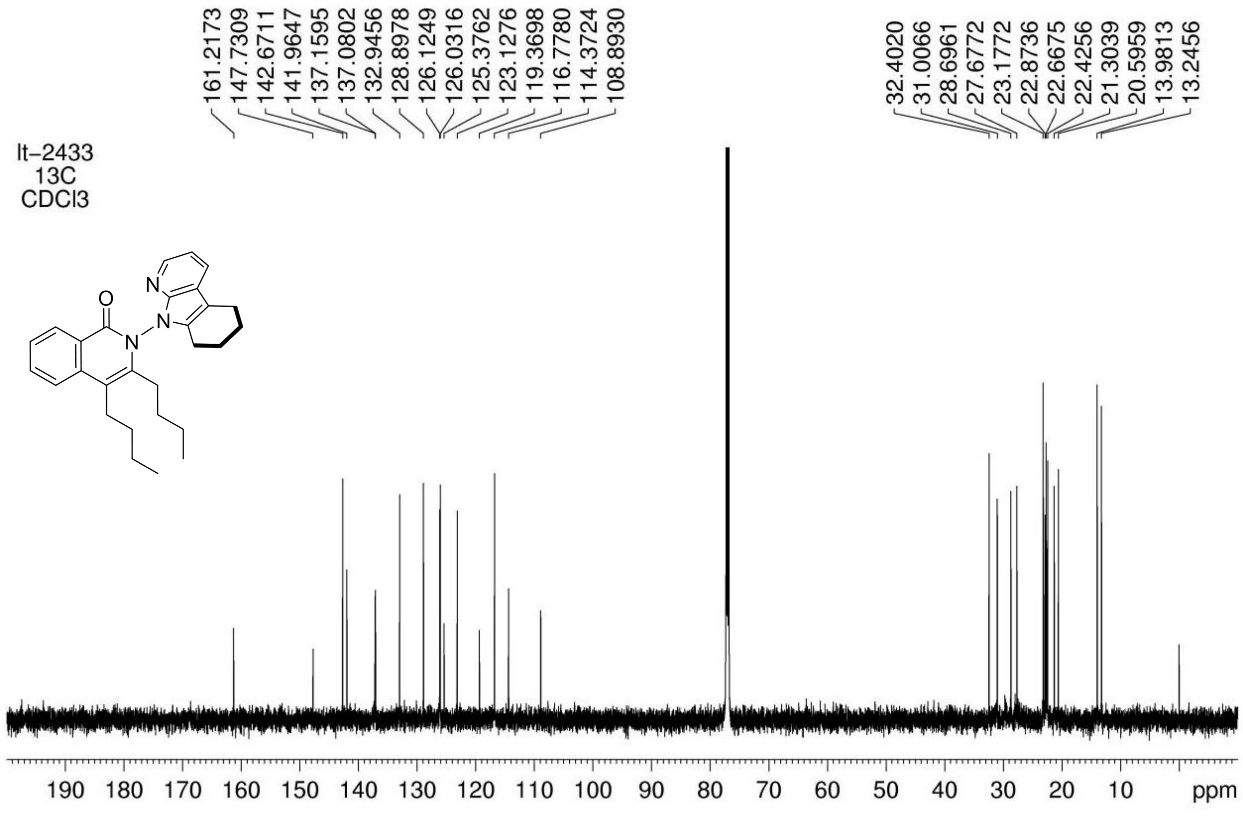

lt-2433
13C
CDCl3
161.2173
147.7309
142.6711
141.9647
137.1595
137.0802
132.9456
128.8978
126.1249
126.0316
125.3762
123.1276
119.3698
116.7780
114.3724
108.8930
32.4020
31.0066
28.6961
27.6772
23.1772
22.8736
22.6675
22.4256
21.3039
20.5959
13.9813
13.2456
ppm

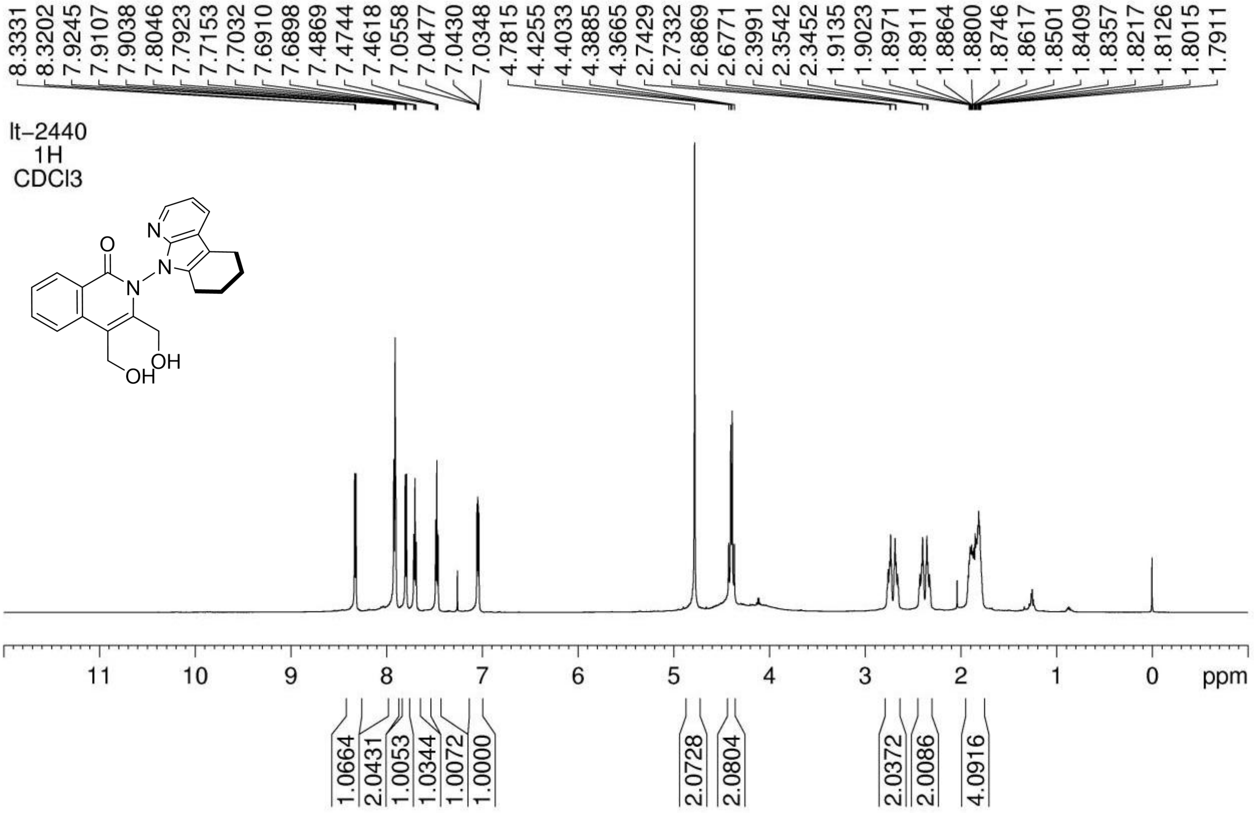

8.3331
8.3202
7.9245
7.9107
7.9038
7.8046
7.7923
7.7153
7.7032
7.6910
7.6898
7.4869
7.4744
7.4618
7.0558
7.0477
7.0430
7.0348
4.7815
4.4255
4.4033
4.3885
4.3665
2.7429
2.7332
2.6869
2.6771
2.3991
2.3542
2.3452
1.9135
1.9023
1.8971
1.8911
1.8864
1.8800
1.8746
1.8617
1.8501
1.8409
1.8357
1.8217
1.8126
1.8015
1.7911
lt–2440
1H
CDCl3
O
N
N
N
OH
OH
11 10 9 8 7 6 5 4 3 2 1 0 ppm
1.0664
2.0431
1.0053
1.0344
1.0072
1.0000
2.0728
2.0804
2.0372
2.0086
4.0916

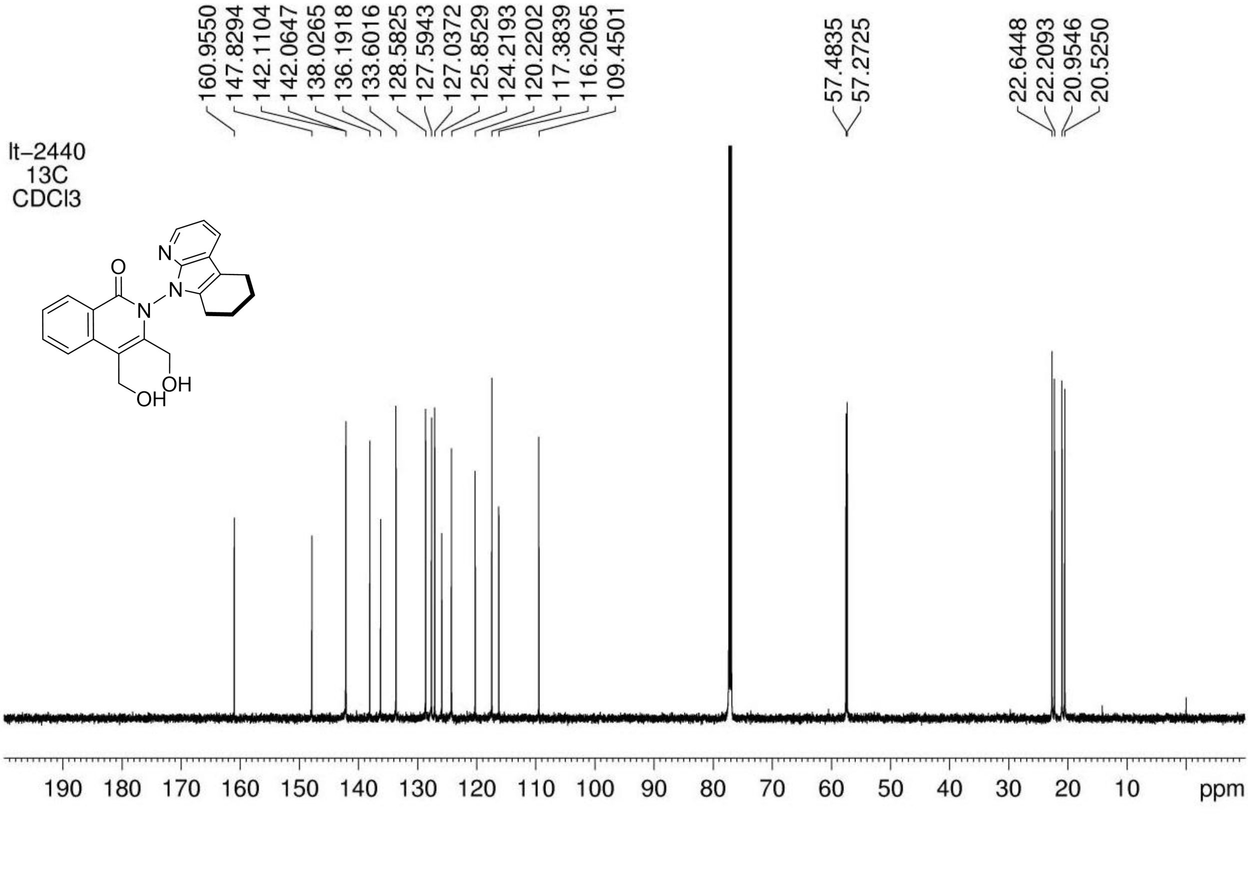

160.9550
147.8294
142.1104
142.0647
138.0265
136.1918
133.6016
128.5825
127.5943
127.0372
125.8529
124.2193
120.2202
117.3839
116.2065
109.4501
57.4835
57.2725
22.6448
22.2093
20.9546
20.5250
lt–2440
13C
CDCl3
O
N
N
N
OH
OH
190 180 170 160 150 140 130 120 110 100 90 80 70 60 50 40 30 20 10 ppm

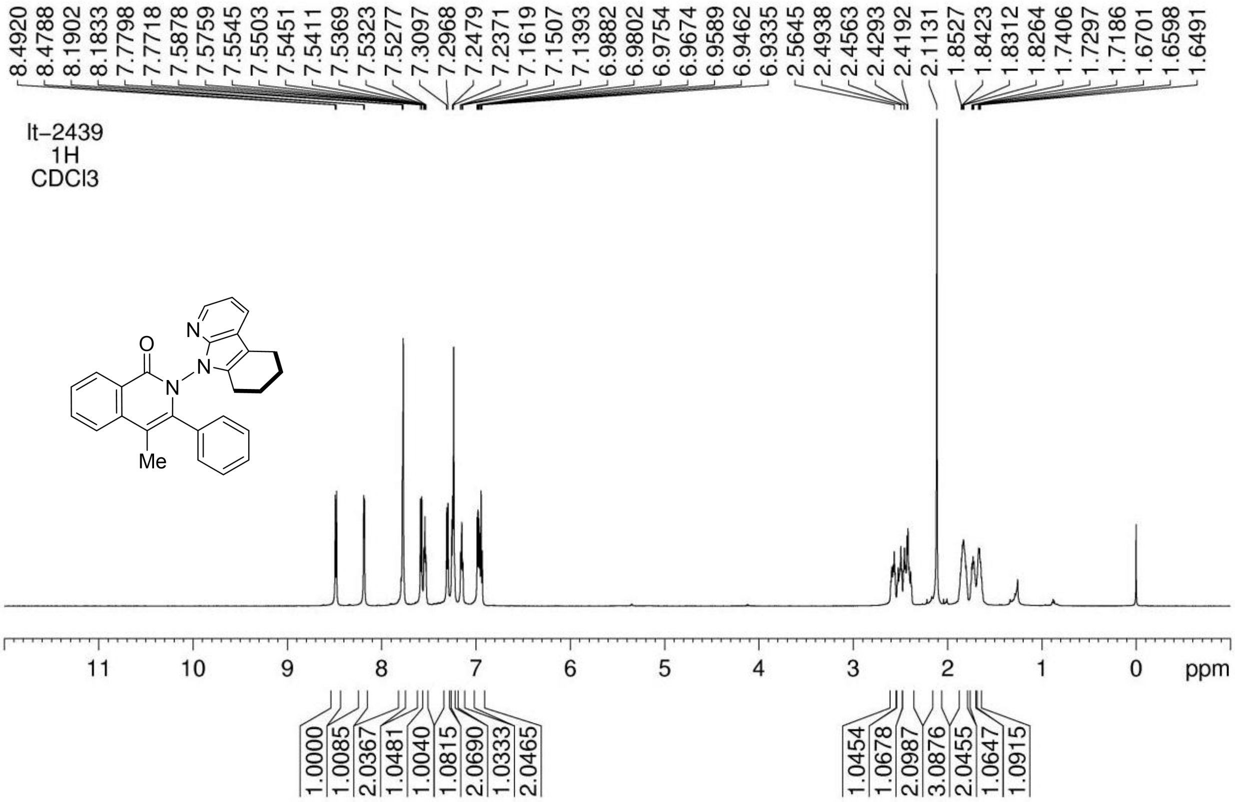

8.4920
8.4788
8.1902
8.1833
7.7798
7.7718
7.5878
7.5759
7.5545
7.5503
7.5451
7.5411
7.5369
7.5323
7.5277
7.3097
7.2968
7.2479
7.2371
7.1619
7.1507
7.1393
6.9882
6.9802
6.9754
6.9674
6.9589
6.9462
6.9335
2.5645
2.4938
2.4563
2.4293
2.4192
2.1131
1.8527
1.8423
1.8312
1.8264
1.7406
1.7297
1.7186
1.6701
1.6598
1.6491
lt−2439
1H
CDCl3
N
O
N
N
Me
11
10
9
8
7
6
5
4
3
2
1
0
ppm
1.0000
1.0085
2.0367
1.0481
1.0040
1.0815
2.0690
1.0333
2.0465
1.0454
1.0678
2.0987
3.0876
2.0455
1.0647
1.0915

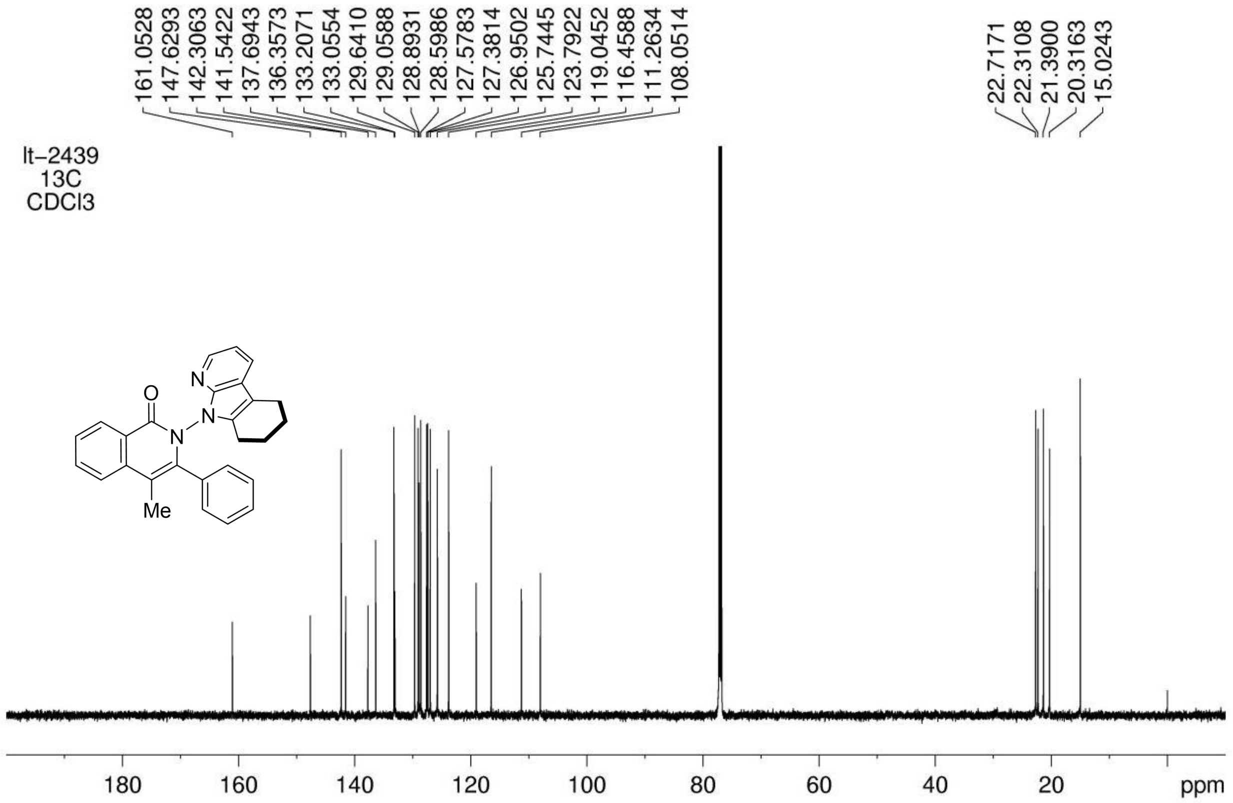

161.0528
147.6293
142.3063
141.5422
137.6943
136.3573
133.2071
133.0554
129.6410
129.0588
128.8931
128.5986
127.5783
127.3814
126.9502
125.7445
123.7922
119.0452
116.4588
111.2634
108.0514
22.7171
22.3108
21.3900
20.3163
15.0243
lt−2439
13C
CDCl3
N
O
N
N
Me
180
160
140
120
100
80
60
40
20
ppm

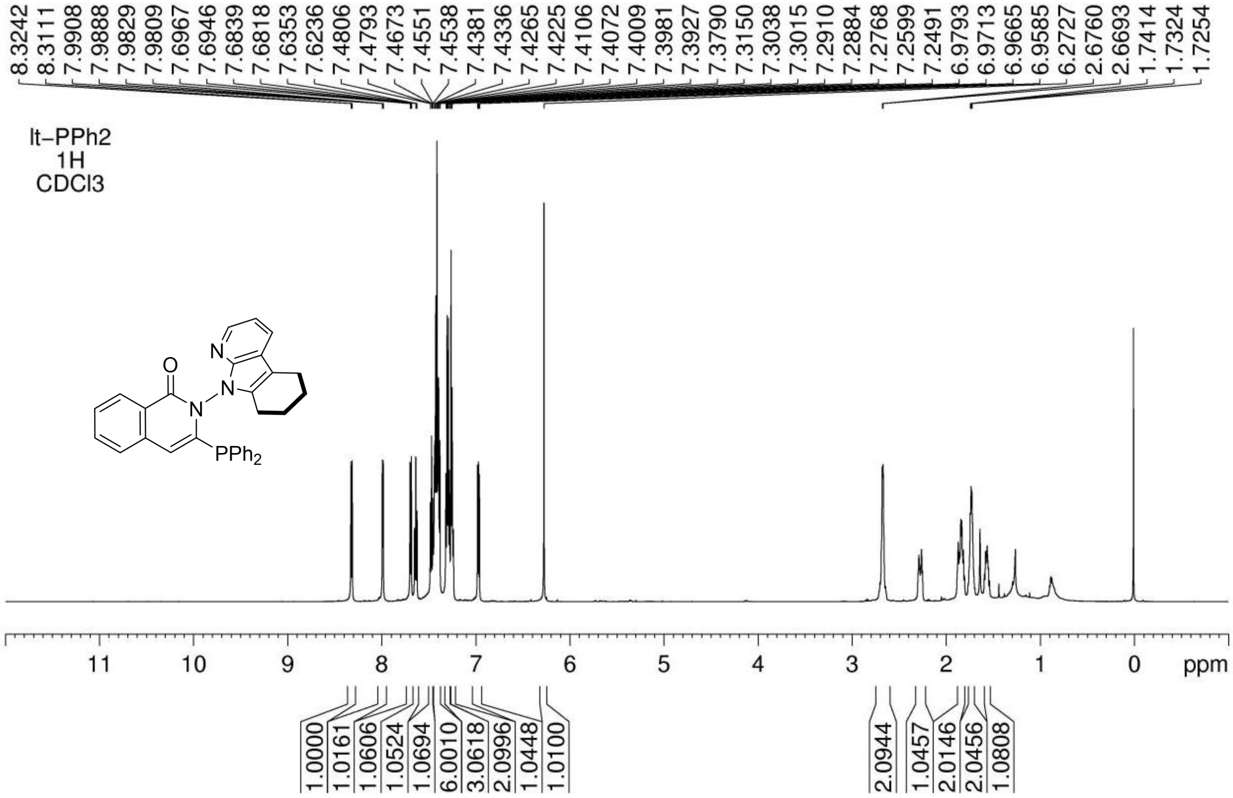
8.3242
8.3111
7.9908
7.9888
7.9829
7.9809
7.6967
7.6946
7.6839
7.6818
7.6353
7.6236
7.4806
7.4793
7.4673
7.4551
7.4538
7.4381
7.4336
7.4265
7.4225
7.4106
7.4072
7.4009
7.3981
7.3927
7.3790
7.3150
7.3038
7.3015
7.2910
7.2884
7.2768
7.2599
7.2491
6.9793
6.9713
6.9665
6.9585
6.2727
2.6760
2.6693
1.7414
1.7324
1.7254
lt–PPh2
1H
CDCl3
O
N
N
N
PPh2
11
10
9
8
7
6
5
4
3
2
1
0
ppm
1.0000
1.0161
1.0606
1.0524
1.0694
6.0010
3.0618
2.0996
1.0448
1.0100
2.0944
1.0457
2.0146
2.0456
1.0808

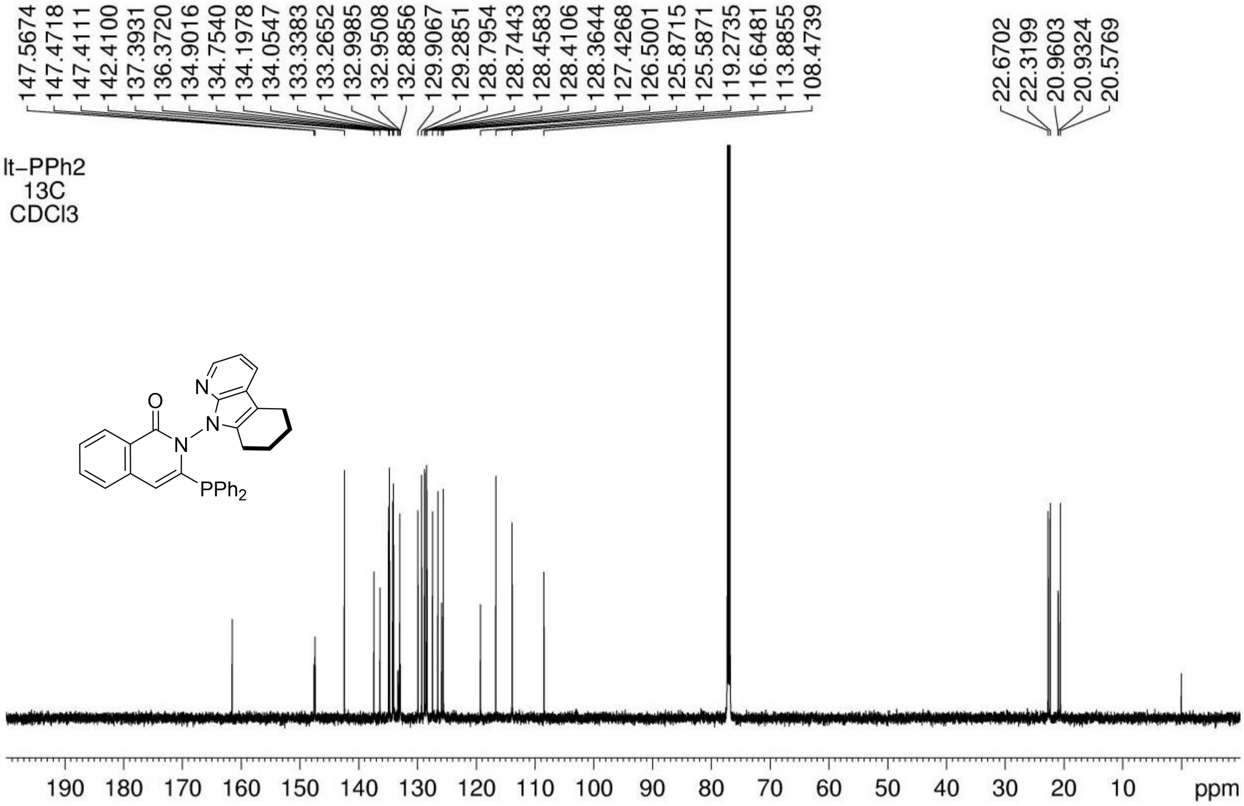
147.5674
147.4718
147.4111
142.4100
137.3931
136.3720
134.9016
134.7540
134.1978
134.0547
133.3383
133.2652
132.9985
132.9508
132.8856
129.9067
129.2851
128.7954
128.7443
128.4583
128.4106
128.3644
127.4268
126.5001
125.8715
125.5871
119.2735
116.6481
113.8855
108.4739
22.6702
22.3199
20.9603
20.9324
20.5769
lt–PPh2
13C
CDCl3
O
N
N
N
PPh2
190
180
170
160
150
140
130
120
110
100
90
80
70
60
50
40
30
20
10
ppm

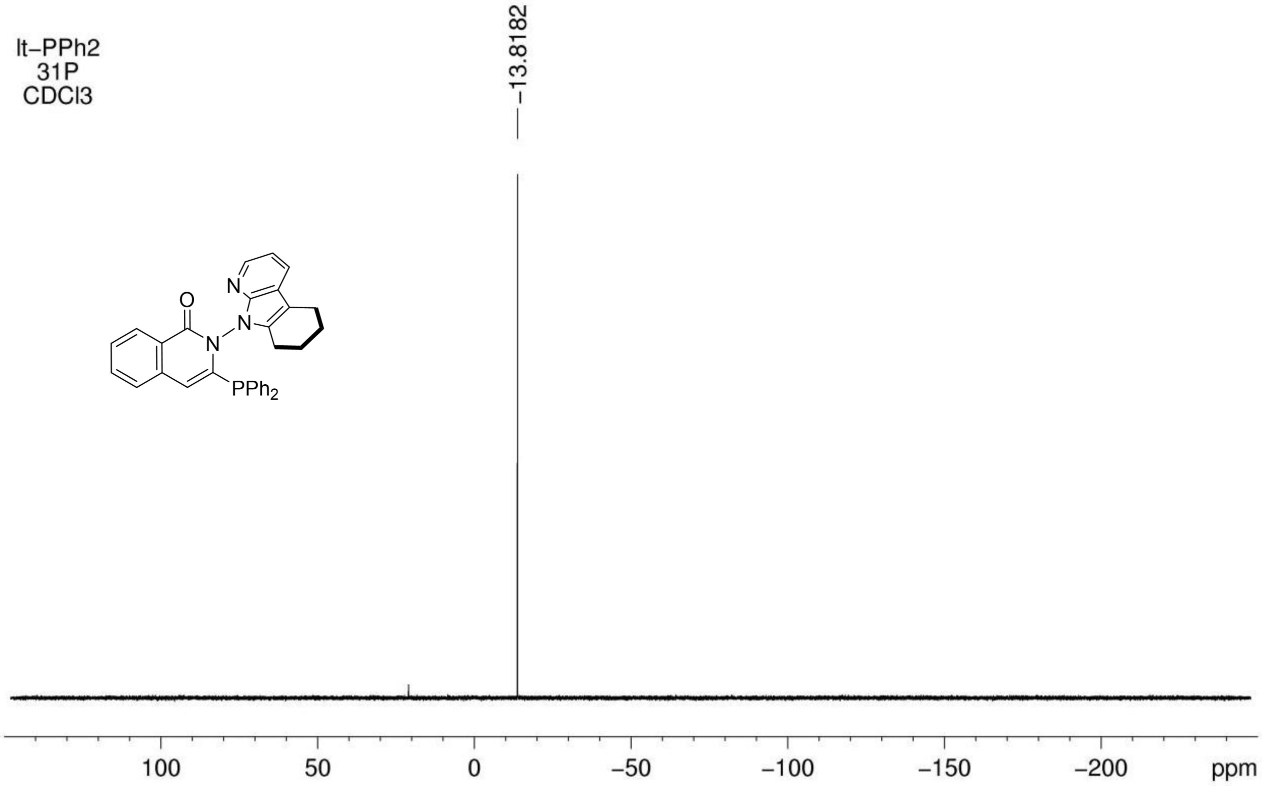
lt–PPh2
31P
CDCl3
–13.8182
O
N
N
N
PPh2
100
50
0
–50
–100
–150
–200
ppm

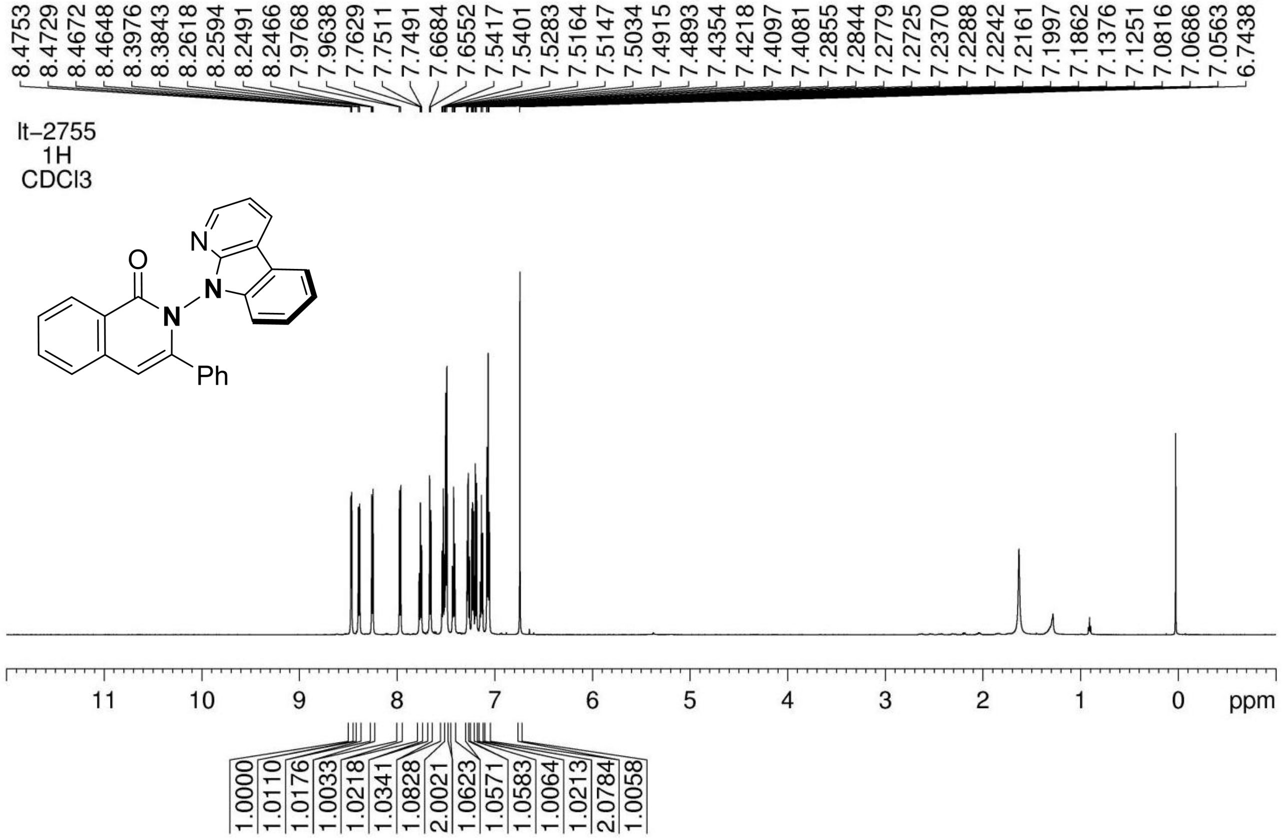
8.4753
8.4729
8.4672
8.4648
8.3976
8.3843
8.2618
8.2594
8.2491
8.2466
7.9768
7.9638
7.7629
7.7511
7.7491
7.6684
7.6552
7.5417
7.5401
7.5283
7.5164
7.5147
7.5034
7.4915
7.4893
7.4354
7.4218
7.4097
7.4081
7.2855
7.2844
7.2779
7.2725
7.2370
7.2288
7.2242
7.2161
7.1997
7.1862
7.1376
7.1251
7.0816
7.0686
7.0563
6.7438
lt−2755
1H
CDCl3
N
O
N
N
Ph
11 10 9 8 7 6 5 4 3 2 1 0 ppm
1.0000
1.0110
1.0176
1.0033
1.0218
1.0341
1.0828
2.0021
1.0623
1.0571
1.0583
1.0064
1.0213
2.0784
1.0058
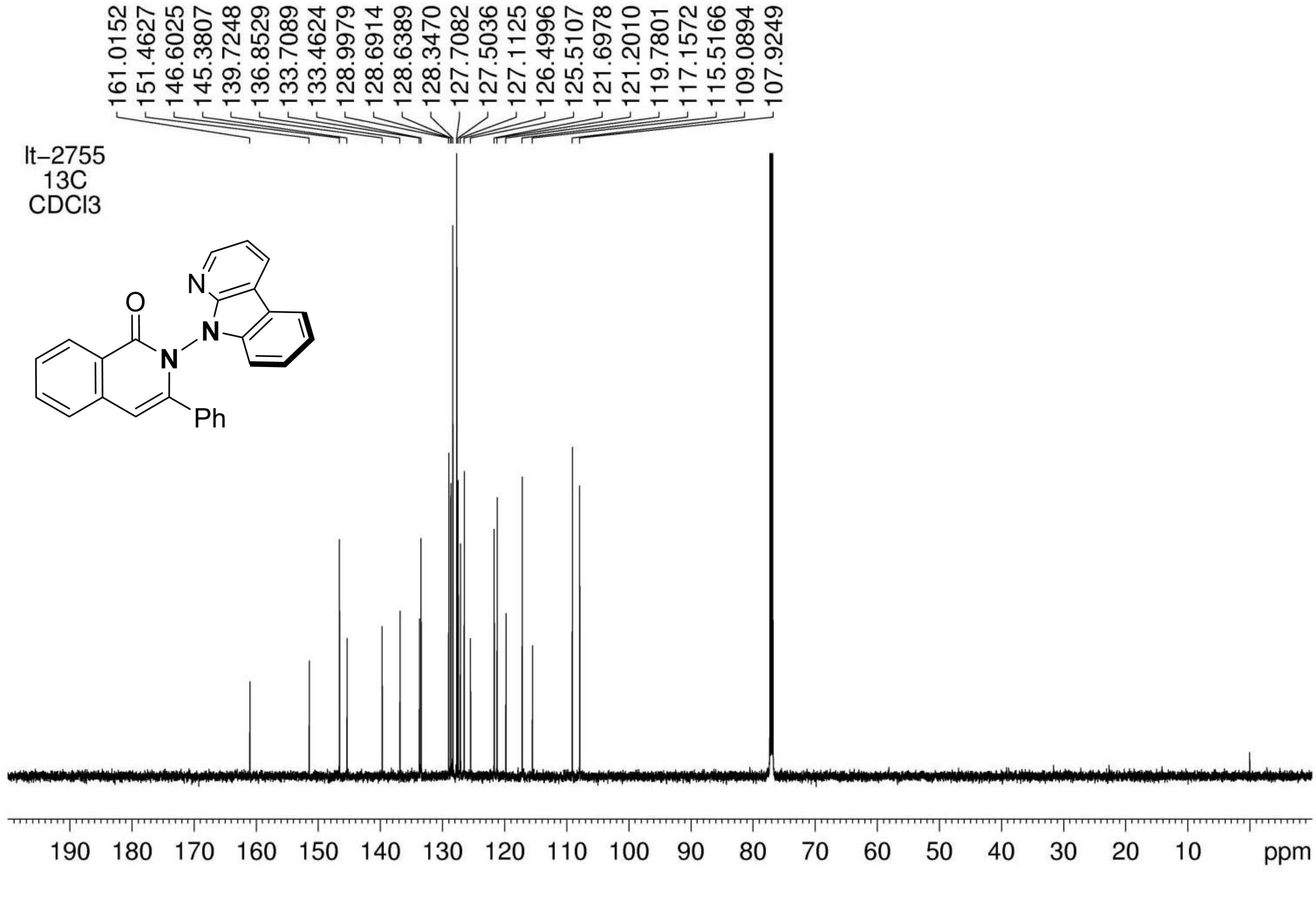
161.0152
151.4627
146.6025
145.3807
139.7248
136.8529
133.7089
133.4624
128.9979
128.6914
128.6389
128.3470
127.7082
127.5036
127.1125
126.4996
125.5107
121.6978
121.2010
119.7801
117.1572
115.5166
109.0894
107.9249
lt−2755
13C
CDCl3
N
O
N
N
Ph
190 180 170 160 150 140 130 120 110 100 90 80 70 60 50 40 30 20 10 ppm

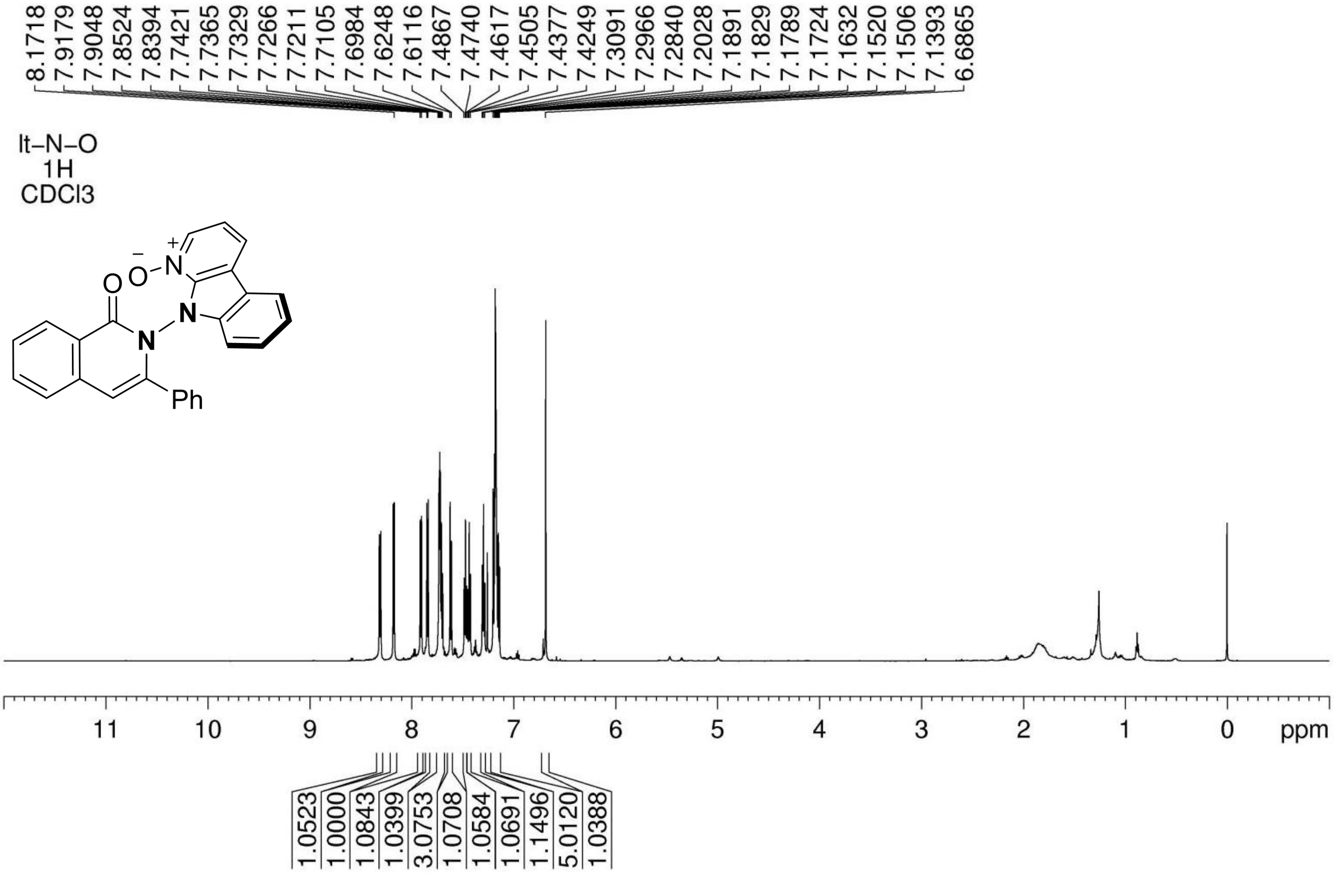
8.1718
7.9179
7.9048
7.8524
7.8394
7.7421
7.7365
7.7329
7.7266
7.7211
7.7105
7.6984
7.6248
7.6116
7.4867
7.4740
7.4617
7.4505
7.4377
7.4249
7.3091
7.2966
7.2840
7.2028
7.1891
7.1829
7.1789
7.1724
7.1632
7.1520
7.1506
7.1393
6.6865
It–N–O
1H
CDCl3
O
N
Ph
11 10 9 8 7 6 5 4 3 2 1 0 ppm
1.0523
1.0000
1.0843
1.0399
3.0753
1.0708
1.0584
1.0691
1.1496
5.0120
1.0388

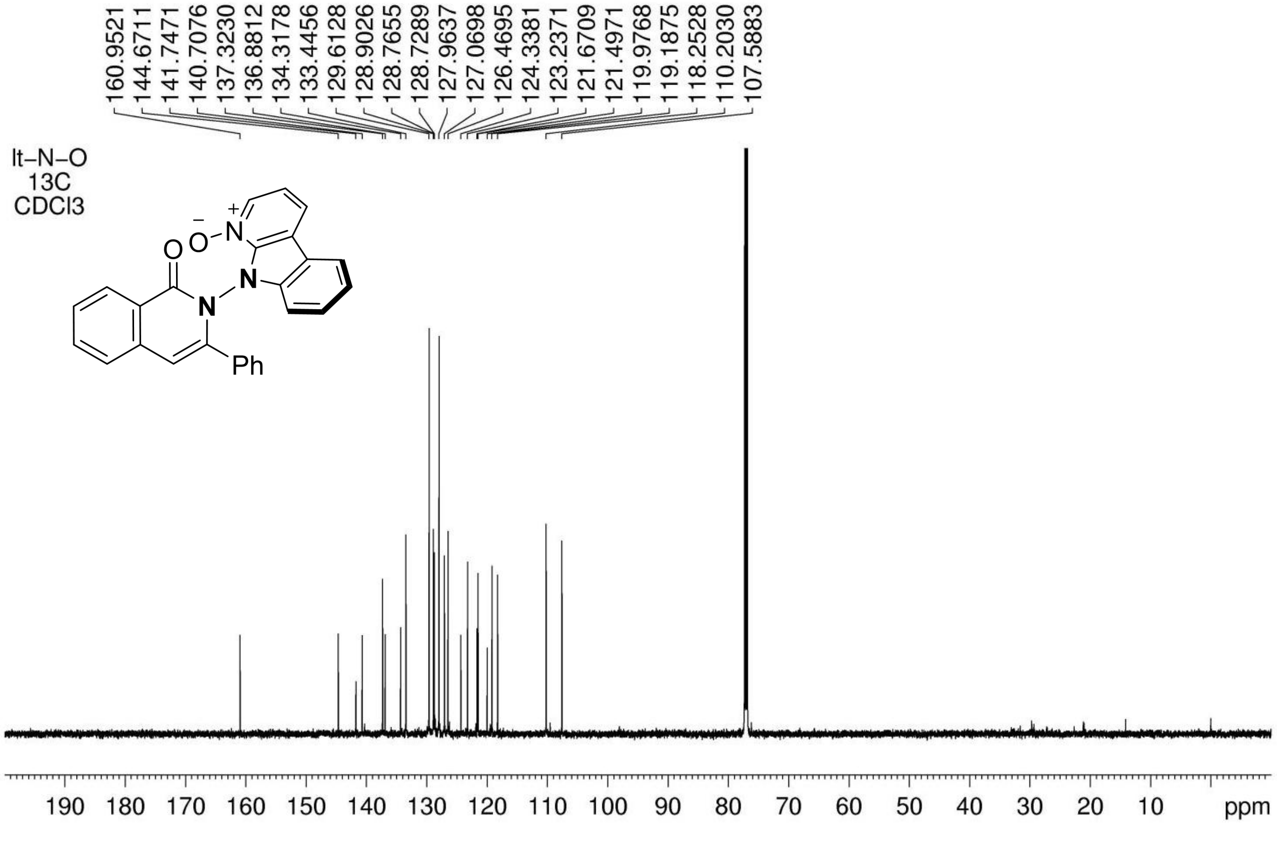
160.9521
144.6711
141.7471
140.7076
137.3230
136.8812
134.3178
133.4456
129.6128
128.9026
128.7655
128.7289
127.9637
127.0698
126.4695
124.3381
123.2371
121.6709
121.4971
119.9768
119.1875
118.2528
110.2030
107.5883
It–N–O
13C
CDCl3
O
N
Ph
190 180 170 160 150 140 130 120 110 100 90 80 70 60 50 40 30 20 10 ppm

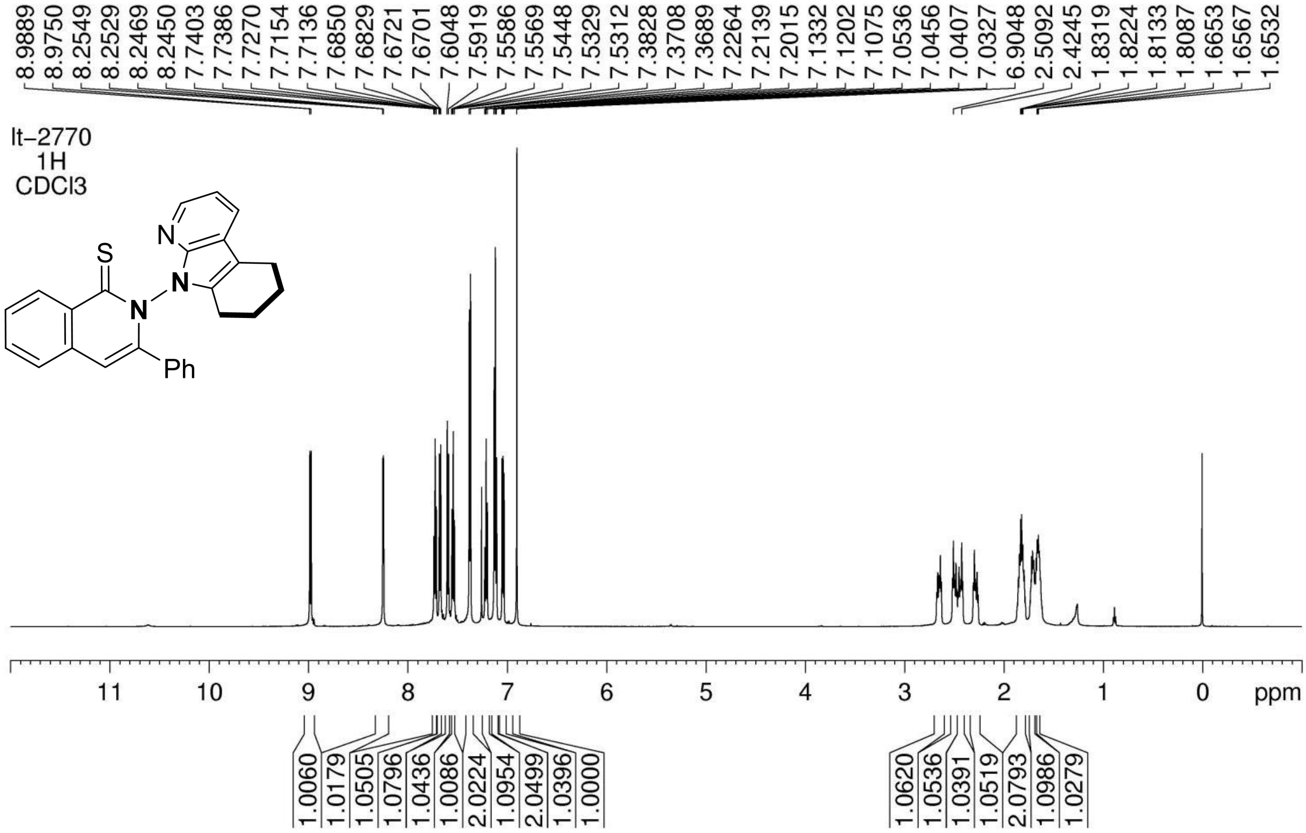

8.9889 8.9750 8.2549 8.2529 8.2469 8.2450 7.7403 7.7386 7.7270 7.7154 7.7136 7.6850 7.6829 7.6721 7.6701 7.6048 7.5919 7.5586 7.5569 7.5448 7.5329 7.5312 7.3828 7.3708 7.3689 7.2264 7.2139 7.2015 7.1332 7.1202 7.1075 7.0536 7.0456 7.0407 7.0327 6.9048 2.5092 2.4245 1.8319 1.8224 1.8133 1.8087 1.6653 1.6567 1.6532
lt–2770
1H
CDCl3
S
N
N
N
Ph
11 10 9 8 7 6 5 4 3 2 1 0 ppm
1.0060 1.0179 1.0505 1.0796 1.0436 1.0086 2.0224 1.0954 2.0499 1.0396 1.0000
1.0620 1.0536 1.0391 1.0519 2.0793 1.0986 1.0279

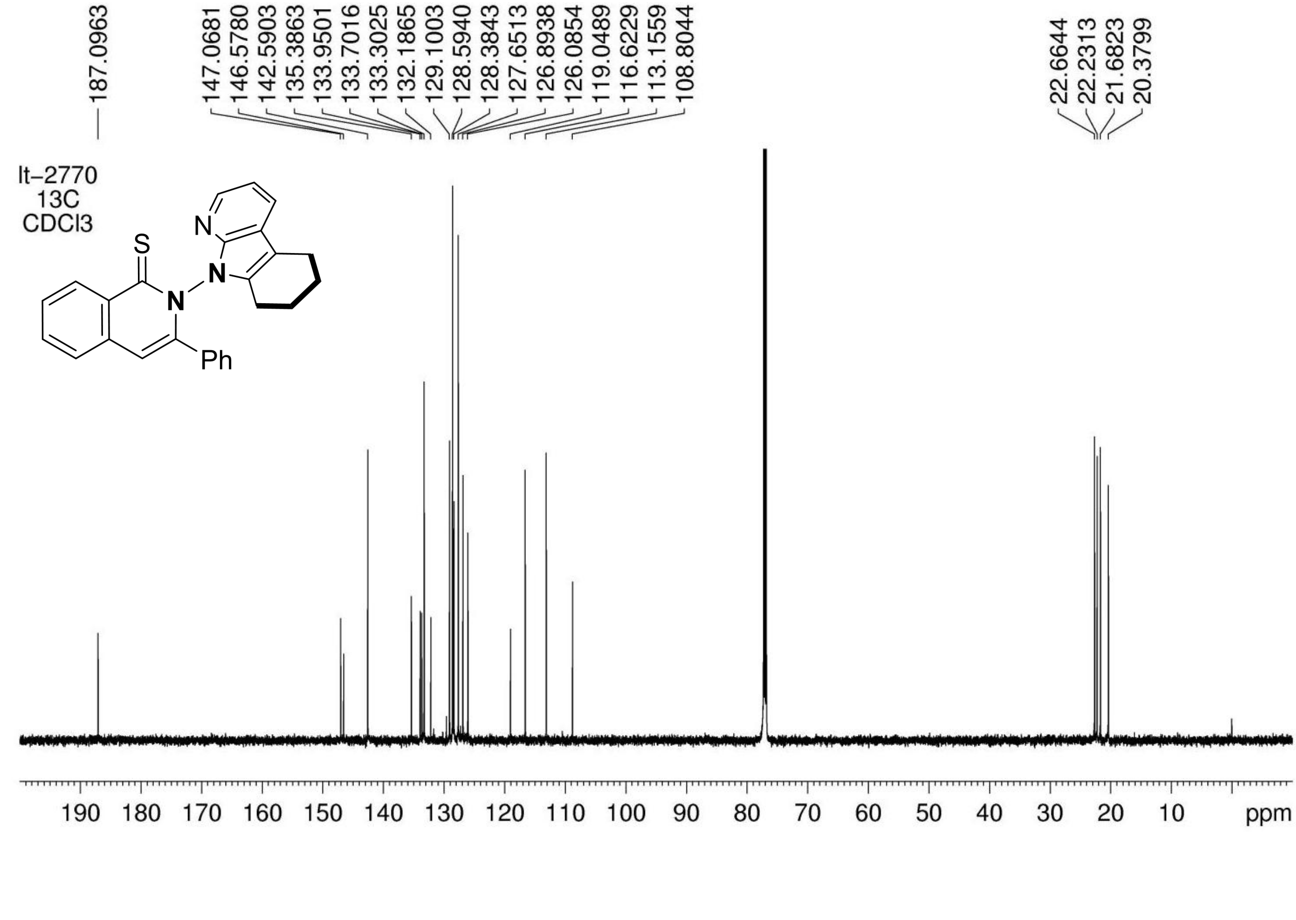

187.0963
147.0681 146.5780 142.5903 135.3863 133.9501 133.7016 133.3025 132.1865 129.1003 128.5940 128.3843 127.6513 126.8938 126.0854 119.0489 116.6229 113.1559 108.8044
22.6644 22.2313 21.6823 20.3799
lt–2770
13C
CDCl3
S
N
N
N
Ph
190 180 170 160 150 140 130 120 110 100 90 80 70 60 50 40 30 20 10 ppm

13. Supplementary references

1. Alekseyev, R. S., Amirova, S. R., Kabanova, E. V. & Terenin, V. I. The Fischer Reaction in the Synthesis of 2,3-Disubstituted 7-Azaindoles. *Chem. Heterocycl. Comp.* **50**, 1305-1315 (2014).
2. Yan, J., Ni, T. & Yan, F. Simple and efficient procedures for selective preparation of 3-haloindoles and 2,3-dihaloindoles by using 1,3-dibromo-5, 5-dimethylhydantoin and 1,3-dichloro-5,5-dimethylhydantoin. *Tetrahedron Lett.* **56**, 1096-1098 (2015).
3. Xia, W. et al. Chiral Phosphoric Acid Catalyzed Atroposelective C−H Amination of Arenes. *Angew. Chem. Int. Ed.* **59**, 6775–6779 (2020).
4. Mino, T. et al. Axially chiral *N*-alkyl-*N*-cinnamoyl amide type P, olefin ligands for Pd-catalyzed reactions. *Org. Biomol. Chem.* **21**, 2775-2778 (2023).
5. Zhu, X. et al. Rhodium-Catalyzed Annulative Approach to N-N Axially Chiral Biaryls via C-H Activation and Dynamic Kinetic Transformation. *Chem. Sci.* DOI: 10.1039/D3SC02800C.
6. Raut, V. S. et al. Enantioselective Syntheses of Furan Atropisomers by an Oxidative Central-to-Axial Chirality Conversion Strategy. *J. Am. Chem. Soc.* **139**, 2140–2143 (2017).
7. Liu, Z.-S. et al. Construction of axial chirality via palladium/chiral norbornene cooperative catalysis. *Nat. Catal.* **3**, 727–733 (2020).
8. Liu, Z.-S. et al. An axial-to-axial chirality transfer strategy for atroposelective construction of C–N axial chirality. *Chem* **7**, 1917–1932 (2021).
9. Frisch, M. J., Trucks, G. W., Schlegel, H. B., Scuseria, G. E., Robb, M. A., Cheeseman, J. R., Scalmani, G., Barone, V., Mennucci, B., Petersson, G. A., Nakatsuji, H., Caricato, M., Li, X., Hratchian, H. P., Izmaylov, A. F., Bloino, J., Zheng, G., Sonnenberg, J. L., Hada, M., Ehara, M., Toyota, K., Fukuda, R., Hasegawa, J., Ishida, M., Nakajima, T., Honda, Y., Kitao, O., Nakai, H., Vreven, T., Montgomery, J. A., Jr., Peralta, J. E., Ogliaro, F., Bearpark, M., Heyd, J. J., Brothers, E., Kudin, K. N., Staroverov, V. N., Kobayashi, R., Normand, J., Raghavachari, K., Rendell, A., Burant, J. C., Iyengar, S. S., Tomasi, J., Cossi, M., Rega, N., Millam, J. M., Klene, M., Knox, J. E., Cross, J. B., Bakken, V., Adamo, C., Jaramillo, J., Gomperts, R., Stratmann, R. E., Yazyev, O., Austin, A. J., Cammi, R., Pomelli, C., Ochterski, J. W., Martin, R. L., Morokuma, K., Zakrzewski, V. G., Voth, G. A., Salvador, P., Dannenberg, J. J., Dapprich, S., Daniels, A. D., Farkas, O., Foresman, J. B., Ortiz, J. V., Cioslowski, J. & Fox, D. J. Gaussian 09, Revision A.02, Gaussian, Inc.: Wallingford CT, 2009.
10. Zhao, Y. & Truhlar, D. G. The M06 suite of density functionals for main group thermochemistry, thermochemical kinetics, non-covalent interactions, excited states, and transition elements: two new functionals and systematic testing of four M06-class functionals and 12 other functionals. *Theor. Chem. Acc.* **120**, 215−241 (2008).
11. Hariharan P. C. & Pople, J. A. The influence of polarization functions on molecular orbital hydrogenation energies. *Theor. Chim. Acta.*, **28**, 213-222 (1973).
12. Francl, M. M. et al. Self-consistent molecular orbital methods. XXIII. A polarization-type basis set for second-row elements. *J. Chem. Phys.* **77**, 3654-3665 (1982).
13. Marenich. A. V., Cramer. C. J. & Truhlar, D. G. Universal Solvation Model Based on Solute Electron Density and on a Continuum Model of the Solvent Defined by the Bulk Dielectric Constant and Atomic Surface Tensions. *J. Phys. Chem. B.* **113**, 6378 (2009).
14. Krishnan, R., Binkley, J. S., Seeger R. & Pople, J. A. Self-consistent molecular orbital methods. XX. A basis set for correlated wave functions. *J. Chem. Phys.* **72**, 650-654 (1980).
15. Clark, T., Chandrasekhar, J., Spitznagel G. W. & Schleyer, P. V. Efficient diffuse function-augmented basis sets for anion calculations. III. The 3-21+G basis set for first-row elements, Li–F. *J. Comput. Chem.* 4, 294-301

(1983).
16. Legault C Y. CYLview, 1.0b. Université de Sherbrooke, 2009: http://www.cylview.org.
