# Supplementary material for: Cobalt-catalyzed atroposelective C−H activation/annulation to access N−N axially chiral frameworks
Source: Nat Commun. 2023 Aug 29;14:5271. doi: 10.1038/s41467-023-40978-4 (PMC10465517; doi:10.1038/s41467-023-40978-4)
Supplement: Supplementary file 3 — Description of Additional Supplementary Files [file 41467_2023_40978_MOESM3_ESM.pdf]

Description of Additional Supplementary Files

File Name: Supplementary Data 1

Description: Cartesian coordinates of the calculated structures
